# Association Complexes of Calix[6]arenes with Amino Acids Explained by Energy-Partitioning Methods

**DOI:** 10.3390/molecules27227938

**Published:** 2022-11-16

**Authors:** Emran Masoumifeshani, Michał Chojecki, Dorota Rutkowska-Zbik, Tatiana Korona

**Affiliations:** 1 Faculty of Chemistry, University of Warsaw, ul. Pasteura 1, 02-093 Warsaw, Poland; 2 Jerzy Haber Institute of Catalysis and Surface Chemistry, Polish Academy of Sciences, ul. Niezapominajek 8, 30-239 Cracow, Poland

**Keywords:** calixarene, SAPT, molecular fragmentation, intermolecular interaction, hydrogen bond, host–guest complex

## Abstract

Intermolecular complexes with calixarenes are intriguing because of multiple possibilities of noncovalent binding for both polar and nonpolar molecules, including docking in the calixarene cavity. In this contribution calix[6]arenes interacting with amino acids are studied with an additional aim to show that tools such as symmetry-adapted perturbation theory (SAPT), functional-group SAPT (F-SAPT), and systematic molecular fragmentation (SMF) methods may provide explanations for different numbers of noncovalent bonds and of their varying strength for various calixarene conformers and guest molecules. The partitioning of the interaction energy provides an easy way to identify hydrogen bonds, including those with unconventional hydrogen acceptors, as well as other noncovalent bonds, and to find repulsive destabilizing interactions between functional groups. Various other features can be explained by energy partitioning, such as the red shift of an IR stretching frequency for some hydroxy groups, which arises from their attraction to the phenyl ring of calixarene. Pairs of hydrogen bonds and other noncovalent bonds of similar magnitude found by F-SAPT explain an increase in the stability of both inclusion and outer complexes.

## 1. Introduction

Calixarenes are phenolic-based macrocycles consisting of repeated units of phenol and hydrocarbon groups, which have attracted a lot of attention in recent years owing to their ability to form host–guest complexes for hydrophobic guests, in particular, in the context of their biological and pharmaceutical potential [1,2,3]. Experimentally known calixarenes usually contain between four and eight phenol units [4]. The simplest calixarene, calix[4]arene, consists of four phenol groups connected through methylene links (–CH_2_–). A theoretical study of the stability of smaller calix[3]arenes [5] revealed strong structure stress, which disappears only when one more phenol ring is added to the macrocycle. A known feature of calixarenes is their ability to form inclusion complexes in one conformation and release the guest molecule after the conformation change. Such adducts, which are examples of intermolecular complexes, are being studied as possible drug carriers [6,7,8], models of enzyme active centers [4], the base for supramolecular catalysts [9,10], building elements of nonlinear optics structures [11], structures binding metal ions [5,12] and their clusters [13], and as chemosensors [14,15,16]. Furthermore, the modification of the calixarenes’ basic core and both upper and lower rims extended the range of their applications towards becoming anticancer drugs [17,18,19], antimicrobial agents [17,20,21,22,23,24,25], and sensitizers for photodynamic therapy [26].

**Figure 1:**
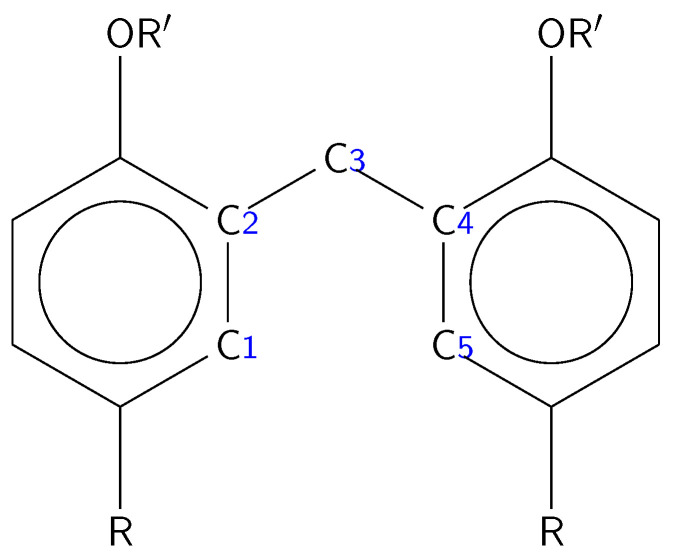
Dihedral angles between adjacent aryl groups of a calixarene. The first dihedral angle is defined through atoms 1–4, and the second one–through atoms 2–5.

The smallest stable calixarene is known to have four conformations, which can be unanimously described by just listing relative orientations of the phenol groups with respect to the macroring, i.e., since each phenol ring can assume either upward (*u*) or downward (*d*) position relative to this ring, one finds four possibilities, denoted as *cone* (uuuu), *partial cone* (uuud), *1,2-alternate* (uudd), and *1,3-alternate* (udud)–in parenthesis, the arrangement of adjacent phenol rings has been listed. This classification turns out to be insufficient for larger calixarenes, where additional degrees of freedom result from more variants of orientations of phenol rings with respect to their left and right neighbors. In order to characterize these calixarene conformers, Ugozzoli and Andreetti [27] proposed to represent a conformation of calix[*n*]arene through a list of *n* pairs of conformational parameters, defined as dihedral angles between a phenol ring and a plane defined by two C-C bonds originating from a linker carbon atom towards phenol groups. Formally these angles are calculated as dihedral angles $C_{1}C_{2}C_{3}C_{4}$ (ϕ) and $C_{2}C_{3}C_{4}C_{5}$ (χ), see Figure 1 for numbering of carbon atoms. Additionally, a “top side” of calixarene is defined in Ref. [27], and the order of (ϕ,χ) descriptors is set to be counterclockwise with respect to the top. For all cases considered in the present paper, the simplest definition of the top side applies, i.e., it is the side with the highest number of phenolic oxygen atoms. Some of these conformations are more stabilized by hydrogen bonds (H-bonds) between the hydroxy groups than others. Moreover, the cavity size and the rim properties differ significantly in various conformers. The conformations described above are in dynamic equilibrium, which is related to the possibility of rotation around the bonds bridging the individual phenol units. This rotation may be blocked by the introduction of large substituents into the aromatic rings, which will form a steric hinge within the upper edge of the calixarene ring (opposite to the hydroxyl groups) [28]. Other factors affecting the possibility of rotation of phenol units are: temperature and solvents. The availability of conformers in a finite temperature is governed by their energetic stability differences according to the Boltzmann law, while polar or nonpolar solvents can either preferably bind with the polar hydroxy groups of calixarenes or–on the opposite–stabilize conformers for which hydroxy groups can be hidden inside the hole creating the intramolecular H-bond network(s).

Many theoretical and experimental studies exist for calixarenes, especially for the smallest calix[4]arenes, see, e.g., Refs. [29,30,31]. Studies of larger calixarenes, calix[6]arenes and calix[8]arenes as reported in Refs. [29,32,33] and [29,34,35], respectively, revealed that in the majority of cases, the cone conformation is the most stable. This finding can be explained by the creation of intramolecular H-bonds, as proposed by Gassoumi et al. [29] after a density-functional theory (DFT) study with several functionals for six- and eight-member unsubstituted calixarenes, while Furer et al. [32,34] arrived at the same conclusion for heavily substituted calix[*n*]arenes, n=6,8, with adamantane and *tert*-butyl serving as substituents.

Inclusion complexes with calixarenes were objects of several studies, too. For instance, Puchta et al. [36] examined the possibility of the binding of small molecules such as CH_3_NH_2_ and CH_3_CN by calix[4]arenes by employing a simple DFT functional and a small basis set as an extension of the nuclear magnetic resonance (NMR) studies. They found that no proper inclusion complexes are created since the most favorable position of the guest molecules would be on the outer part of the edge. The binding energy was small (5 kcal/mol), but one should take into account that the utilized functional and basis set were not optimal for such an investigation. It should be also noted that Puchta et al. observed a proton transfer from the host species after binding of CH_3_NH_2_. In another study Galindo-Murillo et al. [6] examined the applicability of calix[*n*]arenes (where n=4,5,6,8) as hosts for 3-phenyl-1H-[1]benzofur[3,2-c]pyrasol (GTP). Their DFT calculations (with a better B97-D functional) showed that the most promising GTP carriers are the two largest calixarenes, which can be attributed to the existence of a large enough space inside the host molecule and to the presence of hydrogen bondings between the host and guest. The stabilizing role of a partial charge transfer (CT) between GTP and calixarene has also been noted in this work.

The usability of calix[4]rene and calix[4]resorcinarene and their derivatives to host a zoledronate acid molecule has been studied by Jang et al. [37] with DFT plus a dispersion correction (DFT+D), where the cone conformer of calixarene has been used. It turned out that the adduct contains a guest attached to the upper ring edge of the calixarene and that the inclusion of this guest causes only modest changes in the cavity of the host. If calixarene is modified with the sulfone groups or with phosphone ones, their cavities become notably wider, while the bottom remains mostly rigid, as it is stabilized by the intramolecular H-bonds. Further analysis of electronic structures shows that π–π interactions play a decisive role in the binding between the host and the guest. The number of H-bonds is important for the relative stability of similar complexes, e.g., the binding with calix[4]resorcinarene is stronger than that of the sulfonated calix[4]arene. In another study performed by Kryuchkova et al. [38], it has been found that calix[4]arenes modified by the R_2_PO groups can ligate up to two cobalt complexes with the nitrate ions. The smallest calix[4]arenes were also studied as carriers of small gas molecules, such as H_2_, O_2_, N_2_, H_2_O, CO_2_, NH_3_, H_2_S, N_2_O, HCN, SO_2_ [39], if the lower rim of the host has been methylated, either fully or in part. The calculations, performed with the DFT+D approach, revealed that one can modify the selectivity of calixarenes through their methylation. Very recently, a theoretical study on the docking of insulin on calixarenes have appeared, where the CHARMM force field has been applied to determine the preferred amino acids docking to substituted calixarenes [40]. Other investigations of docking on calixarenes have been performed, e.g., in Refs. [41,42].

General studies of the stability of calixarenes usually do not delve into the nuances of the nature of differences between various conformers and/or complexes with calixarenes. It is usually assumed in the literature that binding properties of calixarenes can be explained by the interactions of the CH-π, π-π, and ion-π types [2,43], but–to our knowledge–no systematic studies exist, which would explain the variety of properties of calixarene conformers and their complexes through the application of energy partitioning approaches in order to classify interaction types and to estimate their relative strength. Therefore, in this paper, we would like to fill this gap and present such partitioning analyses resulting from Symmetry-Adapted Perturbation Theory (SAPT) [44,45], Functional-group SAPT (F-SAPT) [46,47], and our recent modification of Systematic Molecular Fragmentation (SMF) [48], described in Refs. [49,50] and denoted as Symmetrized SMF (SSMF). Our study will include a systematic search for the H-bonds and other bonding types within the complexes. The recent redefinition of the H-bond is presented in Ref. [51], and numerous investigations concerning various types of H-bonds have been performed, including “unusual” ones (dihydrogen bonds, bonds including unusual hydrogen donors–such as, e.g., C−H, or unusual hydrogen acceptors–such as, e.g., benzene ring, or inverted H-bonds, etc.), see, e.g., Refs. [52,53,54,55,56,57,58,59]. Such investigations have been performed for other complexes with SAPT (see, e.g., Ref. [52]) or F-SAPT methods [46,47]. Additionally, applications of the SMF to intermolecular interaction energy partitionings or comparisons of F-SAPT and SMF approaches are missing in the literature.

We selected standard amino acids as potential guests and calix[6]arenes as hosts for our study because of the biochemical importance and a wide variety of binding sites of potential guests, which result from hydrophobic, polar, aliphatic, aromatic, etc. groups attached to the amino acid moiety, while the choice of larger calix[6]arenes instead of calix[4]arenes was dictated by the existence of a bigger cavity, in which larger molecules are able to fit. Therefore, the selected set of intermolecular complexes is expected to provide examples of noncovalent bonds of different types and strengths and to show the competition of various bonds under steric constraints, resulting from the existence of a cavity. An additional feature, which will be tracked in this study, is the confinement effect resulting in the enhancement of the attraction. Such cases, reported many times for endohedral complexes of fullerenes [60], appear if a guest molecule has an optimal size for a given cavity and can be attracted from many sides, while a larger molecule starts to feel a repulsion from the cavity wall, and finally a smaller molecule is attracted mostly by one side of the cavity or–if placed in the center–is attracted less strongly because of too large a distance between the guest and the closest atoms of the host. The calixarenes selected for this study are: unsubstituted calix[6]arene and hexa-*p*-*tert*-butylcalix[6]arene, where the latter has been added in order to examine how obstacles created by spatially large nonpolar groups modify host–guest interactions. The *tert*-butyl group is one of the smallest and one of the most popular groups of this type and it is frequently used to decorate calixarene molecules [61]. In the following, these two species will be abbreviated as CX and BCX, respectively.

In the literature, one finds reports on the use of calixarenes for recognition of amino acids [62,63,64,65,66,67,68,69,70,71,72]. The critical review of the available data enabled general observations on amino acid complexation by calixarenes. Usually, the ability to bind these small guests decreases in the order: calix[6]arene > calix[8]arene > calix[4]arene [70,73]. The binding process is controlled by hydrogen bonding, van der Waals, cation–π, and π –π stacking interactions [70]. The strength of the interaction depends strongly on the chemical character of the amino acid and the substituents introduced to the pristine calixarene scaffold. Stone et al. [73] studied the formation of complexes of calixarenes with amino acids with matrix-assisted laser desorption/ionization coupled with mass spectrometer (MALDI-MS) technique. They demonstrated that calix[6]arenes substituted by esters form stable complexes with amino acids which are bound mostly by cation–π interactions and hydrogen bondings. The native calix[6]arenes, on the contrary, do not form complexes stable enough in the gas phase to withstand a relatively energetic process of MALDI. Among the association complexes, one finds both: (i) outer-sphere ones, in which amino acids are located outside the pocket created by the phenyl rings (see, e.g., Ref. [66]); and (ii) typical host–guest structures, where the amino acid molecules are buried inside the calixarene cavity (see, e.g., results of Oshima et al. [74] for complexes between hydrophobic amino acids and calix[6]arenes modified by carboxylic groups). Due to the small size of the interacting moieties, often one calixarene species accommodates only one amino acid molecule, but the ratio increases while increasing the size of calixarene. Douteau-Guével and co-workers demonstrated by the microcalorimetry method that calix[6]arene sulfonates form weak 1:1 complexes with Lys and Arg in water [75]. The same stoichiometry was also determined for the host–guest type of binding of Trp by calix[6]arene carboxylic acid derivative [76]. The 1:1 and 1:2 complexes are found in the case of calix[8]arenes [77].

## 2. Methodology

### 2.1. Geometry Optimization–Calixarenes

The first step in the analysis of complexes is the selection of the most suitable conformers for the calixarene host. In order to facilitate this task, we performed a query in the Cambridge Structural Database (CSD) [78] for accessible calix[6]arene structures. This search resulted in three selected conformers: pinched-cone (CSD-VARGUR [79]) – found as the most stable in nonhydrogen-bonding solvents [80], 1,2,3-alternate (CSD-REGWIM [81]) – preferred in hydrogen-bonding solvents [80], and recently crystalized winged-cone one (CSD-REGWEI [81]). Starting geometries of these conformations were extracted from the corresponding crystal structures, and several starting geometries were generated with the Tinker program [82] by freezing all but dihedral angles of the hydroxyl groups. Then, the geometry optimization was performed with Density Functional Theory (DFT) using the B97-D3 functional [83], which is a popular choice for organic molecules. Electron repulsion integrals were treated within the Density Fitting (DF) approximation [84] and the def2-TZVP orbital basis set [85] was used with the corresponding default auxiliary DF basis. The resulting geometries are similar to the starting ones (no hydroxy group has switched from *u* to *d* or *vice versa* during the optimization), so the final conformers will be denoted as *pc* (from pinched), *al* (from alternate), and *wc* (from winged-cone), as the original structures from the CSD. The stability of these conformers for the CX case was tested by a modification of their geometries through a rotation of selected phenyl rings and reoptimization, which either led to the original conformer, or to local minima energetically higher than *pc*, *al*, or *wc*.

### 2.2. Geometry Optimization–Complexes

The search for the optimal geometries for complexes of twenty amino acids (for formulas and abbreviations see the Supplementary Information) with *pc*, *wc*, and *al* conformers of either calix[6]arene, or hexa-*p*-*tert*-butylcalix[6]arene has been performed in several steps. Firstly, the generation of approximate geometries of the most plausible host–guest conformers was performed with the AutoDock Vina program [86]. Secondly, the resulting geometries were used as starting points for the DFT optimization. Because of the high computational cost, the def2-SVP basis set was used for the search of local minima, and solely the lowest ones were reoptimized using the def2-TZVP basis set. It should be noted that for the arginine (Arg) case, all AutoDock Vina optimizations lead to the protonated (charged) version of the amino acid. Since the interaction energies between two ions would give a completely different interaction picture than for uncharged molecules, we decided to skip this amino acid from our test set. For further analysis, only the lowest minima for each calixarene conformer and amino acid were utilized.

Gaussian16 [87] was used for the geometry optimizations. The harmonic frequency analysis has been performed in order to verify that the stationary point is the minimum.

### 2.3. Energetics

In this part, we focus on the relative stability of *pc*, *al*, and *wc* conformers for both pristine calixarenes and complexes. The stability order can be established by comparing total energies, which are sums of the DFT+D electronic energy calculated at minimum and the nuclear contribution, given by zero-point vibrational energy (ZPVE). In practice, since these total energies have large absolute values and are similar to each other, it is the most useful to find the lowest conformer and to report relative differences of energies for the two remaining conformers. It should be emphasized that the energetic order of complexes of different calixarene conformers with the same amino acid, which results from this procedure, does not always correspond to the order of the interaction energies since a difference in conformers’ energies contains additional deformation contributions for the interacting molecules. In particular, the calixarene’s deformation may vary substantially for different complexes because of the large flexibility of calixarenes.

The selected complexes in their optimal geometries have been studied with the help of several methods in order to perform a detailed analysis of their energetics with special attention paid to energy partitioning. A method of choice for such a study is symmetry-adapted perturbation theory (SAPT) [44,88], which allows us to obtain not only intermolecular interaction energies but also their components, such as electrostatic, induction, dispersion, and their exchange counterparts, with a well-defined physical interpretation,

(1) $$\begin{array}{c} E_{\mathrm{int}}=E_{\mathrm{elst}}^{\left(1\right)}+E_{\mathrm{exch}}^{\left(1\right)}+E_{\mathrm{ind}}^{\left(2\right)}+E_{\mathrm{exch}}^{\left(2\right)}-\mathrm{ind}+E_{\mathrm{disp}}^{\left(2\right)}+E_{\mathrm{exch}}^{\left(2\right)}-\mathrm{disp}+\delta E_{\mathrm{HF}}. \end{array}$$

In this study, we utilized the simplest SAPT variant, denoted as SAPT(HF) or alternatively as SAPT0 [89] SAPT0 is also a method of choice for further F-SAPT analysis.

Supermolecular interaction energies were obtained on the MP2 and Spin-Component-Scaled MP2 (SCS-MP2) [90] levels of theory with the jun-cc-pVDZ basis set [91,92,93]. Since the DF implementation of these methods was used, the corresponding default auxiliary basis sets [94,95,96] were applied in these calculations, which were performed with the Psi4 program [97]. In order to estimate the accuracy of the interaction energies with respect to the basis set, we performed the Complete-Basis-Set (CBS) extrapolation [98] for the correlation part of the interaction energy for one selected complex. For all supermolecular calculations, the Boys–Bernardi counterpoise correction was employed to avoid the basis-set superposition error [99].

Additionally, we studied the interaction energies with the help of a Symmetrized Systematic Molecular Fragmentation on the third level [49] with interaction energies between fragments calculated with the MP2 method (SSMF3-MP2) method. The SSMF arises from the SMF by Annihilation (SMFA) [100], and differs from the original one by treatment of branched molecules [49]. In the SMFA, one first defines molecular units, which are composed either of a single nonhydrogen atom, or atoms connected by multiple bonds, both with attached hydrogen atoms. Then, a molecule is fragmented into overlapping units, where broken single bonds are saturated with hydrogen atoms. Therefore, the molecule is represented as a sum of overlapping fragments with positive and negative weights, and the energy is calculated as a sum of the energies of overlapping fragments corrected with a sum of interaction energies of non-overlapping units [100,101,102], i.e., if the fragmentation of a molecule M into fragments $G_{i}$ with weights $w_{i}$ is performed, the total energy of M is expressed as

(2) $$\begin{array}{c} E\left(M\right)=\sum_{i}w_{i}E\left(G_{i}\right)+E_{\mathrm{nb}}, \end{array}$$

where E(X) is the energy of the molecule *X* and $E_{\mathrm{nb}}$ is the (usually small) interaction energy of the non-overlapping parts. The size of fragments is given in terms of the number of units, and as a rule, one obtains fragments of size *n* and n+1 for the *n*th level of fragmentation. In the SMFA approach, the fragments which incorporate branches are bigger and can be formed of n+2 or more units, which makes the calculation more expensive computationally. In our recent publication [49] we proposed an alternative treatment of branching points, which keeps an immediate symmetry around branches (therefore the name) and–more importantly–preserves the number of units at cost of introducing fractional weights. Numerical tests show that the third fragmentation level (i.e., SSMF3) has sufficient accuracy for practical applications. We also studied the applicability of the SSMF3 approach for intermolecular interaction energies and found that the computationally expensive long-range correlation energy contribution to the interaction energy is well reproduced on the SSMF3 level [50].

The SMF approach may be also utilized to obtain the interaction energies. If one molecule (M) is large enough to undergo fragmentation, while the second one (A) is small and will be left unfragmented, then the interaction energy between M and A can be obtained by utilizing a *method* to calculate the interaction energy for each pair G${}_{i}$ with A, and then by applying the formula,

(3) $$\begin{array}{c} E_{\mathrm{int}}^{\mathrm{method}}\left(\mathrm{MA}\right)=\sum_{i}w_{i}E_{\mathrm{int}}^{\mathrm{method}}\left(G_{i}A\right). \end{array}$$

This is exactly the case of complexes with calixarenes, where the amino acids are left unfragmented, while the calixarene host is fragmented at the SSMF3 level [50]. Altogether 24 such fragments are generated for the CX case: twelve 6-methyl-2,2${}^{\prime }$-methanediyl-di-phenol, six 2,2${}^{\prime }$-methanediyl-di-phenol, and six 2,6-dimethylphenol molecules, so 24 (short) calculations of the interaction energies should be performed instead of one long calculation. The timing gain may be significant, especially for high-level electron-correlated theories, because of the steep scaling of such methods with respect to the molecular size, and since the calculations of fragments can be performed in parallel.

The SSMF3 partitioning has been performed by a homemade C++ program written by one of us (E.M.).

### 2.4. Molecular Properties

For pristine calix[6]arene, in addition to the total energy calculations, we performed computations of selected first-order and second-order properties (electric dipole moments and static dipole polarizabilities) on the HF and MP2 levels. For these properties, we applied Equation (2) with energies replaced by the corresponding property and neglecting the nonbonding contribution. The HF and MP2 properties for SSMF3 fragments were obtained analytically with Molpro [103] (all electrons were correlated in the MP2 method because of the limitations of the MP2 polarizabilities program in Molpro [104]). Properties for unfragmented calixarenes were obtained with the finite-field (FF) approach of a field strength 0.003 a.u. and by comparing the 3- and 5-point formula to control the accuracy of the FF procedure (1s orbitals of nonhydrogen atoms were frozen in the MP2 method to speed up the calculations after verification that this approximation has a very small effect on these properties). Because of the testing character of this investigation, we used a small cc-pVDZ basis, for which the second-order properties for the whole molecules can be obtained in a manageable computational time.

### 2.5. Energy Partitioning

The main workhorse for the energy partitioning in this work is the Functional-group SAPT (F-SAPT) method [46,47] and its intramolecular variant I-SAPT [105], which have been utilized to determine the inter- and intramolecular interactions between interesting groups of atoms. For this purpose, the partitioning on the SAPT0 level in the jun-cc-pVDZ basis set [93] was utilized. In the F-SAPT, both SAPT partitioning of the interaction energy into physically-sound components and the partitioning of these components into contributions, which can be attributed to the interactions between groups of atoms, are utilized, which gives us the opportunity to classify inter-group interactions not only in terms of their strength (i.e., by comparing the absolute value of the interaction energy) but also according to their dominant SAPT contribution. In practice, one usually examines electrostatics, first-order exchange, and effective induction and dispersion, which are obtained by adding up second-order induction, exchange-induction, and the $\delta E_{\mathrm{HF}}$ term, or second-order dispersion and exchange-dispersion, respectively. In order to facilitate the analysis, we developed a graph system [106], where groups of the molecule A and B are placed on the left and right edges of the graph and are connected with red (attractive) or blue (repulsive) lines, whose thickness is proportional to the absolute value of the component under study. It should be emphasized that both F- and I-SAPT energy partitionings should be treated with caution since such partitionings depend to some extent on the applied fragmentation approach. In the (F/I)-SAPT case, this fragmentation is orbital-based and therefore is dependent on the balanced description of various atoms and bonds within a given basis. It also contains contributions from the so-called “linkers”, which appear at places where the bonds between groups are broken. The latter contributions are somewhat arbitrarily divided between adjacent groups in the reduced analysis; therefore, the analysis of tiny energy fluctuations for partitioned energies is of limited value. Numerous studies performed with either F-, or I-SAPT show [107,108,109,110,111] that many useful conclusions can be reached if one adheres to hints given in the original papers of Parrish et al.. Therefore, in this study, we apply a standard (for F-SAPT) jun-cc-pVDZ basis and the simplest SAPT0 rung of SAPT models, as advised in Ref. [46].

For purposes of the energy partitioning with F-SAPT, the selected functional groups for the case of calixarenes are: hydroxy (OH), phenyl (Ph), methylene (CH_2_), and *tert*-butyl groups (in order to make an easier comparison of the calix[6]arene and hexa-*p*-*tert*-butylcalix[6]arene we separated the *para* hydrogen atom in the phenyl ring as a separate group–this hydrogen is replaced by the *tert*-butyl group in the latter molecule). The common functional groups of amino acids are: amino (NH_2_) and carboxy (COOH) groups. Depending on the amino acid type, other functional groups are singled out (see F-SAPT graphs for all the complexes under study in the Supplementary Information). For instance, for the simplest Gly, the only remaining group is the methylene group CH_2_, while for the Phe amino acid, there are additionally the ring and CH groups. We have chosen not to fragment the carboxy group into C=O and OH; therefore, in some cases, the possible classification of the bonding involving this group as the H-bond (or not) will require a visual check.

**Figure 2:**
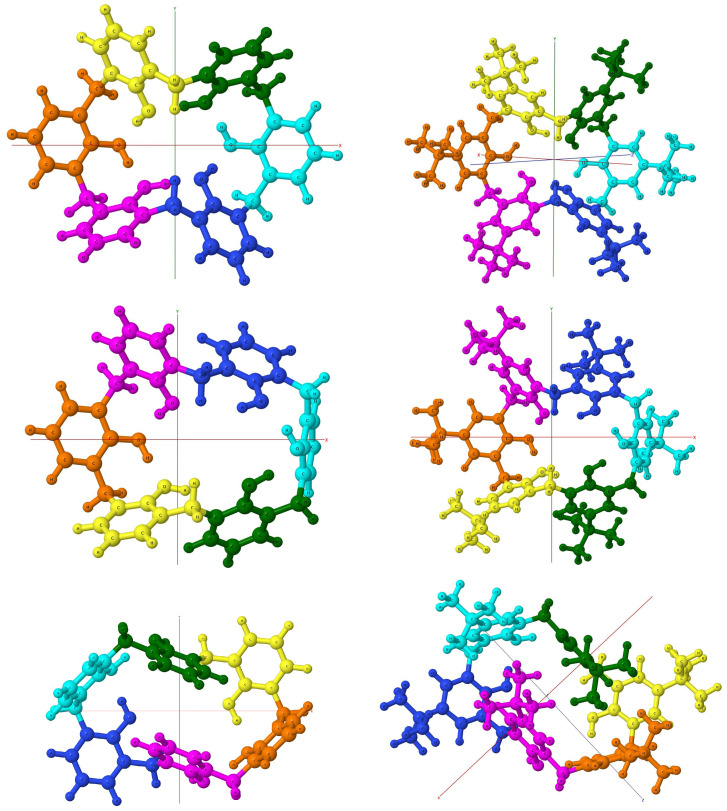
A numeration of calixarene groups for *pc*, *al*, and *wc* conformers (upper, middle, and lower rows, correspondingly). Colors correspond to the following numbers: orange–1, yellow–2, green–3, cyan –4, blue –5, violet– 6.

For sake of conciseness, we define a calixarene “unit” consisting of a phenolic group (i.e., either a phenol group or *tert*-butyl-substituted phenol group) and a methylene bridge, and we will number these units as in Figure 2. If necessary, the respective groups within these units will be distinguished by adding a number behind the group (i.e., OH-*k*, Ph-*k*, CH_2_-*k*, *tert*-butyl-*k* denote the hydroxy, phenyl, methylene, and *tert*-butyl groups of the *k*th calixarene unit, respectively). Analogously, we will differentiate the group X of an amino acid Y as X-Y, e.g., the COOH group of Gly will be denoted as COOH-Gly to avoid confusion.

For the case of the SMF, the molecular units are selected automatically and grouped into fragments by the fragmentation procedure; therefore, in several cases, two or more interesting groups are placed together in one fragment. Nevertheless, by a difference analysis, one can often determine the main “culprit” for a strong interaction within the fragments. These analyses of SSMF3 pair interaction energies between fragments of molecules A and B give us a complementary view of the interaction energy partitioning.

The F-SAPT and I-SAPT partitionings, SAPT0 and most MP2 and SCS-MP2 calculations have been performed with the Psi4 program [112]. Some supermolecular calculations were performed with Molpro [103].

## 3. Results and Discussion

### 3.1. Pristine Calixarenes–Geometries

The characteristic feature of calixarenes is the existence of multiple intramolecular H-bonds, formed between its hydroxy groups. The creation of these bonds can be promoted or prohibited depending on the relative positions of the aryl rings in various conformers. To this end, two trimeric parts (i.e., consisting of three calixarene units), vaguely resembling semi-circles, can be distinguished in the structure of the *pc* conformer of calix[6]arene. All six hydroxy groups are on the same side of the backbone and because of a favorable orientation of semi-circles with respect to each other, each of these groups is involved in hydrogen binding as the H donor and the H acceptor, thus creating a ring-like structure of six H-bonds. Each one from two methylene bridges connecting trimeric subunits has one hydrogen atom pointing inside the macroring with a distance of 3.22 Å (3.02 Å) between these hydrogen atoms for the CX (BCX). Therefore, the cavity of the *pc* conformer is divided into two parts by this dihydrogen bridge and the top-side entrance is blocked by the second ring formed by the H-bonds. The distances between the oxygen atom of one hydroxy group and the hydrogen atom of the neighboring hydroxy group (denoted in the following as the H-bond distance) indicate that indeed all H-bonds formed in this case are strong–they are in range between 1.66 to 1.70 Å and–somewhat surprisingly–for the hexa-*p*-*tert*-butylcalix[6]arene case not all of them become larger, as one could expect (two become larger, while the remaining four–a bit shorter). For the H-bond the orientation of three atoms O-H⋯O should be close to 180${}^{\circ }$ for optimal binding [51]. For a cyclic structure, as in the calixarene case, the H-bondings are always stressed in this aspect, and, e.g., for the *pc* conformer the H-bond angles are: 173, 165, 166${}^{\circ }$ for the CX and 174, 164, 166${}^{\circ }$ for BCX. Similar values of distances and angles in these both cases indicate that the *tert*-butyl substitution does not cause any significant distortion in calixarenes.

A similar semi-circle structure exists for the *al* conformer, but in this case, three adjacent hydroxy groups are placed upside down; therefore, only two H-bonds can be created on each side of the macrocycle. Moreover, in this case, one hydrogen atom from the methylene bridges connecting two trimeric semi-circles points to the interior of the molecule, but the H⋯H distance is significantly larger and amounts to 3.70 Å for the CX and 3.58 Å for the BCX. Judging from the H-bond distances between oxygen and hydrogen atoms of the respective hydroxy groups, the *al* H-bonds should be weaker than for the *pc* case: these distances range between 1.75 and 1.81 Å, i.e., even the shortest distance is about 0.05 Å longer than for the *pc* conformer. From the H-bond angle values, which span from 166 to 168${}^{\circ }$, one can make a conclusion that only those more “stressed” H-bondings survive in the *al* conformer as compared to the *pc* one.

Finally, the *wc* conformer can be visualized by the flattening of two opposite phenyl rings in the *pc* conformer. As a result of this operation, the distance between two pairs of hydroxy groups becomes too large for an effective H-binding and only two triples of adjacent OH groups are still able to create two H-bonds each, similarly to the *al* case. The O⋯H distances for the *wc* conformers range from 1.81 to 1.87 Å, i.e., are larger than in the *al* case, indicating that the formed H-bonds in the *wc* conformer are even weaker than in the *al* case. The addition of the *tert*-butyl groups does not have a significant impact on the lengths of the H-bonds, but it does affect the positioning of the phenol groups and the closest hydrogen atoms from the opposite methylene groups. Interestingly, in this case, the *tert*-butyl substitution results in an increase in the H-bond angles (168–170${}^{\circ }$ for the BCX and 166–168${}^{\circ }$ for CX). As can be expected, the distance between the closest carbon atoms of the opposite phenol rings (third and sixth) increases substantially for the BCX (from 3.82 to 5.28 Å). Additionally, some hydrogen atoms from different *tert*-butyl groups are exceptionally close to each other (2.28 Å).

For unsubstituted calix[6]arenes the *pc* and *wc* conformers have the C${}_{2}$ symmetry axis, while no symmetry elements exist for the *al* conformer. If the molecular skeleton, i.e., the calixarene ring without hydrogen atoms attached to the hydroxy groups and–in the case of *tert*-butyl-substituted calixarenes–the ring without *tert*-butyl groups, is examined. Then, additional similarity features can be detected, although no exact symmetry elements can be named. For instance, for the *al* and *pc* conformers the lower and upper parts of the molecules, as seen in Figure 2, are quite similar to each other. A detailed examination of dihedral angles shows that this is indeed the case: most corresponding angles differ by a few degrees only, with the exception of one-two angles, where these differences are larger.

### 3.2. Pristine calixarenes–IR Spectra

The simulated harmonic IR spectra of three forms of calix[6]arene and hexa-*p*-*tert*-butylcalix[6]arene, together with a complete list of frequencies and intensities are presented in the Supplementary Information. Studies of smaller molecules containing the hydroxy groups, such as, e.g., studies of phenol and its complexes with water and ammonia [113] or a study of water dimer IR spectrum [114] with respect to basis sets, anharmonicity and couplings, etc., show that harmonic stretching frequencies are blue-shifted by 100–150 cm${}^{-1}$ in these cases, so one expects a similar shift of the calculated O−H stretching frequencies for calixarenes.

From IR spectra presented in Figures in the Supplementary Information one can see that there is a group of high-intensity frequencies above 3000 cm${}^{-1}$ for all the cases. The *pc* conformer is characterized by just one high-intensity composite peak, while for the remaining conformers, this peak is shifted to higher frequencies of about 3500 cm${}^{-1}$ and one finds an additional lower peak (or two peaks for *al*) at even higher energies. For the BCX case, an additional bunch of peaks appears at about 3000 cm${}^{-1}$, which is well separated from the first group, and whose intensity is comparable to the previously discussed group for the *al* and *wc* conformers. Let us first focus on the highest vibrational frequencies, which turn out to be related to hydroxy group stretching. The six highest frequencies for the *wc* and *al* conformers correspond to various O-H stretching patterns, as can be expected for six OH groups. Since these frequencies span from about 3300 cm${}^{-1}$ up to about 3700 cm${}^{-1}$, and the C-H stretching modes start from about 3100 cm${}^{-1}$, the stretching modes for O-H and C-H do not mix. A different situation arises for the *pc* conformer. The highest frequency (3270 and 3280 cm${}^{-1}$ for CX and BCX, respectively) is much lower than for the *al* and *wc* conformers, and the examination of mode characters for consecutive frequencies reveals only the five highest ones are dominated by the O-H stretching. The “missing” sixth mode can be found at 3115 cm${}^{-1}$ for the BCX, separated from five OH-stretch frequencies mentioned above by several C-H stretching modes. The situation is even more complicated for the CX case, where instead of one missing mode dominated by the O-H stretch one finds three nearly degenerate modes of frequency 3099 cm${}^{-1}$. They can be described as a simultaneous symmetric stretch of all present O-H bonds mixed with various patterns of C-H stretches, where the C-H bonds come from the phenyl groups. The replacement of the hydrogen atom in the *para* position by the *tert*-butyl group leads to a distortion of this mixing, which results in the absence of such mode combinations in the BCX case.

All the five highest frequencies of *pc* correspond to concerted stretching motions, i.e., involving simultaneously all six O-H bonds. They differ by the pattern of the motions, and, e.g., for the highest energetic mode there is–quite understandably – the alternate motion (when odd O-H bonds stretch then even ones shrink). More precisely, one can differentiate several patterns of these motions and–as usual in such cases–more nodes in the wave function mean higher energy. To this end, if we start counting the O-H bonds as in Figure 2, then the pattern corresponding to the highest frequency is the alternating one: (+,−,+,−,+,−), then the next highest is partially alternate (+,− −,++,+,− −,++), followed by more and more symmetrical patterns, such as (++,−,−,++,−,−), (++,−,− −,− −,+,++), and (++,++,+,− −,− −,−), whereby doubled plus or minus signs try to catch the increased amplitude of the motion. Finally, as discussed above, the expected totally symmetric case, i.e., (+,+,+,+,+,+), is mixed with C−H stretching motions for the CX case.

The lowering of spatial symmetry, as in the case of the *al* conformer, leads to a decoupling of the O-H stretch modes. Their highest two frequencies correspond to the stretching of a single O-H bond (for the OH-5 and OH-2 groups, respectively). The same two OH groups are involved in the two highest modes for the *wc* case. In all these cases, the high-frequency vibration corresponds to the OH group, which is involved in the H-bond as an electron donor only (i.e., through the oxygen ending), while the hydrogen atom does not participate in the H-bond because of the geometry hindrance. As a result, the O-H bond is not weakened by the H-bond and no red shift is observed in the IR spectrum. For the *wc* case there are two such high-energy (about 3700 cm${}^{-1}$) vibrations, one corresponding to a simultaneous stretch and another–to an alternate stretch of the OH-2 and OH-5 groups. It should be noted that values of these frequencies are similar to water stretching frequencies [114], or to the stretching frequency of the hydroxy group in phenol [113]. However, for the *al* conformer (for both unsubstituted and substituted calixarenes) one of these high-energy vibrations becomes lower by approximately 100 cm${}^{-1}$. This fact can be explained by a weak attractive interaction of the hydroxy group with the phenyl ring. Such interactions were reported and studied for the prototypical models of benzene with one or two water molecules in Ref. [115]. We will go back to this topic in Section 3.7.

Returning to the *pc* conformer of the CX, one can detect several characteristic IR peaks at lower frequencies. An examination of these cases shows that some of them belong to the O-H bending (either in-plane or out-of-plane with respect to the phenyl ring), while others are C-H bending from phenyl or breathing Kekule modes of phenyl rings. Similar peaks appear for the *al* and *wc* conformers, but they differ in intensities and exact positions of peaks, allowing us, in principle, to recognize which conformer has been measured.

For the hexa-*p*-*tert*-butylcalix[6]arene case, additional strong features around 3000 cm${}^{-1}$ should be attributed to multiple C-H stretching modes of methyl groups, which together form a *tert*-butyl group. Usually, several C-H bonds are simultaneously involved in these vibrations. Stretching frequencies of methylene bridges are of a similar energy range, so they contribute to these composite peaks as well.

### 3.3. Complexes with Amino Acids–Geometries

#### 3.3.1. Dihedral Angles of Calixarenes

Before we analyze the binding (docking) sites for amino acids, let us first systematically analyze modifications of the calixarene macrocycle upon complexation with help of dihedral angles between selected carbon atoms between calixarene units. The values of the dihedral angles are defined through carbon atoms as indicated in Figure 1 together with the standard nomenclature for the considered calixarene conformers are listed in the Supplementary Information for both CX and BCX types. One should note that for each linking methylene group two angles are defined with the convention that the dihedral angle denoted as R*n*aR*m* has two carbon atoms from the R*n* ring, while R*n*bR*m* has two carbon atoms from the R*m* ring in its definition. The numbering for dihedral angles corresponds to the numeration presented in Figure 2.

Let us first analyze the pristine calixarenes. At the beginning, one should note that *pc*, *al*, and *wc* conformers can be distinguished by distinct sign patterns of the dihedral angles, which are: (±∓±±∓±) for *pc*, (∓=∓±=±) for *wc*, and (∓∓±±∓∓) for *al* (the ± symbol here denotes that first from two dihedral angles around a given methylene group is positive and the second negative, ∓–the opposite, while = denotes that both angles are negative). Secondly, because of the $C_{2}$ point-group symmetry within the *pc* and *wc* conformers for the CX, the second half of the dihedral angles is identical to the first half. No such exact symmetry exists in the *al* conformed; however, as noted above, there is an approximate correspondence between two parts of the *al* conformer and in an ideal case the *k*th angle from the table would correspond to the negative of the (12−k)th angle. The same resemblances can be also found for the *pc* with the majority of differences of the order of a few degrees for the CX. For the hexa-*p*-*tert*-butylcalix[6]arene conformers, no exact point symmetry exists because of the presence of *tert*-butyl groups, but the same approximate resemblance is nevertheless preserved, confirming a small influence of the substitution on the macroring shape. It should be emphasized that no case has been found during geometry optimization where the complexation would modify the geometry of the calixarene to such an extent that the conformer became unrecognizable, although in a couple of cases the sign pattern is not preserved anymore.

In order to make the analysis of numerical values of the dihedral angles more complete, we compare them to two model molecules, which are also built from two phenol groups connected through the methylene linker. The geometry optimization of these molecules has been performed on the same level as for calixarenes. The simplest molecule of this type is diphenylmethane, for which the corresponding dihedral angles are equal to $-62^{\circ }$ and 118${}^{\circ }$. It turns out that there are indeed pairs (R*n*aR*m*,R*n*bR*m*), which resemble this pattern, but only for the *pc* conformer such a correspondence holds for all six pairs, while for the *al* and *wc* cases at least two angles are much smaller (by about 10–20${}^{\circ }$), so here other factors, such as hydroxy groups’ interaction, should play a decisive role. In order to examine this issue in more detail, we likewise performed a geometry optimization for several conformers for the 2,2${}^{\prime }$-dihydroxydiphenylmethane molecule, which is the simplest molecule with two hydroxyphenyl groups connected with the methylene linker. Only one of these conformers has an intramolecular H-bond and the creation of this bond is gratified by the highest stability. The corresponding pair of dihedral angles for this H-bonded conformer is ($-76^{\circ }$,$101^{\circ }$), which corresponds quite accurately to all the pairs of the *pc* conformer and to four from six pairs of the *al* and *wc* conformers. The 2,2${}^{\prime }$-dihydroxydiphenylmethane conformer, which resembles the most the dihedral angles for the fifth and sixth ring of *al* and *wc* (and, because of symmetry, also for the second and third ring of *wc*) has two hydroxy groups separated by the CH_2_ group, but they still point towards each other as an attempt to create an H-bond. It can be seen that although the distance between the corresponding oxygen and hydrogen is too large for effective creation of the H-bond (4.4 Å), it is still 0.3 Å smaller than for the *wc* conformer, i.e., one can say that these hydroxy groups are placed less optimally in the *wc* calixarene. One can also see this by a comparison of dihedral angles, which differ by as much as 22${}^{\circ }$ when comparing *wc* to 2,2${}^{\prime }$-dihydroxydiphenylmethane. For the *al* case no such strains are observed. In order to fully describe the *al* conformer, one more local minimum of 2,2${}^{\prime }$-dihydroxydiphenylmethane should be used, which has the corresponding pair of angles equal to ($-19^{\circ }$,117${}^{\circ }$).

Interestingly, the replacement of hydroxy groups by hydrogens and reoptimization of the resulting hydrocarbons leads to a complete distortion of semi-circles in the *al* and *pc* cases, while the *wc* conformer does not change so dramatically. Therefore, for the former two cases, the intramolecular H-bonds play a pivotal role in the stability of the molecule. Differences between dihedral angles and the corresponding angles for 2,2${}^{\prime }$-dihydroxydiphenylmethane can serve as an indicator of the intramolecular strain. One can see that for pristine calixarenes these differences are small for most angles, which supports the conclusion about the general stability of these conformers. Only for a few angles are these differences larger than 10${}^{\circ }$, such as, e.g., the angles between third and fourth (and symmetrically: first and sixth) rings for the *pc* case, first and second for the *al* and R2bR3 (and symmetrically: R5bR6) for the *wc* conformers of calix[6]arene. The substitution of hydrogens with the *tert*-butyl substituent leads to the largest strains for the *pc*-BCX case (two-digit differences for eight out of twelve angles, with the largest change of 28${}^{\circ }$). For *wc*-BCX conformer, the same R2xR3 (*x* means in the following both *a* and *b*) angles show differences of 10${}^{\circ }$ and $-13^{\circ }$, and the smallest differences with respect to the 2,2${}^{\prime }$-dihydroxydiphenylmethane conformers are found for the *al*-BCX conformer ($-11^{\circ }$ for R1bR2). The largest distortion for the *pc* case can be easily explained by the fact that in this case, all *tert*-butyl groups reside on the same bottom side of the calixarene macroring, so an adaptation of this ring should be performed to avoid repulsion between the *tert*-butyl groups.

The analysis of geometrical changes in calixarenes caused by the complexation can be performed at best by making a comparison between these angles for the pristine calixarenes and angles for calixarenes within complexes. On average, these changes are again quite small and become significantly larger only for a couple of angles (most often these are the angles R2bR3 and R5bR6 for *pc*, R2aR3 and R4bR5 for *al*, and R2bR3 and R5bR6 for *wc*). The presence of *tert*-butyl groups makes the calixarene backbone more rigid for the *wc* and *pc* conformers, since the average change for the BCX is smaller than for the CX. This rigidness can be explained by the fact that the *tert*-butyl groups cannot be easily moved in space because they start to overlap, and this problem is especially severe when all these groups reside on the same side of calix[6]arene (i.e., for the *pc* and to some extent for the *wc* cases). For several amino acids, the distortion of one or more dihedral angles is quite significant, which indicates a larger geometry modification of the calixarene and possibly larger deformation energy. Especially for complexes with the *wc* conformer of CX for the majority of cases, one angle is modified by more than 30${}^{\circ }$. The only amino acids for which there is no such large distortion are: AspH, Cys, Gln, GluH, Lys, and Pro. For the *al* conformer, such a large distortion occurs for two cases only (HisE and Pro), while other large distortions are at most 20–22${}^{\circ }$ (for Cys, Gln, Gly, Ile, and Thr). For the *pc* conformer changes are even smaller: only for two cases the largest discrepancy amounts to 20${}^{\circ }$ (for Asn and GluH) and is smaller for the remaining cases. A comparison to the BCX complexes leads to a conclusion that the largest differences for the *wc* conformers are much smaller than for the unsubstituted counterpart–only for two cases the difference is larger than 20${}^{\circ }$ (HisE and Trp). For the *al*-BCX conformer, there are two cases of distortion larger or close to 30${}^{\circ }$ (Tyr and Phe). Finally, for the *pc* case there is one case of a change close to 20${}^{\circ }$ (for HisE), while the other largest discrepancies are much smaller (about 15${}^{\circ }$ for several cases, but usually–a one-digit number). Therefore, the overall conclusion is that the substitution with *tert*-butyl groups hinders geometry modifications under complexation in the majority of complexes.

#### 3.3.2. Binding Sites

An analysis of complexes of calix[6]arenes with amino acids reveals that several preferred binding sites can be identified. The most straightforward situation arises for the *wc* conformer, where for all forty cases (twenty amino acids and two calixarenes), the preferred binding site is on the top of the molecule, i.e., where all the hydroxy groups reside. For this conformer the hole is smaller because of the flattened shape–one should note that two phenyl rings (third and sixth according to Figure 2) are placed approximately in a position of a “shifted sandwich”, which turns out to be the optimal one for two benzene molecules [116].

For the *al* conformer, two preferred binding sites can be identified, which both have a form of a cavity because of a specific orientation of calixarene units for this conformer: in comparison to the *pc* case three units are turned upside down. The most popular cavity is the cavity created by the third, fourth, and fifth units in the case of CX, but in some cases, the second cavity created by the remaining rings is used by amino acid guests. The latter situation occurs for: Phe-CX, Phe-BCX, Trp-CX, and Tyr-BCX. It should be noted that for all these cases, the amino acid contains an aryl group.

For the *pc* conformer, three docking places can be identified. One of these places is the top of the cone, while the other–the bottom cavity. If a larger amino acid occupies the cone, its residual part is usually placed along the groove made of units two and three, i.e., between two semi-circles, and in some cases, the groove becomes the only docking site (see the complex with Tyr) without attaching to the hydroxy-rich cone part at all. In the latter case, the guest molecule is, as a rule, shifted from the center of the cavity and resides on one side of the semi-circle, but in several cases (for larger molecules) both cavities are used. Somewhat surprisingly, the top position is preferred for the calix[6]arene host, while inclusion complexes are more common for the hexa-*p*-*tert*-butylcalix[6]arene, in spite of space obstacles on the calixarene rim for the latter case. Only Ile, Lys, Pro, and Val amino acids are docked in the cavity for the *pc*-CX. In all these cases one side of the cavity, created by units third to fifth, is used. Because of this position of the guest molecule, the structure of the six-member H-bond ring is often unharmed by the complexation, which can be also seen by the O⋯H distance in the H-bonds, which remains within the range 1.63–1.7 Å after the complexation. The distance between the closest hydrogen atoms from the methylene linkers (CH_2_-5 and CH_2_-2) become larger by 0.2–0.3 Å for Lys and Val, which are the largest amino acids from this set, while it is enlarged by only 0.1 Å for Ile and becomes 0.1 Å smaller for the most compact Pro guest. For the hexa-*p*-*tert*-butylcalix[6]arene host, a majority of amino acids choose the cavity as the preferred binding site and seven only are attached to the hydroxy-decorated top (Asn, Cys, Gly, HisD, HisE, Ser, and Thr).

The Cartesian geometries of all the complexes are listed in the Supplementary Information.

#### 3.3.3. Example: Complexes with Ala

**Figure 3:**
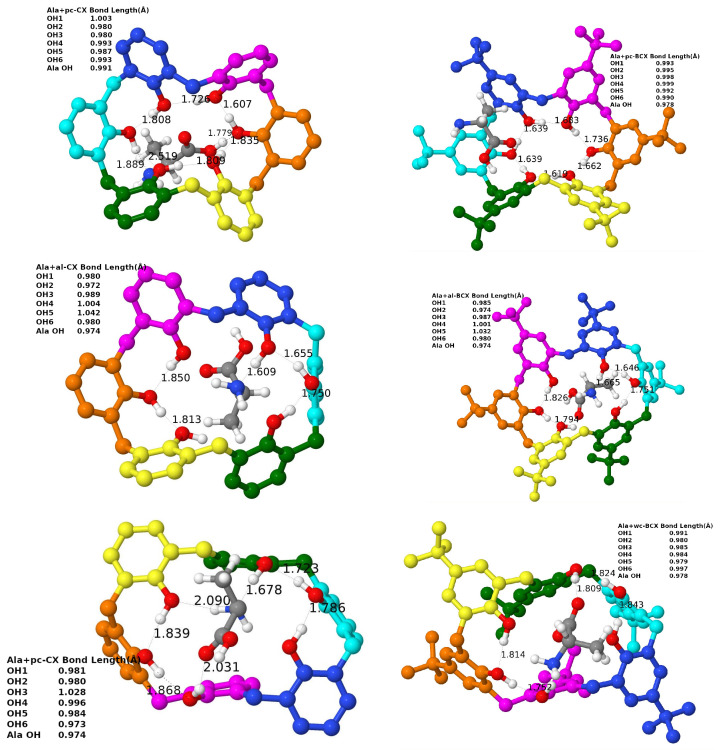
Selected bond lengths for O-H and H-bonds for the case of complexes with Ala. Hydrogen atoms of calixarenes are removed for a better view.

Let us focus on changes in hydroxy-group bond lengths in selected complexes. An exemplary case is shown for the complexes with Ala in Figure 3. For the *wc* and *al* cases, there is one O-H bond longer than 1 Å (for both *al*-CX and *al*-BCX, and for *wc*-CX) and in both cases, this O-H bond seems to be weakened by the formation of the H-bond with the NH_2_ group of amino acids. For the *al*-BCX there is also a second O-H bond longer than 1 Å, and again one can see that this elongation results from the involvement of the network of the H-bonds (this time with the second OH group). The O⋯H distance, in this case, is 0.1 Å smaller than usual. No O-H bond is significantly elongated in the *wc*-BCX, although the OH-3 group does interact with the amino group of Ala. One can see, however, that the H⋯N bond is longer than usual for this intermolecular H-bond. Interestingly, there is another intermolecular H-bond in this case which involves the hydroxy group of Ala and the O-6 atom of calixarene. For the *pc*-CX one has an H-bond with the amino group from Ala, too, but in this case, the H⋯N binding is by about 0.2 Å longer than for the *al* or *wc* cases. One should keep in mind that for the pristine *pc* conformer there is a network of six H-bonds that virtually close up the top part of the calixarene, but the Ala amino acid is able to break this network for the CX case. Interestingly, on the other side of the calixarene cavity, there is one H-bond which becomes shorter by about 0.1 Å in comparison to the uncomplexed case (to 1.6 Å), and the involved O-H bond becomes somewhat elongated (to 1.0 Å). Finally, for the case of the *pc*-BCX the network of the H-bonds remains unharmed by the interaction with Ala. In this case, the amino group is not directly involved in the interaction with calixarene.

When considering all the cases, it turns out that for all the *wc*-CX complexes there is one hydroxy group that becomes significantly elongated after the complexation (e.g., to 1.02 Å for Ala or 1.04 Å for Asn). This length modification should be, in principle, detectable in the IR spectrum. The elongated O-H bond points to the nitrogen atom from the amino acid group, therefore it creates an intermolecular H-bond with the hydroxy group donating its hydrogen. The N⋯H distance for this H-bond is quite small (1.6 Å) as for the intermolecular interaction, indicating its strong character.

### 3.4. Stability of Complexes with Amino Acids

In this study, we are mostly interested in energy partitioning and relative energies; therefore, we do not aim at achieving very accurate interaction energies. Nevertheless, we selected one case (a complex of *pc*-CX with Gly), for which we calculated the interaction energy in two basis sets from a modified Dunning series for the SAPT0, MP2, and SCS-MP2 theories. One of these basis sets, jun-cc-pVDZ, has been used for all other complexes, while the larger one–jun-cc-pVTZ–has been used for the purpose of the CBS study. As expected, the main part of the basis set unsaturation comes from the electron-correlation part of the interaction energy. A difference in the HF interaction energy between both basis sets amounts to 0.7 mH only, while the net dispersion from SAPT0 ($E_{\mathrm{disp}}^{\left(20\right)}+E_{\mathrm{exch}-\mathrm{disp}}^{\left(20\right)}$) is equal to 4.8 mH, a quantity similar to the correlation part of the MP2 and SCS-MP2 interaction energies, where these differences are equal to 5.0 and 4.1 mH, respectively. If a popular inverse cubic extrapolation formula [98],

(4) $$\begin{array}{c} E_{L}=E_{\infty }+\frac{A}{L^{3}}, \end{array}$$

is applied to the correlation part of the interaction energy (with L equal to 2 and 3 for jun-cc-pVDZ and jun-cc-pVTZ, respectively), then the estimated correlation energy decreases by another 1.7 mH for the SCS-MP2 case, so (taking the HF interaction energy on a larger basis) the estimate of the CBS limit of the SCS-MP2 interaction energy for this complex is −21.5 mH, which should be compared with −19.1 mH and −15.6 mH for the jun-cc-pVTZ and jun-cc-pVDZ basis sets, respectively. Therefore, the energetic results in the jun-cc-pVDZ basis set are rather semiquantitative and as a rule of thumb it should be rescaled by approximately $\frac{4}{3}$ to make the estimate for the CBS interaction energy.

**Figure 4:**
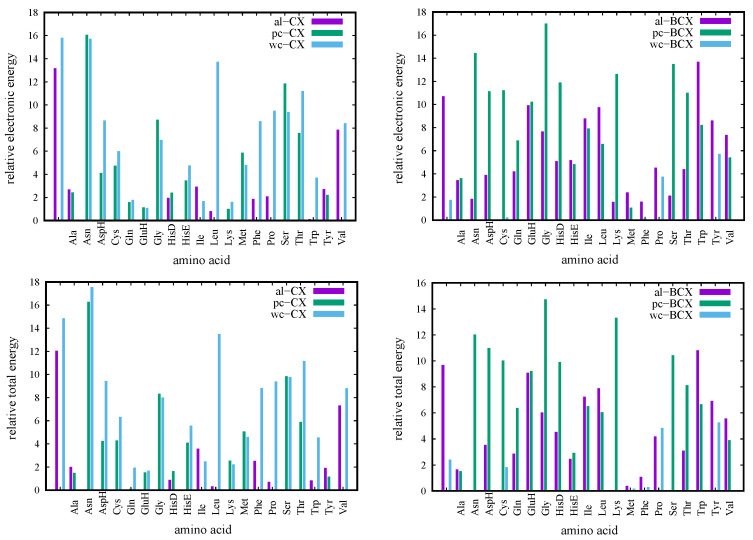
Relative total and electronic energies for complexes with three conformers of calix[6]arene and hexa-*p*-*tert*-butylcalix[6]arene. Energies are in millihartree.

The relative stability of both empty calixarenes for three considered conformations and complexes of these conformations with amino acids calculated from their total energies with respect to the lowest conformation are presented in Table 1 and in Figure 4. For empty calixarenes, the *pc* conformer is the most stable for both the CX and BCX cases, which can be explained by two more H-bonds stabilizing the macrocycle structure in comparison to the *wc* and *al* cases. For the *al* and *wc* conformers, the folding of the calixarene macrocycle prevents the creation of H-bonds between the following hydroxy pairs: first with sixth and fourth with third. The order of the *wc* and *al* conformers differs for the CX and BCX cases–in the former case, the *al* conformer has the lower energy, while the opposite is true for the BCX case. It should also be noted that the *wc*-BCX has the energy higher only by 2.4 mH than the *pc*-BCX, while both *al*-CX and *wc*-CX lie over 12 mH higher than the *pc*-CX. Therefore, at standard conditions, one can expect a sizable (about 7%) contribution of the *wc* conformer for the hexa-*p*-*tert*-butylcalix[6]arene, while for the case of calix[6]arene there is only one dominant conformer (*pc*).

The energetic order of complexes of *pc*, *al*, and *wc* calixarenes with amino acids is in most cases different than the pristine series. The energetic sequence is preserved for amino acids: Leu, Phe, Pro, Trp, and Val interacting with CX and only Met, Phe, and Tyr interacting with BCX. If the energetic difference between the highest and the lowest conformers of empty calixarenes with the analogous energetic span of complexes are compared, one can find that this difference becomes smaller (within 2.0 mH or less) for CX interacting with Ala, Gln, GluH, HisD, and Tyr, and for BCX interacting with Ala, Met, and Phe. For these complexes, all types of conformers are accessible under standard conditions. On the other hand, the energy span between conformers becomes larger than for pristine conformers only for one case (Asn) for the CX case and for Asn, AspH, Cys, Gly, HistD, Lys, Ser, and Trp for the BCX one. However, it should be noted that the energy span between conformers of the empty BCX (10.7 mH) is smaller than for the CX (15.8 mH). In general, the addition of large *tert*-butyl groups seems to reduce energetic differences in both pristine calix[6]arenes and their complexes. In several cases, the complexation diminishes the energetic differences for two from three conformers. If access to a specific conformer is desired, the special cases, for which one conformer has much lower energy than the two remaining ones are the most promising in view of potential applications. For complexes with CX especially, often the *al*-CX complexes become energetically more favorable than the *pc* ones. The exceptionally large difference between the *al*-CX complex and *pc* or *wc* ones occurs for the Asn amino acid (16 mH). Other cases include: Gly (8 mH), Ser (10 mH), and Thr (6 mH). The *wc*-CX complexes have the lowest energy for three cases only, but in all of these cases, the differences are very small (2 mH and smaller). The *pc*-CX remains the lowest one for a couple of cases, but only for the complex with Val energetic differences larger than 2.5 mH.

Contrary to the complexes with CX, it is the *wc*-BCX conformer that becomes the lowest energetically for the majority of complexes with amino acids. The situation where the other two conformers lie higher than 4 mH occurs for AspH, Gln, GluH, Gly, HisD, HisE, Ile, Leu, Thr, Trp, and Val. In several cases, two conformers (*wc* and *al*) are almost isoenergetic and lower than *pc*, such as in the case of Asn, Lys, and Ser. Finally, for the Phe amino acid, the differences between all three conformers are less than 2 mH. It should be noted that the *al*-BCX conformer does not become the preferred one for all but one case, which is the complex with Cys. However, even in this case, the next lowest conformer is the *wc* conformer.

Summarizing, the complexation with amino acids tends to modify the energetic order of both CX and BCX conformers, and in several cases, these differences are quite substantial. The extreme differences are seen more often for the unsubstituted calix[6]arene case. One should mention that one important potential application resulting from such a change of energetic order of *pc*, *al*, or *wc* conformers is a possibility of accessing, e.g., the *al*-type conformer for the purpose of chemical reactions with calix[6]arene, which would otherwise be dominated by the *pc* conformer.

### 3.5. Complexes with Amino Acids–Interaction Energies

**Figure 5:**
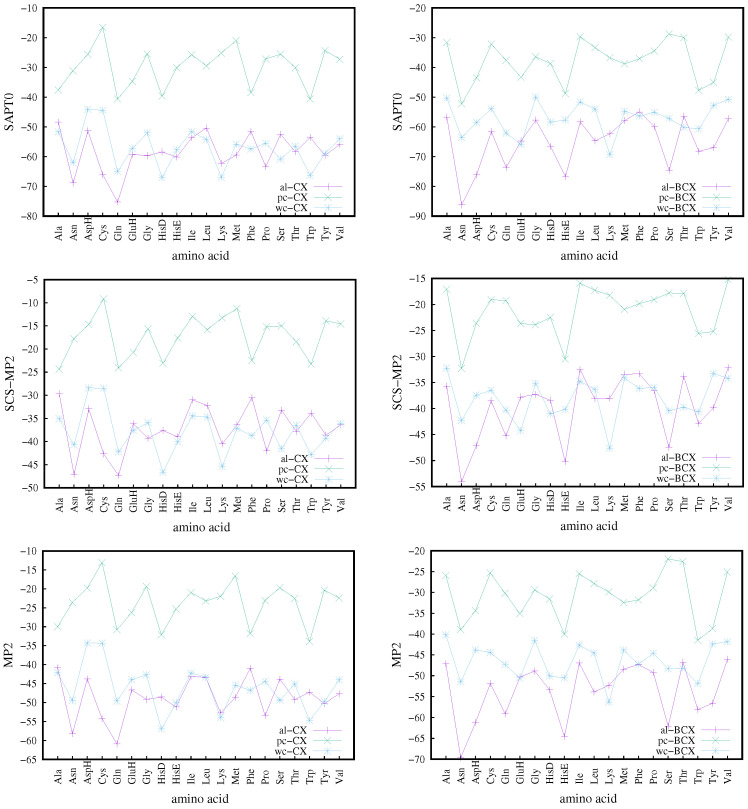
Trends in interaction energies for complexes with three conformers of calix[6]arene and hexa-*p*-*tert*-butylcalix[6]arene. Energies are in millihartree.

Interaction energies of the calixarene complexes with amino acids are presented for SAPT0, MP2, and SCS-MP2 methods in Figure 5. We treat the SCS-MP2 results as the most accurate since the scaling of the MP2 same- and opposite-spin energies, which make up the SCS-MP2 model, is devised [117,118] to reproduce the CCSD(T) correlation energy. The MP2 and SAPT0 results are consistently lower than SCS-MP2, but the interaction energies for all three methods follow the same global trend, as can be seen in Figure 5, where for a given conformer, the lines connecting interaction energies for various amino acids obtained with the same method look similar for SAPT0, MP2, and SCS-MP2. In particular, the *pc* conformer is the least prone to making a strong binding with amino acids, while the remaining conformers provide more attractive contributions (with a slight advantage of *al*). Since the F-SAPT and I-SAPT partitioning schemes utilize the preceding SAPT0 calculations, the consistency of SAPT0 and SCS-MP2 results should be especially emphasized in view of the reliability of further F/I-SAPT analyses. It is also interesting to note that on average the BCX complexes have lower interaction energies than the CX ones. A general analysis of the SAPT contributions shows that indeed the mean SAPT0 interaction energy for the BCX complexes is about 6 mH lower than this for the CX complexes, and the main contribution to this difference is due to the effective dispersion (10 mH lower), while the more repulsive first-order exchange contribution partially counterweights the additional attractive effect coming from electron correlation.

The analysis of Figure 5 leads to the conclusion that the largest attraction appears for the Asn and Gln amino acids for the *al*-CX conformer and for Asn for the *al*-BCX, and for Ala and (again) Asn for the *pc*-CX and *pc*-BCX, respectively. On the other hand, in the case of the *wc* conformer, it is the HisD amino acid, which shows the most attractive interaction for the CX, while the Lys molecule exhibits the largest attraction for the BCX.

The comparison of the SSMF3 interaction energies with the standard (unfragmented) ones, which is presented in Table 2 for the calix[6]arene (the analogous table for hexa-*p*-*tert*-butylcalix[6]arene has been moved to the Supplementary Information) shows that the SSMF3 approach (see Equation (3)) quite accurately reproduces interaction energies for investigated complexes. The first study of the applicability of SSMF3 for the interaction energies [50] revealed that the electron-correlation contribution is well reproduced by the SSMF3, while the accuracy of the HF part depends on the distance between the interacting molecules. The present study supports these conclusions, i.e., the electron-correlation parts of the SCS-MP2 interaction energies for complexes with the CX are reproduced with a mean percent error of 1.7%, with the largest absolute error of 3.7%. The electrostatic and first-order exchange SAPT0 contributions are obtained with good accuracy (the mean percent errors of 0.6% and 0.2%, and maximum ones 2.7% and 0.8%), while the most problematic component is the second-order induction (the mean percent error of 3.2%, but the maximum error is as large as 18%). One should note, however, that large percent errors for induction appear for those cases, for which this component is small with respect to other components (the largest error appears for the *pc*-CX with Met, where the second-order induction and dispersion contribute with −8.6 mH and −26.9 mH, respectively). The second-order dispersion, i.e., the pure electron-correlation effect, is reproduced quite accurately for all the cases (the mean and maximum errors of 1.2% and 2.0%, respectively). For the hexa-*p*-*tert*-butylcalix[6]arene complexes, similar error ranges occur, e.g., the maximum absolute errors for the electrostatic and first-order exchange are equal to 2.3% and 0.9%, and again the highest error arises for the second-order induction, but anyway, the maximum error is more than twice smaller (7.9%). The second-order SAPT0 dispersion is reproduced with a stable mean error of about 2% and the maximum error of 2.8%. The accuracy of the electron-correlated contributions from the MP2 and SCS-MP2 theories are of the same range as for the dispersion. Therefore, the comparison of the SSMF3-SAPT0 and SAPT0 results, as well as SSMF3-(SCS-MP2) and SCS-MP2 allows us to make a conclusion that in the region of a minimum of the intermolecular potential energy hypersurface the SMF-based methods give quite accurate results. Note that in the case of large percentage errors for the HF interaction energy, the energy itself is close to zero. This conclusion is important in view of our perspective applications of SSMF3 in the analysis of energy partitioning among functional groups.

### 3.6. Complexes with Amino Acids–IR Spectra

The simulated IR spectra of complexes of both calixarenes with selected amino acids can be found in the Supplementary Information.

The examination of vibrational modes of complexes of selected amino acids with calixarenes reveals many interesting features, with a general conclusion that the IR spectra in many cases can serve as a fingerprint of a complex. Especially useful in this aspect is the high-energy part of the spectrum, where the X-H stretch modes (either within one isolated bond or combined from several simultaneous motions within such bonds) undergo various modifications depending on their involvement in noncovalent interactions. In the following, as an exemplary case, we present only a short discussion of the IR spectra for the *wc* conformer.

A characteristic feature of all complexes with the *wc* conformer is a strong line at about 2500–2700 cm${}^{-1}$, which corresponds to the O-H stretching motion for the hydroxy group involved in a strong H-bond with the nitrogen atom from the amino acid group. One can see that the elongation of this bond is well correlated with the red shift of the frequency, as can be expected.

When one compares the region of 3000 cm${}^{-1}$ and higher, significant differences in intensities and/or emergence or line shifting can be noticed, which represent a combined result of additional motions of the N-H stretching and–in some cases–O-H stretching from the amino acid side. In particular, there are instances where the N-H stretch becomes the highest energetic line, such as, e.g., in *wc*-CX with Asn, *wc*-CX with Leu, or *wc*-CX with Lys.

The stretch within the COOH group appears for the *wc* case as a well-separated line at about 1700 cm${}^{-1}$. In some cases, there is a second line in this region, such as for the Asn guest, which corresponds to the stretch of the CONH_2_ group. In both cases, the main stretching involves the double C-O bond, but this motion is concerted with the neighboring OH or NH_2_ groups.

### 3.7. Pristine Calixarenes–Energy Partitioning

The calix[6]arenes contain six hydroxy groups, which can be oriented in such a way that one hydroxy group serves as a hydrogen donor, and the second one–as a hydrogen acceptor. Additionally, some hydroxy groups play both roles simultaneously. As already noted, for the *pc* conformer all six groups are connected in a way that allows the creation of six H-bonds, while for the *al* and *wc* cases four H-bonds are present. According to Figure 2 in the *al* conformer, the OH-6 group is connected (by its hydrogen ending) to OH-1, which in turn interacts with OH-2 on the left semi-circle of calixarene, and on the right semi-circle, the OH-3 group donates its hydrogen to OH-4, which in turn passes its hydrogen to OH-5. Similarly, for the *wc* conformer two chains of H-bonds are: OH-5 to OH-4 to OH-3 and OH-2 to OH-1 to OH-6. In Table 3, we list interaction energies between hydroxy groups of all six cases of calixarenes obtained from the I-SAPT method. (Note that I-SAPT calculations for the interaction between hydroxy groups from calixarene have also been performed for selected complexes, see the Supplementary Information).

The results in Table 3 indicate that the intramolecular OH-OH interactions strictly correspond to the H-bond pattern, i.e., for the *pc* conformer there are six and for the remaining conformers–four interaction energies, which are negative and below 9 mH. A more detailed analysis shows that the largest absolute value of these energies appears for the *pc* case (15 mH), followed by *al* (12 mH), while the energies for the *wc* conformer (9 mH) have the smallest absolute value. This order strictly corresponds to the increasing O⋯H distance. It should be noted that the absolute values of interaction energies between the second neighbors (such as OH-3 and OH-5) are larger than for the opposite groups, in full agreement with chemical intuition. An analysis of SAPT components (not shown) reveals that the second-neighbor interaction has a practically pure electrostatic character, while the interaction of the adjacent hydroxy groups contains similarly important contributions from electrostatics, induction, dispersion, and the exchange counterparts. Although a common explanation of the highest stability of the *pc* conformer is the existence of two additional intramolecular H-bonds, a simple addition of the interaction energies, mentioned above, predicts much higher stability than found from the total energies’ differences, see Section 3.4. In a fact, much more factors should be accounted for, among which secondary interactions, such as those involving phenyl groups, etc. should play a significant role.

Hydroxy groups in calixarenes reside in a tight neighborhood of other groups, from which the largest phenyl groups are of the highest importance. Since neighboring hydroxy and phenyl groups are placed in different relative orientations, interactions of various types can be obtained. We performed a detailed analysis of I-SAPT components of the hydroxy–phenyl interactions for the *pc* and *al* conformers, and found that the majority of these pairs interact electrostatically. Additionally, the closeness of the H or O ending of the hydroxy group allows us to predict the sign of the electrostatic interaction. For instance, OH groups with the H-ending placed closer to a neighboring Ph group form as a rule the repelling pair (e.g., OH-3 and Ph-4 with the energy of 9 mH, OH-4 and Ph-5 with the energy of 8 mH, OH-5 and Ph-6 with the energy of 2.5 mH, OH-6 and Ph-1 with the energy of 9 mH, and OH-1 and Ph-2 with the energy of 8 mH, for the *al*-CX). If the O-ending is closer to a neighboring Ph group, an attraction pair is formed (e.g., OH-4 and Ph-3 with the energy of −7 mH, OH-5 and Ph-4 with the energy of −8 mH, and OH-2 and Ph-1 with the energy of −7.5 mH for the *al*-CX). The same picture has been found for the *pc*-CX case, where the electrostatic attraction between pairs: OH-6 and Ph-1 with the energy of −9 mH, OH-1 and Ph-2 with the energy of −6 mH, OH-5 and Ph-6 with the energy of −8 mH can be found, while the electrostatic repulsion of the pairs: OH-6 and Ph-5 with the energy of 11 mH, OH-5 and Ph-4 with the energy of 11 mH, OH-1 and Ph-6 with the energy of 10 mH (plus $C_{2}$ point-group counterparts) reduce the stability of these conformers.

This rule of thumb does not work for neighboring pairs from the first and second semi-circle for the *al* conformers (both CX and BCX), i.e., for OH-6 and Ph-5 (+4 mH for CX and BCX) and OH-2 and Ph-3 (−4.5 mH for CX and −5 mH for BCX). The latter pair is different from all other neighboring hydroxy–phenyl pairs, since in this case the I-SAPT partitioning shows that all energy components are of equal importance and the resulting attraction is a result of a subtle balance of attractive and repulsive components of similar absolute values. Relatively large exchange components signify that electron clouds of both groups significantly overlap, which allows us to identify a weak secondary H-bond of the π-type [119,120]. It is especially worth noting that this noncovalent bonding facilitates the elongation of the OH-2 bond towards Ph-3 during the oscillation. We have already noticed in the study of the IR spectra that the frequency corresponding to the OH-2 stretch for the *al* conformers is lower than the OH-5 counterpart on the opposite site of the calixarene, so the behavior of the I-SAPT interaction energies clarifies the mechanism of this red shift.

### 3.8. Pristine Calixarenes–Molecular Properties

Since in a further discussion the F-SAPT and SSMF3 methods will be employed for the analysis of intermolecular interactions, it is interesting to explore how the latter method reproduces first- and second-order molecular properties, such as electric dipole moments and dipole polarizabilities. As discussed in the previous section, the HF and MP2 methods were selected for this test. For the CX case, the HF (MP2) dipole length is reproduced with the error of 6.6, −7.7, −1.9% (−13.5, −18, −16.5%) for the *pc*, *al*, and *wc* conformers, respectively, i.e., this property is reproduced with medium quality. However, the HF (MP2) average polarizability is reproduced with percent errors of 0.6, 1.4, and 3.2% (0.6, 1.5, 2.9%) for these conformers, respectively, which is a much better result. The average polarizability is a tensor invariant, but it is also interesting to examine off-diagonal terms in a given coordinate system since these terms are much smaller than diagonal terms and are more sensitive to applied approximations (see, e.g., the investigation of the influence of local approximations for static polarizabilities [104]). In particular, it is interesting to see whether the balancing of the fragmentation terms with plus and minus signs is accurate enough to reproduce zero or close-to-zero off-diagonal terms for the unfragmented molecule from possibly large (in terms of absolute values) off-diagonal terms for fragments. It turns out that this is indeed the case and, e.g., a small term $\alpha_{xz}=-1.9$ a.u. for the *al* conformer is reproduced as −1.3 a.u. as a summation of terms, which are individually as large as about ±30 a.u. Therefore, one can conclude that the molecular fragmentation model is useful for a semiqualitative reproduction of first-order molecular properties, while it behaves better for second-order properties if the third level of fragmentation is employed. It should be noted that nonbonded parts of the property were omitted here for the sake of simplicity. A full comparison of the dipole moment and polarizability components is presented in the Supplementary Information.

### 3.9. Complexes with Amino Acids–Energy Partitioning with SSMF and F-SAPT

The energy partitioning in the SMF approaches is a byproduct of the SMF procedure and sometimes two interesting groups are placed in the same fragments, and thus, impossible to separate. However, in many other cases, they are sometimes attributed to different fragments, and then by the elimination analysis one can establish which molecular group in a given molecule is responsible for the highest interaction strength. The systematic way of performing such an analysis consists in a selection of those interaction energies between the calixarene fragment and the amino acid (see Equation (3)), which have the highest absolute values, and the following investigation of how the removal or addition of a neighboring unit influences the interaction energy with the amino acid.

#### 3.9.1. Special Case: SSMF3 and F-SAPT Partitioning Analyses for Complexes of CX⋯Gly

**Figure 6:**
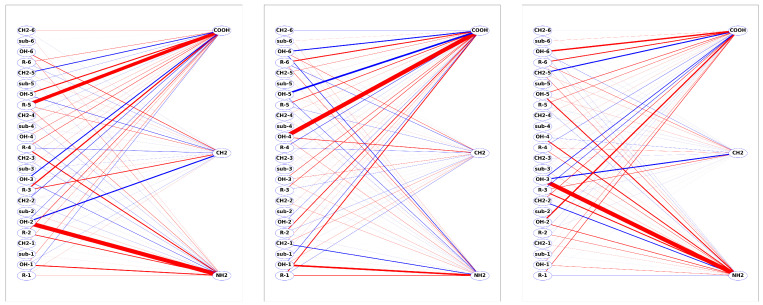
The F-SAPT interaction energy graph for the Gly amino acid interacting with the *al*-CX (leftmost graph), *pc*-CX (middle graph), and *wc*-CX (rightmost graph). Calixarene functional groups are depicted on the left and those for Gly–on the right. The red (blue) lines denote attraction (repulsion), and their thickness is proportional to the interaction strength.

**Figure 7:**
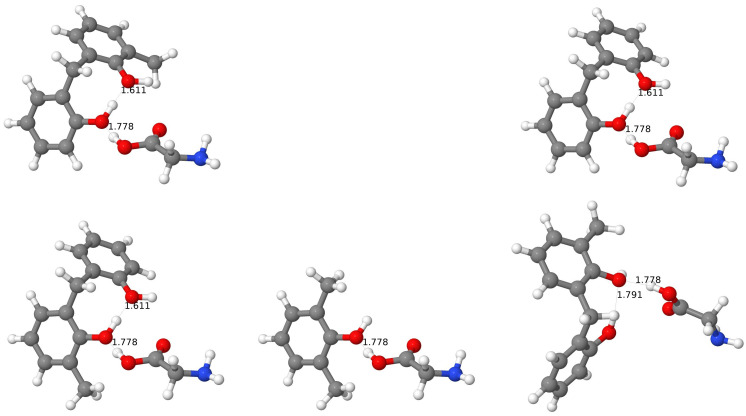
The fragments 7 to 11 generated from the SSMF3 partitioning of the *pc*-CX interacting with Gly. The numeration goes row-wise from top left to bottom right. Selected H-bond distances (in Å) are shown in order to identify their placement in the original calixarene.

The SAPT0 interaction energies of 24 pairs (fragment⋯Gly), resulting from the SSMF3 fragmentation of the CX, are presented in Table 4 for *al*, *pc*, and *wc* conformers of CX, while the corresponding F-SAPT partitionings are presented in Figure 6. One immediately sees that the resulting interaction energies are completely different for these three cases. Let us analyze these differences in more detail, starting from the *pc* conformer. In this case, the SSMF3-SAPT0 fragmentation energies with the largest absolute values correspond to fragments 7, 8, and 9 (see Figure 7). All these fragments contain the same hydroxy group OH-4, which donates its oxygen atom to a strong H-bond with the hydroxy group of Gly (the 1.78 Å distance between the H-ending of the glycine hydroxy group and the oxygen atom from the OH-4). Removal or addition of the methyl group does not have any influence on this energy, which is equal to −18.5 mH. However, it would be incorrect to attribute this energy to the isolated H-bond since the removal of the phenyl ring with the attached OH-3 group reduces the attraction by as much as 5 mH (see fragment 10). The weaker attraction cannot be simply explained as a lack of the attraction between Gly and Ph-3 or OH-3, because the fragment 11, which possesses the same OH-4 group bound to Gly but additionally has the phenyl ring with the OH-5 group on another side, reduces the attraction with Gly by another 5 mH. Therefore, the relative position of the second unit is crucial. One can presume that for fragments 7 to 9 the interaction of OH-4 with OH-3 plays a role since according to the I-SAPT partitioning (see Table 3) there is a strong intramolecular H-bond between these two groups. From geometrical considerations and from a more detailed analysis of induction components, one can see that the OH-4 group donates the H atom while the OH-3 group is the H acceptor, which results in shifting more negative charge to oxygen in OH-4 and making it a better H acceptor for the COOH-Gly. An opposite situation arises for the fragment 11, where the OH-4 group accepts the hydrogen atom from the OH-5 group, what makes the O-4 atom less negative, leading to a weakening of the attraction between the OH-4 and the OH-Gly group. The SSMF3 fragmentation is not detailed enough to directly separate contributions from phenyl ring, methylene, or hydroxy groups, but the F-SAPT partitioning of the complex reveals that indeed *(i)* the attraction of the carboxy group of Gly with the OH-4 is the strongest one (−10 mH), *(ii)* there is an additional attraction of COOH-Gly to the OH-3 and Ph-3 groups (−2 mH), which explains why fragment 10, deprived of those groups, shows a weaker attraction, and *(iii)* there is a relatively strong repulsion between COOH and OH-5 (+4 mH), which explains a reduction in attraction for fragment 11. It should be noted that the attraction between the carboxy group of Gly and the OH-4 group is a net result of a balance between several components of similar importance, such as electrostatics, induction, dispersion and exchange. A significant exchange term indicates that electron clouds of these two groups overlap as in the case of covalent bonds, but the magnitude, which is smaller than for typical covalent bonds, classifies this bond as noncovalent. Therefore, the nonzero exchange and other SAPT components signify that the H-bond should exist between some atoms of COOH-Gly and OH-4 (note that according to the IUPAC criteria atoms participating in H-bonds should be close to each other so that the distance between them was smaller than the sum of atomic Van der Waals radii [121]). If the SSMF3 contributions of a range of about −9 mH are analyzed in the same manner (not shown in the figure), a secondary binding is revealed, which can be attributed to the interaction between NH_2_-Gly and the OH-1 groups with the amino group being the H donor. As it could be guessed from a relatively large distance between the hydrogen of NH_2_ and oxygen of the OH-1 (2.23 Å) and from the value of the H⋯OH-1 angle (143${}^{\circ }$), which is quite different from the full angle, this interaction should be quite weak, but nonetheless, it is still composed of nonzero polarization and exchange contribution, which sum up to −4 mH according to F-SAPT, therefore we can still classify it as an H-bond.

**Figure 8:**
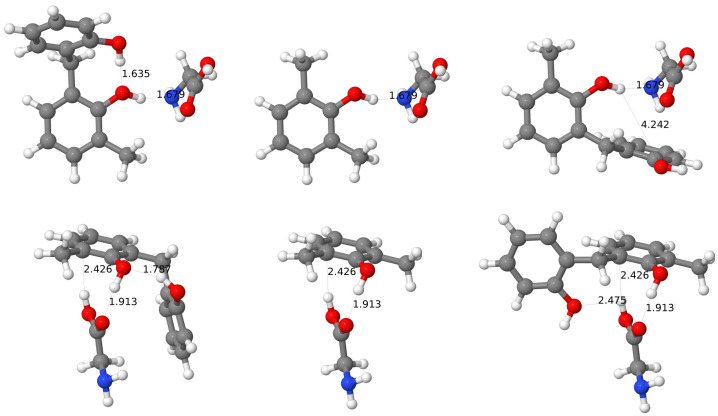
The fragments 1 to 3 and 13 to 15 generated from the SSMF3 partitioning of the *al*-CX interacting with Gly. The numeration goes row-wise from top left to bottom right.

For the *al*-CX⋯Gly complex, there exists much more important terms since the Gly molecule resides in the *al* cavity, and in Figure 8, we present only those fragments, which are the most relevant to the discussion below. The important fragments can be segregated into those corresponding to the binding to the NH_2_-Gly and COOH-Gly sites and–contrary to the *pc* case–these two sets are of similar importance. The most attractive contributions for the first and second sites come from fragments 1 and 13 and amount to −25 and −26 mH, respectively. Fragments 1 to 3 have an H-bond between the OH-2 and NH_2_ groups, while fragments 13 to 15–between OH-5 and COOH. Similarly, as in the *pc*-CX case above, the removal of the phenyl ring with the hydroxy group, serving as a hydrogen donor in the intramolecular H-bond, reduces the interaction energy by about 7 mH (fragments 2 and 14). However, contrary to the *pc*-CX case, the addition of the n+1th calixarene unit (number 3 for the fragment 3 and number 6 for the fragment 15) does not lead to a further reduction in attraction. Quite the opposite, a small rise of the attraction (by 3-4 mH) is observed in comparison to fragments 2 and 14. The geometry analysis shows that these additional phenyl and hydroxy groups are more twisted in comparison to the *pc* case, so that the creation of the H-bond between OH-2 and OH-3 or between OH-5 and OH-6 is prevented, and such bonds would weaken the negative character of the O-2 and O-5 atoms. What remains to be explained is the increase in the attraction for fragment 3 in comparison to fragment 2 (and for fragment 15 in comparison to 14). Since these fragments differ by other phenyl plus hydroxy groups, one (or both) of these groups should be responsible for this phenomenon. Because of the limitations of the SSMF3 partitioning, the explanation of this fact should be postponed till the F-SAPT analysis is made. Now let us move to a complementary view of the F-SAPT partitioning. As expected, there is a strong (−14 mH) binding between the OH-2 and NH_2_ groups. Surprisingly, the interaction between OH-5 and COOH amounts to −3.5 mH only, but the carboxy group is strongly attracted to the Ph-5 group (−11.5 mH). All F-SAPT components, including the exchange one, are significant for the COOH⋯Ph-5 interaction; therefore, the F-SAPT analysis reveals the existence of the untypical H⋯π H-bond. Such interactions were reported, e.g., in molecules containing aromatic rings with those with the S-H bond [122]. Another candidate for the H⋯π H-bond would be the H atom from the NH_2_ group interacting with the Ph-3 group. However, in this case, the total interaction energy is close to zero, which is a result of a perfect cancellation of several contributions of similar magnitude (the electrostatics of −3 mH and dispersion of −4 mH are counterbalanced by the large exchange component). The question remains why fragments 2 and 3 differ in attraction by as much as 4 mH. A perusal of the F-SAPT partitioning table reveals that this difference is due to the electrostatic attraction (−4 mH) between the Ph-3 and COOH groups. Finally, a similar difference for fragments 14 and 15 can be explained by the net attraction to the OH-6 group (−2.5 mH).

**Figure 9:**
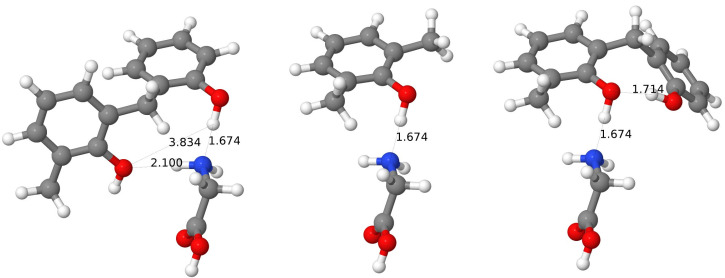
The fragments 3, 6, and 7 generated from the SSMF3 partitioning of the *wc*-CX interacting with Gly. The numeration goes from left to right.

The most important contributions from the SSMF3 partitioning for the *wc*-CX⋯Gly complex provide interaction energies of about −22 to −24 mH (see two representative fragments 3 and 7 in Figure 9) and have in common the H-bond between the OH-3 and NH_2_ with the latter group as the H acceptor (the H⋯N bond of 1.67 Å). As in the previous cases, this energy cannot be attributed to this one H-bond only, since, e.g., fragment 6 containing this H-bond attracts the Gly molecule weaker by 8 mH. The geometry considerations allow us to point to a possible additional interaction with the OH-2 group for fragment 3 since the O⋯H distance in this case is equal to 2.10 Å, that is, the amino group of Gly donates its hydrogen to create a weak H-bond. The mechanism of an increased attraction in case of the fragment 7 is different: here the intramolecular H-bond between the OH-3 and OH-4 groups makes the O-3 oxygen less negative, thus allowing the H-3 to interact more strongly with the nitrogen from the amino group. Not shown in the figure are fragments 15 to 17, for which two interactions: between the amino group and OH-5 (the NH_2_ as H donor) and between the carboxy group and OH-6 (COOH as the H acceptor) can be guessed from the geometry considerations. The F-SAPT partitioning confirms these predictions. First, a strong interaction between the OH-3 and NH_2_ groups has been obtained with this method (−13 mH). There are also three weak H-bonds (again recognized by the significance of all SAPT components, including exchange): between OH-6 and COOH (−4 mH), OH-5 and NH_2_ (−3 mH), and OH-2 and NH_2_ (−2 mH). The latter interaction is too weak to explain a difference between the attraction from fragments 3 and 6, but the F-SAPT provides the additional strong attraction of a purely electrostatic type between the OH-2 and carboxy group (−4 mH) to fill this gap.

Summarizing, all three cases of the interaction with the simplest Gly amino acid provide different mechanisms for secondary stabilizing interactions, which would be difficult to elucidate just from the analysis of the total energies.

#### 3.9.2. Special Case: SSMF3 and F-SAPT Partitioning Analyses for Complexes of BCX⋯Gly

The SSMF3 model for hexa-*p*-*tert*-butylcalix[6]arene produces as many as 150 fragments; therefore, the tables analogous to Table 4 were shifted to the Supplementary Information. The SAPT0 interaction energies for complexes *al*-BCX⋯Gly and *wc*-BCX⋯Gly are close to those for the CX counterpart (−59.7 mH and −57.7 mH for *al*, −52.0 mH and −50.0 mH for *wc*, for CX and BCX, respectively), but the for *pc*-BCX the interaction energy is 11 mH lower than for the unsubstituted case. Naively, one could assume that it is the attraction with the *tert*-butyl groups, which makes the total interaction energy more negative, but the sum of interaction energies between fragments made from *tert*-butyl with Gly gives a negligible contribution. Therefore, the influence of the *tert*-butyl substituents is more subtle–their presence leads to geometry modifications, which, in turn, allow for a better arrangement of Gly on top of the *pc*-BCX. Similarly, as in the unsubstituted case, there is a set of interaction energies of about −19 mH each, which contain the same type of an H-bond between the carboxyl group of Gly and the OH-1 group (note that for the pristine calixarene OH-1 and OH-4 groups etc. are equivalent, see Figure 2). Again, the presence of the calixarene unit 2 causes an enhancement of the electronegative character of the O-1 atom through the intramolecular H-bond with the OH-2 group, which explains its weaker attraction to Gly (to −13 mH for fragments with the unit 2 removed). The first significant difference appears for numerical values of interaction energies for fragments with the unit added on the other side (the unit 6) of the unit 1. Although the intramolecular H-bond between OH-1 and OH-6 is created, as in the CX case, it weakens the attraction to Gly by 2 mH only, while for the CX case this change amounts to 5 mH. Still, this difference alone does not explain the 11 mH gap between the total interaction energies for the CX and BCX for this conformer, and other differing factors should be looked for. It turns out that the missing difference can be found from the examination of fragments, which contain units 4 and 3 of BCX and have interaction energies of −14 to −15 mH. In these fragments, one H-bond between the H atom from the OH-4 group and the NH_2_ group of Gly can be found, while the neighboring OH-3 group forms the intramolecular H-bond acting as the H donor, i.e., it enhances the negative character of the O-4 atom. The latter explanation is confirmed by comparison with fragments without the OH-3 group, for which the interaction energy changes to −10 mH. Summarizing, in comparison to the CX case, the interaction with the NH_2_ group is much stronger and it is this interaction which according to the SSMF3 partitioning is responsible for the stronger attractive force for the *pc*-BCX in comparison to the unsubstituted calixarene. It is also worth noting that the direction of the primary H-bond for the NH_2_ group changes, i.e., this group becomes the H acceptor for the BCX case. The F-SAPT partitioning confirms these findings: there is still a strong bond of −15 mH between the COOH and OH-1 groups, but the attraction of the OH-4 to the amino group is stronger (−15 mH) than for the CX case. This new intermolecular H-bond causes a distortion of one from the intramolecular H-bonds in the BCX, what can be also observed from the pattern of I-SAPT interaction energies between the hydroxy groups, where instead of one strong attraction of −15 mH between OH-1 and OH-6 only a weak one of −4 mH remains.

The *al*-BCX⋯Gly complex is of the inclusion type, and the Gly molecule resides in the cavity created by units 3, 4, and 5 of the BCX, as for the *al*-CX counterpart. The fragments which give the largest attractive SSMF3 contributions (−18 mH) contain the phenyl and hydroxy groups from units 1 and 2, and–similarly as for the *al*-CX case – one of them donates its H atom to the NH_2_-Gly group forming an H-bond, and another one enhances this interaction through a creation of the intramolecular H-bond with the O-2 atom. This effect can be estimated as about 6 mH, based on the energy of the fragment without the unit 1 (−12 mH). There is another group of fragments with no analogs for the CX case, which gives rise to contributions of about −15 mH. It turns out that they all contain the H-bond between NH_2_ (the H donor) and the OH-6 group (H⋯O distance of 2.005 Å) and additionally possess the phenyl and hydroxy groups from the neighboring unit 5. The lack of these two groups leads to a strong decrease in attraction (to −8 mH), but for the *al*-BCX geometry, the intramolecular H-bond between the OH-5 and OH-6 groups cannot exist; therefore, the reason for a large attraction in the former case is the direct interaction of the phenyl and hydroxy groups of unit 5 with Gly. The latter conclusion is confirmed by the interaction energy of about −9 mH for fragments, which contain the Ph-5 and OH-5 groups, but no other groups of these types. It should be noted that a relatively large distance between Gly and such fragments suggests that the interaction should be of an electrostatic type since electrostatic contributions are known to be long-ranged. There are also fragments containing the phenyl and hydroxy groups of the unit 4, which attract Gly with the strength of −10 mH. It is evident from the geometry analysis that the OH-4 group cannot effectively participate in any H-bond with Gly, but since the carboxy group is positioned quite close to the Ph-4 (the closest distance between the hydrogen atom of COOH and the carbon atom is about 2.5 Å), one can predict a formation of an unusual H-bond between this hydrogen and the π cloud of the phenyl ring. Note that this distribution of interaction strengths is different from the *al*-CX case, where contributions as large as −26 mH are present, so–surprisingly–such close total interaction energies result from the summation of contributions of a partially different origin. The F-SAPT partitioning confirms the existence of strong bonding between the OH-2 and NH_2_ groups (−21 mH), as in the CX case. However, contrary to the CX case, the carboxy group is attracted mostly not to the Ph-5 group, but to a closer Ph-4 group with a remarkable strength of −24 mH. Nevertheless, in both cases, the existence of the H-π bond can be postulated based on the position of the hydrogen atom and on the analysis of SAPT components. Another feature of the binding pattern is the existence of a secondary H-bond, in which the NH_2_ group donates a hydrogen atom. This H-bond can be identified based on the analysis of the interaction between the NH_2_ and OH-6 (−5 mH, with about +10 mH from the exchange component). The strong attraction from unit 5 found in the SSMF3 partitioning is also reproduced here as a strong electrostatic-dominated interaction between the COOH and Ph-5 (−10 mH). It should be emphasized that the F-SAPT and SSMF3 partitionings for the *al*-CX and *al*-BCX complexes with Gly reveal that the Gly molecule is attracted by the cavity from several sites with similar strength. The geometry analysis shows that this relatively small molecule seems to fit well into the small cavity of the *al* conformer; therefore, these complexes represent examples of the enhancement of the interaction due to a confinement effect.

The main features of the complex of Gly with the *wc*-BCX are similar to the *wc*-CX case. The first set of interaction energies of about −23.5 mH corresponds to fragments containing the phenyl groups plus hydroxy groups from units 5 and 6. The OH-5 group serves as an H donor, and the OH-6–as an H acceptor for two H-bonds with the NH_2_-Gly. Since the fragment without the OH-5 group, but with the OH-6 group remaining, has the interaction energy reduced to −16 mH, the strength of the second H bond can be estimated as about 7 mH. The addition of the phenyl and OH groups from the unit 1 leads to the increased attraction (−21 mH), which can be explained by the intramolecular H-bond creation between the OH-6 and OH-1 groups, which increases the electronegative character of the O-6 atom (this H-bond is also seen from the I-SAPT analysis). The next sets of interaction energies correspond to the fragments containing either both phenyl and hydroxy groups from units 2 and 3 (energies of −15.5 mH), or having groups from unit 3 only, which reduces the interaction to −10 mH. Since the OH-3 group donates its H atom to the oxygen from the carbonyl group of Gly (the H⋯O distance of 1.99 Å) and the OH-2 group is a hydrogen acceptor for the NH_2_ group; therefore, two H-bonds are present here, and the H-bond between the OH-2 and amino groups can be estimated as about 5.5 mH from a difference analysis. The F-SAPT partitioning results are in line with these findings. Firstly, the most important interaction of −19 mH exists between the amino and OH-6 groups. The carboxy group forms a weaker bond with the OH-3 group with the strength of −7 mH. The analysis of SAPT components confirms that these two bonds are H-bonds. The amino group is also connected with the groups OH-1 and OH-2, but from these two pairs, the first one is dominated by electrostatics, while the second again represents an H-bond. It is interesting to note that the distance between the corresponding hydrogen atoms of the amino group and oxygen atoms of the hydroxy groups is only 0.15 Å longer (2.22 *versus* 2.04 Å) for the electrostatic driven interaction and differences in the total interaction energies are also not very large (−4 mH *versus*−6 mH). Nevertheless, in the second case, one has a significant first-order exchange component of 7 mH, which is counterbalanced by other components, from which the electrostatics gives the most negative contribution (−9 mH). It should be noted that for the CX case, both these interactions were of the H-bond type.

#### 3.9.3. Special Cases: Analysis of *pc*- CX Inclusion Complexes

Let us use the F-SAPT partitioning to examine in detail the only four cases, for which the *pc* conformer of CX prefers to form an inclusion complex with an amino acid (Ile, Lys, Val, and Pro). Since the dispersion energy can be only negative and its magnitude is correlated with the number of electrons, it can be used to locate electron-rich environments. Indeed, for all four cases, one can identify pairs of functional groups (one from CX and one from an amino acid), which are close to each other and have a large number of electrons. In particular, a perusal of the dispersion graphs (see the Supplementary Information) allows us to instantly locate the guest molecule into one from two parts of the *pc*-conformer cavity. (Note that if the dispersion component is significant, then usually other SAPT contributions are nonzero and we deal with a potential candidate for a noncovalent bonding, such as the H-bond.) The next components to examine are the electrostatic and induction energies. One can see that indeed, depending on a relative orientation, some pairs attract each other more strongly than it would be possible with dispersion only. However, the analysis of these three components only is not sufficient to guess the total interaction strength, since many of these components are quenched by short-range first-order exchange contribution. One should emphasize again that the presence of nonzero exchange components together with the polarization (induction and dispersion) components show that we have to deal with a noncovalent bond.

Let us first consider the *pc*-CX⋯Ile case. The F-SAPT reveals here a strong attraction between the OH-2 group of the calixarene and the COOH group of Ile, which can be explained by the creation of a weak H-bond (the O⋯H distance of 2.0 Å). The F-SAPT estimation of this bond strength can be given by the interaction energy of the OH-2 and COOH groups and it amounts to −9 mH, which should be compared with −10 mH for the intramolecular attraction of two adjacent hydroxy groups in calixarene. This interaction is not the only one in this complex. Moreover, the Ph-6 ring is attracted to the carboxy group, but it is also strongly repelled by the neighboring CH group. It is also interesting that the chain of six intra-calixarene H-bonds is not destroyed in spite of a contribution of OH-2 to another H-bond, which can be seen in the examination of the interaction between the hydroxy groups of calixarene with help of I-SAPT (there are still six interaction energies between these groups of value below −10 mH).

For the complex of *pc*-CX with Pro the F-SAPT partitioning of dispersion energies reveals that the ring of Pro is strongly attracted by the calixarene phenyl rings on one part of the macrocycle (with Ph-4, Ph-3, and Ph-5), which agrees with the fact that the Pro amino acid occupies just one side of the calixarene cavity. For the interaction with Ph-4 and Ph-5, there is some attractive electrostatic component, too. However, the major part of the interaction comes from the attraction of the COOH group of Pro with Ph-3, which has a predominantly electrostatic origin. It turns out that a strong attractive dispersion interaction of the Pro-ring with three phenyl rings is to a large extent counterweighted by a repulsive exchange interaction, leaving a weak net attraction for these three pairs. It is also interesting to note that practically no interaction exists between Pro and the hydroxy groups of calixarene, in spite of the fact that both Pro fragments are polar and close to at least the OH-3, OH-4, and OH-5 groups. Apparently, the sextet of H-bonds makes the hydroxy group less polar, so they are not involved even in a long-range electrostatic interaction (it should be also noted that the H-bonds’ distances range between 1.63 to 1.69 Å, i.e., they do not elongate upon the complexation with Pro).

In the complex with the Lys molecule, there are three Ph groups, which are mostly responsible for the net attraction. From the Lys side, all major polar groups contribute to the interaction and it is impossible to select one or two major interacting pairs. One can note that one from two NH_2_ groups of Lys is involved in a secondary bond with the nearest Ph-2 ring. This interaction of −3.2 mH is composed of significant attractive electrostatic and dispersion components and is quenched by the exchange. Interestingly, the interaction of another amino group with the same ring is of a very similar value (−3.7 mH), but is predominantly of the electrostatic character. The hydroxy groups of calixarene do not contribute to these interactions in a significant way (what can be also seen by practically fixed lengths of H-bonds similar to the empty calixarene). It turns out that the COOH and NH_2_ groups of Lys connect through an intramolecular H-bond, which apparently depolarizes this molecule to some extent and makes it a weaker target for calixarene.

Finally, for the complex with Val again the net attraction comes from the same three Ph groups, but in this case, one can pinpoint the COOH group as the main partner on the Val side. The interaction between the COOH group and Ph-1, Ph-2, and Ph-6 is of mostly electrostatic character. There are several cases where the exchange contribution is relatively large, but, e.g., for the largest exchange contribution amounting to +5 mH (the Ph-1 with the amino group) the electrostatic one is close to zero and after adding the dispersion component a small net interaction energy below 1 mH remains. The alkyl skeleton gives together a weak attraction of −1 mH, but the closest CH group is oriented in a way that produces repulsive electrostatics. Therefore, all potential candidates for a formation of weak noncovalent bonds are excluded in this way and altogether the Val@*pc*-CX complex can be described as the electrostatically bound complex.

#### 3.9.4. Types of Noncovalent Bonding in Calixarene-Amino Acid Complexes

As shown in Section 3.9.1, Section 3.9.2 and Section 3.9.3, the F-SAPT analysis is very useful as a tool for the identification of various types of noncovalent bonds. A comprehensive study of all 120 complexes reveals many more interesting bindings, among which the various sorts of H-bonds form an important group. The IUPAC classification of H-bonds [51,54,121] provides the following H-bond criteria, which may be useful to browse through the present data: *(i)* their energy (63–167 $\mathrm{kJ}\cdot {\mathrm{mol}}^{-1}$ for strong, 17–36 $\mathrm{kJ}\cdot {\mathrm{mol}}^{-1}$ for medium, and less than 17 $\mathrm{kJ}\cdot {\mathrm{mol}}^{-1}$ for weak hydrogen bonds); *(ii)* a large contribution to the electrostatic interaction (with a nonnegligible dispersion), and geometry constraints; such as *(iii)* a preferable alignment of a hydrogen donor, a hydrogen atom, and a hydrogen acceptor and; *(iv)* distances between interacting atoms, which should be significantly smaller than the sum of the Van der Waals radii. The SAPT approach has been applied many times for studying H-bonds, such as, e.g., in the comprehensive study of the behavior of various SAPT components for the water dimer [123], where the near-linear preference of the H-O⋯H atoms has been explained by a different angle dependence of first- and second-order SAPT energy components, with the prevailing importance of the second-order induction and dispersion. Therefore, from the SAPT point of view, one should examine not only the pure electrostatic contribution as a fingerprint of the H-bond but also the exchange component, which indicates how large the overlap of the electron clouds of the hydrogen is, the hydrogen donor and the hydrogen acceptor (which is indirectly implied in the H-bond classification by the requirement of a small enough distance between atoms).

The interaction types for all pairs of groups (i.e., 24 groups for a calixarene and a differing number of groups for amino acids) can be first classified according to their total interaction energy $E_{\mathrm{int}}$. According to the IUPAC criteria (64–24 mH–strong, 24–6.5 mH–medium, below 6.5 mH–weak H-bonds), almost all bondings present in calixarenes belong to medium or weak types (note, however, that this criterion is of a limited value here, as the F-SAPT energies are calculated between functional groups containing more atoms than just three involved in an H-bond). The strength of the pair interaction does not imply automatically the H-bond character, although most of the strongest pairs do contain typical H-bonds, i.e., these interactions occur between hydroxy groups of calixarenes and, e.g., hydroxy groups (usually from the carboxy group) or amino groups of amino acids.

The interaction type can be established through the analysis of weights of SAPT components with respect to the interaction energy, i.e., by examining the ratios: $|E_{\mathrm{elst}}^{\left(1\right)}/E_{\mathrm{int}}|$, $|E_{\mathrm{exch}}^{\left(1\right)}/E_{\mathrm{int}}|$, $|E_{\mathrm{ind},\mathrm{eff}}^{\left(2\right)}/E_{\mathrm{int}}|$, and $|E_{\mathrm{disp},\mathrm{eff}}^{\left(2\right)}/E_{\mathrm{int}}|$, where the “eff" subscript denotes effective components as described in Section 2.5. In particular, a large contribution of the exchange energy signifies that both functional groups are close enough for the electron exchange and are a necessary prerequisite of a true bonding (of course, it could also signify a repulsion). A large exchange contribution usually implies that the remaining ratios are nonnegligible. If the only significant contribution is the electrostatic energy, then the functional groups do not share the electron cloud and their attraction or repulsion can be described classically *via* the Coulomb law. The selection of a cutoff value for such ratios is to a large extent arbitrary, and we assumed that if a given component accounts for at least 30% of the interaction energy, the contribution from this component is significant.

The F-SAPT partitioning for selected pairs is presented in Table 5, while tables for all pairs are shifted to the Supplementary Information. A representative of a typical moderately strong H-bond can be characterized by high ratios for electrostatic, exchange, and induction–usually above 1.0 for both first-order components and above 0.5 for the induction and by a smaller contribution from the dispersion (below 0.3). The contribution from the dispersion rises for special cases, such as Pro or His, where a ring with a heteroatom (nitrogen) with the attached hydrogen serves as a hydrogen donor, but additionally, the ring itself interacts with the calixarene hydroxy group, which occurs to a large extent through the dispersion interaction. It should be noted that in such cases, the first three ratios remain high, similar to other H-bonds discussed so far.

Apart from such cases, there are also several moderately strong bondings, which are characterized by a different ratio pattern. Usually, they arise from the interaction of a phenyl ring of calixarene with a polar group belonging to an amino acid. They are characterized by a lower ratio for the exchange (much lower than 1, but not less than 0.3), the still high ratio for electrostatics, and usually by smaller ratios for induction and dispersion (about 0.2–0.3).

An examination of the relative position of the calixarene phenyl ring in question and the hydrogen atom from the polar group of the amino acid reveals that such cases correspond to a partial donation of the hydrogen atom to the ring, i.e., here, the untypical H-bond is detected (H⋯π bond), where the phenyl ring serves as a hydrogen acceptor. One should add that usually an elongation of the H–X bond in comparison to the isolated amino acid is found in such cases, similarly to typical H-bonds.

The next characteristic pattern occurs for the interaction between a calixarene phenyl ring and a ring of an amino acid (e.g., for His). For such interactions there is still a high electrostatics ratio, the exchange ratio is higher than for the H⋯π case, but lower than for a typical H-bond (usually about 0.5), and finally, a large dispersion contribution amounts to at least 0.5. Summarizing, in comparison to all other cases, these pairs are to a large extent dispersion-bound, especially if one takes into account that the electrostatics and first-order exchange contributions partially cancel each other.

One should add that even among quite strong interactions one could find electrostatic-driven ones, especially for the interaction of a calixarene ring with a polar group of the amino acid; therefore, the ratio check of components is usually necessary in order to determine the interaction type. In the case of calixarenes, the macrocyclic structure represents an obstacle in relaxing the geometry after the complexation. In some cases, this means that apart from the dominant attractive pairs, some other pairs exist, for which the interaction energy becomes zero or is even positive (repulsive). In the interaction table, the largest such contributions amount to 7–8 mH. Among them, there are pairs that repel each other electrostatically, but also those that are close enough to have a large exchange contribution.

The sorting of F-SAPT total energies for pair interactions reveals in many cases two or more pairs with interaction energies of a similar value (which one can set arbitrarily for, e.g., no more than 50% difference). Among such cases, both inclusion and outer complexes can be found. For the inclusion complexes, this analysis helps to identify the confinement (encapsulation) effects, i.e., those cases where an amino acid occupies the calixarene cavity and utilizes its active groups to bind with several groups of the host. One such example has already been presented in Section 3.9.1 and Section 3.9.2, where the *al* conformers of both CX and BCX use both polar groups of Gly to encapsulate it effectively in the cavity. More such complexes can be found, e.g., for the *pc*-CX case for complexes with Val, Pro, and Ile and for the *pc*-BCX one–with Pro, GluH, Ala, and AspH. In all these cases, the guest molecule fits into one of two cavities created by three calixarene units and the dihydrogen bridge. Other complexes with the confinement effect are: complexes with Ile, Val, Tyr, Thr, and Ser for the *al*-CX, and complexes with Val, Trp, Thr, Ser, Pro, Met, HisD, Gln, AspH, and Asn for the *al*-BCX. It should be noted that in the case of partial confinement, which we have in the case of calixarenes, the cage (or better saying: half-cage) has more flexibility to adapt itself for a particular guest than an inherently rigid full cage, such as in fullerenes. In all these cases, the guest molecules are attached in the cavity by at least two comparable interactions, which are usually (but not always) the proper H-bonds.

The same analysis for the outer complexes reveals that a general classification into: either cases for which one molecule has a dominating group interacting with at least two groups of another molecule, or cases for which each molecule has at least two such groups. The first scheme corresponds to the pattern $A_{1}↔B_{1}⋀A_{1}↔B_{2}$, and the second–to $A_{1}↔B_{1}⋀A_{2}↔B_{2}$, where $A_{i}$ and $B_{i}$ are functional groups of molecules A and B. The first case can be named, similarly as in the ligand theory, as di- (or in general: poly-) dentate complex, while the second case–as di- (or in general: poly-) site complex. The di-dentate complexes are the most common. Among several examples, one can name the complex of the *pc*-CX with Gln, where the Gln molecule uses its carboxy group to make two H-bonds with the OH-1 and OH-6 groups (of similar strength of about −9 mH), or with Tyr, for which the carboxy group binds electrostatically with the Ph-3 and Ph-2 groups. Yet another example of this type is the *pc*-BCX complex with Asn, where the carboxy group forms two H-bonds with two hydroxy groups of calixarene, with the interaction energies of −13 and −11 mH.

The poly-site complexes are, e.g., complexes of the *pc*-CX with Ala, where two H-bonds are created: between the OH-4 and NH_2_ groups and between the OH-1 and COOH groups with the interaction energies of −17 mH and −15 mH, respectively, or with AspH, where again two opposite hydroxy groups (OH-3 and OH-6) form H-bonds with two AspH carboxy groups. For the *wc*-CX case, the complex with AspH is particularly interesting, since the examination of the SAPT energy decomposition allows us to identify two H-bonds as for the *pc* case, but additionally both COOH groups interact electrostatically with other groups of calixarene with total energies of the same magnitude. Other *wc*-CX examples of poly-site binding are: *(i)* Gln, where two large groups (COOH and CONH) each form two H-bonds with hydroxy groups of calixarene (the strongest two interactions amount to −18 and −17 mH); *(ii)* HisE, where one typical H-bond is formed between the OH-3 and NH_2_ groups, but additionally a strong dispersion-dominated interaction between two rings (the Ph-2 group with the HisE ring) occurs (the first interaction amounts to −20 mH, and the second–to −13 mH); *(iii)* Leu or Lys, where two H-bonds are formed: one with help of the COOH, and the second–with the NH_2_ group.

There is also a number of poly-site complexes for the hexa-*p*-*tert*-butylcalix[6]arene case, such as, e.g., already discussed *pc*-BCX⋯Gly case, see Section 3.9.2. For the *wc*-BCX complex with HisE an analogous situation appears as for the CX case with very similar interaction energies of −18 and −15 mH for the H-bond and the dispersion-dominated bond, respectively. The same pair of interactions exists for the complex with Trp, where apart from a typical H-bond between the OH-6 and NH_2_ groups there exists a dispersion-dominated interaction between the Ph-5 and Trp rings (of energies of −17 and −15 mH, respectively). Other interesting cases are: *(i)* the complex with Thr, where apart from a typical H-bond between the OH-3 and NH_2_, the Ph-6 and carboxy groups attract each other electrostatically (both bonds of strength −16 mH) and; *(ii)* a similar pair of H-bond and electrostatic interactions in the complex with Pro, where the nitrogen from the Pro ring forms the H-bond, while the COOH group is attracted by the phenyl ring (with the interaction energies of −14 and −15 mH, respectively), or; *(iii)* the interaction with Met, where again pairs OH-6 with NH_2_ and Ph-3 with COOH form the H-bond and the electrostatic-dominated interaction of the same strength of −14 mH. There are also cases of typical H-bonds of similar strength, such as in the complex with Ser (the OH-6 with NH_2_ and the OH-3 with OH-Ser groups with interaction energies of −19 and −13 mH, respectively), with Lys (interaction energies of about −20 mH) or with GluH (−19 mH).

Many more similar examples can be found after the analysis of the F-SAPT interaction energy partitioning, listed in the Supplementary Information.

## 4. Conclusions

Three calix[6]arene and hexa-*p*-*tert*-butylcalix[6]arene conformers and their most stable complexes with amino acids were studied with DFT+D, SSMF, MP2, SCS-MP2, SAPT0, and F-SAPT methods.

The *pc* (pinched-cone) conformer is the most stable for the pristine calix[6]arene and hexa-*p*-*tert*-butylcalix[6]arene. A ring of six H-bonds strongly stabilizes the calixarene pinched-cone shape, with a strength of the H-bond of about −15 mH, according to the I-SAPT analysis. This ring partially closes the top of the calixarene molecule. No significant differences between calix[6]arene and hexa-*p*-*tert*-butylcalix[6]arene stability of the *pc* conformer have been found. The *wc* (winged-cone) and *al* (alternate) conformers each have two triples of hydroxy groups connected by H-bonds, which are weaker than for the *pc* case (−10 mH and −12 mH).

The energetic order of complexes with the same amino acid usually differs from the stability order of empty calixarene conformers. The interaction energy order is different too, which can be attributed to deformations, which strongly depend on the conformer and on the amino acid type. The absolute values of interaction energies of the *pc* conformers are about two times smaller than those for the *wc* and *al* ones, indicating that because of the more stable structure the *pc* conformer is less prone to interact with polar molecules such as amino acids. No molecule was able to penetrate inside the *wc* conformer, while for other conformers both inclusion and outer complexes are found as the lowest complex conformers.

Systematic molecular fragmentation is proven to reproduce the interaction energy of the complexes with good accuracy. Especially the dispersion energy (i.e., electron-correlated part of the interaction energy) is well suited to be treated by the SSMF3 approach. The examination of individual contributions to the interaction energy calculated *via* the SSMF3 method is useful to select those parts of the calixarene molecule, which are of major importance for the interaction. By a difference analysis of two or more such fragments, more information can be obtained about the nature of the interaction, such as, e.g., about the cooperative or anti-cooperative interaction of the major binding site with neighboring functional groups or about a subtle dependence of the sign of such effects on the relative position of these groups.

A partition of the SAPT interaction energy into components and – simultaneously–into contributions from pairs of functional groups with help of the F-SAPT approach, gives a plethora of information about the strength and nature of interactions between these groups. Depending on the relative importance of the electrostatic, first-order exchange, and effective induction and dispersion contributions with respect to the total interaction energy of the pair, we propose a preliminary classification of the interactions into: (i) typical H-bonds; (ii) H-bonds with a dispersion flavor; (iii) H-bonds with an aryl ring as the hydrogen acceptor; (iv) dispersion-dominated, and finally; (v) electrostatic-dominated interactions, depending on ratios of SAPT components with respect to the total interaction energy between these two groups. The characteristic feature of H-bonds is a high ratio of first-order exchange, which is a prerequisite of the effective overlap of the electron clouds.

After pair interactions are classified according to their strength and origin, one can single out cases, for which two or more such interactions are of similar importance. Numerous such cases arise in this study because of multiple candidates for binding sites for both calixarenes and amino acids. Especially interesting among them are those inclusion complexes for which the confinement effect plays a role in stabilization.

Finally, smaller-than-expected stability of some complexes can be explained by the existence of pair interactions that are weakly bound or which repel each other because of an inadequate alignment of the interacting pair.

The picture of the binding between the studied calixarenes and the amino acids is not simple and unequivocal. Therefore, it was not possible to find a clear tendency in the studied set of complexes. However, the analysis of data reveals that irrespectively of the type of the amino acid its NH_2_ group and, to a lesser extent also the COOH group, participate in the calixarene-amino acid binding and that they can form H-bonds of various strengths and geometries, as described in more detail above. Further, in the case of His, a large contribution comes from the interaction of its imidazole ring. This ring can participate both in the H-bond formation through its nitrogen atoms, as well as in the π-π stacking interactions. The latter is also very well developed and plays a decisive role in the case of the binding of Phe. Surprisingly, the indole ring of Trp contributes less to the overall binding. The amino acids possessing the OH group (Ser, Thr, and Tyr) involve this entity to form relatively strong H-bonds with the calixarenes hosts. Finally, the contribution of the SH entity is also seen in the case of the Cys complexes.

This study shows that the I- and F-SAPT approaches are very useful to elucidate the nature of inter- and intramolecular noncovalent bindings. Additionally, they can be used to refine explanations or other phenomena, such as the lowering of the IR frequency for the *al*-CX, which–as revealed by the I-SAPT analysis–resulted from the noncovalent attractive interaction between hydroxy and phenyl groups of different calixarene units.

## Figures and Tables

**Table 1:** The relative electronic and total energy of both empty calixarenes in three considered conformations, and complexes of these conformations with amino acids with respect to the lowest conformation energy. Energies in millihartree.

	CX Electronic Energy			CX Total Energy			BCX Electronic Energy			BCX Total Energy		
**Amino Acid**	***al*** **-CX**	***pc*** **-CX**	***wc*** **-CX**	***al*** **-CX**	***pc*** **-CX**	***wc*** **-CX**	***al*** **-BCX**	***pc*** **-BCX**	***wc*** **-BCX**	***al*** **-BCX**	***pc*** **-BCX**	***wc*** **-BCX**
-	13.2	0.0	15.8	12.0	0.0	14.9	10.7	0.0	1.7	9.7	0.0	2.4
Ala	2.7	2.4	0.0	2.0	1.5	0.0	3.5	3.6	0.0	1.6	1.5	0.0
Asn	0.0	16.1	15.7	0.0	16.3	17.5	1.8	14.4	0.0	0.0	12.0	0.0
AspH	0.0	4.1	8.7	0.0	4.2	9.4	3.9	11.1	0.0	3.5	11.0	0.0
Cys	0.0	4.7	6.0	0.0	4.3	6.3	0.0	11.2	0.2	0.0	10.0	1.8
Gln	0.0	1.6	1.8	0.0	0.1	1.9	4.2	6.9	0.0	2.9	6.4	0.0
GluH	0.0	1.1	1.1	0.0	1.5	1.7	9.9	10.2	0.0	9.1	9.2	0.0
Gly	0.0	8.7	7.0	0.0	8.3	8.0	7.6	17.0	0.0	6.0	14.7	0.0
HisD	2.0	2.4	0.0	0.9	1.6	0.0	5.1	11.9	0.0	4.5	9.9	0.0
HisE	0.0	3.5	4.8	0.0	4.1	5.6	5.2	4.8	0.0	2.5	2.9	0.0
Ile	2.9	0.0	1.7	3.6	0.0	2.5	8.8	7.9	0.0	7.2	6.5	0.0
Leu	0.8	0.0	13.7	0.3	0.0	13.5	9.8	6.6	0.0	7.9	6.1	0.0
Lys	0.0	1.0	1.6	0.0	2.5	2.2	1.6	12.6	0.0	0.0	13.3	0.0
Met	0.0	5.9	4.8	0.0	5.1	4.6	2.4	1.1	0.0	0.4	0.0	0.2
Phe	1.9	0.0	8.6	2.5	0.0	8.8	1.6	0.1	0.0	1.1	0.0	0.3
Pro	2.1	0.0	9.5	0.7	0.0	9.4	4.5	0.0	3.8	4.2	0.0	4.8
Ser	0.0	11.8	9.4	0.0	9.8	9.8	2.1	13.5	0.0	0.0	10.4	0.0
Thr	0.0	7.6	11.2	0.0	5.9	11.2	4.4	11.0	0.0	3.1	8.1	0.0
Trp	0.1	0.0	3.7	0.8	0.0	4.5	13.7	8.2	0.0	10.8	6.7	0.0
Tyr	2.7	2.2	0.0	1.9	1.2	0.0	8.6	0.0	5.7	6.9	0.0	5.3
Val	7.9	0.0	8.4	7.3	0.0	8.8	7.4	5.4	0.0	5.6	3.9	0.0

**Table 2:** The SAPT0 energy components, SAPT0 and HF interaction energies, MP2 and SCS-MP2 electron-correlated parts of the interaction energies for CX + amino acid complexes. The percent errors of the SSMF3 fragmentation scheme are given in parentheses. Energies in millihartree.

	$E_{\mathrm{elst}}^{\left(10\right)}$	$E_{\mathrm{exch}}^{\left(10\right)}$	$E_{\mathrm{ind},\mathrm{resp}}^{\left(20\right)}$	$E_{\mathrm{exch}-\mathrm{ind},\mathrm{resp}}^{\left(20\right)}$	$E_{\mathrm{disp}}^{\left(20\right)}$	$E_{\mathrm{exch}-\mathrm{disp}}^{\left(20\right)}$	$E_{\mathrm{int}}^{\mathrm{HF}}$	$E_{\mathrm{tot}}^{\mathrm{SAPT}0}$	$E_{\mathrm{corr},\mathrm{int}}^{\mathrm{MP}2}$	$E_{\mathrm{corr},\mathrm{int}}^{\mathrm{SCS}-\mathrm{MP}2}$
*al*−CX-Ala	−66.5 (−0.3)	97.9 (0.6)	−46.2 (1.4)	29.4 (0.7)	−56.6 (−1.4)	8.3 (−2.9)	−0.1 (−423.9)	−48.4 (−1.9)	−40.7 (−1.0)	−29.6 (−1.1)
*al*−CX-Asn	−86.9 (0.4)	110.4 (0.1)	−58.9 (−1.1)	35.0 (−1.4)	−57.0 (−0.9)	8.5 (−1.3)	−20.3 (−3.8)	−68.8 (−1.7)	−37.9 (−1.4)	−26.8 (−1.7)
*al*−CX-AspH	−67.5 (−0.3)	94.5 (0.3)	−46.7 (−1.0)	29.9 (−1.9)	−55.3 (−1.1)	7.6 (−1.4)	−3.7 (−24.7)	−51.3 (−2.8)	−40.1 (−1.4)	−29.2 (−1.5)
*al*−CX-Cys	−84.5 (0.4)	104.7 (0.1)	−51.6 (−0.4)	31.4 (−1.1)	−58.8 (−1.5)	8.6 (−2.7)	−15.8 (−0.8)	−66.0 (−1.1)	−38.4 (−1.8)	−26.9 (−2.2)
*al*−CX-Gln	−102.6 (0.0)	128.1 (0.6)	−59.0 (−0.3)	36.0 (−0.5)	−68.9 (−0.9)	10.1 (−1.4)	−16.5 (−4.8)	−75.3 (−1.7)	−44.4 (−1.3)	−30.8 (−1.6)
*al*−CX-GluH	−59.4 (1.2)	66.9 (0.1)	−30.4 (0.5)	18.7 (0.0)	−54.0 (−1.8)	6.5 (−3.5)	−11.8 (7.1)	−59.2 (0.2)	−34.9 (−2.0)	−24.3 (−2.7)
*al*−CX-Gly	−74.7 (0.5)	91.3 (0.2)	−46.8 (−0.4)	28.2 (−1.2)	−50.6 (−1.6)	7.5 (−2.7)	−16.6 (−1.2)	−59.7 (−1.3)	−32.6 (−1.4)	−22.7 (−1.6)
*al*−CX-HisD	−57.1 (1.7)	67.4 (0.2)	−30.6 (2.7)	18.3 (0.3)	−54.6 (−1.4)	6.4 (−3.2)	−10.2 (18.7)	−58.4 (2.3)	−38.4 (−1.1)	−27.4 (−1.4)
*al*−CX-HisE	−67.6 (−0.1)	96.5 (0.4)	−49.0 (−1.4)	30.6 (−0.8)	−63.7 (−0.8)	8.7 (−0.8)	−5.2 (−26.7)	−60.1 (−3.1)	−45.9 (−1.3)	−33.8 (−1.4)
*al*−CX-Ile	−56.1 (−0.3)	76.0 (0.1)	−32.6 (0.9)	21.5 (−0.6)	−61.6 (−1.5)	7.6 (−3.3)	0.4 (−73.8)	−53.6 (−0.7)	−43.6 (−1.5)	−31.4 (−1.9)
*al*−CX-Leu	−58.3 (−0.1)	83.7 (0.3)	−39.0 (−1.1)	25.0 (−1.7)	−57.6 (−1.2)	7.5 (−2.1)	−0.3 (−267.8)	−50.4 (−2.7)	−43.1 (−1.5)	−31.9 (−1.6)
*al*−CX-Lys	−75.7 (0.2)	102.7 (0.4)	−48.3 (−0.6)	29.8 (−1.5)	−63.0 (−0.9)	8.7 (−2.0)	−7.9 (−11.3)	−62.2 (−2.1)	−44.7 (−1.1)	−32.5 (−1.3)
*al*−CX-Met	−58.3 (1.9)	74.6 (0.1)	−33.7 (1.5)	21.2 (−0.4)	−62.2 (−1.7)	7.6 (−3.5)	−4.9 (38.4)	−59.5 (1.8)	−43.7 (−1.1)	−31.5 (−1.4)
*al*−CX-Phe	−52.8 (−0.2)	68.5 (−0.1)	−29.5 (−0.4)	18.2 (−0.4)	−53.5 (−1.0)	6.4 (−2.0)	−4.5 (−11.2)	−51.6 (−1.8)	−36.4 (−1.5)	−26.0 (−1.7)
*al*−CX-Pro	−85.3 (−0.1)	115.5 (0.3)	−59.8 (−1.5)	36.2 (−1.1)	−58.3 (−1.1)	8.8 (−1.7)	−13.8 (−11.9)	−63.3 (−3.4)	−39.5 (−1.2)	−28.2 (−1.4)
*al*−CX-Ser	−61.7 (−0.4)	74.4 (0.4)	−31.1 (1.6)	19.8 (0.8)	−53.1 (−1.8)	6.8 (−4.3)	−6.2 (3.1)	−52.5 (−0.9)	−37.6 (−1.4)	−27.0 (−1.7)
*al*−CX-Thr	−62.4 (−0.2)	72.3 (0.2)	−29.8 (1.2)	18.9 (0.2)	−57.1 (−1.7)	7.1 (−3.6)	−8.3 (3.8)	−58.3 (−0.7)	−41.0 (−1.4)	−29.5 (−1.7)
*al*−CX-Trp	−53.4 (−0.3)	79.1 (0.3)	−34.7 (−0.4)	24.3 (−0.7)	−67.7 (−0.7)	8.3 (−1.4)	5.7 (5.4)	−53.6 (−1.2)	−53.0 (−0.9)	−39.6 (−1.0)
*al*−CX-Tyr	−60.8 (−0.1)	73.3 (0.4)	−31.7 (1.9)	20.1 (0.8)	−60.1 (−1.6)	7.4 (−3.5)	−6.9 (7.7)	−59.6 (−0.3)	−43.4 (−1.5)	−31.7 (−1.8)
*al*−CX-Val	−60.1 (−0.8)	78.8 (0.2)	−36.6 (−0.2)	22.9 (−1.7)	−57.7 (−1.2)	7.3 (−2.9)	−5.4 (−11.2)	−55.9 (−2.0)	−42.3 (−1.7)	−31.0 (−2.0)
*pc*−CX-Ala	−55.1 (−0.2)	62.7 (0.1)	−29.5 (2.9)	18.2 (0.0)	−30.1 (−1.4)	4.6 (−1.9)	−12.0 (2.8)	−37.6 (0.0)	−18.0 (−1.4)	−12.3 (−1.8)
*pc*−CX-Asn	−34.1 (1.2)	37.7 (0.0)	−14.4 (13.3)	10.1 (2.1)	−31.2 (−2.0)	3.4 (−2.6)	−3.3 (65.5)	−31.1 (5.3)	−20.3 (−2.0)	−14.4 (−2.6)
*pc*−CX-AspH	−26.6 (2.1)	32.8 (0.4)	−13.5 (13.9)	8.7 (2.7)	−26.9 (−1.4)	2.8 (−1.8)	−1.4 (147.3)	−25.6 (7.0)	−18.3 (−1.6)	−13.2 (−2.1)
*pc*−CX-Cys	−14.6 (2.7)	19.6 (0.3)	−7.0 (14.8)	5.0 (1.9)	−19.7 (−1.1)	1.8 (−1.0)	1.4 (−95.5)	−16.6 (6.7)	−14.4 (−1.1)	−10.5 (−1.3)
*pc*−CX-Gln	−50.1 (0.2)	57.3 (0.3)	−26.1 (6.5)	16.2 (1.3)	−35.4 (−1.5)	4.5 (−1.8)	−9.7 (15.8)	−40.6 (2.7)	−21.1 (−1.2)	−14.4 (−1.7)
*pc*−CX-GluH	−41.8 (0.6)	45.6 (0.2)	−20.7 (9.7)	13.3 (2.0)	−29.8 (−1.6)	3.6 (−2.1)	−8.5 (25.2)	−34.7 (5.0)	−17.8 (−2.0)	−12.3 (−2.6)
*pc*−CX-Gly	−33.4 (0.8)	36.0 (0.0)	−15.9 (6.8)	9.7 (0.9)	−20.1 (−1.4)	2.6 (−1.9)	−8.0 (15.3)	−25.5 (3.9)	−11.4 (−1.3)	−7.7 (−1.9)
*pc*−CX-HisD	−39.8 (0.6)	52.5 (0.1)	−22.6 (7.0)	16.3 (1.2)	−47.0 (−1.1)	5.4 (−1.1)	2.0 (−84.7)	−39.6 (3.2)	−34.1 (−0.9)	−25.1 (−1.1)
*pc*−CX-HisE	−24.9 (0.2)	36.2 (0.3)	−15.2 (7.0)	11.8 (1.6)	−39.4 (−0.6)	4.3 (−0.1)	5.0 (−18.9)	−30.1 (2.3)	−30.3 (−0.7)	−22.6 (−0.7)
*pc*−CX-Ile	−22.2 (2.4)	39.8 (0.8)	−14.7 (4.1)	11.4 (1.2)	−41.2 (−1.3)	4.4 (−1.8)	11.1 (−6.8)	−25.7 (1.1)	−32.1 (−1.6)	−24.1 (−1.8)
*pc*−CX-Leu	−31.0 (1.3)	43.8 (0.3)	−16.1 (10.4)	11.8 (1.7)	−38.5 (−1.0)	4.1 (−1.4)	4.9 (−40.4)	−29.5 (5.5)	−28.1 (−0.8)	−20.7 (−1.0)
*pc*−CX-Lys	−19.3 (0.3)	38.9 (0.2)	−12.4 (3.2)	9.4 (0.5)	−43.2 (−1.0)	4.5 (−1.4)	13.4 (−3.5)	−25.2 (0.4)	−35.4 (−0.9)	−26.7 (−1.0)
*pc*−CX-Met	−17.4 (2.1)	25.6 (0.5)	−8.6 (17.7)	6.0 (2.2)	−26.9 (−0.9)	2.3 (−0.2)	3.5 (−49.2)	−21.0 (7.1)	−20.1 (−1.6)	−14.8 (−1.9)
*pc*−CX-Phe	−39.9 (0.9)	56.2 (0.0)	−24.9 (5.8)	18.4 (0.9)	−49.1 (−1.0)	6.0 (−1.7)	4.6 (−36.2)	−38.5 (3.3)	−36.4 (−0.8)	−27.1 (−1.0)
*pc*−CX-Pro	−20.1 (0.0)	34.2 (0.2)	−12.8 (3.4)	9.6 (0.7)	−39.0 (−0.9)	4.1 (−0.9)	7.7 (−4.9)	−27.2 (0.3)	−30.8 (−1.1)	−22.9 (−1.2)
*pc*−CX-Ser	−30.0 (1.4)	34.7 (0.3)	−14.4 (11.5)	9.6 (2.0)	−24.8 (−1.5)	2.8 (−1.8)	−3.5 (57.0)	−25.6 (6.6)	−16.2 (−1.3)	−11.5 (−1.7)
*pc*−CX-Thr	−37.9 (0.7)	39.6 (−0.3)	−17.7 (4.3)	10.5 (0.2)	−22.5 (−1.3)	2.8 (−1.7)	−10.4 (11.0)	−30.1 (3.0)	−12.1 (−1.4)	−8.0 (−2.0)
*pc*−CX-Trp	−35.8 (1.0)	54.5 (0.0)	−22.2 (7.7)	16.9 (1.1)	−55.7 (−0.9)	6.2 (−1.1)	8.9 (−22.9)	−40.6 (3.9)	−42.7 (−0.7)	−32.1 (−0.8)
*pc*−CX-Tyr	−17.3 (1.8)	27.4 (0.1)	−10.8 (4.6)	7.0 (1.2)	−31.4 (−0.8)	3.1 (−0.7)	4.0 (−18.4)	−24.3 (2.1)	−24.3 (−0.5)	−17.9 (−0.5)
*pc*−CX-Val	−23.1 (0.7)	37.5 (0.5)	−13.0 (4.1)	9.6 (1.4)	−39.5 (−1.1)	4.2 (−1.3)	8.0 (−5.8)	−27.3 (0.3)	−30.4 (−1.1)	−22.6 (−1.3)
*wc*−CX-Ala	−72.9 (−0.1)	80.5 (0.1)	−39.3 (−1.3)	23.0 (−1.9)	−36.4 (−1.2)	5.7 (−3.0)	−20.9 (−0.4)	−51.7 (−0.7)	−21.3 (−1.3)	−14.2 (−1.6)
*wc*−CX-Asn	−92.1 (−0.1)	109.0 (0.2)	−55.3 (−0.6)	31.5 (−1.4)	−45.4 (−1.0)	7.1 (−2.5)	−23.8 (−1.0)	−62.1 (−0.8)	−25.6 (−1.3)	−16.9 (−1.8)
*wc*−CX-AspH	−49.7 (0.6)	48.1 (−0.3)	−22.4 (−0.8)	12.5 (−1.2)	−30.0 (−1.2)	3.6 (−2.3)	−17.6 (1.6)	−44.1 (0.0)	−16.7 (−2.0)	−10.8 (−2.8)
*wc*−CX-Cys	−54.9 (−0.3)	58.0 (−0.3)	−27.8 (−0.2)	16.0 (−0.7)	−30.9 (−1.3)	4.1 (−3.2)	−17.7 (−1.4)	−44.4 (−1.2)	−16.7 (−2.0)	−10.8 (−2.6)
*wc*−CX-Gln	−83.6 (−0.2)	85.2 (−0.4)	−41.1 (0.1)	22.2 (−1.0)	−39.3 (−1.3)	5.7 (−2.9)	−31.4 (0.7)	−65.0 (−0.2)	−18.1 (−2.0)	−10.8 (−2.9)
*wc*−CX-GluH	−71.5 (0.2)	73.2 (−0.4)	−37.1 (−0.9)	19.9 (−0.9)	−34.0 (−1.3)	4.8 (−3.1)	−28.0 (−0.7)	−57.2 (−0.9)	−16.0 (−2.6)	−9.6 (−3.7)
*wc*−CX-Gly	−74.2 (0.0)	80.0 (0.2)	−39.5 (−1.2)	23.0 (−1.8)	−34.7 (−1.3)	5.6 (−3.3)	−22.9 (−0.4)	−52.0 (−0.7)	−19.8 (−1.5)	−13.0 (−1.9)
*wc*−CX-HisD	−89.0 (0.2)	104.4 (0.1)	−52.6 (−0.5)	31.5 (−1.2)	−52.9 (−1.0)	7.9 (−2.4)	−22.0 (0.0)	−67.1 (−0.5)	−34.9 (−1.1)	−24.6 (−1.3)
*wc*−CX-HisE	−71.0 (0.1)	83.3 (0.1)	−40.8 (−0.6)	25.9 (−1.2)	−51.2 (−1.0)	7.1 (−2.0)	−13.7 (1.4)	−57.7 (−0.3)	−36.3 (−0.7)	−26.4 (−0.8)
*wc*−CX-Ile	−68.1 (0.0)	75.6 (0.0)	−35.0 (−1.0)	20.5 (−1.8)	−39.4 (−1.1)	5.5 (−2.7)	−17.7 (0.7)	−51.6 (−0.3)	−24.6 (−1.2)	−16.8 (−1.5)
*wc*−CX-Leu	−73.8 (0.1)	86.4 (−0.1)	−42.5 (−0.2)	25.2 (−1.1)	−43.6 (−1.2)	6.4 (−2.2)	−17.0 (2.1)	−54.2 (0.0)	−26.3 (−1.4)	−17.7 (−1.7)
*wc*−CX-Lys	−92.1 (0.2)	105.1 (−0.3)	−55.9 (−0.6)	32.2 (−0.9)	−44.8 (−1.4)	7.1 (−2.9)	−29.3 (0.1)	−66.9 (−0.5)	−24.7 (−2.1)	−16.1 (−2.8)
*wc*−CX-Met	−77.5 (−0.1)	90.1 (0.1)	−43.9 (−0.8)	25.6 (−1.9)	−42.4 (−1.1)	6.3 (−2.8)	−19.7 (−0.1)	−55.8 (−0.6)	−25.7 (−1.1)	−17.4 (−1.4)
*wc*−CX-Phe	−78.8 (0.1)	88.2 (0.1)	−43.6 (−1.2)	25.3 (−2.1)	−41.3 (−1.2)	6.2 (−3.1)	−22.4 (0.5)	−57.4 (−0.4)	−24.4 (−1.3)	−16.4 (−1.7)
*wc*−CX-Pro	−70.7 (0.9)	81.1 (−0.1)	−39.8 (−0.1)	24.7 (−0.6)	−46.7 (−1.6)	6.6 (−3.1)	−15.4 (6.1)	−55.5 (0.7)	−29.0 (−1.6)	−20.0 (−2.0)
*wc*−CX-Ser	−81.1 (0.0)	84.1 (−0.1)	−41.5 (−0.4)	23.3 (−1.9)	−39.4 (−1.0)	5.9 (−2.8)	−27.3 (1.4)	−60.8 (0.3)	−22.0 (−1.3)	−14.2 (−1.7)
*wc*−CX-Thr	−77.1 (0.3)	85.9 (0.0)	−40.0 (−0.2)	23.7 (−1.9)	−43.5 (−1.3)	6.3 (−3.0)	−19.3 (3.8)	−56.5 (0.6)	−25.8 (−1.7)	−17.1 (−2.2)
*wc*−CX-Trp	−79.9 (0.2)	93.8 (−0.1)	−43.3 (0.1)	27.4 (−1.6)	−60.4 (−1.1)	8.0 (−2.6)	−13.9 (5.8)	−66.3 (0.5)	−40.8 (−1.3)	−28.9 (−1.6)
*wc*−CX-Tyr	−72.2 (0.1)	83.8 (−0.1)	−40.2 (−0.7)	26.7 (−1.3)	−53.7 (−0.7)	7.5 (−1.7)	−12.7 (1.5)	−58.9 (−0.1)	−37.1 (−0.7)	−26.6 (−0.8)
*wc*−CX-Val	−71.5 (0.2)	78.8 (−0.1)	−37.3 (−1.1)	21.8 (−2.0)	−40.3 (−1.1)	5.7 (−2.8)	−19.4 (1.2)	−54.0 (−0.1)	−24.6 (−1.3)	−16.7 (−1.6)

**Table 3:** I-SAPT interaction energies between hydroxy groups for empty calixarenes. The upper triangle presents the BCX case, the bottom triangle–the CX case, energy values for the *al*, *pc*, and *wc* conformers, respectively, are separated by a dash. Energies in millihartree.

	OH-1	OH-2	OH-3	OH-4	OH-5	OH-6
OH-1	-	−10.9/−12.3/−9.3	−0.5/−1.6/0.0	−0.4/−1.0/0.0	−0.7/−3.2/−0.3	−11.4/−15.1/−9.7
OH-2	−11.8/−12.6/−9.6	-	−0.1/−14.3/−1.2	−0.2/−3.2/−0.3	0.4 /2.1/0.0	−4.0 /−5.7/−2.7
OH-3	−0.3/−1.5/−0.3	−0.1/−13.9/−1.1	-	−11.6/−15.1/−9.6	−2.9/−5.7/−2.7	−0.3 /−1.5 /1.2
OH-4	−0.4/−0.9/0.0	−0.6/−3.2/0.1	−11.2/−15.2/−9.2	-	−10.5/−12.3/−9.3	−0.3/−1.6 /0.0
OH-5	−0.3/−3.2/0.1	−0.4/ 2.2/1.4	−4.0/−5.9/−2.7	−11.7/−12.6/−9.6	-	−1.8/−14.3/−1.2
OH-6	−11.2/−15.3/−9.2	−3.0/−5.9/−2.7	0.4 /−1.2/0.0	−0.8/−1.5/−0.3	−1.8/−13.8/−1.1	-

**Table 4:** The total SAPT0 interaction energies of unfragmented and the SSMF3 fragments for all CX conformers + Gly complex are presented. Energies are in millihartree.

	Weight	*al*-CX	*pc*-CX	*wc*-CX
No Fragmentation		−59.7	−25.5	−52.0
Fragment #1	1	−25.3	−8.7	−9.5
Fragment #2	−1	−18.1	−1.7	−6.1
Fragment #3	1	−22.6	−4.8	−24.3
Fragment #4	−1	−22.6	−4.7	−24.1
Fragment #5	1	−22.7	−4.7	−24.2
Fragment #6	−1	−4.3	−2.7	−16.3
Fragment #7	1	−9.6	−18.7	−22.5
Fragment #8	−1	−6.9	−18.5	−23.1
Fragment #9	1	−7.3	−18.6	−23.0
Fragment #10	−1	−6.5	−13.3	−3.5
Fragment #11	1	−25.5	−9.7	−10.0
Fragment #12	−1	−25.0	−9.4	−9.8
Fragment #13	1	−26.4	−9.7	−9.5
Fragment #14	−1	−19.4	2.5	−5.4
Fragment #15	1	−21.8	2.7	−16.1
Fragment #16	−1	−21.1	3.1	−16.0
Fragment #17	1	−21.2	1.9	−16.2
Fragment #18	−1	−3.6	−0.9	−10.8
Fragment #19	1	−7.8	−8.6	−14.4
Fragment #20	−1	−7.2	−8.7	−14.5
Fragment #21	1	−7.4	−8.7	−14.5
Fragment #22	−1	−4.0	−7.8	−2.9
Fragment #23	1	−24.5	−9.4	−10.9
Fragment #24	−1	−24.6	−8.6	−10.8
Sum		−58.9	−26.5	−51.6

**Table 5:** The F-SAPT partitioning for selected pairs. Energies are in millihartree. The numbers in parenthesis are ratios with respect to total interaction energies between pairs, as defined in Section 3.9.4.

Complex	Group A	Group B	$E_{\mathrm{elst}}$ (Ratio)	$E_{\mathrm{exch}}$ (Ratio)	$E_{\mathrm{ind},\mathrm{eff}}$ (Ratio)	$E_{\mathrm{disp},\mathrm{eff}}$ (Ratio)	$E_{\mathrm{total}}$	Bond Type
*al*-CX-Asn	OH-2	NH_2_-1	−53.9 (1.8)	60.7 (2.0)	−29.0 (1.0)	−7.5 (0.3)	−29.7	typical H-bond
*al*-BCX-Lys	OH-2	NH_2_-1	−48.3 (1.6)	47.9 (1.6)	−23.1 (0.8)	−6.5 (0.2)	−29.9	typical H-bond
*al*-CX-Pro	OH-2	Ring	−44.7 (1.7)	54.0 (2.1)	−26.2 (1.0)	−8.7 (0.3)	−25.5	typical H-bond+disp
*wc*-CX-Pro	OH-3	Ring	−26.8 (1.8)	29.1 (2.0)	−11.2 (0.8)	−5.8 (0.4)	−14.6	typical H-bond+disp
*wc*-BCX-Pro	OH-6	Ring	−25.7 (1.8)	28.4 (2.0)	−11.0 (0.8)	−5.6 (0.4)	−13.9	typical H-bond+disp
*wc*-CX-Leu	Ph-6	COOH	−19.7 (0.9)	7.5 (0.3)	−4.7 (0.2)	−4.8 (0.2)	−21.7	H⋯π
*wc*-CX-Pro	Ph-6	COOH	−16.6 (0.9)	6.2 (0.3)	−3.8 (0.2)	−4.5 (0.2)	−18.6	H⋯π
*al*-BCX-Ser	Ph-5	COOH	−19.4 (0.9)	10.2 (0.5)	−5.8 (0.3)	−5.8 (0.3)	−20.8	H⋯π
*pc*-BCX-Tyr	Ph-4	OH	−10.2 (1.0)	5.5 (0.6)	−2.5 (0.3)	−2.8 (0.3)	−9.9	H⋯π
*pc*-CX-HisE	Ph-6	Ring	−11.9 (0.9)	10.1 (0.8)	−2.7 (0.2)	−8.5 (0.7)	−13.0	disp
*wc*-CX-HisE	Ph-2	Ring	−11.8 (0.9)	7.5 (0.6)	−1.7 (0.1)	−7.2 (0.5)	−13.2	disp
*pc*-BCX-HisE	Ph-5	Ring	−13.4 (0.9)	10.6 (0.7)	−3.5 (0.2)	−8.8 (0.6)	−15.1	disp
*wc*-BCX-HisE	Ph-5	Ring	−13.9 (0.9)	10.0 (0.7)	−2.3 (0.2)	−9.0 (0.6)	−15.1	disp
*wc*-BCX-Trp	Ph-5	Ring	−12.1 (0.8)	10.7 (0.7)	−2.6 (0.2)	−10.8 (0.7)	−14.8	disp
*pc*-CX-Tyr	Ph-3	COOH	−15.7 (0.9)	1.2 (0.1)	−1.4 (0.1)	−2.2 (0.1)	−18.1	elst
*al*-CX-HisD	Ph-4	COOH	−11.7 (0.8)	0.3 (0.0)	−2.3 (0.2)	−1.5 (0.1)	−15.1	elst
*al*-BCX-Gly	Ph-5	COOH	−7.7 (0.7)	0.8 (0.1)	−1.4 (0.1)	−2.1 (0.2)	−10.5	elst
*pc*-BCX-GluH	Ph-3	COOH-1	−15.4 (0.8)	2.3 (0.1)	−2.2 (0.1)	−3.2 (0.2)	−18.5	elst

## Data Availability

Data is contained within the article or supplementary material.

## Supplementary Materials

The following are available at https://www.mdpi.com/article/10.3390/molecules27227938/s1, Figure S1: Amino acids as guest molecules used in this study, Figures S2–S9: IR spectra of calixarenes, Figures S10–105: The F-SAPT partitioning graphs, Tables S1 and S2: Dihedral angles for complexes of calixarenes, Table S3: I-SAPT interaction energies al-CX, Tables S4–S9: A comparison of the SSMF3 fragmentation scheme with the unfragmented results for supermolecular and SAPT0 interaction energies for amino acid and calixarene complex, Table S10: The SAPT0 energy components, SAPT0 and HF interaction energies, MP2 and SCS-MP2 electron-correlated parts of the interaction energies for BCX+amino acid complexes, Table S11: The electronic and total energy of both empty calixarenes in three considered conformations, and complexes of these conformations with amino acids, Tables S12–S17: Percent errors of the SSMF3 fragmentation scheme in respect to unfragmented results for amino acid+calixarene complex, Tables S18–S23: A complete list of frequencies and intensities of calixarenes, Tables S24–S26: The total SAPT0 interaction energies of unfragmented and the SSMF3 fragments for BCX+Gly complex, Table S27: The accuracy of the SSMF3-HF(b) and SSMF3-MP2(b) electric dipole moments and dipole static polarizabilities for three conformers of calix[6]arene, Table S28: The F-SAPT partitioning for selected CX+amino acid pairs with their classification, Table S29: The F-SAPT partitioning for selected BCX+amino acid pairs with their classification, Table S30: Interaction of hydroxy groups of the BCX calixarene with and without the presence of Gly as calculated with the I-SAPT method. FSAPT_b-sub.ods, F-SAPT partitioning for complexes with calix[6]arene; FSAPT_h-sub.ods, F-SAPT partitioning for complexes with hexa-*p*-*tert*-butylcalix[6]arene; xyz_files, Cartesian coordinates of studied systems.

## Abbreviations

The following abbreviations are used in this manuscript:

al	1,2,3-alternate
BCX	hexa-*p*-*tert*-butylcalix[6]arene
CBS	Complete Basis Set
CCSD(T)	coupled cluster singles and doubles with perturbative triples correction
CSD	Cambridge Structural Database
CX	calix[6]arene
DF	density fitting
DFT	Density-Functional Theory
DFT+D	Density-Functional Theory with dispersion correction
FF	Finite-field
F-SAPT	Functional-group SAPT
I-SAPT	Intramolecular SAPT
HF	Hartree-Fock
MP2	Møller-Plesset theory to the second order
pc	pinched-cone
SAPT	Symmetry-Adapted Perturbation Theory
SMFA	Systematic Molecular Fragmentation by Annihilation
SSMF	Symmetrized Systematic Molecular Fragmentation
SCS-MP2	spin-component-scaled MP2
wc	winged-cone
ZPVE	zero-point vibrational energy
