# Supplementary material for: Association Complexes of Calix[6]arenes with Amino Acids Explained by Energy-Partitioning Methods
Source: Molecules. 2022 Nov 16;27(22):7938. doi: 10.3390/molecules27227938 (PMC9699162; doi:10.3390/molecules27227938)
Supplement: Supplementary file 1 [file molecules-27-07938-s001.zip › SI_calixarene.pdf]

Association Complexes of Calix[6]arenes with Amino Acids Explained by Energy-Partitioning Methods – Supplementary Information

Emran Masoumifeshani,[*,†,‡] Michał Chojecki,[*,†,¶] Dorota Rutkowska-Zbik,[*,§,‖] and Tatiana Korona[*,†,⊥]

†*Faculty of Chemistry, University of Warsaw, ul. Pasteura 1, 02-093 Warsaw, Poland*

‡*ORCID:0000-0002-5872-3557*

¶*ORCID: 0000-0002-4590-5845*

§*Jerzy Haber Institute of Catalysis and Surface Chemistry, Polish Academy of Sciences, ul. Niezapominajek 8, 30-239 Cracow, Poland*

‖*ORCID: 0000-0001-9323-1710*

⊥*ORCID: 0000-0001-7169-3412*

E-mail: emran@chem.uw.edu.pl; mic1.054571726ael@gmail.com; dorota.rutkowska-zbik@ikifp.edu.pl; tania@chem.uw.edu.pl

List of Figures

S1 Amino acids as guest molecules used in this study 10
S2 IR spectra for pristine CX complexes 13
S3 IR spectra for pristine BCX complexes 14
S4 IR spectra for the *pc*-CX and its complexes with amino acids. 15
S5 IR spectra for the *al*-CX and its complexes with amino acids. 18
S6 IR spectra for the *wc*-CX and its complexes with amino acids. 21
S7 IR spectra for the *pc*-BCX and its complexes with amino acids. 24
S8 IR spectra for the *al*-BCX and its complexes with amino acids. 27
S9 IR spectra for the *wc*-BCX and its complexes with amino acids. 30
S10 The F-SAPT partitioning for *al*-CX alanine. 33
S11 The F-SAPT partitioning for *al*-CX asparagine 34
S12 The F-SAPT partitioning for *al*-CX aspartate 35
S13 The F-SAPT partitioning for *al*-CX cysteine 36
S14 The F-SAPT partitioning for *al*-CX glycine 37
S15 The F-SAPT partitioning for *al*-CX histidineD 38
S16 The F-SAPT partitioning for *al*-CX histidineE 39
S17 The F-SAPT partitioning for *al*-CX isoleucine 40
S18 The F-SAPT partitioning for *al*-CX leucine 41
S19 The F-SAPT partitioning for *al*-CX phenylalanine 42
S20 The F-SAPT partitioning for *al*-CX proline 43
S21 The F-SAPT partitioning for *al*-CX serine 44
S22 The F-SAPT partitioning for *al*-CX threonine 45
S23 The F-SAPT partitioning for *al*-CX tryptophan 46
S24 The F-SAPT partitioning for *al*-CX tyrosine 47
S25 The F-SAPT partitioning for *al*-CX valine 48
S26 The F-SAPT partitioning for *pc*-CX alanine 49
S27 The F-SAPT partitioning for *pc*-CX asparagine 50

S28 The F-SAPT partitioning for *pc*-CX aspartate 51
S29 The F-SAPT partitioning for *pc*-CX cysteine 52
S30 The F-SAPT partitioning for *pc*-CX glycine 53
S31 The F-SAPT partitioning for *pc*-CX histidineD 54
S32 The F-SAPT partitioning for *pc*-CX histidineE 55
S33 The F-SAPT partitioning for *pc*-CX isoleucine 56
S34 The F-SAPT partitioning for *pc*-CX leucine 57
S35 The F-SAPT partitioning for *pc*-CX phenylalanine 58
S36 The F-SAPT partitioning for *pc*-CX proline 59
S37 The F-SAPT partitioning for *pc*-CX serine 60
S38 The F-SAPT partitioning for *pc*-CX threonine 61
S39 The F-SAPT partitioning for *pc*-CX tryptophan 62
S40 The F-SAPT partitioning for *pc*-CX tyrosine 63
S41 The F-SAPT partitioning for *pc*-CX valine 64
S42 The F-SAPT partitioning for *wc*-CX alanine 65
S43 The F-SAPT partitioning for *wc*-CX asparagine 66
S44 The F-SAPT partitioning for *wc*-CX aspartate 67
S45 The F-SAPT partitioning for *wc*-CX cysteine 68
S46 The F-SAPT partitioning for *wc*-CX glycine 69
S47 The F-SAPT partitioning for *wc*-CX histidineD 70
S48 The F-SAPT partitioning for *wc*-CX histidineE 71
S49 The F-SAPT partitioning for *wc*-CX isoleucine 72
S50 The F-SAPT partitioning for *wc*-CX leucine 73
S51 The F-SAPT partitioning for *wc*-CX phenylalanine 74
S52 The F-SAPT partitioning for *wc*-CX proline 75
S53 The F-SAPT partitioning for *wc*-CX serine 76
S54 The F-SAPT partitioning for *wc*-CX threonine 77
S55 The F-SAPT partitioning for *wc*-CX tryptophan 78

S56 The F-SAPT partitioning for *wc*-CX tyrosine 79
S57 The F-SAPT partitioning for *wc*-CX valine 80
S58 The F-SAPT partitioning for *al*-BCX alanine 81
S59 The F-SAPT partitioning for *al*-BCX asparagine 82
S60 The F-SAPT partitioning for *al*-BCX aspartate 83
S61 The F-SAPT partitioning for *al*-BCX cysteine 84
S62 The F-SAPT partitioning for *al*-BCX glycine 85
S63 The F-SAPT partitioning for *al*-BCX histidineD 86
S64 The F-SAPT partitioning for *al*-BCX histidineE 87
S65 The F-SAPT partitioning for *al*-BCX isoleucine 88
S66 The F-SAPT partitioning for *al*-BCX leucine 89
S67 The F-SAPT partitioning for *al*-BCX phenylalanine 90
S68 The F-SAPT partitioning for *al*-BCX proline 91
S69 The F-SAPT partitioning for *al*-BCX serine 92
S70 The F-SAPT partitioning for *al*-BCX threonine 93
S71 The F-SAPT partitioning for *al*-BCX tryptophan 94
S72 The F-SAPT partitioning for *al*-BCX tyrosine 95
S73 The F-SAPT partitioning for *al*-BCX valine 96
S74 The F-SAPT partitioning for *pc*-BCX alanine 97
S75 The F-SAPT partitioning for *pc*-BCX asparagine 98
S76 The F-SAPT partitioning for *pc*-BCX aspartate 99
S77 The F-SAPT partitioning for *pc*-BCX cysteine 100
S78 The F-SAPT partitioning for *pc*-BCX glycine 101
S79 The F-SAPT partitioning for *pc*-BCX histidineD 102
S80 The F-SAPT partitioning for *pc*-BCX histidineE 103
S81 The F-SAPT partitioning for *pc*-BCX isoleucine 104
S82 The F-SAPT partitioning for *pc*-BCX leucine 105
S83 The F-SAPT partitioning for *pc*-BCX phenylalanine 106

S84 The F-SAPT partitioning for *pc*-BCX proline 107
S85 The F-SAPT partitioning for *pc*-BCX serine 108
S86 The F-SAPT partitioning for *pc*-BCX threonine 109
S87 The F-SAPT partitioning for *pc*-BCX tryptophan 110
S88 The F-SAPT partitioning for *pc*-BCX tyrosine 111
S89 The F-SAPT partitioning for *pc*-BCX valine 112
S90 The F-SAPT partitioning for *wc*-BCX alanine 113
S91 The F-SAPT partitioning for *wc*-BCX asparagine 114
S92 The F-SAPT partitioning for *wc*-BCX aspartate 115
S93 The F-SAPT partitioning for *wc*-BCX cysteine 116
S94 The F-SAPT partitioning for *wc*-BCX glycine 117
S95 The F-SAPT partitioning for *wc*-BCX histidineD 118
S96 The F-SAPT partitioning for *wc*-BCX histidineE 119
S97 The F-SAPT partitioning for *wc*-BCX isoleucine 120
S98 The F-SAPT partitioning for *wc*-BCX leucine 121
S99 The F-SAPT partitioning for *wc*-BCX phenylalanine 122
S100 The F-SAPT partitioning for *wc*-BCX proline 123
S101 The F-SAPT partitioning for *wc*-BCX serine 124
S102 The F-SAPT partitioning for *wc*-BCX threonine 125
S103 The F-SAPT partitioning for *wc*-BCX tryptophan 126
S104 The F-SAPT partitioning for *wc*-BCX tyrosine 127
S105 The F-SAPT partitioning for *wc*-BCX valine 128

List of Tables

S1 Dihedral angles for the amino acid + CX complexes under study. The structures of amino acid + CX were optimized using the def2-TZVP basis set at the DFT level with the B97-D3 functional. 129

S2 Dihedral angles for the amino acid + BCX complexes under study. The structures of amino acid + BCX were optimized using the def2-TZVP basis set at the DFT level with the B97-D3 functional. 130

S3 I-SAPT interaction energies (in mH) of *al*-CX. 131

S4 A comparison of the SSMF3 fragmentation scheme with the unfragmented results for supermolecular and SAPT0 interaction energies for amino acid + *al*-CX complex. HF, MP2, and SCS-MP2 interaction energies, as well as SAPT0 components and total SAPT0 interaction energies, are presented. Energies are in millihartree 132

S5 A comparison of the SSMF3 fragmentation scheme with the unfragmented results for supermolecular and SAPT0 interaction energies for amino acid + *pc*-CX complex. HF, MP2, and SCS-MP2 interaction energies, as well as SAPT0 components and total SAPT0 interaction energies, are presented. Energies are in millihartree 133

S6 A comparison of the SSMF3 fragmentation scheme with the unfragmented results for supermolecular and SAPT0 interaction energies for amino acid + *wc*-CX complex. HF, MP2, and SCS-MP2 interaction energies, as well as SAPT0 components and total SAPT0 interaction energies, are presented. Energies are in millihartree 134

S7 A comparison of the SSMF3 fragmentation scheme with the unfragmented results for supermolecular and SAPT0 interaction energies for amino acid + *al*-BCX complex. HF, MP2, and SCS-MP2 interaction energies, as well as SAPT0 components and total SAPT0 interaction energies, are presented. Energies are in millihartree 135

S8 A comparison of the SSMF3 fragmentation scheme with the unfragmented results for supermolecular and SAPT0 interaction energies for amino acid + *pc*-BCX complex. HF, MP2, and SCS-MP2 interaction energies, as well as SAPT0 components and total SAPT0 interaction energies, are presented. Energies are in millihartree 136

S9 A comparison of the SSMF3 fragmentation scheme with the unfragmented results for supermolecular and SAPT0 interaction energies for amino acid + *wc*-BCX complex. HF, MP2, and SCS-MP2 interaction energies, as well as SAPT0 components and total SAPT0 interaction energies, are presented. Energies are in millihartree 137

S10 The SAPT0 energy components, SAPT0 and HF interaction energies, MP2 and SCS-MP2 electron-correlated parts of the interaction energies (in mH) for BCX + amino acid complexes. The percent errors of the SSMF3 fragmentation scheme are given in parenthesis. 138

S11 The electronic and total energy (in Hartree) of both empty calixarenes in three considered conformations, and complexes of these conformations with amino acids. 139

S12 Percent errors of the SSMF3 fragmentation scheme in respect to unfragmented results for amino acid + *al*-CX complex. HF, electron-correlated part of MP2, and SCS-MP2 interaction energies, as well as SAPT0 components and total SAPT0 interaction energies, are presented. Energies are in millihartree. 140

S13 Percent errors of the SSMF3 fragmentation scheme in respect to unfragmented results for amino acid + *pc*-CX complex. HF, electron-correlated part of MP2, and SCS-MP2 interaction energies, as well as SAPT0 components and total SAPT0 interaction energies, are presented. Energies are in millihartree. 141

S14 Percent errors of the SSMF3 fragmentation scheme in respect to unfragmented results for amino acid + *wc*-CX complex. HF, electron-correlated part of MP2, and SCS-MP2 interaction energies, as well as SAPT0 components and total SAPT0 interaction energies, are presented. Energies are in millihartree. 142

S15 Percent errors of the SSMF3 fragmentation scheme in respect to unfragmented results for amino acid + *al*-BCX complex. HF, electron-correlated part of MP2, and SCS-MP2 interaction energies, as well as SAPT0 components and total SAPT0 interaction energies, are presented. Energies are in millihartree. 143

S16 Percent errors of the SSMF3 fragmentation scheme in respect to unfragmented results for amino acid + *pc*-BCX complex. HF, electron-correlated part of MP2, and SCS-MP2 interaction energies, as well as SAPT0 components and total SAPT0 interaction energies, are presented. Energies are in millihartree. 144

S17 Percent errors of the SSMF3 fragmentation scheme in respect to unfragmented results for amino acid + *wc*-BCX complex. HF, electron-correlated part of MP2, and SCS-MP2 interaction energies, as well as SAPT0 components and total SAPT0 interaction energies, are presented. Energies are in millihartree. 145

S18 A complete list of frequencies and intensities of *al*-CX 146

S19 A complete list of frequencies and intensities of *pc*-CX 147

S20 A complete list of frequencies and intensities of *wc*-CX 148

S21 A complete list of frequencies and intensities of *al*-BCX 149

S22 A complete list of frequencies and intensities of *pc*-BCX 150

S23 A complete list of frequencies and intensities of *wc*-BCX 151

S24 The total SAPT0 interaction energies of unfragmented and the SSMF3 fragments for *al*-BCX conformer + Gly complex are presented. Energies are in millihartree. 152

S25 The total SAPT0 interaction energies of unfragmented and the SSMF3 fragments for *pc*-BCX conformer + Gly complex are presented. Energies are in millihartree. 153

S26 The total SAPT0 interaction energies of unfragmented and the SSMF3 fragments for *wc*-BCX conformer + Gly complex are presented. Energies are in millihartree. 154

S27 The accuracy of the SSMF3-HF(b) and SSMF3-MP2(b) electric dipole moments and dipole static polarizabilities for three conformers of calix[6]arene. "(b)" denotes that only the bonded part of the property has been used in calculations. The same orientation as presented the Supplementary information has been used to define axes. The cc-pVDZ basis has been used. All data in a.u. 155

S28 The F-SAPT partitioning for selected CX+ amino acid pairs. Energies are in millihartree. 156

S29 The F-SAPT partitioning for selected BCX+ amino acid pairs with their classification. Energies are in millihartree. 157

S30 Interaction of hydroxy groups of the BCX calixarene with and without the presence of Gly as calculated with the I-SAPT method. Energies are in millihartree. 158

Figure S1: Amino acids as guest molecules used in this study

(1) alanine

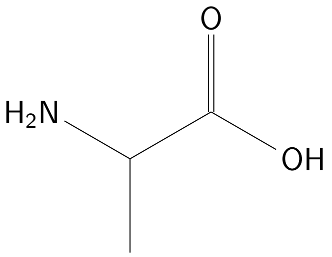

(2) asparagine

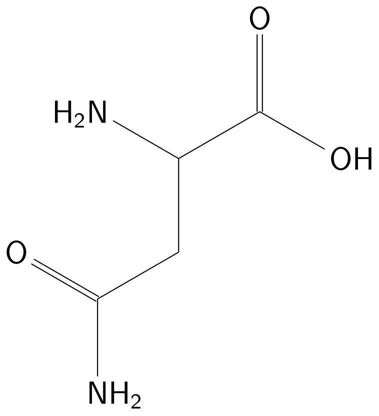

(3) aspartate

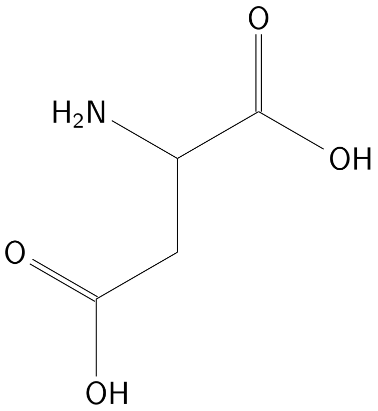

(4) cysteine

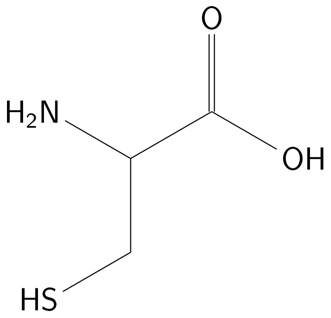

(5) glutamine

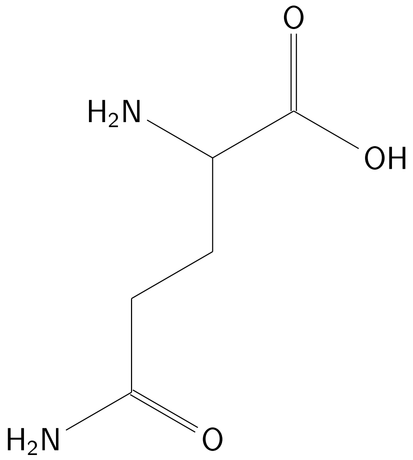

(6) glutamate

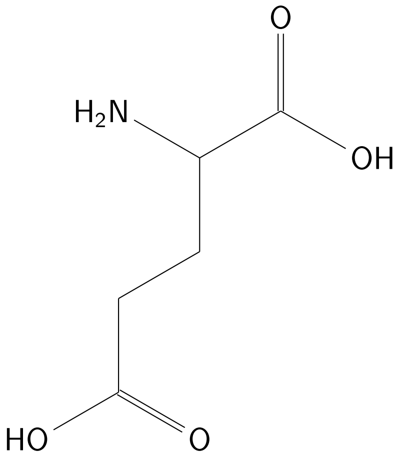

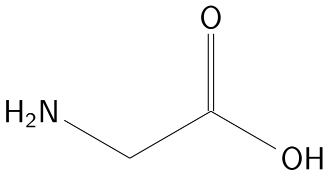

(7) glycine

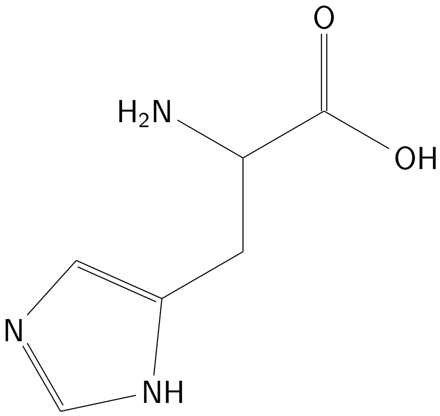

(8) histidine-D

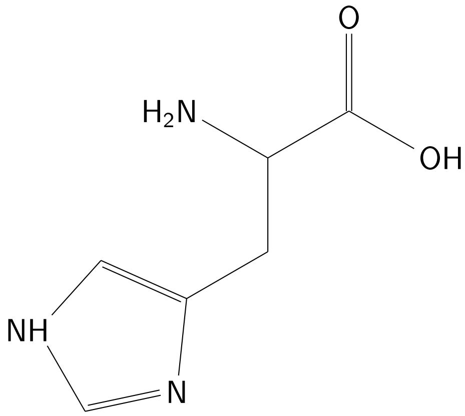

(9) histidine-E

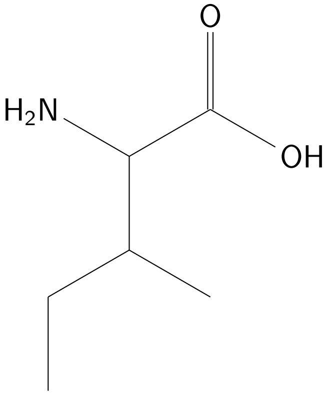

(10) isoleucine

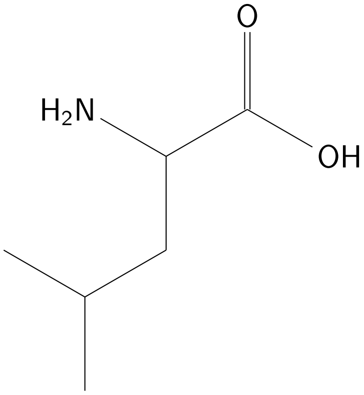

(11) leucine

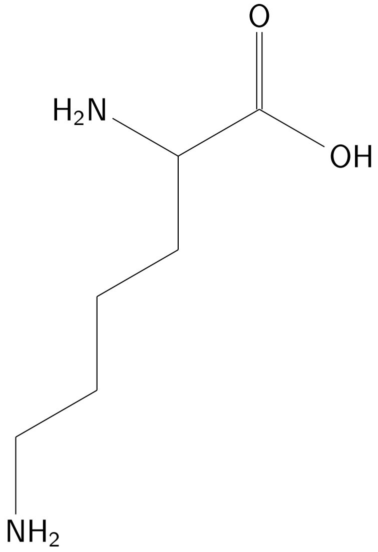

(12) lysine

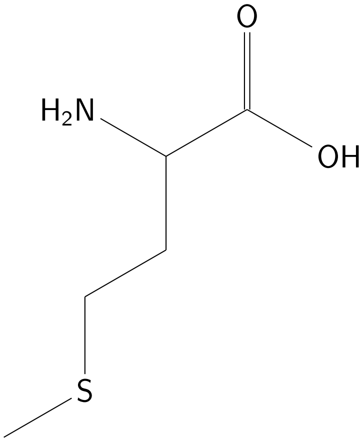

(13) methionine

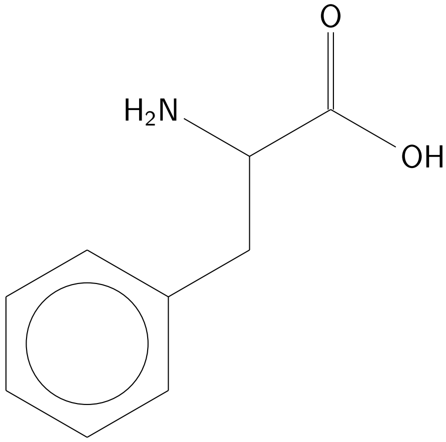

(14) phenylalanine

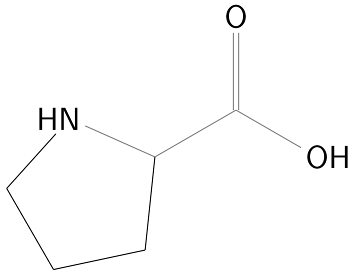

(15) proline

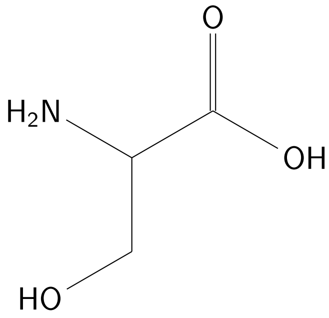

(16) serine

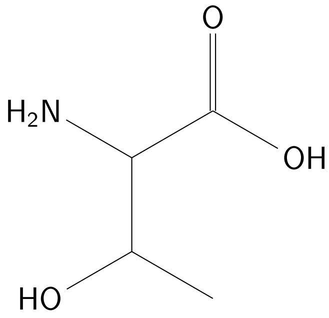

(17) threonine

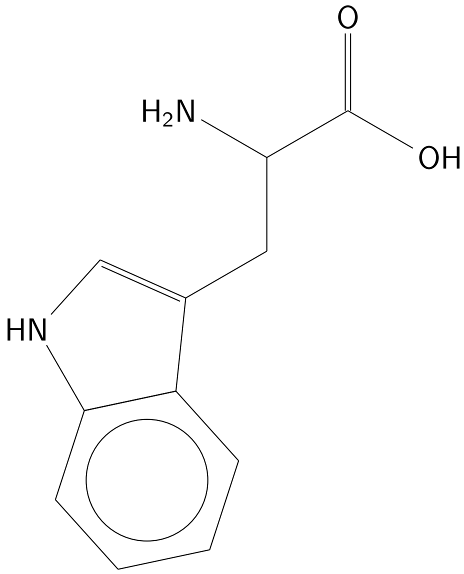

(18) tryptophan

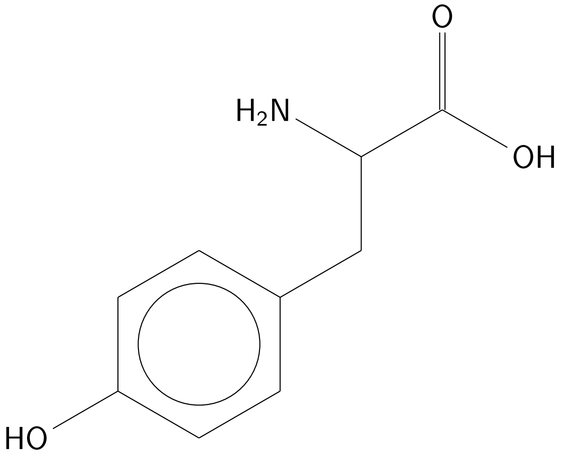

(19) tyrosine

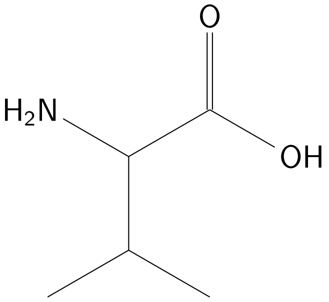

(20) valine

Figure S2: IR spectra for pristine CX complexes

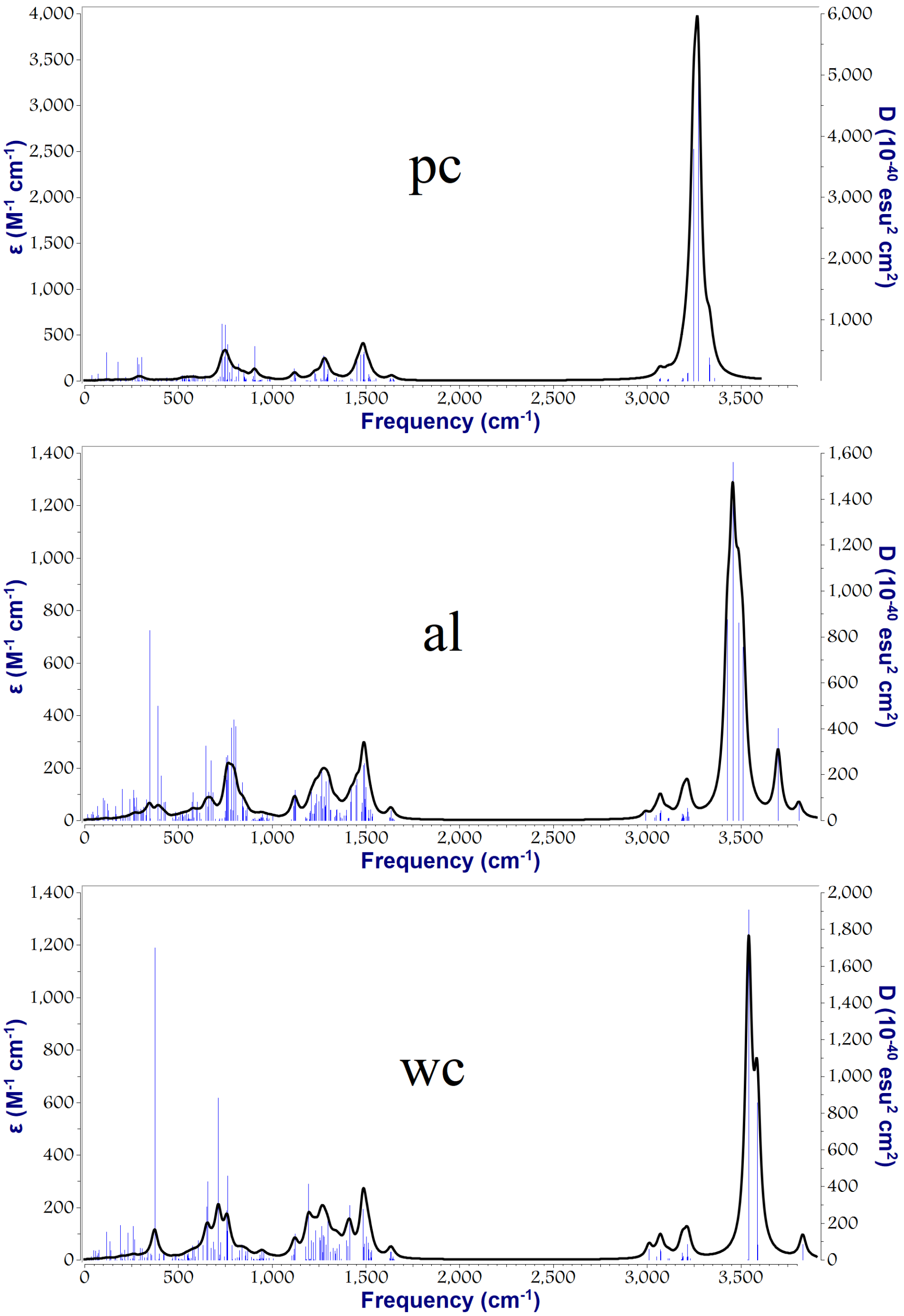

Figure S3: IR spectra for pristine BCX complexes

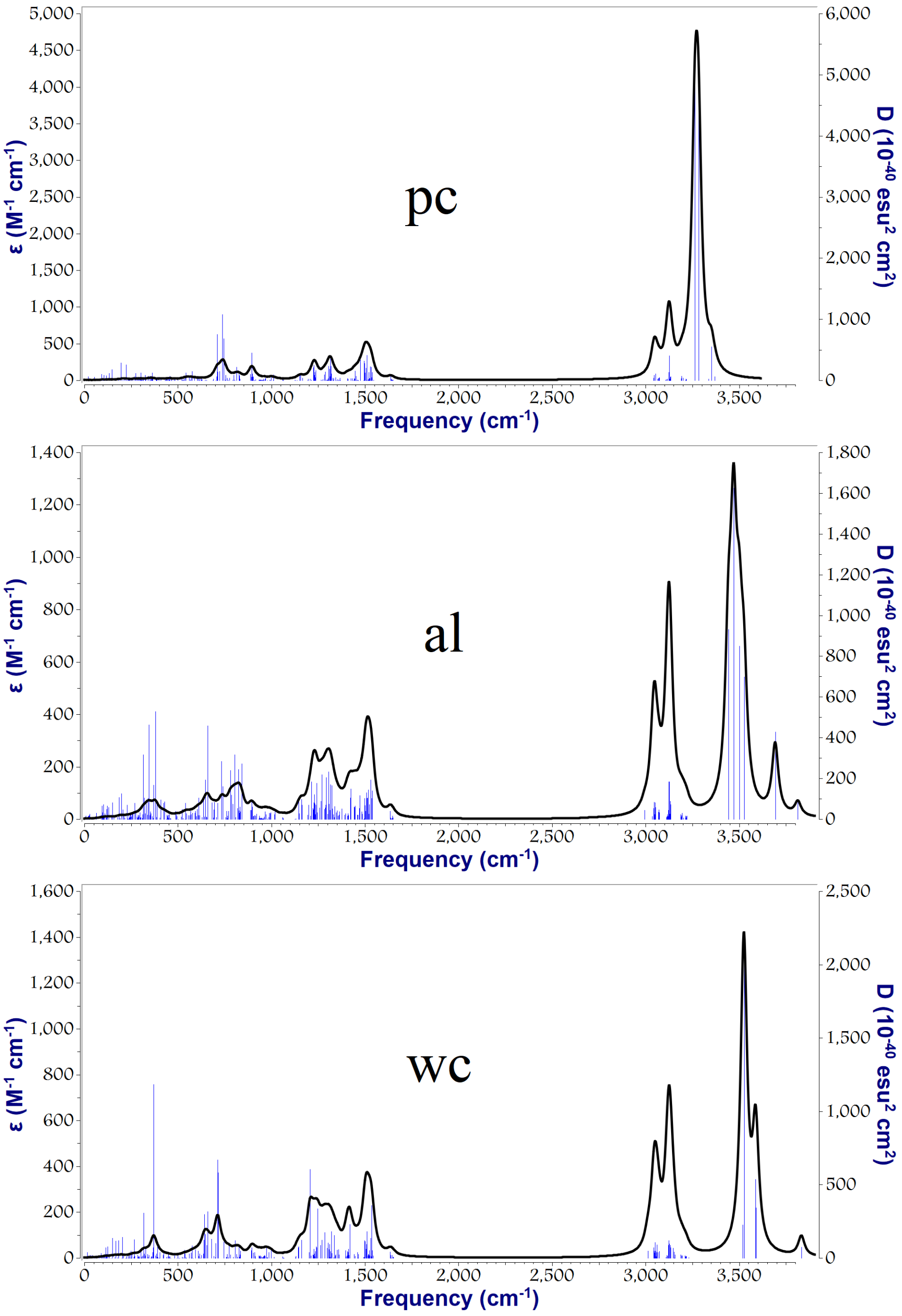

Figure S4: IR spectra for the *pc*-CX and its complexes with amino acids.

(1) pristine *pc*-CX

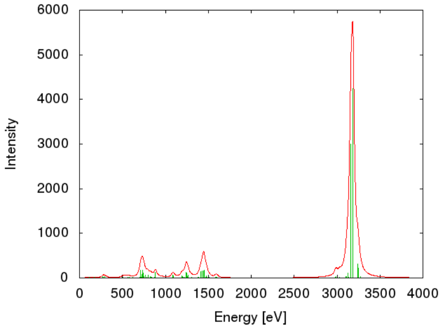

(2) alanine *pc*-CX

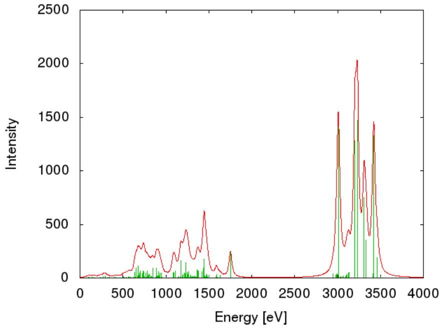

(3) asparagine *pc*-CX

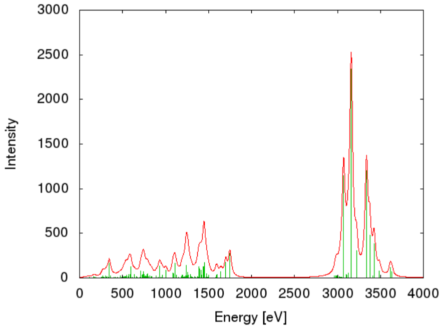

(4) aspartate *pc*-CX

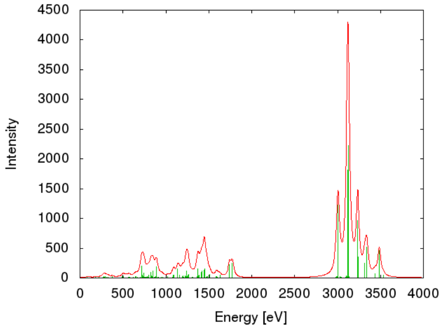

(5) cysteine *pc*-CX

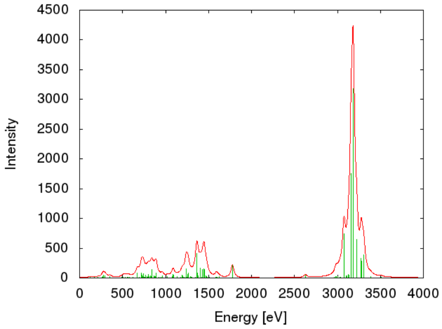

(6) glutamine *pc*-CX

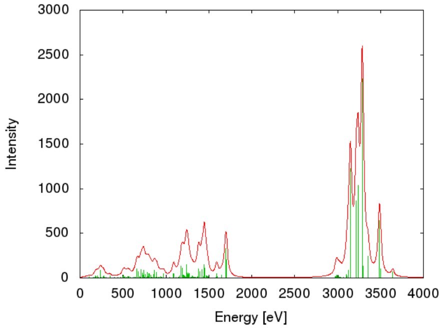

(7) glutamate *pc*-CX

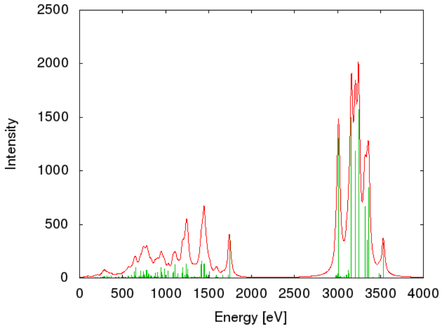

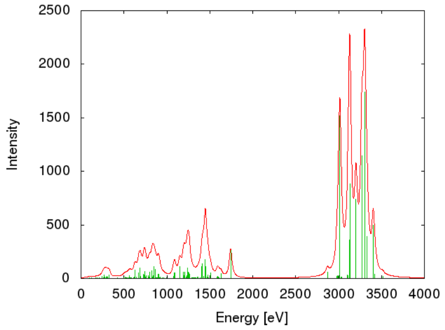

(8) glycine *pc*-CX

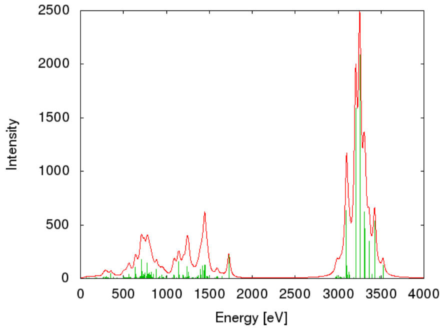

(9) histidine-D *pc*-CX

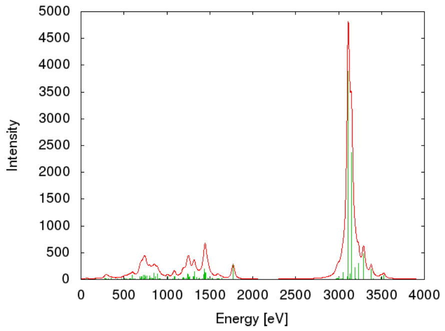

(10) histidine-E *pc*-CX

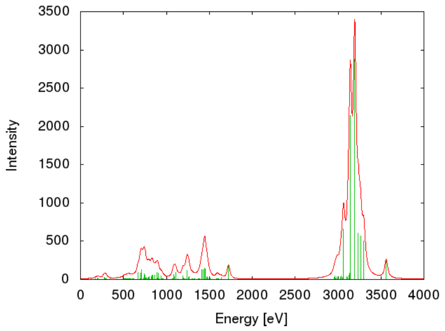

(11) isoleucine *pc*-CX

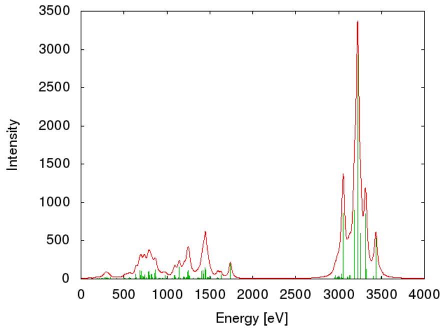

(12) leucine *pc*-CX

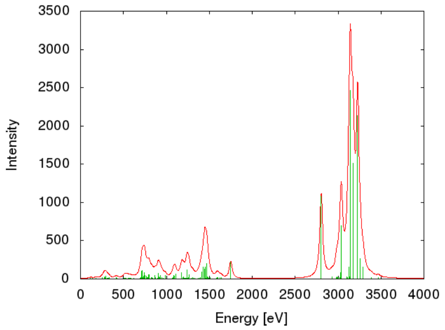

(13) lysine *pc*-CX

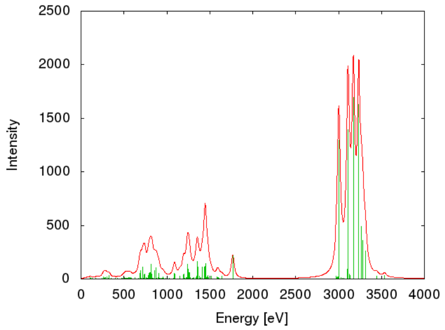

(14) methionine *pc*-CX

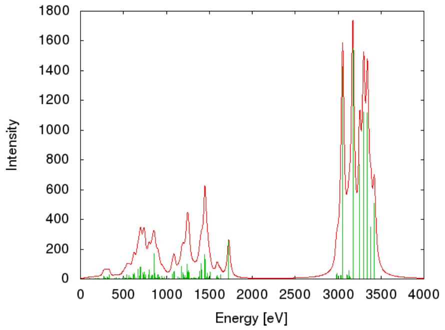

(15) phenylalanine *pc*-CX

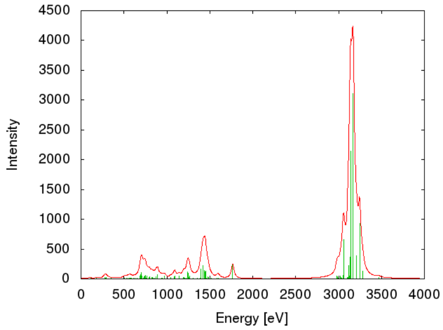

(16) proline *pc*-CX

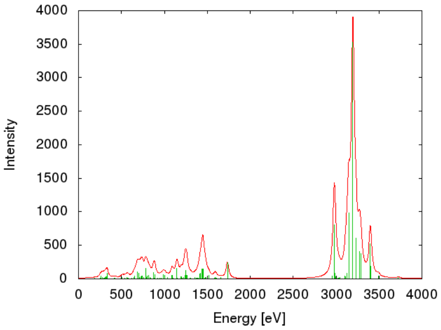

(17) serine *pc*-CX

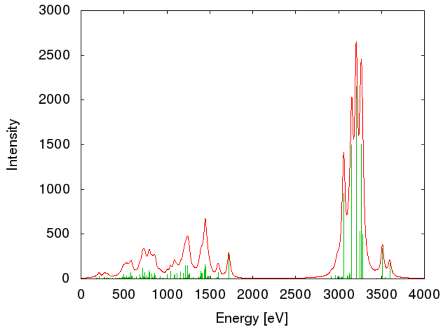

(18) threonine *pc*-CX

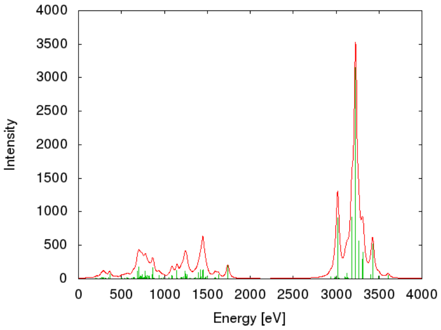

(19) tryptophan *pc*-CX

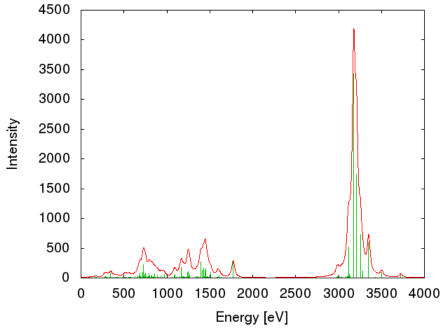

(20) tyrosine *pc*-CX

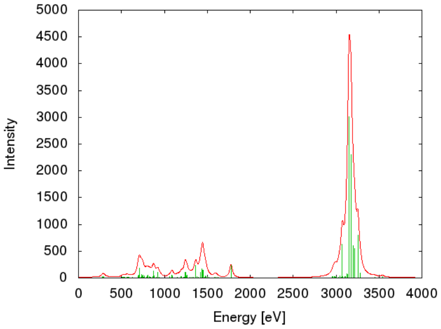

(21) valine *pc*-CX

Figure S5: IR spectra for the *al*-CX and its complexes with amino acids.

(1) pristine *al*-CX

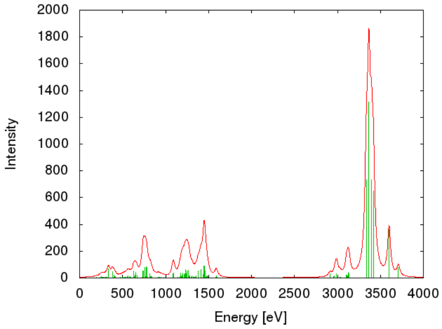

(2) alanine *al*-CX

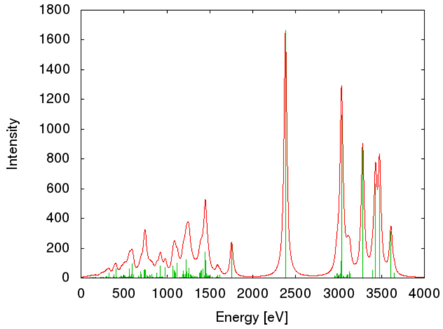

(3) asparagine *al*-CX

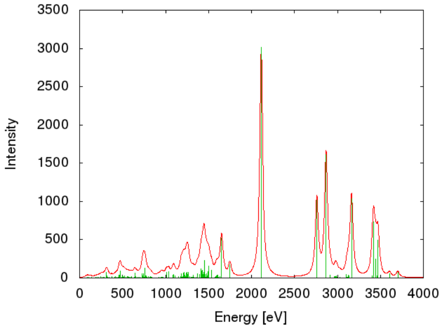

(4) aspartate *al*-CX

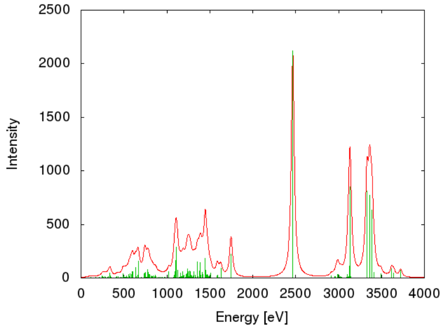

(5) cysteine *al*-CX

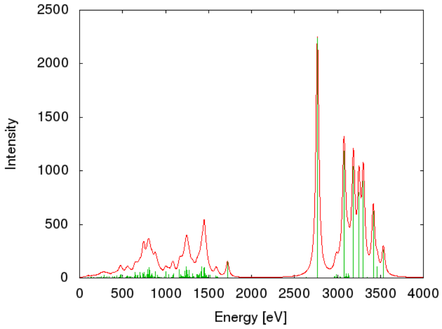

(6) glutamine *al*-CX

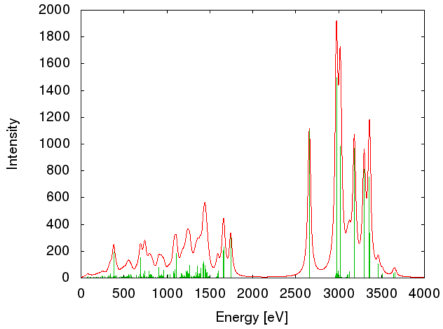

(7) glutamate *al*-CX

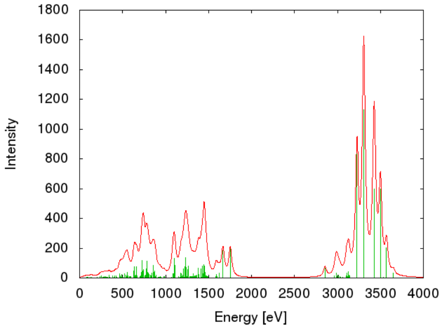

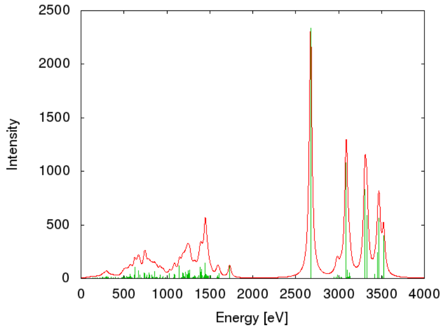

(8) glycine *al*-CX

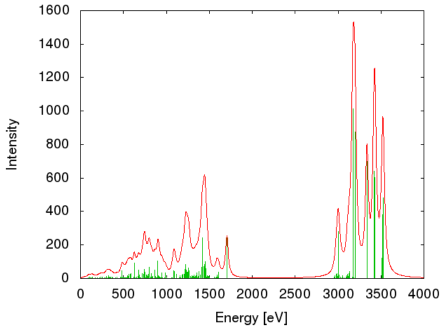

(9) histidine-D *al*-CX

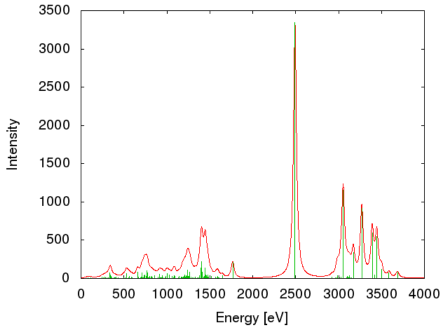

(10) histidine-E *al*-CX

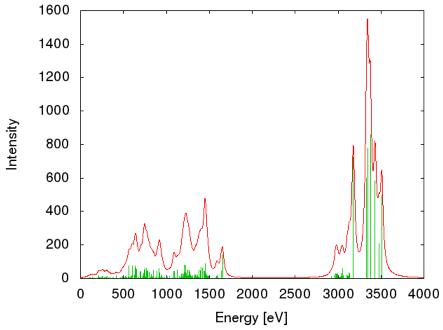

(11) isoleucine *al*-CX

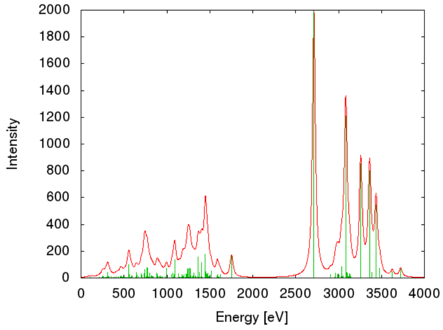

(12) leucine *al*-CX

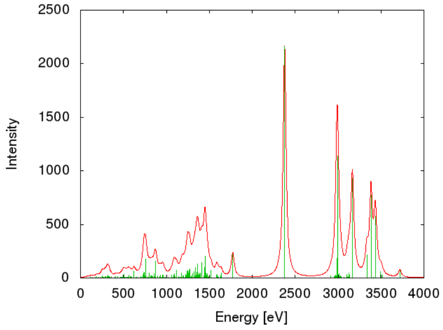

(13) lysine *al*-CX

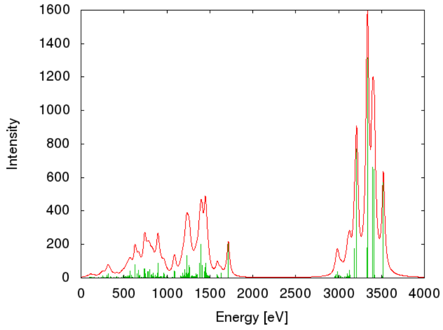

(14) methionine *al*-CX

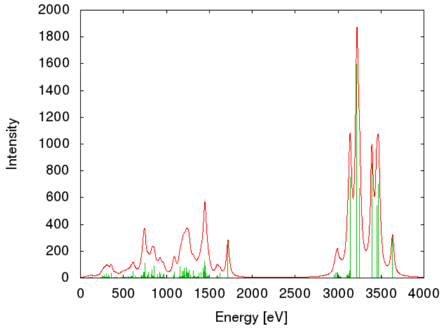

(15) phenylalanine *al*-CX

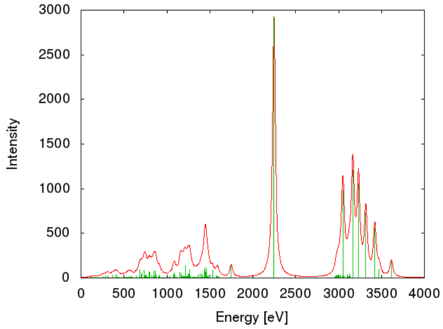

(16) proline *al*-CX

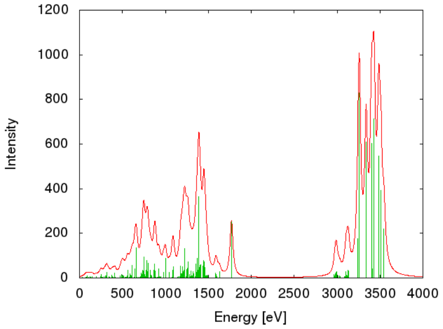

(17) serine *al*-CX

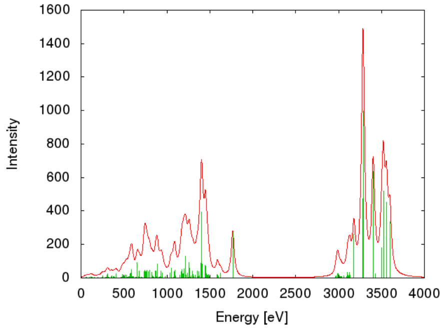

(18) threonine *al*-CX

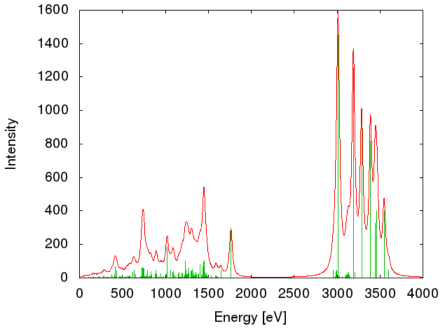

(19) tryptophan *al*-CX

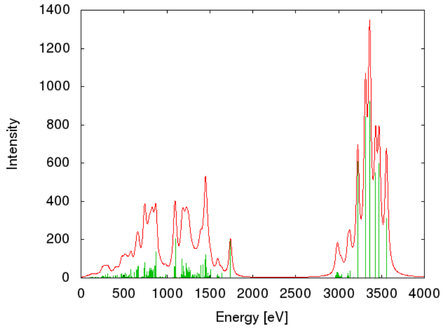

(20) tyrosine *al*-CX

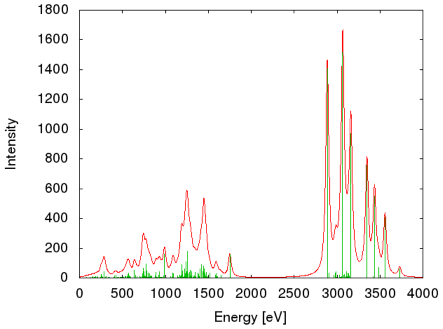

(21) valine *al*-CX

Figure S6: IR spectra for the *wc*-CX and its complexes with amino acids.

(1) pristine *wc*-CX

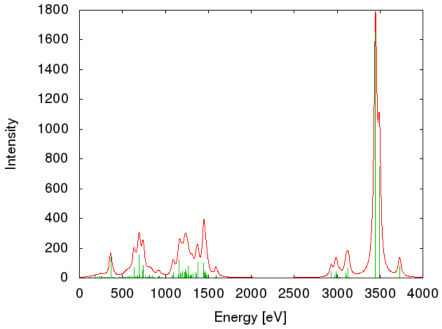

(2) alanine *wc*-CX

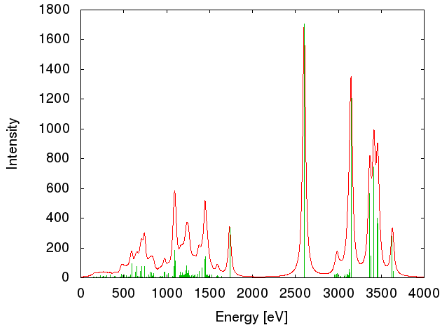

(3) asparagine *wc*-CX

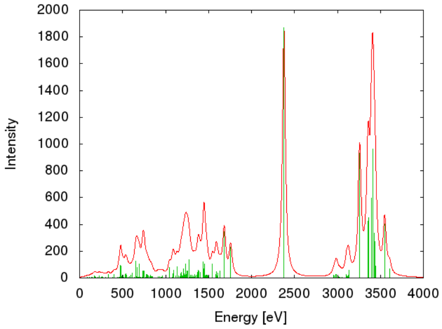

(4) aspartate *wc*-CX

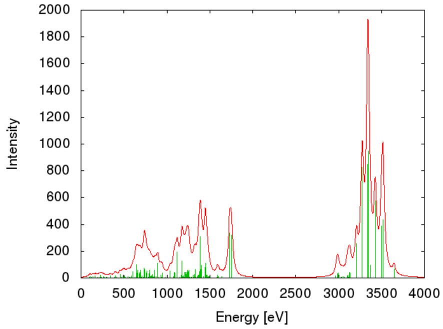

(5) cysteine *wc*-CX

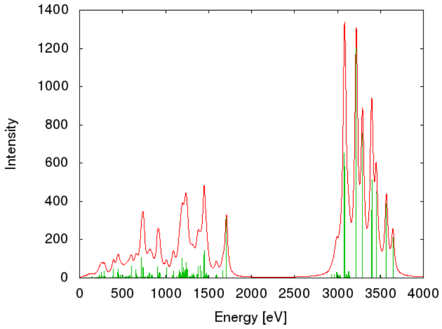

(6) glutamine *wc*-CX

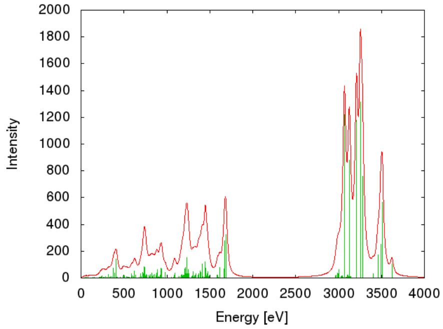

(7) glutamate *wc*-CX

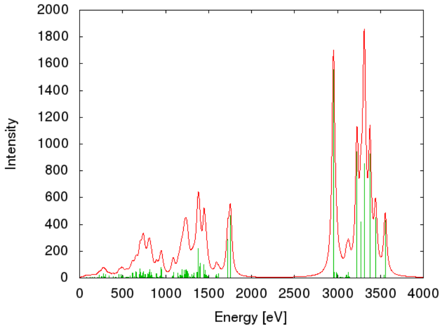

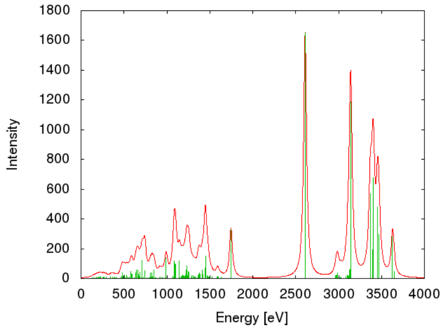

(8) glycine *wc*-CX

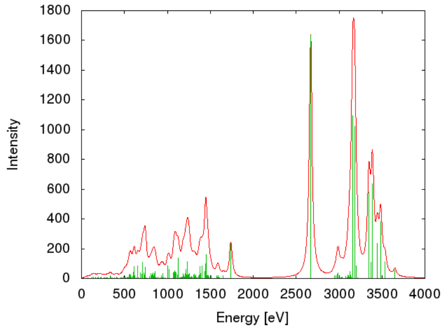

(9) histidine-D *wc*-CX

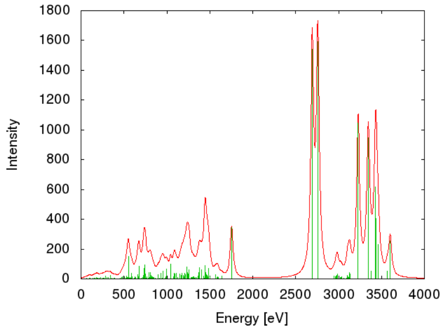

(10) histidine-E *wc*-CX

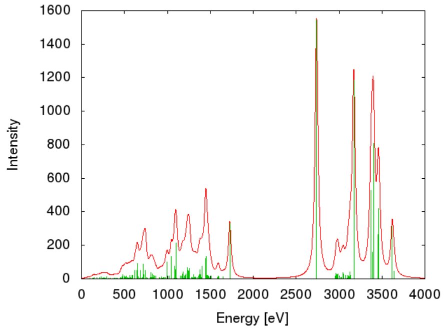

(11) isoleucine *wc*-CX

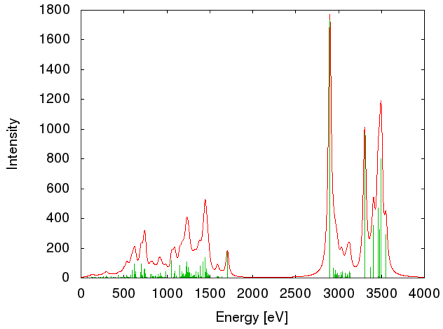

(12) leucine *wc*-CX

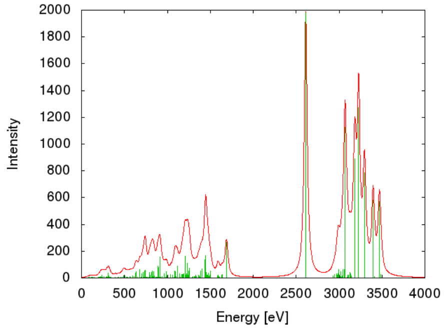

(13) lysine *wc*-CX

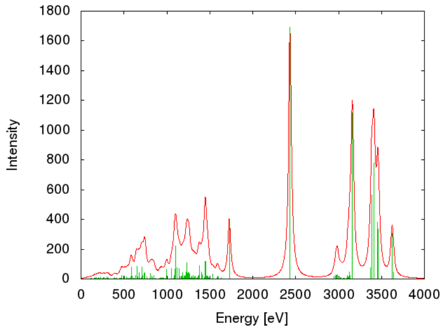

(14) methionine *wc*-CX

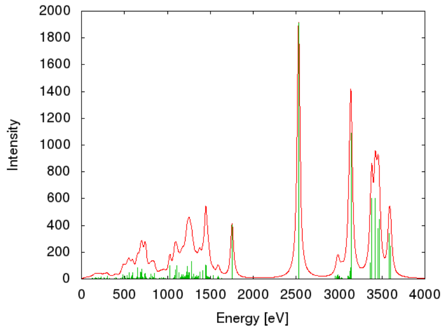

(15) phenylalanine *wc*-CX

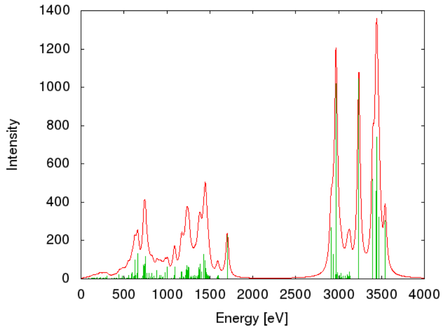

(16) proline *wc*-CX

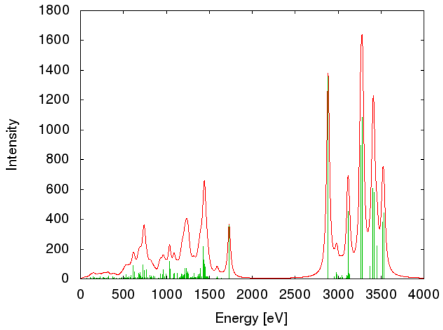

(17) serine *wc*-CX

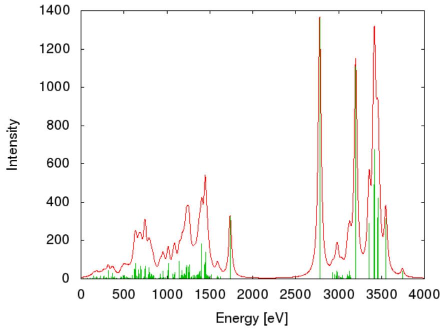

(18) threonine *wc*-CX

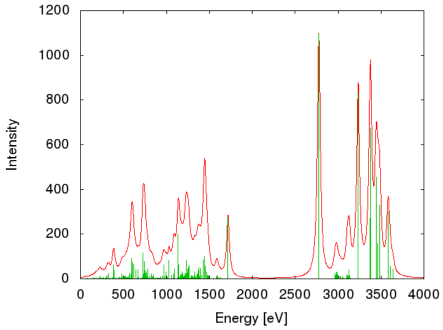

(19) tryptophan *wc*-CX

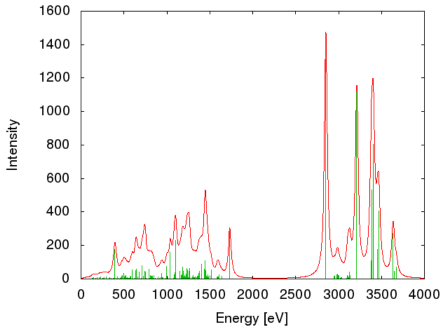

(20) tyrosine *wc*-CX

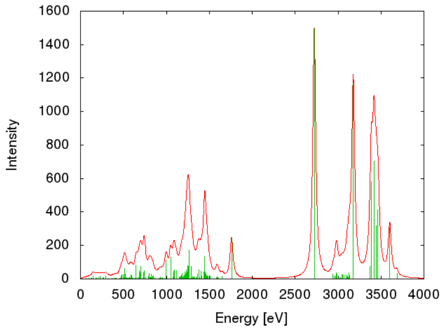

(21) valine *wc*-CX

Figure S7: IR spectra for the *pc*-BCX and its complexes with amino acids.

(1) pristine *pc*-BCX

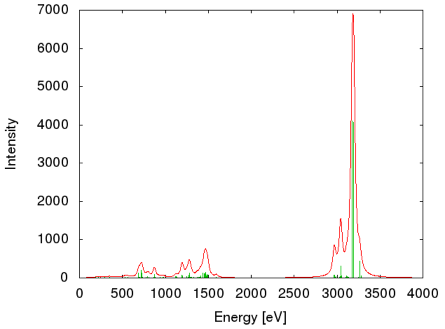

(2) alanine *pc*-BCX

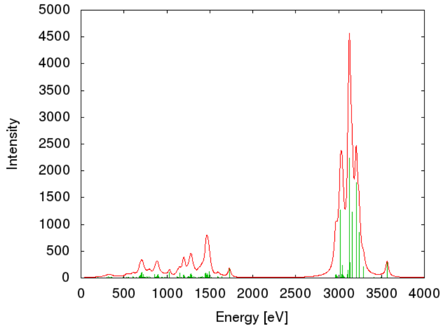

(3) asparagine *pc*-BCX

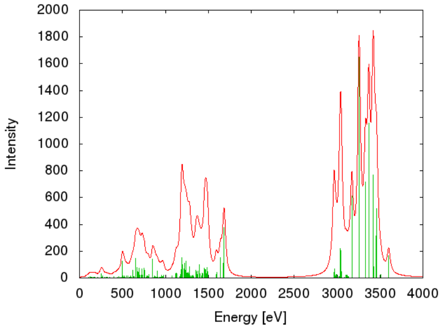

(4) aspartate *pc*-BCX

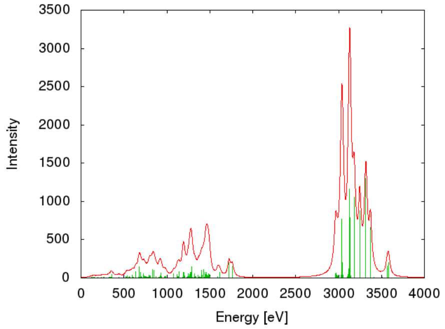

(5) cysteine *pc*-BCX

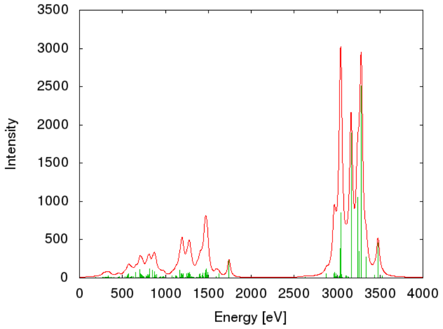

(6) glutamine *pc*-BCX

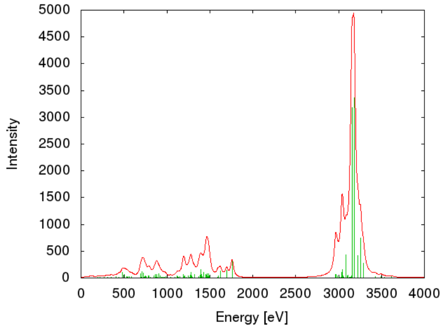

(7) glutamate *pc*-BCX

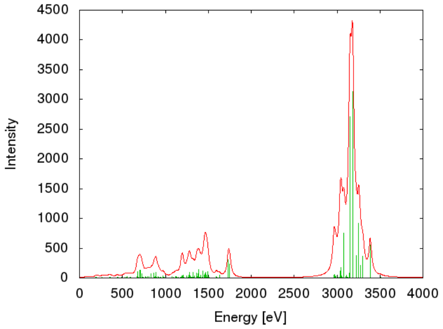

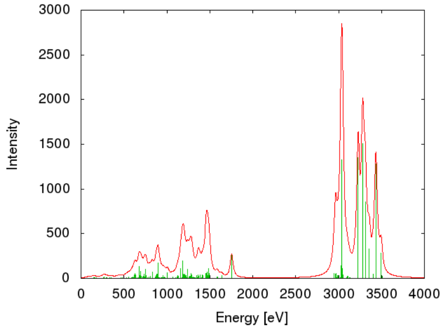

(8) glycine *pc*-BCX

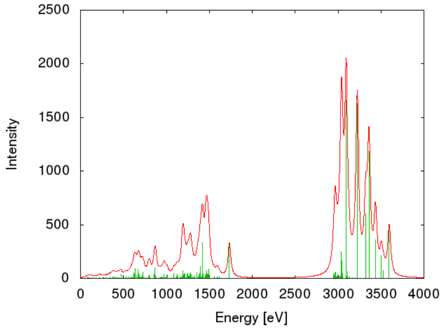

(9) histidine-D *pc*-BCX

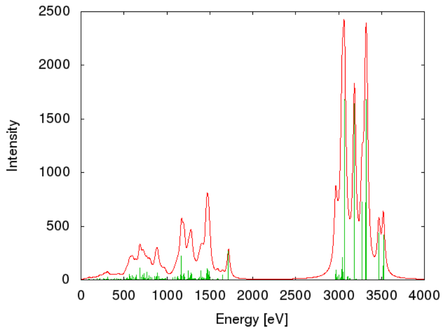

(10) histidine-E *pc*-BCX

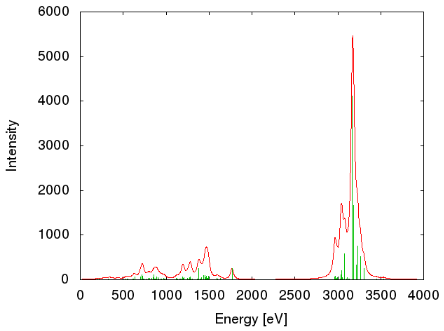

(11) isoleucine *pc*-BCX

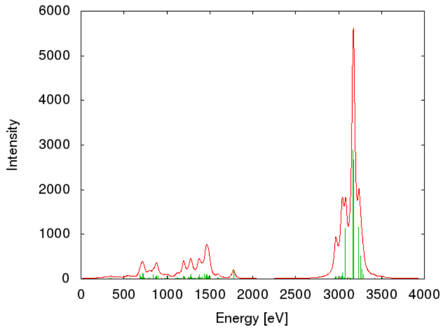

(12) leucine *pc*-BCX

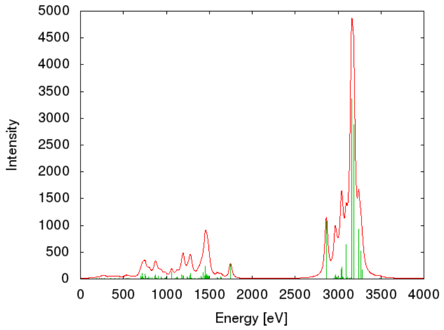

(13) lysine *pc*-BCX

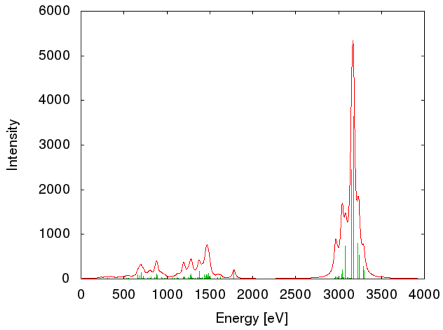

(14) methionine *pc*-BCX

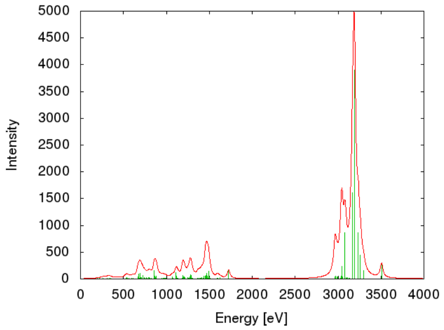

(15) phenylalanine *pc*-BCX

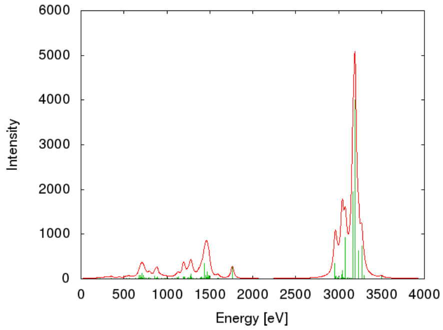

(16) proline *pc*-BCX

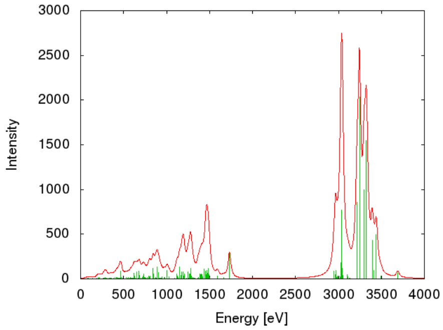

(17) serine *pc*-BCX

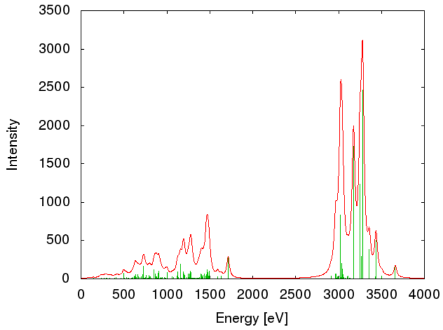

(18) threonine *pc*-BCX

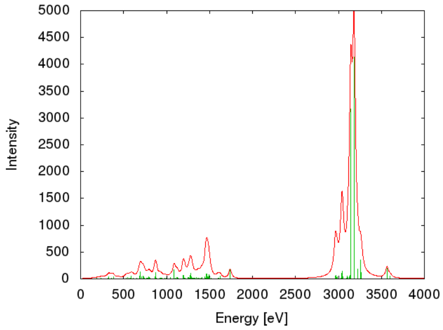

(19) tryptophan *pc*-BCX

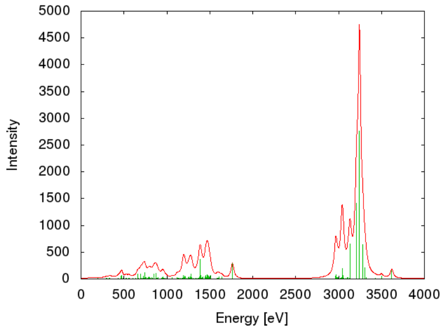

(20) tyrosine *pc*-BCX

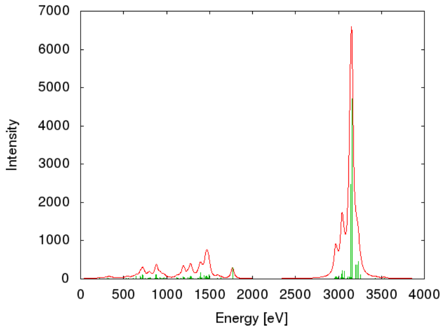

(21) valine *pc*-BCX

Figure S8: IR spectra for the *al*-BCX and its complexes with amino acids.

(1) pristine *al*-BCX

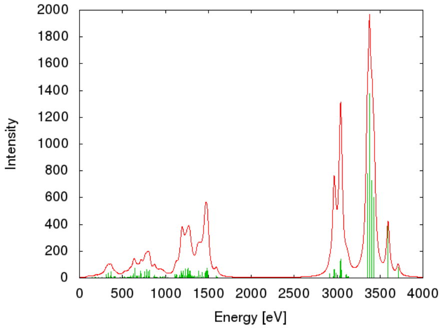

(2) alanine *al*-BCX

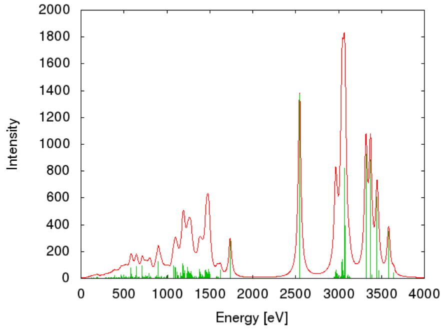

(3) asparagine *al*-BCX

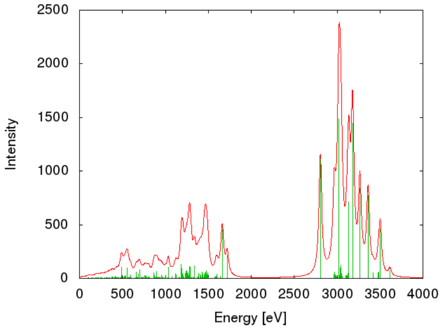

(4) aspartate *al*-BCX

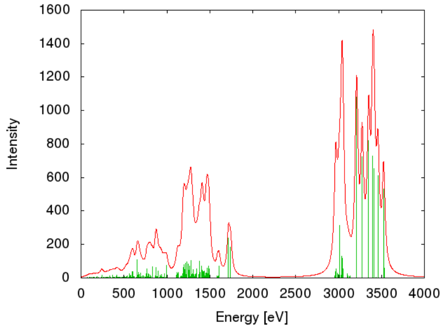

(5) cysteine *al*-BCX

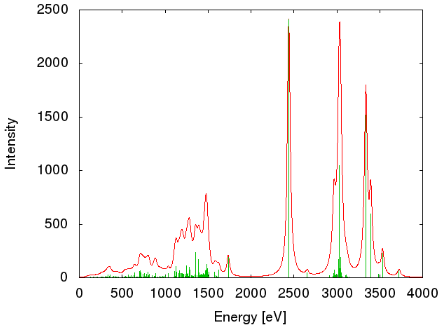

(6) glutamine *al*-BCX

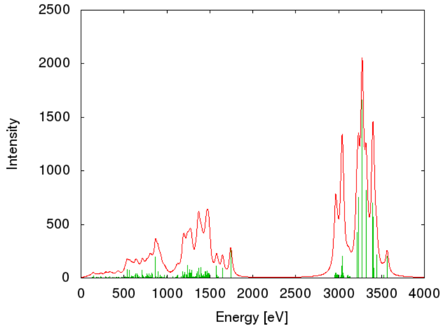

(7) glutamate *al*-BCX

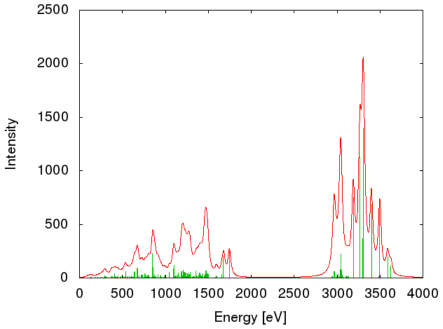

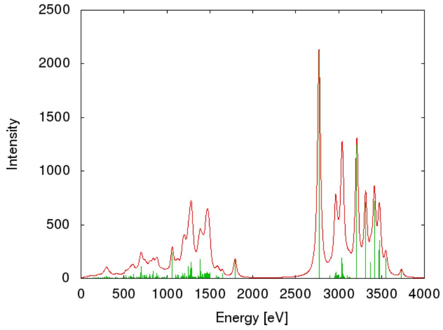

(8) glycine *al*-BCX

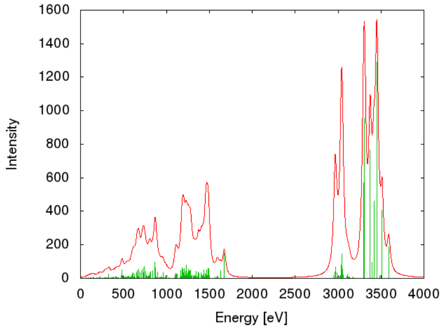

(9) histidine-D *al*-BCX

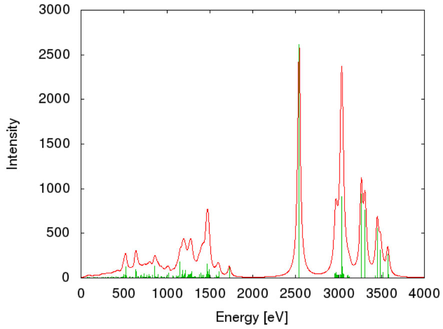

(10) histidine-E *al*-BCX

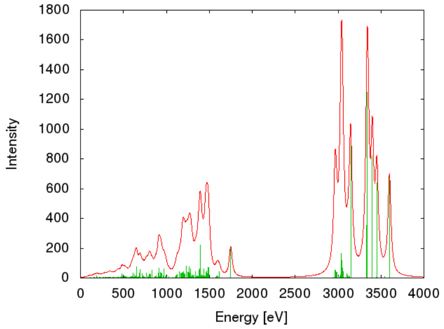

(11) isoleucine *al*-BCX

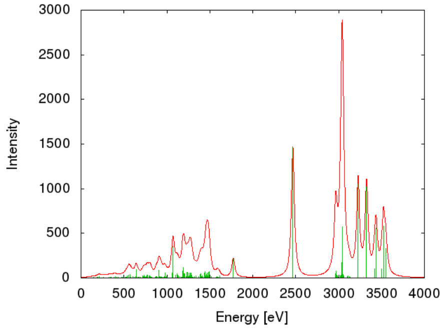

(12) leucine *al*-BCX

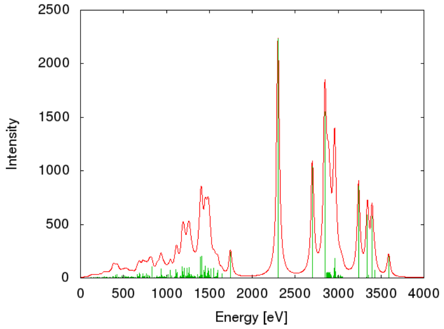

(13) lysine *al*-BCX

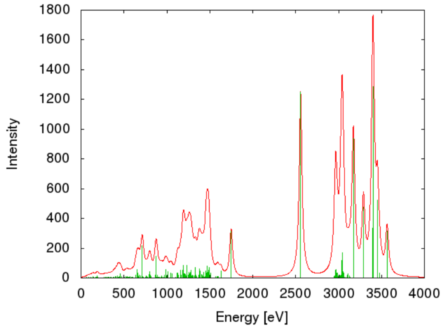

(14) methionine *al*-BCX

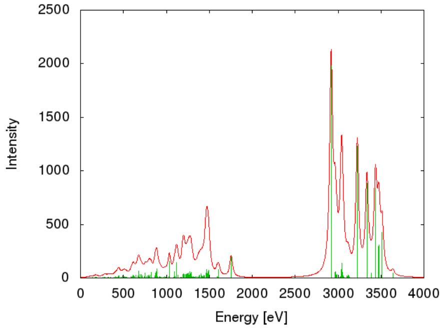

(15) phenylalanine *al*-BCX

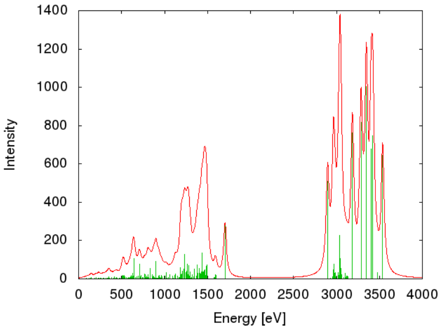

(16) proline *al*-BCX

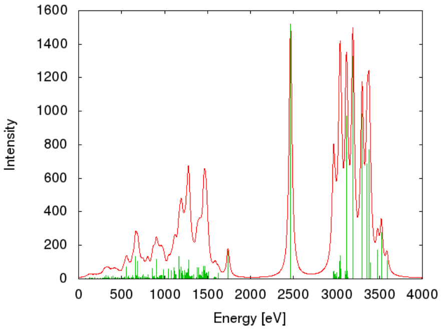

(17) serine *al*-BCX

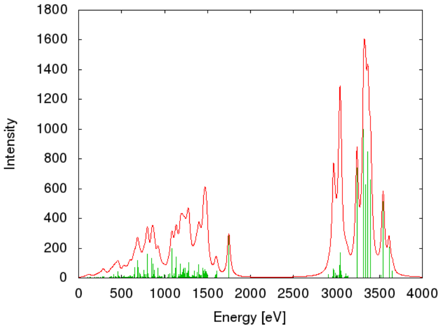

(18) threonine *al*-BCX

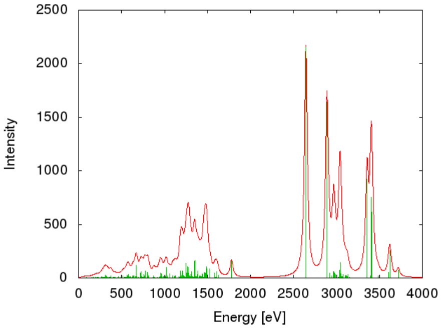

(19) tryptophan *al*-BCX

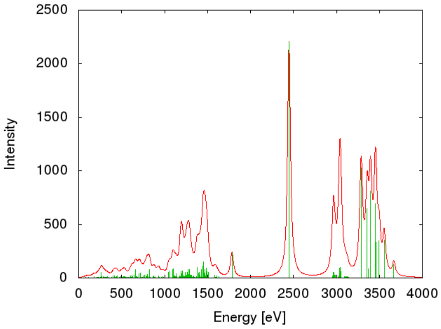

(20) tyrosine *al*-BCX

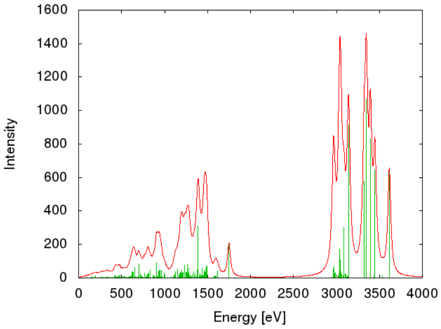

(21) valine *al*-BCX

Figure S9: IR spectra for the *wc*-BCX and its complexes with amino acids.

(1) pristine *wc*-BCX

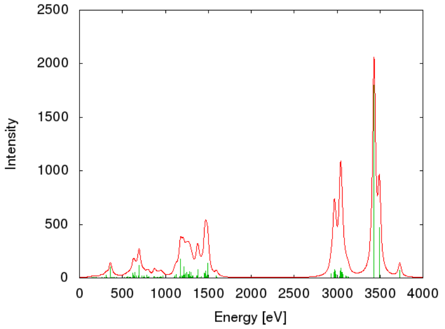

(2) alanine *wc*-BCX

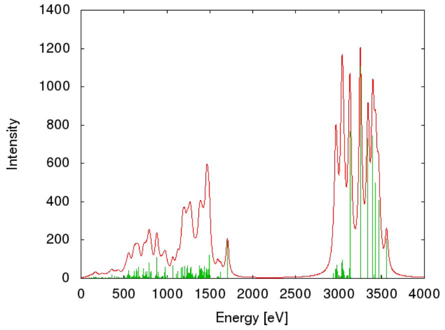

(3) asparagine *wc*-BCX

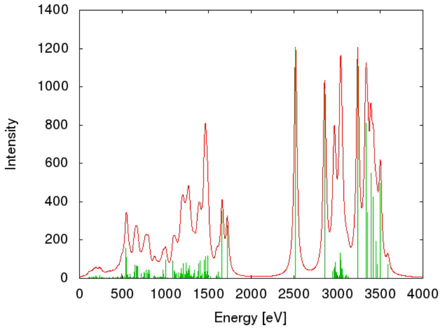

(4) aspartate *wc*-BCX

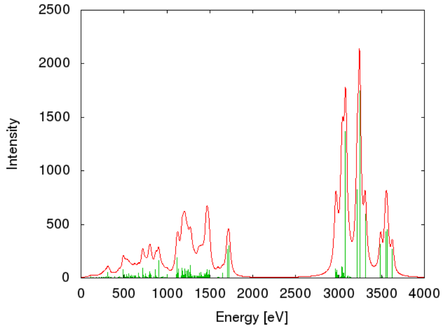

(5) cysteine *wc*-BCX

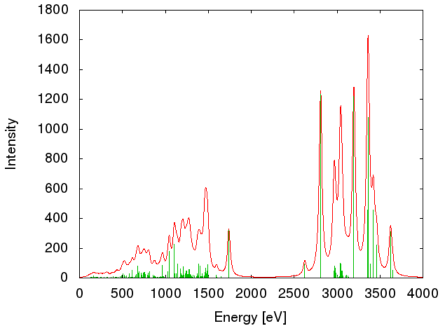

(6) glutamine *wc*-BCX

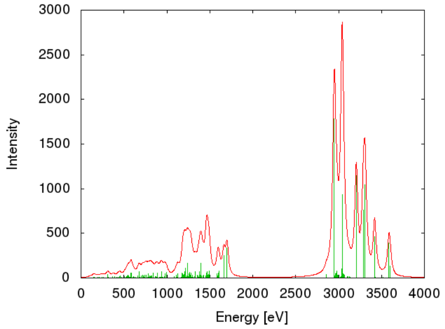

(7) glutamate *wc*-BCX

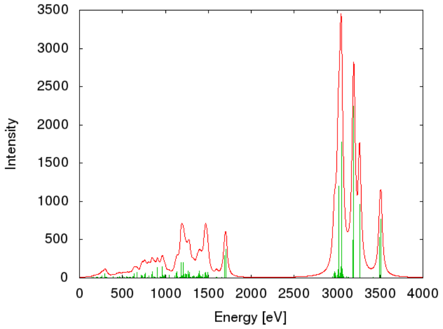

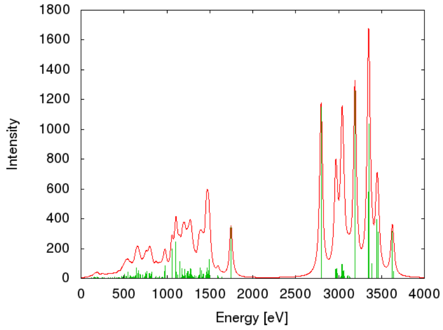

(8) glycine *wc*-BCX

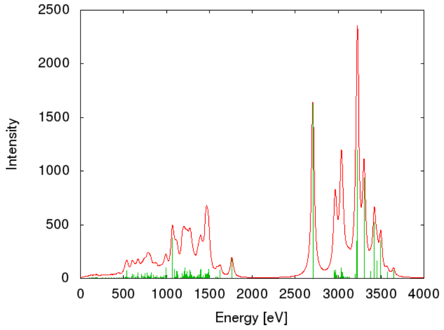

(9) histidine-D *wc*-BCX

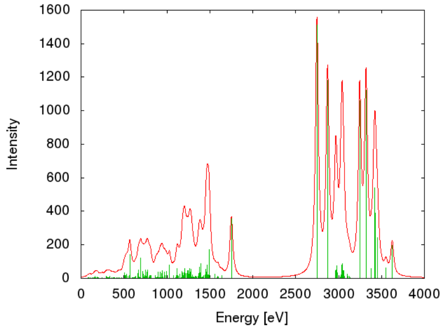

(10) histidine-E *wc*-BCX

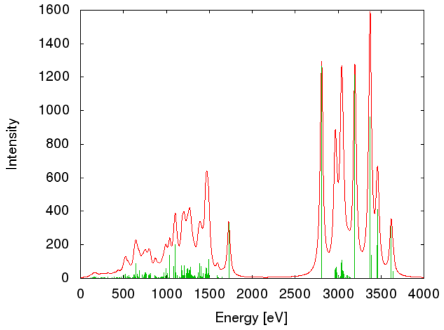

(11) isoleucine *wc*-BCX

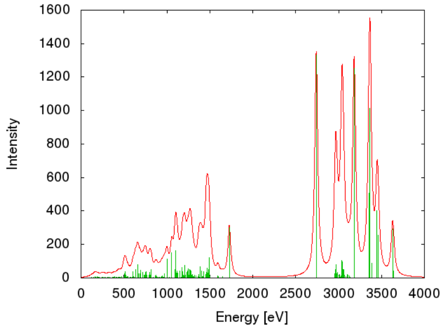

(12) leucine *wc*-BCX

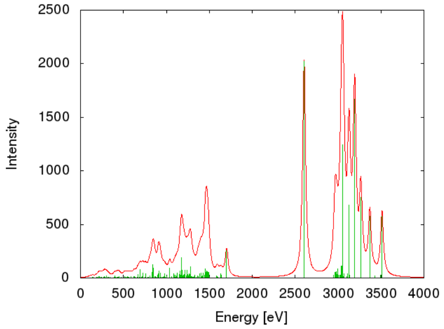

(13) lysine *wc*-BCX

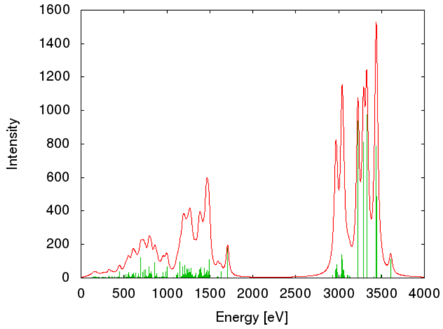

(14) methionine *wc*-BCX

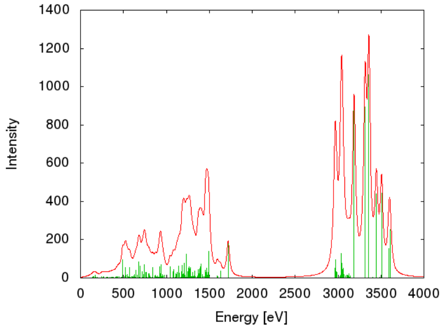

(15) phenylalanine *wc*-BCX

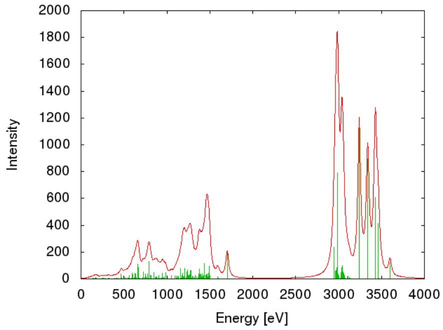

(16) proline *wc*-BCX

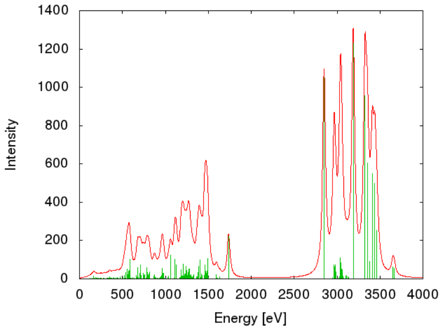

(17) serine *wc*-BCX

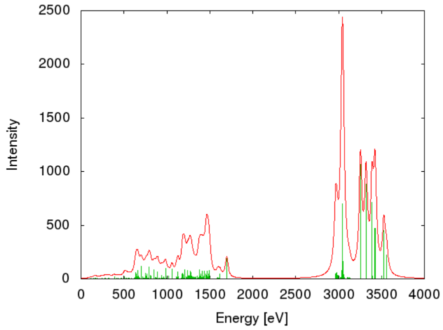

(18) threonine *wc*-BCX

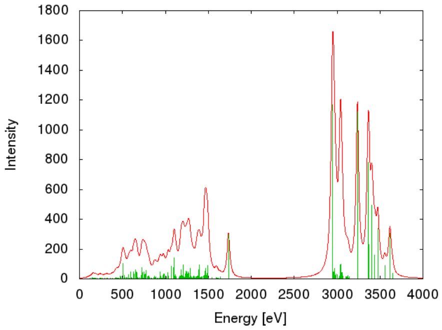

(19) tryptophan *wc*-BCX

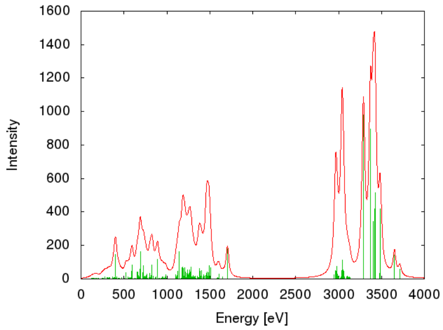

(20) tyrosine *wc*-BCX

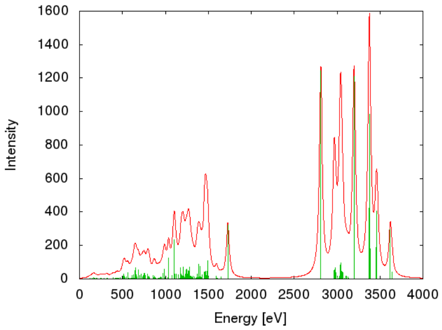

(21) valine *wc*-BCX

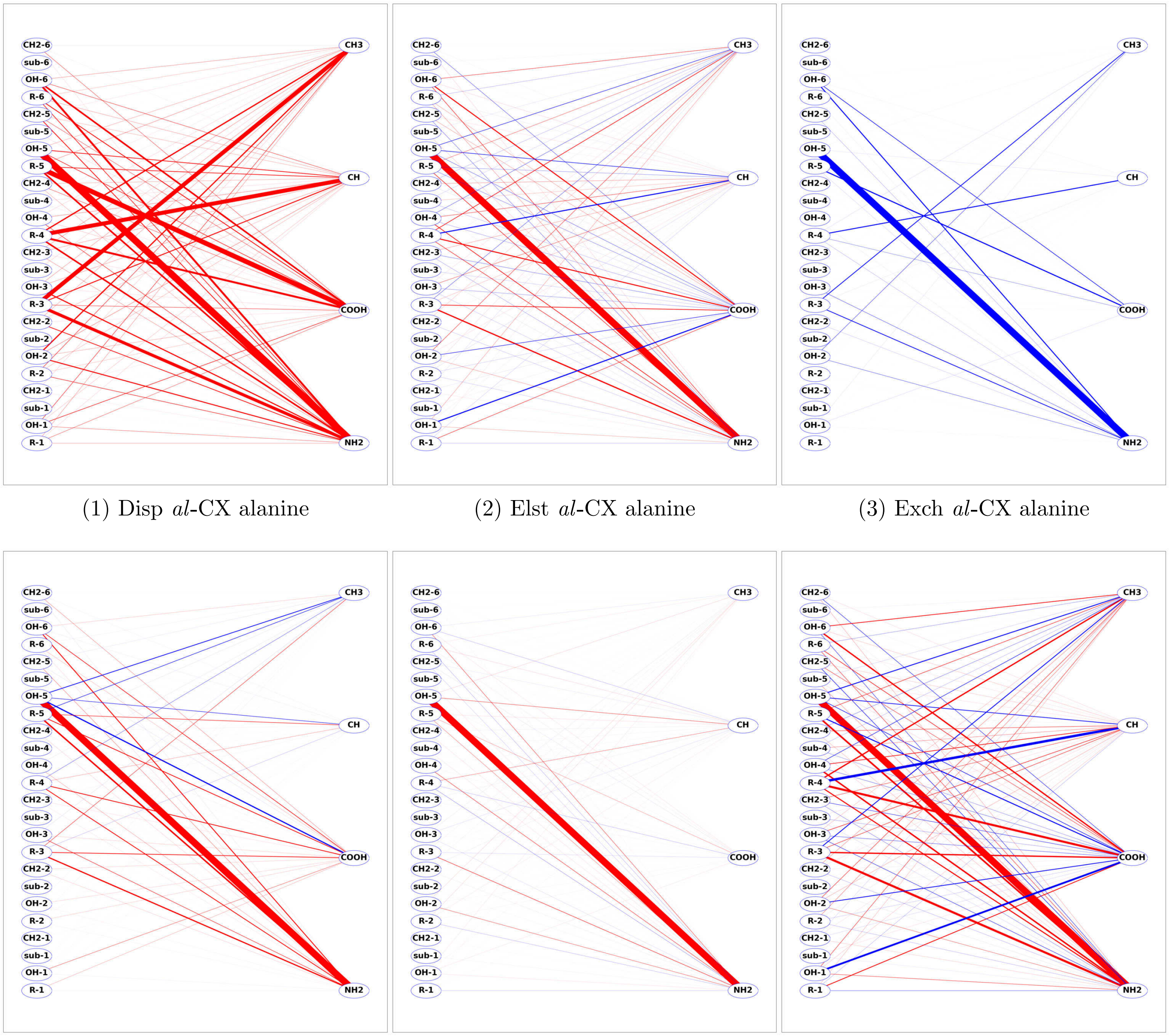

(1) Disp *al*-CX alanine (2) Elst *al*-CX alanine (3) Exch *al*-CX alanine

(4) IndAB *al*-CX alanine (5) IndBA *al*-CX alanine (6) Total *al*-CX alanine

Figure S10: The F-SAPT partitioning for *al*-CX alanine.
In this and the following figures the Elst and Exch denote the first-order electrostatic and exchange components, IndAB and IndBA – effective (i.e. induction plus exchange-induction) second-order induction components for the monomer B polarizing the monomer A and *vice versa*, and finally Disp – is the effective second-order dispersion component, where all components were obtained at the SAPT0 level with the jun-cc-pVDZ basis set.

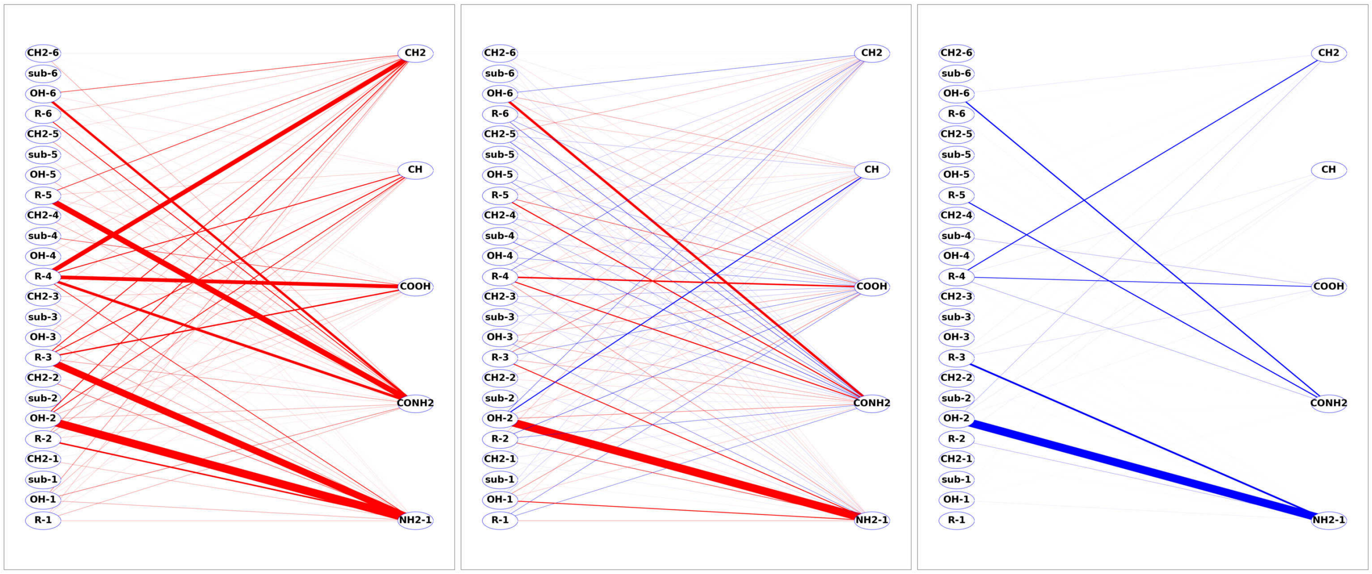

(1) Disp *al*-CX asparagine (2) Elst *al*-CX asparagine (3) Exch *al*-CX asparagine

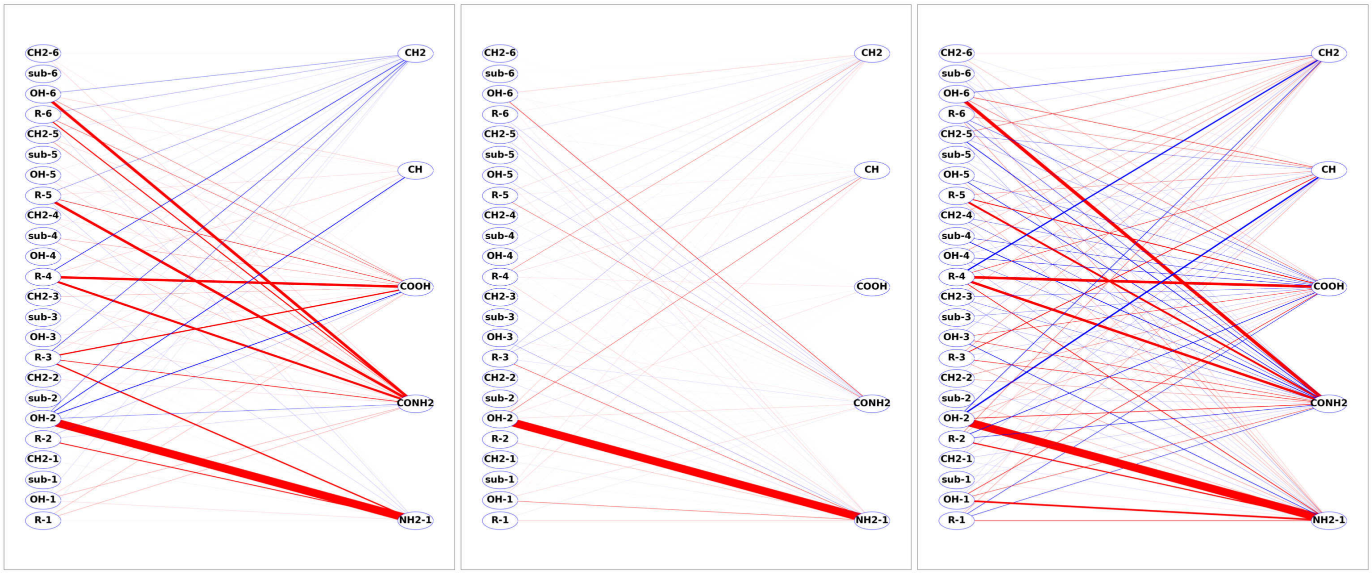

(4) IndAB *al*-CX asparagine (5) IndBA *al*-CX asparagine (6) Total *al*-CX asparagine

Figure S11: The F-SAPT partitioning for *al*-CX asparagine

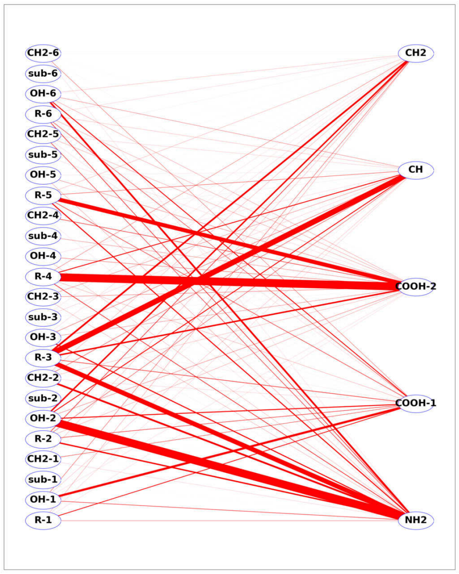

(1) Disp *al*-CX aspartate

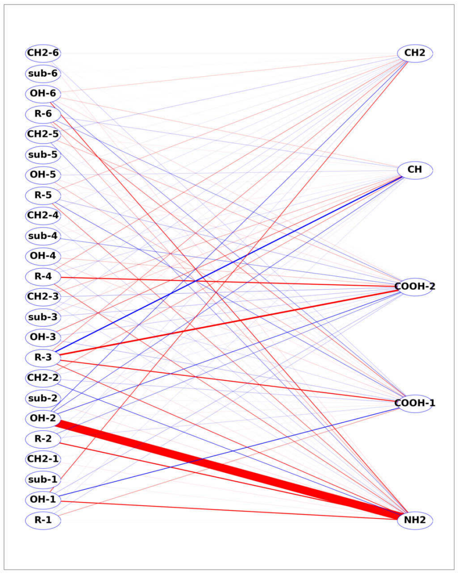

(2) Elst *al*-CX aspartate

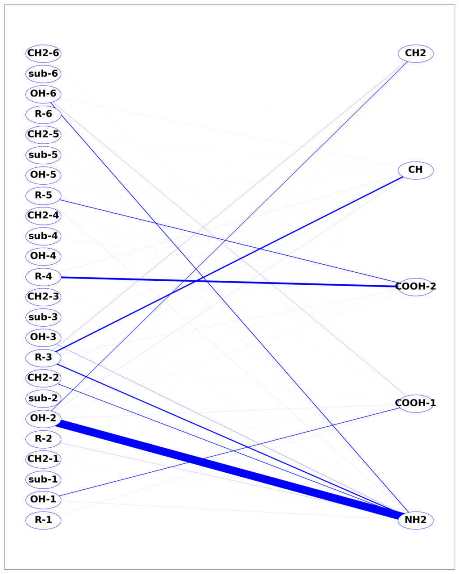

(3) Exch *al*-CX aspartate

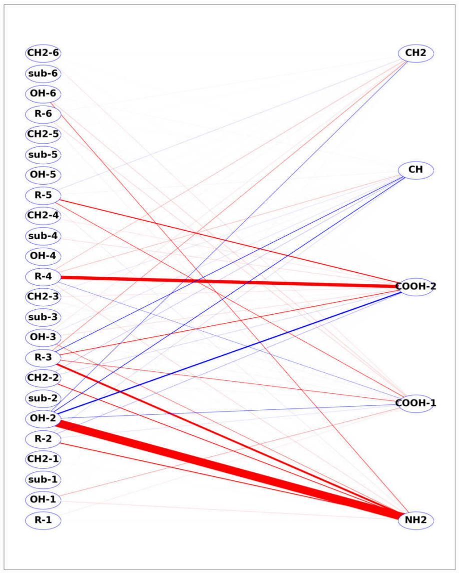

(4) IndAB *al*-CX aspartate

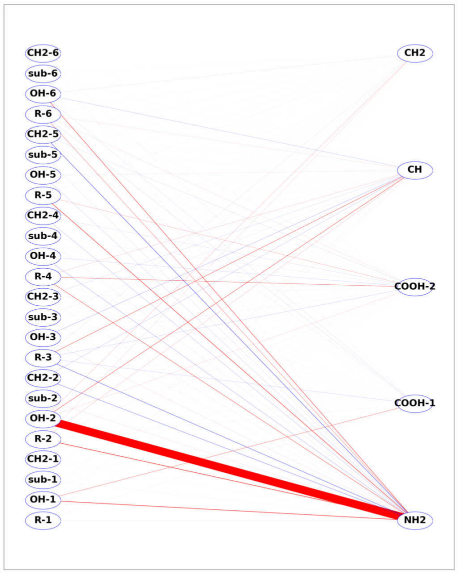

(5) IndBA *al*-CX aspartate

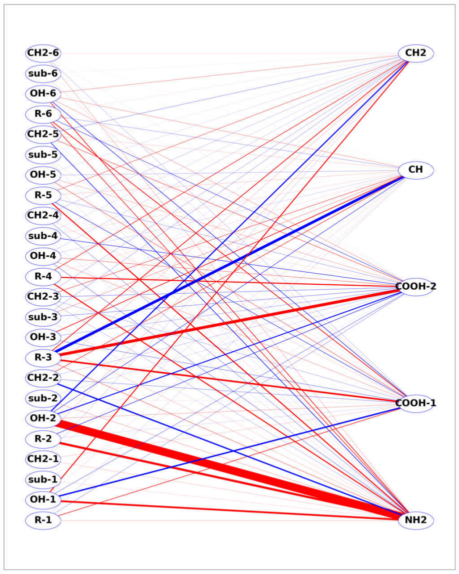

(6) Total *al*-CX aspartate

Figure S12: The F-SAPT partitioning for *al*-CX aspartate

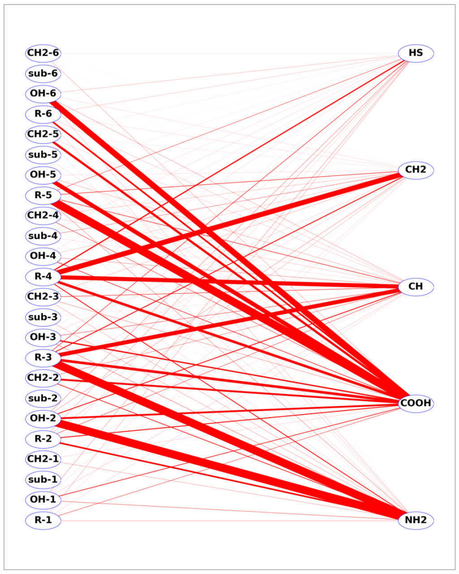

(1) Disp *al*-CX cysteine

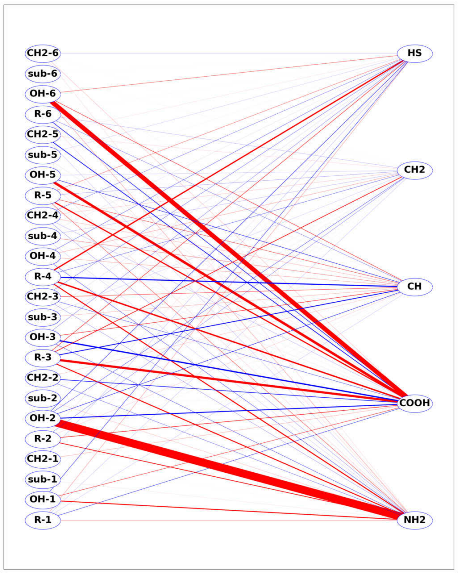

(2) Elst *al*-CX cysteine

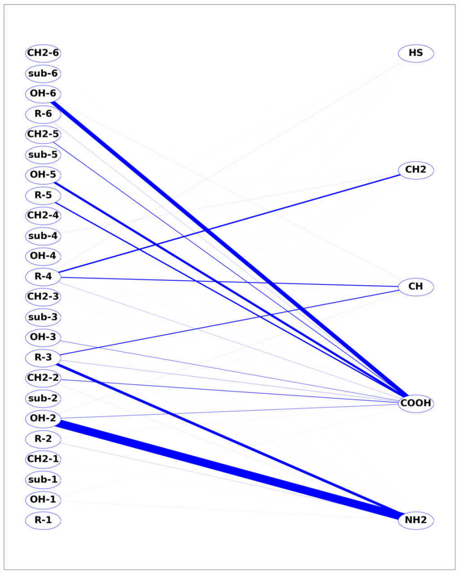

(3) Exch *al*-CX cysteine

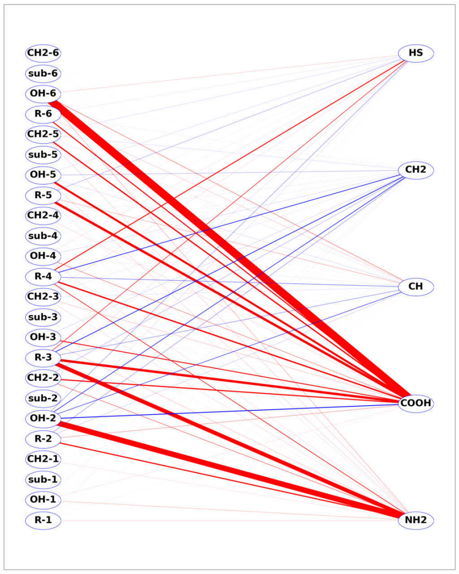

(4) IndAB *al*-CX cysteine

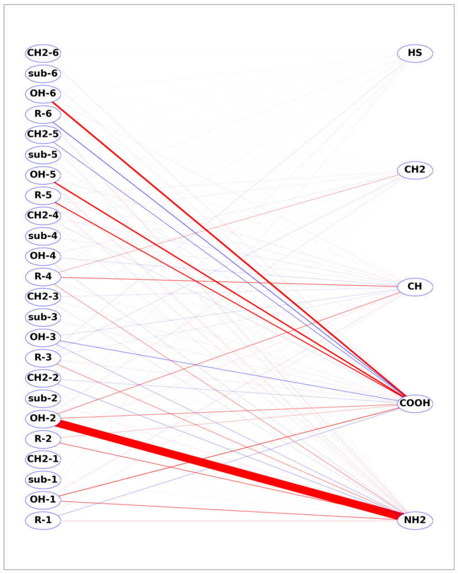

(5) IndBA *al*-CX cysteine

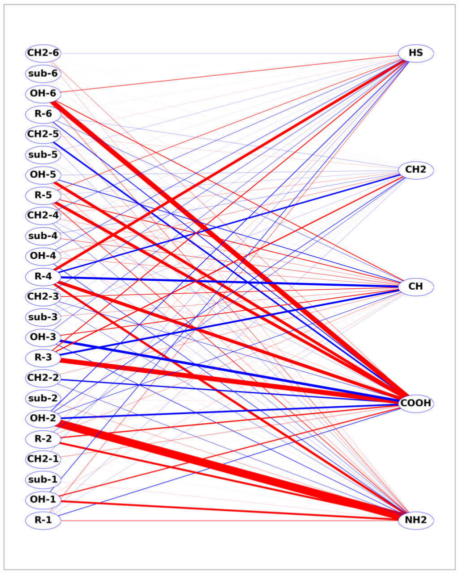

(6) Total *al*-CX cysteine

Figure S13: The F-SAPT partitioning for *al*-CX cysteine

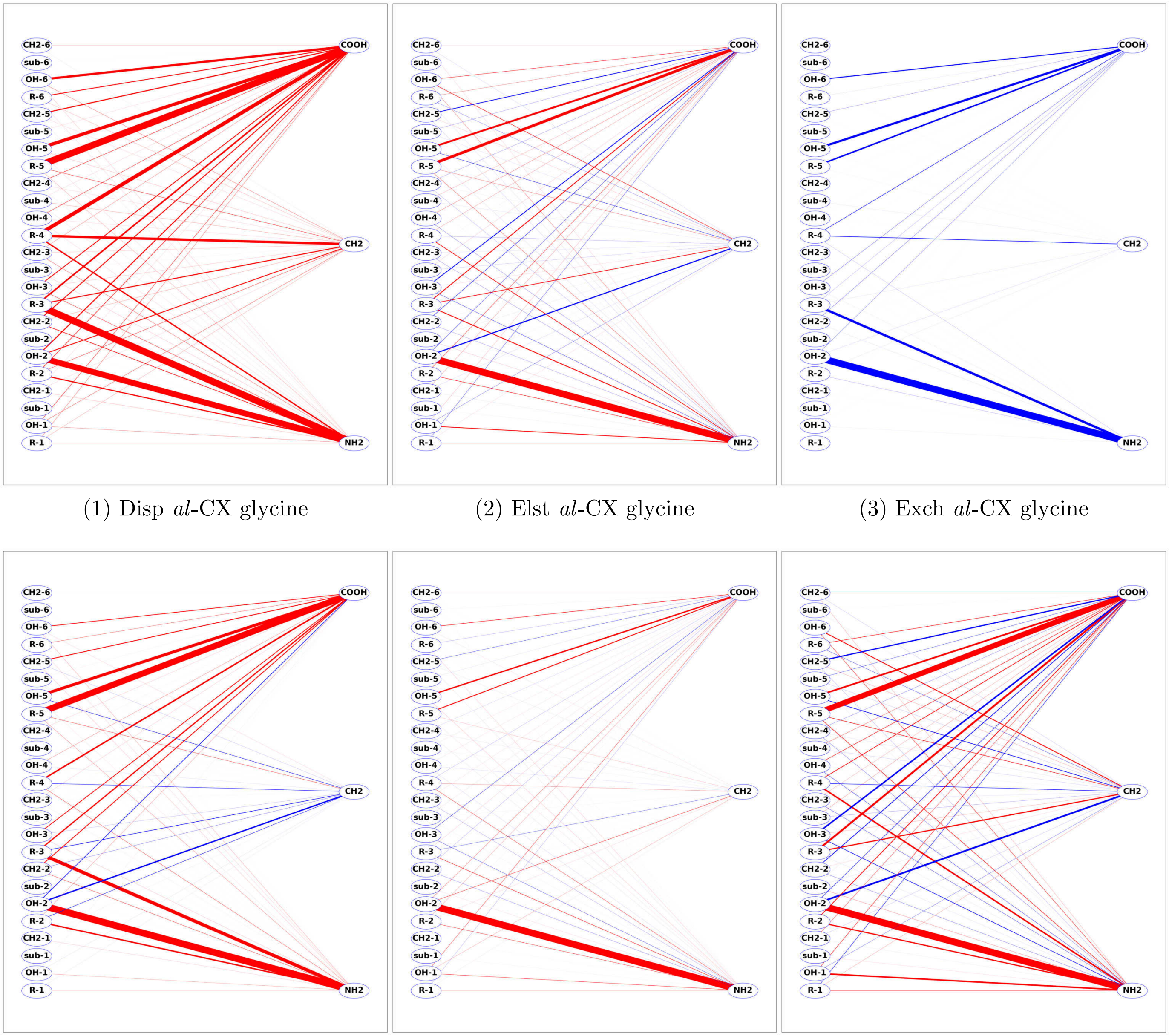

(1) Disp *al*-CX glycine (2) Elst *al*-CX glycine (3) Exch *al*-CX glycine

(4) IndAB *al*-CX glycine (5) IndBA *al*-CX glycine (6) Total *al*-CX glycine

Figure S14: The F-SAPT partitioning for *al*-CX glycine

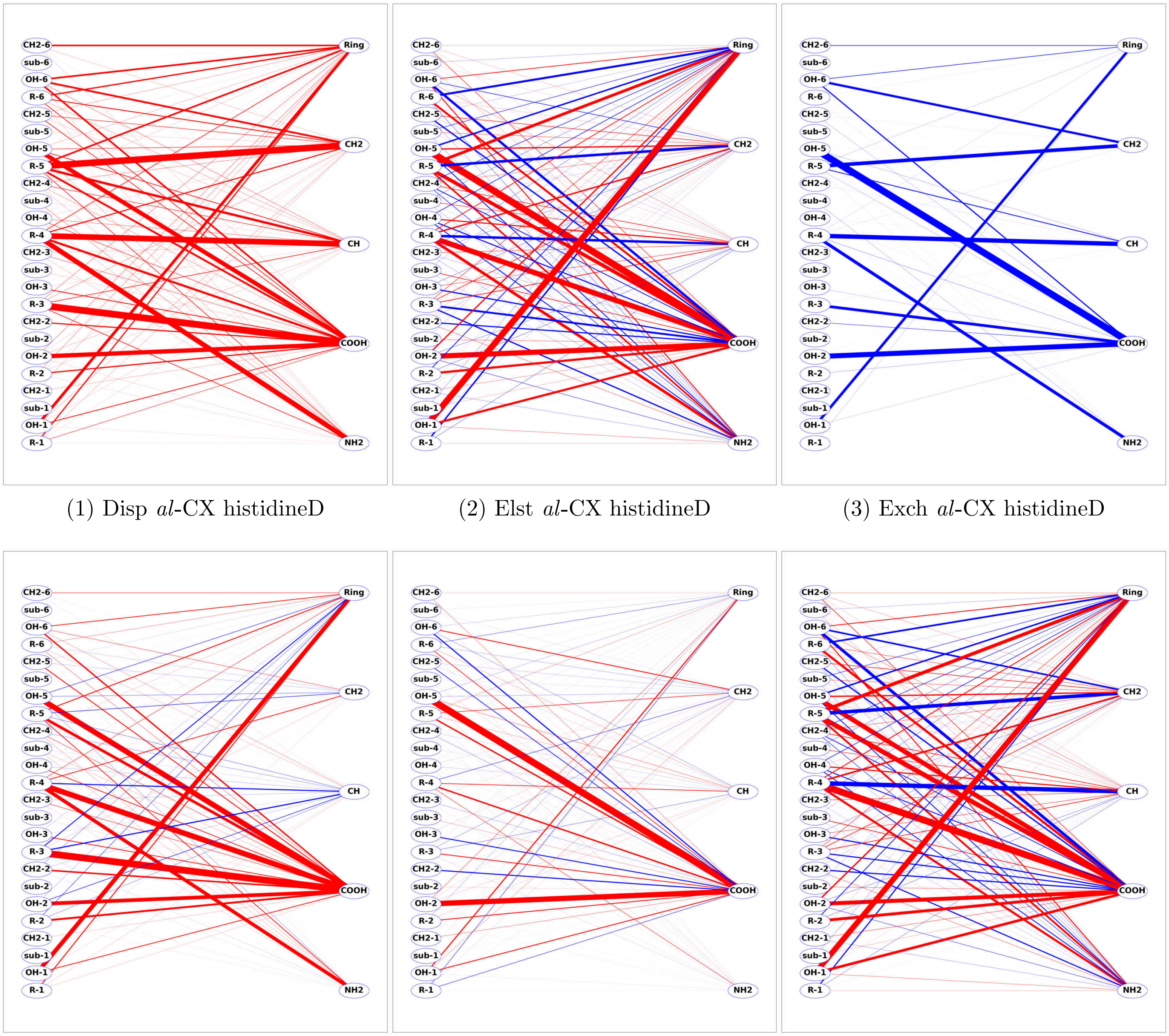

(1) Disp *al*-CX histidineD (2) Elst *al*-CX histidineD (3) Exch *al*-CX histidineD

(4) IndAB *al*-CX histidineD (5) IndBA *al*-CX histidineD (6) Total *al*-CX histidineD

Figure S15: The F-SAPT partitioning for *al*-CX histidineD

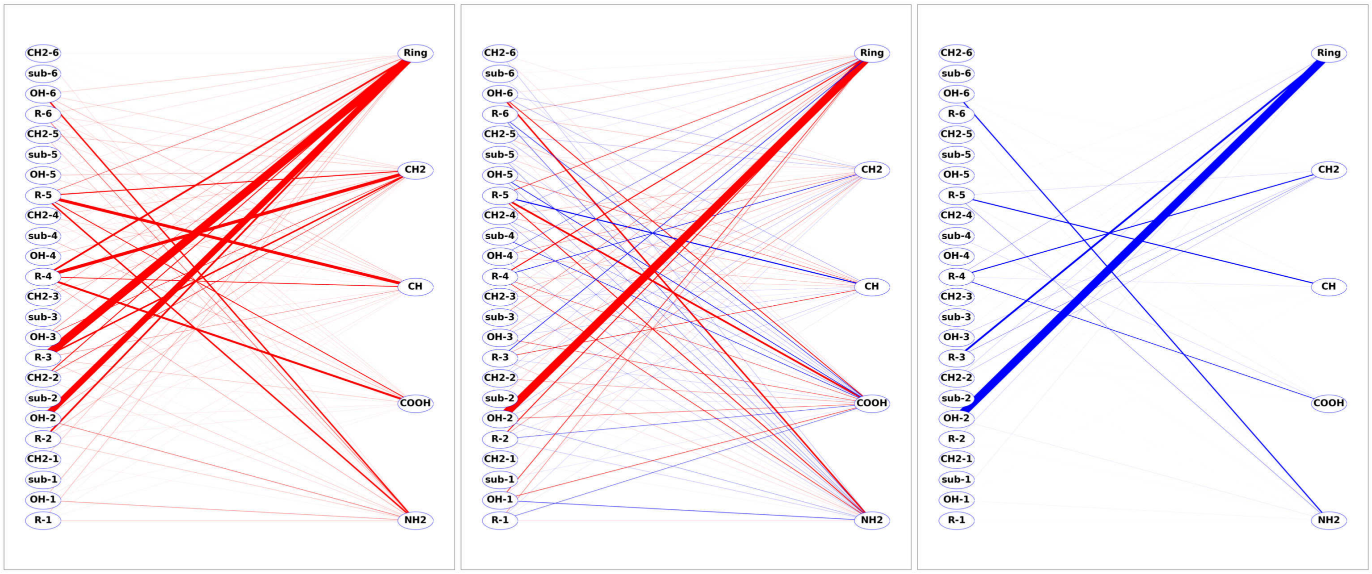

(1) Disp *al*-CX histidineE (2) Elst *al*-CX histidineE (3) Exch *al*-CX histidineE

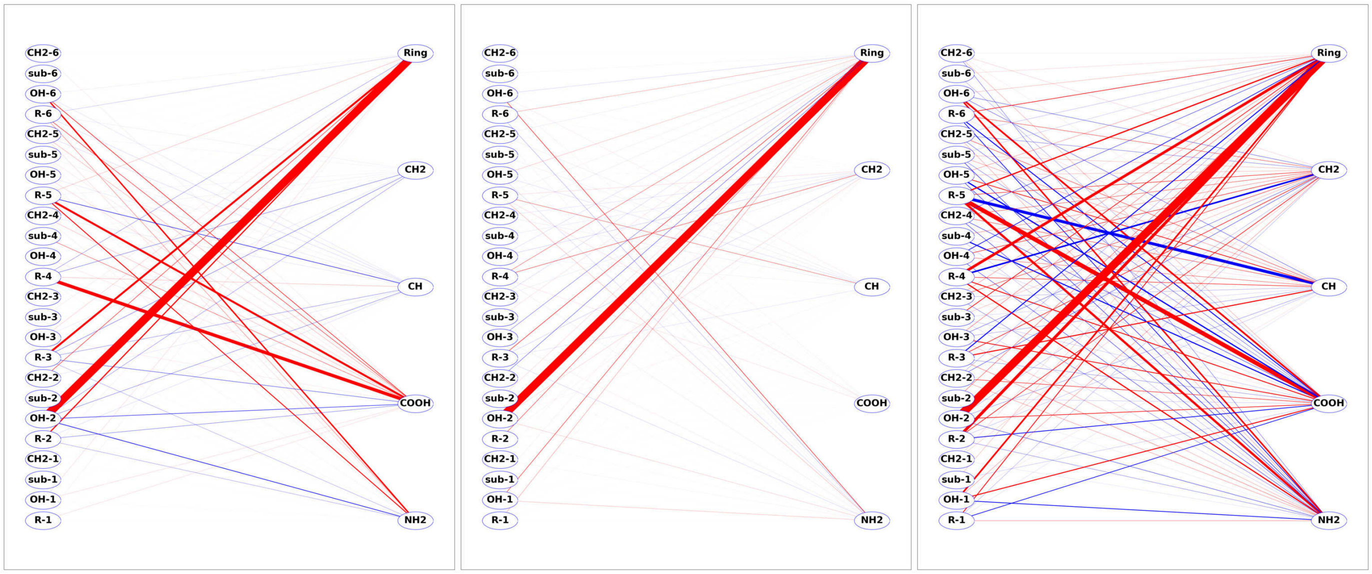

(4) IndAB *al*-CX histidineE (5) IndBA *al*-CX histidineE (6) Total *al*-CX histidineE

Figure S16: The F-SAPT partitioning for *al*-CX histidineE

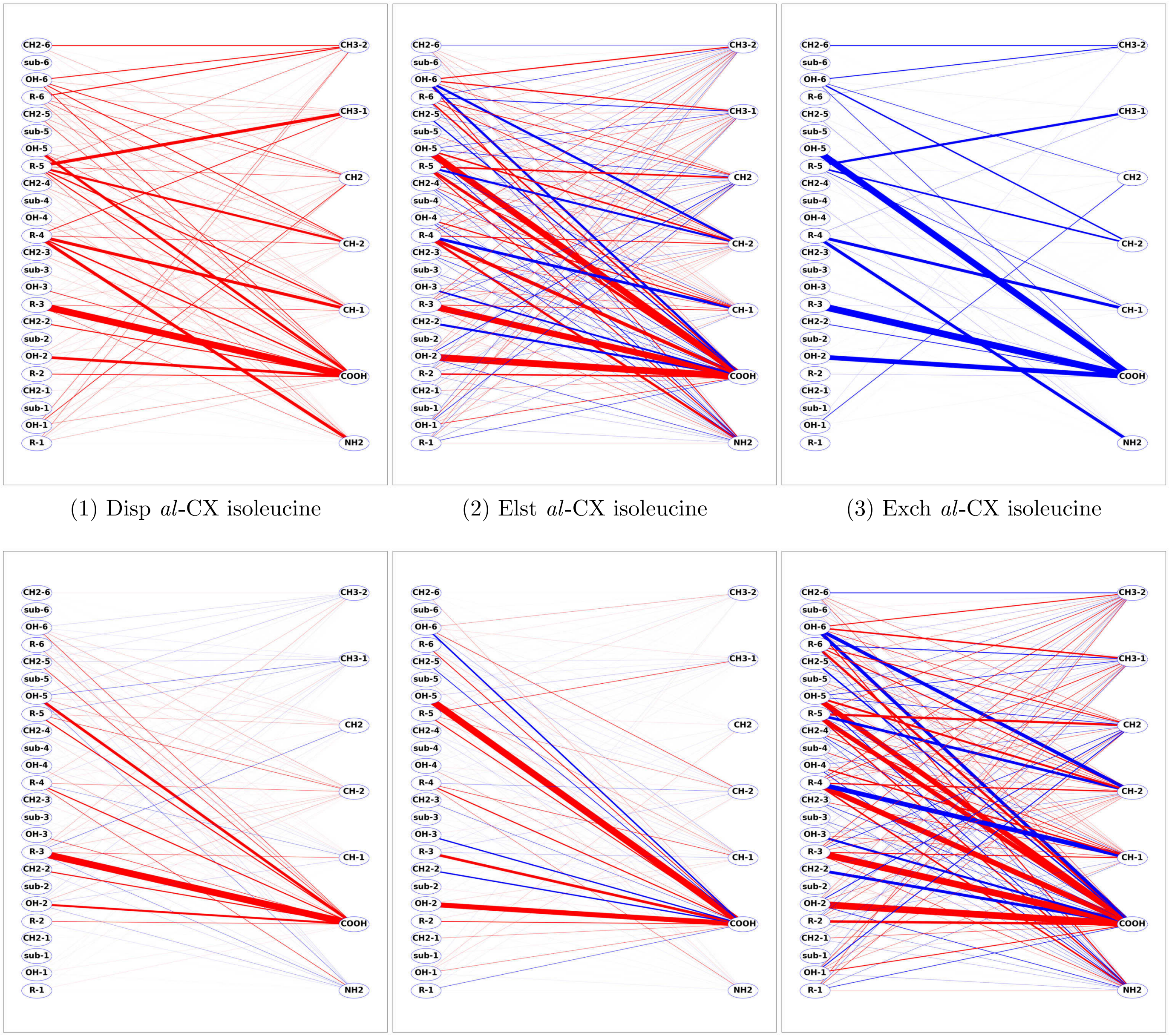

(1) Disp *al*-CX isoleucine | (2) Elst *al*-CX isoleucine | (3) Exch *al*-CX isoleucine

(4) IndAB *al*-CX isoleucine | (5) IndBA *al*-CX isoleucine | (6) Total *al*-CX isoleucine

Figure S17: The F-SAPT partitioning for *al*-CX isoleucine

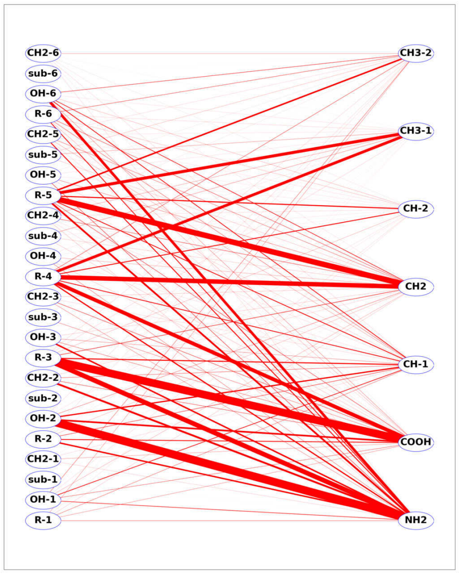

(1) Disp *al*-CX leucine

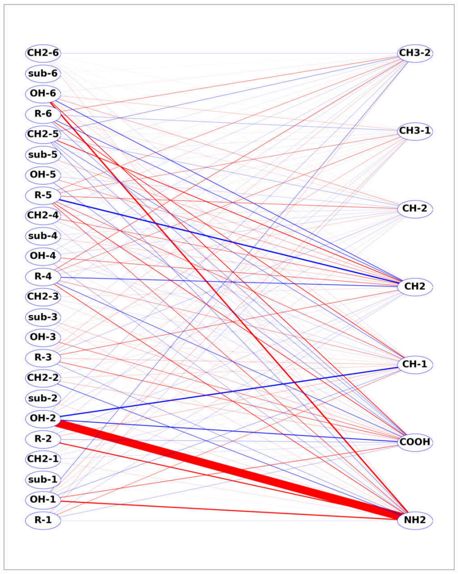

(2) Elst *al*-CX leucine

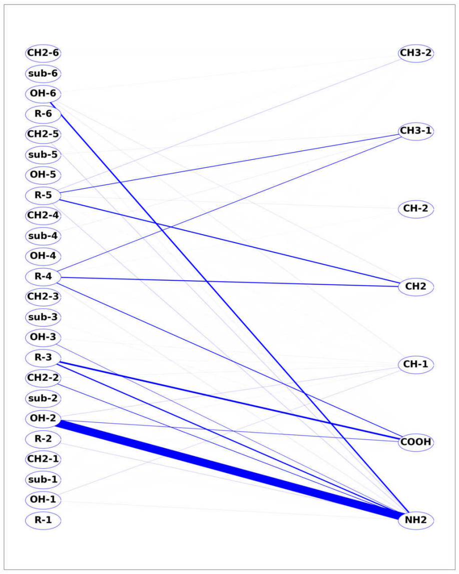

(3) Exch *al*-CX leucine

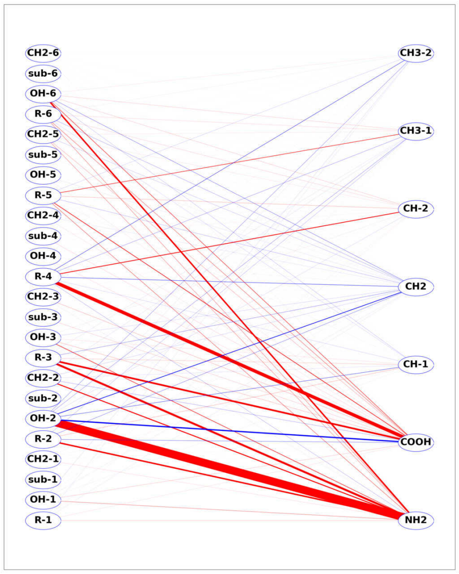

(4) IndAB *al*-CX leucine

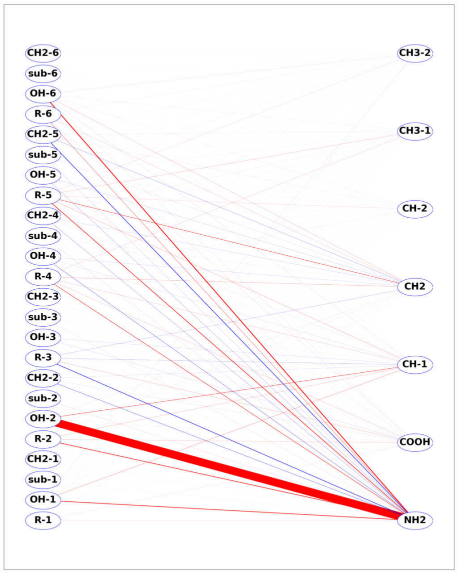

(5) IndBA *al*-CX leucine

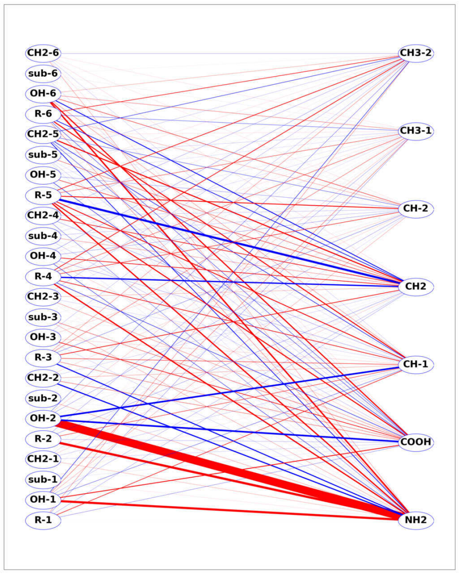

(6) Total *al*-CX leucine

Figure S18: The F-SAPT partitioning for *al*-CX leucine

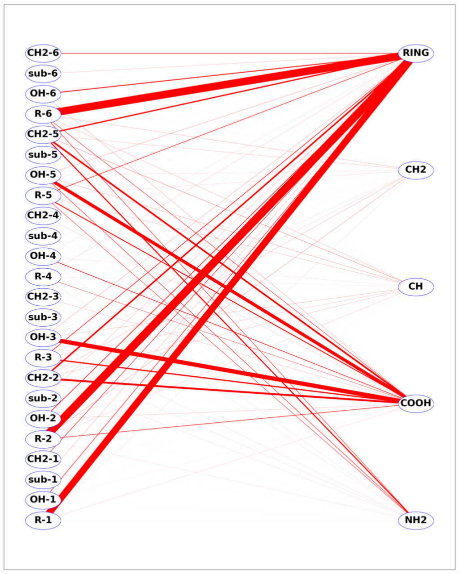

(1) Disp *al*-CX phenylalanine

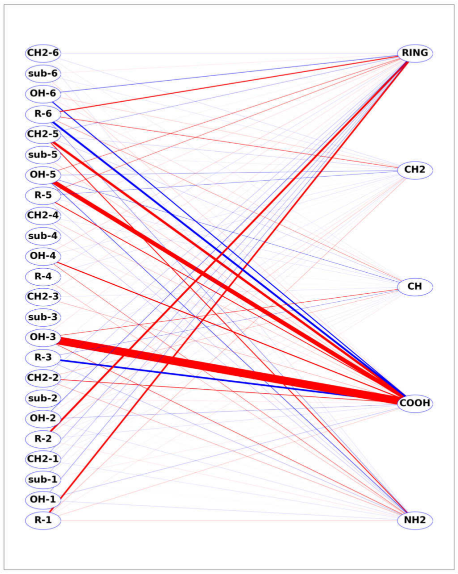

(2) Elst *al*-CX phenylalanine

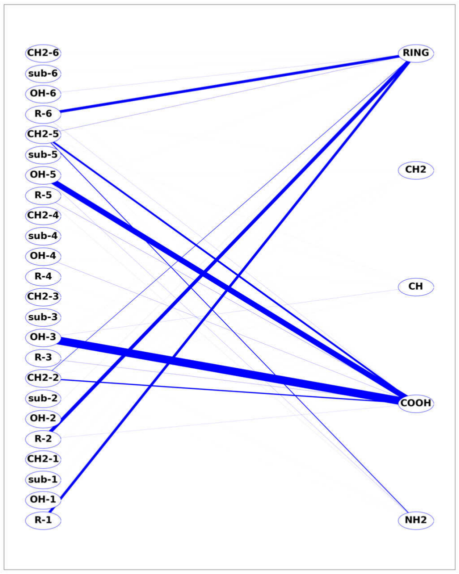

(3) Exch *al*-CX phenylalanine

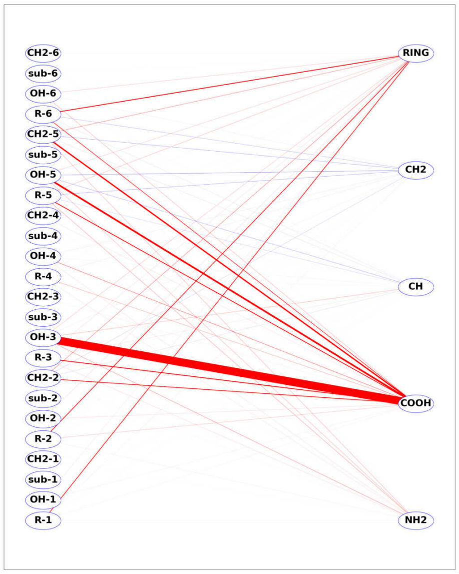

(4) IndAB *al*-CX phenylalanine

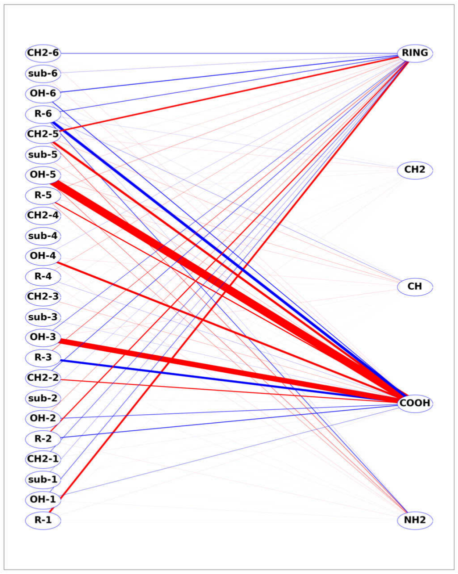

(5) IndBA *al*-CX phenylalanine

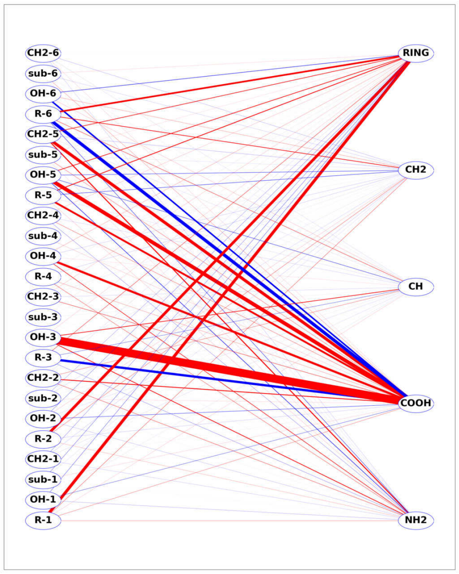

(6) Total *al*-CX phenylalanine

Figure S19: The F-SAPT partitioning for *al*-CX phenylalanine

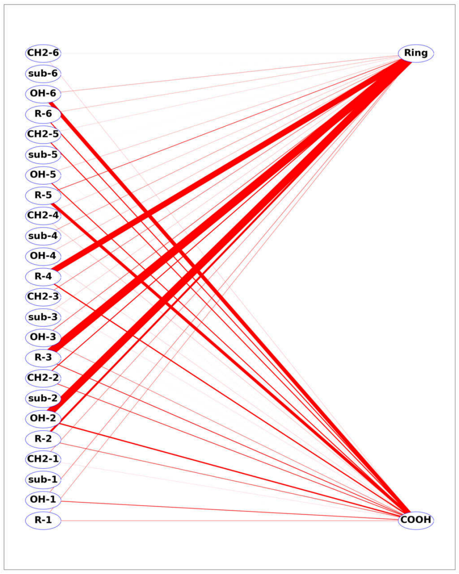

(1) Disp *al*-CX proline

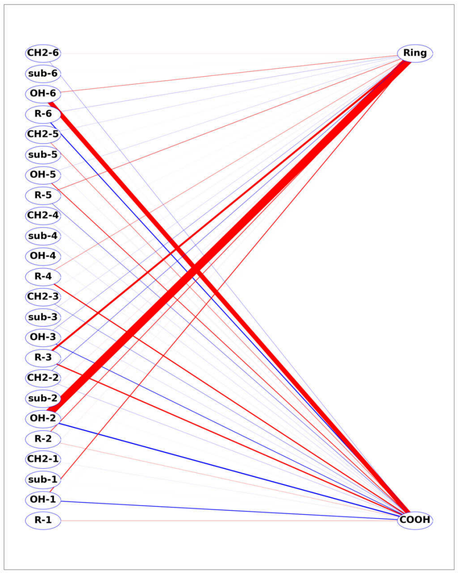

(2) Elst *al*-CX proline

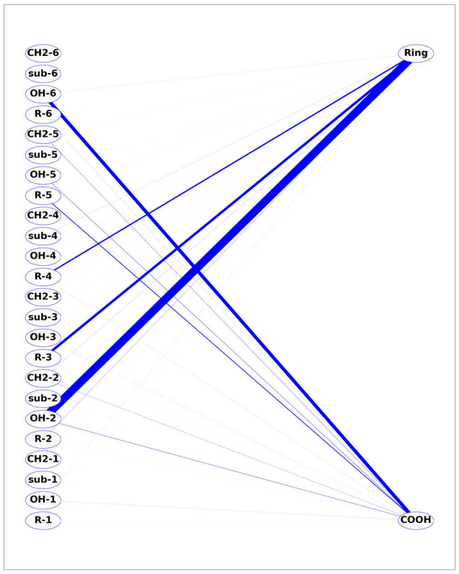

(3) Exch *al*-CX proline

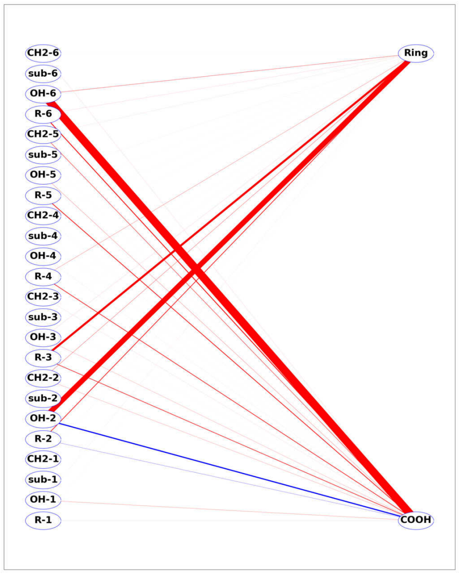

(4) IndAB *al*-CX proline

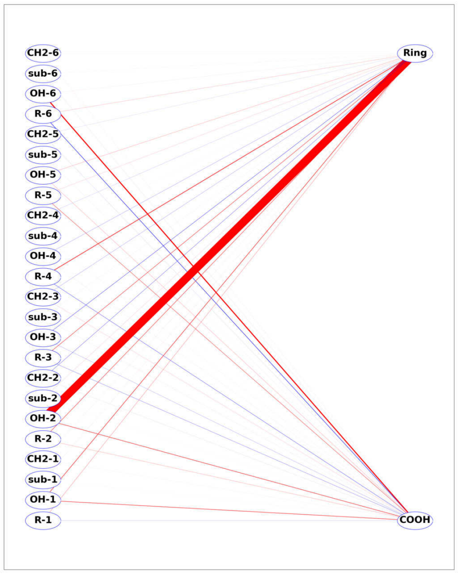

(5) IndBA *al*-CX proline

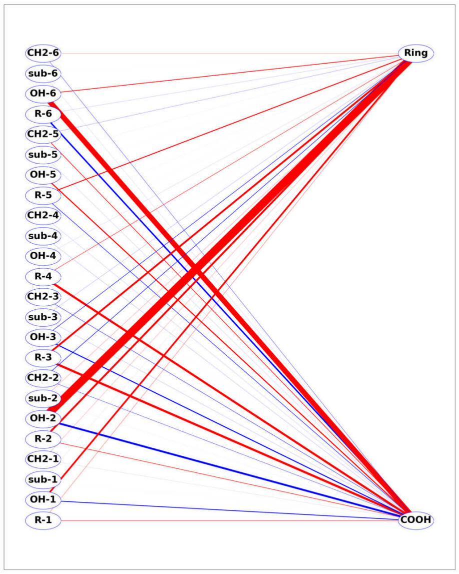

(6) Total *al*-CX proline

Figure S20: The F-SAPT partitioning for *al*-CX proline

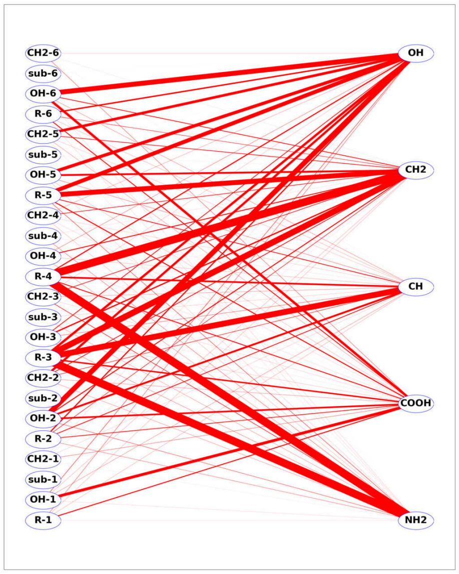

(1) Disp *al*-CX serine

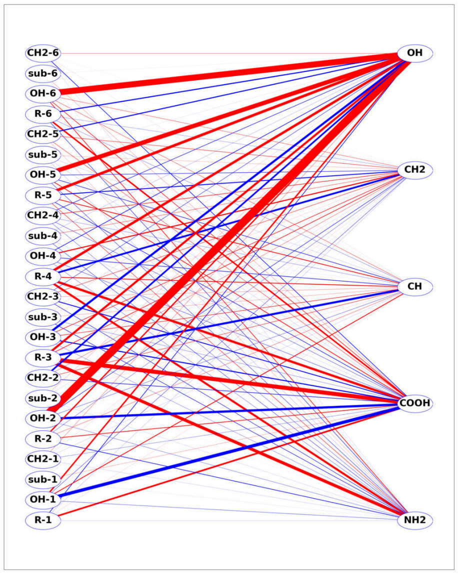

(2) Elst *al*-CX serine

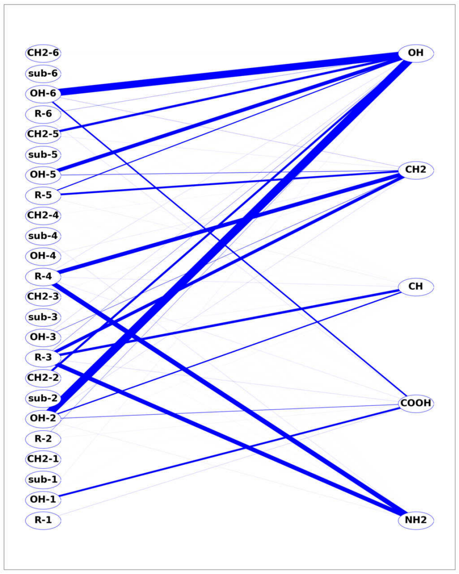

(3) Exch *al*-CX serine

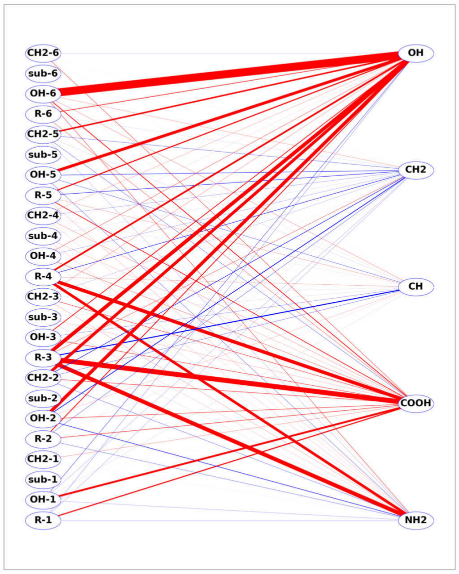

(4) IndAB *al*-CX serine

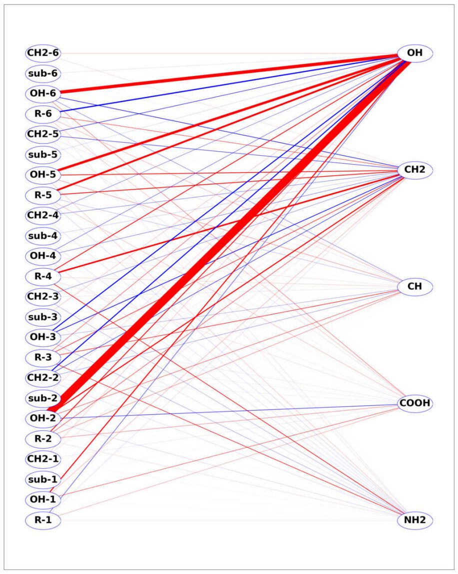

(5) IndBA *al*-CX serine

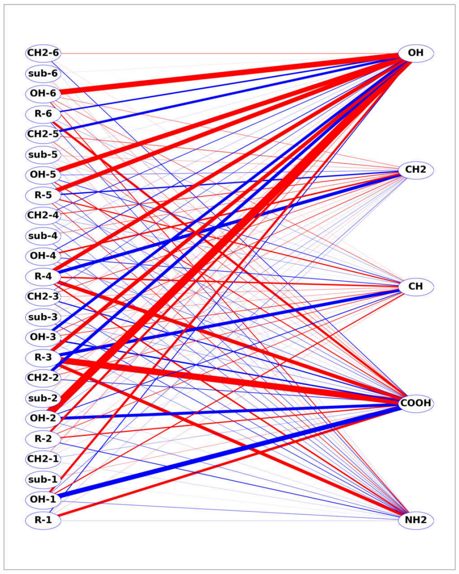

(6) Total *al*-CX serine

Figure S21: The F-SAPT partitioning for *al*-CX serine

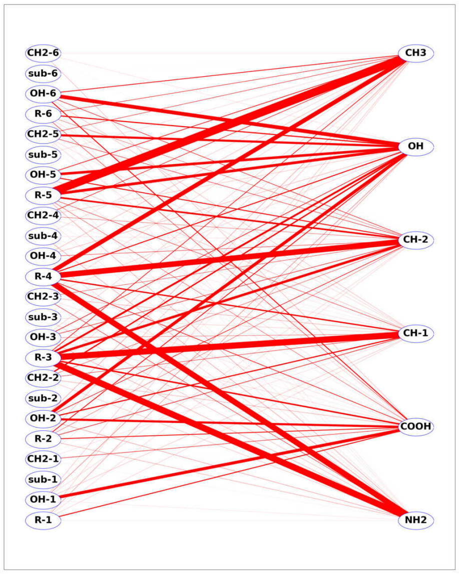

(1) Disp *al*-CX threonine

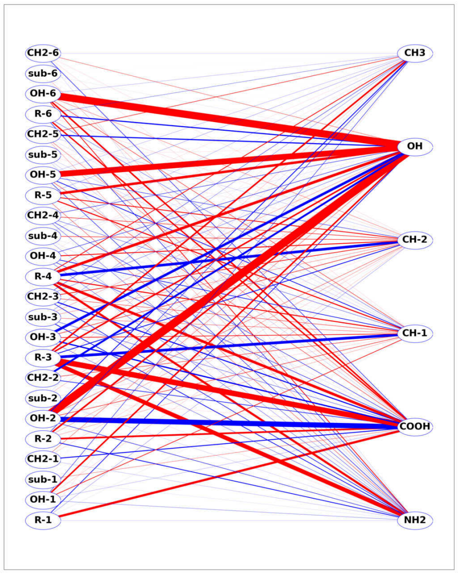

(2) Elst *al*-CX threonine

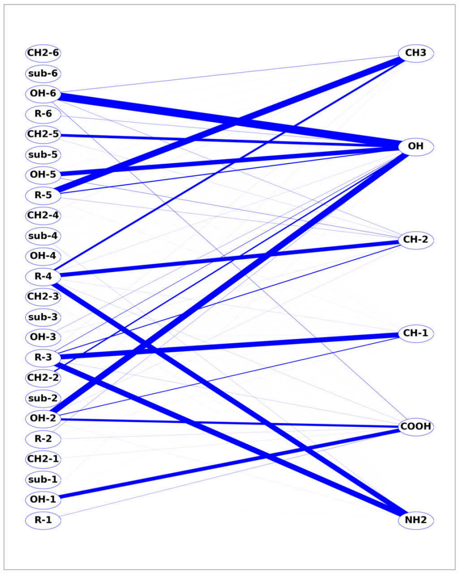

(3) Exch *al*-CX threonine

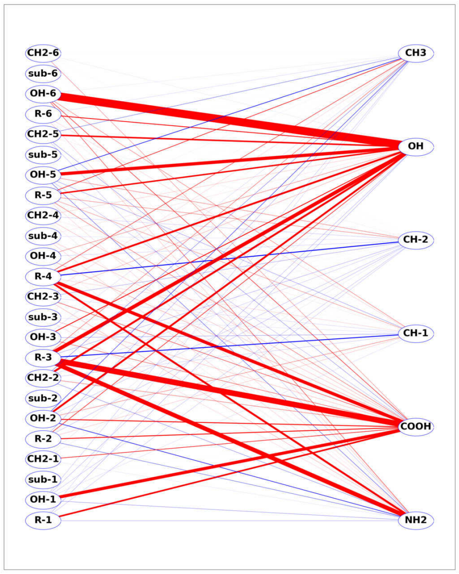

(4) IndAB *al*-CX threonine

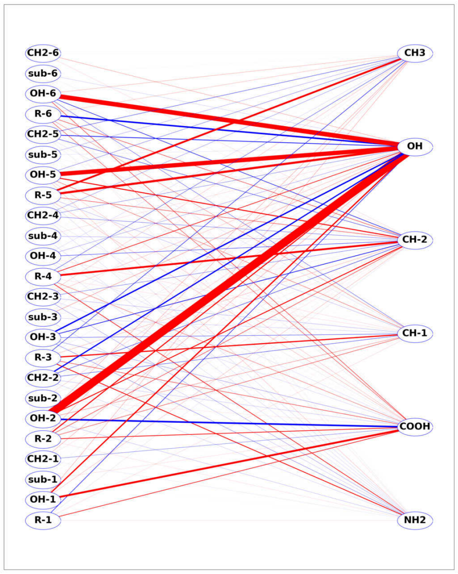

(5) IndBA *al*-CX threonine

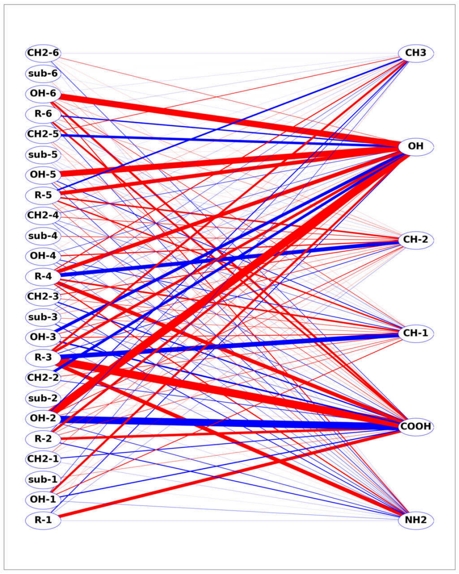

(6) Total *al*-CX threonine

Figure S22: The F-SAPT partitioning for *al*-CX threonine

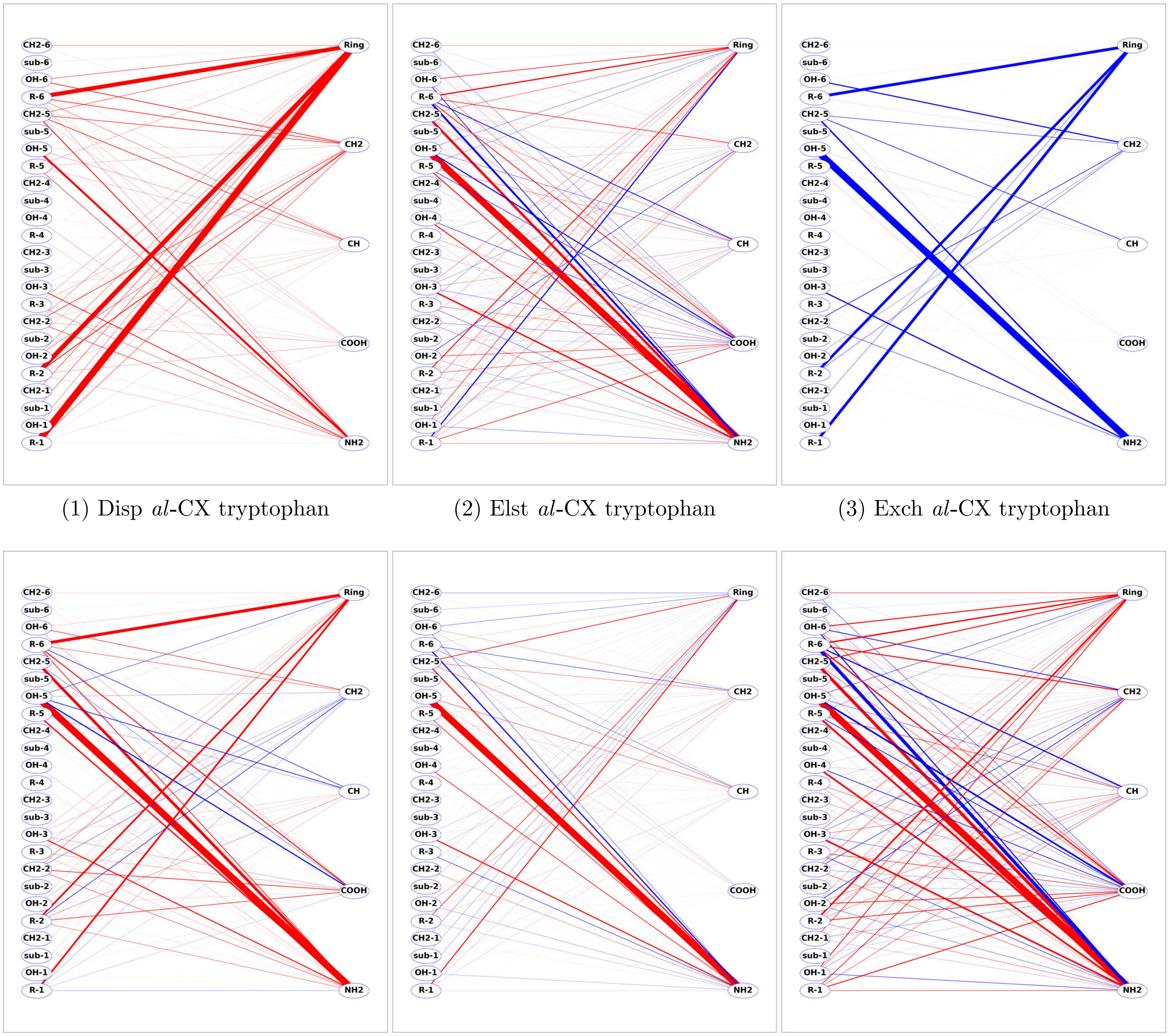

(1) Disp *al*-CX tryptophan (2) Elst *al*-CX tryptophan (3) Exch *al*-CX tryptophan

(4) IndAB *al*-CX tryptophan (5) IndBA *al*-CX tryptophan (6) Total *al*-CX tryptophan

Figure S23: The F-SAPT partitioning for *al*-CX tryptophan

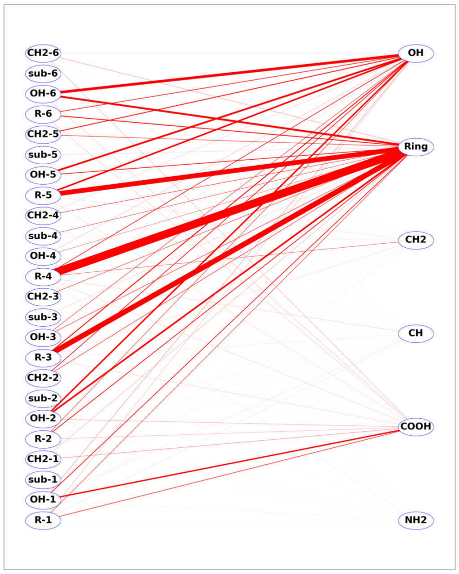

(1) Disp *al*-CX tyrosine

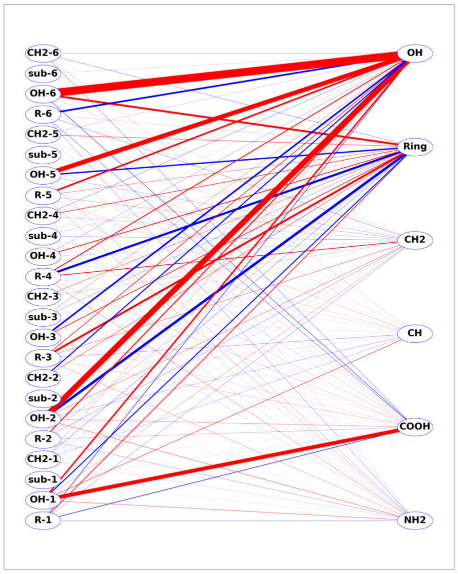

(2) Elst *al*-CX tyrosine

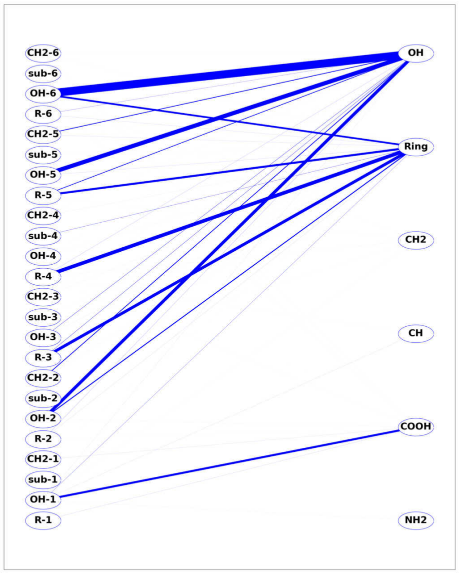

(3) Exch *al*-CX tyrosine

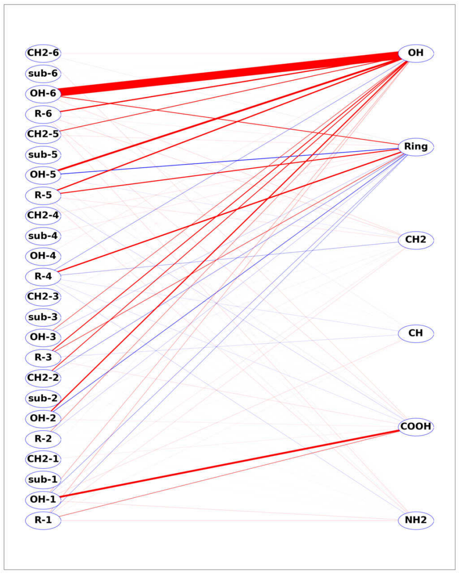

(4) IndAB *al*-CX tyrosine

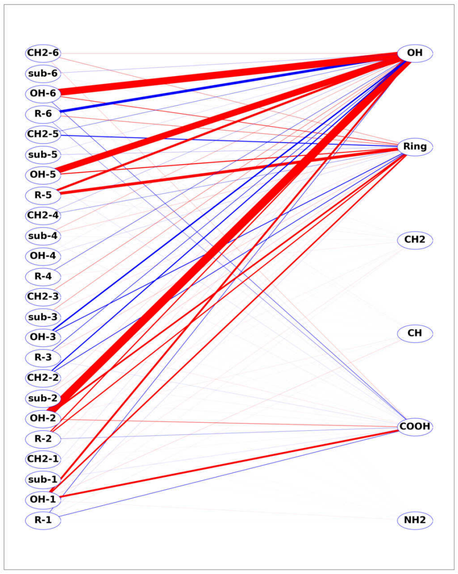

(5) IndBA *al*-CX tyrosine

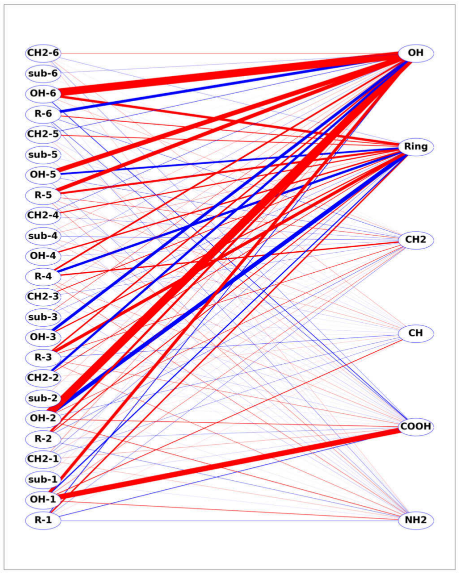

(6) Total *al*-CX tyrosine

Figure S24: The F-SAPT partitioning for *al*-CX tyrosine

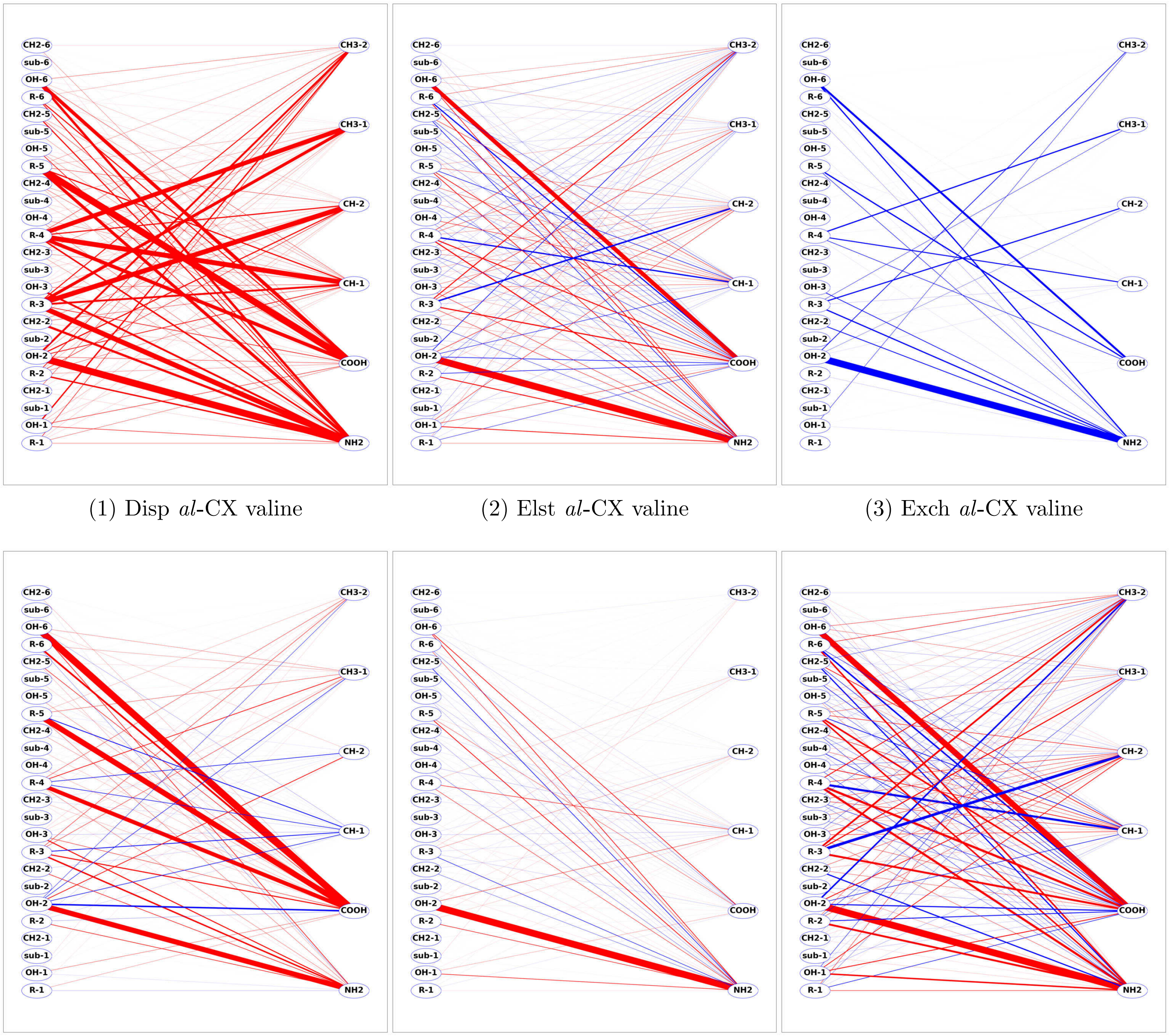

(1) Disp *al*-CX valine (2) Elst *al*-CX valine (3) Exch *al*-CX valine

(4) IndAB *al*-CX valine (5) IndBA *al*-CX valine (6) Total *al*-CX valine

Figure S25: The F-SAPT partitioning for *al*-CX valine

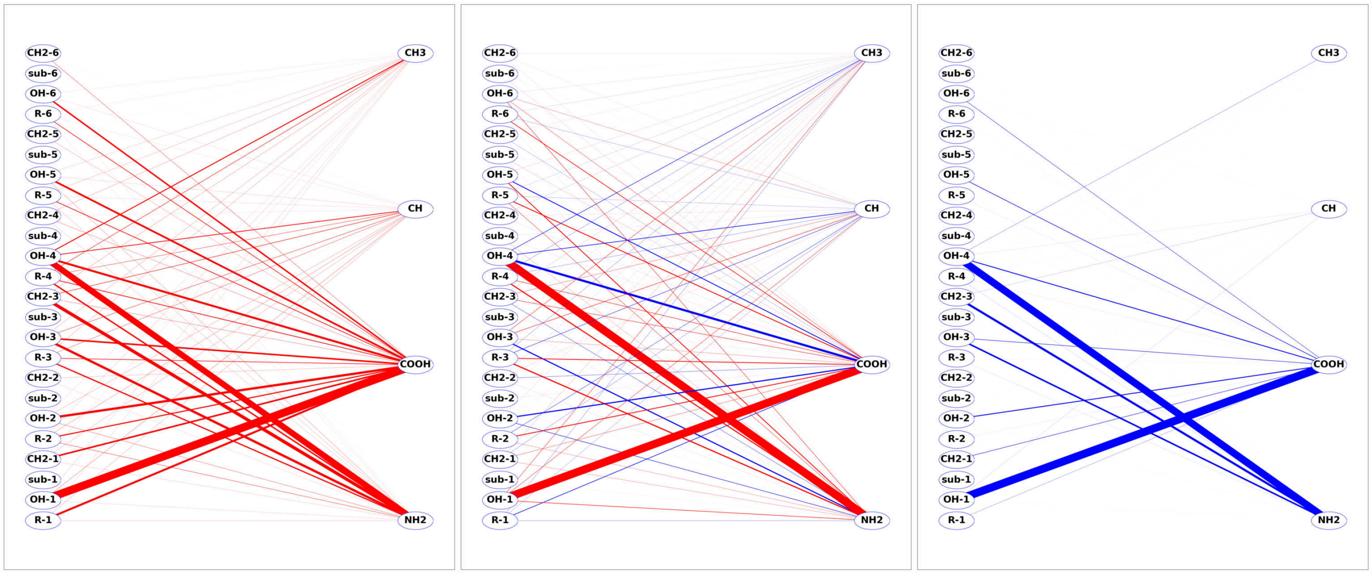

(1) Disp *pc*-CX alanine (2) Elst *pc*-CX alanine (3) Exch *pc*-CX alanine

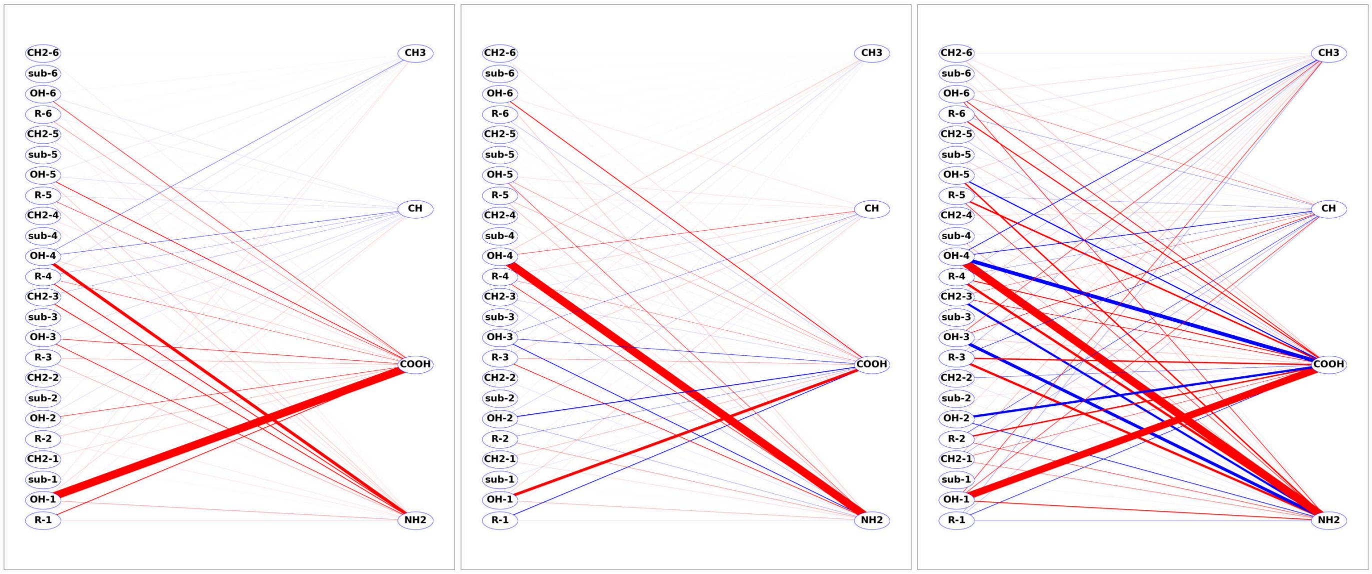

(4) IndAB *pc*-CX alanine (5) IndBA *pc*-CX alanine (6) Total *pc*-CX alanine

Figure S26: The F-SAPT partitioning for *pc*-CX alanine

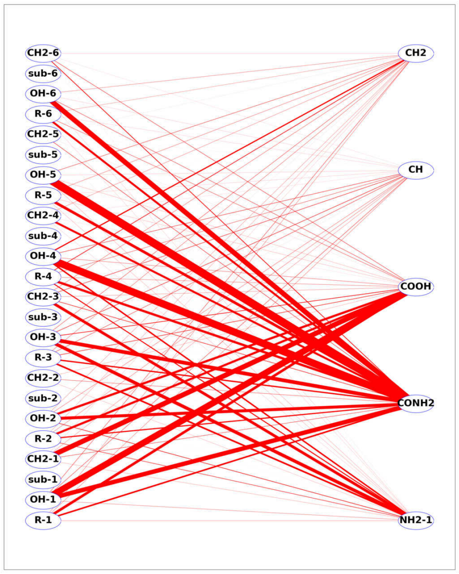

(1) Disp *pc*-CX asparagine

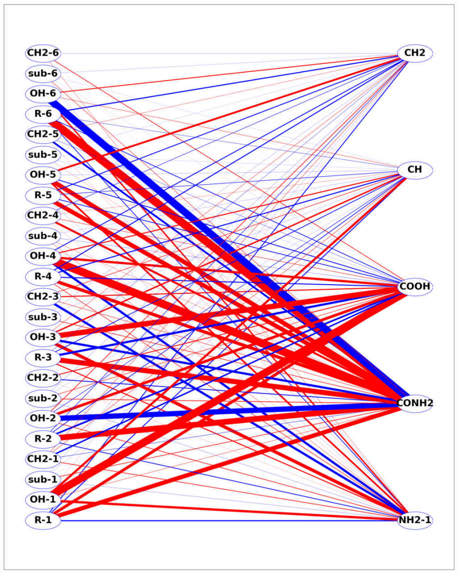

(2) Elst *pc*-CX asparagine

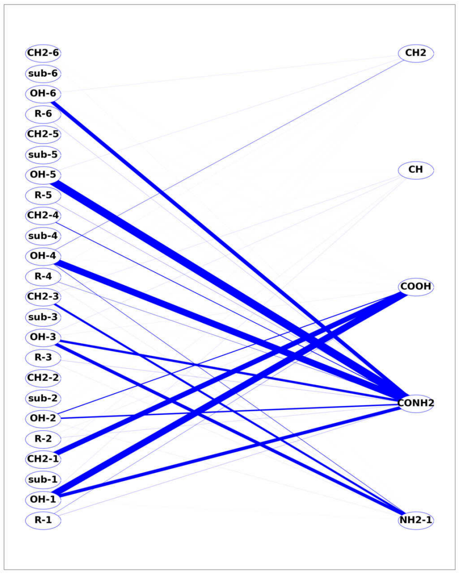

(3) Exch *pc*-CX asparagine

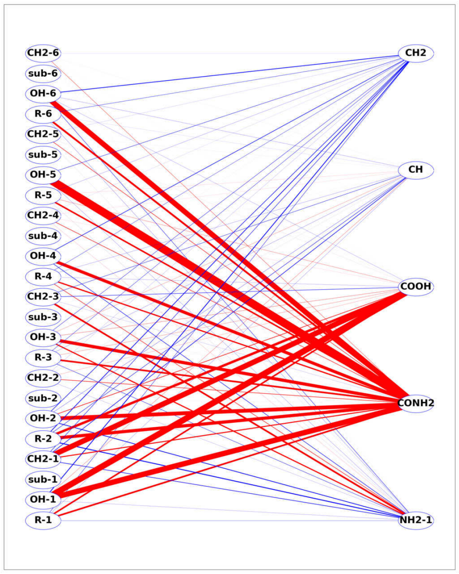

(4) IndAB *pc*-CX asparagine

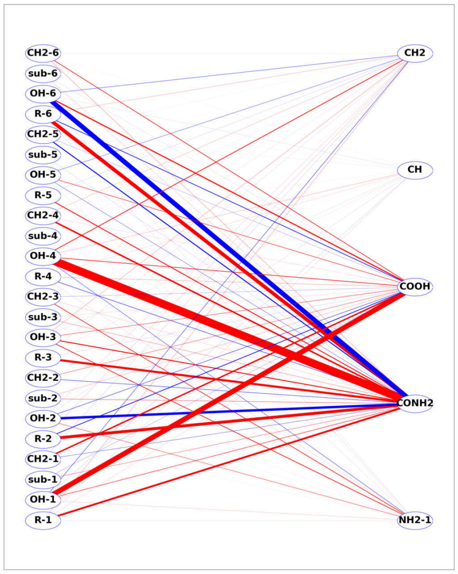

(5) IndBA *pc*-CX asparagine

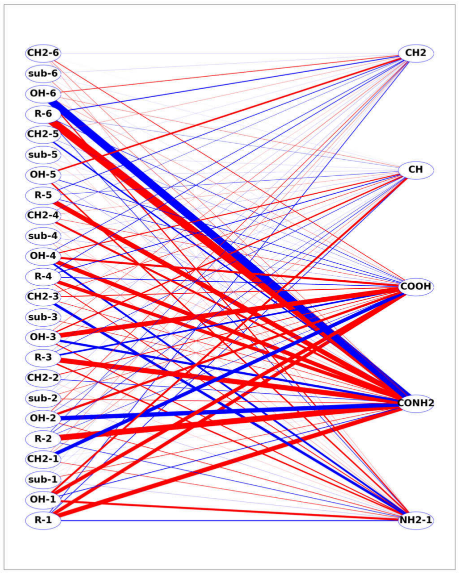

(6) Total *pc*-CX asparagine

Figure S27: The F-SAPT partitioning for *pc*-CX asparagine

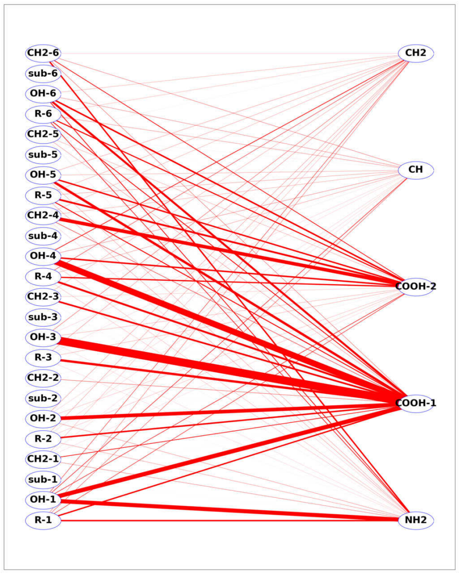

(1) Disp *pc*-CX aspartate

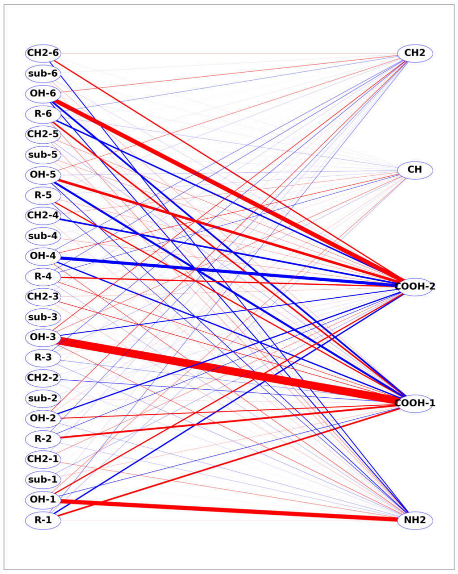

(2) Elst *pc*-CX aspartate

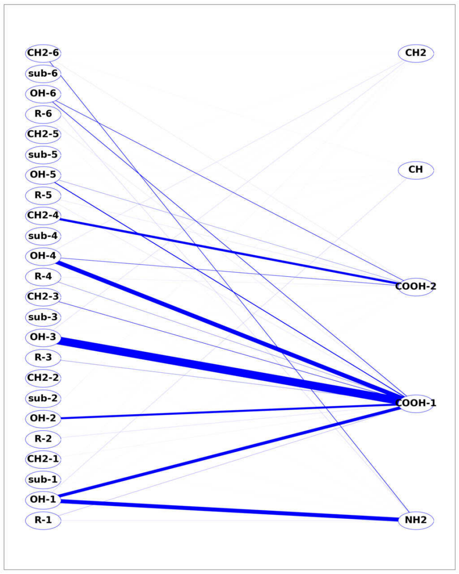

(3) Exch *pc*-CX aspartate

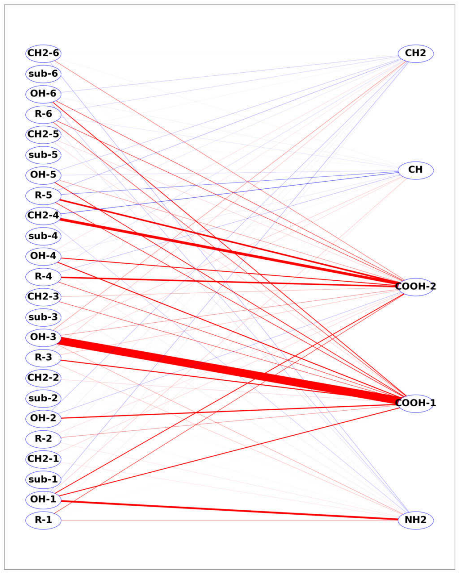

(4) IndAB *pc*-CX aspartate

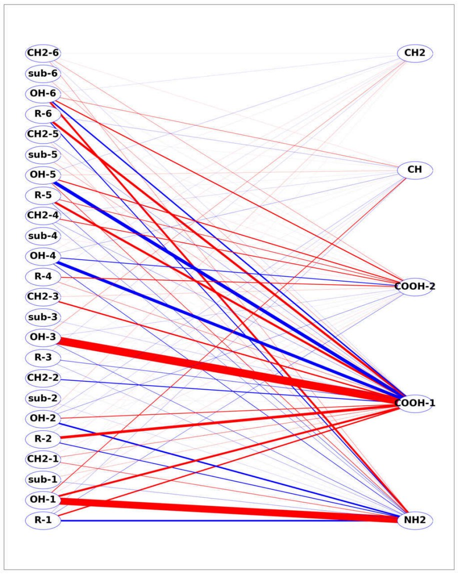

(5) IndBA *pc*-CX aspartate

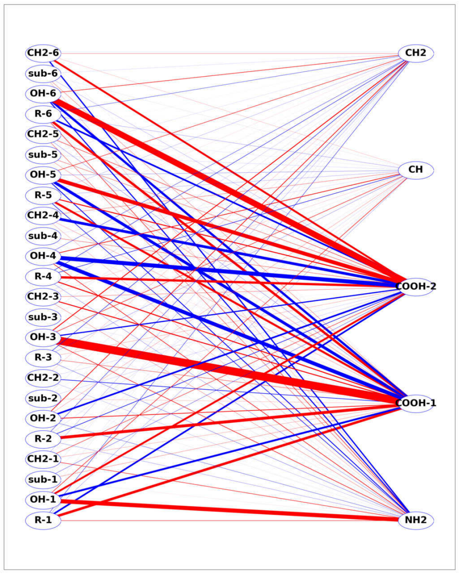

(6) Total *pc*-CX aspartate

Figure S28: The F-SAPT partitioning for *pc*-CX aspartate

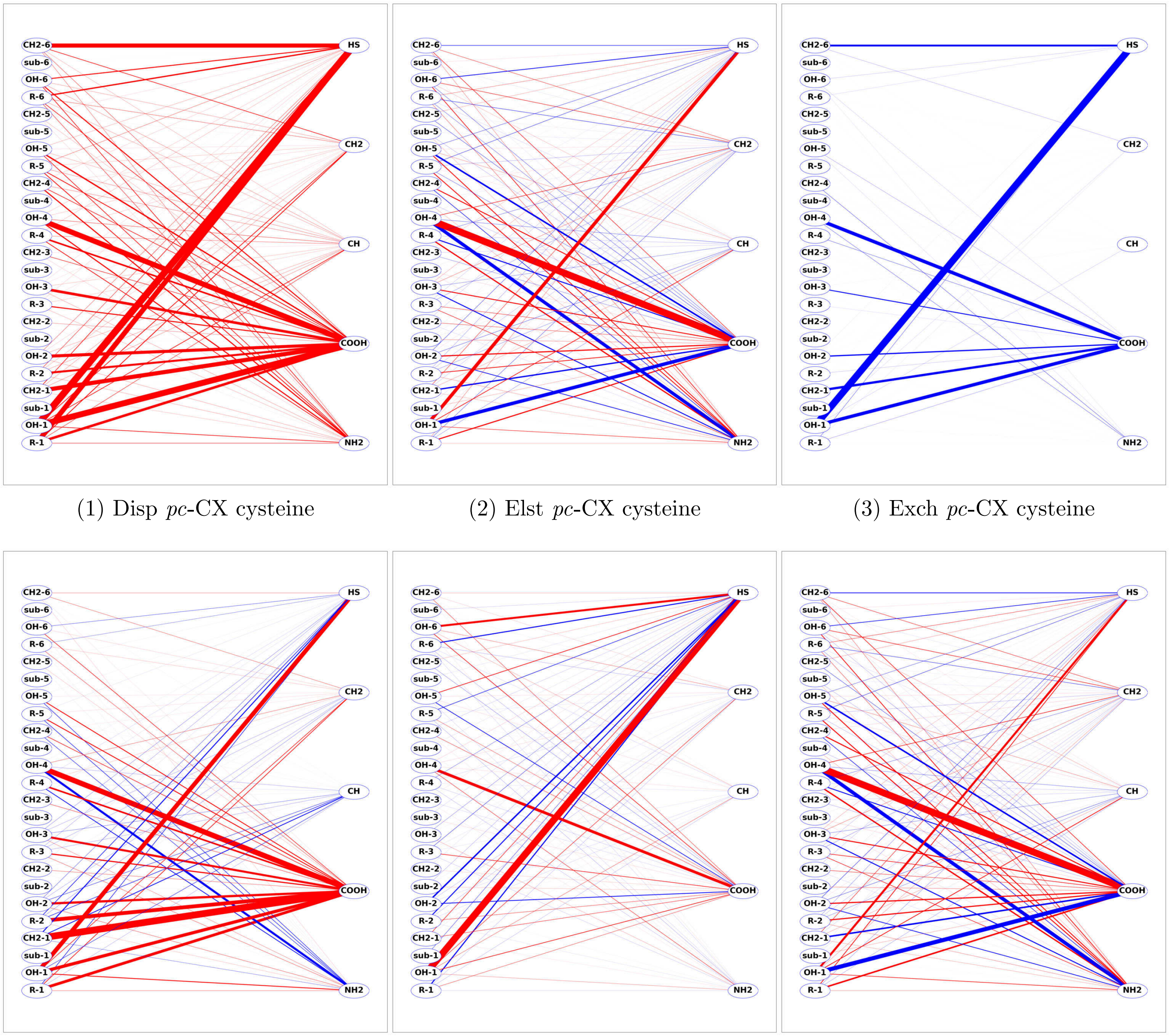

(1) Disp *pc*-CX cysteine

(2) Elst *pc*-CX cysteine

(3) Exch *pc*-CX cysteine

(4) IndAB *pc*-CX cysteine

(5) IndBA *pc*-CX cysteine

(6) Total *pc*-CX cysteine

Figure S29: The F-SAPT partitioning for *pc*-CX cysteine

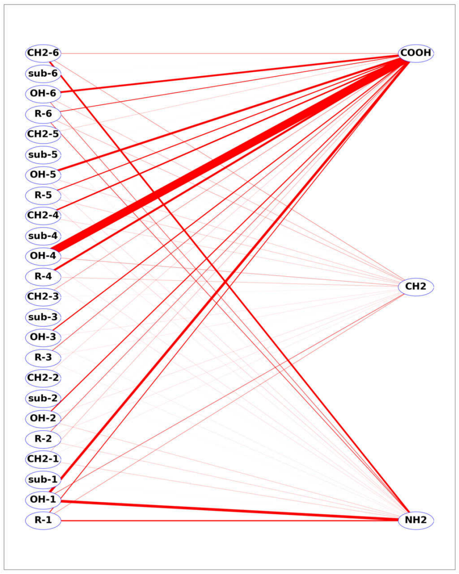

(1) Disp *pc*-CX glycine

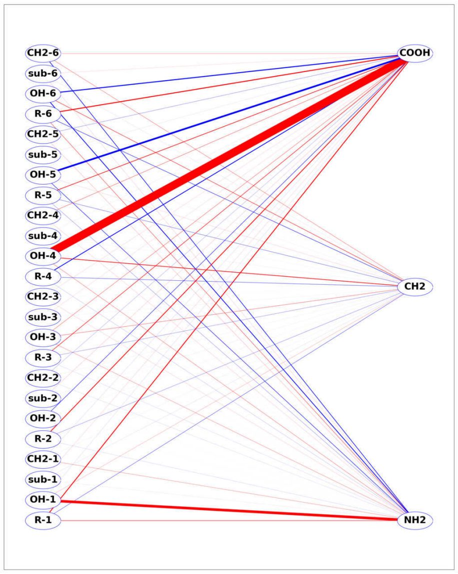

(2) Elst *pc*-CX glycine

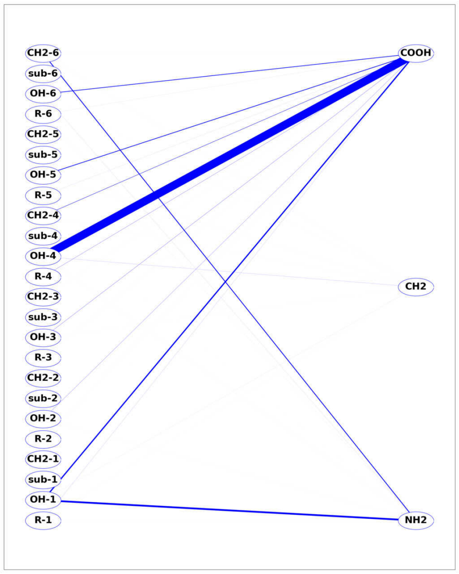

(3) Exch *pc*-CX glycine

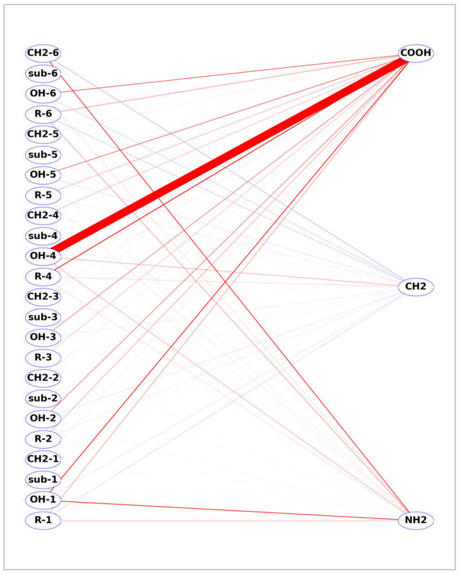

(4) IndAB *pc*-CX glycine

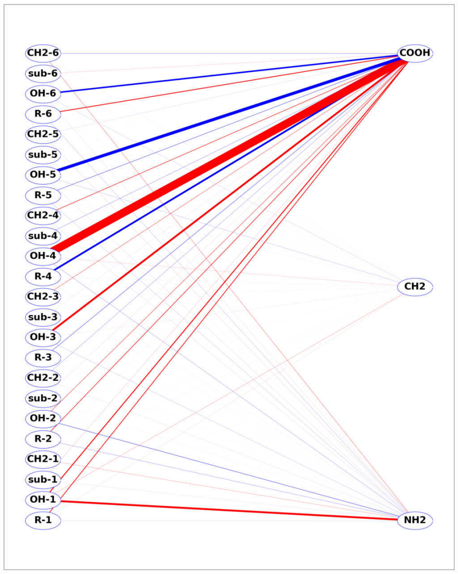

(5) IndBA *pc*-CX glycine

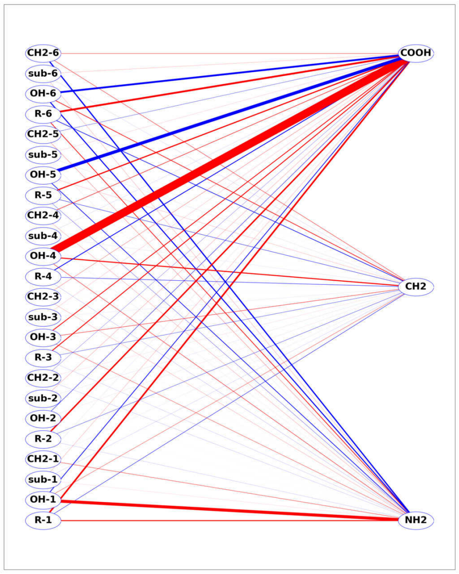

(6) Total *pc*-CX glycine

Figure S30: The F-SAPT partitioning for *pc*-CX glycine

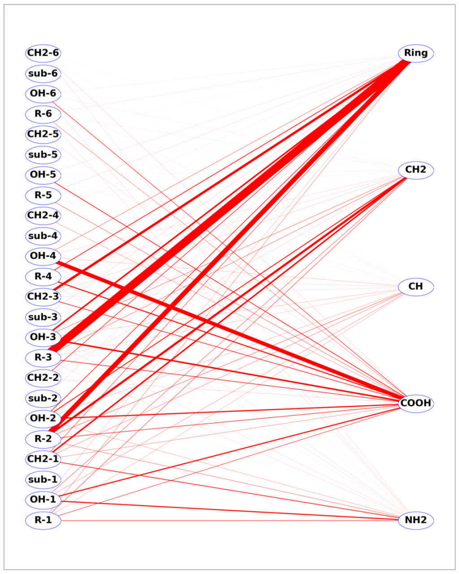

(1) Disp *pc*-CX histidineD

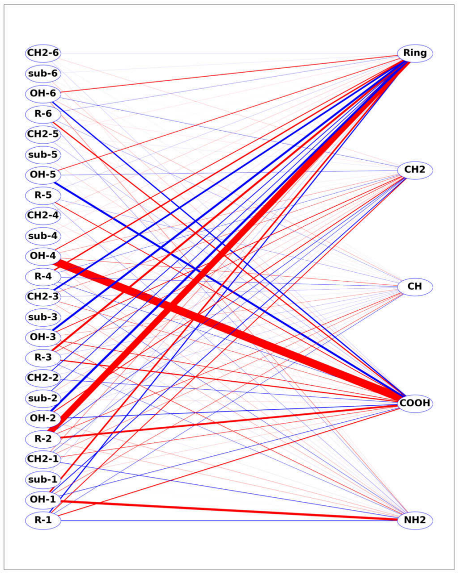

(2) Elst *pc*-CX histidineD

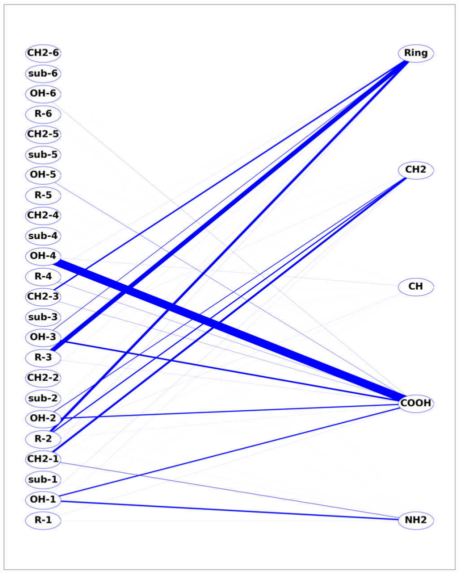

(3) Exch *pc*-CX histidineD

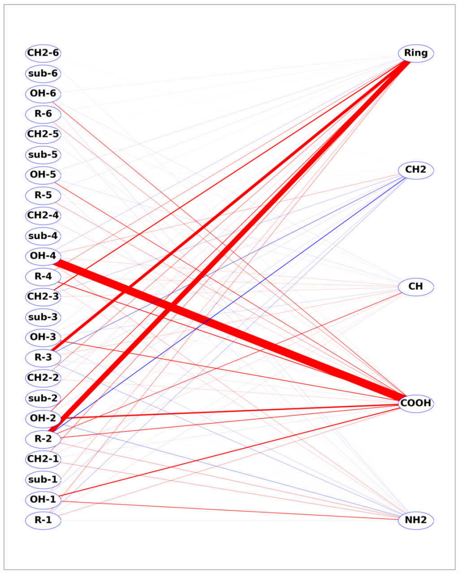

(4) IndAB *pc*-CX histidineD

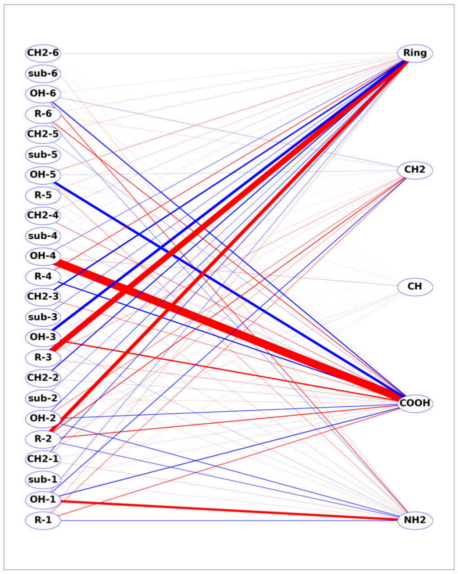

(5) IndBA *pc*-CX histidineD

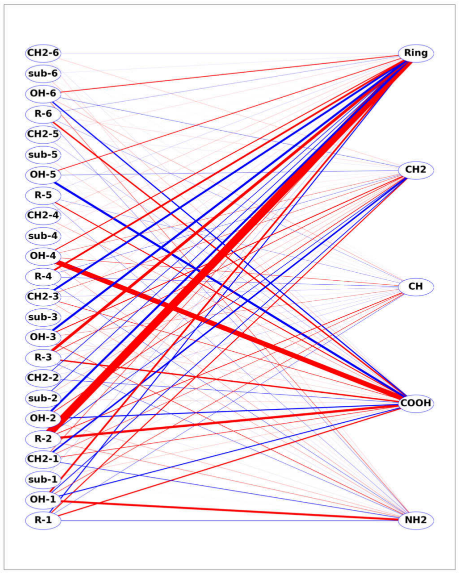

(6) Total *pc*-CX histidineD

Figure S31: The F-SAPT partitioning for *pc*-CX histidineD

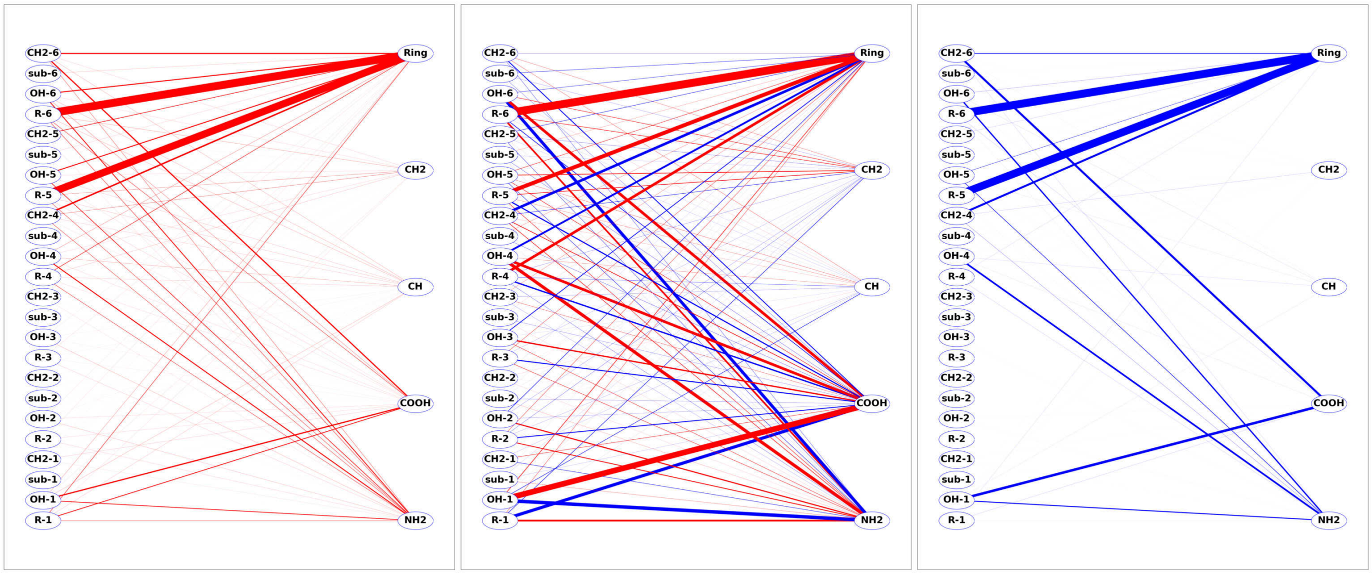

(1) Disp *pc*-CX histidineE (2) Elst *pc*-CX histidineE (3) Exch *pc*-CX histidineE

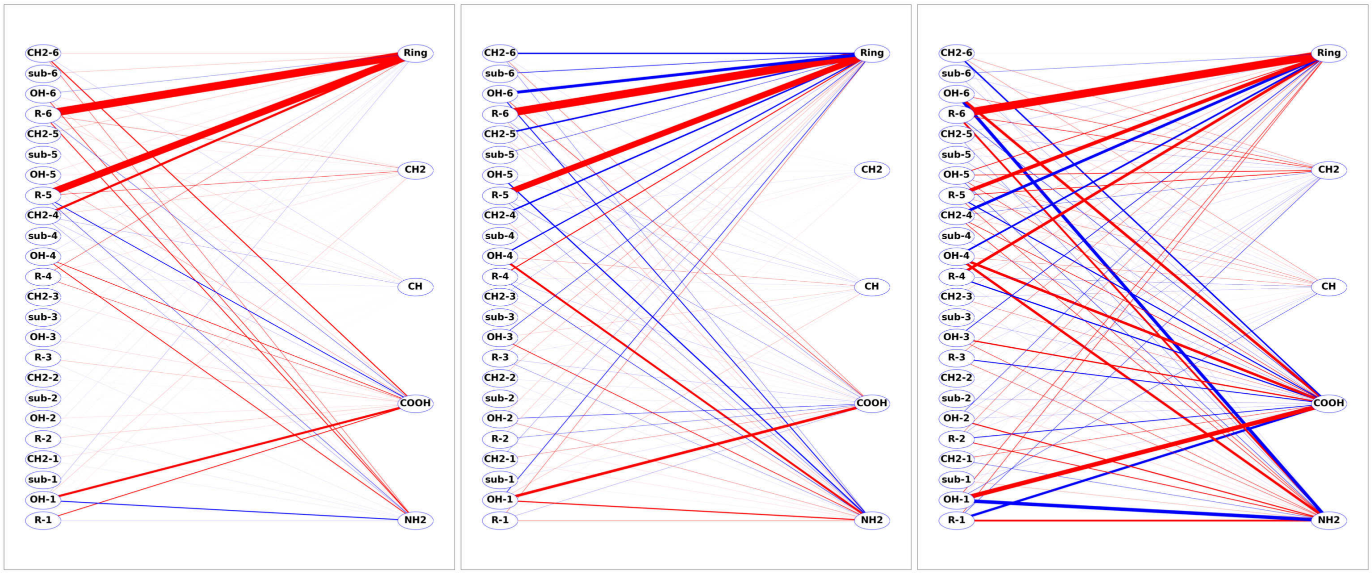

(4) IndAB *pc*-CX histidineE (5) IndBA *pc*-CX histidineE (6) Total *pc*-CX histidineE

Figure S32: The F-SAPT partitioning for *pc*-CX histidineE

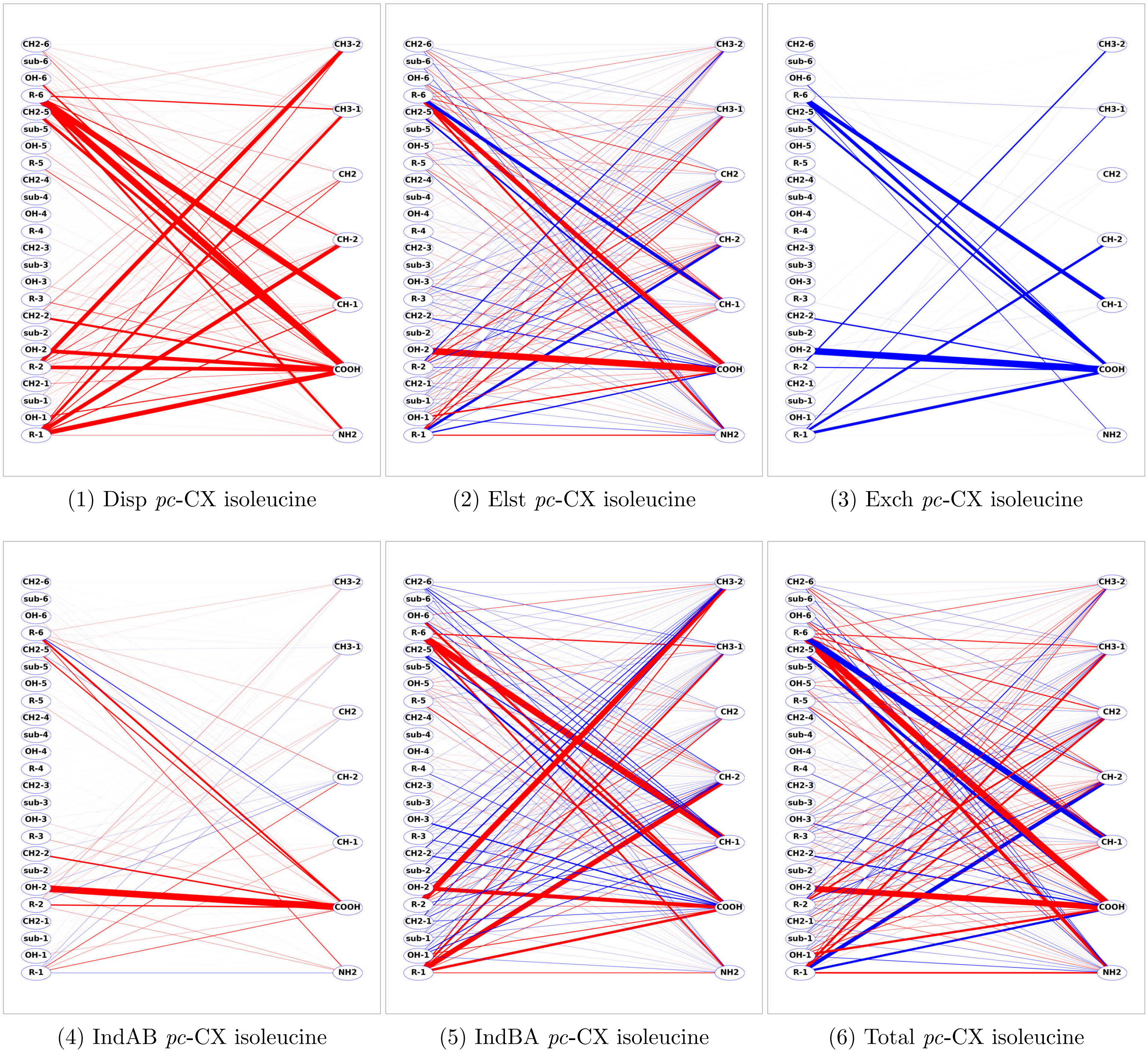

(1) Disp *pc*-CX isoleucine

(2) Elst *pc*-CX isoleucine

(3) Exch *pc*-CX isoleucine

(4) IndAB *pc*-CX isoleucine

(5) IndBA *pc*-CX isoleucine

(6) Total *pc*-CX isoleucine

Figure S33: The F-SAPT partitioning for *pc*-CX isoleucine

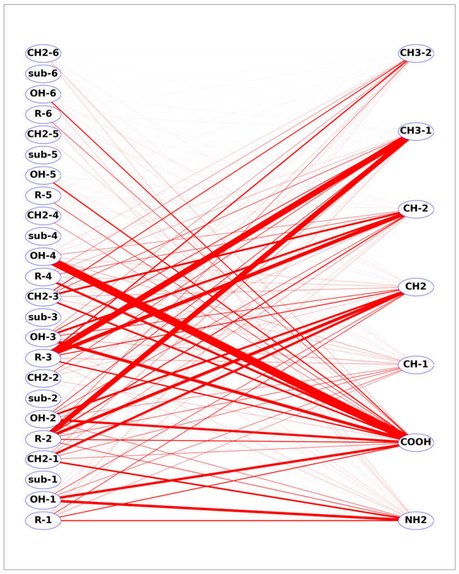

(1) Disp *pc*-CX leucine

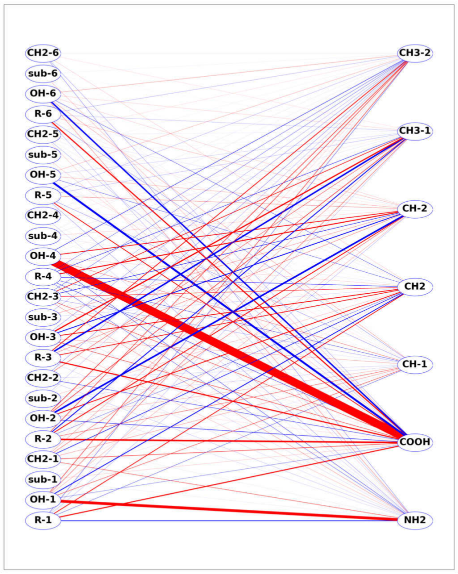

(2) Elst *pc*-CX leucine

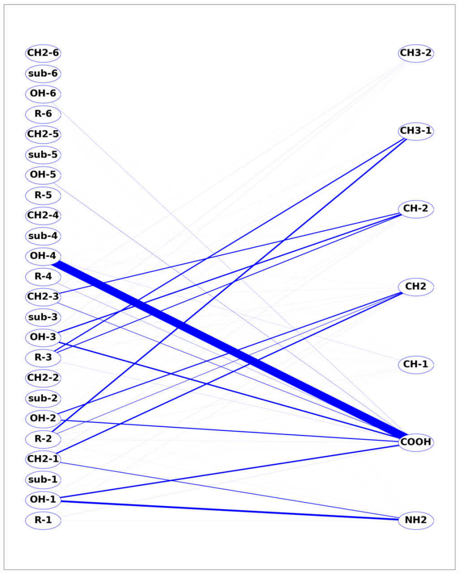

(3) Exch *pc*-CX leucine

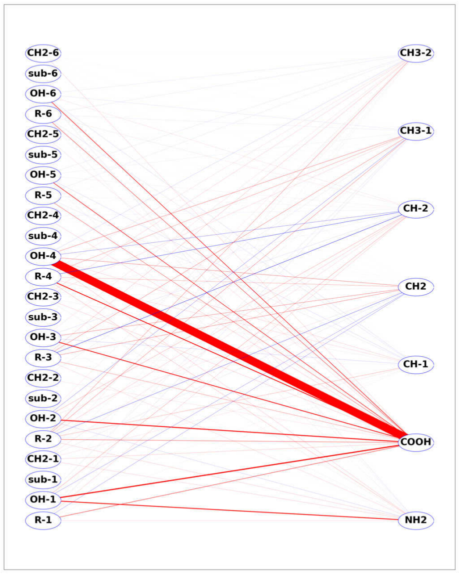

(4) IndAB *pc*-CX leucine

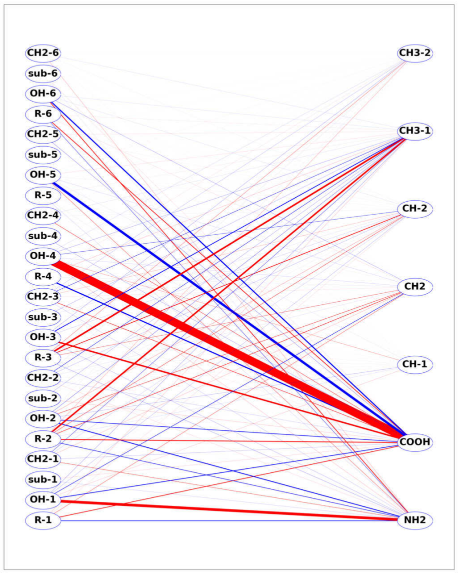

(5) IndBA *pc*-CX leucine

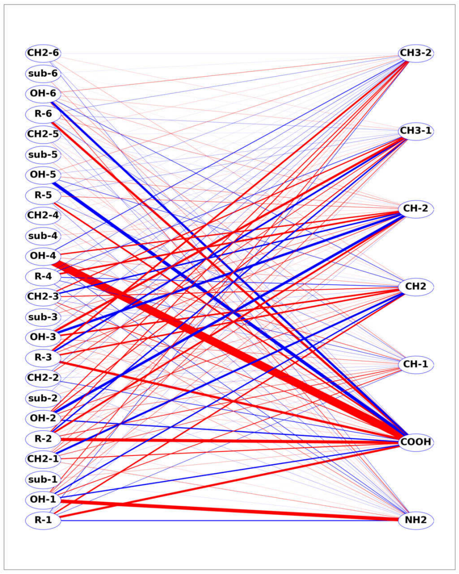

(6) Total *pc*-CX leucine

Figure S34: The F-SAPT partitioning for *pc*-CX leucine

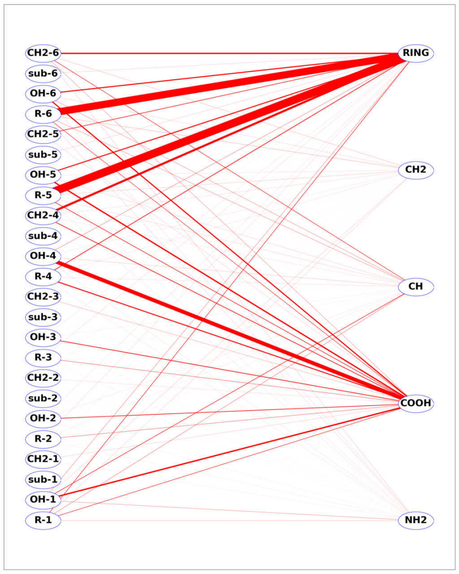

(1) Disp *pc*-CX phenylalanine

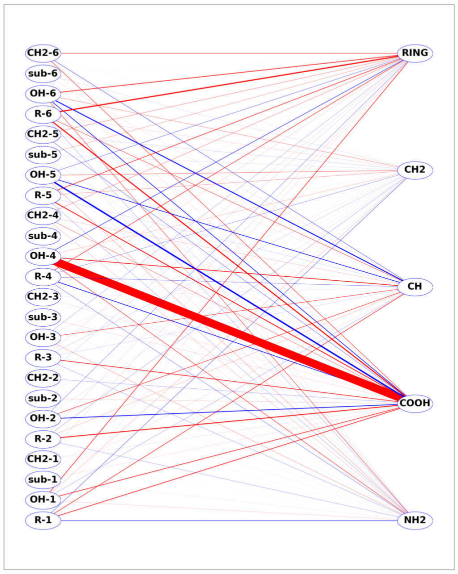

(2) Elst *pc*-CX phenylalanine

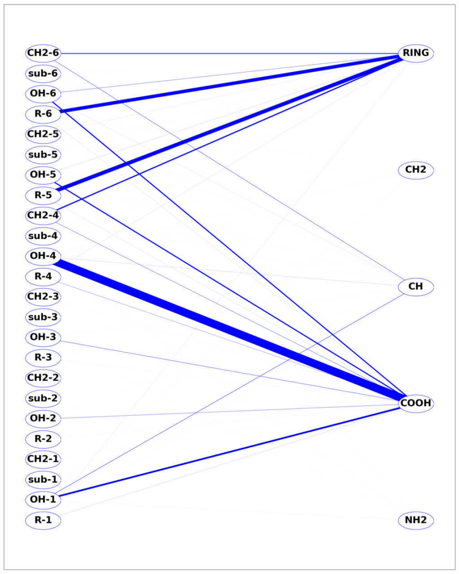

(3) Exch *pc*-CX phenylalanine

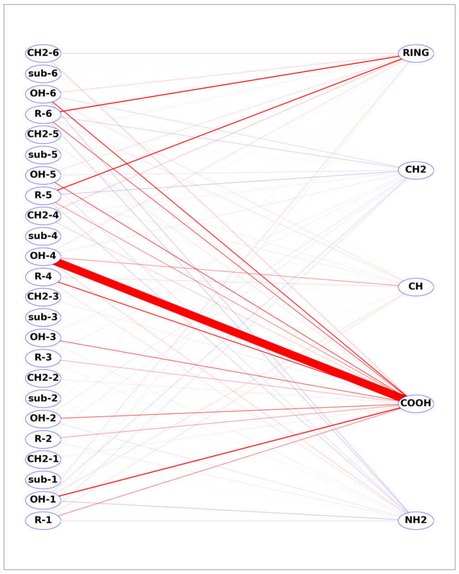

(4) IndAB *pc*-CX phenylalanine

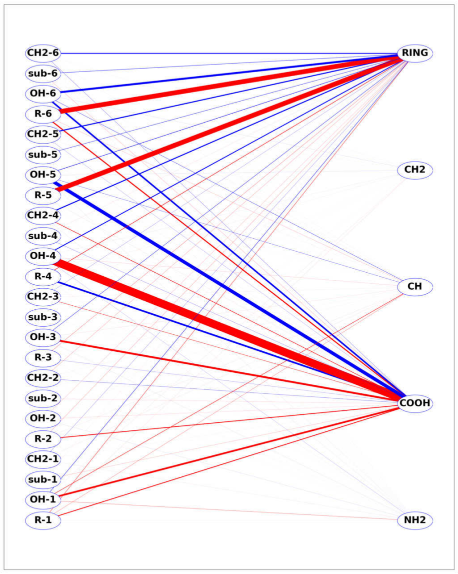

(5) IndBA *pc*-CX phenylalanine

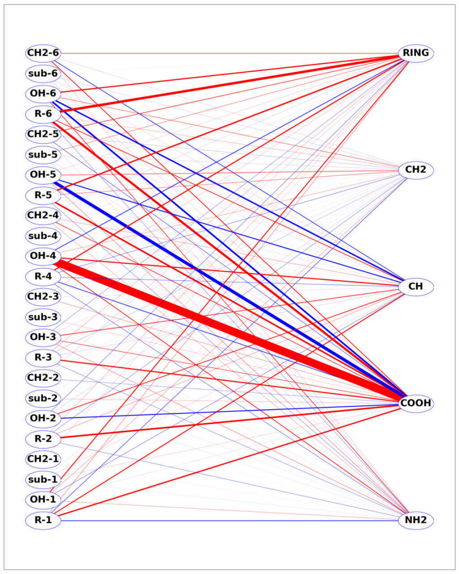

(6) Total *pc*-CX phenylalanine

Figure S35: The F-SAPT partitioning for *pc*-CX phenylalanine

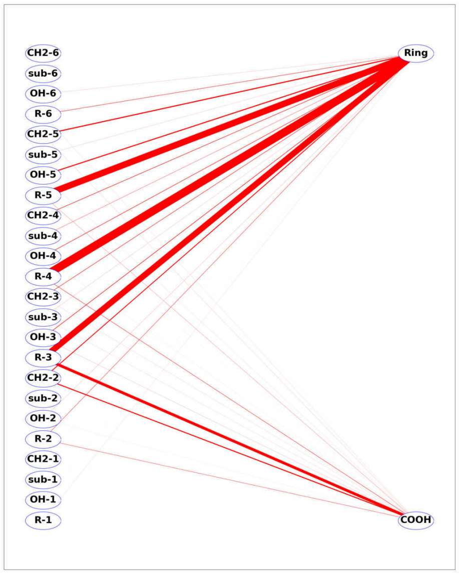

(1) Disp *pc*-CX proline

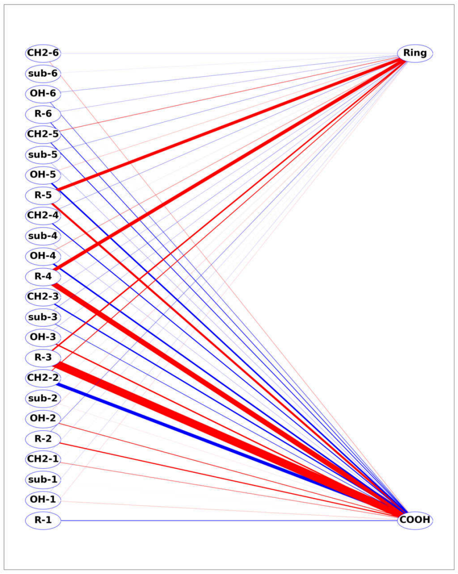

(2) Elst *pc*-CX proline

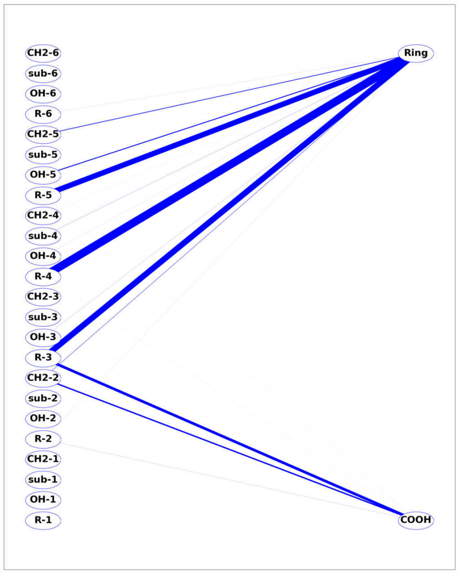

(3) Exch *pc*-CX proline

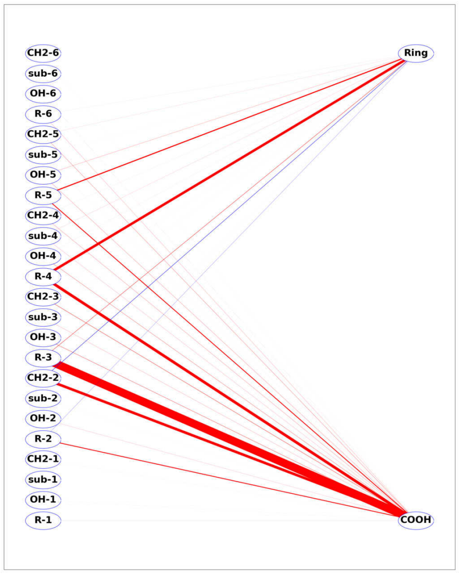

(4) IndAB *pc*-CX proline

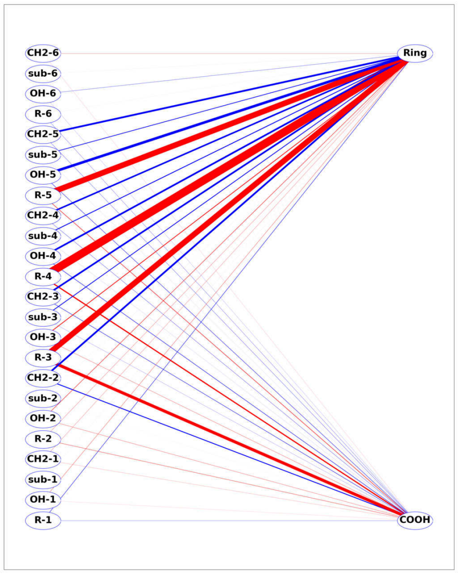

(5) IndBA *pc*-CX proline

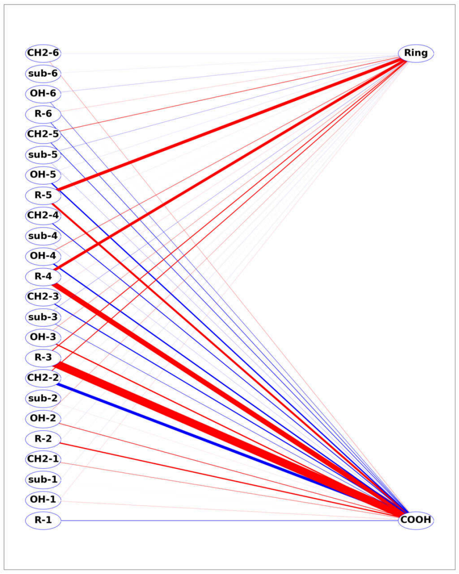

(6) Total *pc*-CX proline

Figure S36: The F-SAPT partitioning for *pc*-CX proline

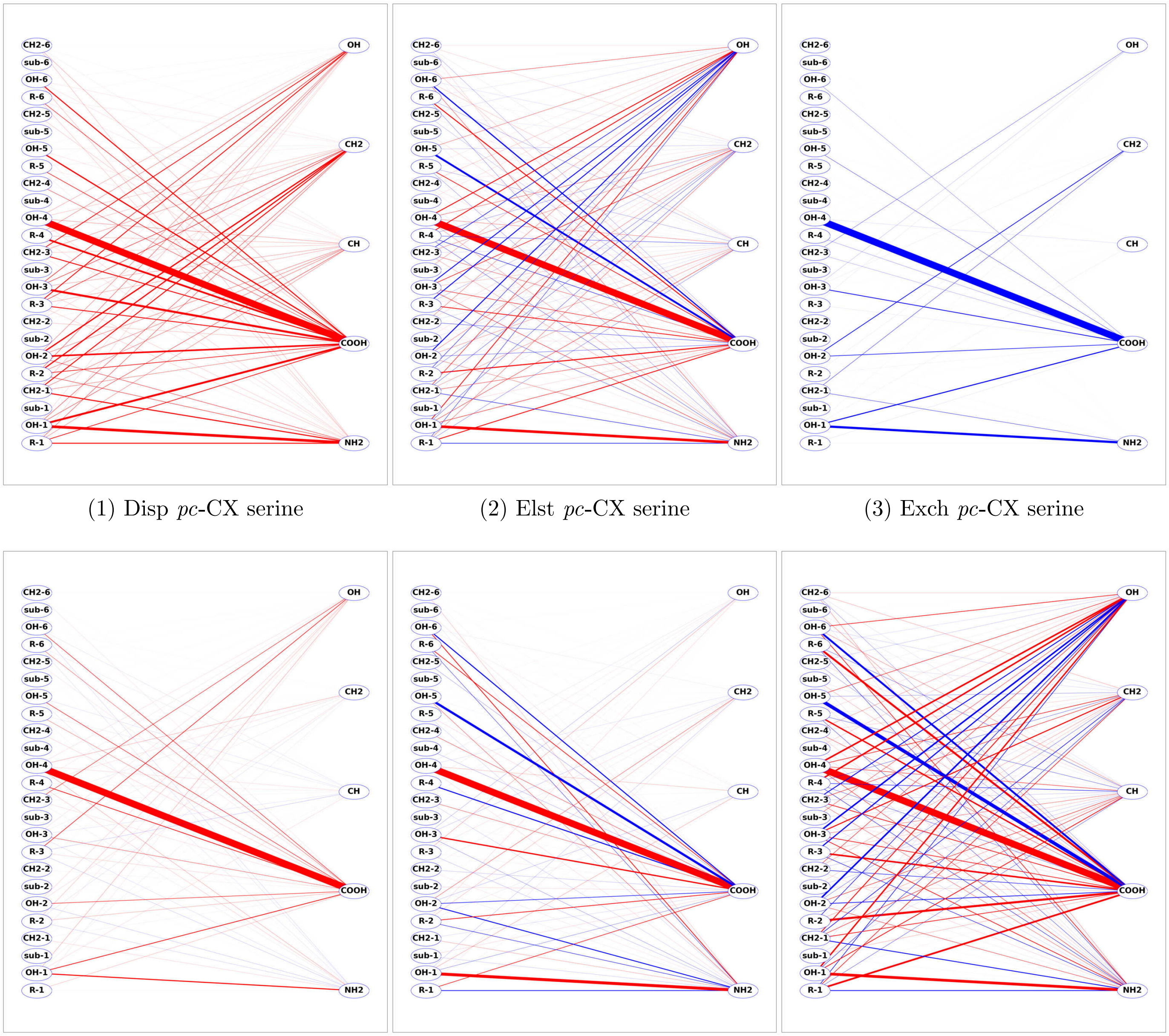

(1) Disp *pc*-CX serine

(2) Elst *pc*-CX serine

(3) Exch *pc*-CX serine

(4) IndAB *pc*-CX serine

(5) IndBA *pc*-CX serine

(6) Total *pc*-CX serine

Figure S37: The F-SAPT partitioning for *pc*-CX serine

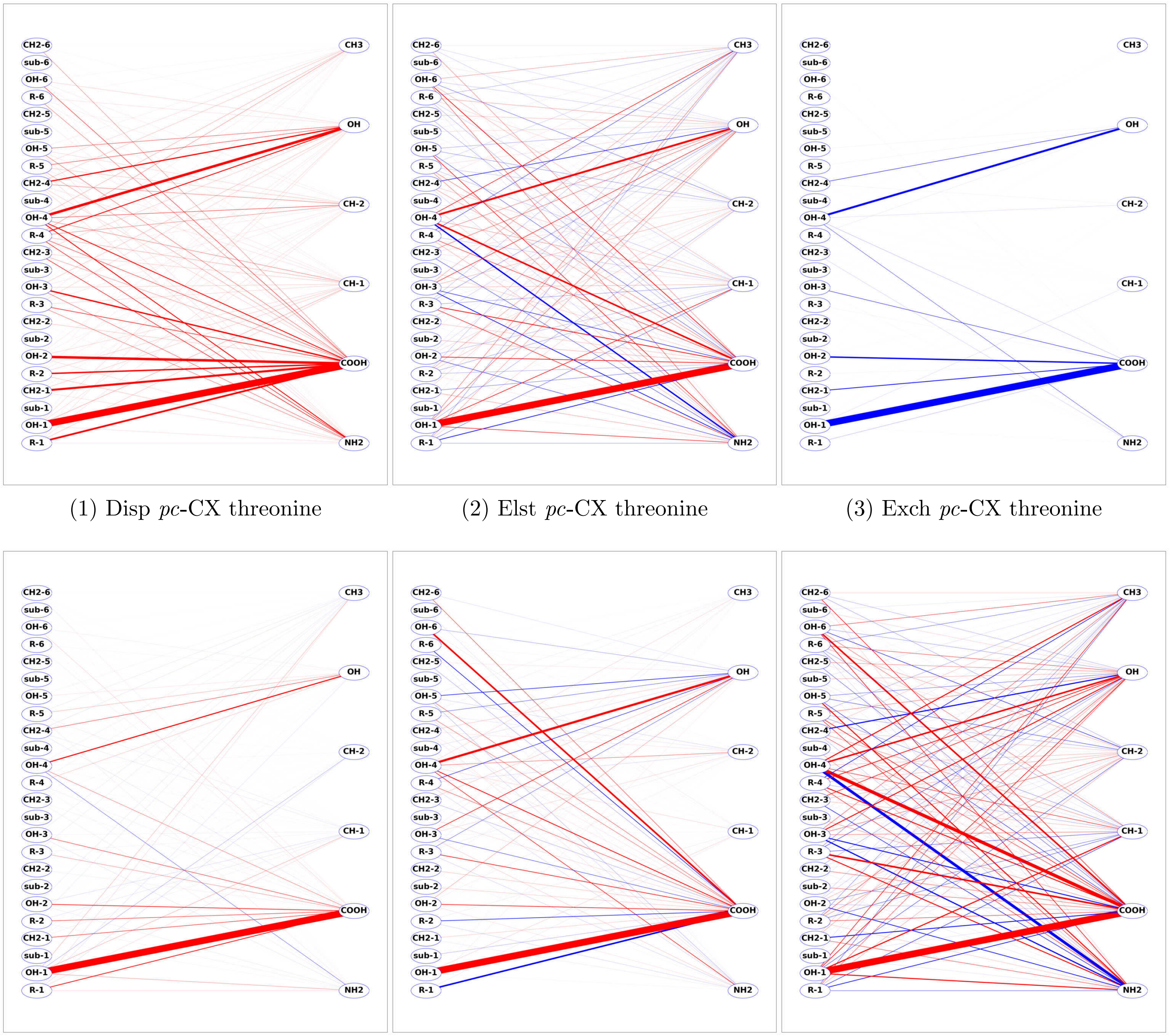

(1) Disp *pc*-CX threonine (2) Elst *pc*-CX threonine (3) Exch *pc*-CX threonine

(4) IndAB *pc*-CX threonine (5) IndBA *pc*-CX threonine (6) Total *pc*-CX threonine

Figure S38: The F-SAPT partitioning for *pc*-CX threonine

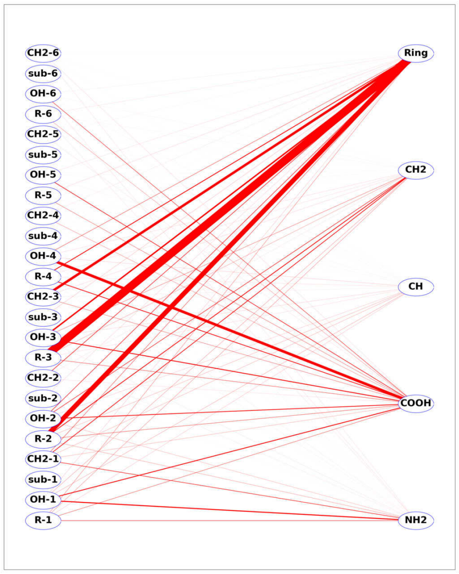

(1) Disp *pc*-CX tryptophan

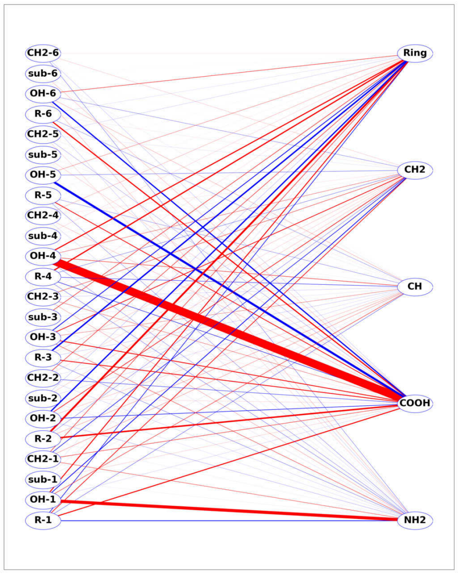

(2) Elst *pc*-CX tryptophan

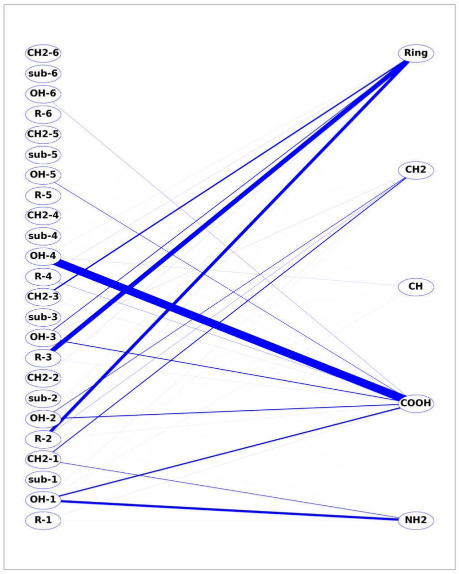

(3) Exch *pc*-CX tryptophan

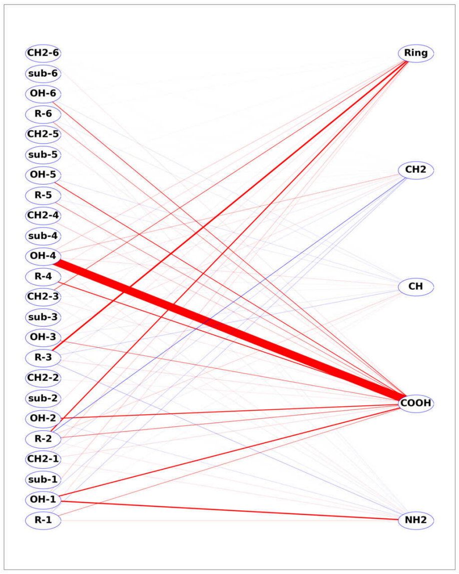

(4) IndAB *pc*-CX tryptophan

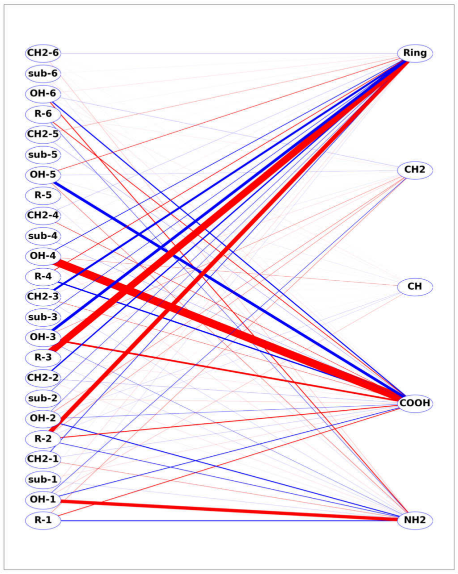

(5) IndBA *pc*-CX tryptophan

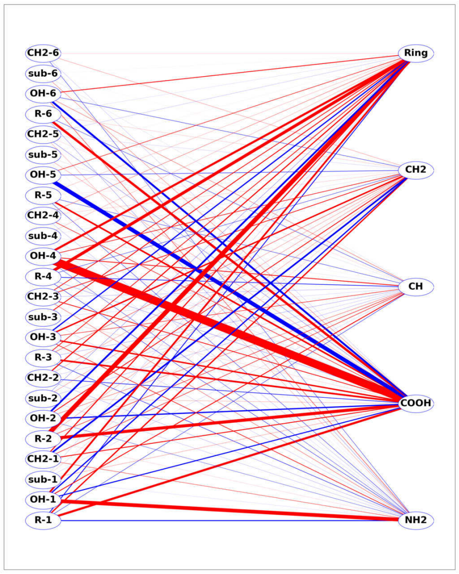

(6) Total *pc*-CX tryptophan

Figure S39: The F-SAPT partitioning for *pc*-CX tryptophan

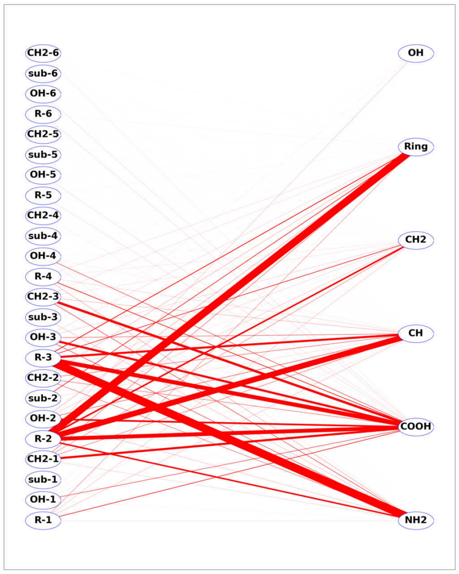

(1) Disp *pc*-CX tyrosine

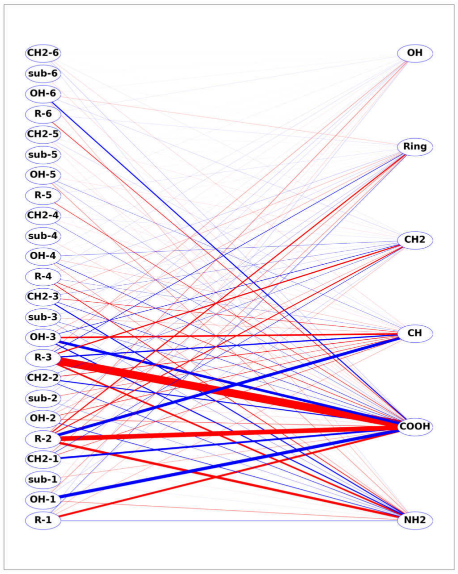

(2) Elst *pc*-CX tyrosine

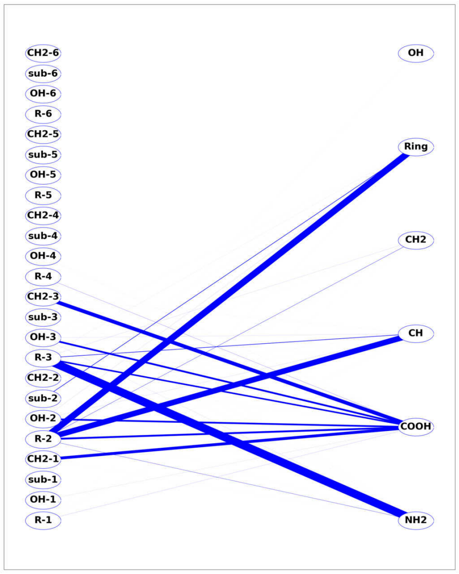

(3) Exch *pc*-CX tyrosine

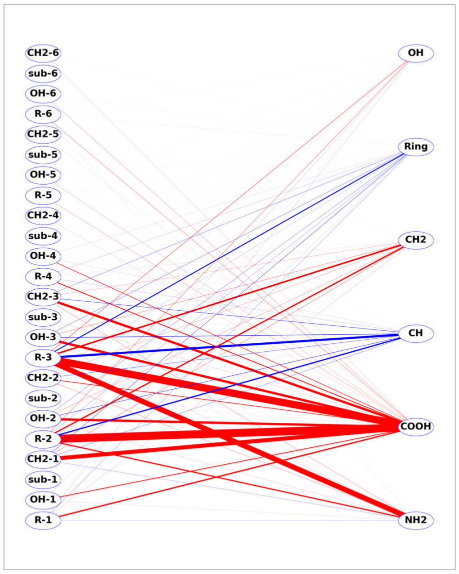

(4) IndAB *pc*-CX tyrosine

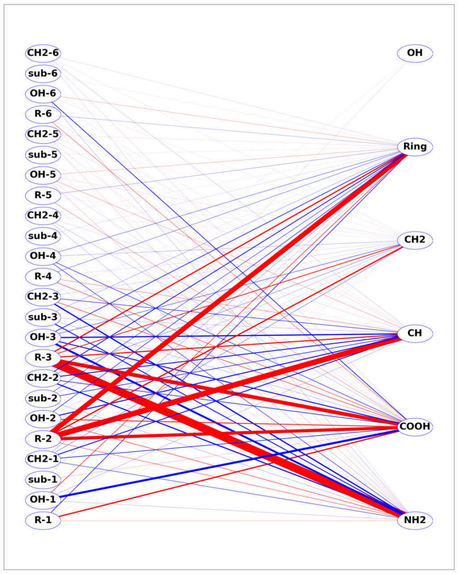

(5) IndBA *pc*-CX tyrosine

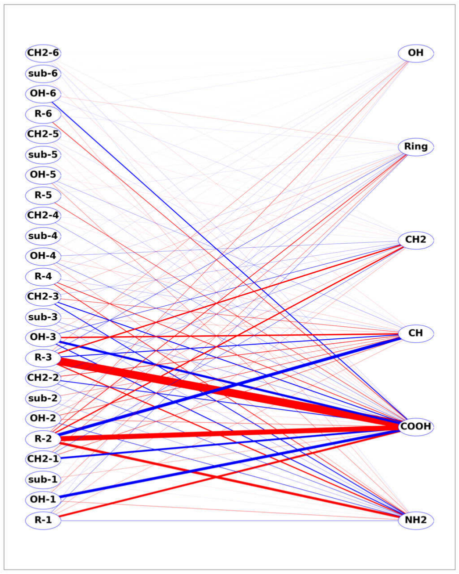

(6) Total *pc*-CX tyrosine

Figure S40: The F-SAPT partitioning for *pc*-CX tyrosine

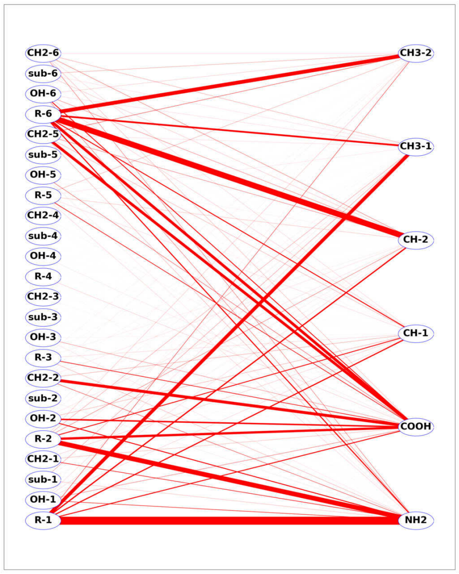

(1) Disp *pc*-CX valine

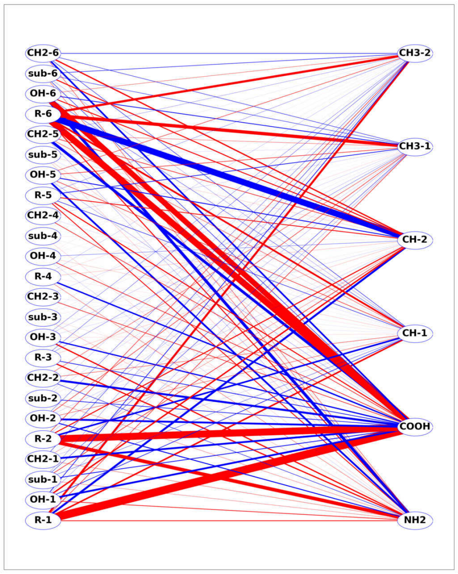

(2) Elst *pc*-CX valine

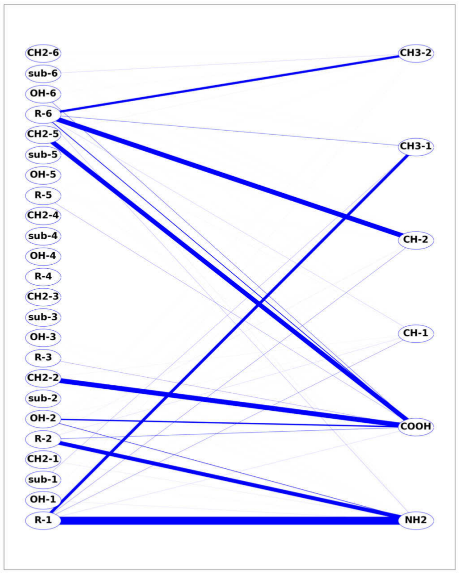

(3) Exch *pc*-CX valine

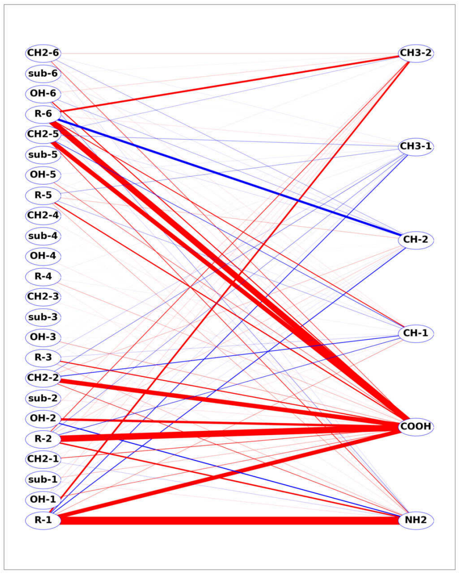

(4) IndAB *pc*-CX valine

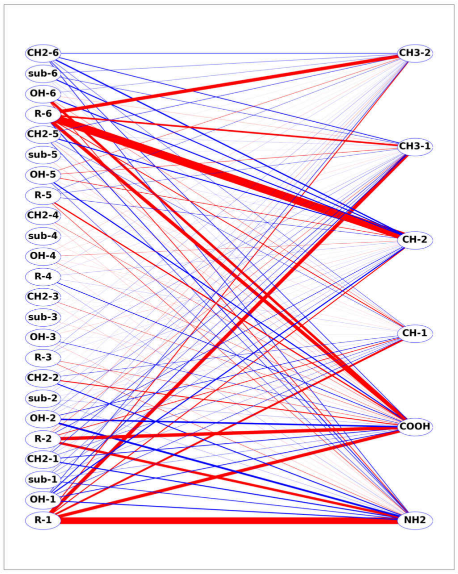

(5) IndBA *pc*-CX valine

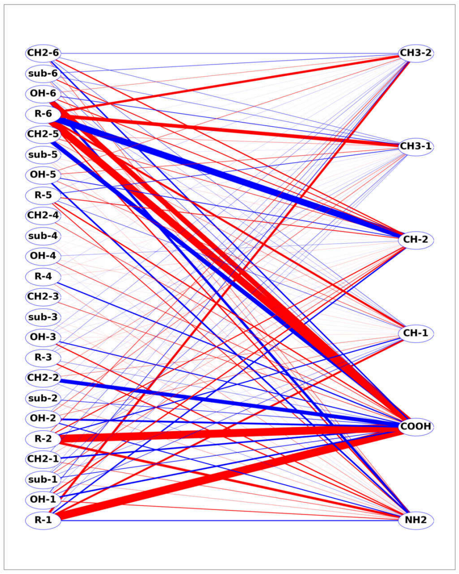

(6) Total *pc*-CX valine

Figure S41: The F-SAPT partitioning for *pc*-CX valine

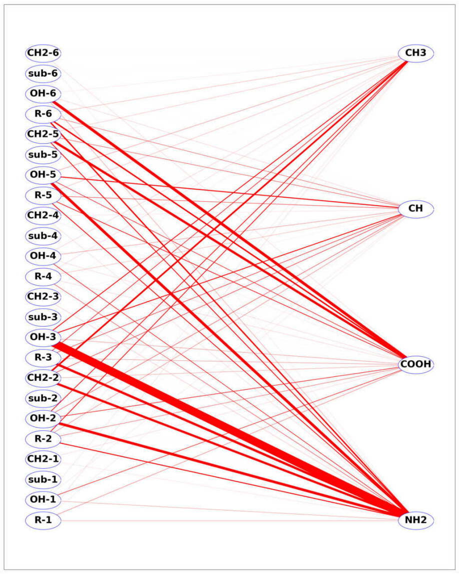

(1) Disp *wc*-CX alanine

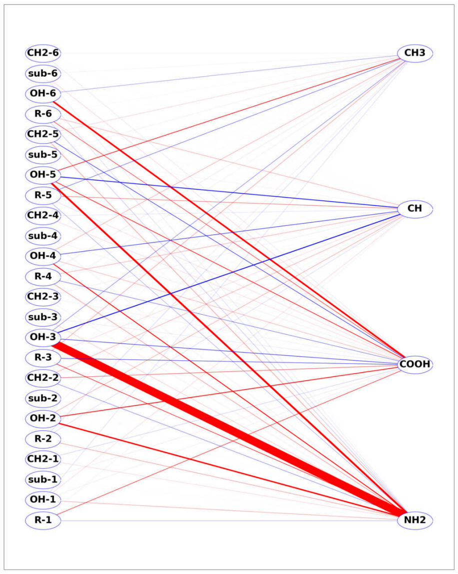

(2) Elst *wc*-CX alanine

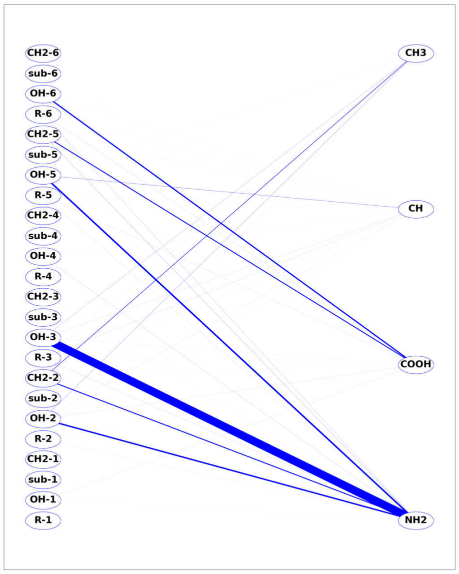

(3) Exch *wc*-CX alanine

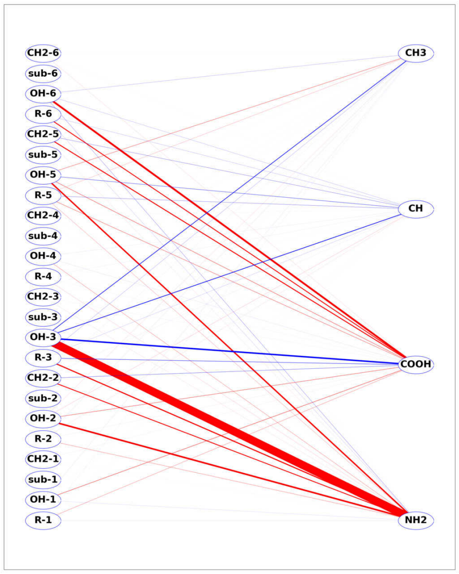

(4) IndAB *wc*-CX alanine

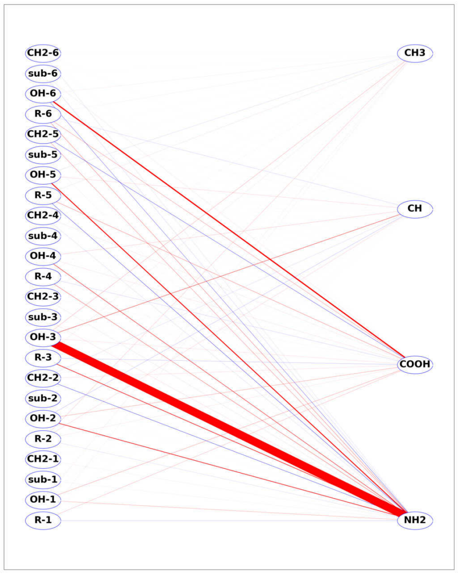

(5) IndBA *wc*-CX alanine

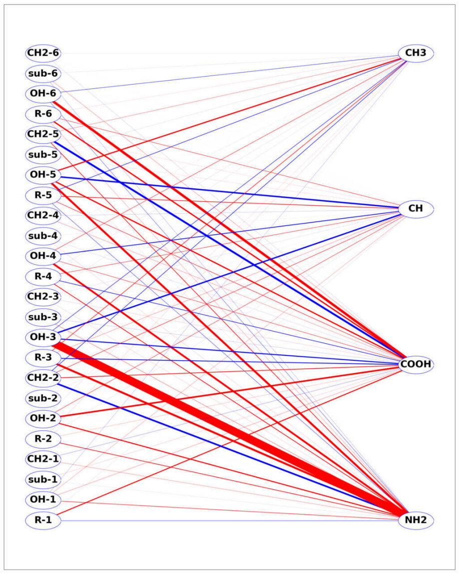

(6) Total *wc*-CX alanine

Figure S42: The F-SAPT partitioning for *wc*-CX alanine

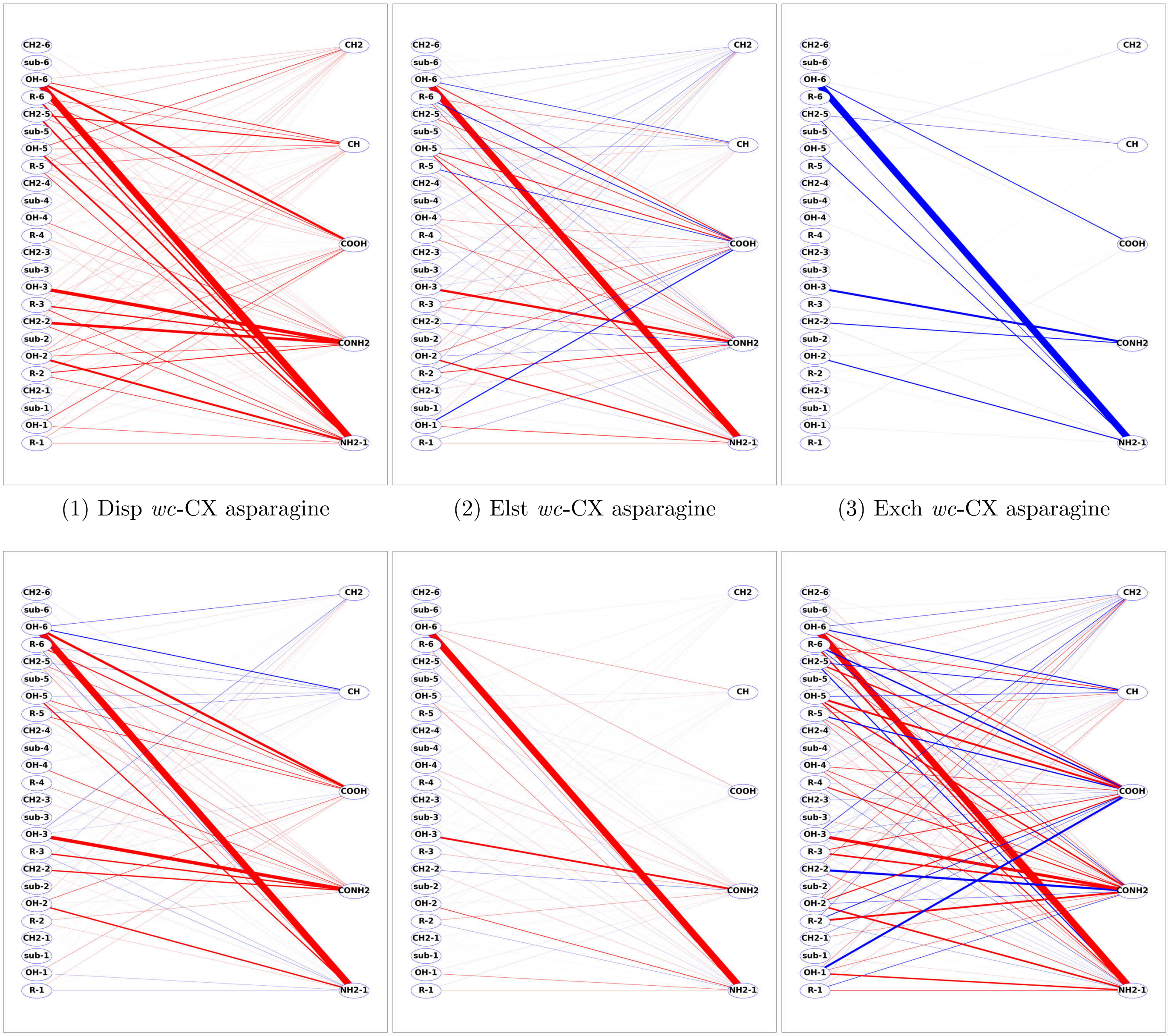

(1) Disp *wc*-CX asparagine (2) Elst *wc*-CX asparagine (3) Exch *wc*-CX asparagine

(4) IndAB *wc*-CX asparagine (5) IndBA *wc*-CX asparagine (6) Total *wc*-CX asparagine

Figure S43: The F-SAPT partitioning for *wc*-CX asparagine

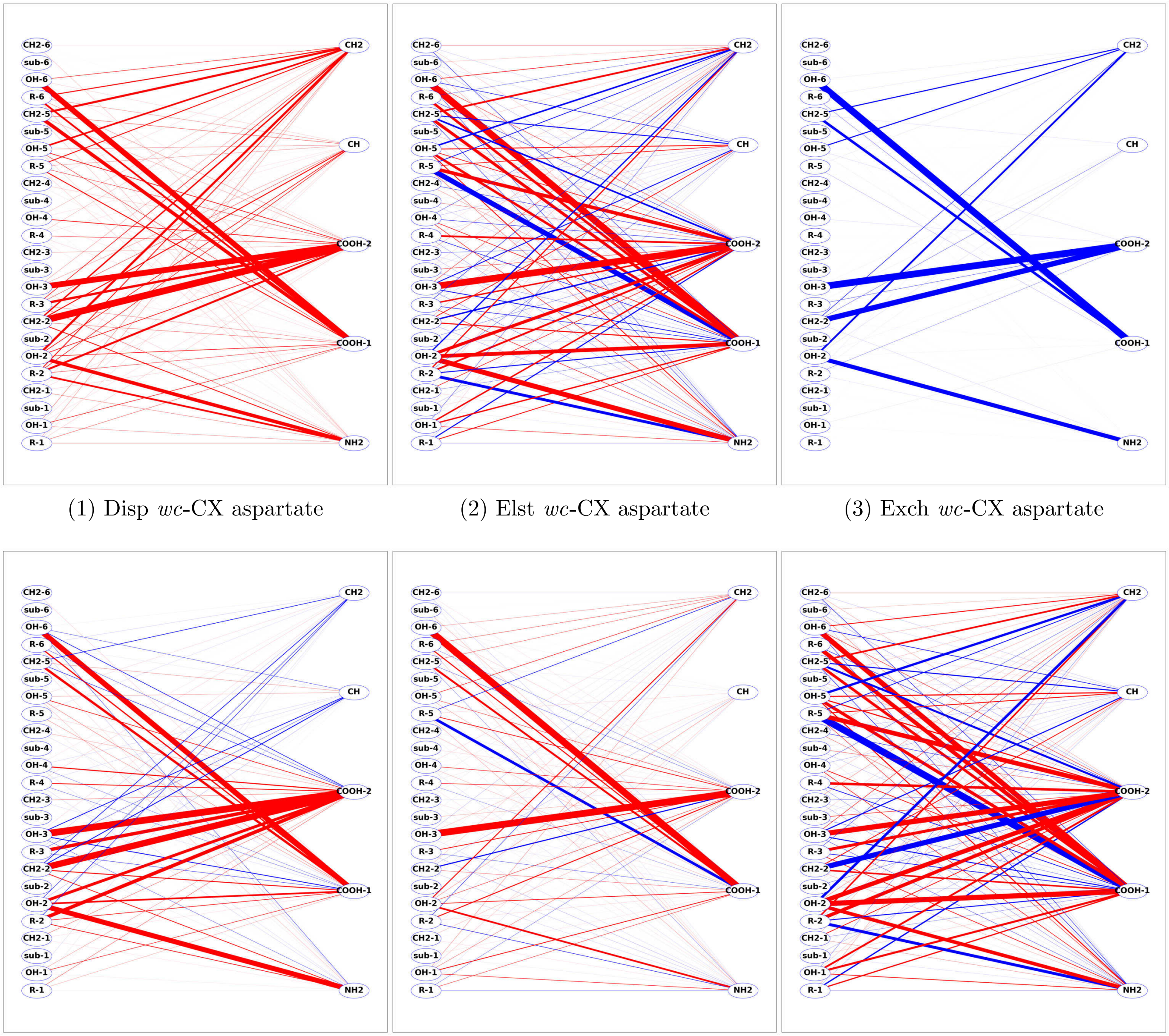

(1) Disp *wc*-CX aspartate

(2) Elst *wc*-CX aspartate

(3) Exch *wc*-CX aspartate

(4) IndAB *wc*-CX aspartate

(5) IndBA *wc*-CX aspartate

(6) Total *wc*-CX aspartate

Figure S44: The F-SAPT partitioning for *wc*-CX aspartate

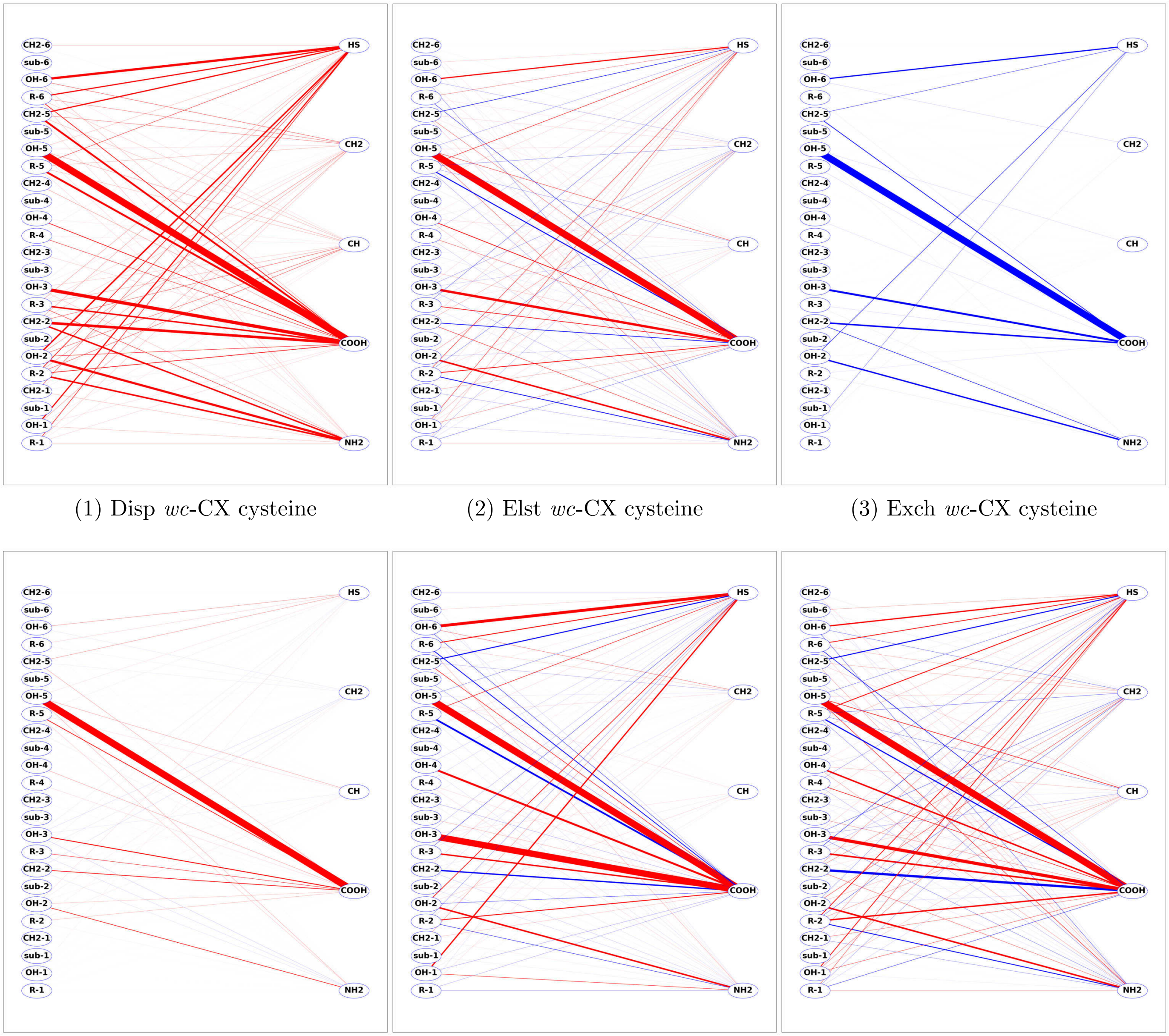

(1) Disp *wc*-CX cysteine (2) Elst *wc*-CX cysteine (3) Exch *wc*-CX cysteine

(4) IndAB *wc*-CX cysteine (5) IndBA *wc*-CX cysteine (6) Total *wc*-CX cysteine

Figure S45: The F-SAPT partitioning for *wc*-CX cysteine

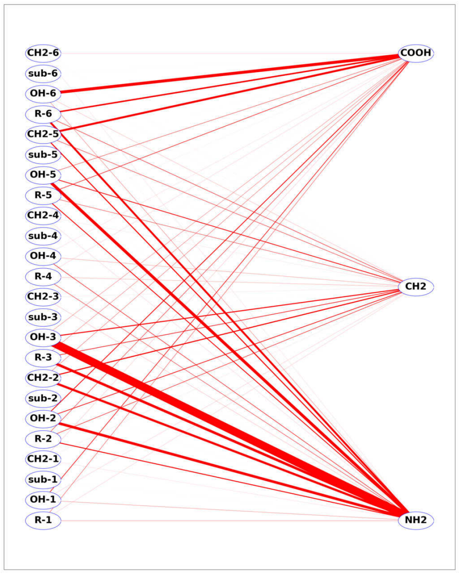

(1) Disp *wc*-CX glycine

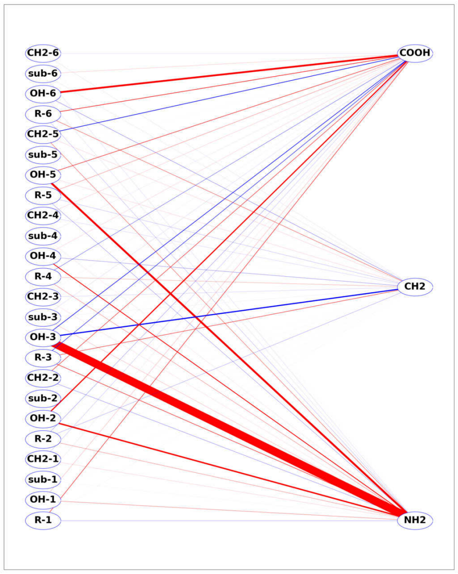

(2) Elst *wc*-CX glycine

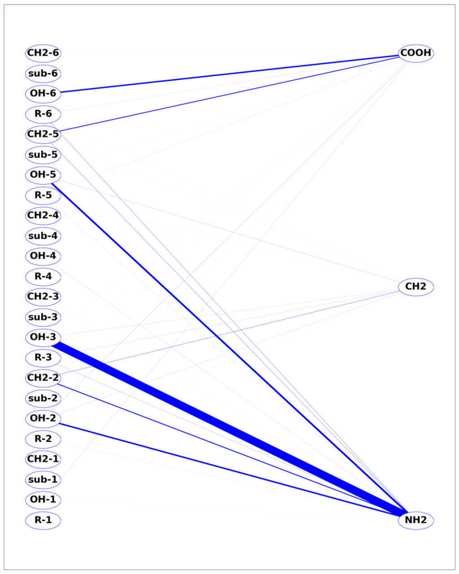

(3) Exch *wc*-CX glycine

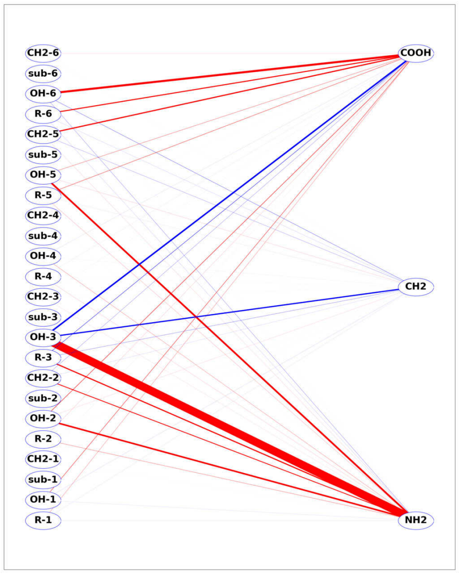

(4) IndAB *wc*-CX glycine

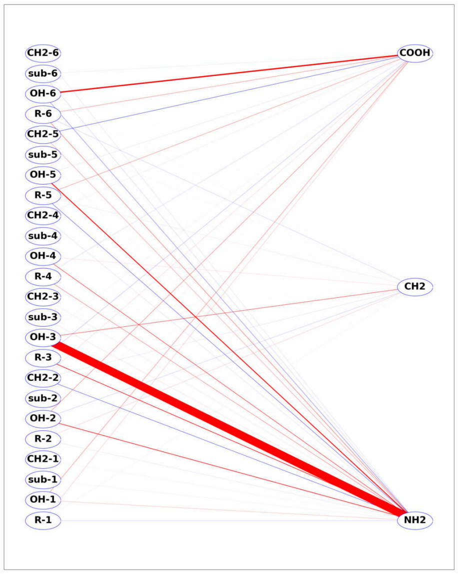

(5) IndBA *wc*-CX glycine

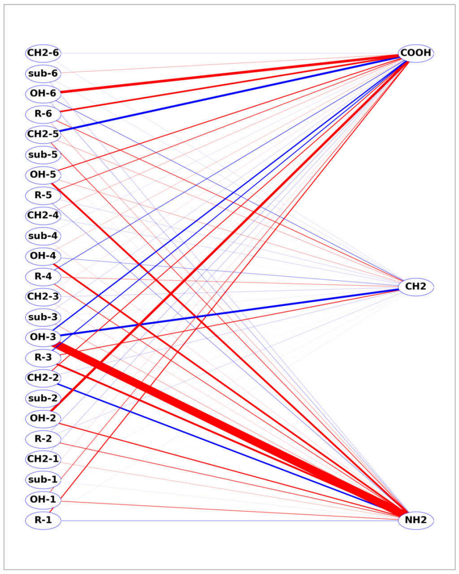

(6) Total *wc*-CX glycine

Figure S46: The F-SAPT partitioning for *wc*-CX glycine

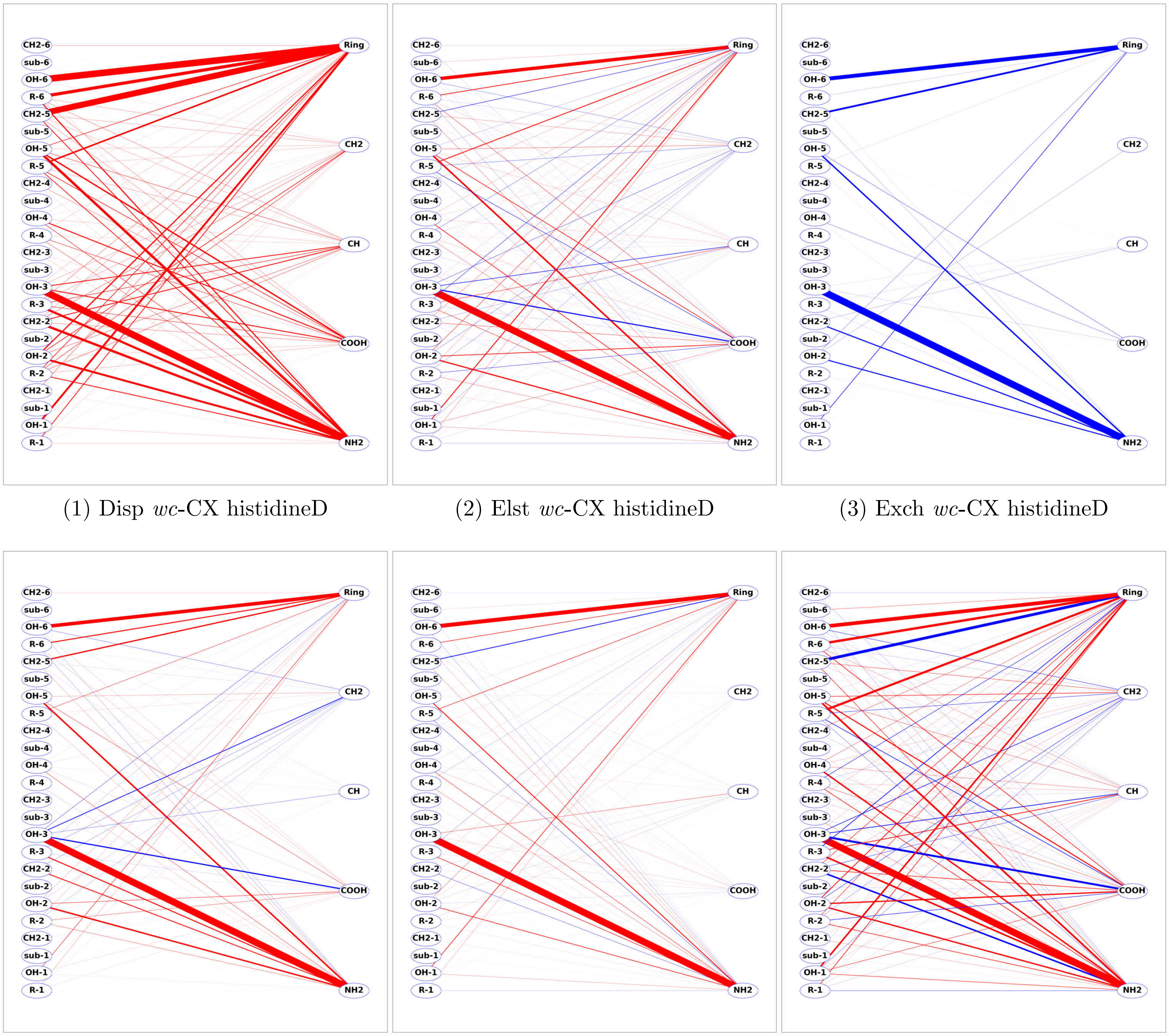

(1) Disp *wc*-CX histidineD (2) Elst *wc*-CX histidineD (3) Exch *wc*-CX histidineD

(4) IndAB *wc*-CX histidineD (5) IndBA *wc*-CX histidineD (6) Total *wc*-CX histidineD

Figure S47: The F-SAPT partitioning for *wc*-CX histidineD

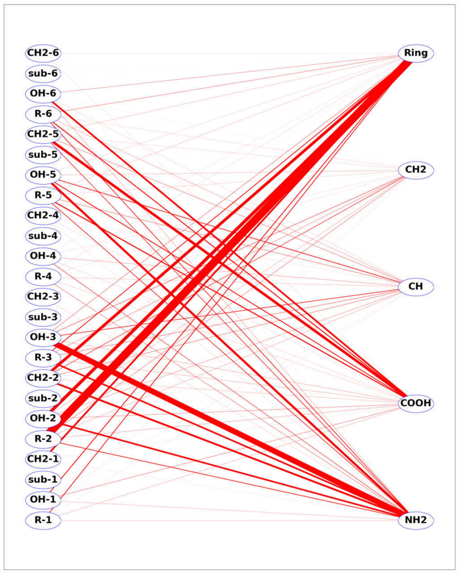

(1) Disp *wc*-CX histidineE

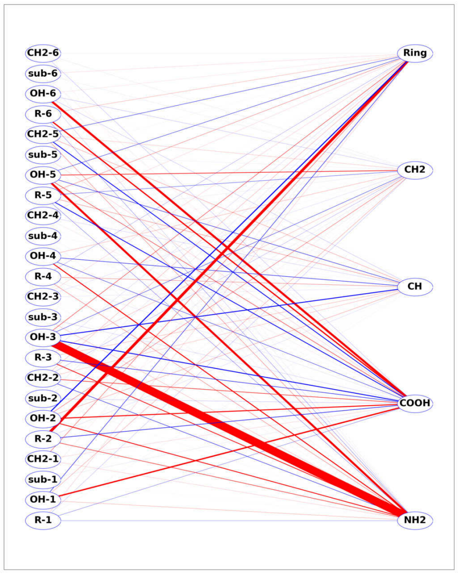

(2) Elst *wc*-CX histidineE

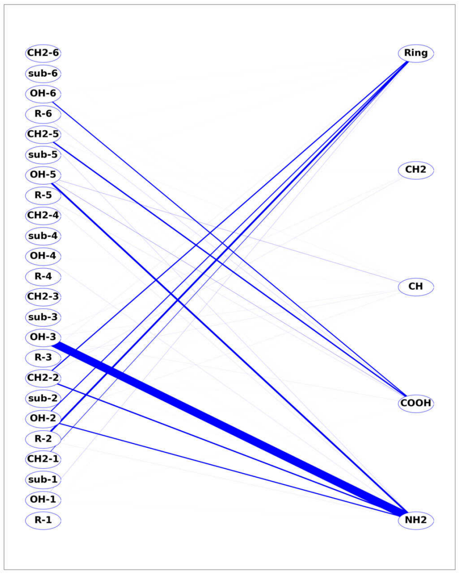

(3) Exch *wc*-CX histidineE

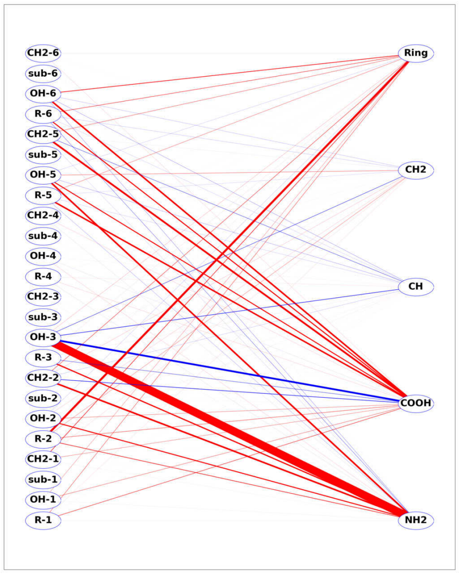

(4) IndAB *wc*-CX histidineE

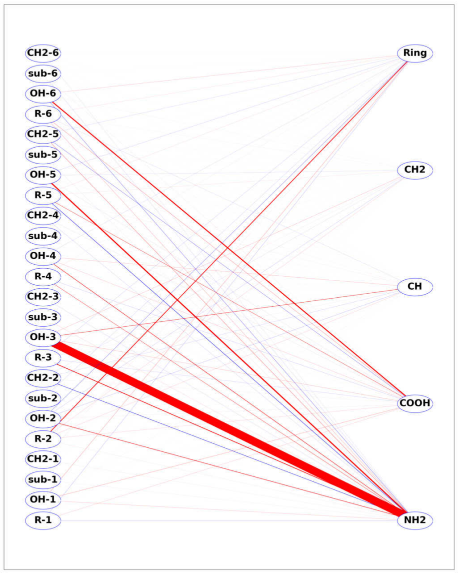

(5) IndBA *wc*-CX histidineE

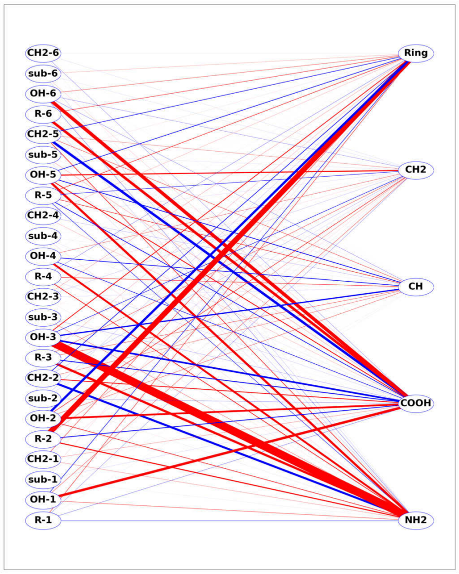

(6) Total *wc*-CX histidineE

Figure S48: The F-SAPT partitioning for *wc*-CX histidineE

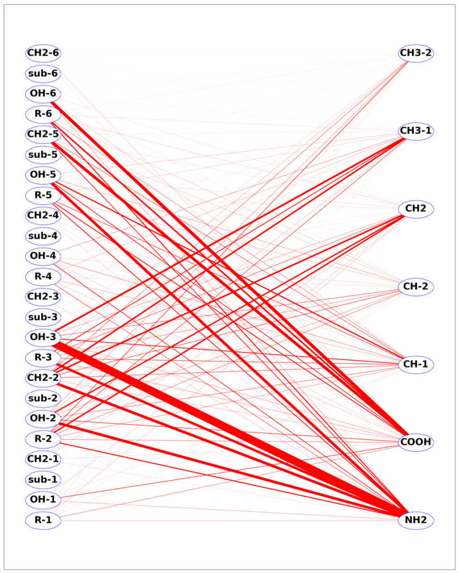

(1) Disp *wc*-CX isoleucine

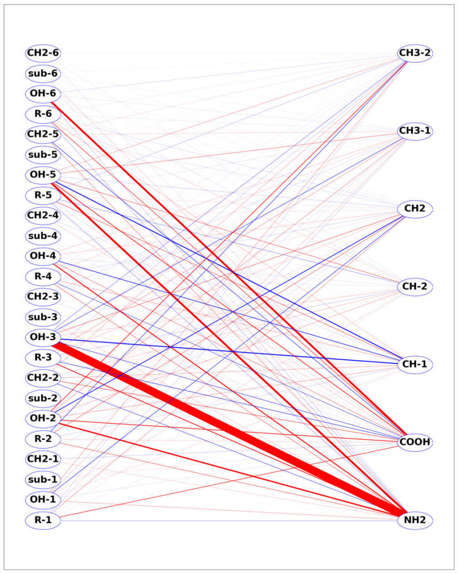

(2) Elst *wc*-CX isoleucine

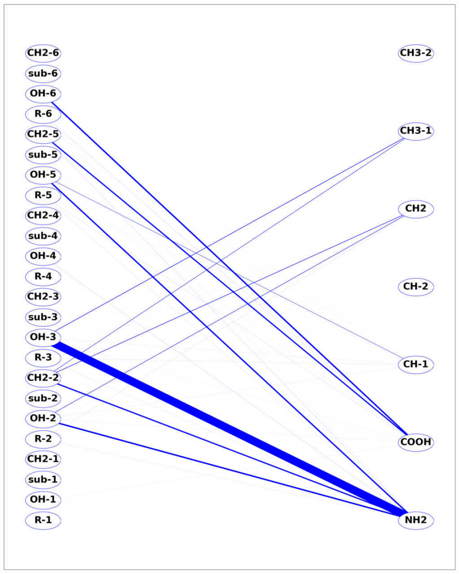

(3) Exch *wc*-CX isoleucine

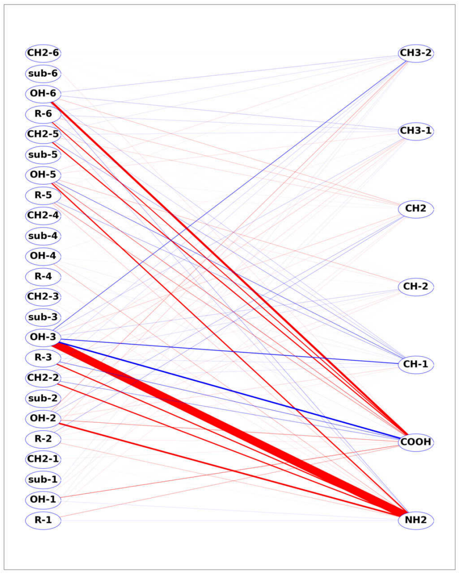

(4) IndAB *wc*-CX isoleucine

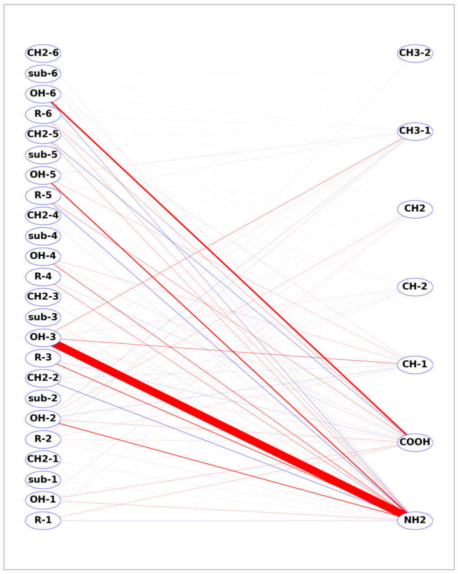

(5) IndBA *wc*-CX isoleucine

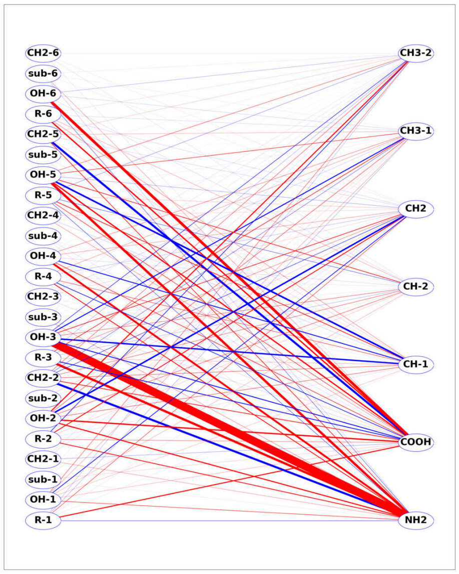

(6) Total *wc*-CX isoleucine

Figure S49: The F-SAPT partitioning for *wc*-CX isoleucine

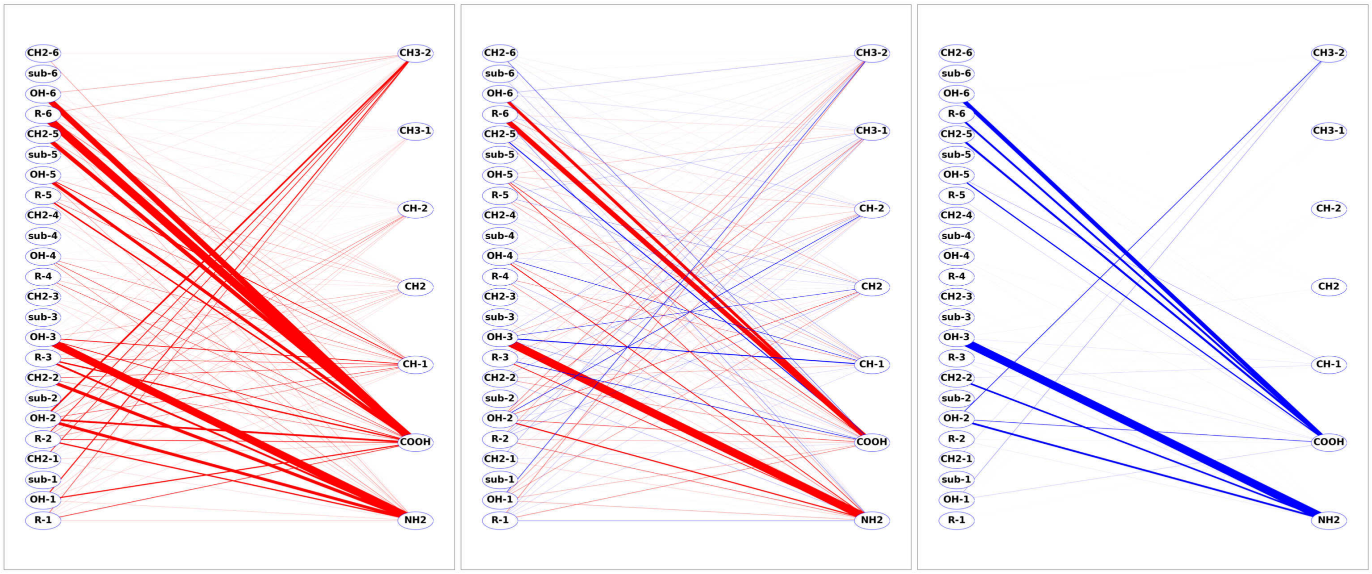

(1) Disp *wc*-CX leucine (2) Elst *wc*-CX leucine (3) Exch *wc*-CX leucine

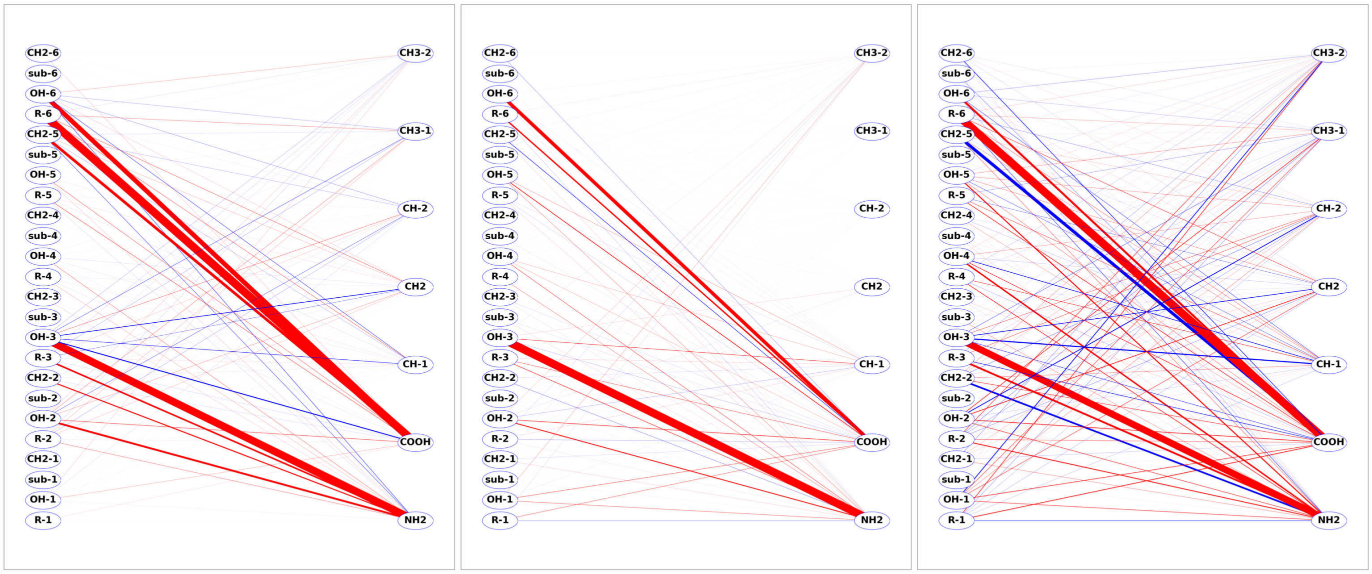

(4) IndAB *wc*-CX leucine (5) IndBA *wc*-CX leucine (6) Total *wc*-CX leucine

Figure S50: The F-SAPT partitioning for *wc*-CX leucine

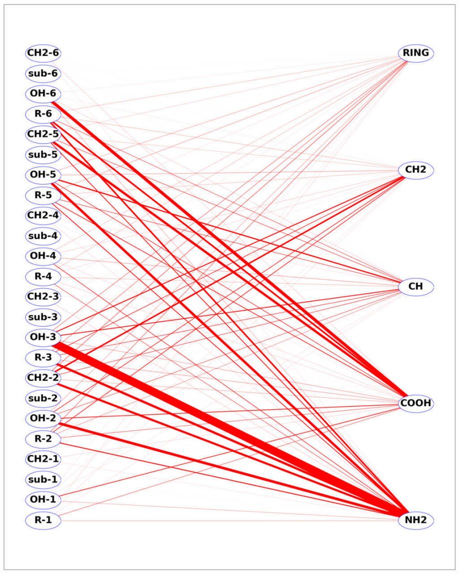

(1) Disp *wc*-CX phenylalanine

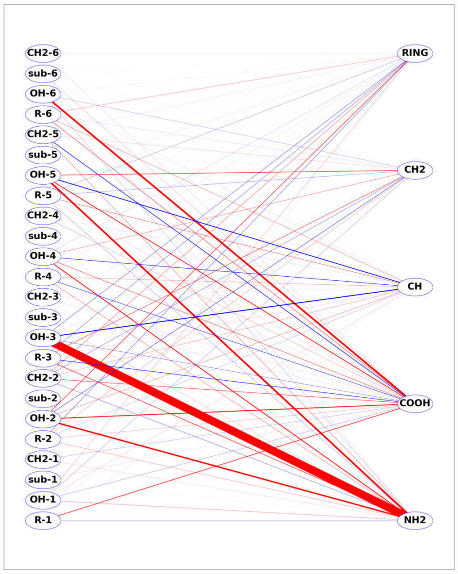

(2) Elst *wc*-CX phenylalanine

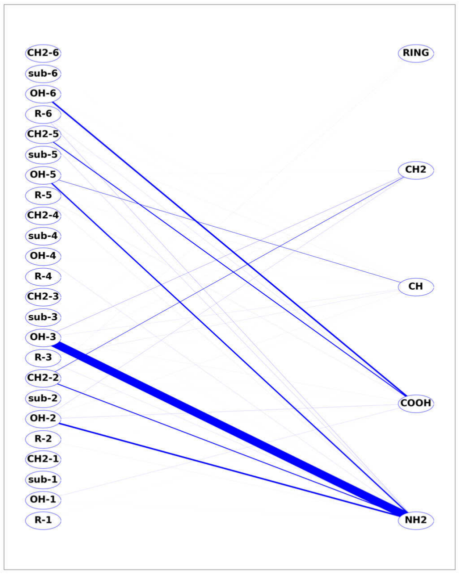

(3) Exch *wc*-CX phenylalanine

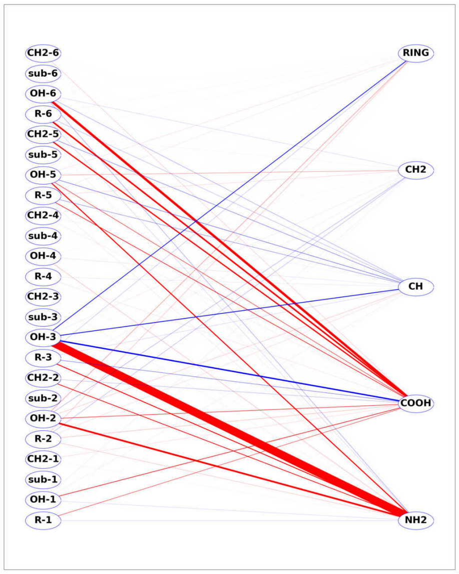

(4) IndAB *wc*-CX phenylalanine

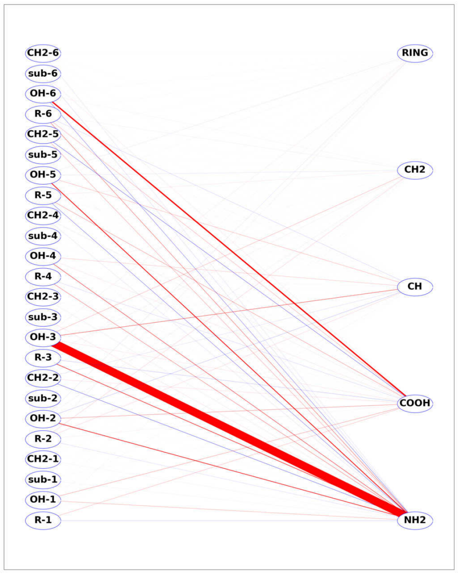

(5) IndBA *wc*-CX phenylalanine

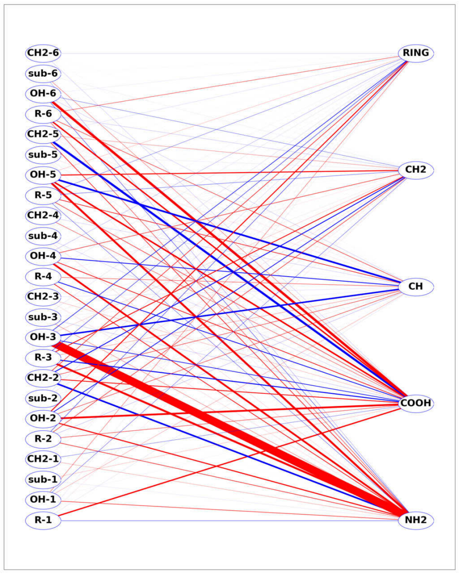

(6) Total *wc*-CX phenylalanine

Figure S51: The F-SAPT partitioning for *wc*-CX phenylalanine

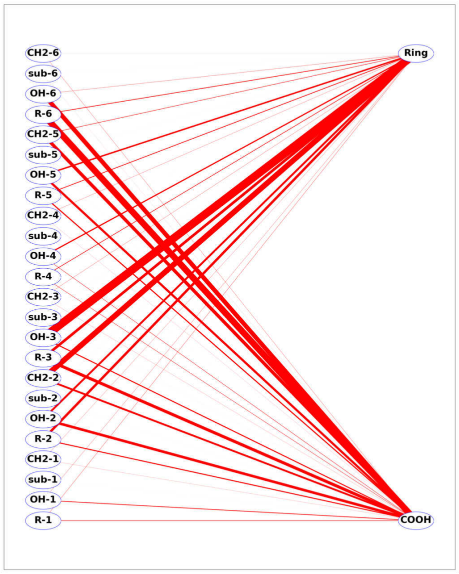

(1) Disp *wc*-CX proline

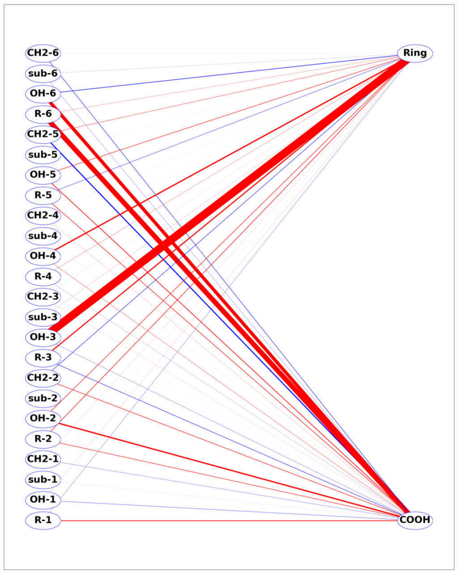

(2) Elst *wc*-CX proline

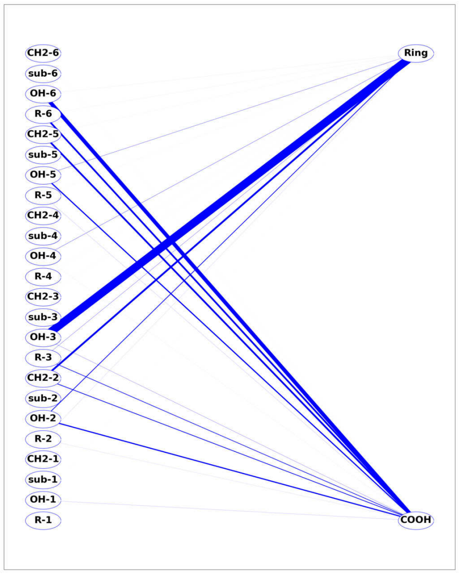

(3) Exch *wc*-CX proline

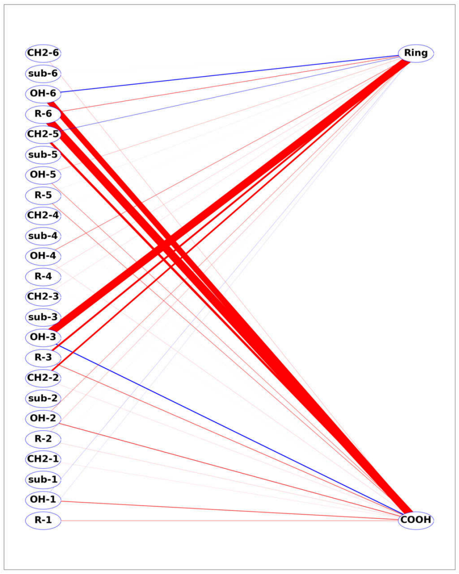

(4) IndAB *wc*-CX proline

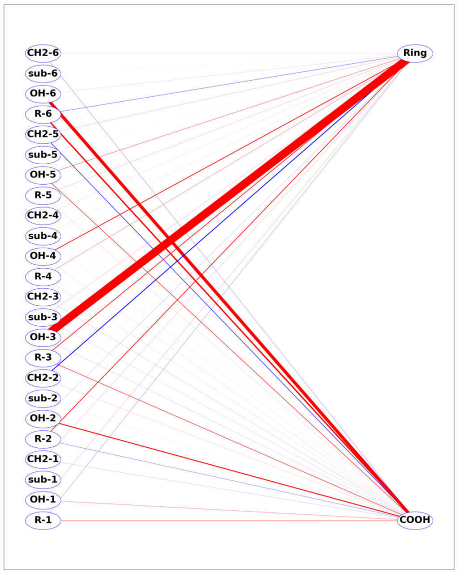

(5) IndBA *wc*-CX proline

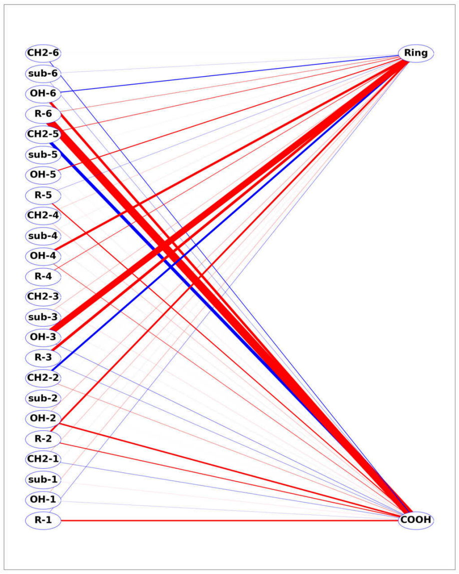

(6) Total *wc*-CX proline

Figure S52: The F-SAPT partitioning for *wc*-CX proline

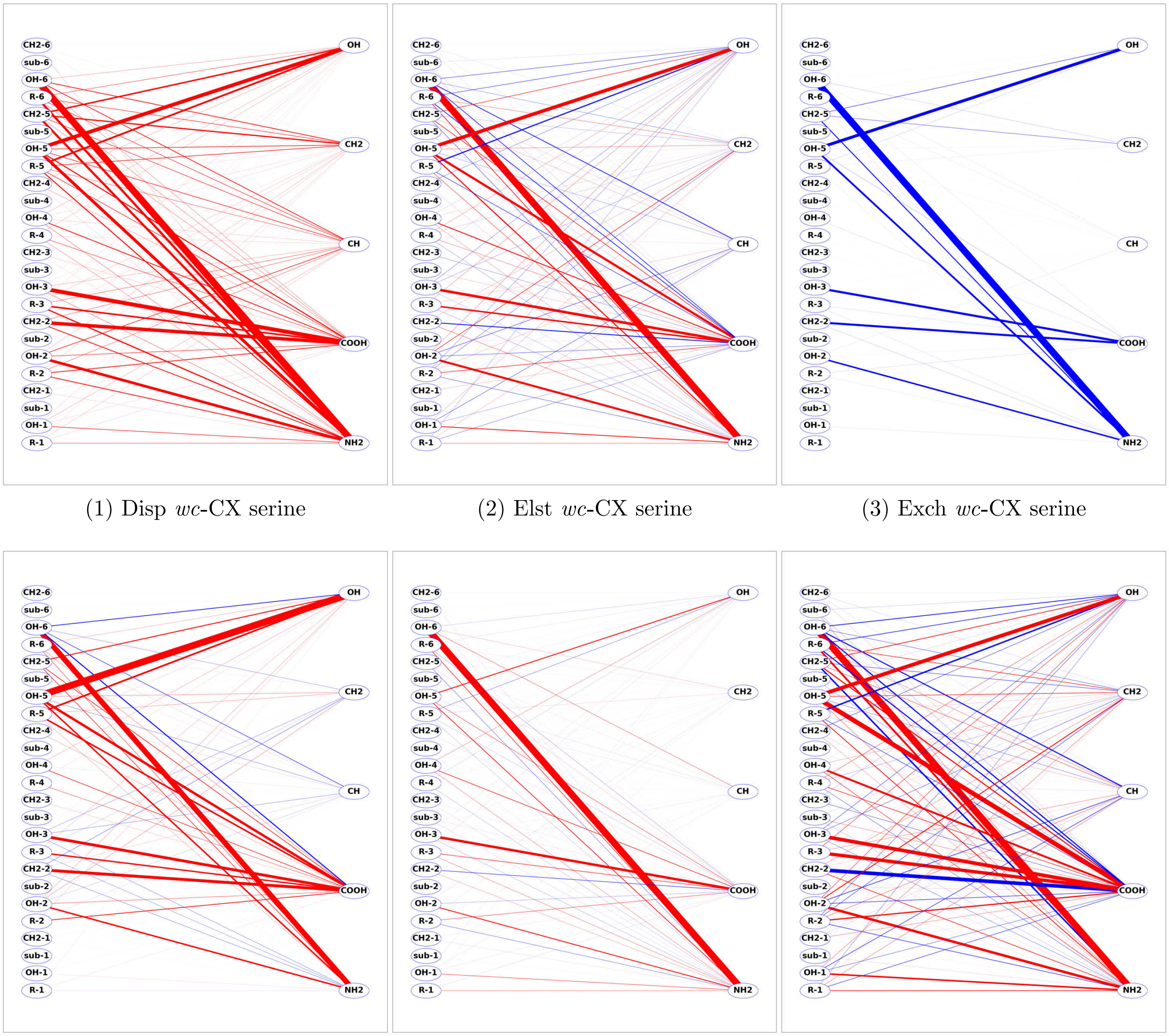

(1) Disp *wc*-CX serine

(2) Elst *wc*-CX serine

(3) Exch *wc*-CX serine

(4) IndAB *wc*-CX serine

(5) IndBA *wc*-CX serine

(6) Total *wc*-CX serine

Figure S53: The F-SAPT partitioning for *wc*-CX serine

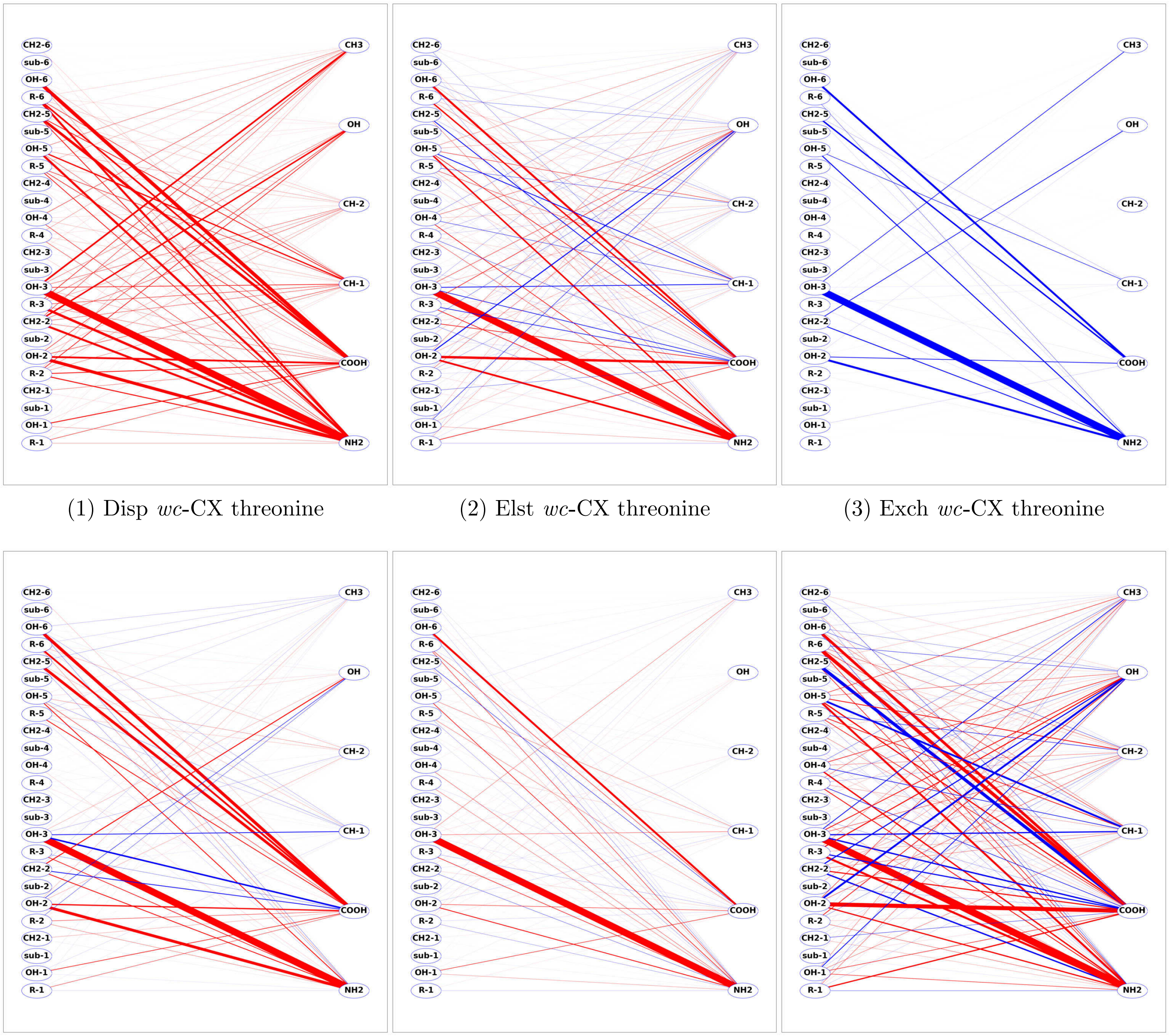

(1) Disp *wc*-CX threonine (2) Elst *wc*-CX threonine (3) Exch *wc*-CX threonine

(4) IndAB *wc*-CX threonine (5) IndBA *wc*-CX threonine (6) Total *wc*-CX threonine

Figure S54: The F-SAPT partitioning for *wc*-CX threonine

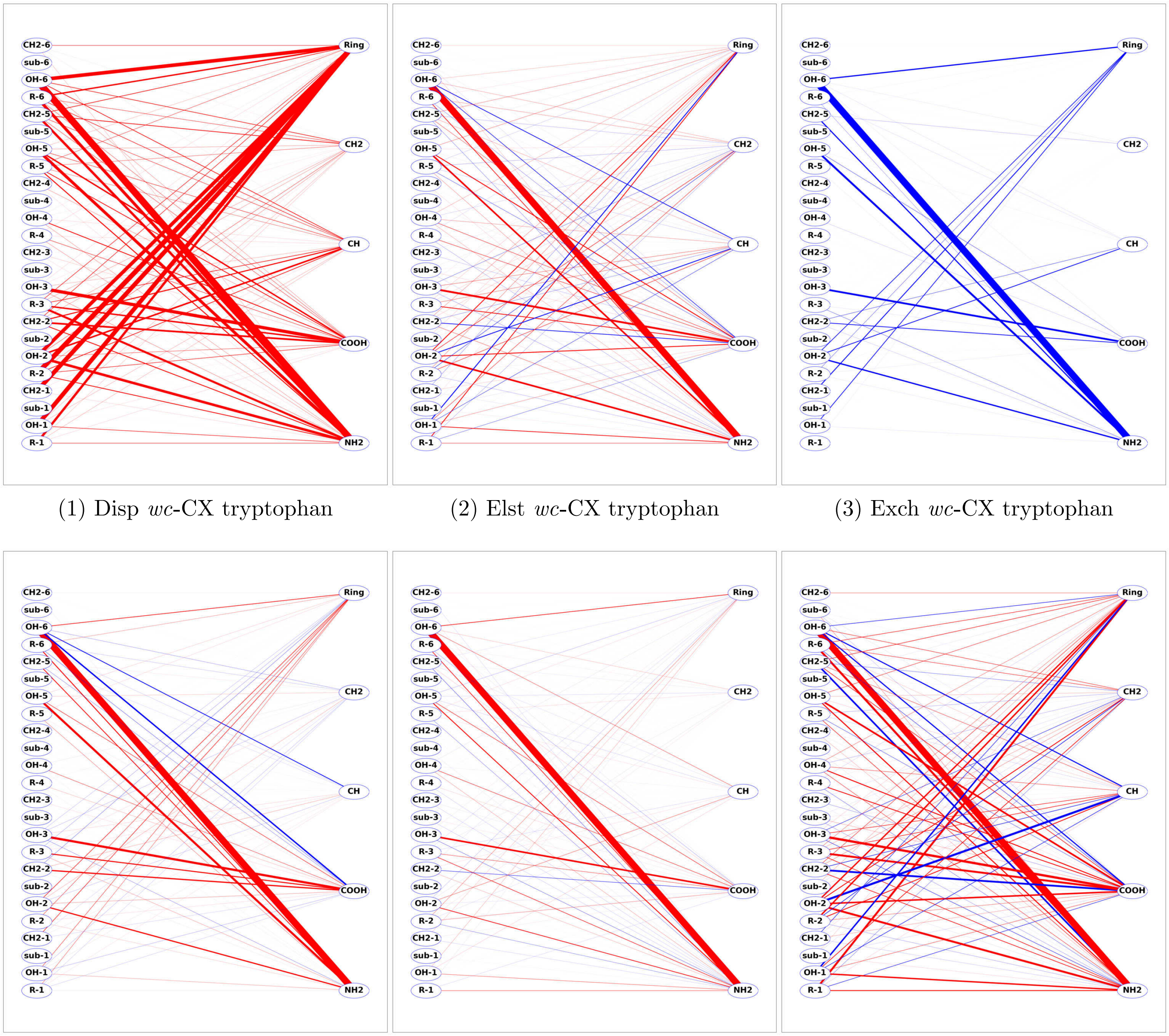

(1) Disp *wc*-CX tryptophan

(2) Elst *wc*-CX tryptophan

(3) Exch *wc*-CX tryptophan

(4) IndAB *wc*-CX tryptophan

(5) IndBA *wc*-CX tryptophan

(6) Total *wc*-CX tryptophan

Figure S55: The F-SAPT partitioning for *wc*-CX tryptophan

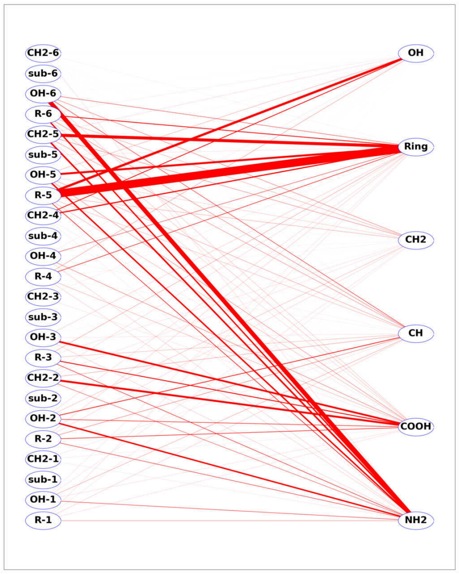

(1) Disp *wc*-CX tyrosine

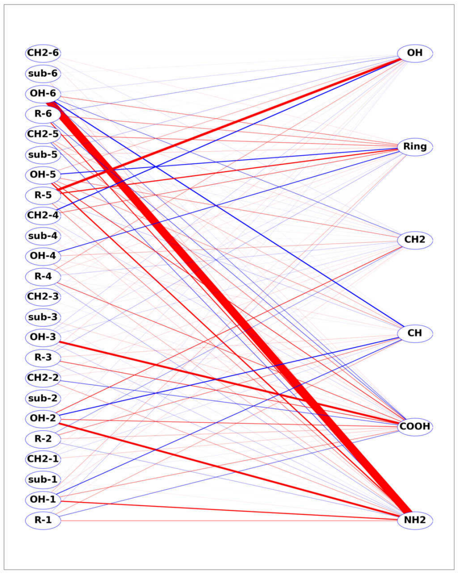

(2) Elst *wc*-CX tyrosine

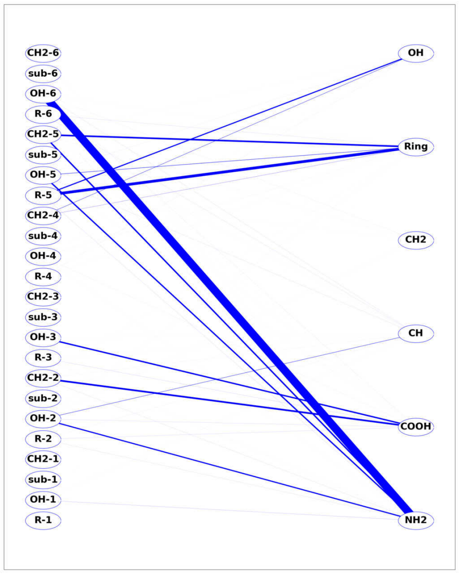

(3) Exch *wc*-CX tyrosine

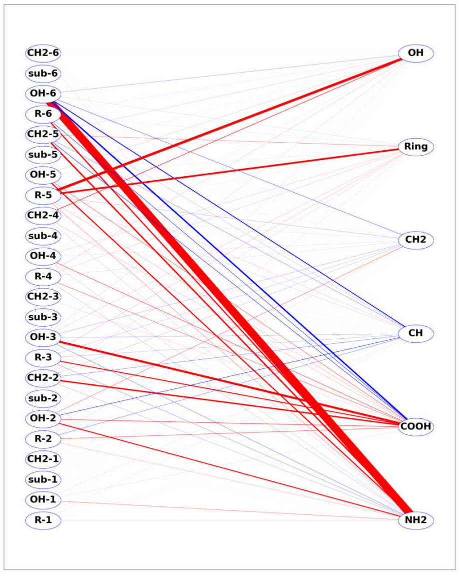

(4) IndAB *wc*-CX tyrosine

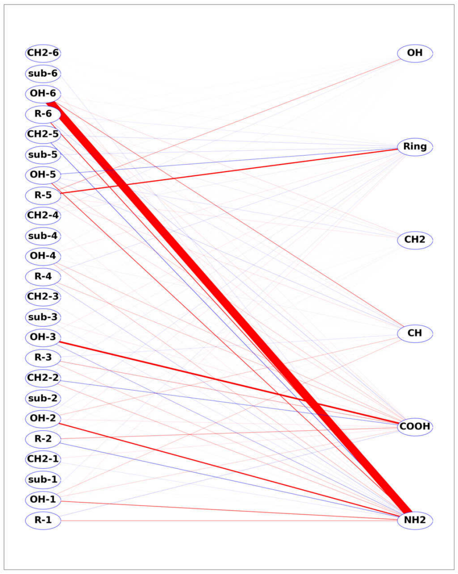

(5) IndBA *wc*-CX tyrosine

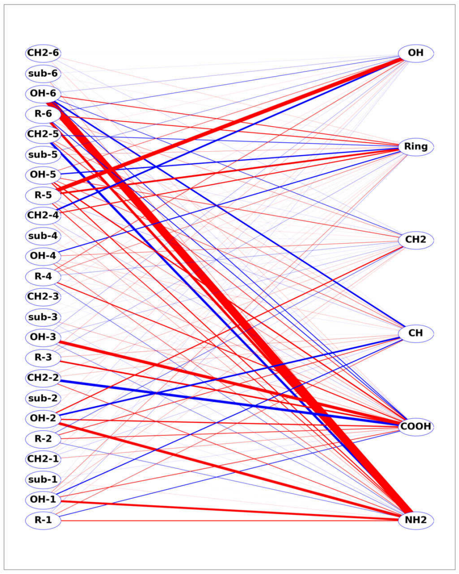

(6) Total *wc*-CX tyrosine

Figure S56: The F-SAPT partitioning for *wc*-CX tyrosine

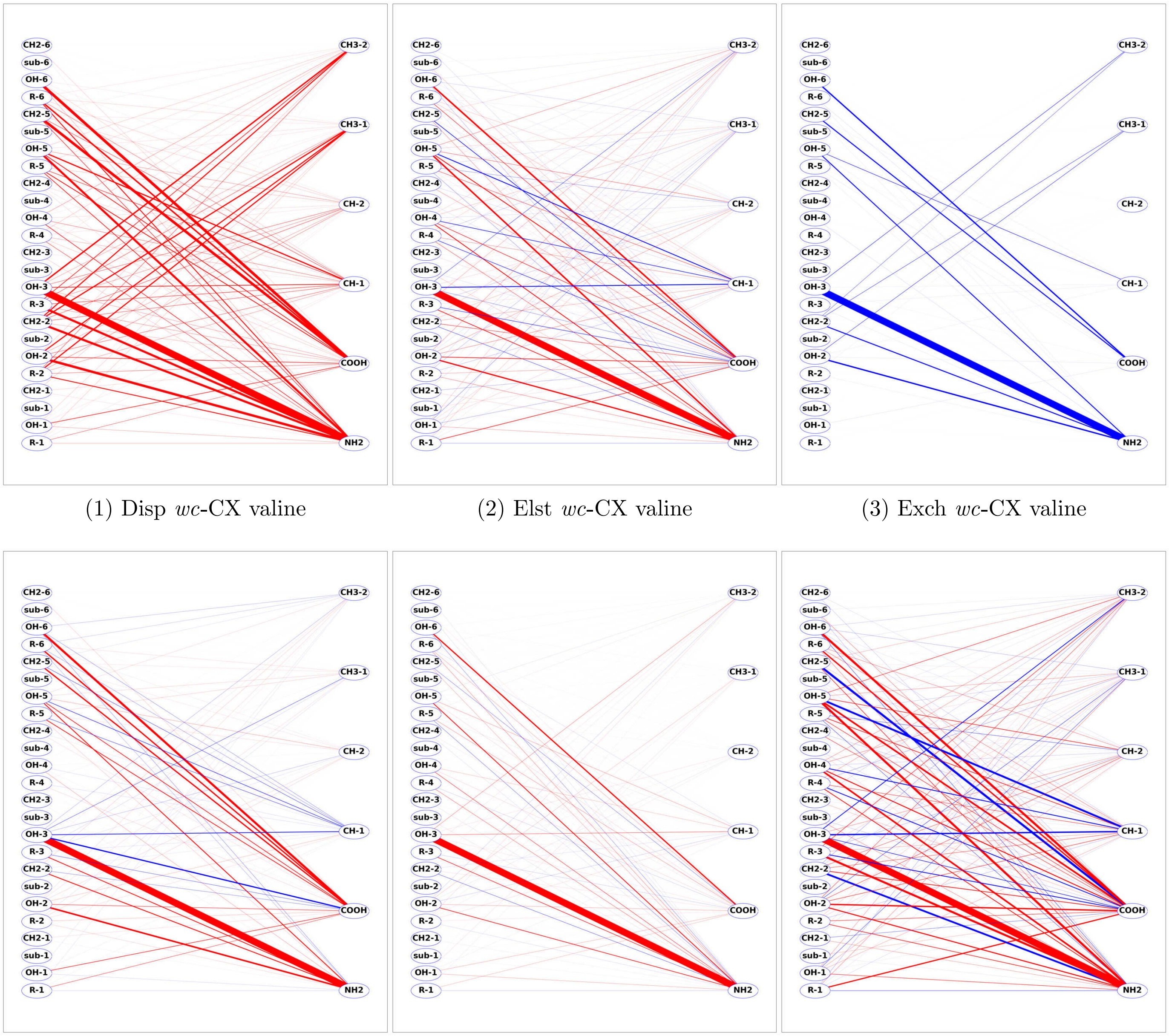

(1) Disp *wc*-CX valine

(2) Elst *wc*-CX valine

(3) Exch *wc*-CX valine

(4) IndAB *wc*-CX valine

(5) IndBA *wc*-CX valine

(6) Total *wc*-CX valine

Figure S57: The F-SAPT partitioning for *wc*-CX valine

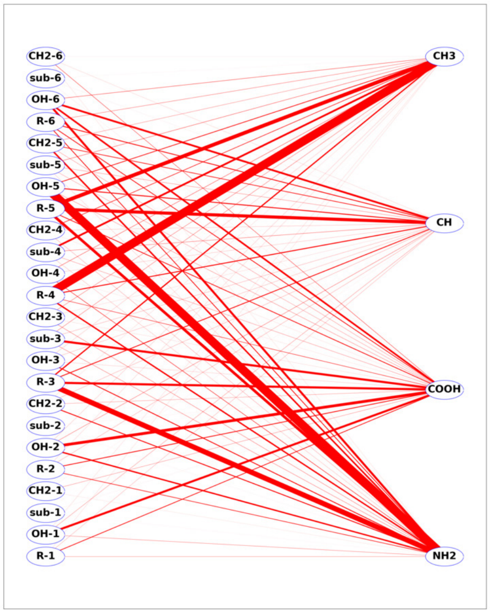

(1) Disp *al*-BCX alanine

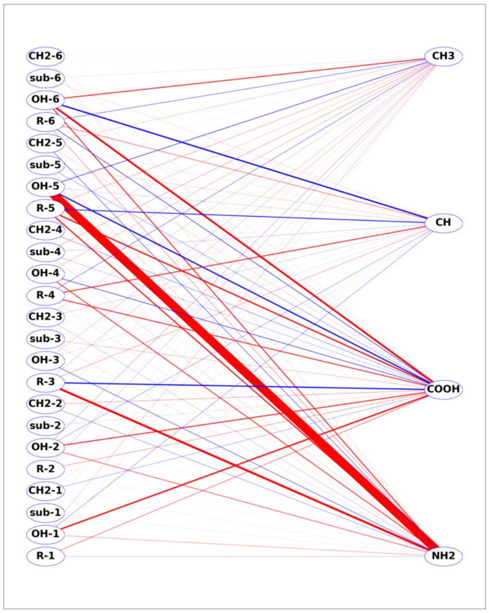

(2) Elst *al*-BCX alanine

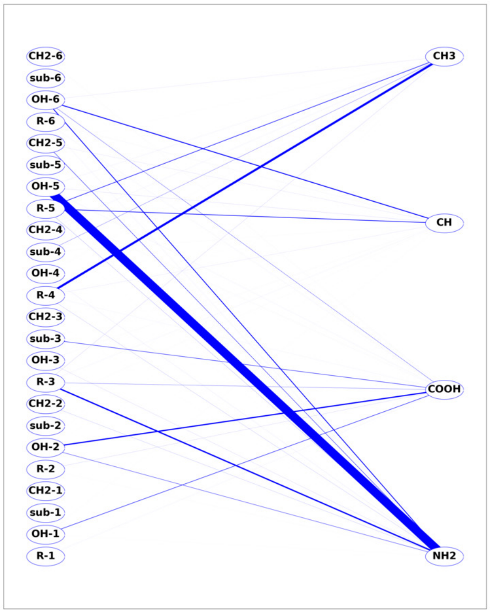

(3) Exch *al*-BCX alanine

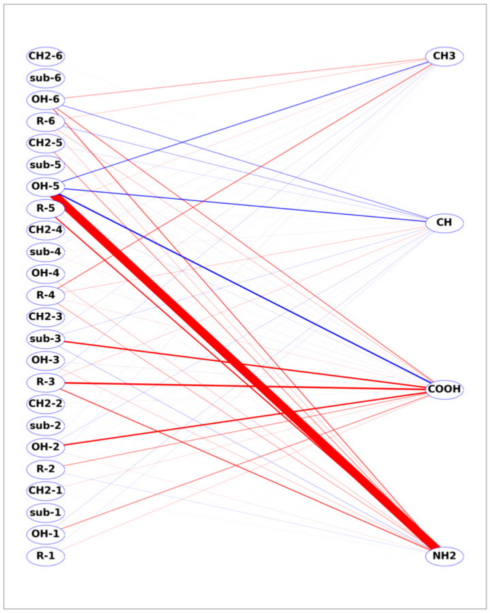

(4) IndAB *al*-BCX alanine

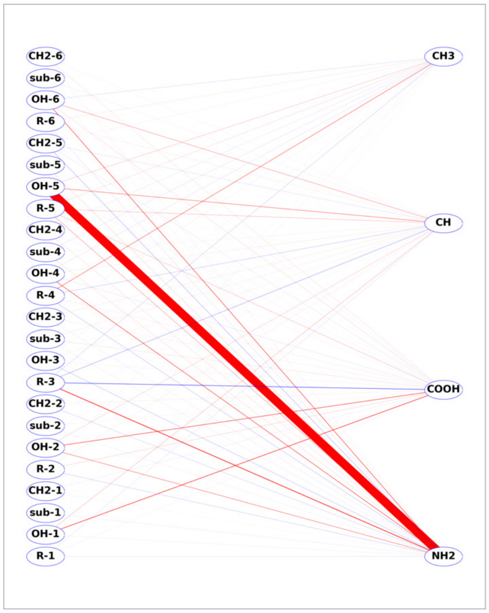

(5) IndBA *al*-BCX alanine

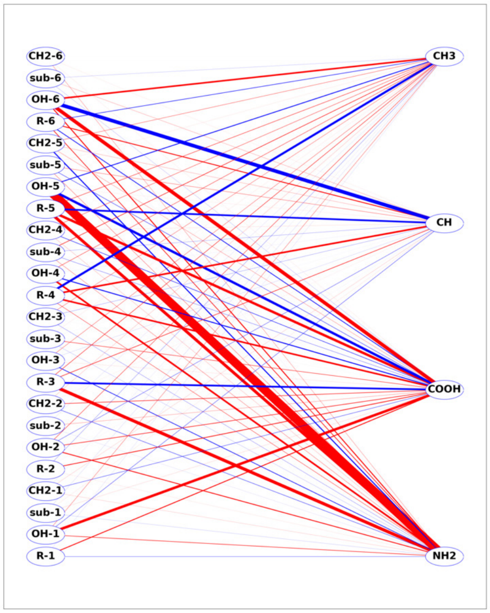

(6) Total *al*-BCX alanine

Figure S58: The F-SAPT partitioning for *al*-BCX alanine

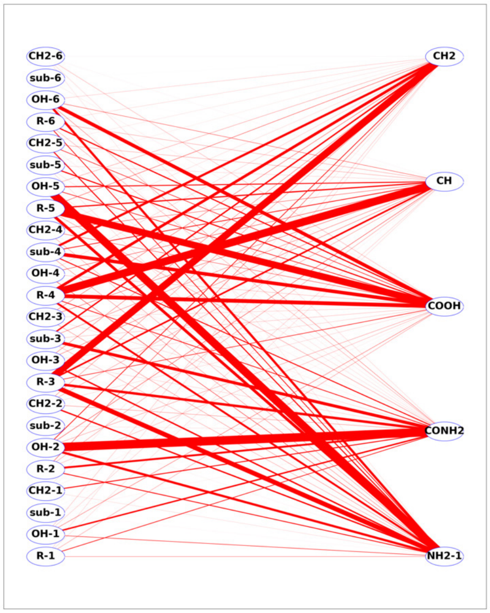

(1) Disp *al*-BCX asparagine

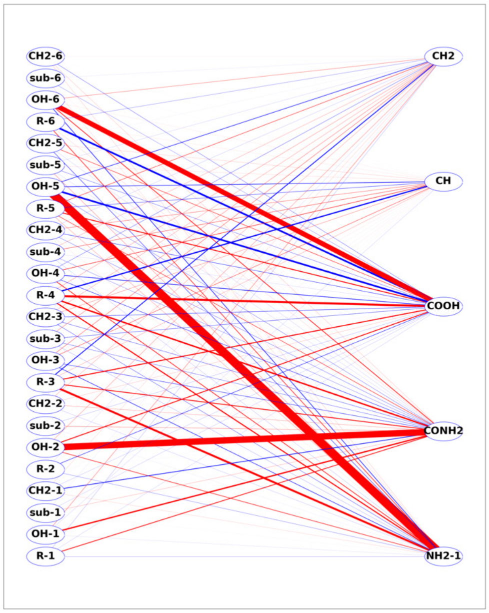

(2) Elst *al*-BCX asparagine

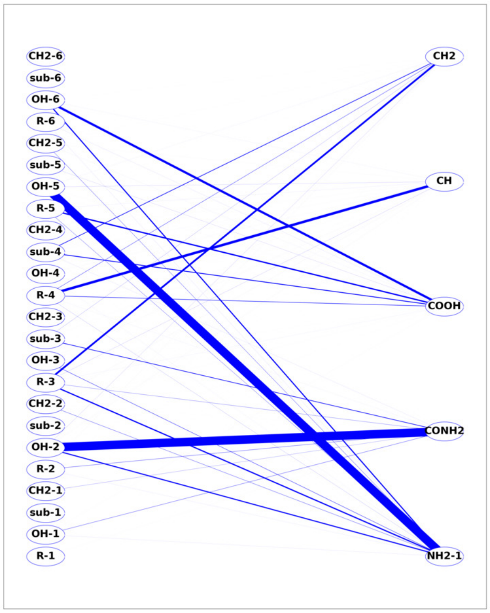

(3) Exch *al*-BCX asparagine

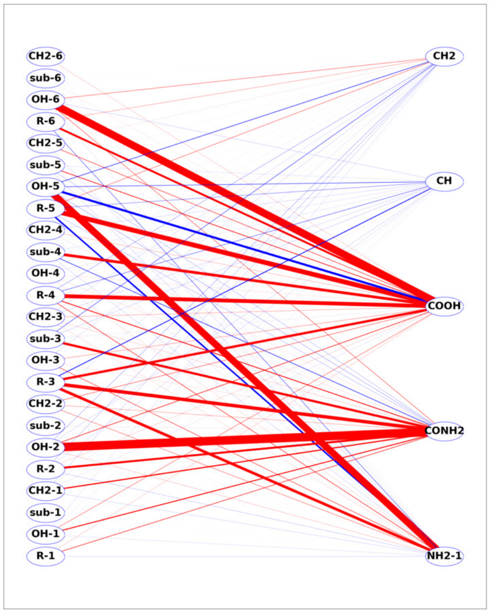

(4) IndAB *al*-BCX asparagine

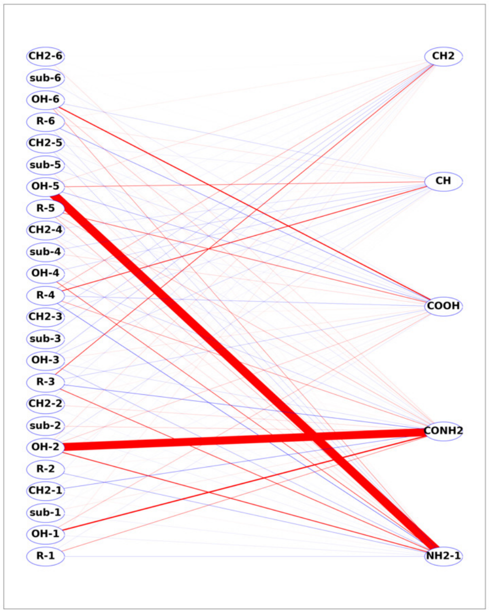

(5) IndBA *al*-BCX asparagine

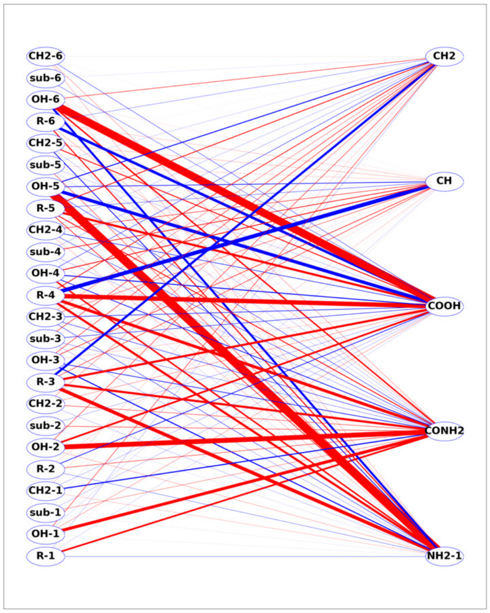

(6) Total *al*-BCX asparagine

Figure S59: The F-SAPT partitioning for *al*-BCX asparagine

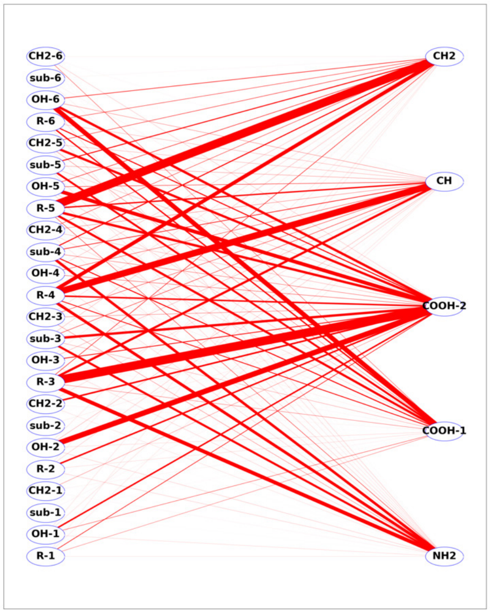

(1) Disp *al*-BCX aspartate

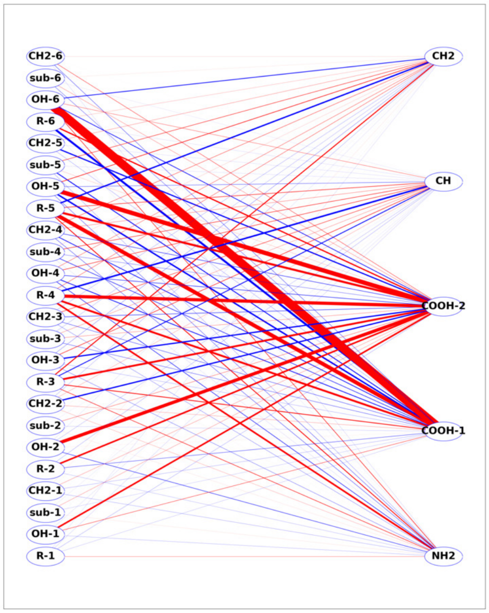

(2) Elst *al*-BCX aspartate

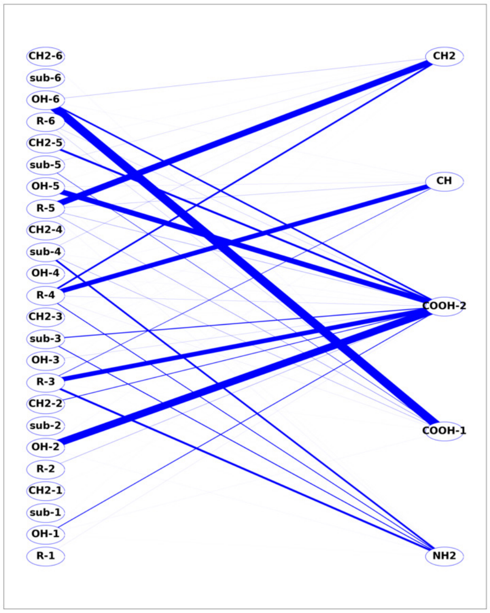

(3) Exch *al*-BCX aspartate

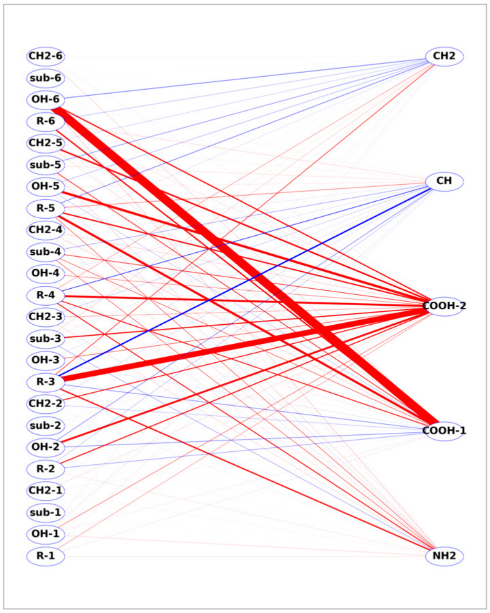

(4) IndAB *al*-BCX aspartate

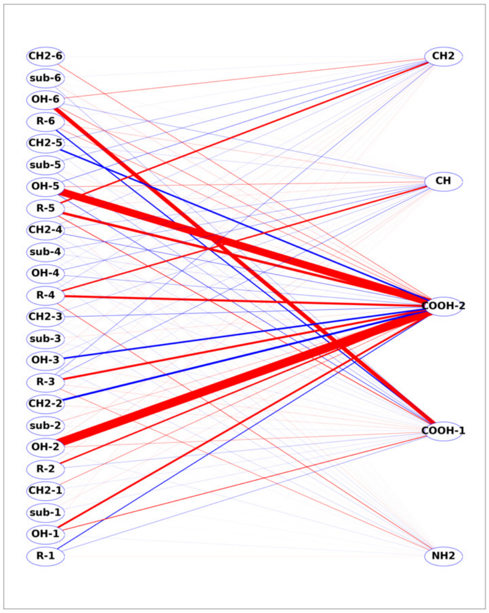

(5) IndBA *al*-BCX aspartate

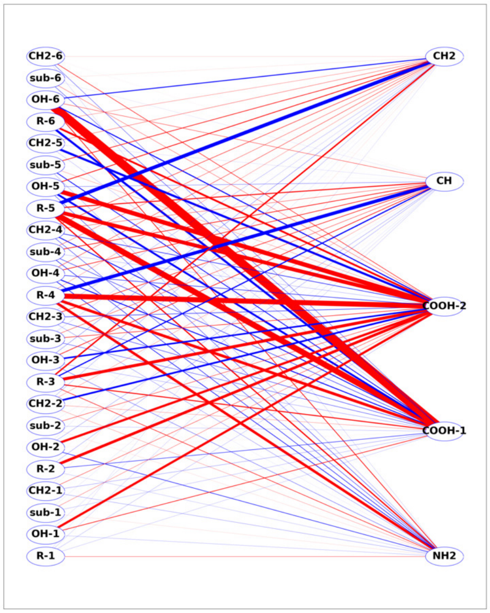

(6) Total *al*-BCX aspartate

Figure S60: The F-SAPT partitioning for *al*-BCX aspartate

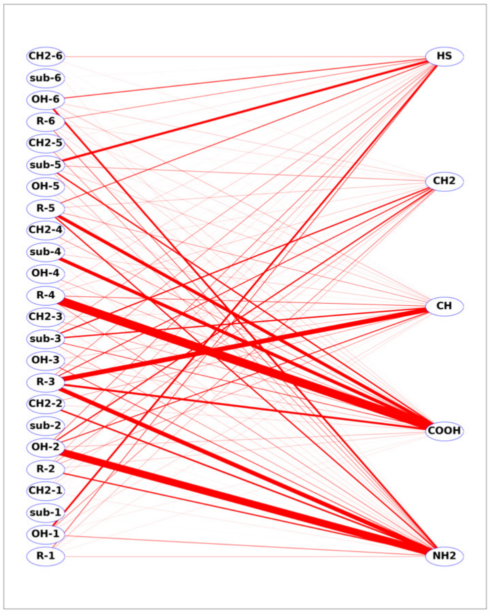

(1) Disp *al*-BCX cysteine

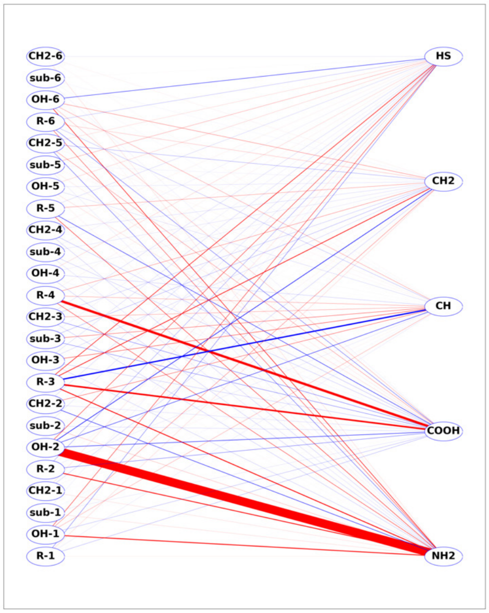

(2) Elst *al*-BCX cysteine

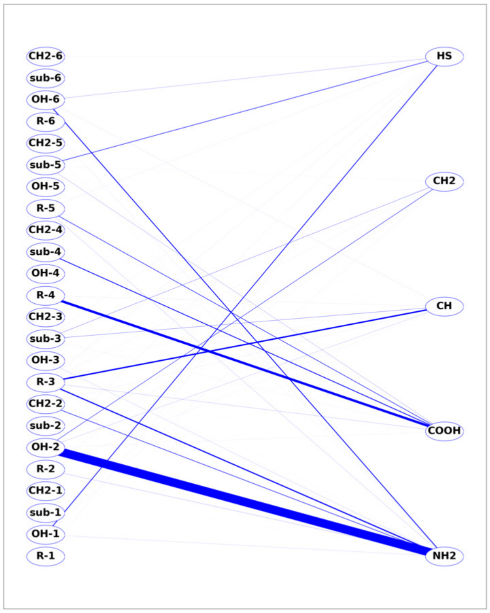

(3) Exch *al*-BCX cysteine

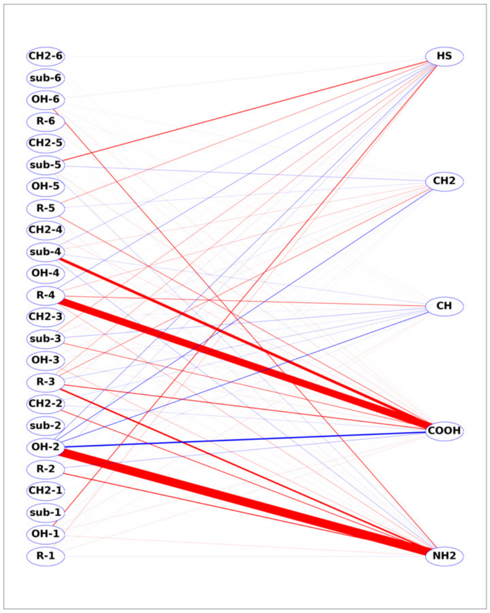

(4) IndAB *al*-BCX cysteine

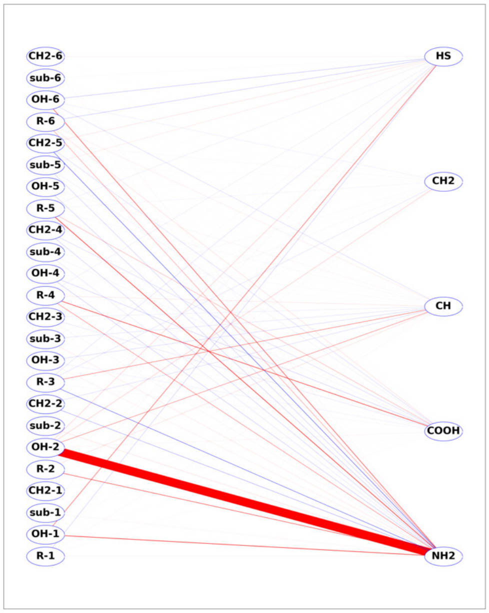

(5) IndBA *al*-BCX cysteine

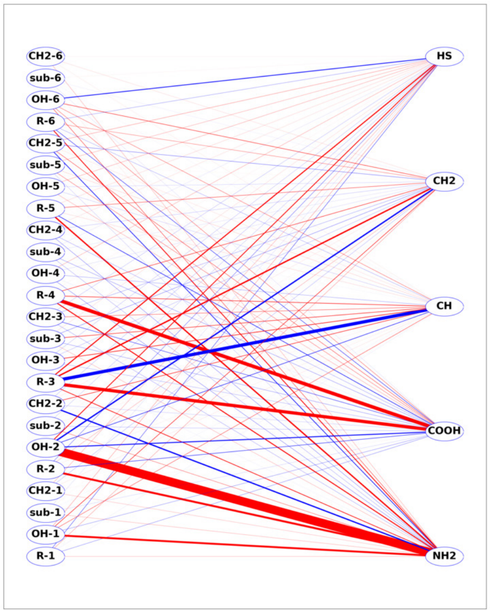

(6) Total *al*-BCX cysteine

Figure S61: The F-SAPT partitioning for *al*-BCX cysteine

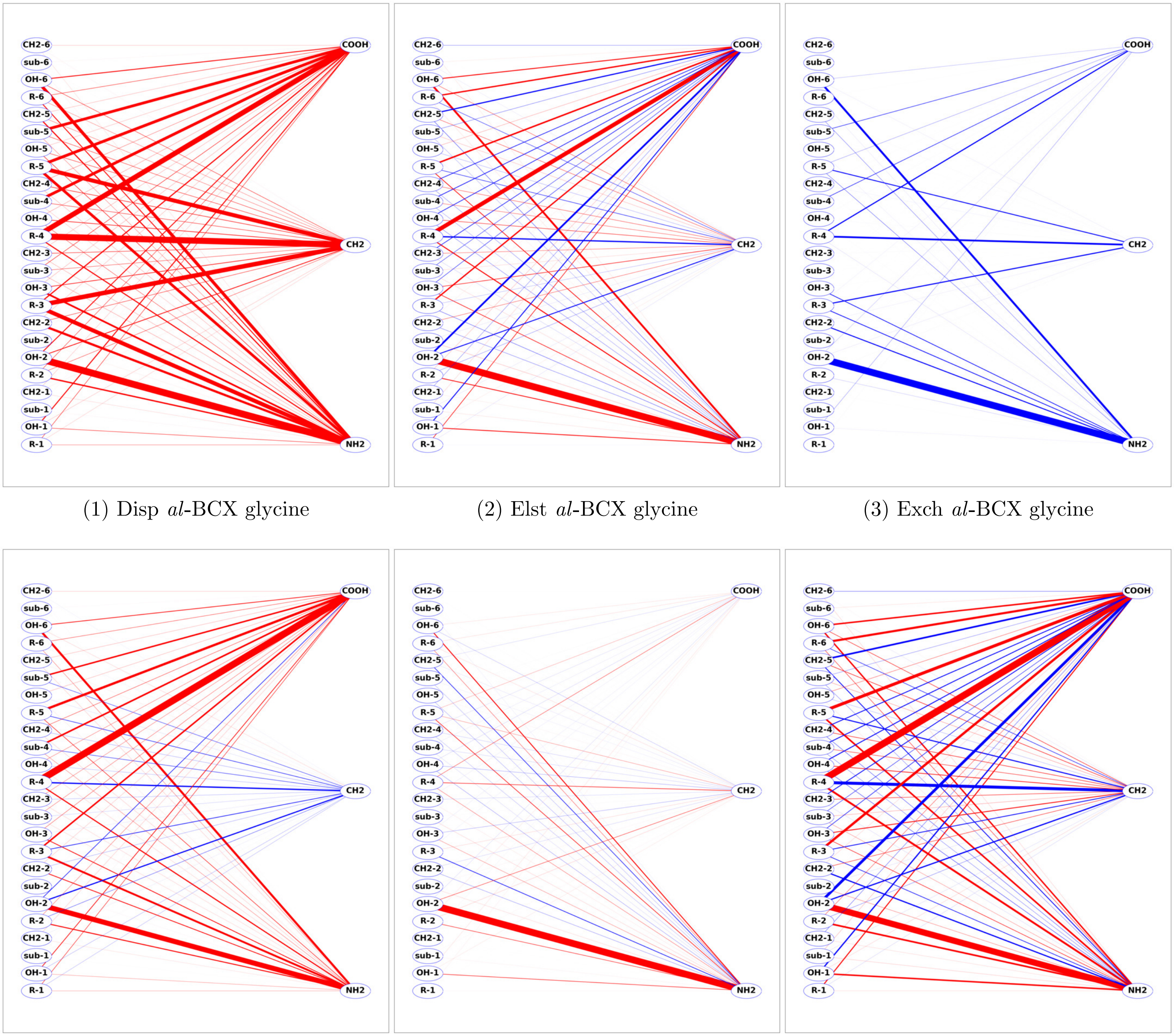

(1) Disp *al*-BCX glycine (2) Elst *al*-BCX glycine (3) Exch *al*-BCX glycine

(4) IndAB *al*-BCX glycine (5) IndBA *al*-BCX glycine (6) Total *al*-BCX glycine

Figure S62: The F-SAPT partitioning for *al*-BCX glycine

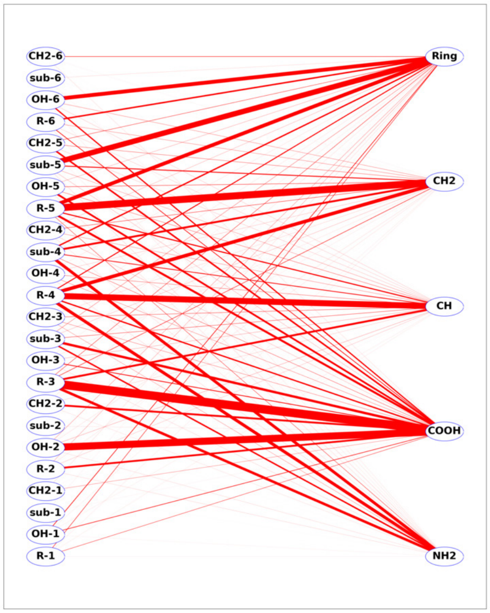

(1) Disp *al*-BCX histidineD

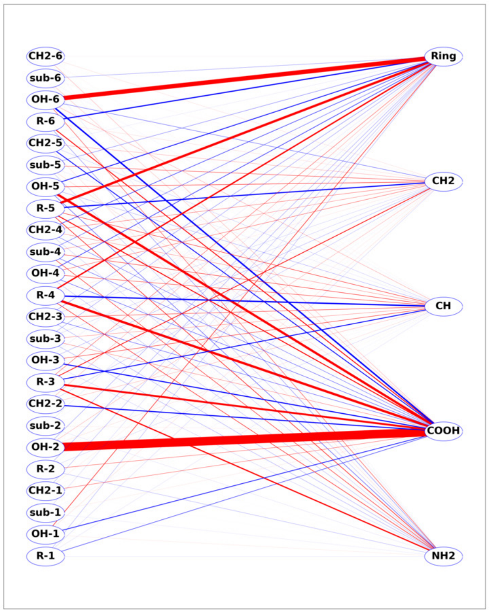

(2) Elst *al*-BCX histidineD

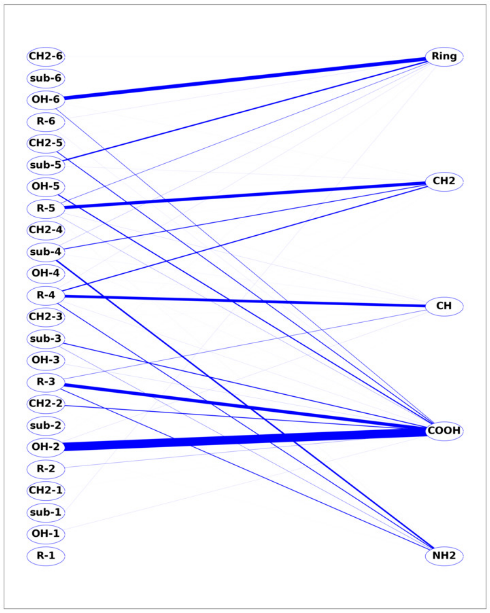

(3) Exch *al*-BCX histidineD

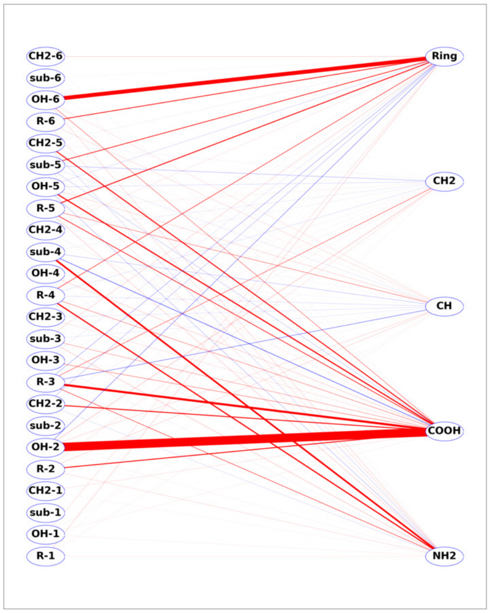

(4) IndAB *al*-BCX histidineD

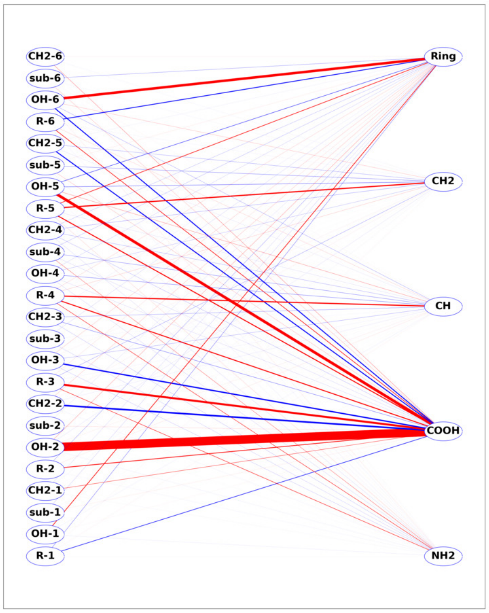

(5) IndBA *al*-BCX histidineD

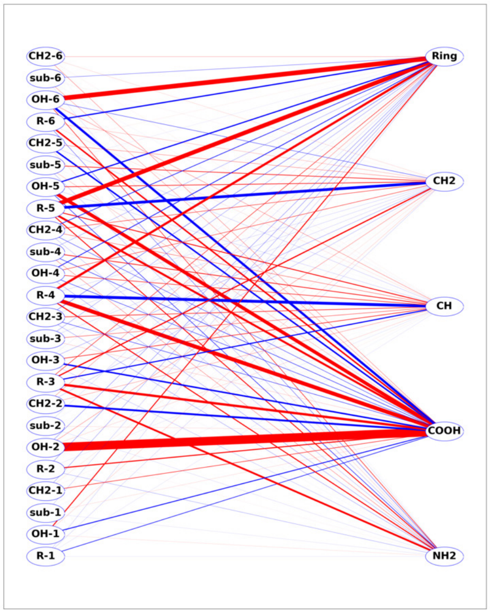

(6) Total *al*-BCX histidineD

Figure S63: The F-SAPT partitioning for *al*-BCX histidineD

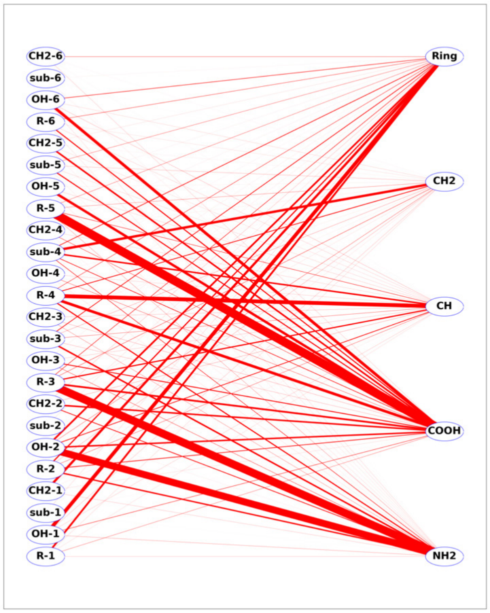

(1) Disp *al*-BCX histidineE

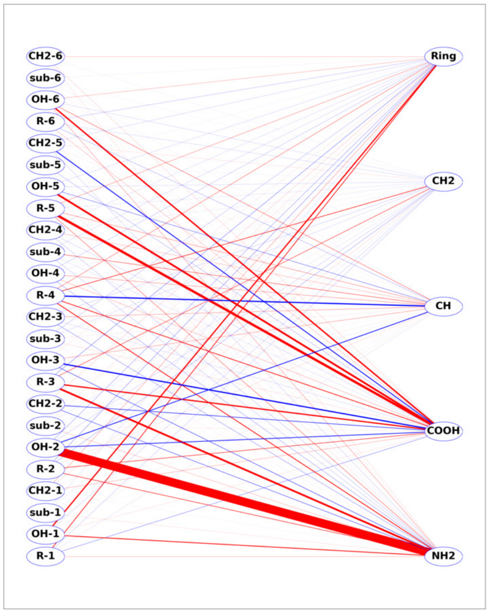

(2) Elst *al*-BCX histidineE

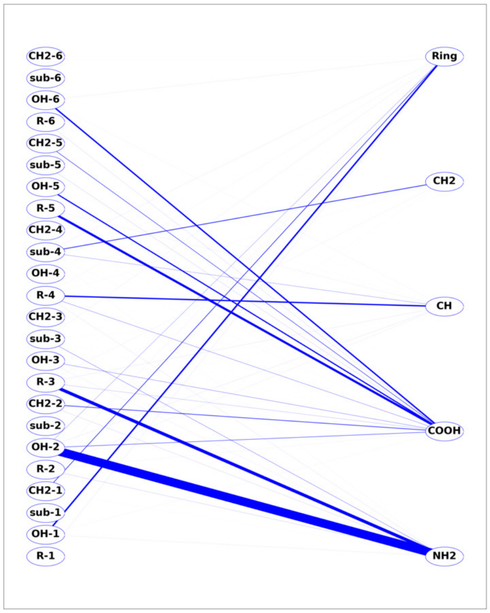

(3) Exch *al*-BCX histidineE

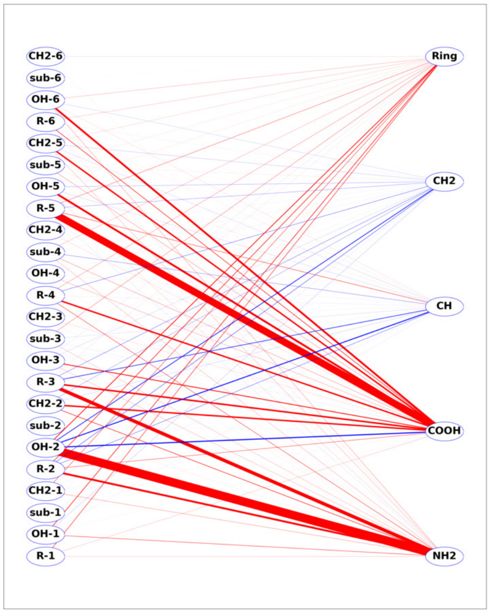

(4) IndAB *al*-BCX histidineE

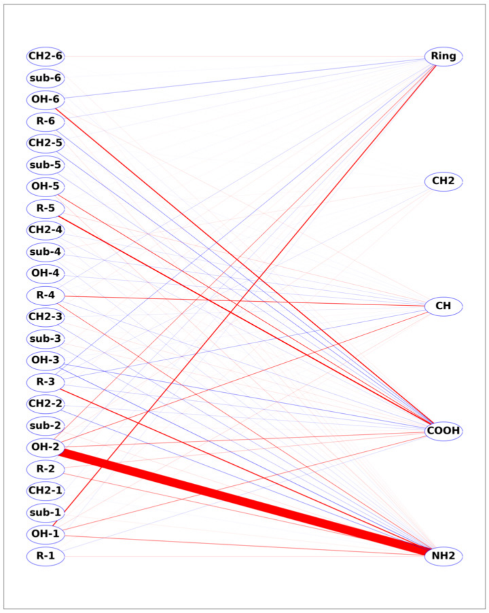

(5) IndBA *al*-BCX histidineE

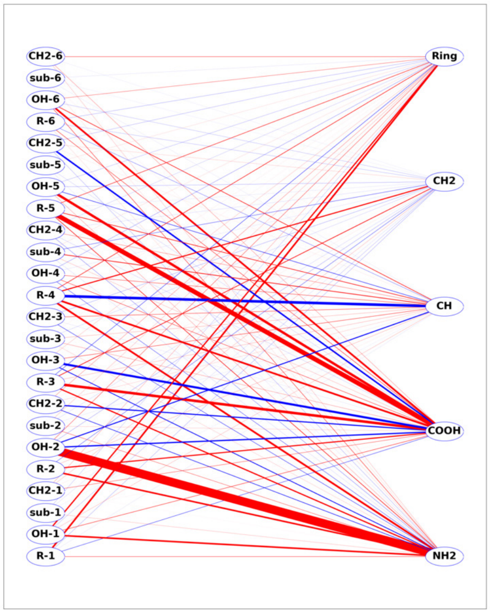

(6) Total *al*-BCX histidineE

Figure S64: The F-SAPT partitioning for *al*-BCX histidineE

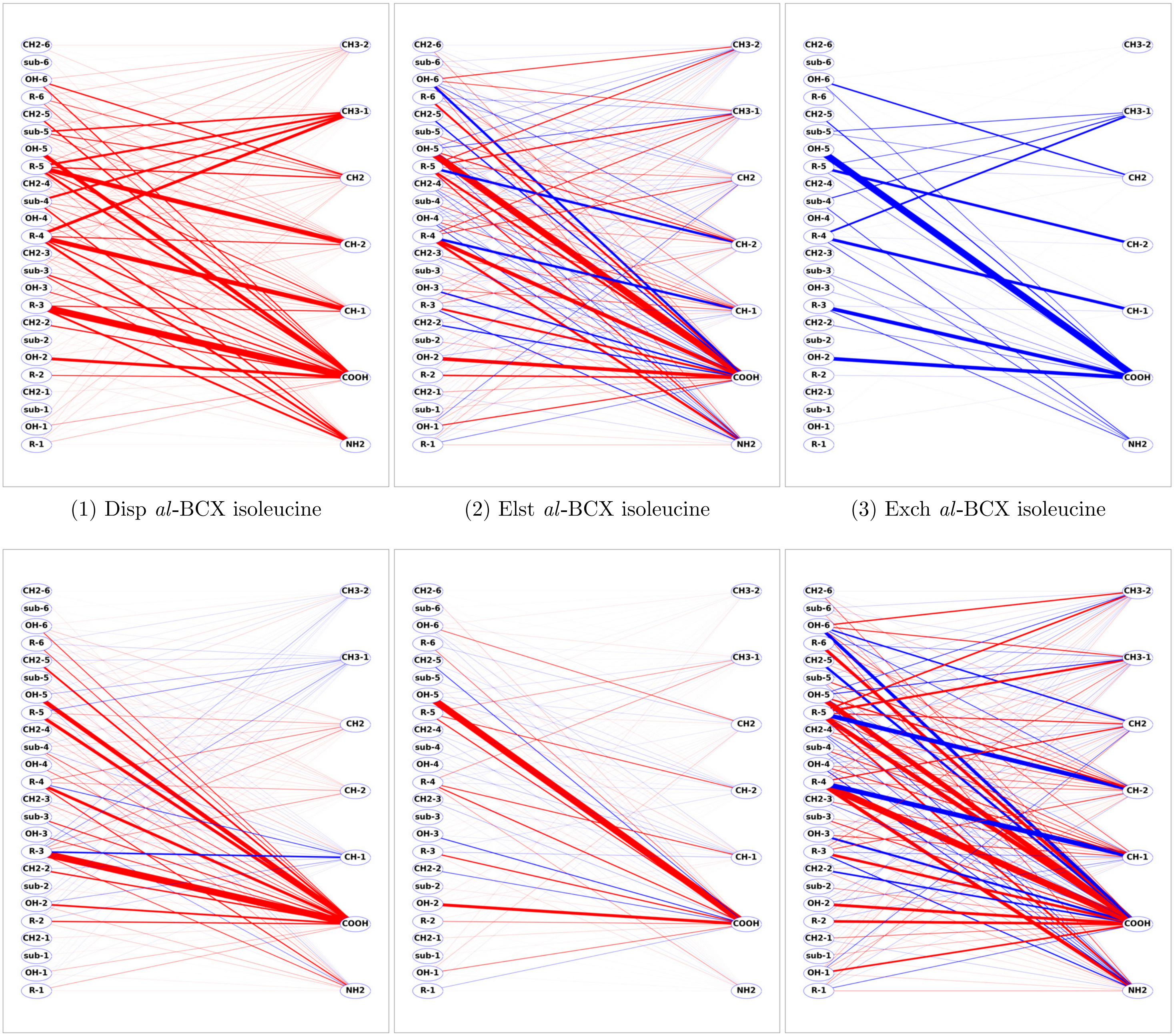

(1) Disp *al*-BCX isoleucine (2) Elst *al*-BCX isoleucine (3) Exch *al*-BCX isoleucine

(4) IndAB *al*-BCX isoleucine (5) IndBA *al*-BCX isoleucine (6) Total *al*-BCX isoleucine

Figure S65: The F-SAPT partitioning for *al*-BCX isoleucine

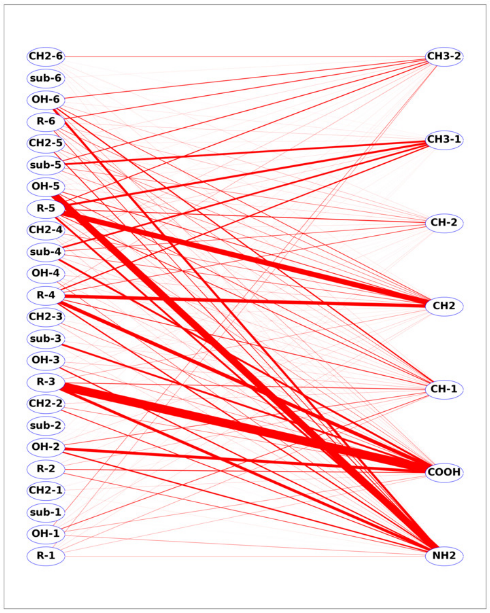

(1) Disp *al*-BCX leucine

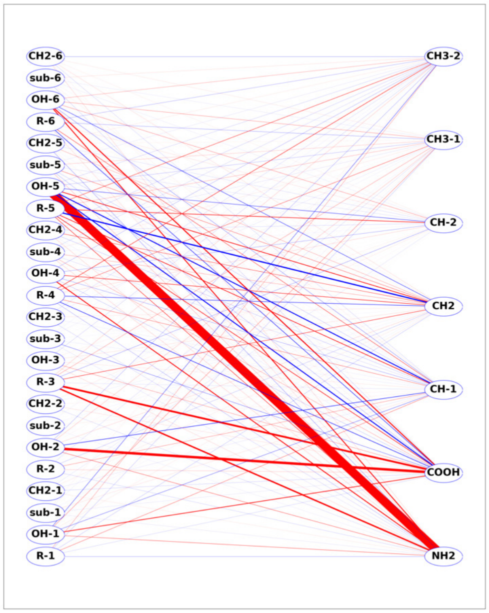

(2) Elst *al*-BCX leucine

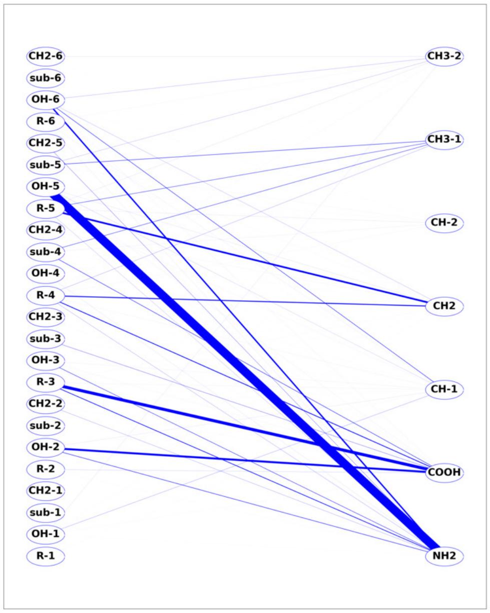

(3) Exch *al*-BCX leucine

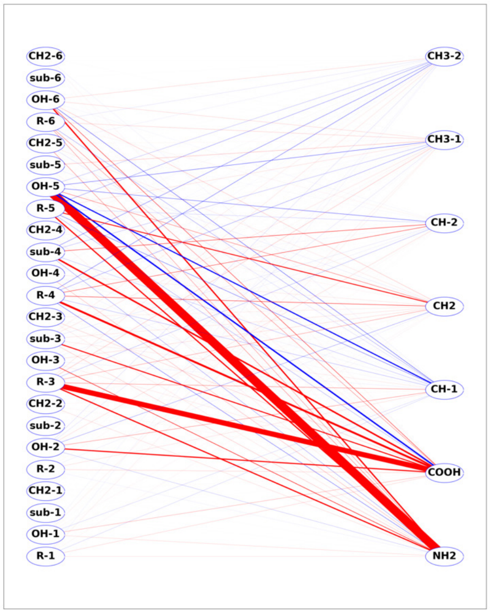

(4) IndAB *al*-BCX leucine

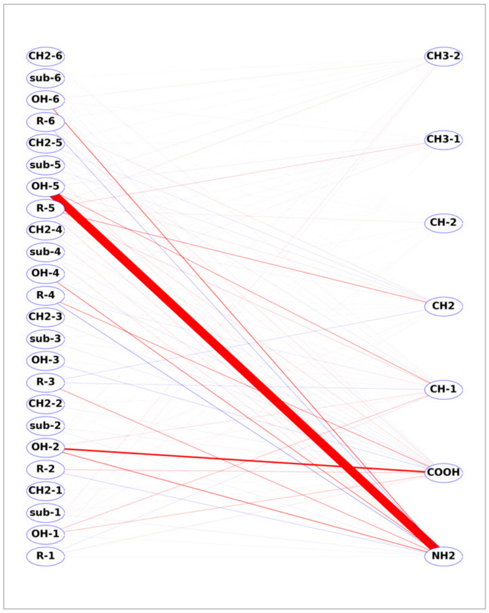

(5) IndBA *al*-BCX leucine

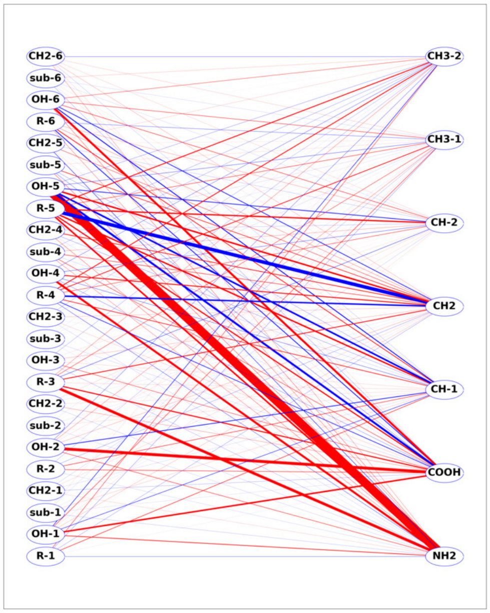

(6) Total *al*-BCX leucine

Figure S66: The F-SAPT partitioning for *al*-BCX leucine

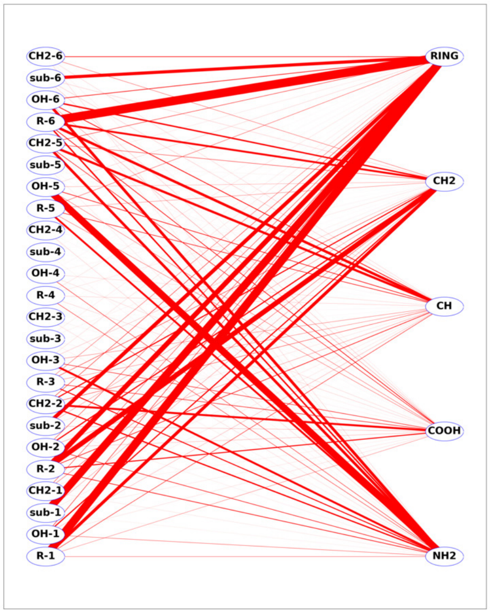

(1) Disp *al*-BCX phenylalanine

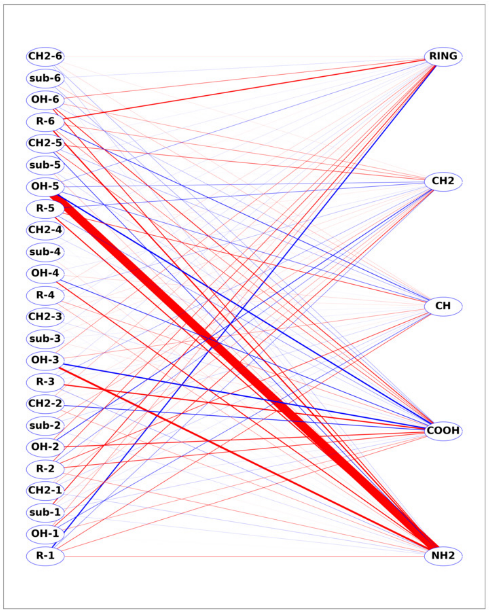

(2) Elst *al*-BCX phenylalanine

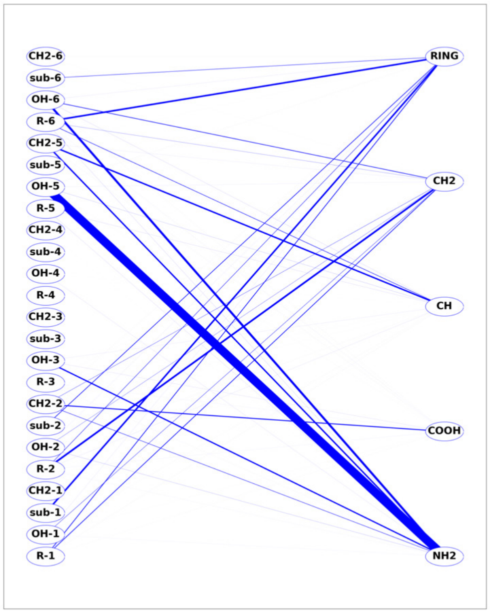

(3) Exch *al*-BCX phenylalanine

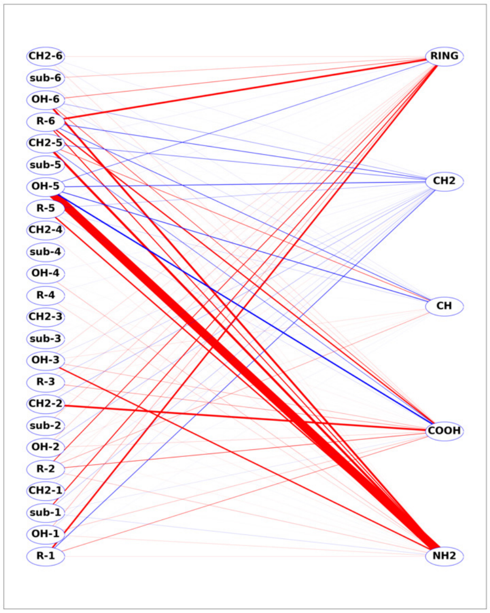

(4) IndAB *al*-BCX phenylalanine

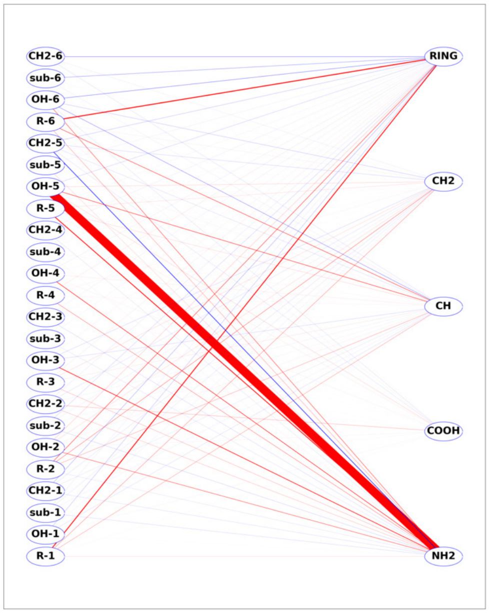

(5) IndBA *al*-BCX phenylalanine

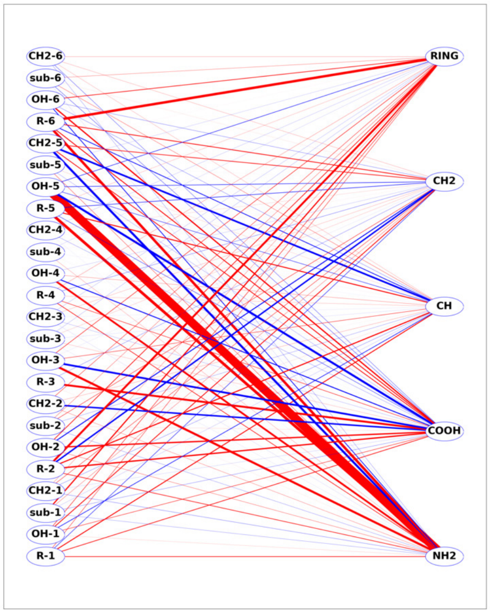

(6) Total *al*-BCX phenylalanine

Figure S67: The F-SAPT partitioning for *al*-BCX phenylalanine

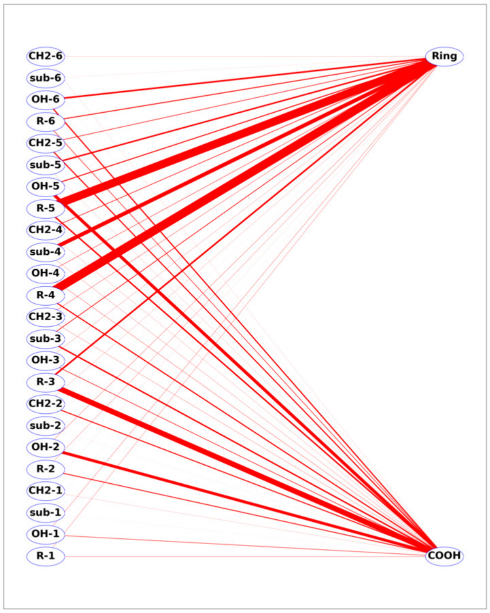

(1) Disp *al*-BCX proline

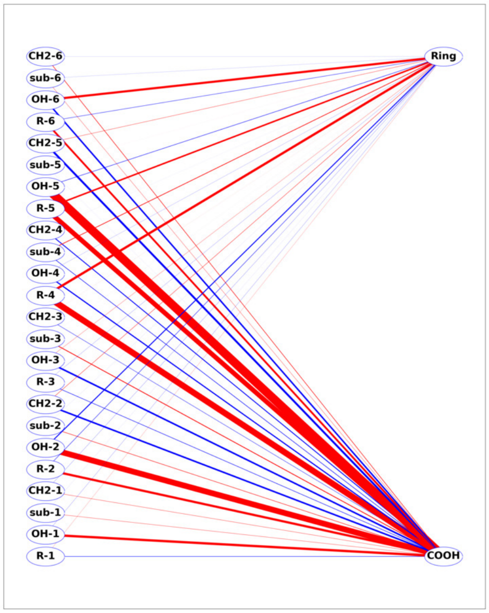

(2) Elst *al*-BCX proline

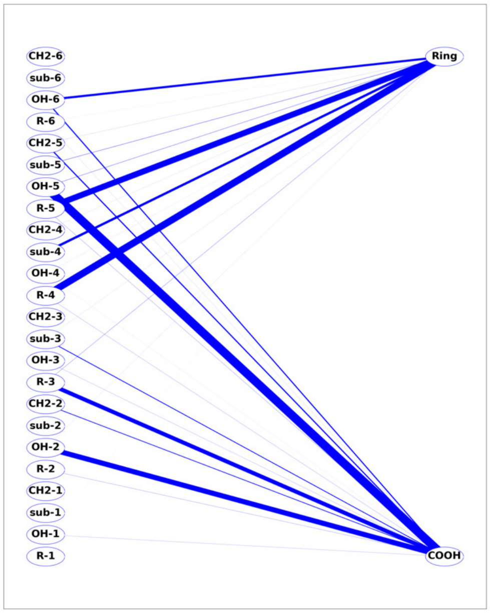

(3) Exch *al*-BCX proline

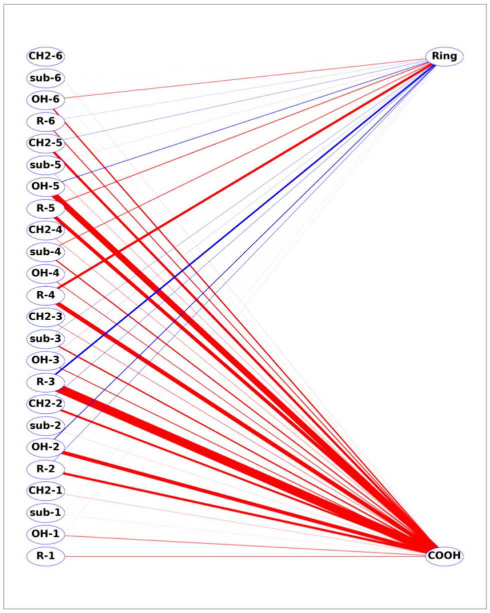

(4) IndAB *al*-BCX proline

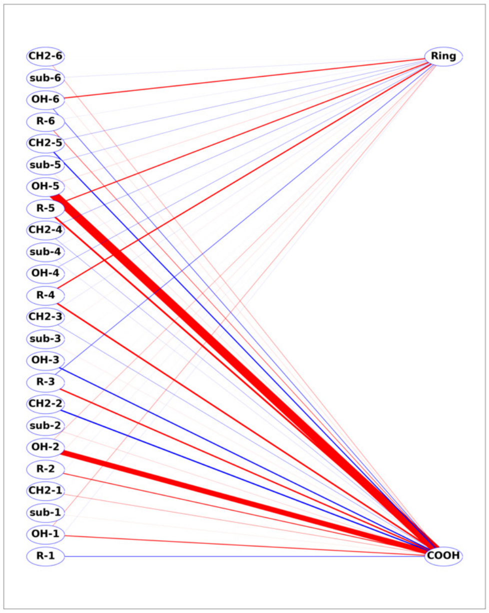

(5) IndBA *al*-BCX proline

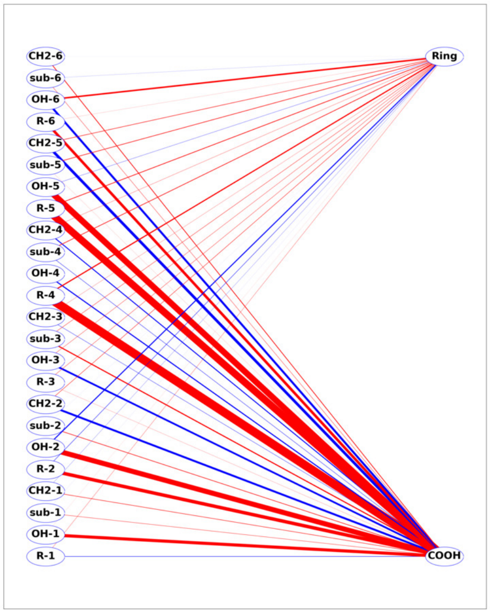

(6) Total *al*-BCX proline

Figure S68: The F-SAPT partitioning for *al*-BCX proline

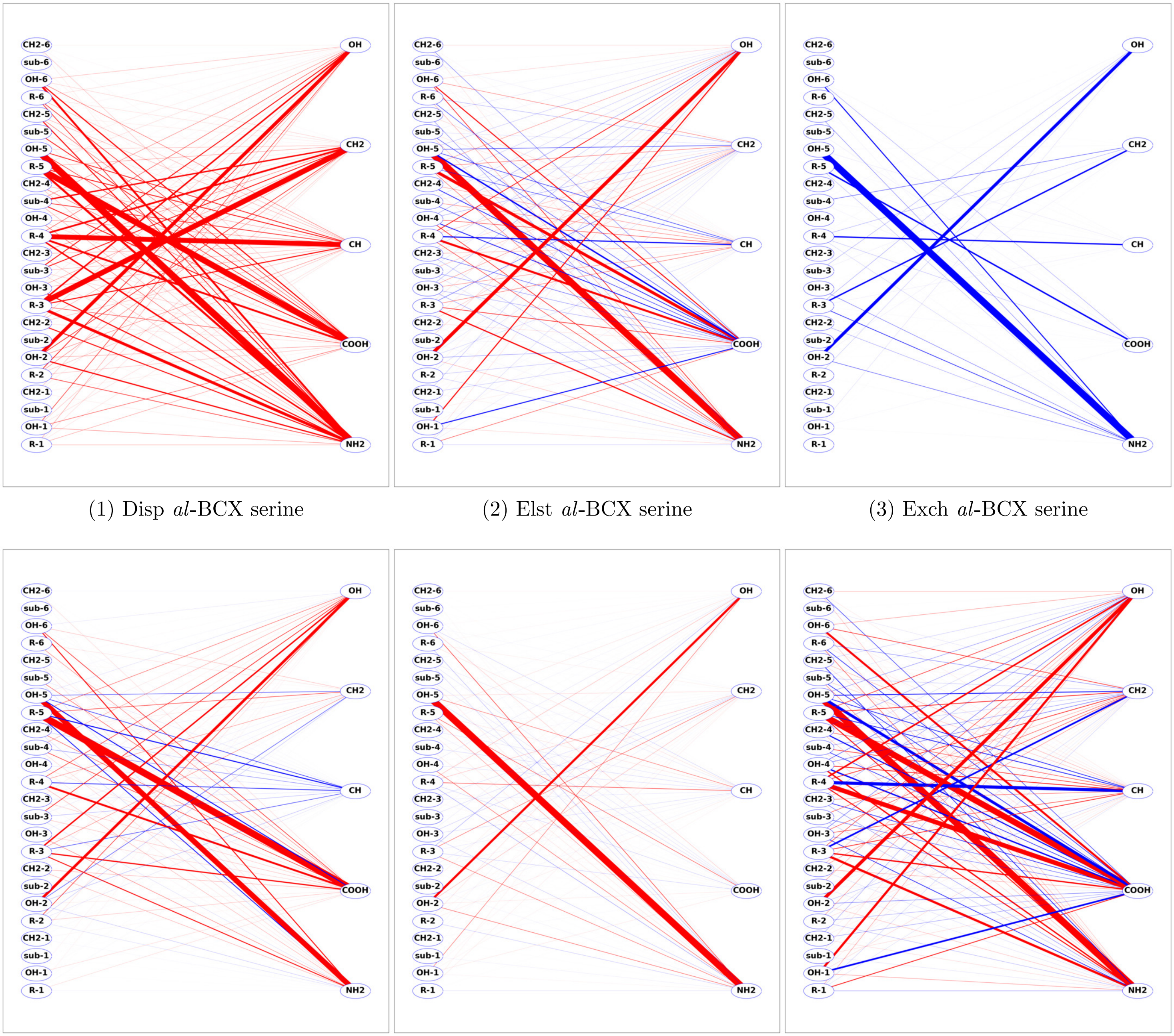

(1) Disp *al*-BCX serine

(2) Elst *al*-BCX serine

(3) Exch *al*-BCX serine

(4) IndAB *al*-BCX serine

(5) IndBA *al*-BCX serine

(6) Total *al*-BCX serine

Figure S69: The F-SAPT partitioning for *al*-BCX serine

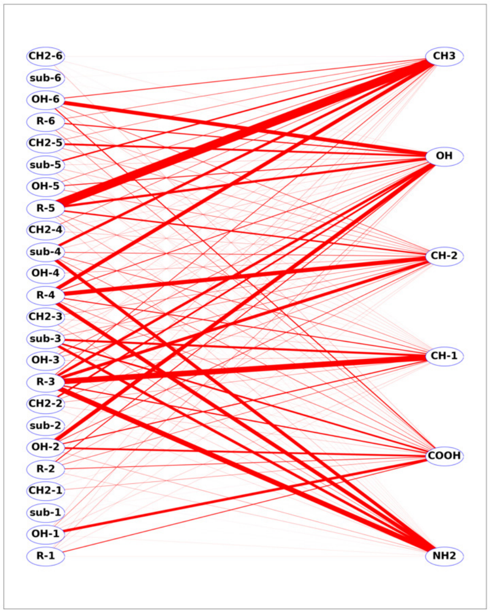

(1) Disp *al*-BCX threonine

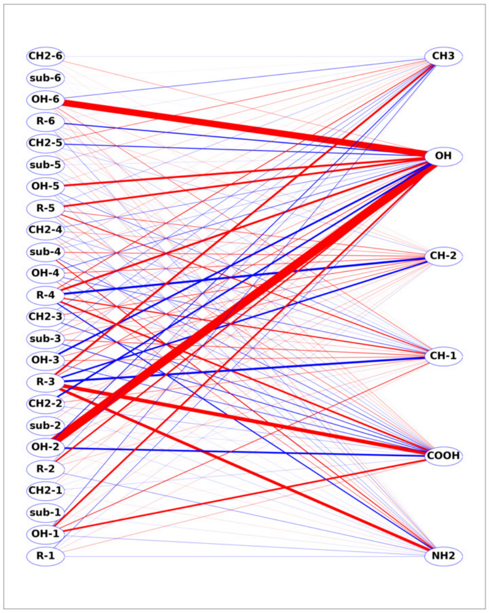

(2) Elst *al*-BCX threonine

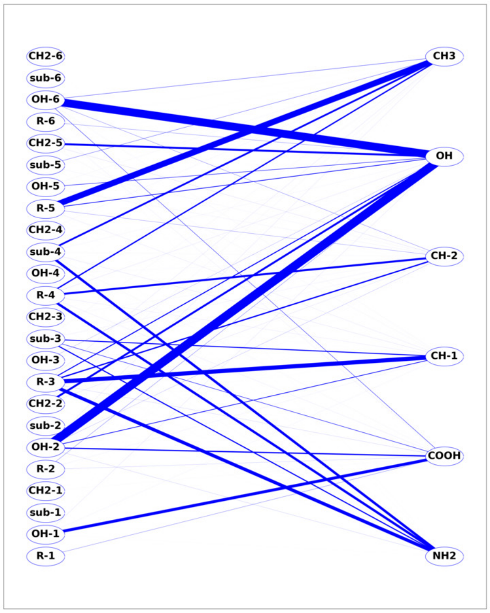

(3) Exch *al*-BCX threonine

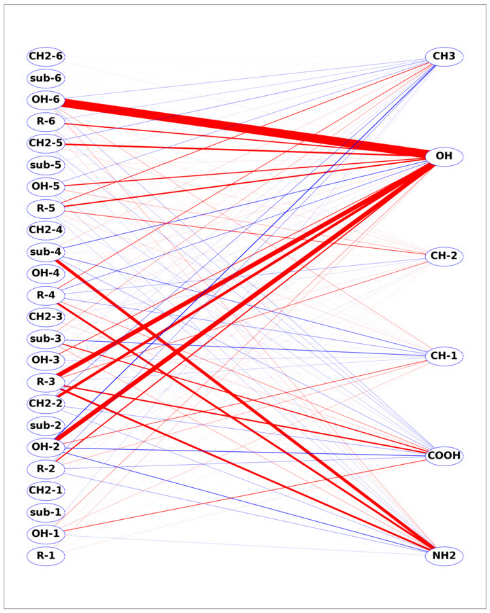

(4) IndAB *al*-BCX threonine

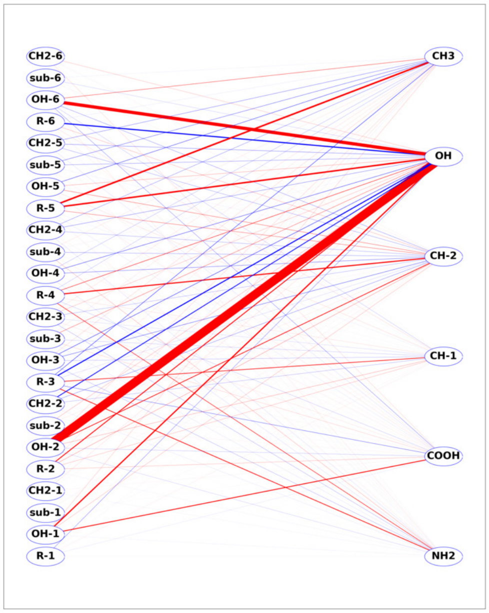

(5) IndBA *al*-BCX threonine

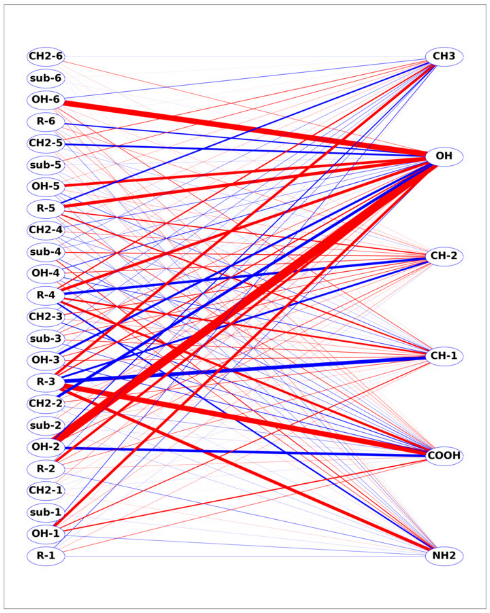

(6) Total *al*-BCX threonine

Figure S70: The F-SAPT partitioning for *al*-BCX threonine

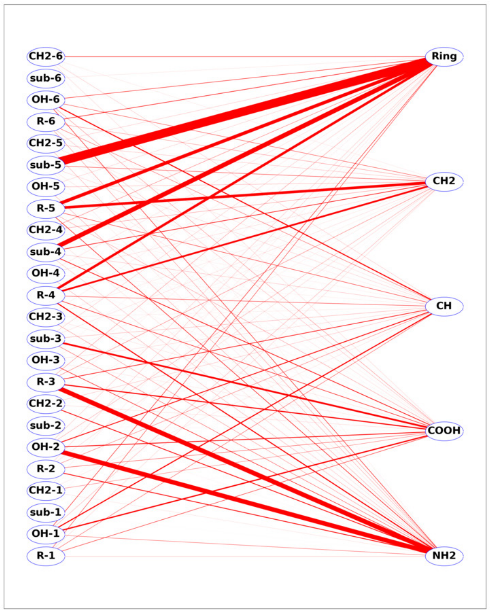

(1) Disp *al*-BCX tryptophan

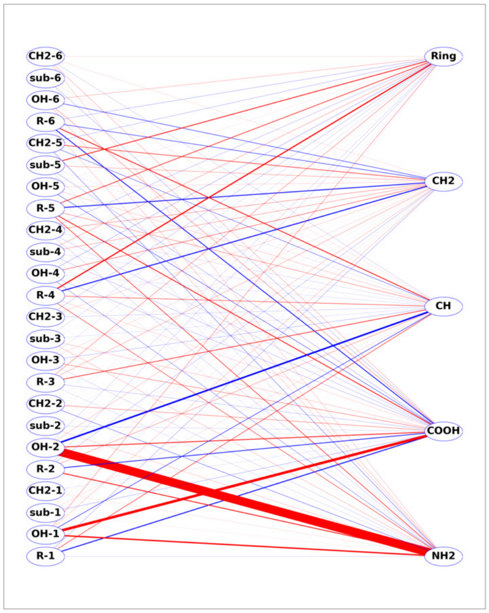

(2) Elst *al*-BCX tryptophan

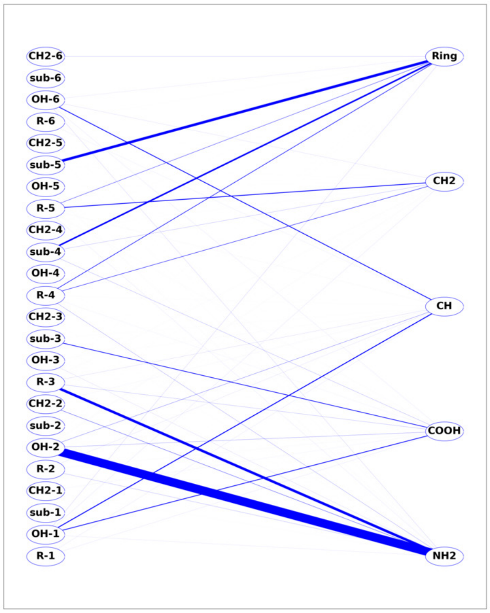

(3) Exch *al*-BCX tryptophan

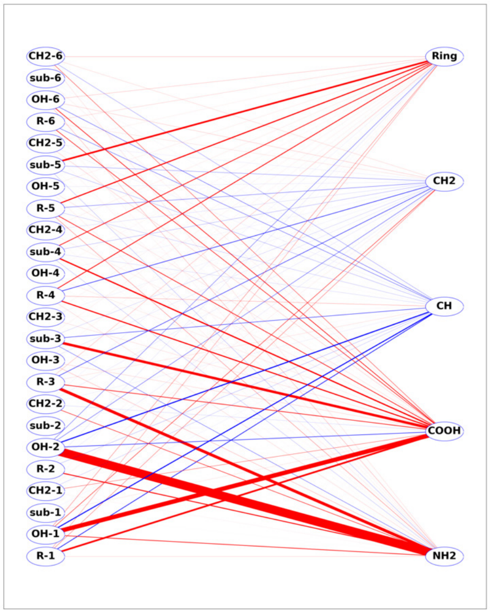

(4) IndAB *al*-BCX tryptophan

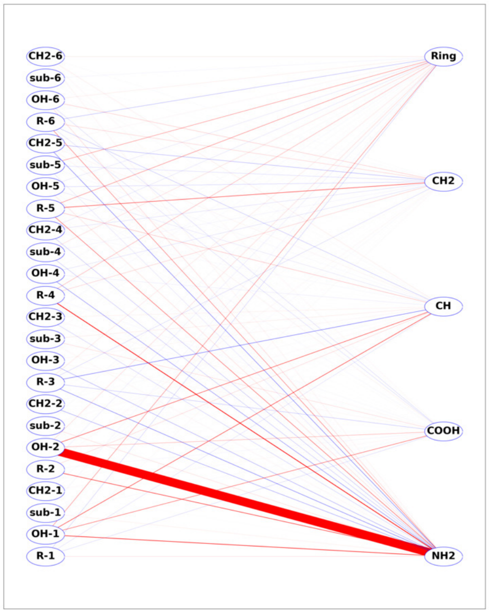

(5) IndBA *al*-BCX tryptophan

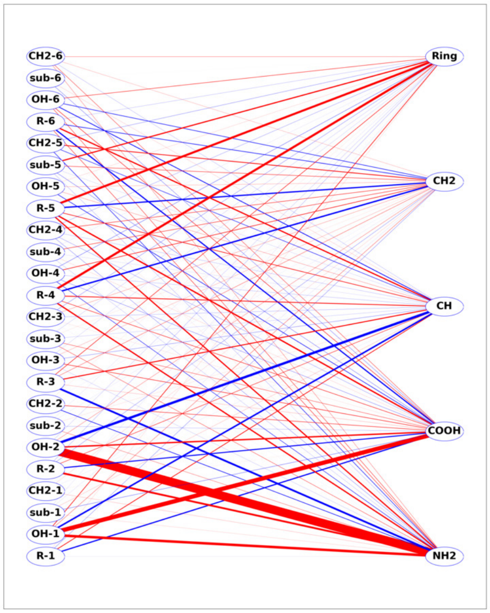

(6) Total *al*-BCX tryptophan

Figure S71: The F-SAPT partitioning for *al*-BCX tryptophan

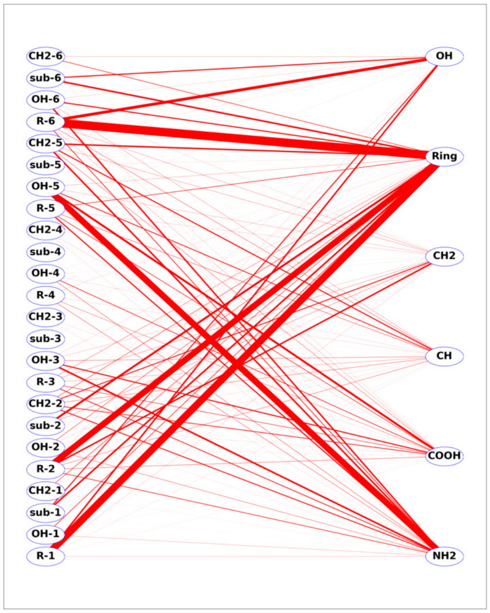

(1) Disp *al*-BCX tyrosine

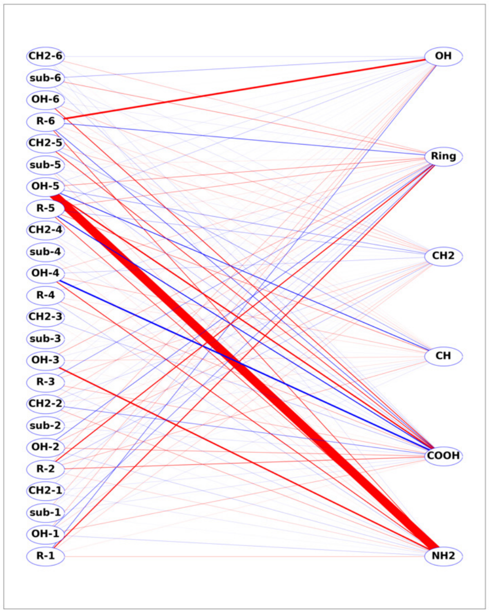

(2) Elst *al*-BCX tyrosine

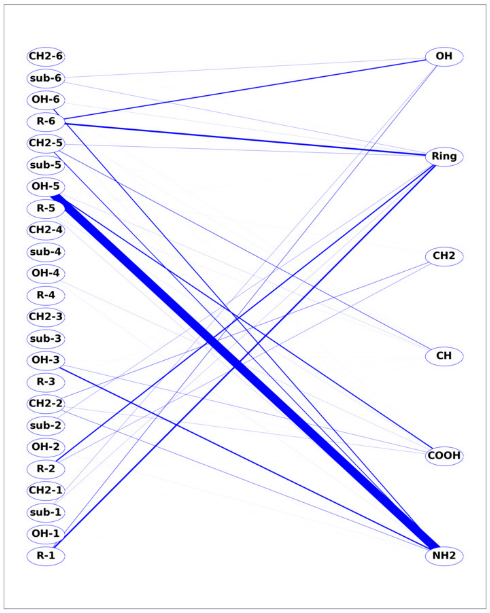

(3) Exch *al*-BCX tyrosine

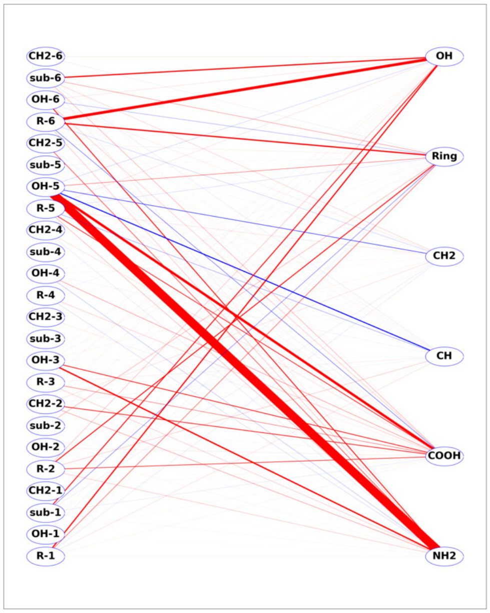

(4) IndAB *al*-BCX tyrosine

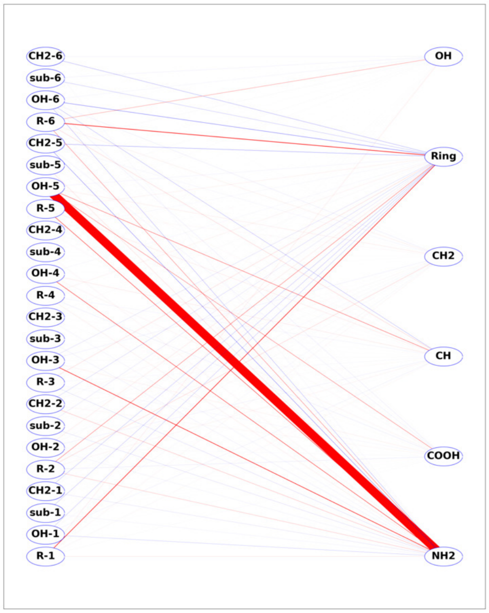

(5) IndBA *al*-BCX tyrosine

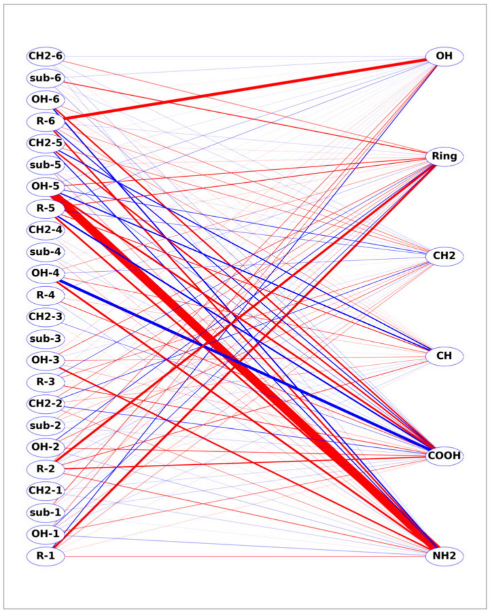

(6) Total *al*-BCX tyrosine

Figure S72: The F-SAPT partitioning for *al*-BCX tyrosine

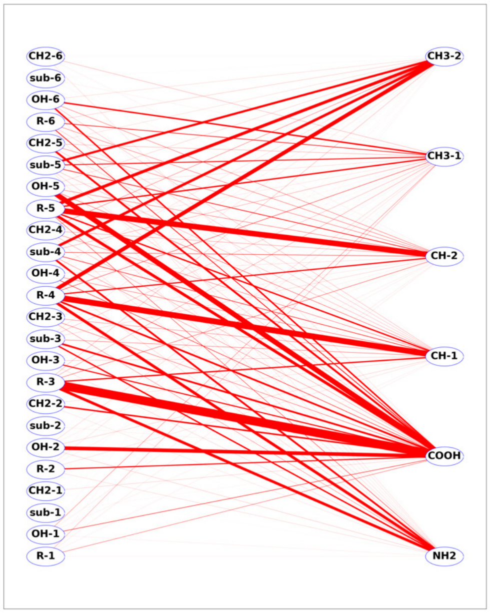

(1) Disp *al*-BCX valine

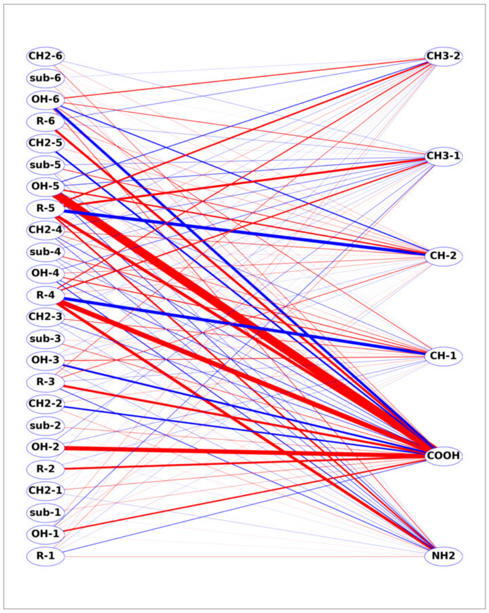

(2) Elst *al*-BCX valine

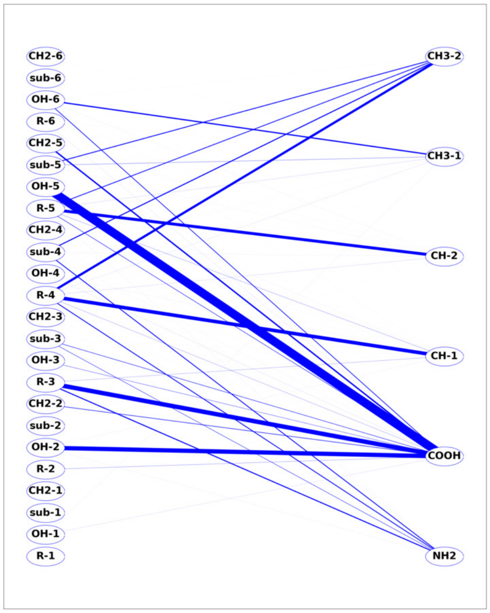

(3) Exch *al*-BCX valine

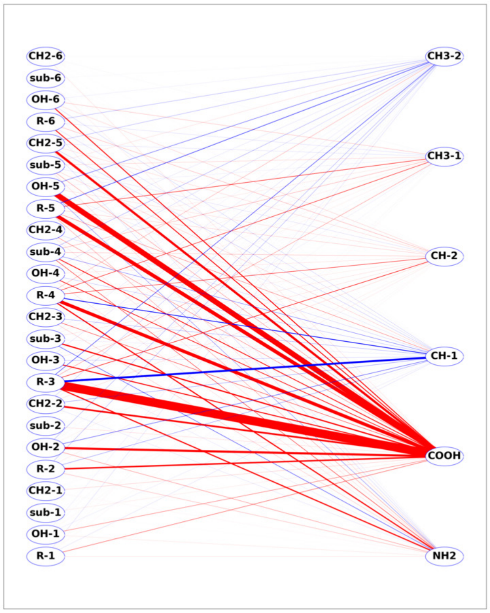

(4) IndAB *al*-BCX valine

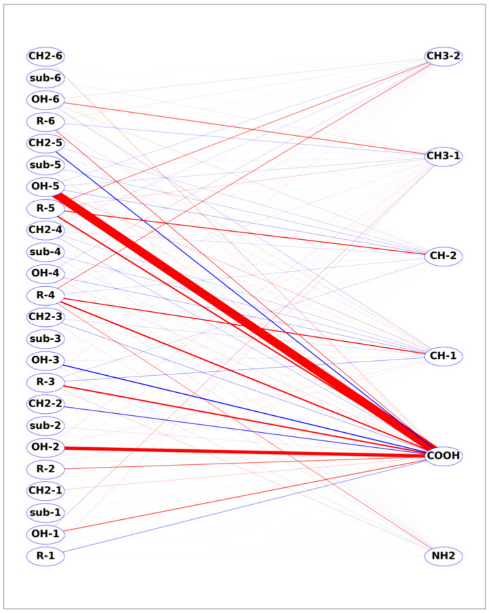

(5) IndBA *al*-BCX valine

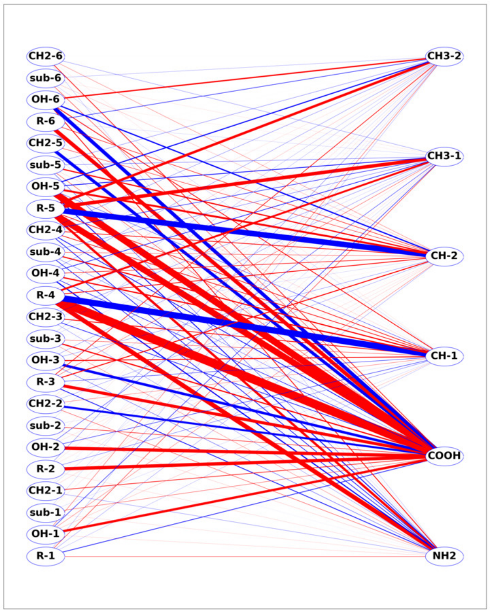

(6) Total *al*-BCX valine

Figure S73: The F-SAPT partitioning for *al*-BCX valine

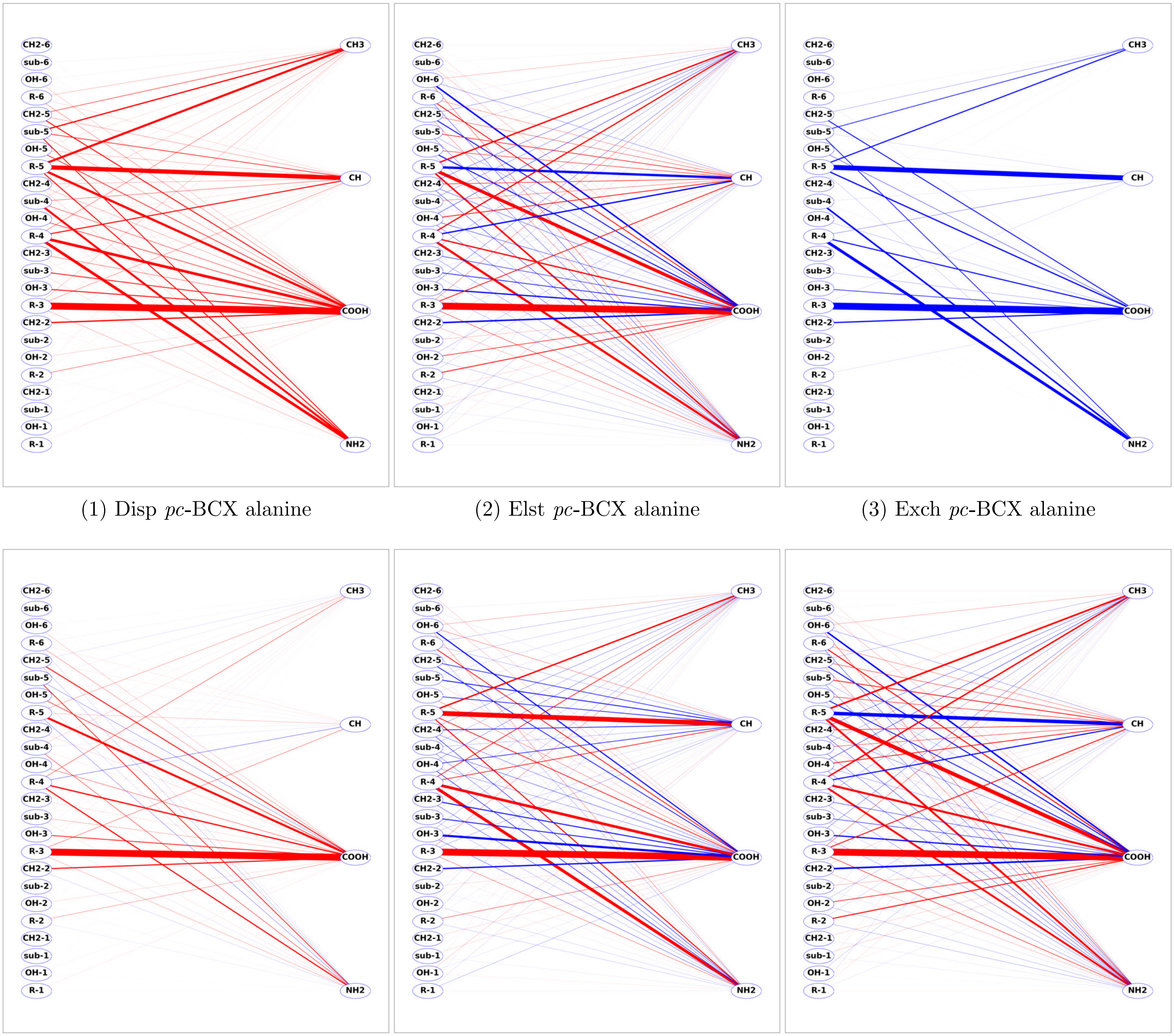

(1) Disp *pc*-BCX alanine

(2) Elst *pc*-BCX alanine

(3) Exch *pc*-BCX alanine

(4) IndAB *pc*-BCX alanine

(5) IndBA *pc*-BCX alanine

(6) Total *pc*-BCX alanine

Figure S74: The F-SAPT partitioning for *pc*-BCX alanine

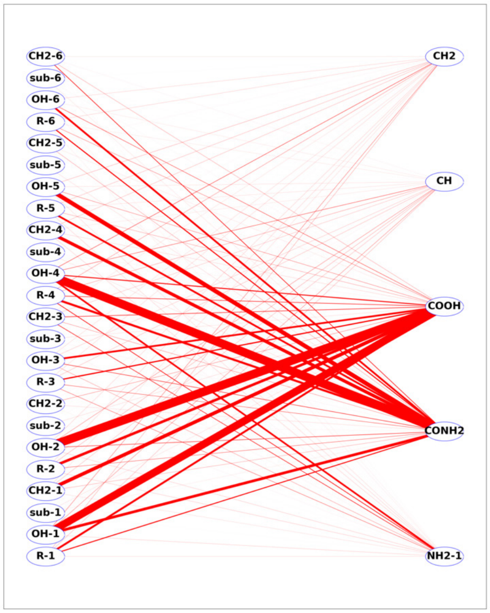

(1) Disp *pc*-BCX asparagine

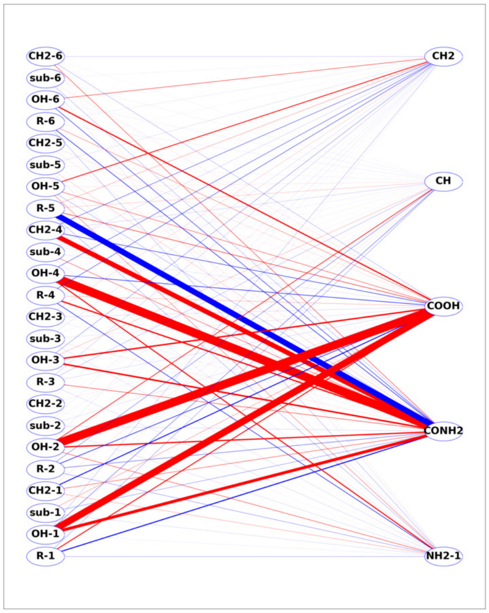

(2) Elst *pc*-BCX asparagine

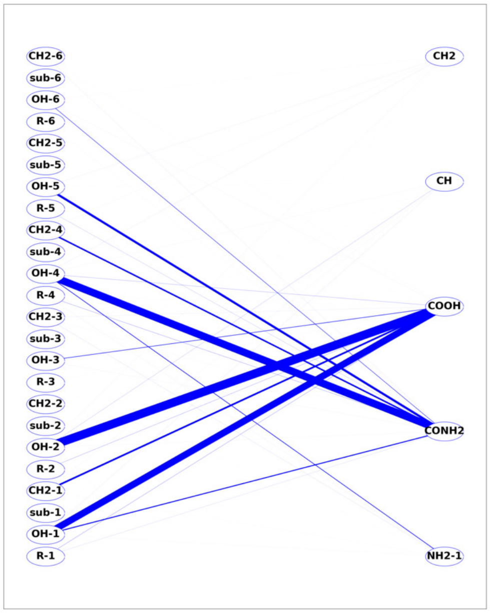

(3) Exch *pc*-BCX asparagine

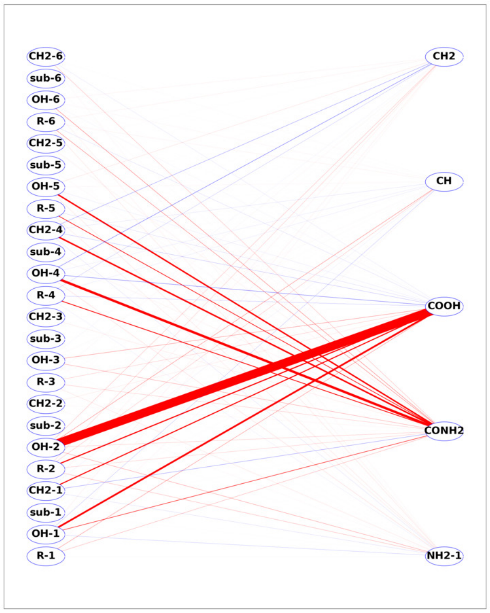

(4) IndAB *pc*-BCX asparagine

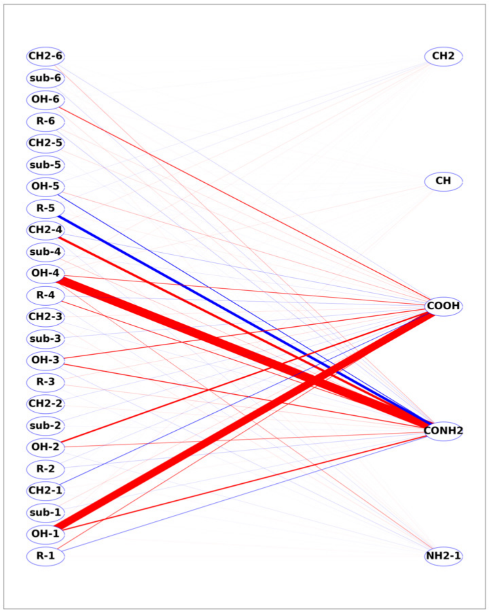

(5) IndBA *pc*-BCX asparagine

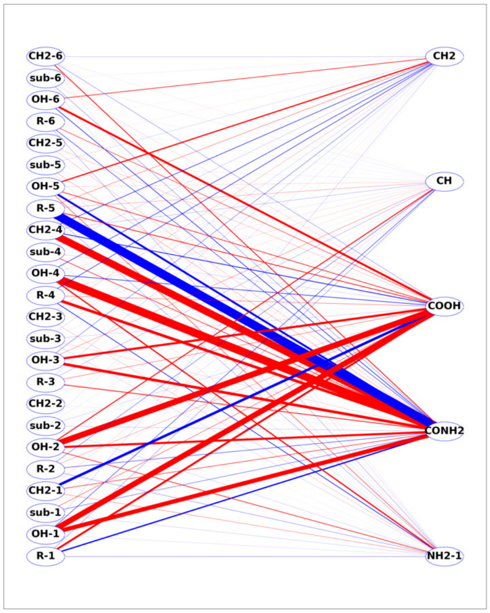

(6) Total *pc*-BCX asparagine

Figure S75: The F-SAPT partitioning for *pc*-BCX asparagine

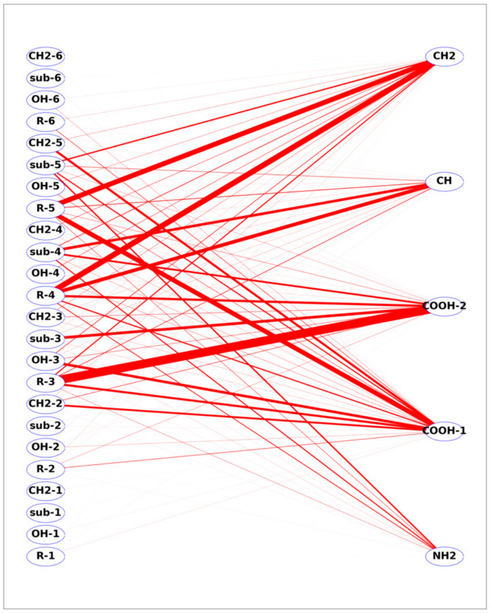

(1) Disp *pc*-BCX aspartate

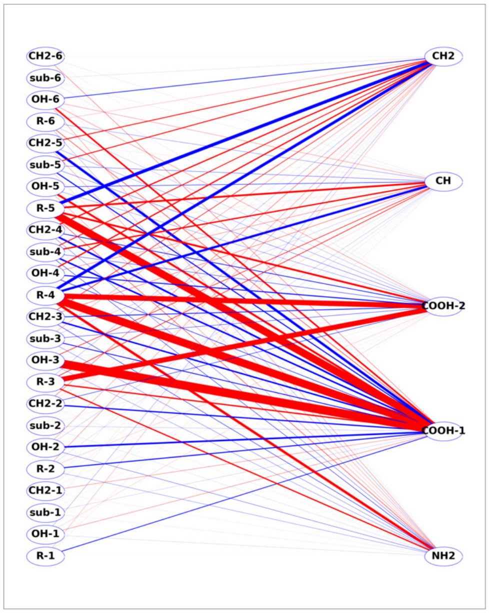

(2) Elst *pc*-BCX aspartate

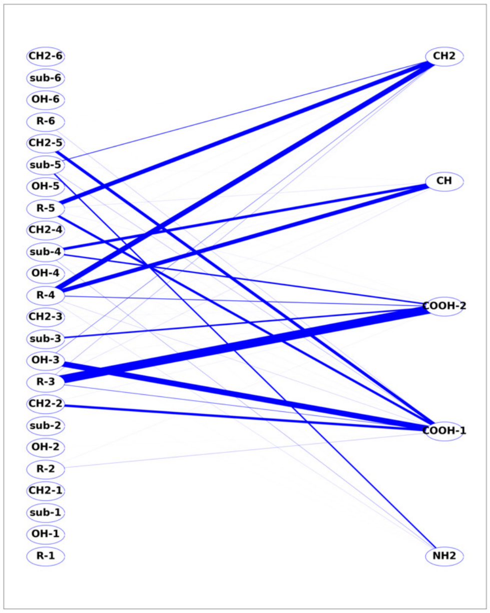

(3) Exch *pc*-BCX aspartate

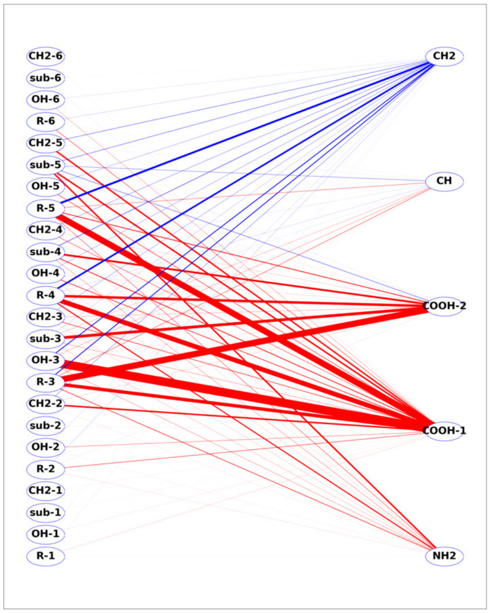

(4) IndAB *pc*-BCX aspartate

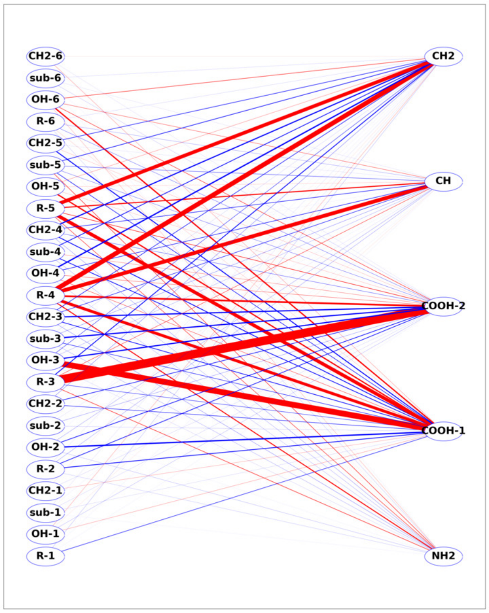

(5) IndBA *pc*-BCX aspartate

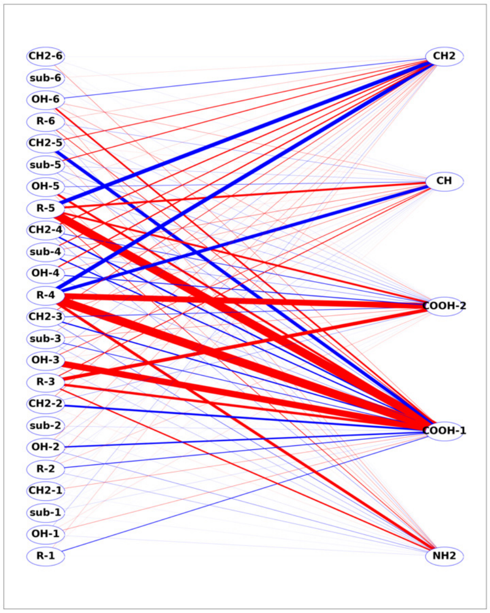

(6) Total *pc*-BCX aspartate

Figure S76: The F-SAPT partitioning for *pc*-BCX aspartate

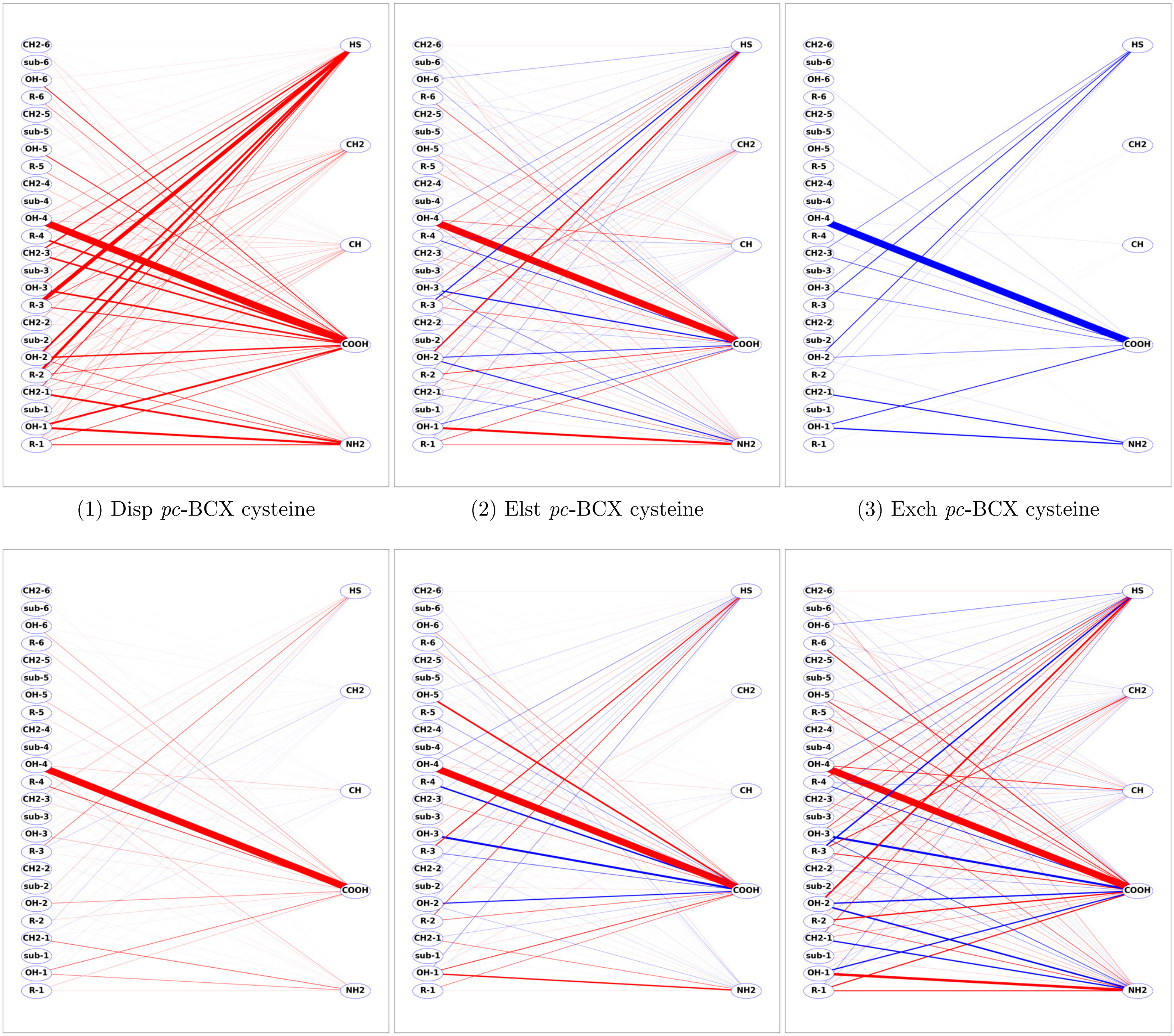

(1) Disp *pc*-BCX cysteine

(2) Elst *pc*-BCX cysteine

(3) Exch *pc*-BCX cysteine

(4) IndAB *pc*-BCX cysteine

(5) IndBA *pc*-BCX cysteine

(6) Total *pc*-BCX cysteine

Figure S77: The F-SAPT partitioning for *pc*-BCX cysteine

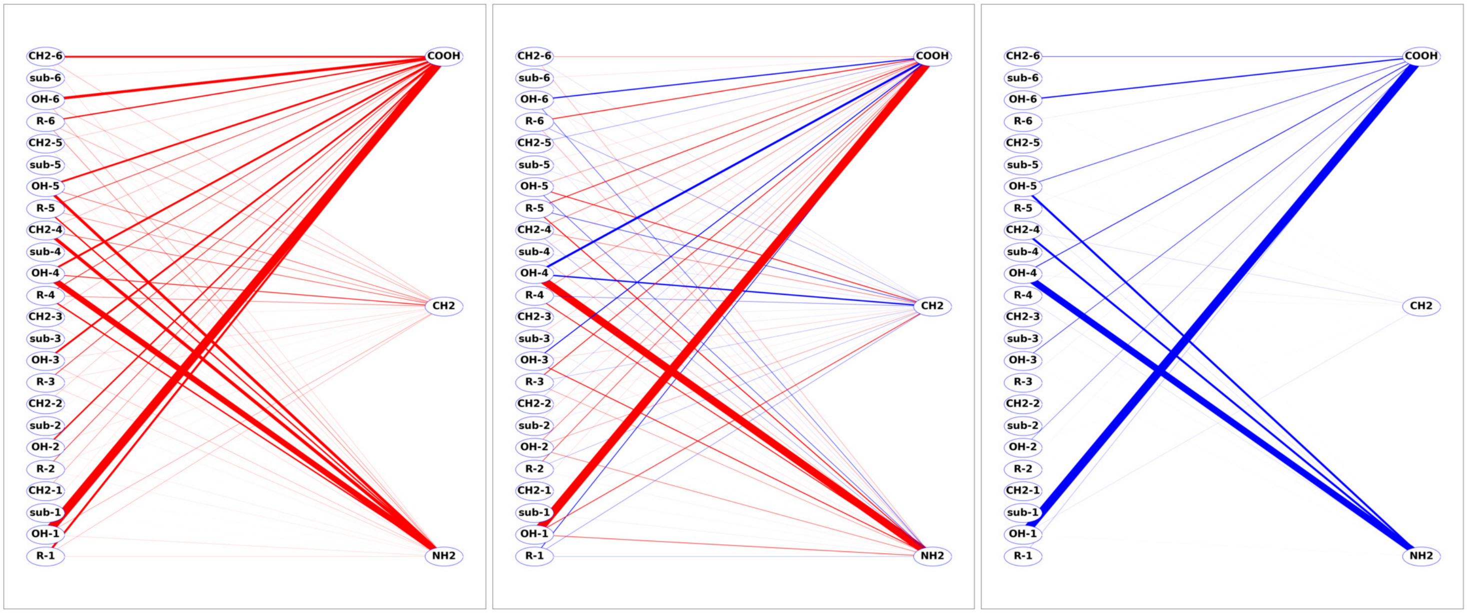

(1) Disp *pc*-BCX glycine (2) Elst *pc*-BCX glycine (3) Exch *pc*-BCX glycine

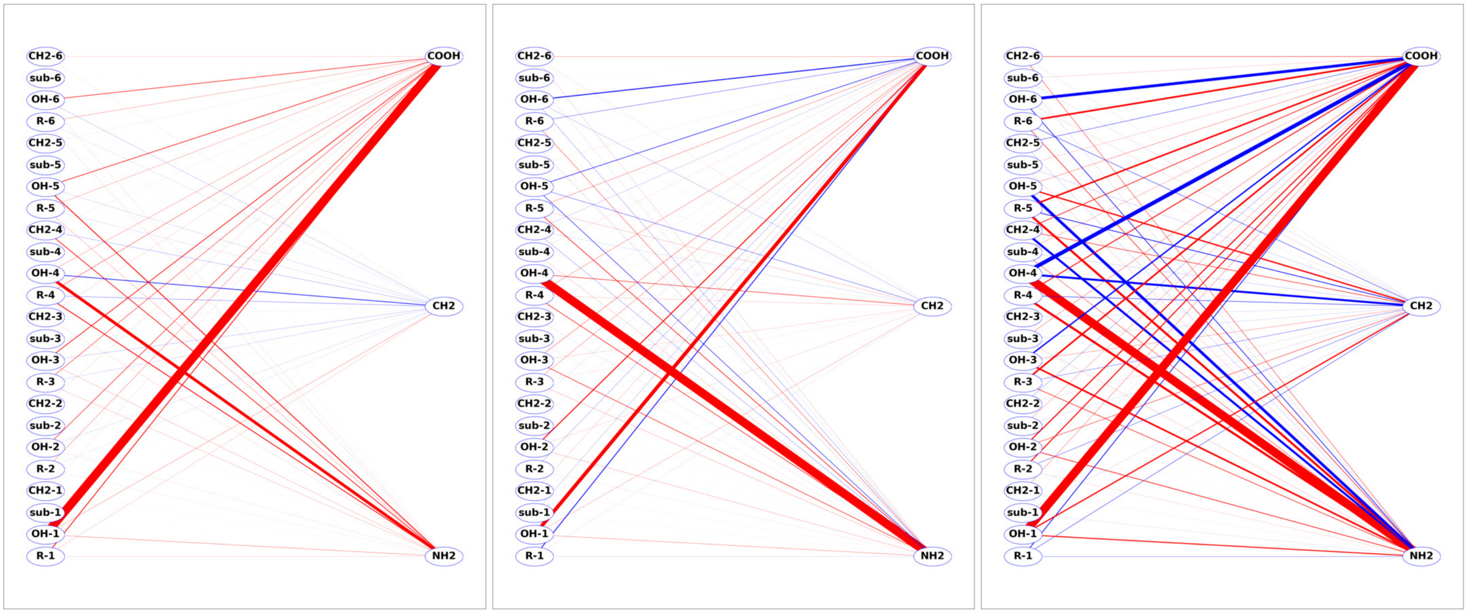

(4) IndAB *pc*-BCX glycine (5) IndBA *pc*-BCX glycine (6) Total *pc*-BCX glycine

Figure S78: The F-SAPT partitioning for *pc*-BCX glycine

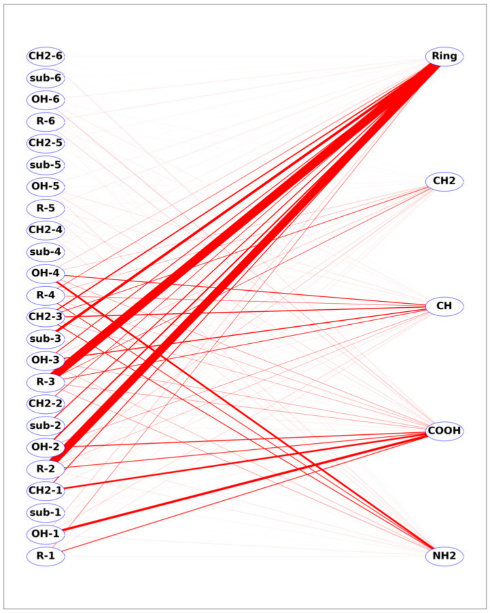

(1) Disp *pc*-BCX histidineD

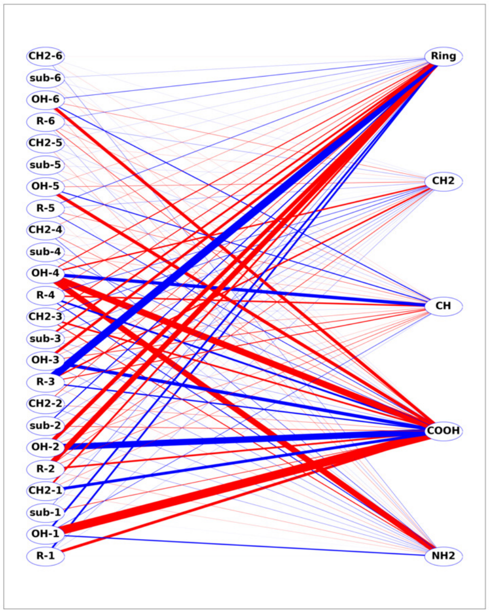

(2) Elst *pc*-BCX histidineD

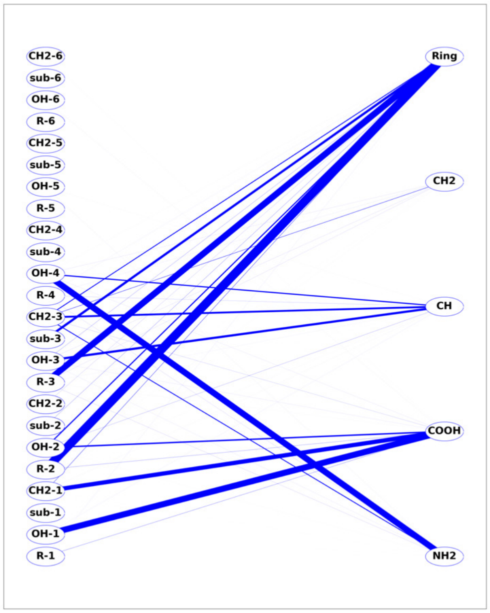

(3) Exch *pc*-BCX histidineD

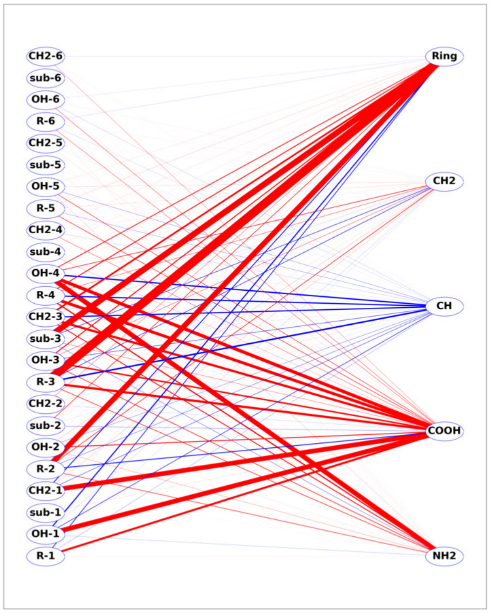

(4) IndAB *pc*-BCX histidineD

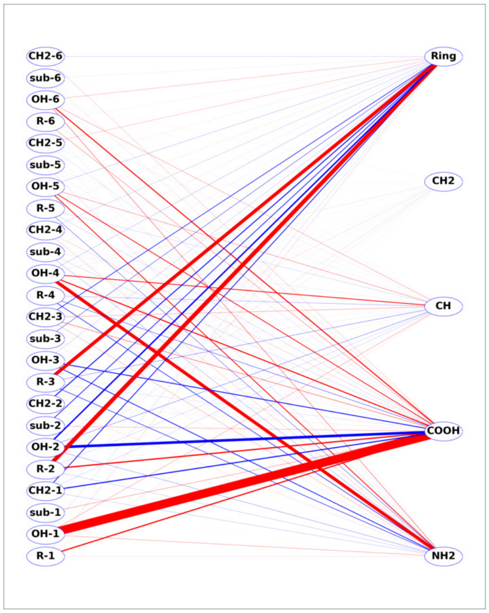

(5) IndBA *pc*-BCX histidineD

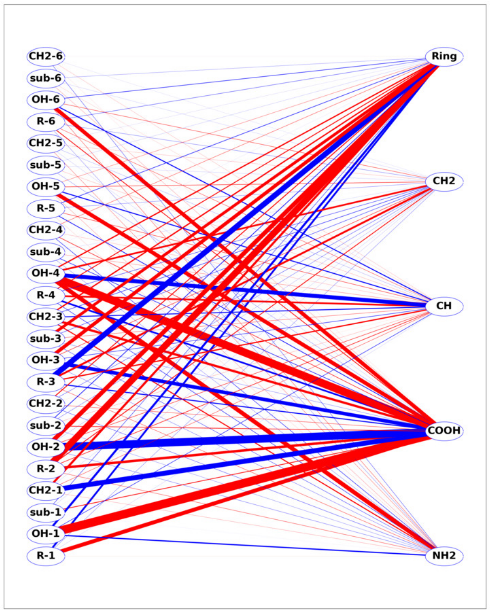

(6) Total *pc*-BCX histidineD

Figure S79: The F-SAPT partitioning for *pc*-BCX histidineD

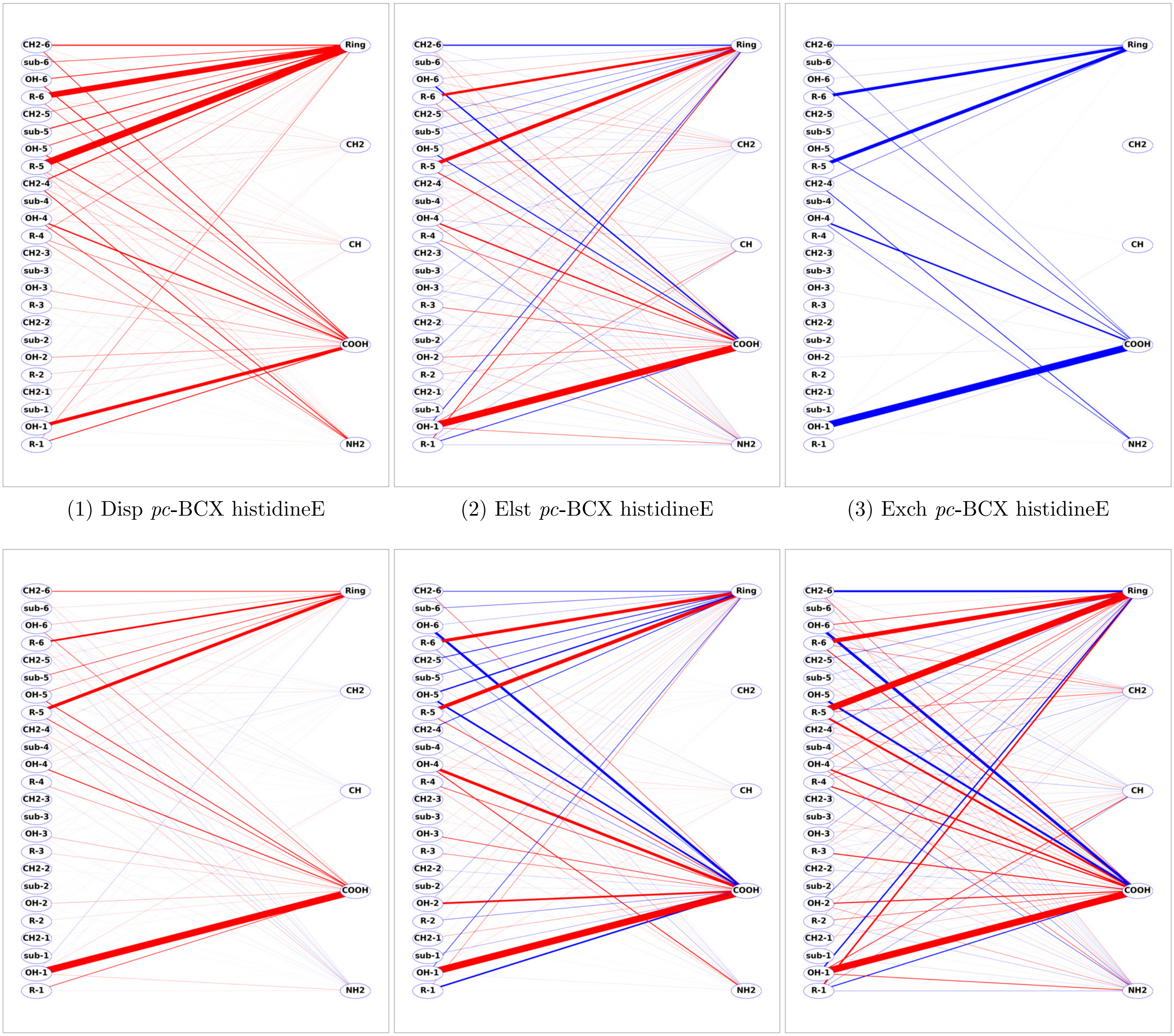

(1) Disp *pc*-BCX histidineE (2) Elst *pc*-BCX histidineE (3) Exch *pc*-BCX histidineE

(4) IndAB *pc*-BCX histidineE (5) IndBA *pc*-BCX histidineE (6) Total *pc*-BCX histidineE

Figure S80: The F-SAPT partitioning for *pc*-BCX histidineE

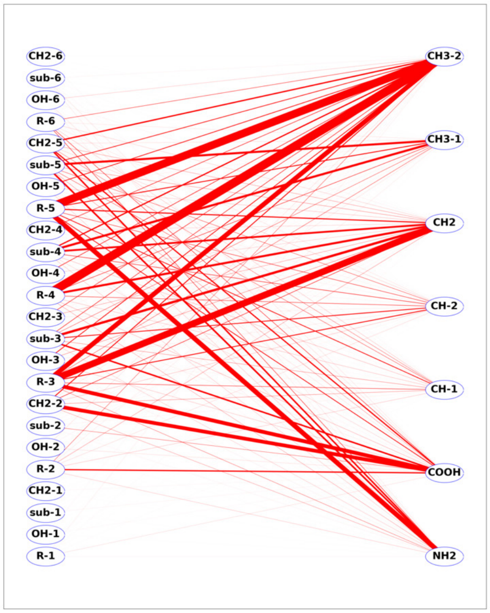

(1) Disp *pc*-BCX isoleucine

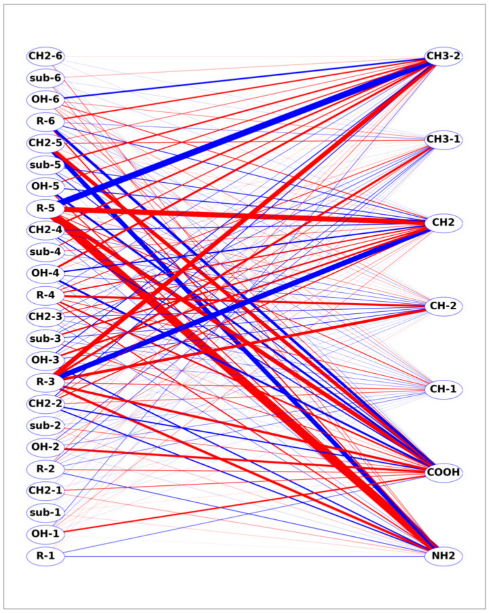

(2) Elst *pc*-BCX isoleucine

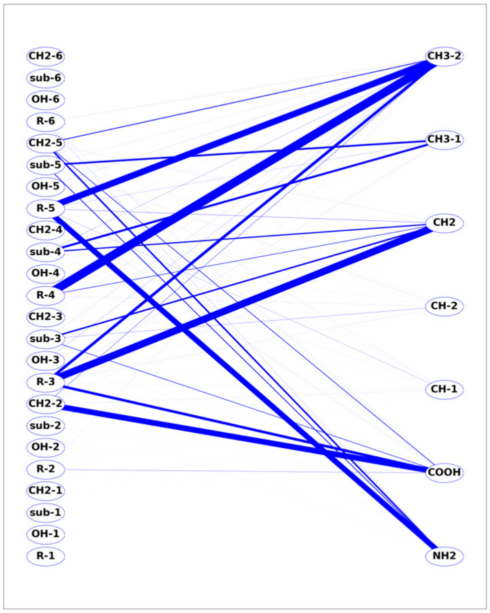

(3) Exch *pc*-BCX isoleucine

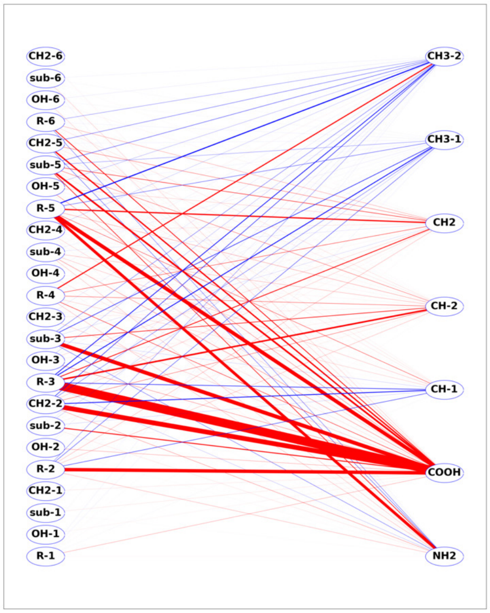

(4) IndAB *pc*-BCX isoleucine

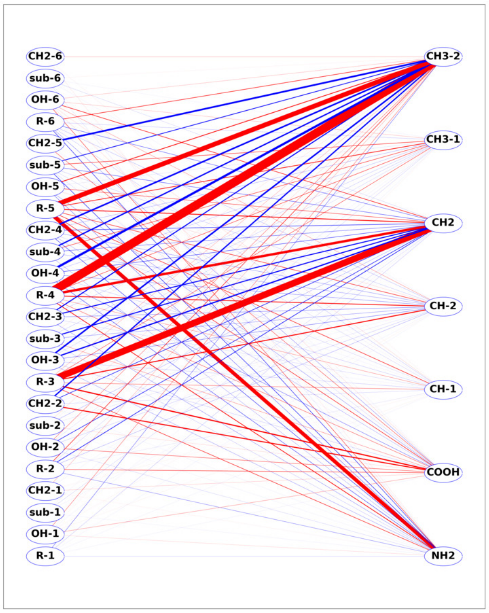

(5) IndBA *pc*-BCX isoleucine

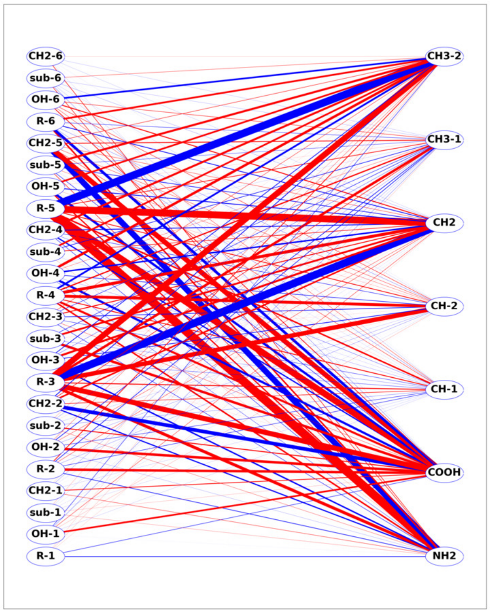

(6) Total *pc*-BCX isoleucine

Figure S81: The F-SAPT partitioning for *pc*-BCX isoleucine

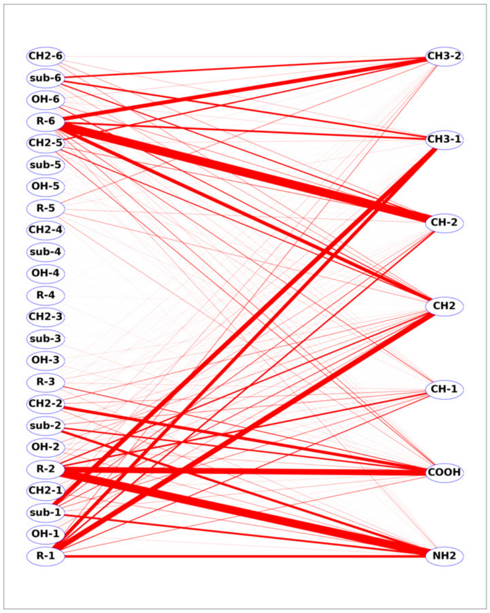

(1) Disp *pc*-BCX leucine

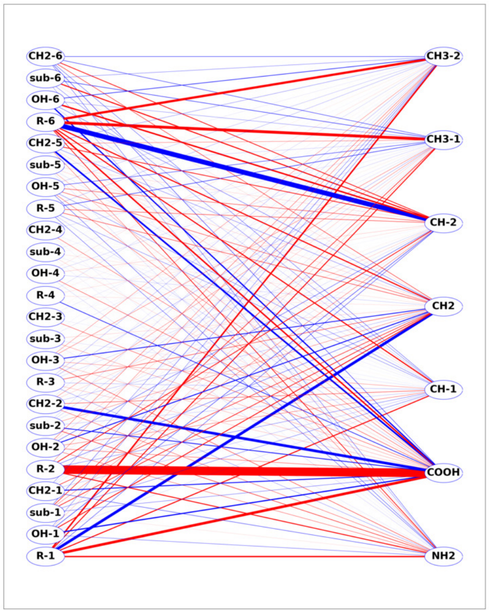

(2) Elst *pc*-BCX leucine

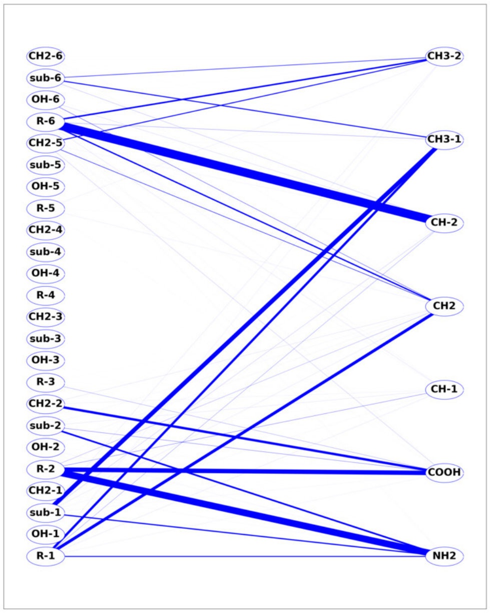

(3) Exch *pc*-BCX leucine

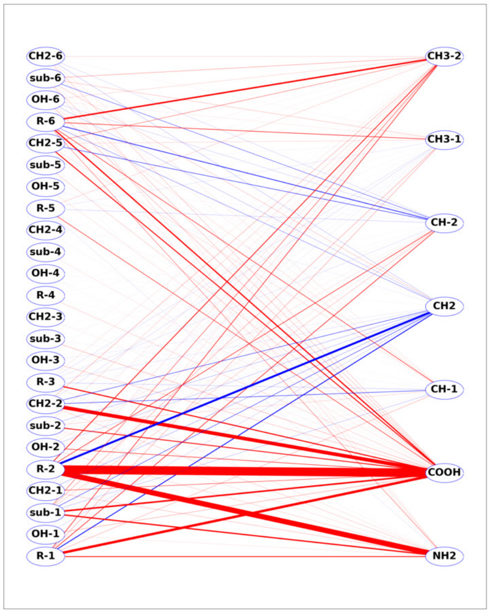

(4) IndAB *pc*-BCX leucine

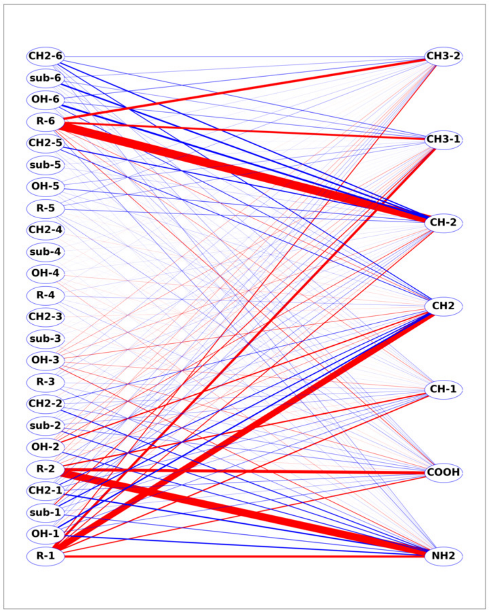

(5) IndBA *pc*-BCX leucine

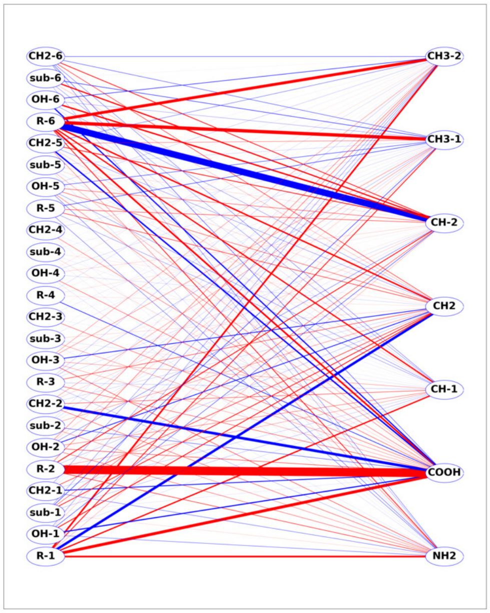

(6) Total *pc*-BCX leucine

Figure S82: The F-SAPT partitioning for *pc*-BCX leucine

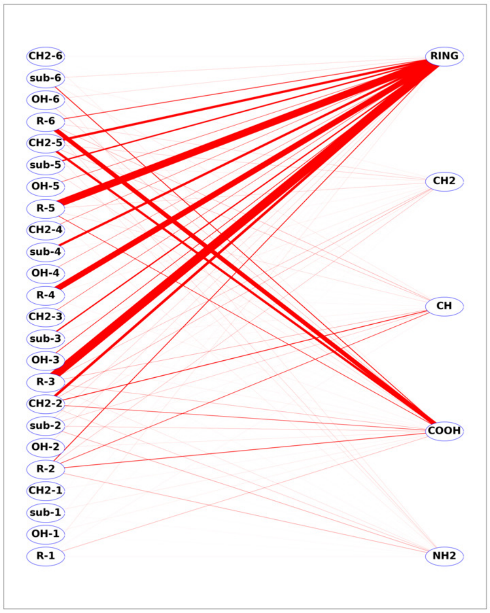

(1) Disp *pc*-BCX phenylalanine

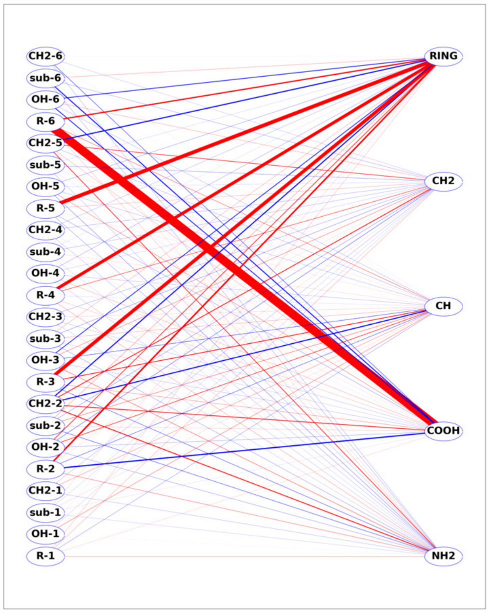

(2) Elst *pc*-BCX phenylalanine

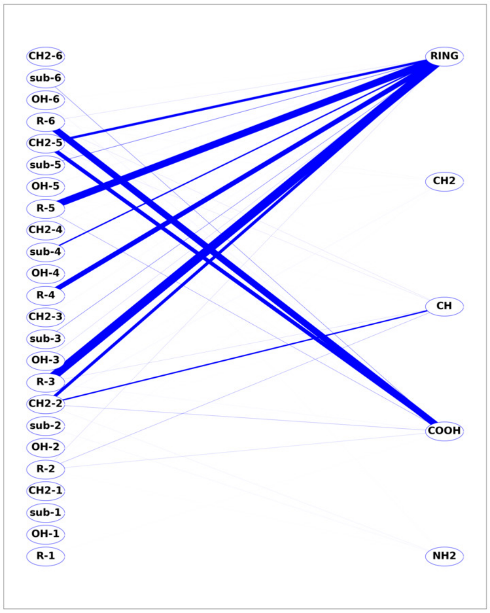

(3) Exch *pc*-BCX phenylalanine

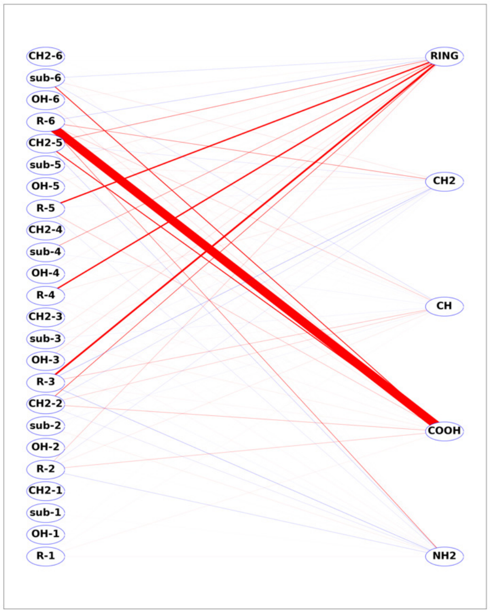

(4) IndAB *pc*-BCX phenylalanine

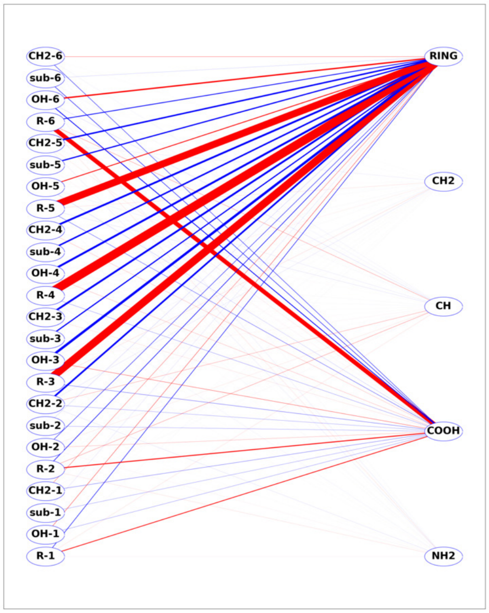

(5) IndBA *pc*-BCX phenylalanine

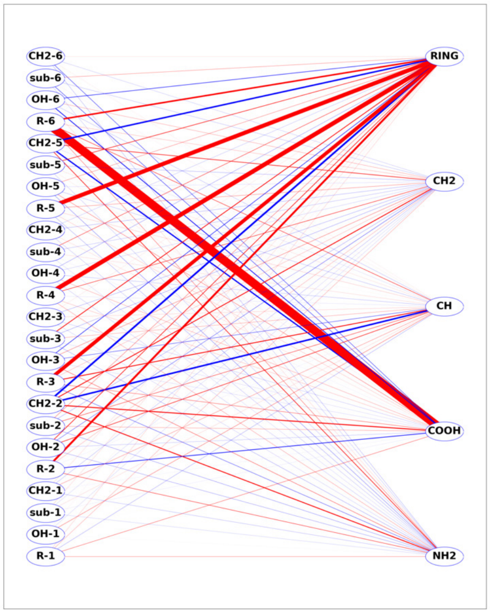

(6) Total *pc*-BCX phenylalanine

Figure S83: The F-SAPT partitioning for *pc*-BCX phenylalanine

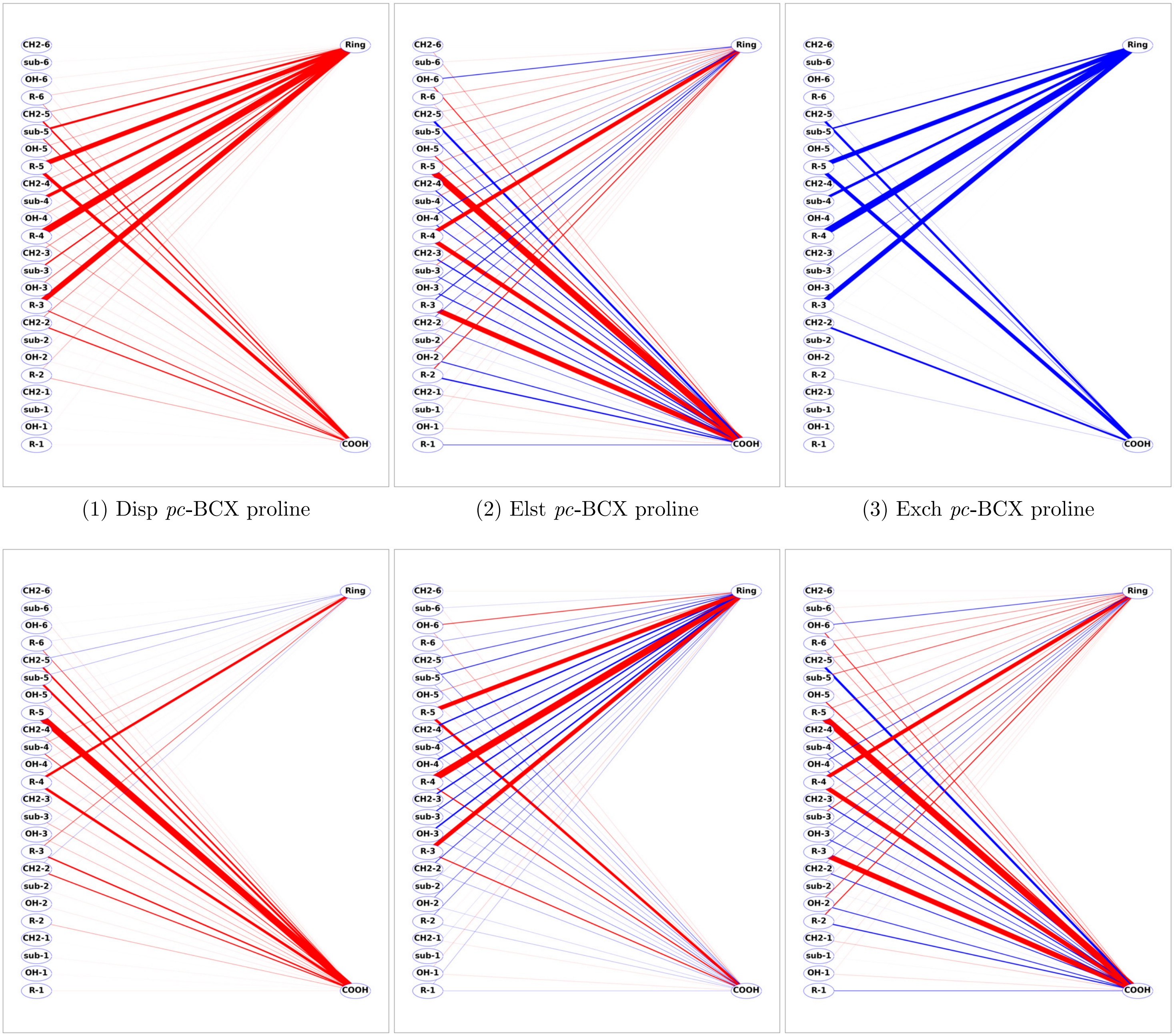

(1) Disp *pc*-BCX proline (2) Elst *pc*-BCX proline (3) Exch *pc*-BCX proline

(4) IndAB *pc*-BCX proline (5) IndBA *pc*-BCX proline (6) Total *pc*-BCX proline

Figure S84: The F-SAPT partitioning for *pc*-BCX proline

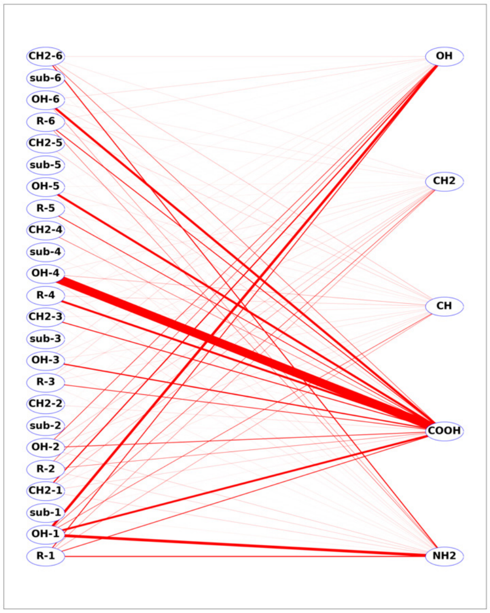

(1) Disp *pc*-BCX serine

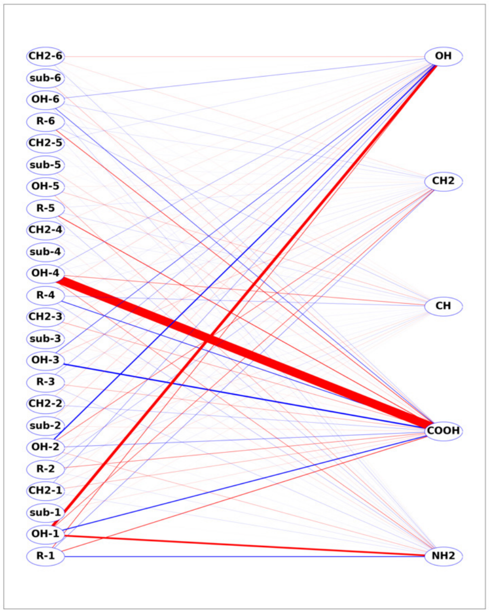

(2) Elst *pc*-BCX serine

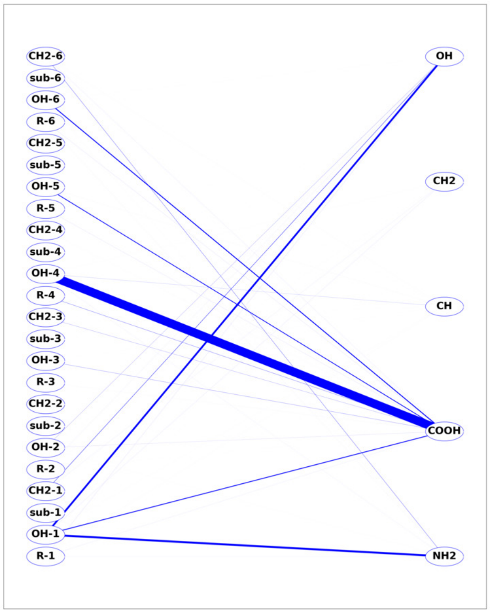

(3) Exch *pc*-BCX serine

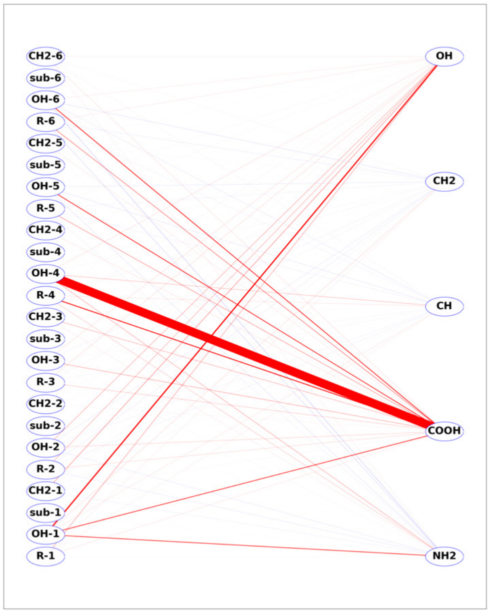

(4) IndAB *pc*-BCX serine

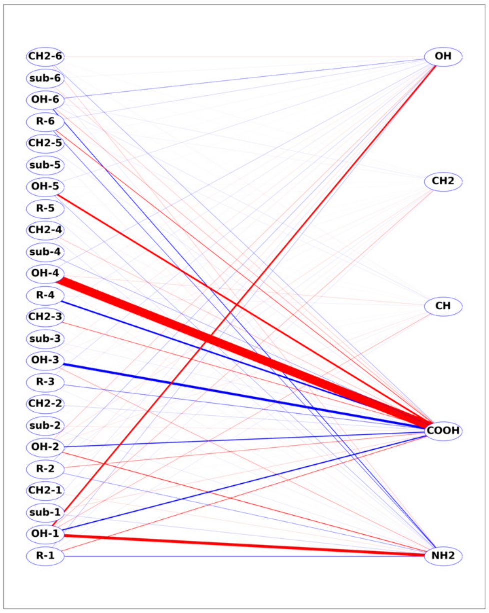

(5) IndBA *pc*-BCX serine

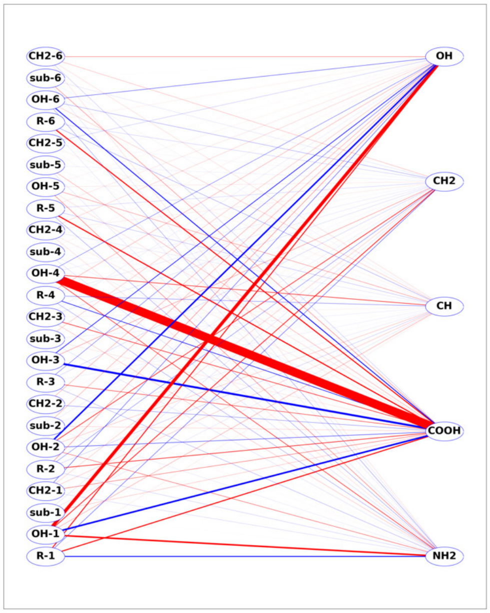

(6) Total *pc*-BCX serine

Figure S85: The F-SAPT partitioning for *pc*-BCX serine

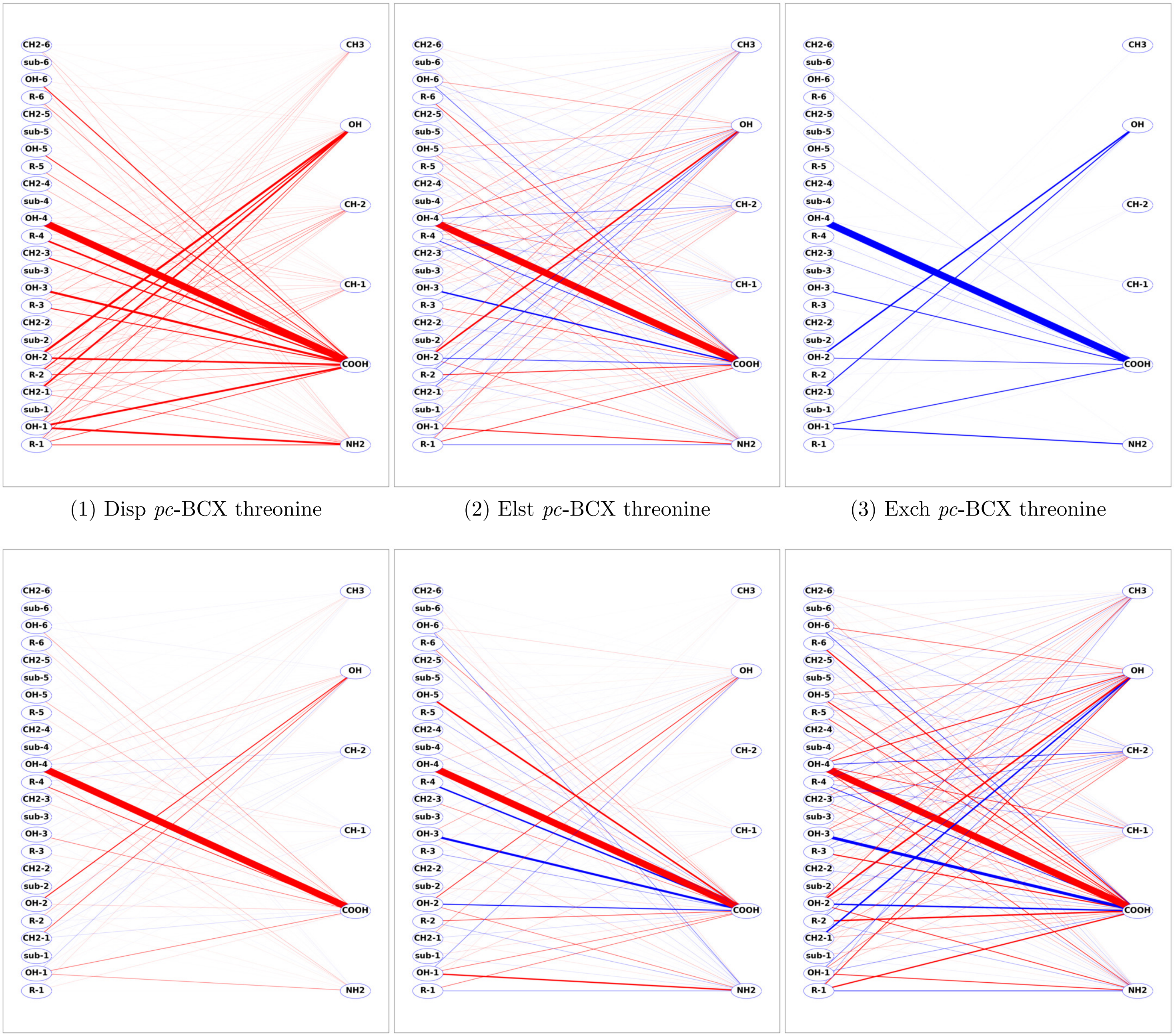

(1) Disp *pc*-BCX threonine

(2) Elst *pc*-BCX threonine

(3) Exch *pc*-BCX threonine

(4) IndAB *pc*-BCX threonine

(5) IndBA *pc*-BCX threonine

(6) Total *pc*-BCX threonine

Figure S86: The F-SAPT partitioning for *pc*-BCX threonine

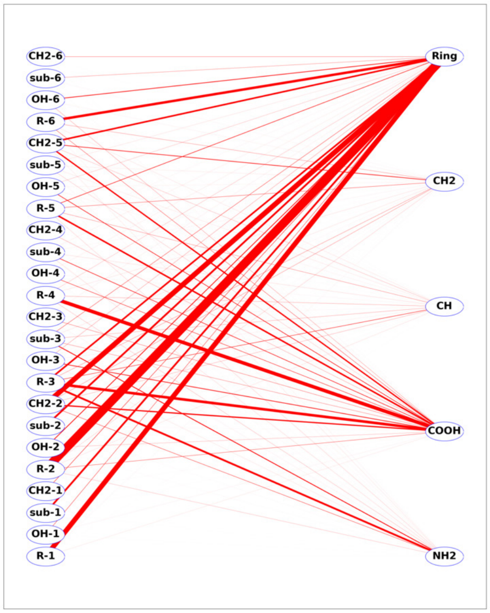

(1) Disp *pc*-BCX tryptophan

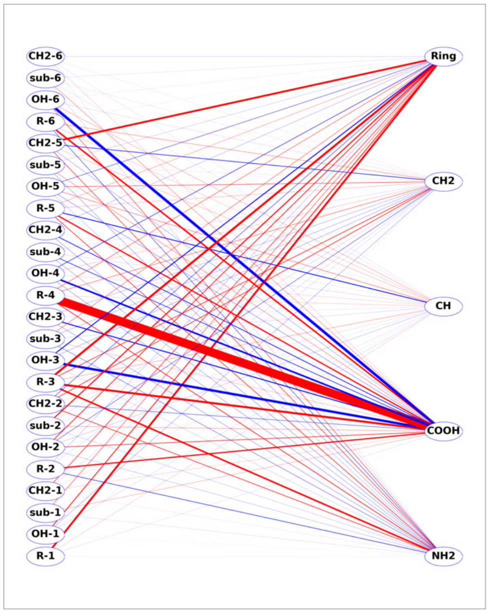

(2) Elst *pc*-BCX tryptophan

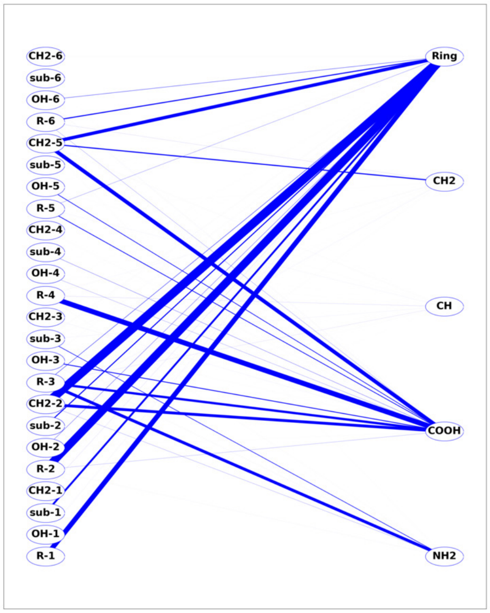

(3) Exch *pc*-BCX tryptophan

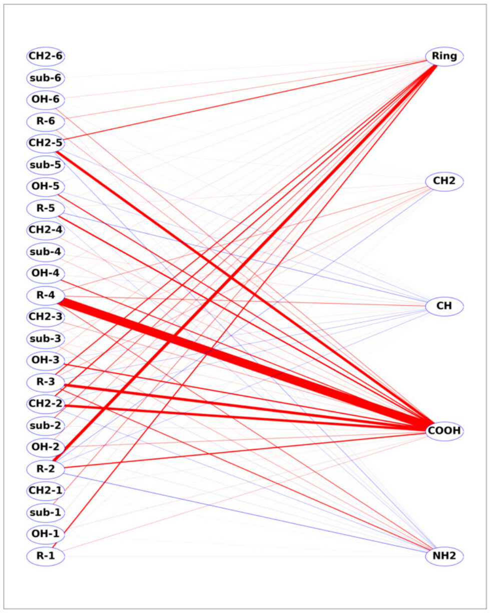

(4) IndAB *pc*-BCX tryptophan

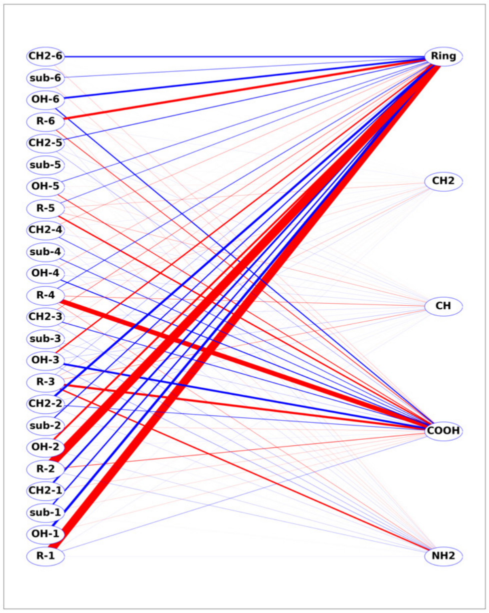

(5) IndBA *pc*-BCX tryptophan

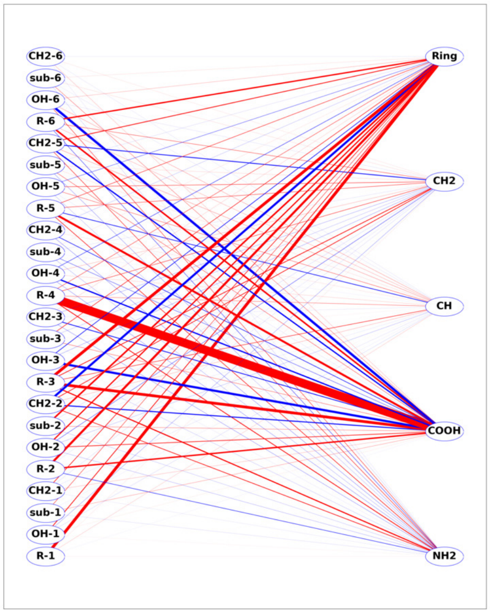

(6) Total *pc*-BCX tryptophan

Figure S87: The F-SAPT partitioning for *pc*-BCX tryptophan

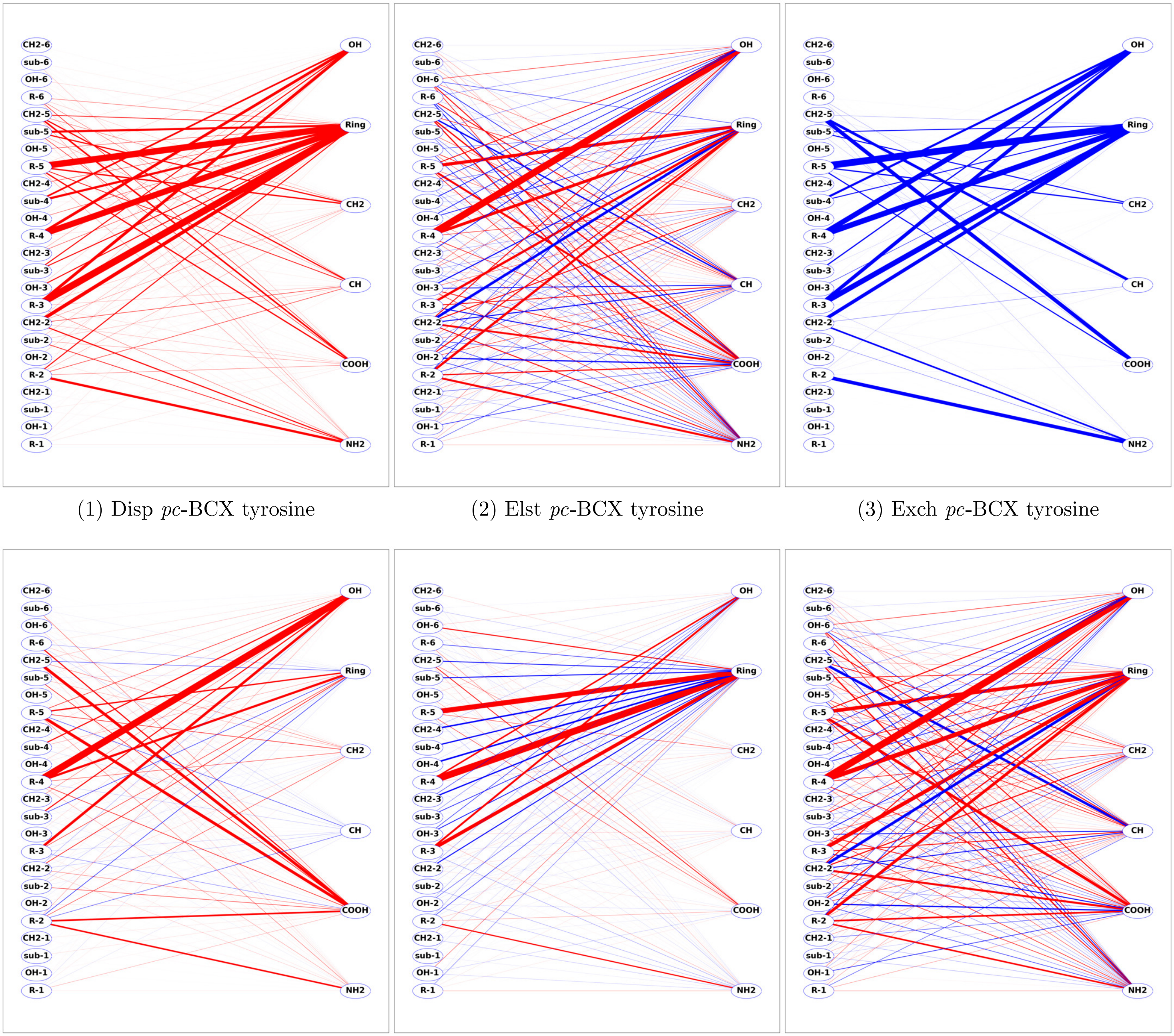

(1) Disp *pc*-BCX tyrosine (2) Elst *pc*-BCX tyrosine (3) Exch *pc*-BCX tyrosine

(4) IndAB *pc*-BCX tyrosine (5) IndBA *pc*-BCX tyrosine (6) Total *pc*-BCX tyrosine

Figure S88: The F-SAPT partitioning for *pc*-BCX tyrosine

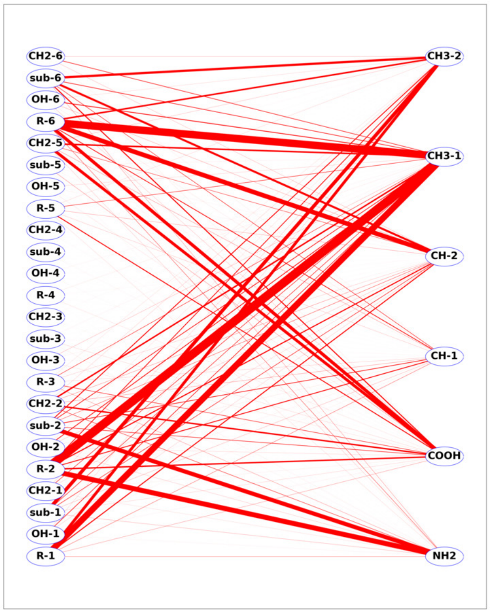

(1) Disp *pc*-BCX valine

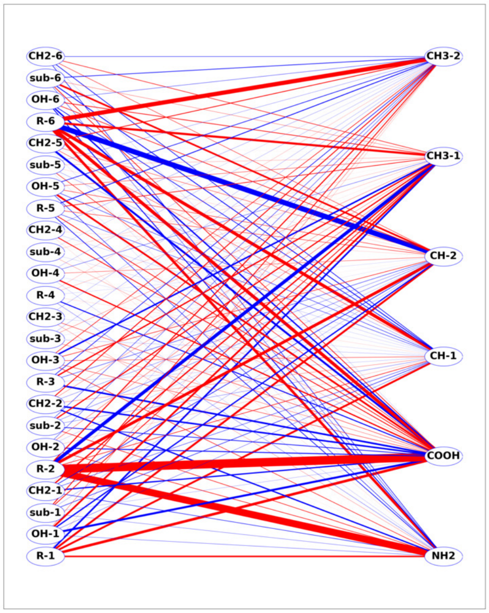

(2) Elst *pc*-BCX valine

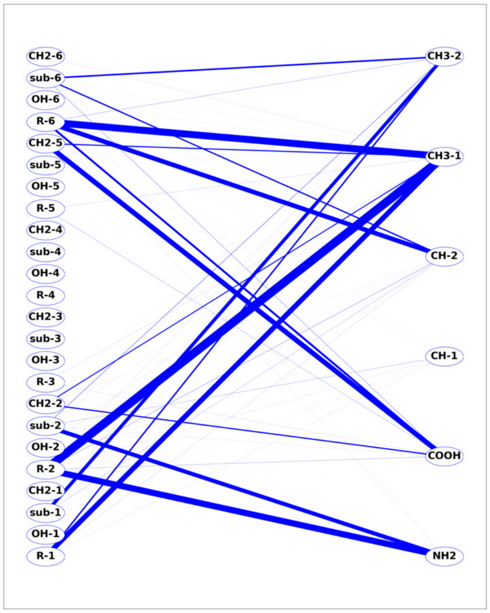

(3) Exch *pc*-BCX valine

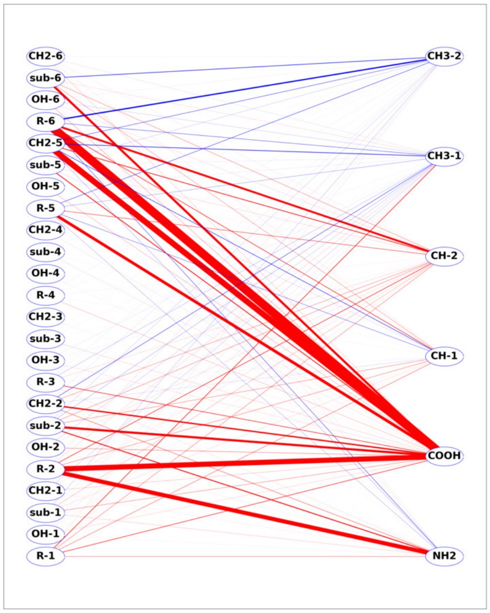

(4) IndAB *pc*-BCX valine

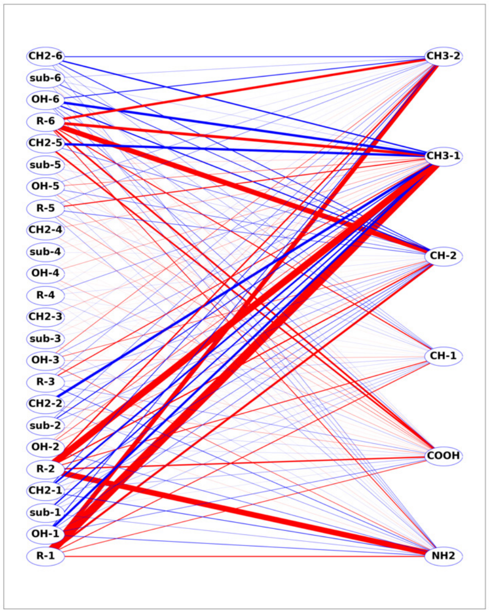

(5) IndBA *pc*-BCX valine

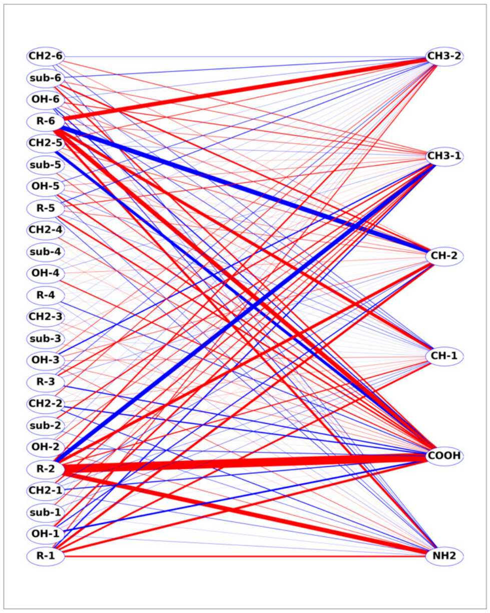

(6) Total *pc*-BCX valine

Figure S89: The F-SAPT partitioning for *pc*-BCX valine

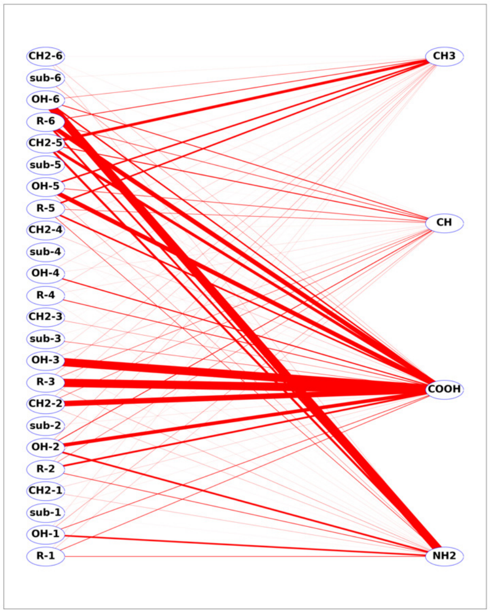

(1) Disp *wc*-BCX alanine

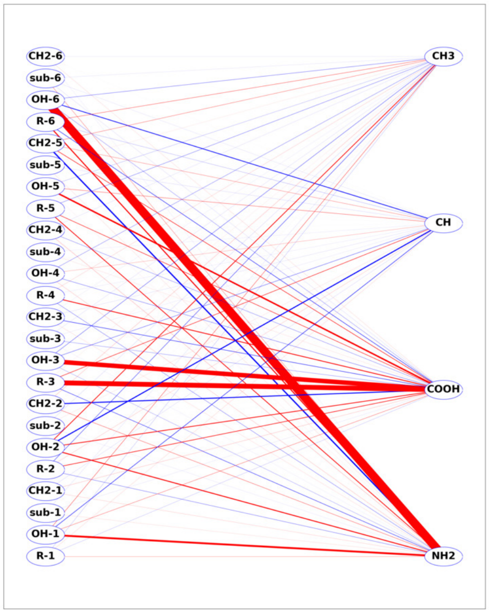

(2) Elst *wc*-BCX alanine

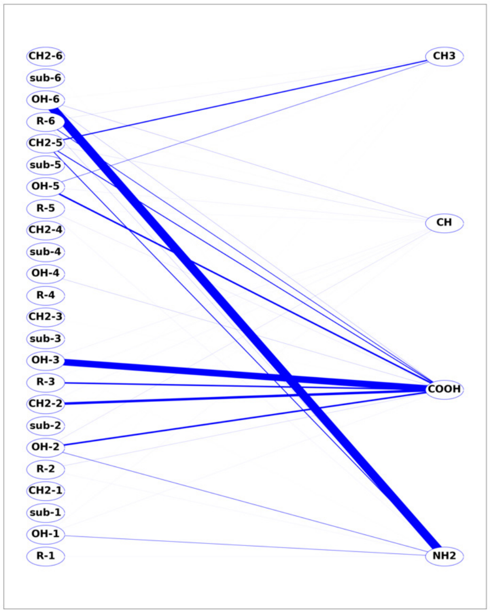

(3) Exch *wc*-BCX alanine

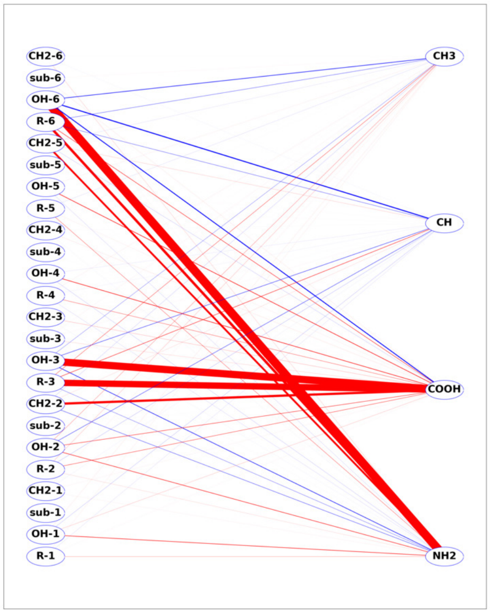

(4) IndAB *wc*-BCX alanine

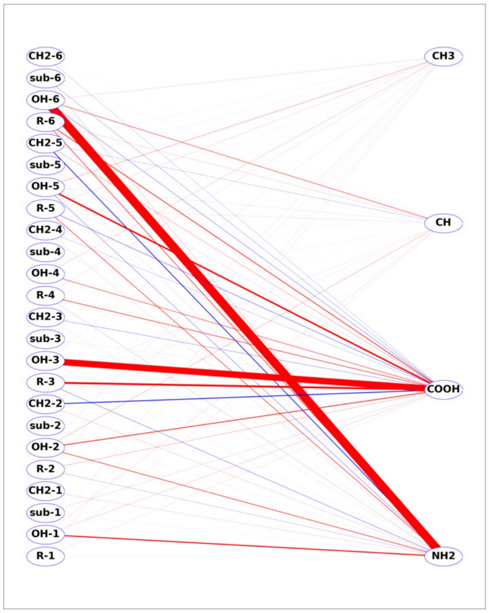

(5) IndBA *wc*-BCX alanine

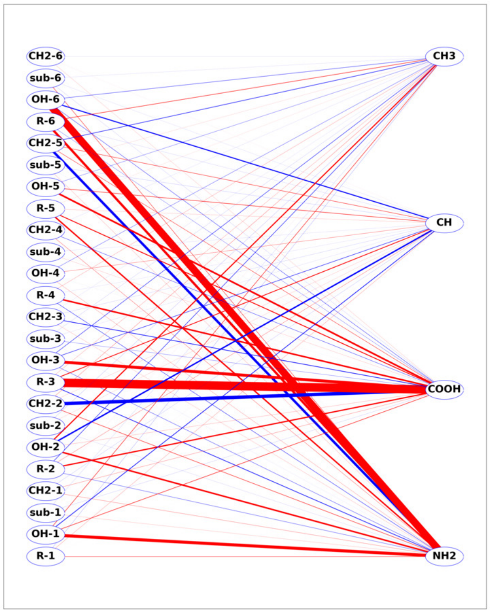

(6) Total *wc*-BCX alanine

Figure S90: The F-SAPT partitioning for *wc*-BCX alanine

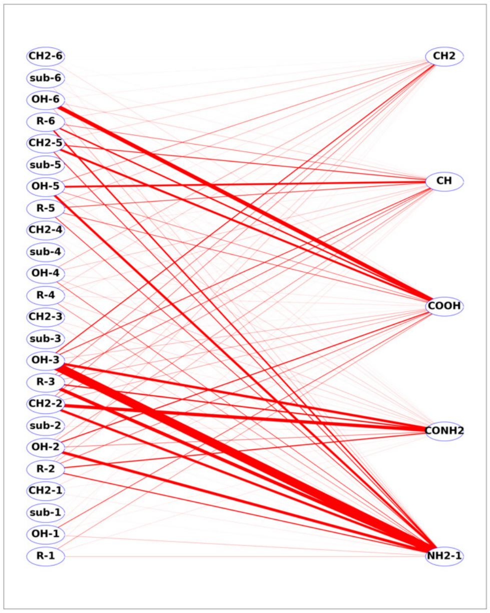

(1) Disp *wc*-BCX asparagine

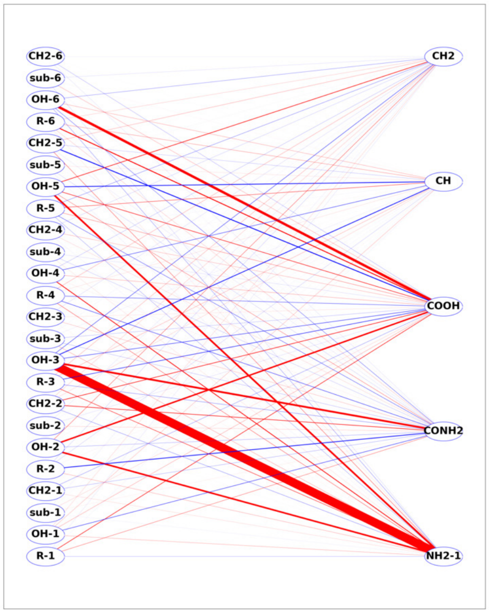

(2) Elst *wc*-BCX asparagine

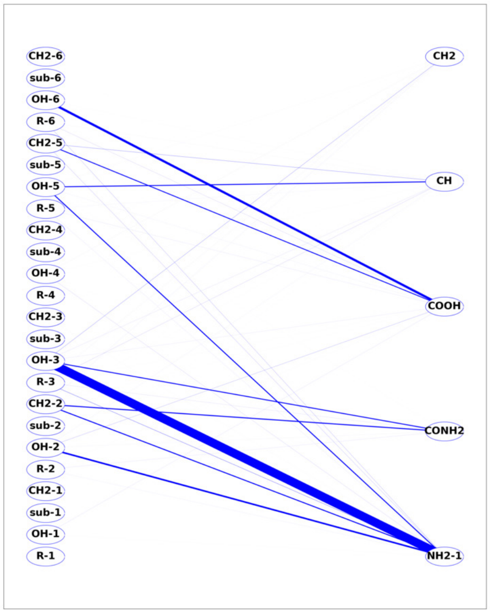

(3) Exch *wc*-BCX asparagine

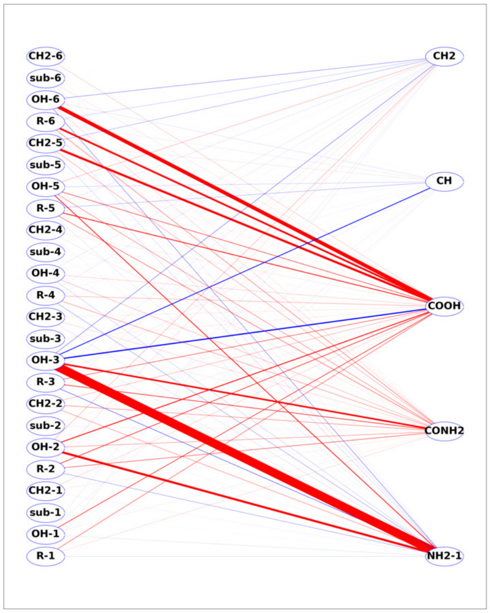

(4) IndAB *wc*-BCX asparagine

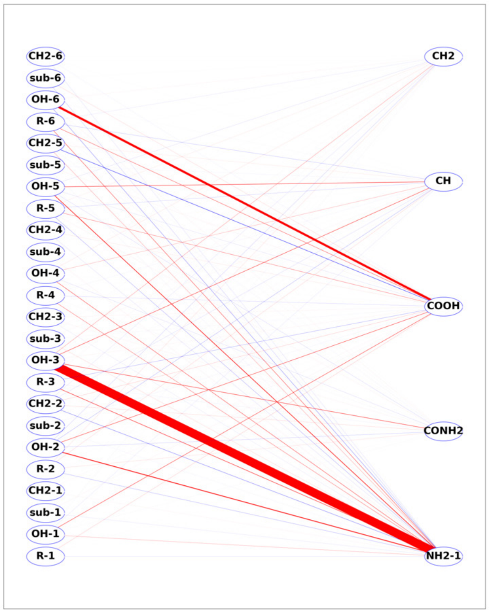

(5) IndBA *wc*-BCX asparagine

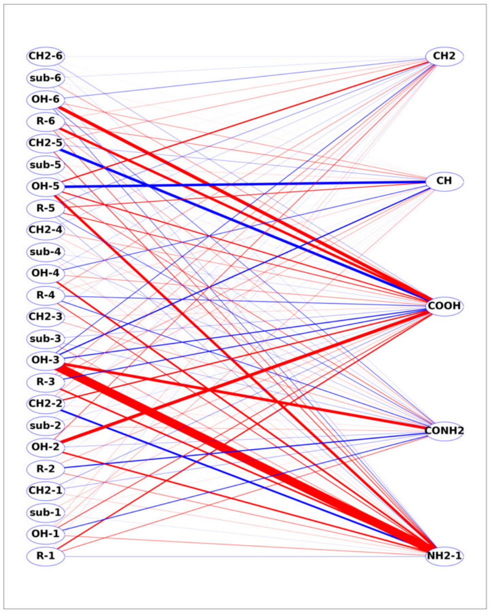

(6) Total *wc*-BCX asparagine

Figure S91: The F-SAPT partitioning for *wc*-BCX asparagine

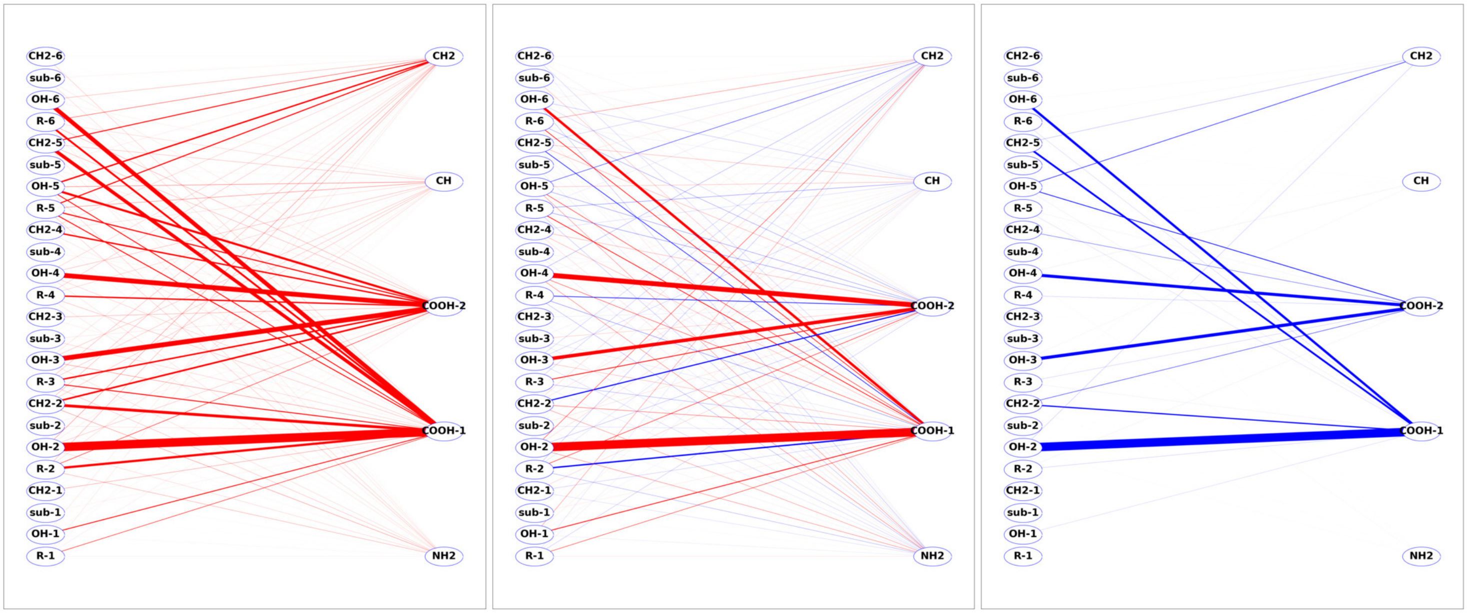

(1) Disp *wc*-BCX aspartate (2) Elst *wc*-BCX aspartate (3) Exch *wc*-BCX aspartate

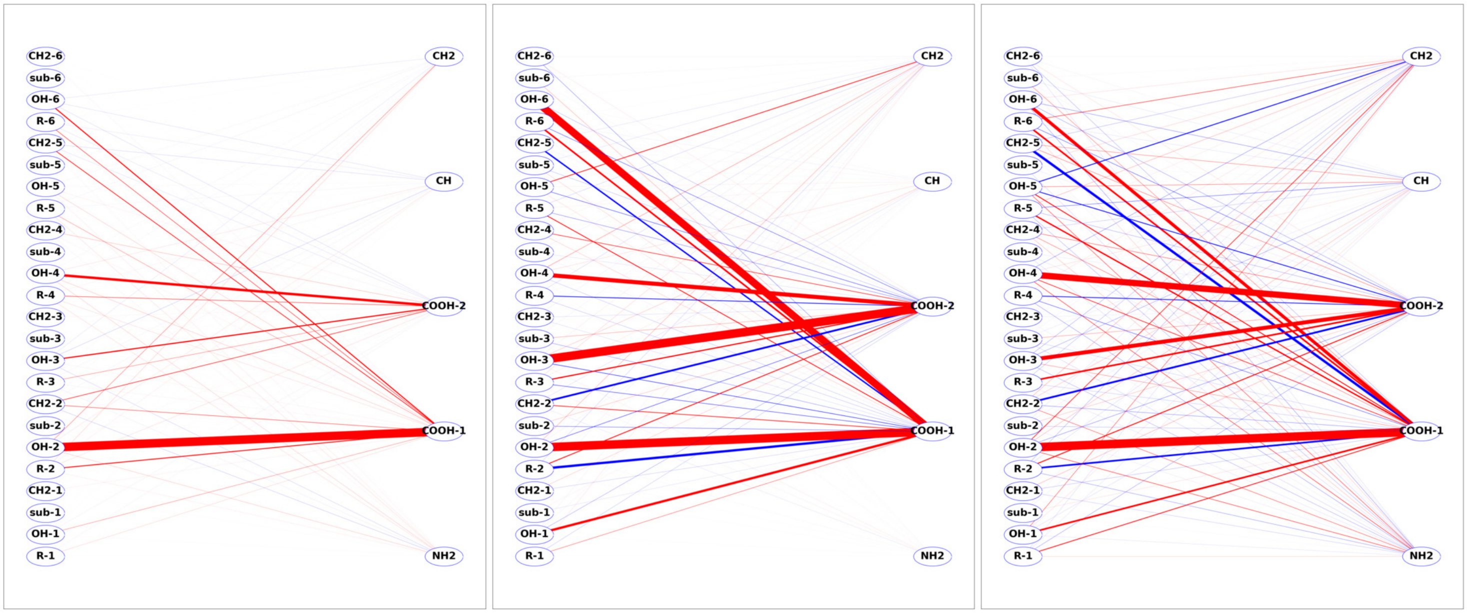

(4) IndAB *wc*-BCX aspartate (5) IndBA *wc*-BCX aspartate (6) Total *wc*-BCX aspartate

Figure S92: The F-SAPT partitioning for *wc*-BCX aspartate

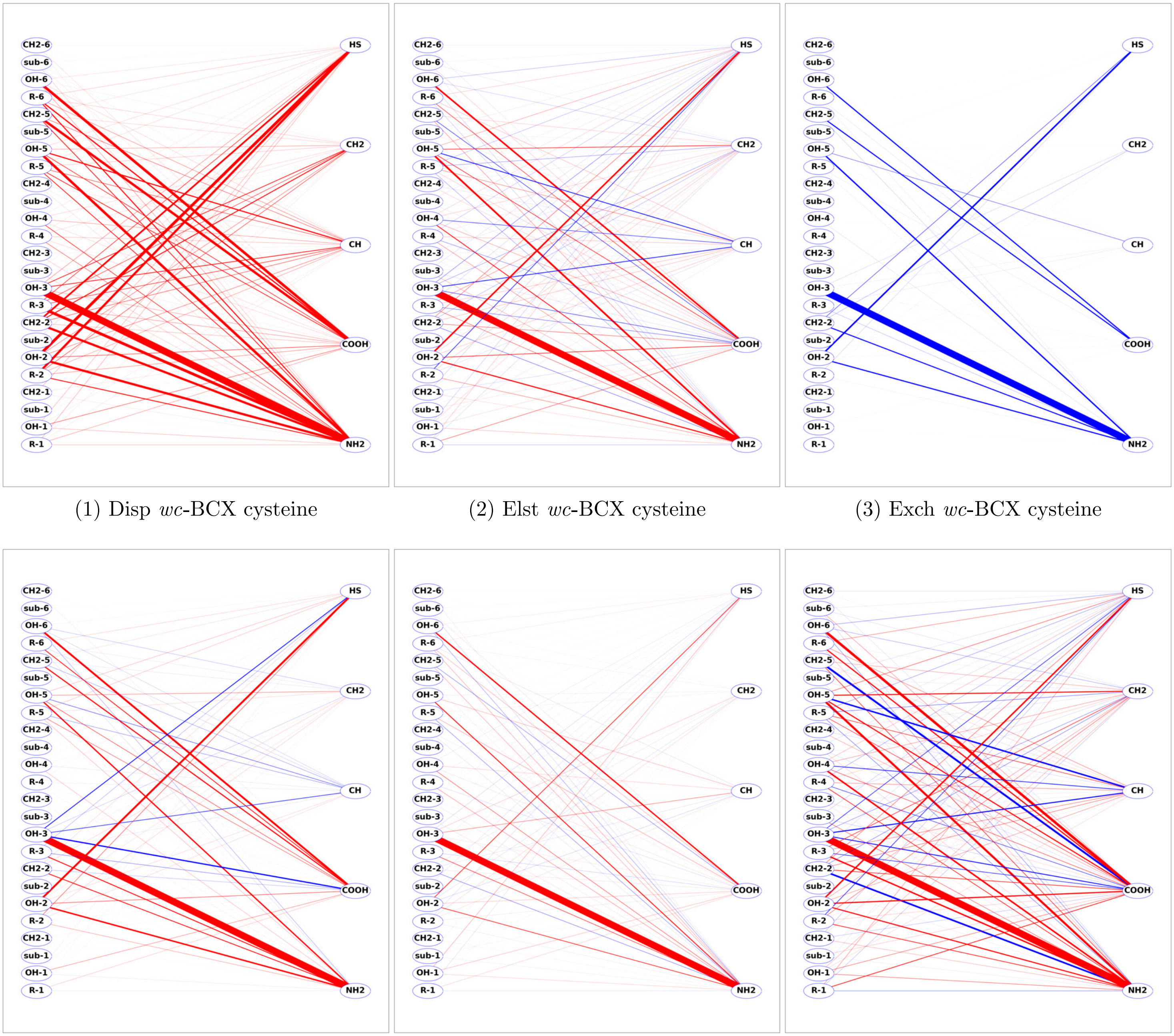

(1) Disp *wc*-BCX cysteine

(2) Elst *wc*-BCX cysteine

(3) Exch *wc*-BCX cysteine

(4) IndAB *wc*-BCX cysteine

(5) IndBA *wc*-BCX cysteine

(6) Total *wc*-BCX cysteine

Figure S93: The F-SAPT partitioning for *wc*-BCX cysteine

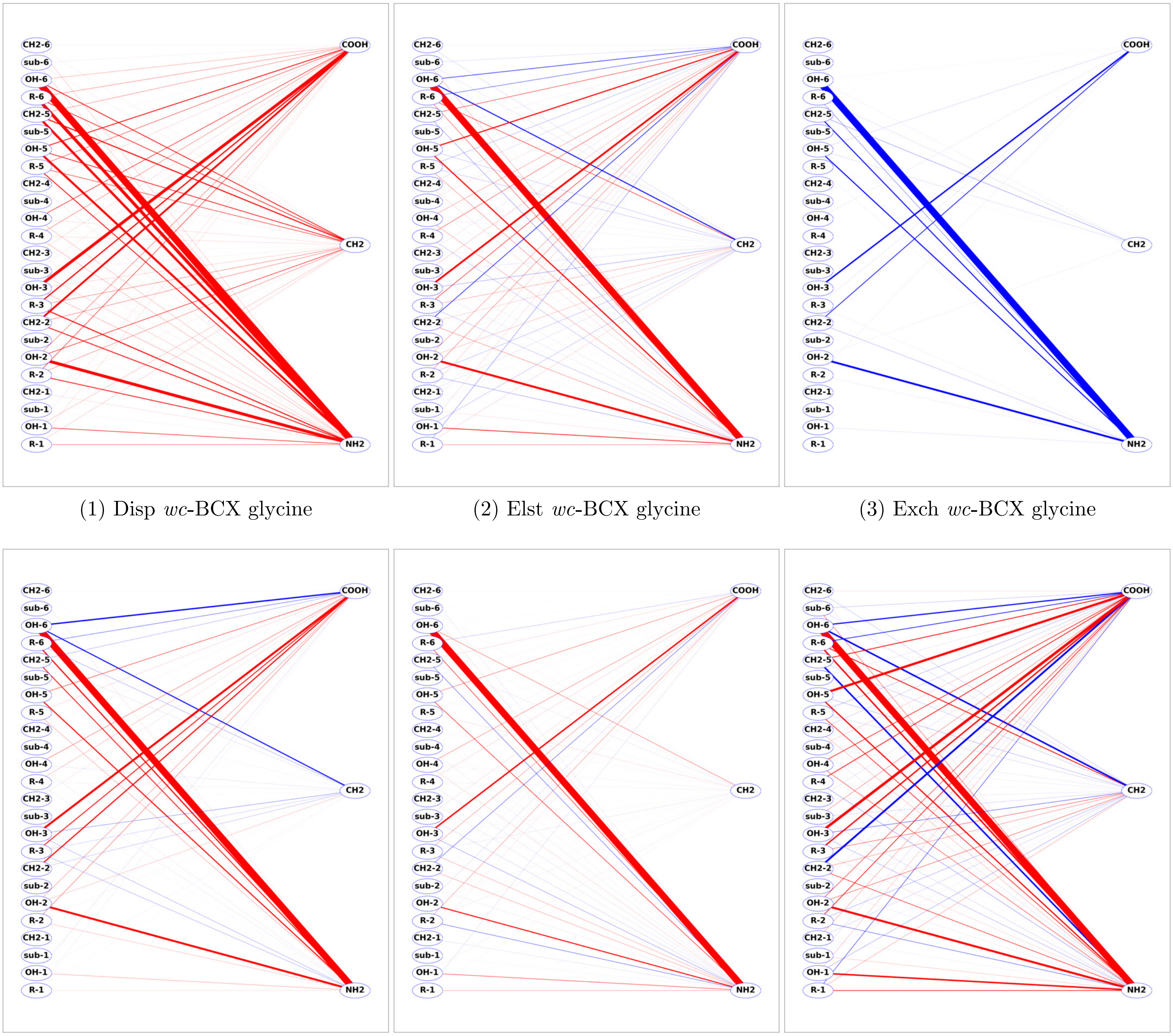

(1) Disp *wc*-BCX glycine (2) Elst *wc*-BCX glycine (3) Exch *wc*-BCX glycine

(4) IndAB *wc*-BCX glycine (5) IndBA *wc*-BCX glycine (6) Total *wc*-BCX glycine

Figure S94: The F-SAPT partitioning for *wc*-BCX glycine

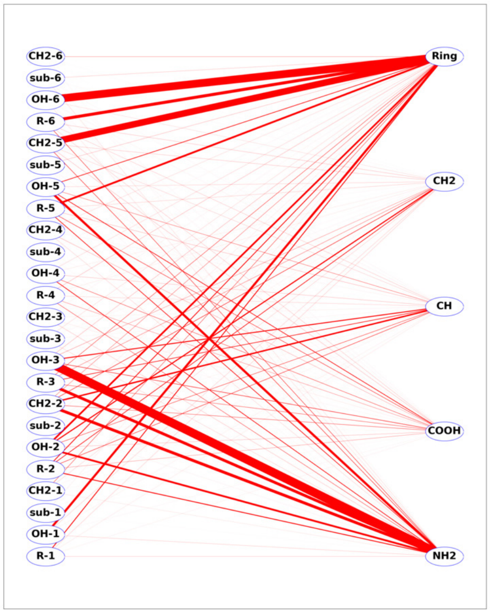

(1) Disp *wc*-BCX histidineD

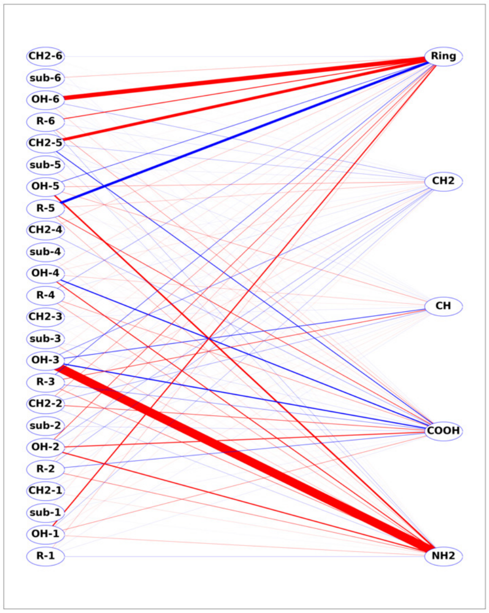

(2) Elst *wc*-BCX histidineD

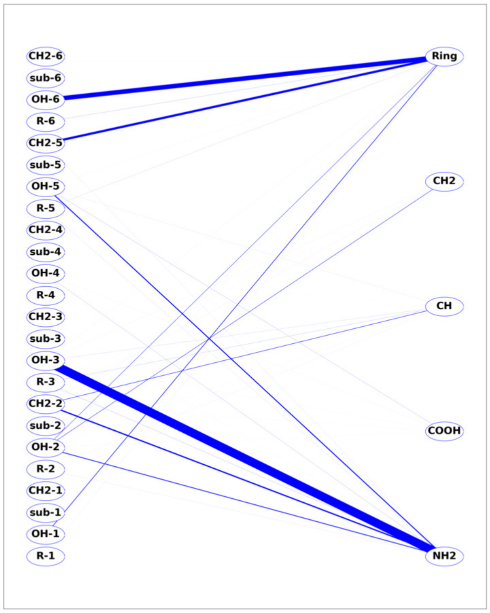

(3) Exch *wc*-BCX histidineD

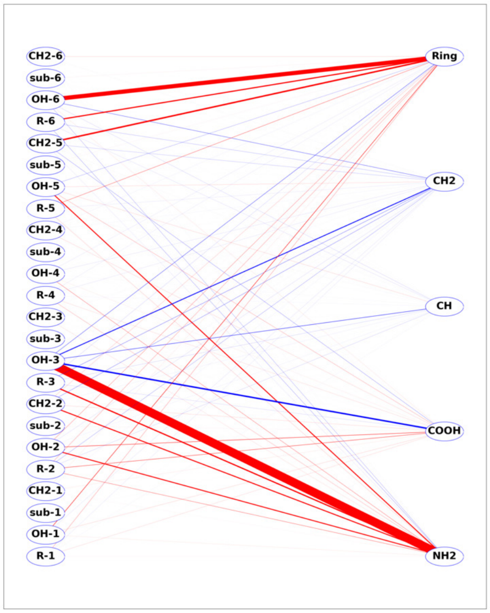

(4) IndAB *wc*-BCX histidineD

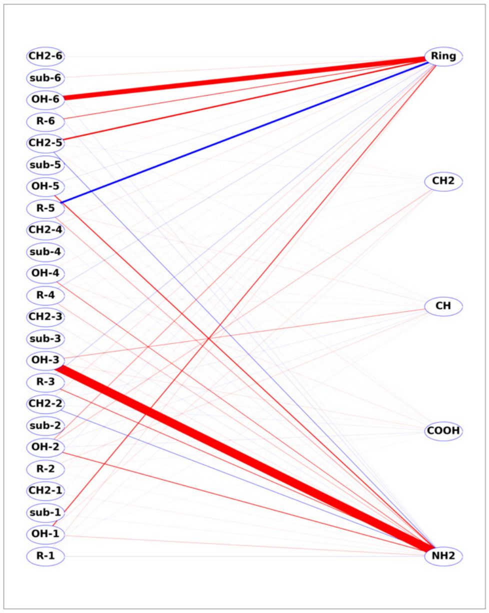

(5) IndBA *wc*-BCX histidineD

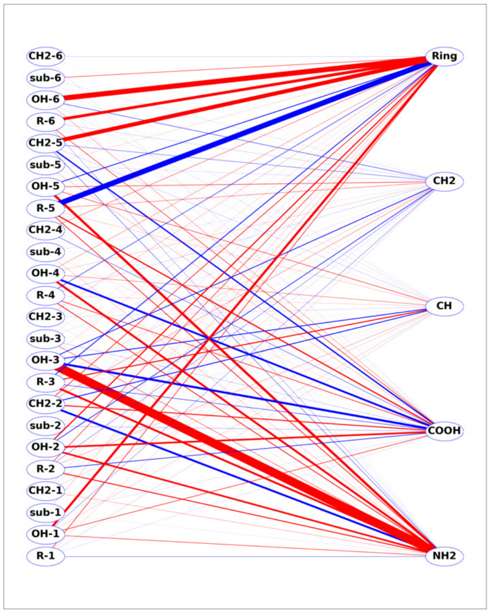

(6) Total *wc*-BCX histidineD

Figure S95: The F-SAPT partitioning for *wc*-BCX histidineD

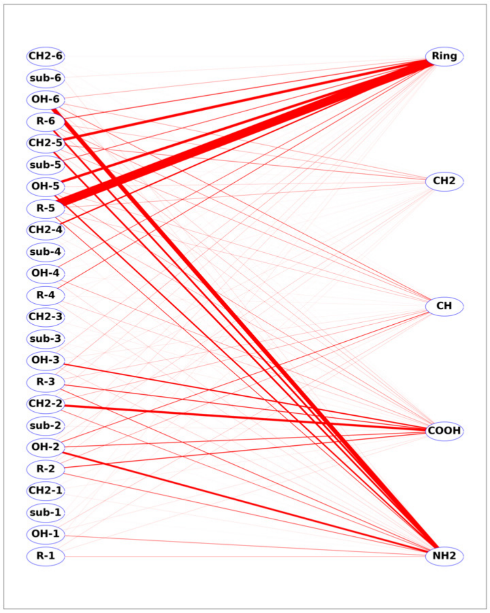

(1) Disp *wc*-BCX histidineE

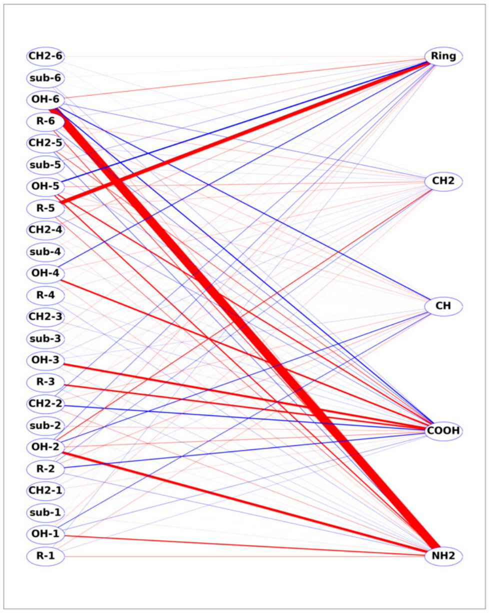

(2) Elst *wc*-BCX histidineE

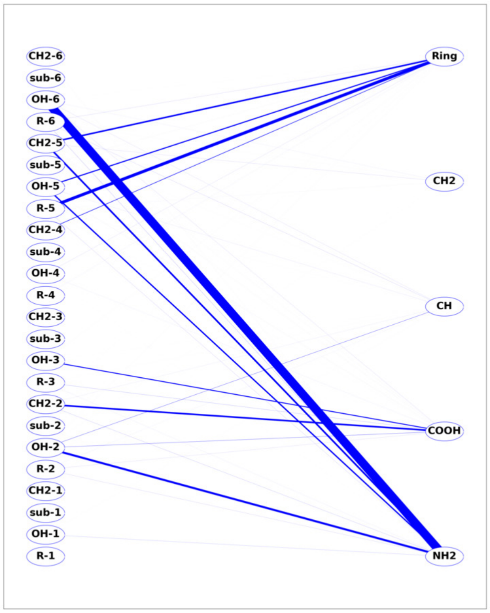

(3) Exch *wc*-BCX histidineE

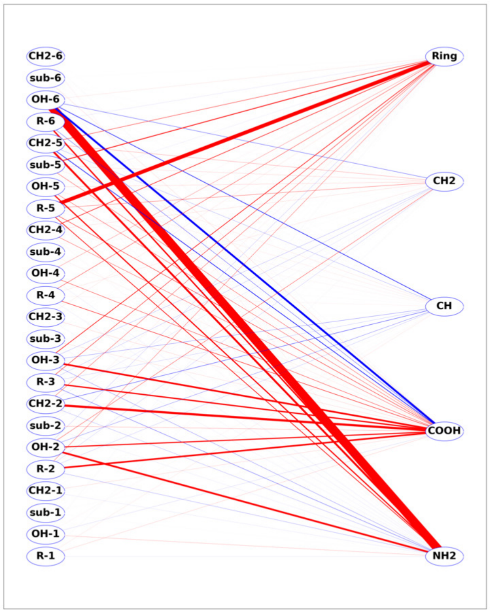

(4) IndAB *wc*-BCX histidineE

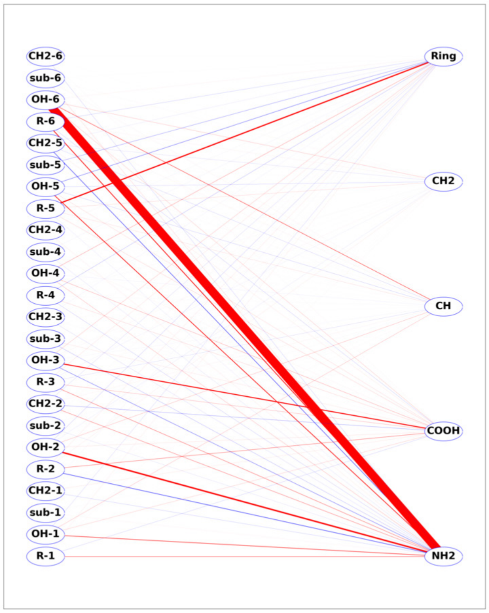

(5) IndBA *wc*-BCX histidineE

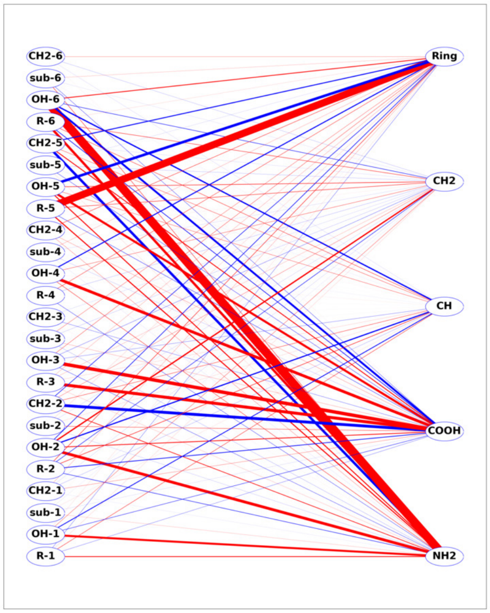

(6) Total *wc*-BCX histidineE

Figure S96: The F-SAPT partitioning for *wc*-BCX histidineE

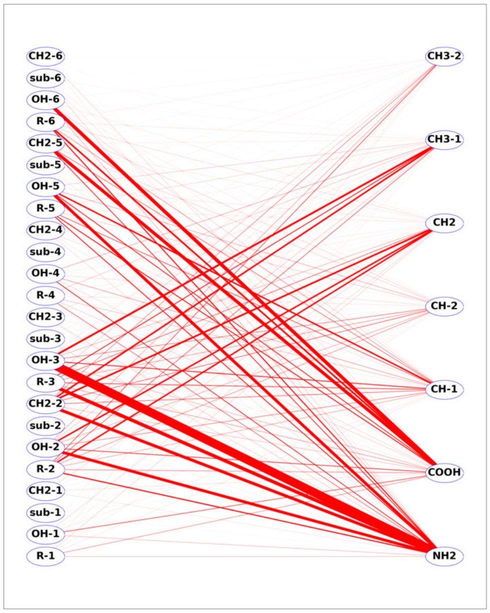

(1) Disp *wc*-BCX isoleucine

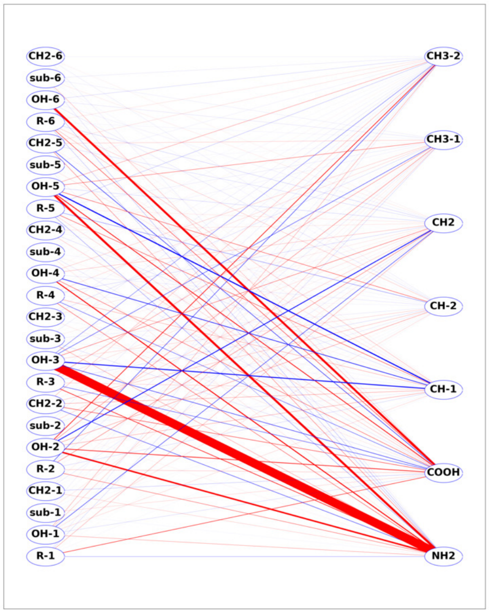

(2) Elst *wc*-BCX isoleucine

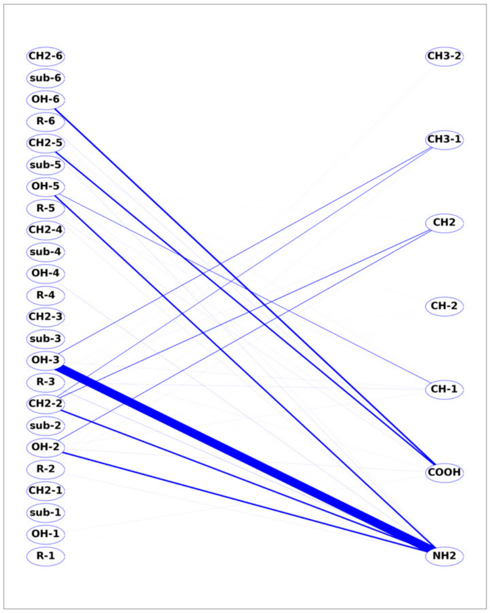

(3) Exch *wc*-BCX isoleucine

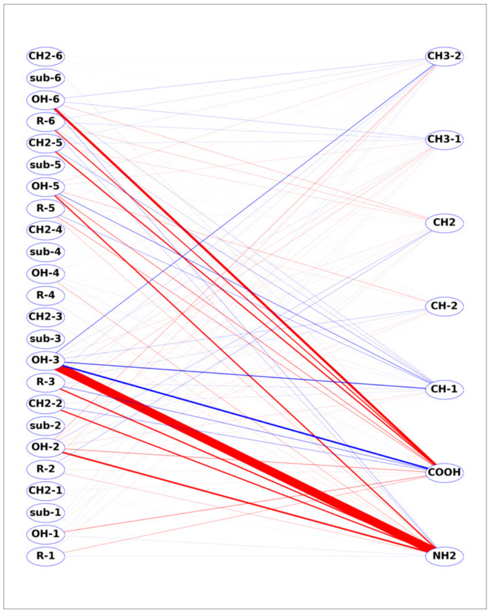

(4) IndAB *wc*-BCX isoleucine

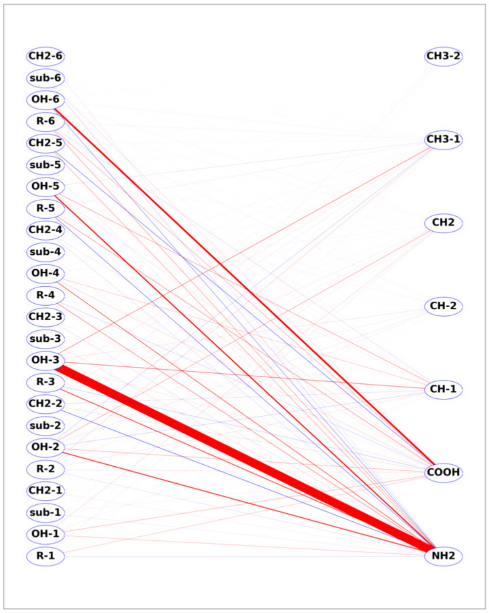

(5) IndBA *wc*-BCX isoleucine

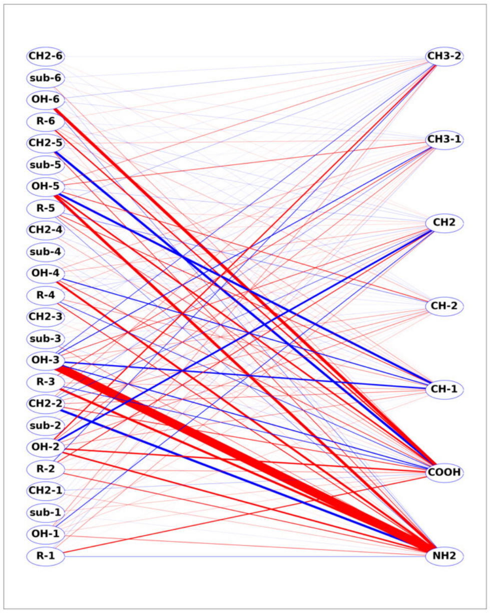

(6) Total *wc*-BCX isoleucine

Figure S97: The F-SAPT partitioning for *wc*-BCX isoleucine

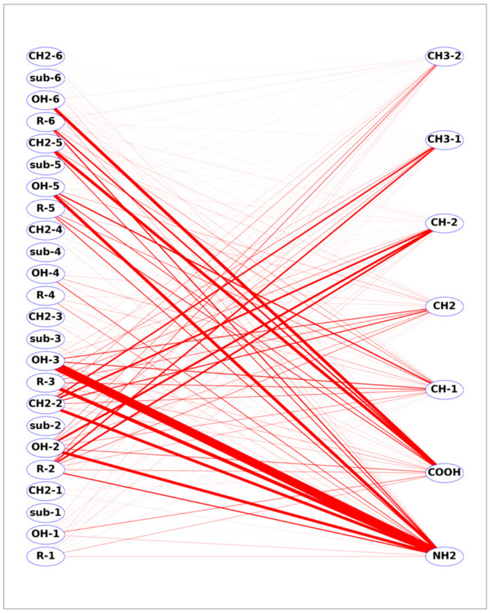

(1) Disp *wc*-BCX leucine

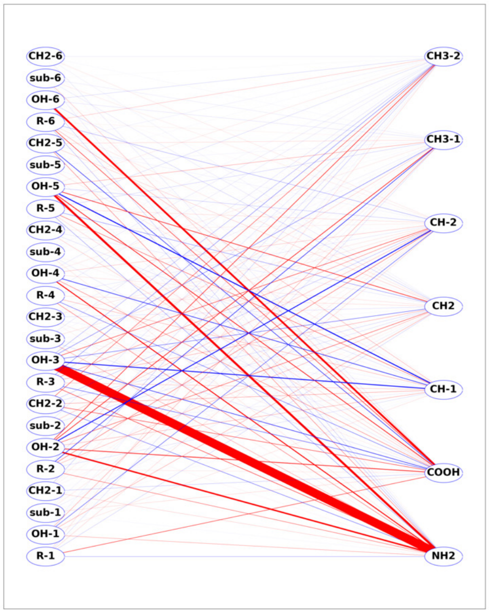

(2) Elst *wc*-BCX leucine

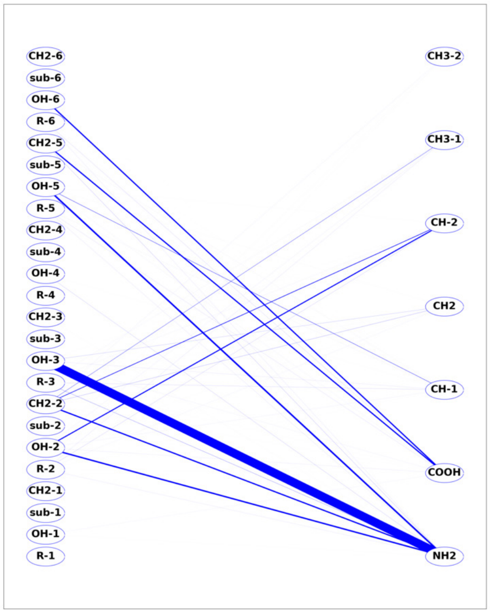

(3) Exch *wc*-BCX leucine

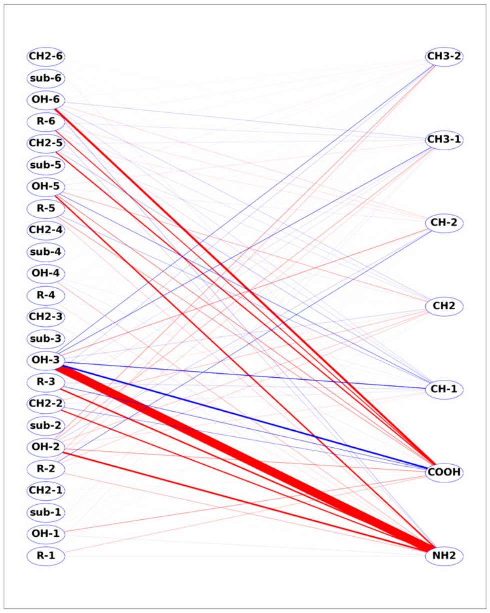

(4) IndAB *wc*-BCX leucine

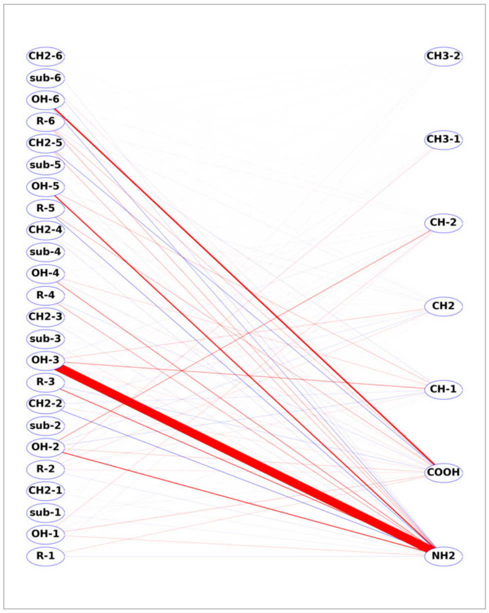

(5) IndBA *wc*-BCX leucine

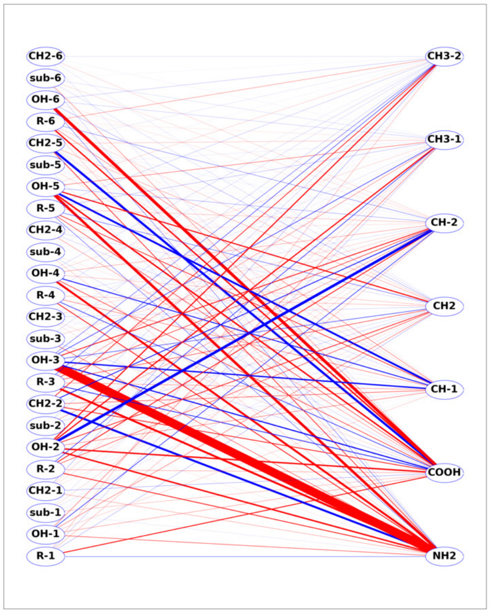

(6) Total *wc*-BCX leucine

Figure S98: The F-SAPT partitioning for *wc*-BCX leucine

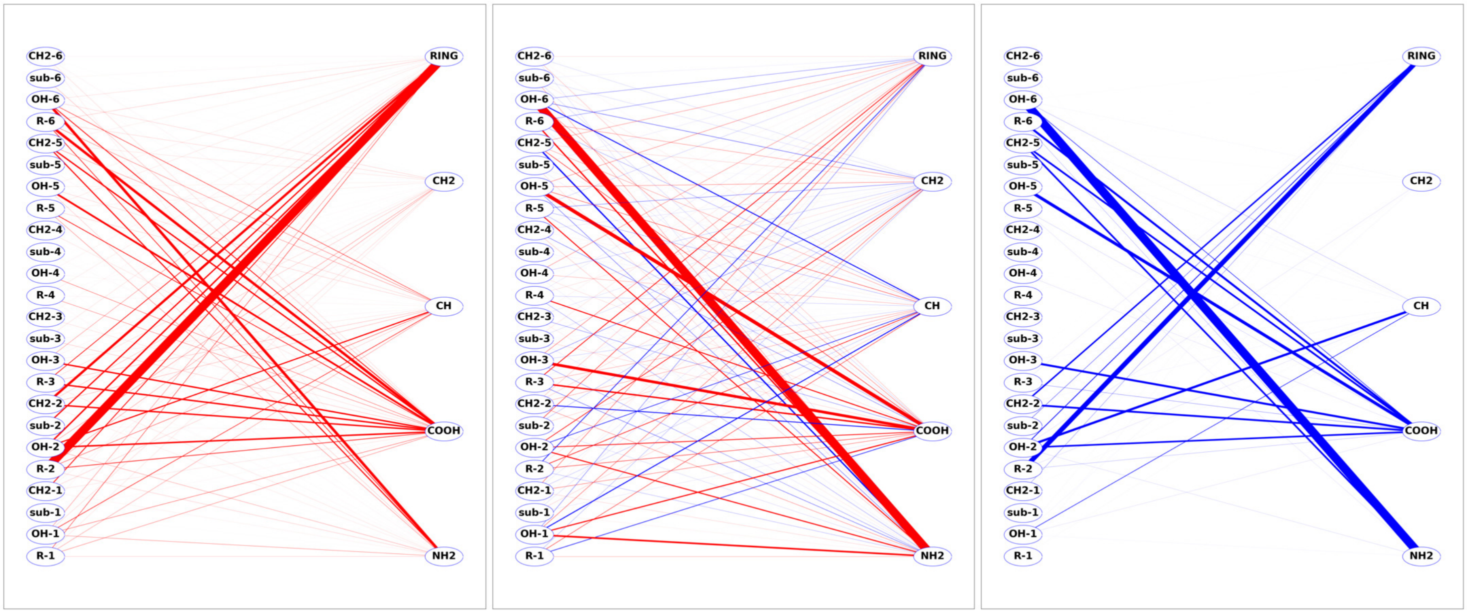

(1) Disp *wc*-BCX phenylalanine (2) Elst *wc*-BCX phenylalanine (3) Exch *wc*-BCX phenylalanine

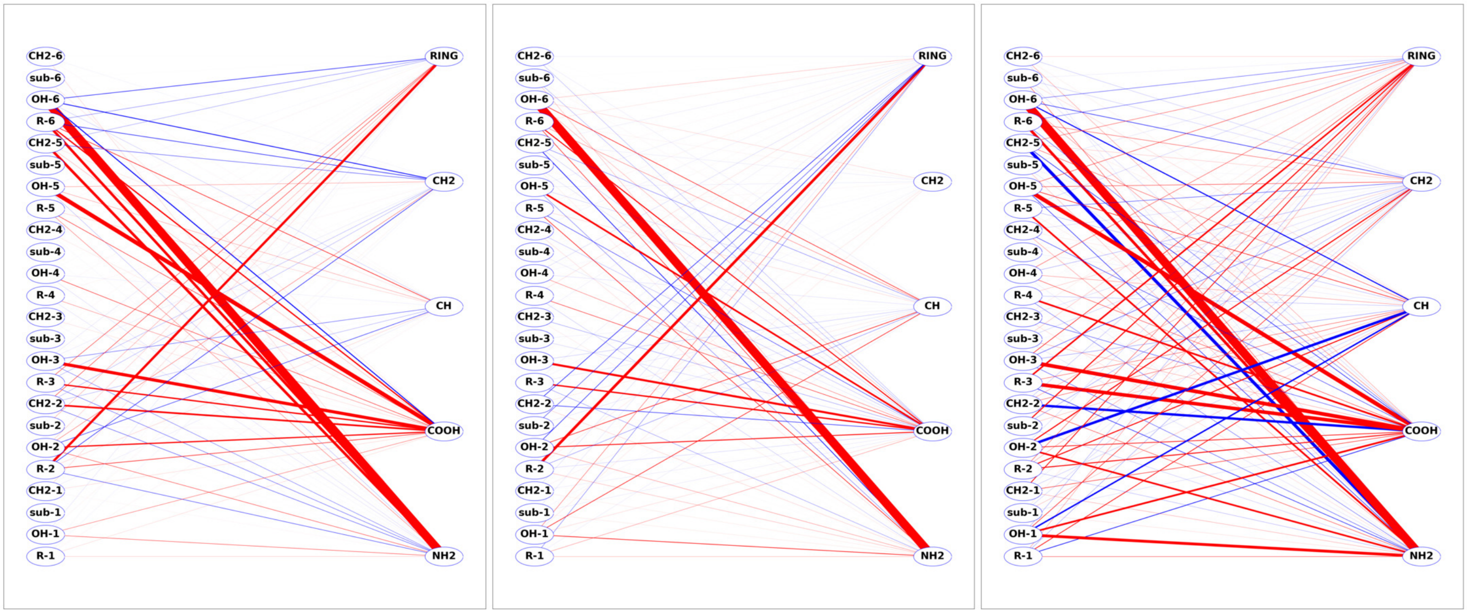

(4) IndAB *wc*-BCX phenylalanine (5) IndBA *wc*-BCX phenylalanine (6) Total *wc*-BCX phenylalanine

Figure S99: The F-SAPT partitioning for *wc*-BCX phenylalanine

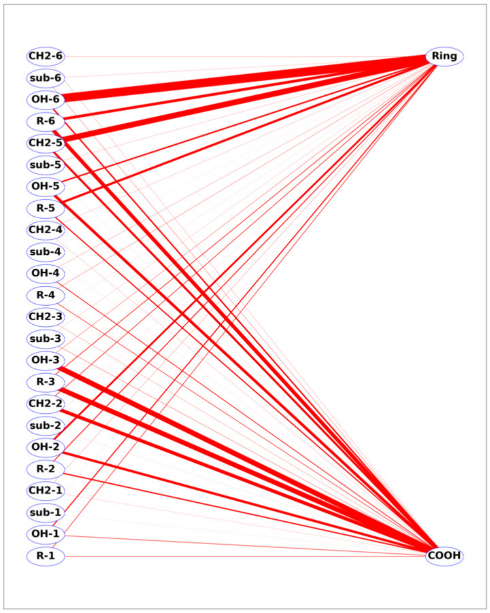

(1) Disp *wc*-BCX proline

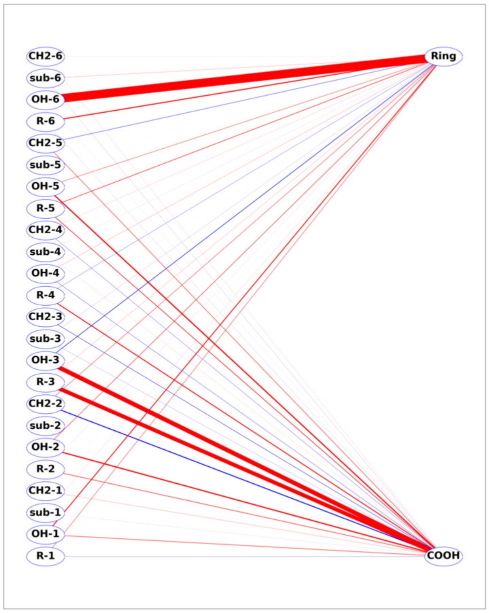

(2) Elst *wc*-BCX proline

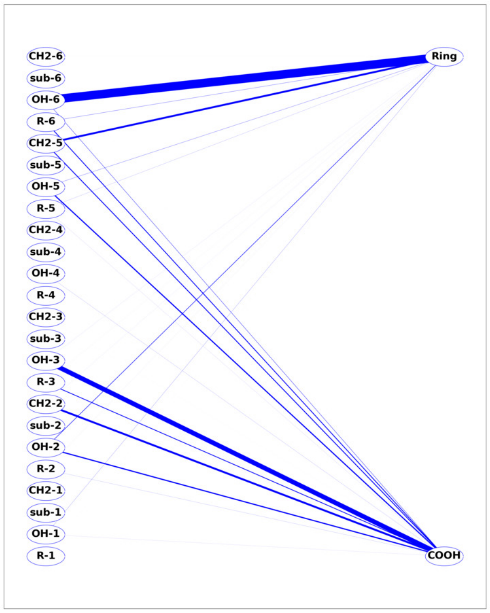

(3) Exch *wc*-BCX proline

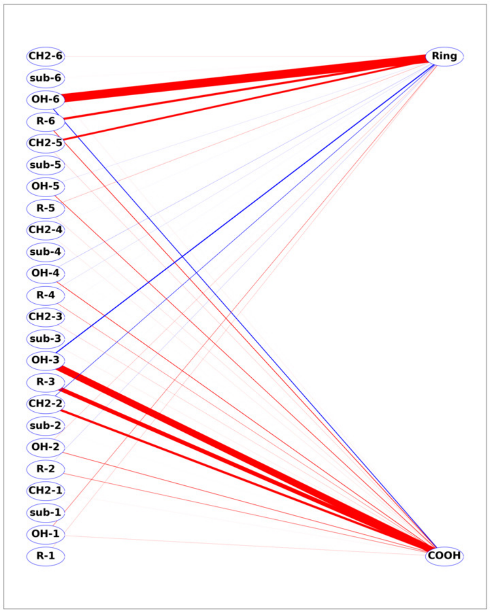

(4) IndAB *wc*-BCX proline

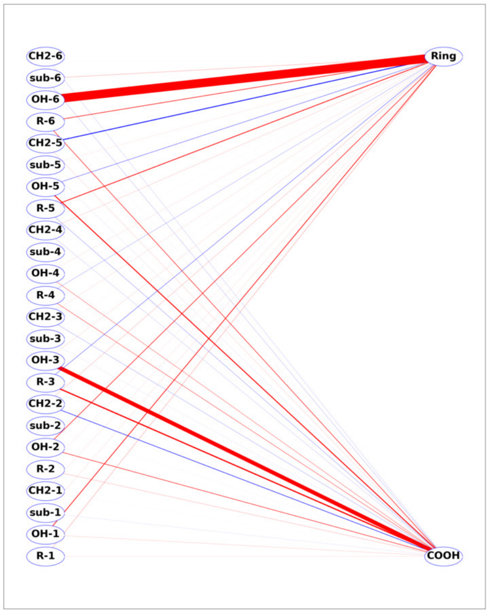

(5) IndBA *wc*-BCX proline

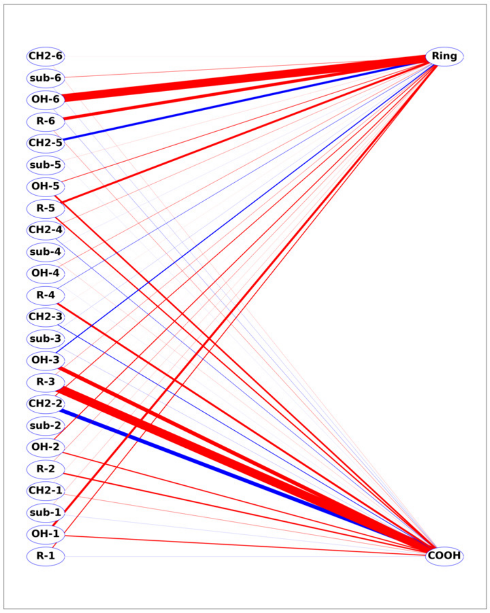

(6) Total *wc*-BCX proline

Figure S100: The F-SAPT partitioning for *wc*-BCX proline

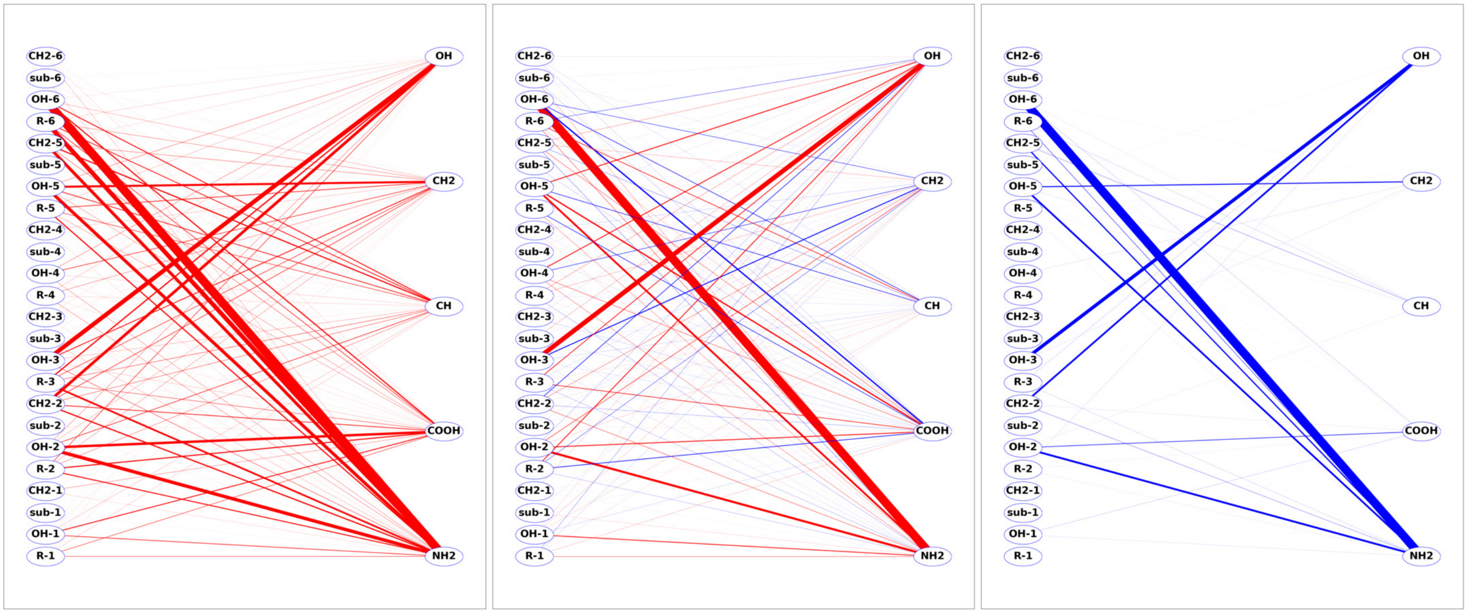

(1) Disp *wc*-BCX serine (2) Elst *wc*-BCX serine (3) Exch *wc*-BCX serine

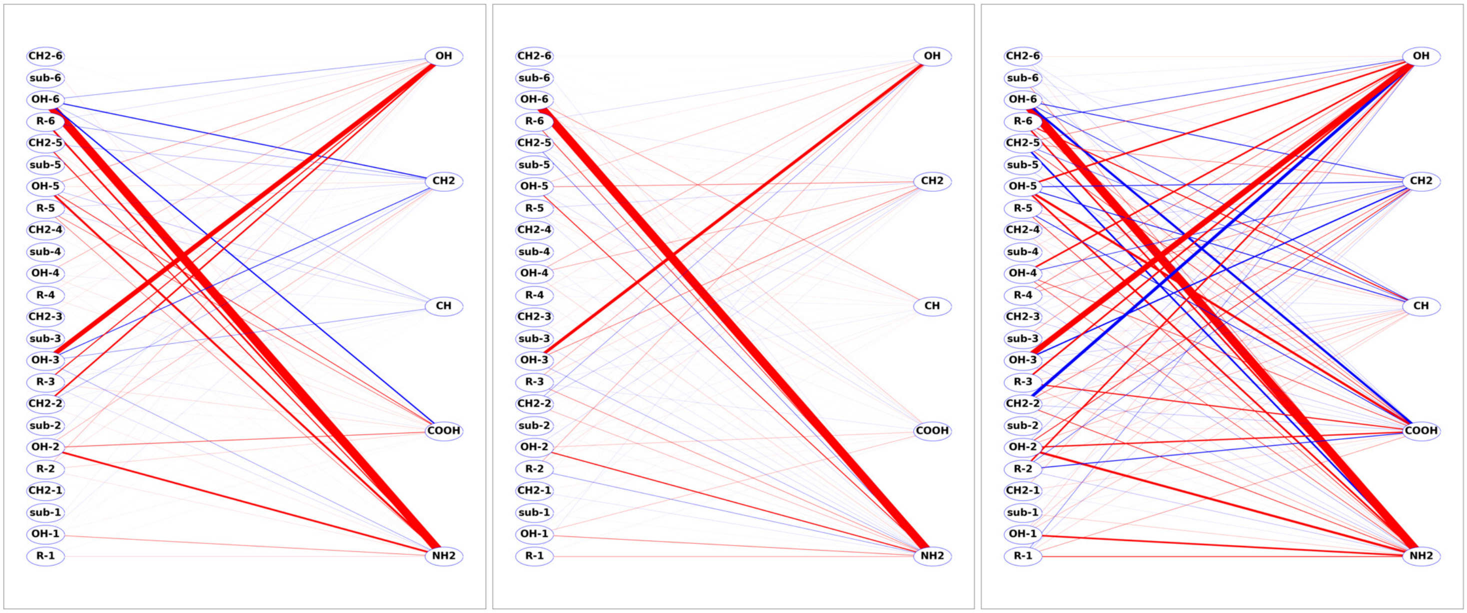

(4) IndAB *wc*-BCX serine (5) IndBA *wc*-BCX serine (6) Total *wc*-BCX serine

Figure S101: The F-SAPT partitioning for *wc*-BCX serine

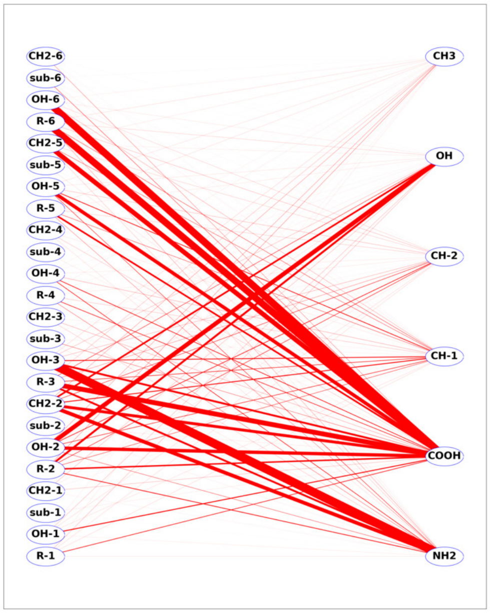

(1) Disp *wc*-BCX threonine

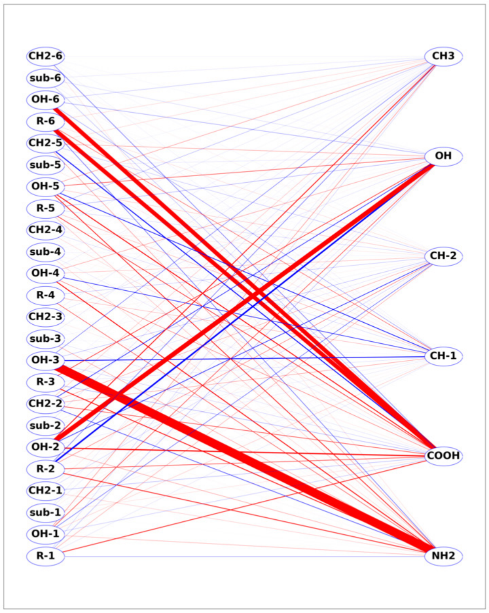

(2) Elst *wc*-BCX threonine

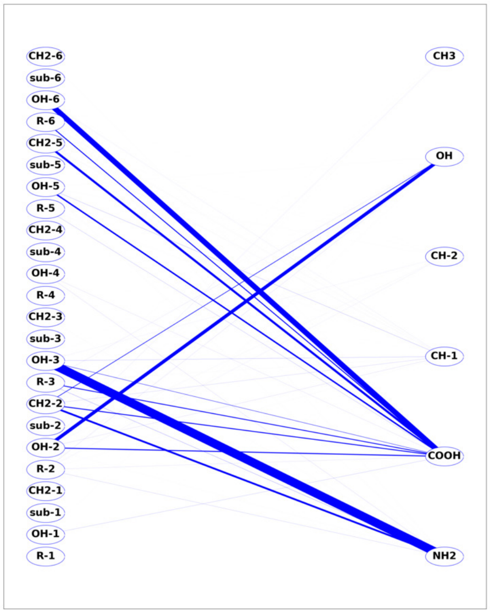

(3) Exch *wc*-BCX threonine

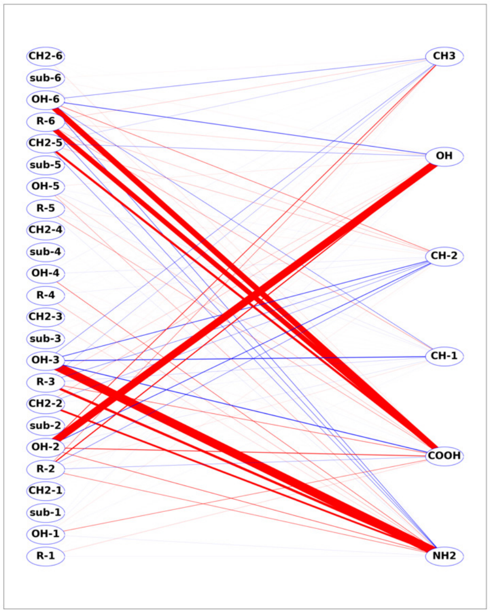

(4) IndAB *wc*-BCX threonine

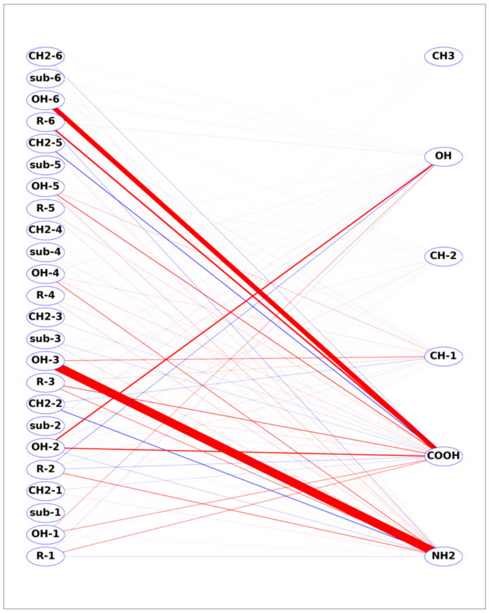

(5) IndBA *wc*-BCX threonine

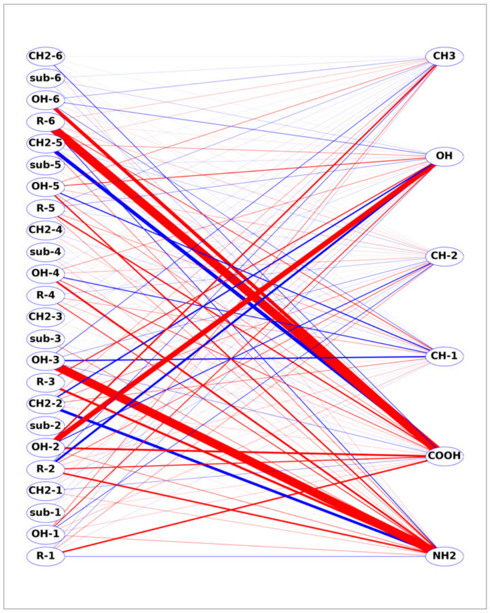

(6) Total *wc*-BCX threonine

Figure S102: The F-SAPT partitioning for *wc*-BCX threonine

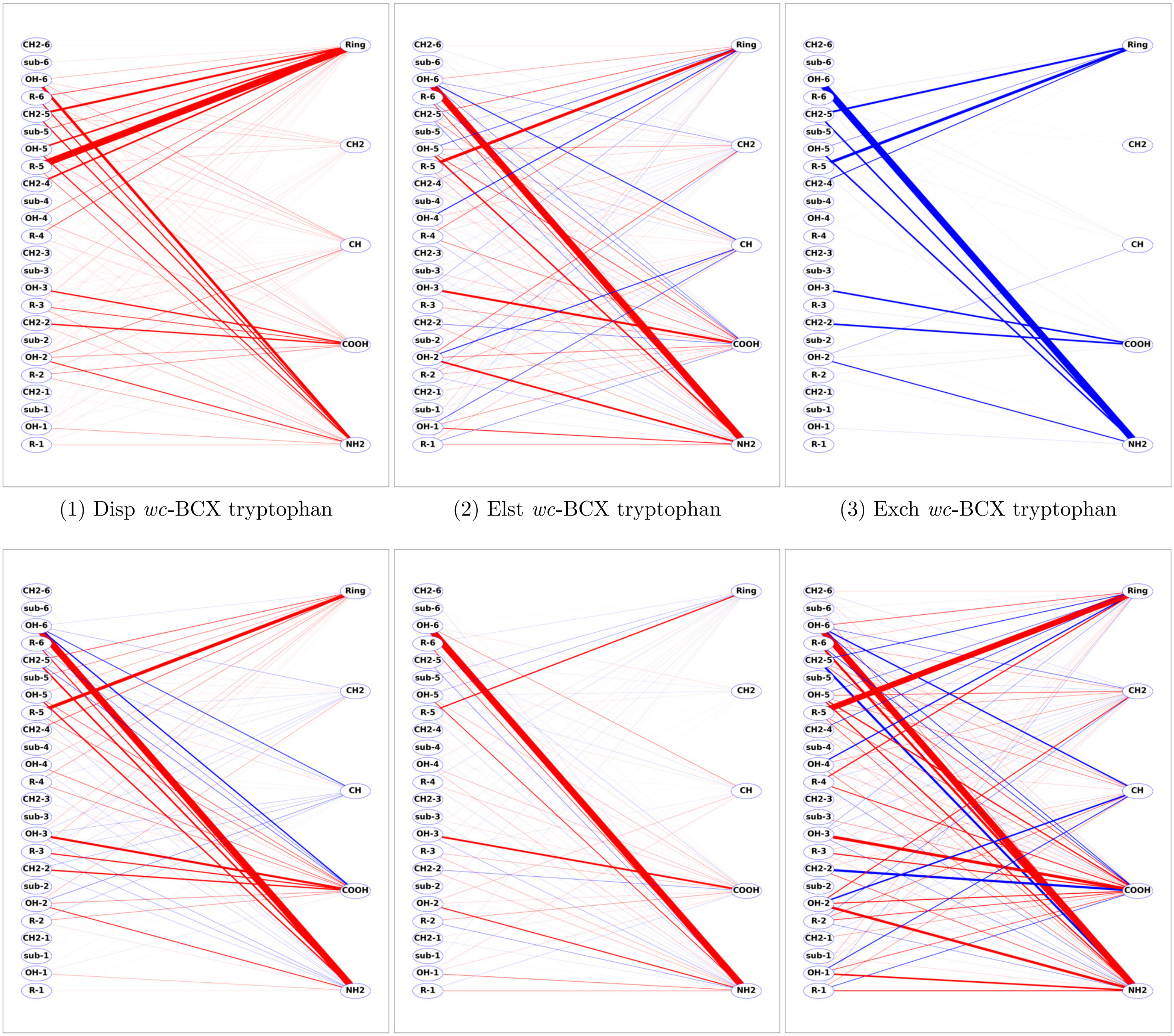

(1) Disp *wc*-BCX tryptophan

(2) Elst *wc*-BCX tryptophan

(3) Exch *wc*-BCX tryptophan

(4) IndAB *wc*-BCX tryptophan

(5) IndBA *wc*-BCX tryptophan

(6) Total *wc*-BCX tryptophan

Figure S103: The F-SAPT partitioning for *wc*-BCX tryptophan

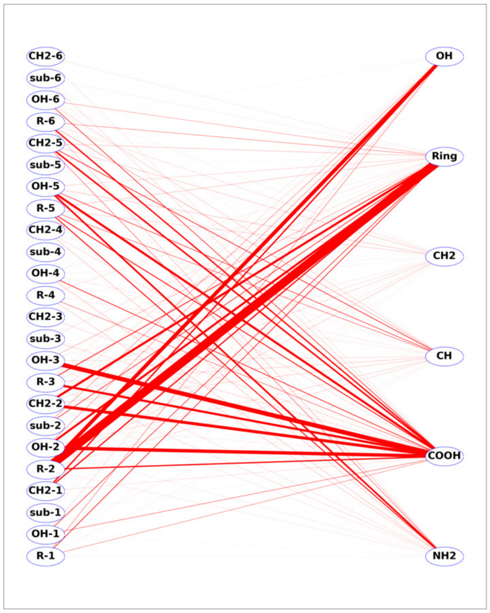

(1) Disp *wc*-BCX tyrosine

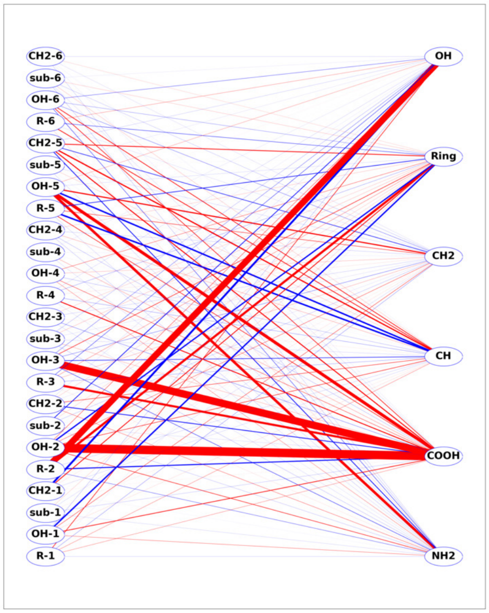

(2) Elst *wc*-BCX tyrosine

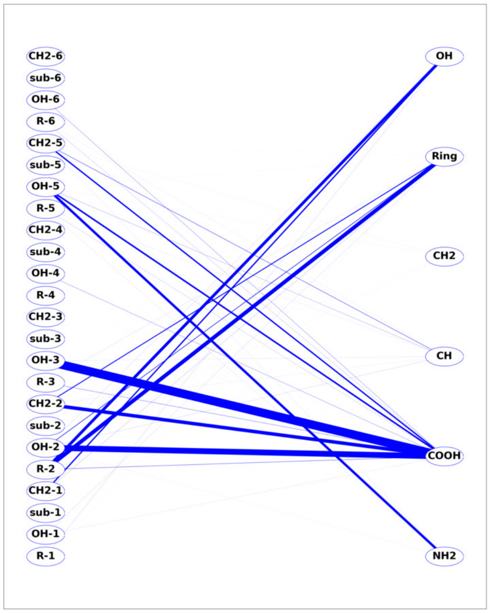

(3) Exch *wc*-BCX tyrosine

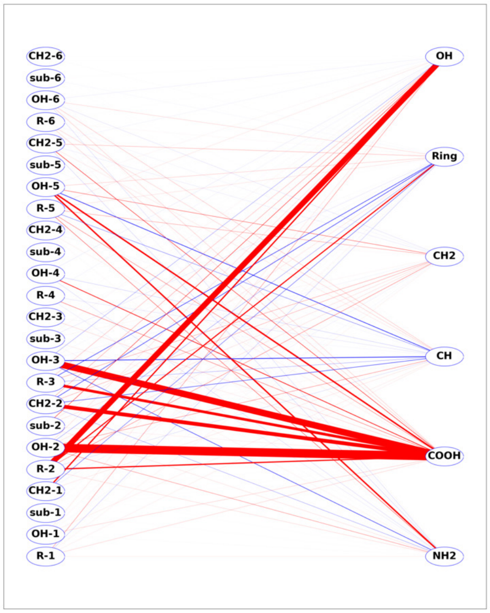

(4) IndAB *wc*-BCX tyrosine

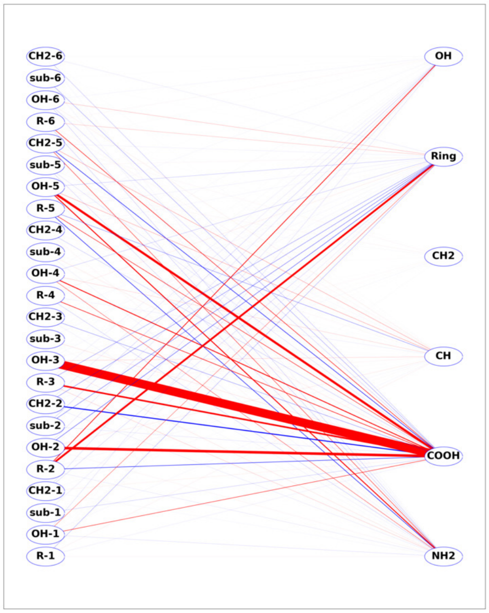

(5) IndBA *wc*-BCX tyrosine

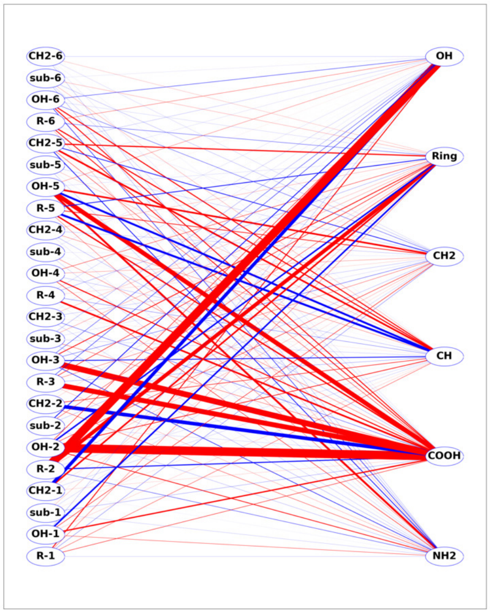

(6) Total *wc*-BCX tyrosine

Figure S104: The F-SAPT partitioning for *wc*-BCX tyrosine

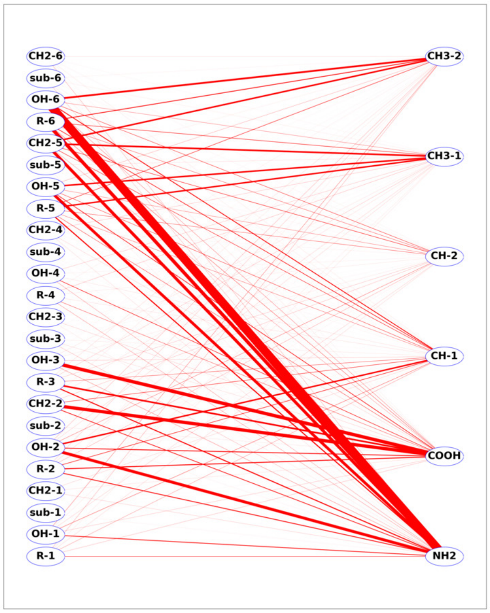

(1) Disp *wc*-BCX valine

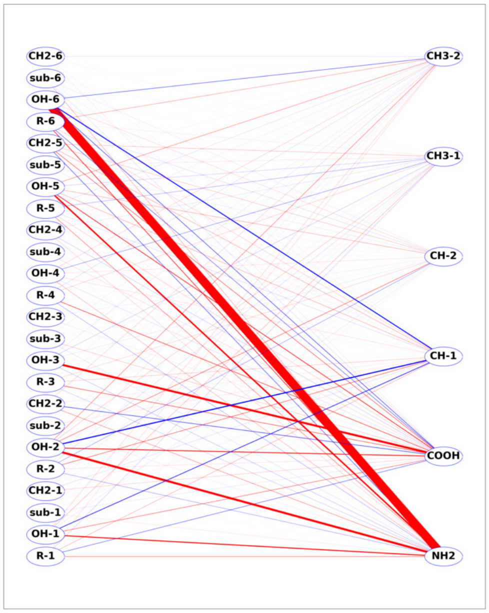

(2) Elst *wc*-BCX valine

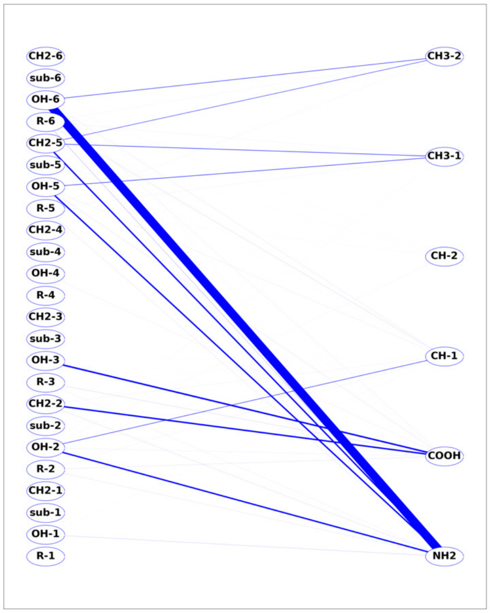

(3) Exch *wc*-BCX valine

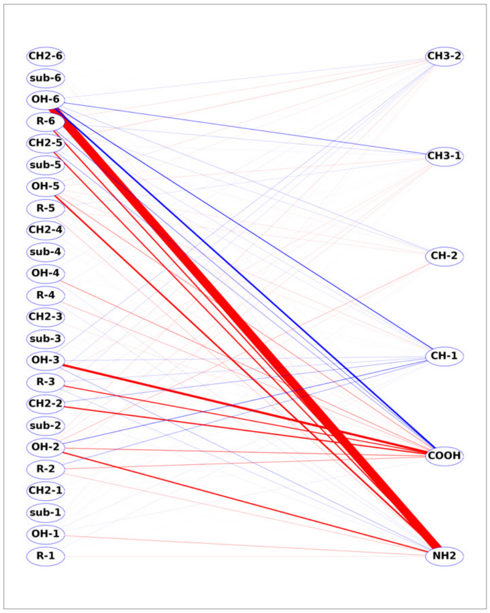

(4) IndAB *wc*-BCX valine

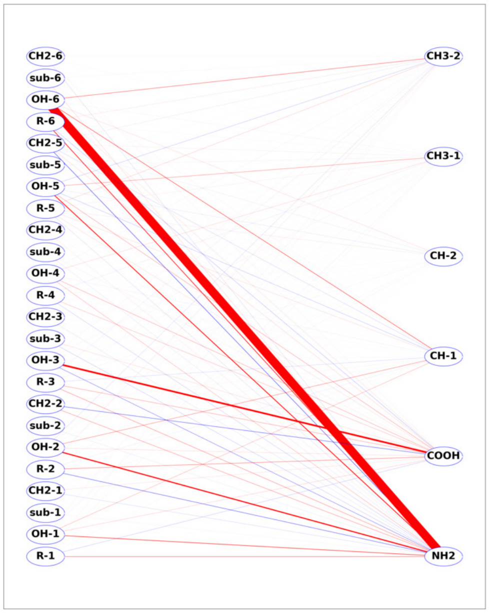

(5) IndBA *wc*-BCX valine

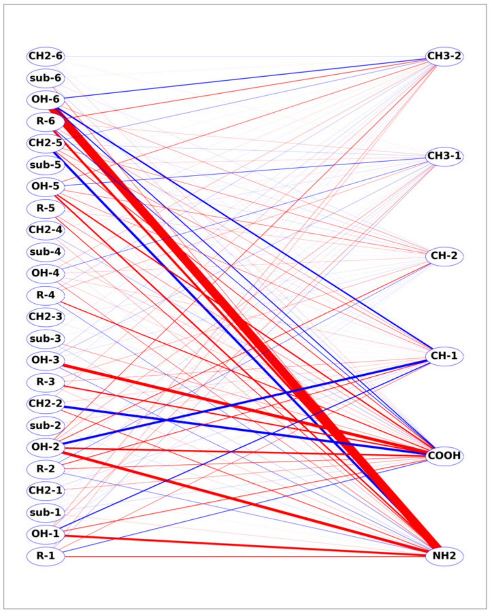

(6) Total *wc*-BCX valine

Figure S105: The F-SAPT partitioning for *wc*-BCX valine

Table S1: Dihedral angles for the amino acid + CX complexes under study. The structures of amino acid + CX were optimized using the def2-TZVP basis set at the DFT level with the B97-D3 functional.

al-CX

	R1aR2	R1bR2	R2aR3	R2bR3	R3aR4	R3bR4	R4aR5	R4bR5	R5aR6	R5bR6	R6aR1	R6bR1
	-86.53	90.23	120.08	-15.81	98.47	-83.30	83.70	-93.64	11.36	-103.98	-98.28	82.60
Ala	-82.74	94.27	119.60	-13.92	96.11	-85.01	81.47	-112.09	30.04	-92.88	-101.69	78.06
Asn	-89.23	93.12	103.18	-20.06	108.75	-79.20	81.04	-103.06	18.68	-106.84	-97.37	86.64
AspH	-83.81	97.84	105.21	-5.32	99.45	-84.53	86.78	-87.67	-5.31	-98.96	-102.45	80.45
Cys	-85.47	96.06	97.35	-6.72	97.02	-83.83	84.70	-101.37	8.95	-108.76	-95.72	86.77
Gln	-85.00	91.85	98.03	-7.09	99.48	-85.66	89.88	-116.80	22.34	-107.36	-97.12	87.00
GluH	-81.69	99.01	105.10	-6.10	91.78	-85.11	82.78	-105.89	10.40	-108.33	-92.43	86.59
Gly	-87.22	92.60	98.94	-8.20	102.09	-76.05	78.38	-108.87	16.98	-108.17	-95.24	86.13
HisD	-83.20	101.06	112.92	-8.98	90.93	-91.19	84.23	-109.71	30.77	-98.29	-102.08	79.68
HisE	-85.80	95.78	81.44	5.67	106.56	-78.01	83.18	-99.20	-5.09	-101.47	-103.00	82.34
Ile	-79.68	100.34	107.35	-6.49	94.47	-90.01	84.08	-114.88	26.05	-92.50	-103.82	80.16
Leu	-84.56	98.79	103.48	-11.34	101.11	-83.53	83.97	-92.78	4.80	-104.88	-99.21	82.96
Lys	-85.99	98.24	109.42	-6.37	93.57	-86.77	85.72	-94.30	7.32	-102.02	-99.90	83.54
Met	-80.02	111.93	105.99	-1.99	88.21	-91.59	81.42	-110.10	20.57	-91.59	-98.29	79.93
Phe	-84.30	91.30	120.48	-11.05	87.62	-91.86	91.55	-88.06	11.83	-111.05	-98.22	81.90
Pro	-86.94	92.61	90.03	-8.55	108.20	-78.87	82.52	-110.12	13.49	-109.57	-96.34	86.70
Ser	-77.49	99.51	101.07	-3.47	93.25	-84.65	83.12	-103.47	11.99	-105.58	-101.18	80.72
Thr	-74.52	100.82	98.78	-6.04	99.48	-82.90	81.19	-109.03	10.55	-98.03	-100.60	80.37
Trp	-81.66	96.22	121.94	-28.23	93.99	-87.52	84.91	-94.24	24.19	-108.58	-97.95	81.12
Tyr	-83.47	94.54	108.34	-11.25	97.31	-78.78	79.00	-107.94	22.49	-107.46	-98.88	82.57
Val	-86.75	94.17	104.63	-8.01	98.71	-82.18	82.98	-94.90	5.54	-107.00	-99.18	85.71

pc-CX

	R1aR2	R1bR2	R2aR3	R2bR3	R3aR4	R3bR4	R4aR5	R4bR5	R5aR6	R5bR6	R6aR1	R6bR1
	81.21	-106.71	-84.61	105.46	95.33	-85.41	81.21	-106.71	-84.61	105.46	95.33	-85.41
Ala	78.28	-107.21	-81.58	101.71	99.81	-74.22	74.95	-106.12	-80.96	102.78	93.48	-85.52
Asn	78.50	-103.78	-89.19	94.95	94.00	-86.64	87.46	-94.18	-98.08	85.39	105.53	-76.87
AspH	82.35	-104.92	-86.50	103.23	92.75	-85.41	83.46	-101.76	-88.62	98.09	97.25	-84.74
Cys	80.60	-105.35	-84.47	106.95	95.69	-86.32	80.52	-107.07	-83.23	108.34	94.37	-86.59
Gln	80.53	-101.00	-87.23	95.45	96.19	-83.16	78.75	-107.36	-83.49	101.70	93.53	-76.38
GluH	81.61	-99.50	-93.75	89.23	99.91	-84.04	86.18	-93.42	-99.51	85.30	105.13	-80.71
Gly	82.40	-101.11	-89.81	95.32	97.52	-83.72	80.04	-103.43	-89.23	92.78	102.38	-79.88
HisD	80.93	-116.15	-79.19	97.19	100.27	-82.92	82.72	-100.05	-89.84	94.29	100.71	-79.96
HisE	82.12	-108.11	-89.58	98.31	98.23	-83.40	81.82	-106.25	-90.96	89.71	107.69	-80.43
Ile	81.64	-104.19	-86.88	104.52	96.23	-85.17	80.48	-107.09	-84.77	106.04	94.78	-85.11
Leu	81.05	-106.00	-88.21	90.42	103.84	-81.92	82.49	-100.31	-91.77	92.75	100.21	-81.16
Lys	84.69	-104.32	-85.49	107.23	97.86	-84.34	79.26	-109.30	-84.46	117.64	88.71	-93.59
Met	81.03	-107.54	-85.13	106.74	94.84	-86.84	80.54	-107.73	-84.74	103.16	99.07	-83.36
Phe	80.34	-102.64	-89.43	94.67	97.82	-83.16	79.69	-103.34	-90.64	88.37	107.83	-78.03
Pro	82.04	-103.27	-86.90	100.84	95.22	-85.48	83.83	-103.27	-87.34	101.10	95.58	-84.76
Ser	82.44	-100.71	-92.11	90.38	101.15	-82.58	84.29	-98.03	-93.66	91.73	99.51	-82.71
Thr	74.59	-107.04	-82.40	108.09	95.74	-85.78	80.77	-104.29	-84.00	105.54	97.77	-83.73
Trp	80.34	-108.42	-81.96	101.40	94.80	-84.85	83.20	-100.59	-88.14	97.60	98.53	-82.57
Tyr	80.91	-105.93	-87.34	96.28	103.64	-80.50	79.44	-108.97	-85.33	103.51	95.64	-84.91
Val	82.94	-101.79	-91.10	99.52	101.22	-82.98	79.74	-109.26	-86.52	103.44	97.10	-85.54

wc-CX

	R1aR2	R1bR2	R2aR3	R2bR3	R3aR4	R3bR4	R4aR5	R4bR5	R5aR6	R5bR6	R6aR1	R6bR1
	-74.58	104.06	-92.95	-17.50	-75.82	105.21	-74.58	104.06	-92.95	-17.50	-75.82	105.21
Ala	-75.15	104.19	-89.40	18.83	-88.85	93.22	-75.95	104.23	-90.89	-7.50	-75.11	106.16
Asn	-74.93	105.64	-89.73	-11.50	-75.56	109.36	-75.62	103.37	-88.52	21.81	-90.58	90.24
AspH	-79.92	96.53	-74.08	-20.20	-77.09	104.55	-77.50	100.29	-85.63	-14.47	-77.24	106.45
Cys	-75.97	102.90	-88.69	-6.57	-80.56	101.84	-78.53	101.07	-88.20	-17.74	-75.40	106.02
Gln	-74.98	103.90	-88.81	-9.20	-82.14	98.20	-77.95	102.77	-88.29	-23.87	-78.30	104.90
GluH	-78.11	99.49	-88.20	-18.86	-77.50	105.02	-75.16	102.21	-91.83	-15.17	-78.19	106.07
Gly	-75.00	105.20	-90.08	18.27	-91.03	91.67	-74.99	103.92	-92.40	-11.66	-74.77	106.98
HisD	-73.84	101.88	-98.82	16.14	-86.54	95.29	-76.05	100.06	-92.11	-26.63	-71.28	114.94
HisE	-77.22	116.26	-96.89	18.08	-88.38	92.44	-75.21	110.61	-90.30	0.10	-78.02	101.17
Ile	-74.86	105.61	-92.45	18.38	-87.41	94.82	-76.01	104.85	-89.64	-4.39	-76.73	105.14
Leu	-71.38	101.02	-105.07	29.94	-80.99	99.64	-74.29	101.28	-101.59	6.43	-71.75	109.47
Lys	-77.22	103.83	-90.13	-21.96	-79.05	103.53	-76.18	104.36	-87.69	-22.98	-79.63	103.02
Met	-74.99	105.67	-91.71	-6.72	-75.33	105.60	-75.22	104.79	-88.15	19.99	-90.45	91.51
Phe	-75.16	103.82	-89.20	19.62	-89.80	92.55	-75.33	103.88	-92.82	-9.64	-73.48	107.82
Pro	-74.84	105.15	-89.98	-1.10	-82.11	100.77	-75.75	99.77	-94.18	-6.50	-74.87	102.60
Ser	-76.89	103.85	-88.83	-1.52	-73.66	106.85	-78.88	101.04	-84.46	20.23	-86.04	93.05
Thr	-74.66	106.71	-95.54	19.64	-89.66	92.71	-74.31	103.58	-96.62	-10.91	-72.36	108.79
Trp	-70.69	107.88	-100.08	-10.68	-75.68	107.33	-74.22	107.76	-98.34	16.31	-90.00	91.30
Tyr	-76.82	103.08	-88.44	-2.06	-76.85	105.66	-74.16	108.52	-96.59	17.79	-84.97	96.84
Val	-74.70	104.85	-93.33	18.57	-86.72	95.44	-75.56	104.20	-92.57	-4.84	-74.88	106.87

Table S2: Dihedral angles for the amino acid + BCX complexes under study. The structures of amino acid + BCX were optimized using the def2-TZVP basis set at the DFT level with the B97-D3 functional.

	R1aR2	R1bR2	R2aR3	R2bR3	R3aR4	R3bR4	R4aR5	R4bR5	R5aR6	R5bR6	R6aR1	R6bR1
						al-BCX						
	R1aR2	R1bR2	R2aR3	R2bR3	R3aR4	R3bR4	R4aR5	R4bR5	R5aR6	R5bR6	R6aR1	R6bR1
	-84.01	89.72	121.52	-13.67	97.28	-81.39	81.76	-92.61	9.38	-103.53	-99.38	79.85
Ala	-78.15	102.69	116.50	-2.97	89.77	-91.87	82.68	-105.23	22.10	-84.63	-110.54	75.13
Asn	-82.50	97.51	108.28	-10.81	95.99	-83.53	83.02	-112.38	24.33	-102.10	-98.71	82.87
AspH	-80.28	118.83	101.69	-2.08	88.38	-91.49	80.85	-100.96	10.27	-96.93	-94.42	80.58
Cys	-84.80	94.26	109.17	-1.32	98.17	-85.72	89.46	-81.59	-11.37	-102.03	-104.23	79.91
Gln	-80.65	97.92	109.93	-6.63	91.08	-86.03	80.91	-103.58	14.26	-103.78	-96.65	83.11
GluH	-81.24	96.07	107.49	-4.65	91.03	-87.30	86.12	-98.28	5.50	-111.26	-94.44	84.26
Gly	-79.47	95.33	111.89	-10.18	86.91	-80.48	85.51	-83.33	0.33	-112.79	-104.94	81.63
HisD	-75.61	104.96	103.21	-0.22	91.51	-88.65	82.99	-101.88	1.07	-95.42	-99.74	80.02
HisE	-85.94	90.53	93.20	-3.88	102.26	-78.59	82.38	-106.24	5.77	-114.36	-91.90	88.50
Ile	-79.66	100.65	113.96	3.45	89.89	-92.35	86.37	-101.90	11.92	-84.27	-107.24	75.42
Leu	-80.51	99.29	112.33	-16.07	98.58	-91.25	86.82	-108.32	29.72	-92.47	-106.52	76.27
Lys	-86.46	88.72	110.38	-5.99	103.37	-79.30	80.47	-106.63	13.13	-99.90	-100.49	81.83
Met	-81.26	90.51	118.11	-7.59	95.06	-80.29	78.32	-110.17	24.11	-83.89	-113.90	78.12
Phe	-82.92	85.60	133.60	-36.42	95.13	-82.70	81.75	-95.69	43.15	-126.83	-90.13	79.23
Pro	-78.02	108.78	108.80	-3.34	89.01	-93.08	83.76	-108.04	17.59	-92.59	-99.55	78.48
Ser	-81.51	102.46	105.50	-9.85	96.01	-82.18	81.31	-112.76	24.28	-95.25	-101.13	79.34
Thr	-81.70	93.85	107.23	0.07	99.19	-83.72	82.37	-103.45	1.61	-102.36	-94.28	82.75
Trp	-85.90	93.10	114.05	-6.18	92.00	-87.65	90.82	-97.98	15.55	-101.69	-102.59	79.55
Tyr	-91.84	78.09	147.86	-47.23	100.17	-81.55	76.42	-101.60	43.19	-109.98	-96.21	80.39
Val	-79.49	101.70	114.19	2.70	89.63	-92.68	86.33	-102.05	11.49	-86.69	-104.28	75.63
						pc-BCX						
	R1aR2	R1bR2	R2aR3	R2bR3	R3aR4	R3bR4	R4aR5	R4bR5	R5aR6	R5bR6	R6aR1	R6bR1
	82.38	-96.07	-101.94	87.04	104.75	-79.70	82.16	-96.03	-101.93	86.87	104.87	-79.68
Ala	82.11	-98.12	-95.27	96.87	92.29	-87.21	86.73	-92.87	-102.63	85.67	105.97	-78.57
Asn	70.36	-94.11	-96.66	84.81	112.49	-75.76	69.40	-96.38	-94.67	85.42	112.18	-76.00
AspH	80.05	-98.54	-105.66	84.81	108.76	-84.07	89.69	-85.51	-117.29	85.94	110.05	-78.89
Cys	78.01	-100.36	-96.80	84.85	106.39	-75.77	81.94	-95.23	-98.94	88.29	100.20	-79.83
Gln	83.31	-94.76	-104.48	88.30	100.84	-85.13	88.59	-90.62	-109.47	85.57	105.97	-78.91
GluH	83.87	-92.61	-101.16	88.92	100.62	-83.56	82.58	-99.61	-95.16	88.80	101.66	-80.20
Gly	82.91	-92.66	-103.62	81.44	104.26	-72.83	72.29	-98.12	-104.17	80.76	107.67	-74.95
HisD	70.79	-107.39	-90.49	83.41	109.22	-79.70	84.25	-90.95	-100.37	84.52	103.98	-74.41
HisE	82.43	-95.21	-99.72	87.63	105.97	-76.20	72.24	-115.20	-85.02	87.99	107.23	-75.90
Ile	86.04	-90.19	-102.63	90.03	95.14	-80.89	80.91	-92.93	-102.68	85.84	102.31	-80.59
Leu	83.14	-101.24	-96.10	88.75	103.74	-80.01	83.26	-93.54	-104.13	85.54	107.56	-83.87
Lys	88.00	-99.55	-102.26	86.68	108.66	-79.59	82.65	-96.75	-105.16	83.69	111.35	-84.55
Met	82.51	-98.89	-95.28	89.09	101.10	-80.82	83.97	-92.48	-100.47	89.57	99.82	-83.24
Phe	87.19	-87.28	-104.99	85.67	103.10	-79.52	82.22	-96.99	-93.48	97.45	88.59	-87.36
Pro	82.44	-94.99	-104.03	86.60	101.88	-82.23	86.70	-92.61	-106.48	85.72	104.30	-78.92
Ser	79.16	-98.64	-101.05	84.59	103.64	-77.45	82.62	-95.05	-103.92	84.59	103.40	-75.30
Thr	80.61	-95.70	-102.76	83.95	105.08	-76.90	82.84	-94.47	-101.45	85.95	100.90	-79.27
Trp	82.09	-102.77	-91.90	102.93	91.22	-83.25	81.41	-93.84	-100.32	90.04	104.13	-80.37
Tyr	85.29	-91.56	-116.82	83.43	106.16	-76.63	87.75	-90.13	-117.69	85.62	102.54	-77.00
Val	86.85	-89.54	-103.41	86.26	101.15	-80.54	86.05	-90.16	-98.99	92.94	90.12	-87.71
						wc-BCX						
	R1aR2	R1bR2	R2aR3	R2bR3	R3aR4	R3bR4	R4aR5	R4bR5	R5aR6	R5bR6	R6aR1	R6bR1
	82.38	-96.07	-101.94	87.04	104.75	-79.70	82.16	-96.03	-101.93	86.87	104.87	-79.68
Ala	82.11	-98.12	-95.27	96.87	92.29	-87.21	86.73	-92.87	-102.63	85.67	105.97	-78.57
Asn	70.36	-94.11	-96.66	84.81	112.49	-75.76	69.40	-96.38	-94.67	85.42	112.18	-76.00
AspH	80.05	-98.54	-105.66	84.81	108.76	-84.07	89.69	-85.51	-117.29	85.94	110.05	-78.89
Cys	78.01	-100.36	-96.80	84.85	106.39	-75.77	81.94	-95.23	-98.94	88.29	100.20	-79.83
Gln	83.31	-94.76	-104.48	88.30	100.84	-85.13	88.59	-90.62	-109.47	85.57	105.97	-78.91
GluH	83.87	-92.61	-101.16	88.92	100.62	-83.56	82.58	-99.61	-95.16	88.80	101.66	-80.20
Gly	82.91	-92.66	-103.62	81.44	104.26	-72.83	72.29	-98.12	-104.17	80.76	107.67	-74.95
HisD	70.79	-107.39	-90.49	83.41	109.22	-79.70	84.25	-90.95	-100.37	84.52	103.98	-74.41
HisE	82.43	-95.21	-99.72	87.63	105.97	-76.20	72.24	-115.20	-85.02	87.99	107.23	-75.90
Ile	86.04	-90.19	-102.63	90.03	95.14	-80.89	80.91	-92.93	-102.68	85.84	102.31	-80.59
Leu	83.14	-101.24	-96.10	88.75	103.74	-80.01	83.26	-93.54	-104.13	85.54	107.56	-83.87
Lys	88.00	-99.55	-102.26	86.68	108.66	-79.59	82.65	-96.75	-105.16	83.69	111.35	-84.55
Met	82.51	-98.89	-95.28	89.09	101.10	-80.82	83.97	-92.48	-100.47	89.57	99.82	-83.24
Phe	87.19	-87.28	-104.99	85.67	103.10	-79.52	82.22	-96.99	-93.48	97.45	88.59	-87.36
Pro	82.44	-94.99	-104.03	86.60	101.88	-82.23	86.70	-92.61	-106.48	85.72	104.30	-78.92
Ser	79.16	-98.64	-101.05	84.59	103.64	-77.45	82.62	-95.05	-103.92	84.59	103.40	-75.30
Thr	80.61	-95.70	-102.76	83.95	105.08	-76.90	82.84	-94.47	-101.45	85.95	100.90	-79.27
Trp	82.09	-102.77	-91.90	102.93	91.22	-83.25	81.41	-93.84	-100.32	90.04	104.13	-80.37
Tyr	85.29	-91.56	-116.82	83.43	106.16	-76.63	87.75	-90.13	-117.69	85.62	102.54	-77.00
Val	86.85	-89.54	-103.41	86.26	101.15	-80.54	86.05	-90.16	-98.99	92.94	90.12	-87.71

Table S3: I-SAPT interaction energies (in mH) of *al*-CX.

1st_group	2nd_group	$E^{(10)}_{elst}$	$E^{(10)}_{exch}$	$E^{(20)}_{ind,resp}$	$E^{(20)}_{exch-ind,resp}$	$E^{(20)}_{disp}$	$E^{(20)}_{exch-disp}$	E^{HF}_{int}	E^{SAPT0}_{tot}
OH-1	OH-2	-23.842	22.103	-11.377	7.069	-3.841	0.928	-8.895	-11.808
OH-1	OH-3	-0.253	0.001	-0.004	0.000	-0.007	0.000	-0.255	-0.262
OH-1	OH-4	-0.394	0.000	-0.001	0.000	-0.002	0.000	-0.395	-0.397
OH-1	OH-5	-0.326	0.000	-0.003	0.000	-0.005	0.000	-0.329	-0.334
OH-1	OH-6	-22.575	20.793	-11.054	7.284	-3.667	0.905	-8.392	-11.154
OH-2	OH-3	-0.104	0.146	-0.114	0.046	-0.102	0.006	-0.050	-0.147
OH-2	OH-4	-0.576	0.000	-0.004	0.000	-0.007	0.000	-0.580	-0.588
OH-2	OH-5	-0.392	0.000	-0.004	0.000	-0.008	0.000	-0.396	-0.403
OH-2	OH-6	-2.784	0.082	-0.158	0.037	-0.176	0.007	-2.828	-2.997
OH-3	OH-4	-23.617	22.445	-12.070	8.039	-3.869	0.980	-8.268	-11.157
OH-3	OH-5	-3.789	0.121	-0.181	0.055	-0.206	0.009	-3.801	-3.998
OH-3	OH-6	0.386	0.000	-0.011	0.000	-0.016	0.000	0.376	0.360
OH-4	OH-5	-24.335	22.801	-11.925	7.647	-3.888	0.972	-8.765	-11.681
OH-4	OH-6	-0.738	0.000	-0.006	0.000	-0.008	0.000	-0.744	-0.752
OH-5	OH-6	-1.720	0.025	-0.047	0.007	-0.064	0.002	-1.738	-1.800
OH-1	Ph-2	9.329	0.396	-1.336	0.582	-0.673	0.027	8.942	8.296
OH-1	Ph-3	-0.647	0.042	-0.076	0.018	-0.089	0.003	-0.673	-0.759
OH-1	Ph-4	0.389	0.000	-0.006	0.000	-0.013	0.000	0.383	0.369
OH-1	Ph-5	0.965	0.004	-0.029	0.002	-0.060	0.001	0.942	0.883
OH-1	Ph-6	-6.068	0.556	-1.418	0.890	-0.649	0.045	-6.098	-6.702
OH-2	Ph-1	-6.594	0.422	-1.169	0.650	-0.775	0.044	-6.770	-7.501
OH-2	Ph-3	-6.868	8.925	-7.476	5.321	-3.482	0.597	-1.573	-4.458
OH-2	Ph-4	0.306	0.002	-0.025	0.005	-0.040	0.000	0.288	0.248
OH-2	Ph-5	0.573	0.000	-0.010	0.000	-0.030	0.000	0.563	0.533
OH-2	Ph-6	-1.955	0.010	-0.067	0.018	-0.074	0.001	-1.995	-2.068
OH-3	Ph-1	0.266	0.000	-0.003	0.000	-0.011	0.000	0.263	0.252
OH-3	Ph-2	-0.911	0.400	-0.787	0.620	-0.271	0.030	-0.696	-0.937
OH-3	Ph-4	10.379	0.408	-1.377	0.497	-0.926	0.033	9.874	8.982
OH-3	Ph-5	0.009	0.012	-0.063	0.020	-0.107	0.001	-0.023	-0.129
OH-3	Ph-6	-0.178	0.001	-0.010	0.001	-0.022	0.000	-0.187	-0.209
OH-4	Ph-1	0.185	0.000	-0.001	0.000	-0.003	0.000	0.183	0.180
OH-4	Ph-2	-0.136	0.005	-0.017	0.010	-0.018	0.000	-0.139	-0.156
OH-4	Ph-3	-6.297	0.580	-1.507	0.957	-0.671	0.048	-6.329	-6.953
OH-4	Ph-5	8.808	0.409	-1.330	0.592	-0.654	0.026	8.447	7.820
OH-4	Ph-6	0.717	0.004	-0.018	0.006	-0.019	0.000	0.709	0.691
OH-5	Ph-1	0.088	0.000	-0.005	0.000	-0.009	0.000	0.083	0.074
OH-5	Ph-2	0.029	0.000	-0.006	0.000	-0.017	0.000	0.024	0.006
OH-5	Ph-3	-1.349	0.011	-0.065	0.019	-0.087	0.001	-1.384	-1.469
OH-5	Ph-4	-6.559	0.354	-1.072	0.570	-0.835	0.043	-6.789	-7.581
OH-5	Ph-6	2.513	0.465	-0.897	0.630	-0.270	0.030	2.692	2.452
OH-6	Ph-1	10.409	0.390	-1.329	0.472	-0.911	0.033	9.905	9.027
OH-6	Ph-2	0.173	0.006	-0.054	0.009	-0.114	0.001	0.134	0.021
OH-6	Ph-3	-0.368	0.000	-0.026	0.000	-0.053	0.000	-0.393	-0.446
OH-6	Ph-4	0.657	0.000	-0.025	0.001	-0.038	0.000	0.634	0.596
OH-6	Ph-5	4.193	1.380	-1.320	0.706	-1.393	0.138	4.849	3.594

Table S4: A comparison of the SSMF3 fragmentation scheme with the unfragmented results for supermolecular and SAPT0 interaction energies for amino acid + *al*-CX complex. HF, MP2, and SCS-MP2 interaction energies, as well as SAPT0 components and total SAPT0 interaction energies, are presented. Energies are in millihartree

	No Fragmentation									
	$E^{(10)}_{elst}$	$E^{(10)}_{exch}$	$E^{(20)}_{ind,resp}$	$E^{(20)}_{exch-ind,resp}$	$E^{(20)}_{disp}$	$E^{(20)}_{exch-disp}$	E^{HF}_{int}	E^{SAPT0}_{tot}	E^{MP2}_{int}	$E^{SCS-MP2}_{int}$
Ala	-66.511	97.946	-46.244	29.354	-56.574	8.258	-0.086	-48.402	-40.813	-29.637
Asn	-86.888	110.433	-58.943	35.023	-57.001	8.461	-20.297	-68.837	-58.199	-47.113
AspH	-67.476	94.486	-46.685	29.850	-55.251	7.647	-3.743	-51.346	-43.807	-32.962
Cys	-84.532	104.660	-51.560	31.424	-58.791	8.604	-15.811	-65.998	-54.202	-42.686
Gln	-102.619	128.131	-58.977	36.001	-68.946	10.127	-16.477	-75.296	-60.867	-47.283
GluH	-59.400	66.858	-30.384	18.749	-53.972	6.523	-11.791	-59.240	-46.690	-36.128
Gly	-74.734	91.281	-46.798	28.239	-50.562	7.491	-16.605	-59.676	-49.170	-39.275
HisD	-57.099	67.446	-30.630	18.306	-54.576	6.384	-10.228	-58.420	-48.582	-37.601
HisE	-67.587	96.522	-49.049	30.577	-63.662	8.701	-5.170	-60.130	-51.110	-38.982
Ile	-56.079	76.037	-32.572	21.456	-61.625	7.591	0.430	-53.604	-43.122	-30.991
Leu	-58.328	83.653	-39.000	25.015	-57.618	7.485	-0.308	-50.441	-43.383	-32.246
Lys	-75.732	102.650	-48.311	29.825	-62.988	8.689	-7.944	-62.242	-52.678	-40.493
Met	-58.322	74.616	-33.669	21.212	-62.184	7.571	-4.888	-59.501	-48.595	-36.388
Phe	-52.761	68.503	-29.500	18.249	-53.481	6.393	-4.526	-51.614	-40.976	-30.546
Pro	-85.332	115.454	-59.798	36.191	-58.267	8.771	-13.833	-63.330	-53.377	-42.020
Ser	-61.679	74.400	-31.141	19.750	-53.122	6.839	-6.247	-52.531	-43.869	-33.231
Thr	-62.370	72.339	-29.794	18.937	-57.117	7.069	-8.288	-58.336	-49.241	-37.798
Trp	-53.373	79.108	-34.654	24.261	-67.661	8.347	5.740	-53.574	-47.286	-33.888
Tyr	-60.824	73.332	-31.702	20.102	-60.059	7.360	-6.874	-59.573	-50.240	-38.592
Val	-60.088	78.762	-36.571	22.854	-57.711	7.250	-5.408	-55.869	-47.686	-36.399
	SSMF3									
	$E^{(10)}_{elst}$	$E^{(10)}_{exch}$	$E^{(20)}_{ind,resp}$	$E^{(20)}_{exch-ind,resp}$	$E^{(20)}_{disp}$	$E^{(20)}_{exch-disp}$	E^{HF}_{int}	E^{SAPT0}_{tot}	E^{MP2}_{int}	$E^{SCS-MP2}_{int}$
Ala	-66.300	98.545	-46.889	29.565	-55.788	8.018	0.280	-47.491	-40.050	-28.956
Asn	-87.214	110.554	-58.302	34.547	-56.501	8.350	-19.526	-67.677	-56.892	-45.892
AspH	-67.289	94.786	-46.201	29.289	-54.632	7.540	-2.819	-49.911	-42.332	-31.598
Cys	-84.844	104.790	-51.379	31.082	-57.931	8.373	-15.690	-65.247	-53.399	-41.980
Gln	-102.623	128.864	-58.796	35.808	-68.303	9.988	-15.685	-74.000	-59.502	-46.003
GluH	-60.142	66.946	-30.540	18.748	-53.011	6.293	-12.626	-59.345	-46.809	-36.317
Gly	-75.115	91.426	-46.609	27.900	-49.778	7.287	-16.405	-58.895	-48.528	-38.706
HisD	-58.078	67.589	-31.454	18.365	-53.804	6.183	-12.139	-59.760	-50.083	-39.126
HisE	-67.516	96.877	-48.386	30.317	-63.127	8.631	-3.788	-58.285	-49.142	-37.133
Ile	-55.924	76.113	-32.854	21.330	-60.687	7.339	0.113	-53.236	-42.773	-30.724
Leu	-58.295	83.904	-38.575	24.579	-56.922	7.324	0.516	-49.082	-41.929	-30.912
Lys	-75.895	103.101	-48.044	29.376	-62.407	8.514	-7.048	-60.942	-51.269	-39.176
Met	-59.417	74.718	-34.174	21.124	-61.100	7.303	-6.766	-60.564	-49.979	-37.816
Phe	-52.641	68.462	-29.389	18.181	-52.949	6.265	-4.018	-50.702	-39.936	-29.586
Pro	-85.230	115.797	-58.909	35.794	-57.599	8.619	-12.185	-61.166	-51.257	-39.990
Ser	-61.422	74.679	-31.652	19.900	-52.164	6.546	-6.443	-52.061	-43.522	-32.972
Thr	-62.273	72.461	-30.161	18.980	-56.166	6.814	-8.599	-57.952	-48.966	-37.620
Trp	-53.236	79.355	-34.521	24.093	-67.218	8.232	6.051	-52.935	-46.515	-33.188
Tyr	-60.770	73.629	-32.317	20.267	-59.122	7.105	-7.401	-59.418	-50.131	-38.563
Val	-59.626	78.955	-36.498	22.475	-56.993	7.037	-4.804	-54.760	-46.342	-35.187

Table S5: A comparison of the SSMF3 fragmentation scheme with the unfragmented results for supermolecular and SAPT0 interaction energies for amino acid + *pc*-CX complex. HF, MP2, and SCS-MP2 interaction energies, as well as SAPT0 components and total SAPT0 interaction energies, are presented. Energies are in millihartree

	No Fragmentation									
	$E^{(10)}_{elst}$	$E^{(10)}_{exch}$	$E^{(20)}_{ind,resp}$	$E^{(20)}_{exch-ind,resp}$	$E^{(20)}_{disp}$	$E^{(20)}_{exch-disp}$	E^{HF}_{int}	E^{SAPT0}_{tot}	E^{MP2}_{int}	$E^{SCS-MP2}_{int}$
Ala	-55.113	62.693	-29.486	18.193	-30.112	4.566	-12.025	-37.571	-30.012	-24.342
Asn	-34.136	37.688	-14.354	10.096	-31.154	3.398	-3.350	-31.106	-23.622	-17.775
AspH	-26.622	32.757	-13.509	8.736	-26.938	2.751	-1.450	-25.637	-19.800	-14.675
Cys	-14.631	19.574	-7.016	4.967	-19.711	1.774	1.371	-16.566	-13.064	-9.165
Gln	-50.095	57.311	-26.055	16.187	-35.378	4.457	-9.701	-40.622	-30.752	-24.056
GluH	-41.790	45.630	-20.656	13.288	-29.835	3.583	-8.460	-34.712	-26.265	-20.761
Gly	-33.437	35.977	-15.939	9.697	-20.125	2.612	-7.984	-25.497	-19.370	-15.643
HisD	-39.837	52.540	-22.550	16.312	-47.012	5.367	2.014	-39.630	-32.119	-23.063
HisE	-24.949	36.153	-15.155	11.786	-39.352	4.267	4.986	-30.099	-25.301	-17.608
Ile	-22.192	39.788	-14.672	11.352	-41.197	4.351	11.115	-25.731	-20.961	-12.944
Leu	-30.955	43.766	-16.135	11.803	-38.547	4.127	4.893	-29.527	-23.221	-15.810
Lys	-19.312	38.923	-12.384	9.442	-43.176	4.534	13.446	-25.196	-21.970	-13.255
Met	-17.447	25.627	-8.626	5.954	-26.880	2.348	3.517	-21.015	-16.608	-11.261
Phe	-39.893	56.243	-24.854	18.446	-49.130	6.037	4.626	-38.467	-31.793	-22.489
Pro	-20.118	34.235	-12.811	9.561	-38.956	4.087	7.711	-27.158	-23.066	-15.196
Ser	-30.005	34.680	-14.420	9.586	-24.822	2.761	-3.506	-25.566	-19.690	-15.008
Thr	-37.853	39.620	-17.701	10.500	-22.471	2.811	-10.427	-30.087	-22.549	-18.407
Trp	-35.839	54.524	-22.220	16.868	-55.662	6.201	8.864	-40.596	-33.864	-23.224
Tyr	-17.303	27.443	-10.792	7.041	-31.439	3.132	3.969	-24.338	-20.328	-13.919
Val	-23.078	37.511	-12.952	9.634	-39.479	4.192	8.003	-27.284	-22.444	-14.571
	SSMF3									
	$E^{(10)}_{elst}$	$E^{(10)}_{exch}$	$E^{(20)}_{ind,resp}$	$E^{(20)}_{exch-ind,resp}$	$E^{(20)}_{disp}$	$E^{(20)}_{exch-disp}$	E^{HF}_{int}	E^{SAPT0}_{tot}	E^{MP2}_{int}	$E^{SCS-MP2}_{int}$
Ala	-55.010	62.745	-30.338	18.184	-29.685	4.477	-12.367	-37.574	-30.108	-24.467
Asn	-34.539	37.684	-16.265	10.307	-30.528	3.308	-5.544	-32.764	-25.401	-19.592
AspH	-27.185	32.898	-15.386	8.976	-26.549	2.700	-3.586	-27.435	-21.641	-16.539
Cys	-15.027	19.642	-8.056	5.061	-19.485	1.755	0.062	-17.668	-14.219	-10.338
Gln	-50.179	57.504	-27.754	16.404	-34.847	4.375	-11.237	-41.708	-32.025	-25.350
GluH	-42.054	45.703	-22.664	13.556	-29.354	3.509	-10.591	-36.436	-28.046	-22.574
Gly	-33.708	35.978	-17.025	9.787	-19.843	2.563	-9.206	-26.486	-20.442	-16.719
HisD	-40.067	52.595	-24.121	16.509	-46.518	5.306	0.307	-40.904	-33.523	-24.499
HisE	-25.009	36.267	-16.213	11.971	-39.103	4.264	4.044	-30.795	-26.044	-18.382
Ile	-22.714	40.096	-15.271	11.488	-40.650	4.275	10.360	-26.015	-21.194	-13.260
Leu	-31.365	43.883	-17.806	12.007	-38.149	4.071	2.914	-31.164	-24.968	-17.575
Lys	-19.368	39.003	-12.780	9.489	-42.750	4.471	12.978	-25.301	-22.118	-13.449
Met	-17.806	25.754	-10.149	6.085	-26.642	2.343	1.786	-22.513	-18.015	-12.708
Phe	-40.255	56.230	-26.301	18.610	-48.629	5.937	2.951	-39.741	-33.185	-23.900
Pro	-20.123	34.307	-13.242	9.626	-38.612	4.052	7.331	-27.229	-23.104	-15.290
Ser	-30.433	34.780	-16.083	9.781	-24.460	2.711	-5.505	-27.254	-21.479	-16.808
Thr	-38.121	39.504	-18.469	10.517	-22.180	2.762	-11.579	-30.996	-23.534	-19.400
Trp	-36.187	54.536	-23.930	17.056	-55.149	6.135	6.836	-42.179	-35.600	-24.987
Tyr	-17.623	27.479	-11.285	7.127	-31.195	3.112	3.237	-24.846	-20.948	-14.554
Val	-23.228	37.692	-13.490	9.764	-39.038	4.137	7.542	-27.359	-22.571	-14.749

Table S6: A comparison of the SSMF3 fragmentation scheme with the unfragmented results for supermolecular and SAPT0 interaction energies for amino acid + *wc*-CX complex. HF, MP2, and SCS-MP2 interaction energies, as well as SAPT0 components and total SAPT0 interaction energies, are presented. Energies are in millihartree

	$E^{(10)}_{elst}$	$E^{(10)}_{exch}$	$E^{(20)}_{ind,resp}$	$E^{(20)}_{exch-ind,resp}$	$E^{(20)}_{disp}$	$E^{(20)}_{exch-disp}$	E^{HF}_{int}	E^{SAPT0}_{tot}	E^{MP2}_{int}	$E^{SCS-MP2}_{int}$
					No Fragmentation					
Ala	-72.884	80.455	-39.285	23.007	-36.439	5.665	-20.904	-51.678	-42.154	-35.055
Asn	-92.079	108.997	-55.302	31.454	-45.361	7.106	-23.809	-62.064	-49.442	-40.710
AspH	-49.717	48.131	-22.429	12.494	-30.021	3.581	-17.615	-44.056	-34.277	-28.413
Cys	-54.894	57.958	-27.841	15.979	-30.850	4.102	-17.669	-44.417	-34.339	-28.452
Gln	-83.647	85.179	-41.127	22.207	-39.288	5.699	-31.393	-64.981	-49.541	-42.231
GluH	-71.491	73.171	-37.095	19.877	-34.024	4.827	-28.005	-57.202	-43.969	-37.578
Gly	-74.211	80.043	-39.474	23.001	-34.674	5.579	-22.909	-52.005	-42.698	-35.909
HisD	-89.046	104.420	-52.597	31.528	-52.916	7.882	-22.038	-67.072	-56.960	-46.661
HisE	-71.048	83.268	-40.817	25.906	-51.162	7.140	-13.673	-57.695	-50.003	-40.029
Ile	-68.096	75.550	-34.973	20.515	-39.448	5.492	-17.677	-51.634	-42.231	-34.467
Leu	-73.804	86.407	-42.484	25.242	-43.586	6.392	-17.017	-54.212	-43.278	-34.691
Lys	-92.131	105.094	-55.859	32.243	-44.792	7.135	-29.271	-66.928	-53.936	-45.403
Met	-77.540	90.093	-43.903	25.611	-42.446	6.311	-19.694	-55.829	-45.419	-37.128
Phe	-78.849	88.198	-43.554	25.254	-41.302	6.227	-22.354	-57.429	-46.774	-38.731
Pro	-70.665	81.086	-39.778	24.680	-46.706	6.577	-15.367	-55.496	-44.408	-35.363
Ser	-81.076	84.059	-41.457	23.296	-39.381	5.887	-27.341	-60.835	-49.357	-41.549
Thr	-77.149	85.870	-40.021	23.701	-43.481	6.282	-19.305	-56.504	-45.127	-36.441
Trp	-79.851	93.771	-43.320	27.378	-60.352	7.973	-13.928	-66.307	-54.705	-42.853
Tyr	-72.223	83.761	-40.223	26.709	-53.663	7.462	-12.652	-58.853	-49.720	-39.235
Val	-71.502	78.800	-37.333	21.794	-40.313	5.676	-19.363	-54.000	-44.013	-36.099
					SSMF3					
	$E^{(10)}_{elst}$	$E^{(10)}_{exch}$	$E^{(20)}_{ind,resp}$	$E^{(20)}_{exch-ind,resp}$	$E^{(20)}_{disp}$	$E^{(20)}_{exch-disp}$	E^{HF}_{int}	E^{SAPT0}_{tot}	E^{MP2}_{int}	$E^{SCS-MP2}_{int}$
Ala	-72.842	80.525	-38.783	22.560	-35.985	5.494	-20.815	-51.306	-41.786	-34.732
Asn	-91.959	109.179	-54.950	31.002	-44.912	6.931	-23.580	-61.561	-48.874	-40.184
AspH	-49.996	47.985	-22.246	12.348	-29.668	3.498	-17.893	-44.064	-34.215	-28.391
Cys	-54.735	57.784	-27.791	15.873	-30.441	3.972	-17.418	-43.886	-33.756	-27.922
Gln	-83.492	84.875	-41.172	21.986	-38.764	5.535	-31.615	-64.843	-49.394	-42.141
GluH	-71.637	72.897	-36.751	19.703	-33.568	4.679	-27.812	-56.701	-43.366	-37.029
Gly	-74.216	80.219	-38.988	22.589	-34.218	5.395	-22.819	-51.643	-42.309	-35.567
HisD	-89.214	104.498	-52.337	31.144	-52.366	7.691	-22.046	-66.720	-56.582	-46.338
HisE	-71.089	83.337	-40.576	25.582	-50.670	7.000	-13.864	-57.534	-49.927	-39.998
Ile	-68.104	75.530	-34.627	20.139	-39.010	5.344	-17.800	-51.465	-42.059	-34.337
Leu	-73.900	86.332	-42.401	24.965	-43.082	6.254	-17.373	-54.201	-43.279	-34.749
Lys	-92.317	104.764	-55.542	31.944	-44.184	6.926	-29.314	-66.572	-53.452	-44.988
Met	-77.440	90.218	-43.568	25.135	-41.960	6.131	-19.669	-55.497	-45.106	-36.857
Phe	-78.949	88.281	-43.034	24.735	-40.791	6.034	-22.455	-57.212	-46.545	-38.550
Pro	-71.304	80.995	-39.751	24.528	-45.959	6.373	-16.304	-55.891	-44.875	-35.908
Ser	-81.059	83.971	-41.272	22.857	-39.004	5.723	-27.717	-60.997	-49.451	-41.679
Thr	-77.386	85.846	-39.942	23.254	-42.914	6.093	-20.033	-56.854	-45.419	-36.786
Trp	-80.017	93.676	-43.375	26.951	-59.696	7.763	-14.735	-66.668	-54.996	-43.208
Tyr	-72.279	83.706	-39.935	26.362	-53.263	7.339	-12.842	-58.766	-49.665	-39.217
Val	-71.626	78.736	-36.908	21.353	-39.854	5.517	-19.587	-53.924	-43.920	-36.049

Table S7: A comparison of the SSMF3 fragmentation scheme with the unfragmented results for supermolecular and SAPT0 interaction energies for amino acid + *al*-BCX complex. HF, MP2, and SCS-MP2 interaction energies, as well as SAPT0 components and total SAPT0 interaction energies, are presented. Energies are in millihartree

	No Fragmentation									
	$E^{(10)}_{elst}$	$E^{(10)}_{exch}$	$E^{(20)}_{ind,resp}$	$E^{(20)}_{exch-ind,resp}$	$E^{(20)}_{disp}$	$E^{(20)}_{exch-disp}$	E^{HF}_{int}	E^{SAPT0}_{tot}	E^{MP2}_{int}	$E^{SCS-MP2}_{int}$
Ala	-72.586	94.819	-43.129	27.796	-58.940	8.075	-5.907	-56.772	-47.135	-35.686
Asn	-107.118	133.174	-64.800	39.473	-79.087	11.095	-18.234	-86.225	-69.605	-54.057
AspH	-76.579	91.098	-42.327	26.185	-71.923	8.849	-13.069	-76.143	-61.156	-47.134
Cys	-75.995	108.237	-52.038	32.984	-67.607	9.069	-3.042	-61.580	-51.850	-38.516
Gln	-72.572	88.558	-39.919	23.697	-71.163	8.753	-11.207	-73.617	-59.086	-45.244
GluH	-66.459	81.030	-34.100	20.985	-64.294	7.545	-7.976	-64.725	-50.318	-37.788
Gly	-68.549	90.820	-42.527	25.907	-57.926	7.899	-7.678	-57.705	-48.887	-37.279
HisD	-70.793	94.017	-39.496	27.157	-77.301	9.562	1.155	-66.584	-53.389	-38.403
HisE	-82.761	105.599	-52.117	32.282	-73.195	9.757	-13.248	-76.686	-64.527	-50.247
Ile	-58.990	88.174	-36.551	23.839	-74.143	9.050	6.787	-58.306	-46.940	-32.524
Leu	-84.040	125.391	-56.033	36.736	-81.377	11.080	5.623	-64.674	-53.886	-38.155
Lys	-70.017	110.985	-52.510	31.601	-75.269	9.778	3.209	-62.282	-52.302	-38.013
Met	-69.110	107.446	-47.500	31.731	-76.489	9.963	8.667	-57.859	-48.413	-33.407
Phe	-56.578	85.046	-34.136	22.896	-69.848	8.117	6.749	-54.982	-47.317	-33.330
Pro	-58.031	76.715	-34.178	20.935	-64.162	7.811	-3.498	-59.850	-49.209	-36.576
Ser	-97.562	128.790	-62.524	38.360	-73.285	10.684	-11.970	-74.572	-62.050	-47.454
Thr	-60.507	79.828	-29.472	20.161	-67.001	8.132	2.455	-56.414	-46.781	-33.786
Trp	-67.796	97.888	-44.665	28.653	-77.904	9.178	0.542	-68.184	-58.043	-42.835
Tyr	-79.945	122.719	-56.056	37.031	-85.777	11.132	7.688	-66.956	-56.552	-39.801
Val	-58.273	86.089	-36.087	23.372	-71.503	8.766	5.524	-57.213	-46.049	-32.158
	SSMF3									
	$E^{(10)}_{elst}$	$E^{(10)}_{exch}$	$E^{(20)}_{ind,resp}$	$E^{(20)}_{exch-ind,resp}$	$E^{(20)}_{disp}$	$E^{(20)}_{exch-disp}$	E^{HF}_{int}	E^{SAPT0}_{tot}	E^{MP2}_{int}	$E^{SCS-MP2}_{int}$
Ala	-73.076	95.300	-43.304	27.556	-57.531	7.771	-6.554	-56.314	-46.795	-35.502
Asn	-107.691	134.157	-65.191	39.533	-77.379	10.652	-18.360	-85.087	-68.380	-53.050
AspH	-77.643	91.285	-43.090	26.093	-69.919	8.321	-14.719	-76.317	-61.562	-47.744
Cys	-75.947	108.889	-51.876	32.533	-65.902	8.753	-2.217	-59.366	-49.532	-36.492
Gln	-73.423	89.134	-40.834	23.995	-69.319	8.295	-12.519	-73.542	-59.049	-45.382
GluH	-67.015	81.366	-34.461	21.078	-62.657	7.191	-8.418	-63.884	-49.379	-37.064
Gly	-68.346	91.642	-42.716	25.920	-56.422	7.491	-6.091	-55.022	-46.000	-34.679
HisD	-71.445	94.368	-40.201	27.144	-75.348	9.091	0.087	-66.170	-52.898	-38.174
HisE	-83.349	106.174	-52.575	32.182	-71.519	9.419	-13.378	-75.479	-63.455	-49.384
Ile	-59.736	88.488	-37.536	23.737	-72.379	8.656	5.113	-58.609	-47.582	-33.362
Leu	-84.040	126.126	-56.372	36.654	-79.772	10.658	5.837	-63.277	-52.664	-37.118
Lys	-70.269	111.794	-52.967	31.345	-73.404	9.374	3.707	-60.323	-50.477	-36.436
Met	-69.464	108.097	-48.197	31.806	-74.788	9.631	8.033	-57.124	-47.818	-33.032
Phe	-56.818	85.449	-34.020	22.715	-68.726	7.867	7.100	-53.759	-45.994	-32.201
Pro	-58.998	77.045	-35.023	20.993	-62.491	7.460	-5.275	-60.306	-50.108	-37.611
Ser	-97.606	129.965	-63.133	38.599	-71.646	10.233	-11.562	-72.975	-60.578	-46.180
Thr	-60.757	80.190	-29.834	20.168	-65.186	7.713	1.944	-55.529	-45.902	-33.146
Trp	-68.256	98.272	-45.208	28.379	-76.405	8.899	0.039	-67.468	-57.252	-42.269
Tyr	-79.629	123.379	-56.280	36.844	-84.442	10.814	8.485	-65.144	-54.654	-38.137
Val	-59.082	86.414	-37.030	23.269	-69.766	8.377	3.865	-57.524	-46.708	-33.006

Table S8: A comparison of the SSMF3 fragmentation scheme with the unfragmented results for supermolecular and SAPT0 interaction energies for amino acid + *pc*-BCX complex. HF, MP2, and SCS-MP2 interaction energies, as well as SAPT0 components and total SAPT0 interaction energies, are presented. Energies are in millihartree

	No Fragmentation									
	$E^{(10)}_{elst}$	$E^{(10)}_{exch}$	$E^{(20)}_{ind,resp}$	$E^{(20)}_{exch-ind,resp}$	$E^{(20)}_{disp}$	$E^{(20)}_{exch-disp}$	E^{HF}_{int}	E^{SAPT0}_{tot}	E^{MP2}_{int}	$E^{SCS-MP2}_{int}$
Ala	-24.358	40.912	-16.633	11.803	-44.745	4.860	8.209	-31.677	-25.937	-17.081
Asn	-69.279	73.027	-34.606	19.689	-35.362	4.874	-21.753	-52.241	-38.914	-32.277
AspH	-38.017	55.904	-22.077	14.719	-54.104	5.721	4.901	-43.482	-34.361	-23.615
Cys	-38.020	46.603	-20.419	13.692	-32.926	3.980	-3.198	-32.143	-25.299	-19.075
Gln	-27.349	48.027	-17.341	12.883	-55.350	5.739	12.014	-37.596	-30.219	-19.261
GluH	-31.013	50.424	-21.297	14.472	-56.548	5.835	7.393	-43.320	-35.087	-23.670
Gly	-53.093	59.566	-27.594	16.864	-28.760	4.335	-11.974	-36.400	-29.356	-23.903
HisD	-30.363	44.213	-18.976	12.395	-46.748	4.906	3.124	-38.718	-31.551	-22.482
HisE	-48.730	60.037	-27.576	17.678	-48.906	5.776	-5.666	-48.796	-40.000	-30.486
Ile	-17.854	37.891	-11.720	8.565	-47.900	4.471	13.718	-29.712	-25.475	-15.921
Leu	-22.958	44.680	-14.697	11.313	-53.452	5.451	14.683	-33.318	-27.824	-17.249
Lys	-28.166	53.969	-18.615	13.818	-59.919	6.392	16.743	-36.783	-29.900	-18.234
Met	-27.478	49.251	-19.163	14.409	-57.212	6.100	12.310	-38.802	-32.422	-20.948
Phe	-26.719	51.509	-19.796	16.138	-60.179	6.506	16.621	-37.052	-31.777	-19.840
Pro	-23.322	41.016	-14.883	10.652	-49.012	4.841	9.722	-34.448	-28.906	-19.047
Ser	-34.898	36.563	-16.355	9.975	-22.562	2.711	-8.895	-28.746	-21.953	-17.800
Thr	-38.583	43.039	-18.386	11.672	-25.957	3.190	-7.201	-29.968	-22.616	-17.847
Trp	-27.770	62.443	-23.302	17.913	-80.071	8.141	24.237	-47.693	-41.474	-25.558
Tyr	-29.375	55.410	-21.160	15.098	-66.290	6.680	14.635	-44.976	-38.648	-25.207
Val	-21.081	42.860	-13.424	9.851	-49.319	4.927	14.589	-29.803	-25.070	-15.245
	SSMF3									
	$E^{(10)}_{elst}$	$E^{(10)}_{exch}$	$E^{(20)}_{ind,resp}$	$E^{(20)}_{exch-ind,resp}$	$E^{(20)}_{disp}$	$E^{(20)}_{exch-disp}$	E^{HF}_{int}	E^{SAPT0}_{tot}	E^{MP2}_{int}	$E^{SCS-MP2}_{int}$
Ala	-24.570	41.158	-17.183	11.935	-43.753	4.696	7.648	-31.409	-25.809	-17.091
Asn	-69.223	73.264	-35.501	19.805	-34.838	4.809	-22.085	-52.114	-38.734	-32.168
AspH	-38.337	56.343	-22.605	14.888	-52.921	5.522	4.673	-42.726	-33.513	-22.972
Cys	-38.184	46.598	-21.704	13.788	-32.305	3.880	-4.537	-32.962	-26.288	-20.110
Gln	-27.695	48.361	-17.829	12.963	-54.206	5.542	11.659	-37.004	-29.421	-18.675
GluH	-31.223	50.683	-21.815	14.551	-55.456	5.659	7.060	-42.737	-34.332	-23.122
Gly	-52.869	59.579	-28.425	16.859	-28.152	4.218	-12.245	-36.179	-29.408	-23.988
HisD	-30.428	44.281	-19.636	12.465	-45.991	4.804	2.683	-38.504	-31.261	-22.324
HisE	-48.799	59.982	-28.494	17.786	-48.046	5.636	-6.571	-48.981	-40.461	-31.023
Ile	-18.058	38.101	-12.023	8.620	-46.713	4.304	13.537	-28.873	-24.577	-15.234
Leu	-22.993	44.898	-15.026	11.350	-52.530	5.303	14.545	-32.681	-27.147	-16.732
Lys	-28.800	54.405	-19.011	13.921	-58.891	6.227	16.304	-36.361	-29.381	-17.900
Met	-27.612	49.518	-19.578	14.465	-56.042	5.907	12.080	-38.055	-31.578	-20.315
Phe	-26.762	51.705	-20.139	16.297	-58.949	6.335	16.471	-36.143	-30.857	-19.131
Pro	-23.283	41.223	-15.265	10.698	-47.998	4.684	9.585	-33.729	-28.124	-18.441
Ser	-35.204	36.554	-17.641	10.041	-22.146	2.649	-10.595	-30.092	-23.567	-19.419
Thr	-38.724	43.038	-19.680	11.776	-25.454	3.105	-8.586	-30.936	-23.780	-19.031
Trp	-27.930	62.676	-24.145	18.018	-78.553	7.935	23.408	-47.210	-41.096	-25.410
Tyr	-29.361	55.629	-21.501	15.194	-64.973	6.473	14.614	-43.885	-37.446	-24.257
Val	-21.303	43.142	-13.797	9.904	-48.168	4.741	14.378	-29.049	-24.228	-14.608

Table S9: A comparison of the SSMF3 fragmentation scheme with the unfragmented results for supermolecular and SAPT0 interaction energies for amino acid + *wc*-BCX complex. HF, MP2, and SCS-MP2 interaction energies, as well as SAPT0 components and total SAPT0 interaction energies, are presented. Energies are in millihartree

	No Fragmentation									
	$E^{(10)}_{elst}$	$E^{(10)}_{exch}$	$E^{(20)}_{ind,resp}$	$E^{(20)}_{exch-ind,resp}$	$E^{(20)}_{disp}$	$E^{(20)}_{exch-disp}$	E^{HF}_{int}	E^{SAPT0}_{tot}	E^{MP2}_{int}	$E^{SCS-MP2}_{int}$
Ala	-64.693	72.210	-34.610	21.052	-40.690	5.695	-15.325	-50.320	-40.169	-32.257
Asn	-86.934	97.753	-48.553	27.913	-47.173	7.022	-23.414	-63.565	-51.515	-42.321
AspH	-72.604	70.311	-34.120	18.460	-34.170	4.645	-28.997	-58.522	-43.795	-37.423
Cys	-70.799	76.206	-35.870	21.443	-39.934	5.711	-19.652	-53.874	-44.407	-36.492
Gln	-82.064	87.852	-45.266	24.889	-38.243	5.807	-29.619	-62.054	-47.296	-40.303
GluH	-85.440	85.689	-44.687	23.400	-34.197	5.210	-36.984	-65.972	-50.539	-44.242
Gly	-67.930	69.237	-33.708	19.673	-32.280	4.981	-22.651	-49.950	-41.512	-35.136
HisD	-78.865	92.964	-47.186	28.429	-46.290	6.909	-19.003	-58.383	-50.072	-41.014
HisE	-67.324	78.367	-38.435	24.900	-52.790	7.155	-12.038	-57.672	-50.531	-40.139
Ile	-65.411	71.415	-32.966	19.420	-39.691	5.359	-17.292	-51.624	-42.641	-34.780
Leu	-69.450	76.437	-35.987	21.582	-41.823	5.839	-17.989	-53.973	-44.555	-36.306
Lys	-94.394	105.385	-54.154	30.838	-45.567	7.166	-30.948	-69.349	-56.302	-47.637
Met	-64.087	72.754	-31.908	20.331	-50.058	6.330	-10.998	-54.725	-43.833	-34.109
Phe	-61.198	73.861	-33.098	22.796	-58.474	7.463	-5.302	-56.313	-47.259	-36.141
Pro	-68.570	76.716	-37.043	22.598	-44.742	6.113	-16.471	-55.101	-44.604	-35.919
Ser	-75.705	78.611	-37.556	22.327	-39.875	5.876	-23.159	-57.157	-48.307	-40.416
Thr	-78.588	83.978	-40.231	23.937	-44.171	6.397	-22.347	-60.122	-48.207	-39.726
Trp	-69.280	80.560	-36.989	24.122	-56.704	7.345	-11.323	-60.681	-51.890	-40.642
Tyr	-55.553	60.607	-27.835	18.099	-46.605	5.562	-11.668	-52.711	-42.365	-33.288
Val	-64.865	70.582	-32.735	19.289	-38.610	5.265	-17.375	-50.721	-41.854	-34.216
	SSMF3									
	$E^{(10)}_{elst}$	$E^{(10)}_{exch}$	$E^{(20)}_{ind,resp}$	$E^{(20)}_{exch-ind,resp}$	$E^{(20)}_{disp}$	$E^{(20)}_{exch-disp}$	E^{HF}_{int}	E^{SAPT0}_{tot}	E^{MP2}_{int}	$E^{SCS-MP2}_{int}$
Ala	-65.387	72.148	-34.652	20.953	-39.758	5.488	-16.281	-50.552	-40.497	-32.703
Asn	-87.122	97.740	-48.279	27.439	-46.404	6.808	-23.963	-63.560	-51.479	-42.374
AspH	-72.970	70.207	-34.373	18.471	-33.666	4.515	-29.412	-58.563	-43.768	-37.461
Cys	-70.920	76.153	-35.528	21.103	-39.233	5.519	-19.891	-53.605	-44.107	-36.285
Gln	-82.186	87.822	-45.193	24.892	-37.672	5.667	-29.221	-61.226	-46.416	-39.495
GluH	-85.017	85.324	-44.165	23.207	-33.583	5.026	-35.970	-64.526	-49.079	-42.851
Gly	-68.076	69.297	-33.349	19.392	-31.700	4.811	-22.834	-49.723	-41.274	-34.976
HisD	-79.090	92.966	-47.041	28.198	-45.723	6.771	-19.135	-58.087	-49.898	-40.893
HisE	-67.621	78.427	-38.224	24.669	-52.009	6.999	-12.362	-57.371	-50.263	-39.983
Ile	-65.546	71.359	-32.676	19.122	-39.071	5.198	-17.544	-51.417	-42.446	-34.661
Leu	-69.542	76.421	-35.618	21.257	-41.172	5.663	-18.124	-53.632	-44.228	-36.062
Lys	-94.540	105.272	-53.942	30.685	-44.825	6.960	-30.763	-68.629	-55.647	-47.050
Met	-64.828	72.655	-31.977	20.200	-48.992	6.109	-12.080	-54.962	-44.097	-34.522
Phe	-61.735	73.875	-33.258	22.775	-57.503	7.283	-6.021	-56.241	-47.225	-36.257
Pro	-69.188	76.577	-36.942	22.424	-43.814	5.917	-17.450	-55.348	-45.013	-36.441
Ser	-76.013	78.654	-37.389	22.036	-39.138	5.679	-23.689	-57.148	-48.309	-40.511
Thr	-79.185	83.825	-40.145	23.749	-43.251	6.174	-23.262	-60.339	-48.528	-40.161
Trp	-69.340	80.491	-36.855	23.880	-55.970	7.195	-11.610	-60.385	-51.660	-40.508
Tyr	-56.093	60.554	-27.857	18.044	-45.813	5.410	-12.226	-52.629	-42.303	-33.340
Val	-65.002	70.522	-32.432	18.988	-37.991	5.104	-17.613	-50.500	-41.651	-34.088

Table S10: The SAPT0 energy components, SAPT0 and HF interaction energies, MP2 and SCS-MP2 electron-correlated parts of the interaction energies (in mH) for BCX + amino acid complexes. The percent errors of the SSMF3 fragmentation scheme are given in parenthesis.

	$E^{(10)}_{elst}$	$E^{(10)}_{exch}$	$E^{(20)}_{ind,resp}$	$E^{(20)}_{exch-ind,resp}$	$E^{(20)}_{disp}$	$E^{(20)}_{exch-disp}$	E^{HF}_{int}	E^{SAPT0}_{tot}	$E^{MP2}_{corr,int}$	$E^{SCS-MP2}_{corr,int}$
al-BCX- Ala	-72.6 (0.7)	94.8 (0.5)	-43.1 (0.4)	27.8 (-0.9)	-58.9 (-2.4)	8.1 (-3.8)	-5.9 (11.0)	-56.8 (-0.8)	-41.2 (-2.4)	-29.8 (-2.8)
al-BCX- Asn	-107.1 (0.5)	133.2 (0.7)	-64.8 (0.6)	39.5 (0.2)	-79.1 (-2.2)	11.1 (-4.0)	-18.2 (0.7)	-86.2 (-1.3)	-51.4 (-2.6)	-35.8 (-3.2)
al-BCX- AspH	-76.6 (1.4)	91.1 (0.2)	-42.3 (1.8)	26.2 (-0.4)	-71.9 (-2.8)	8.8 (-6.0)	-13.1 (12.6)	-76.1 (0.2)	-48.1 (-2.6)	-34.1 (-3.1)
al-BCX- Cys	-76.0 (-0.1)	108.2 (0.6)	-52.0 (-0.3)	33.0 (-1.4)	-67.6 (-2.5)	9.1 (-3.5)	-3.0 (-27.1)	-61.6 (-3.6)	-48.8 (-3.1)	-35.5 (-3.4)
al-BCX- Gln	-72.6 (1.2)	88.6 (0.7)	-39.9 (2.3)	23.7 (1.3)	-71.2 (-2.6)	8.8 (-5.2)	-11.2 (11.7)	-73.6 (-0.1)	-47.9 (-2.8)	-34.0 (-3.5)
al-BCX- GluH	-66.5 (0.8)	81.0 (0.4)	-34.1 (1.1)	21.0 (0.4)	-64.3 (-2.5)	7.5 (-4.7)	-8.0 (5.5)	-64.7 (-1.3)	-42.3 (-3.3)	-29.8 (-3.9)
al-BCX- Gly	-68.5 (-0.3)	90.8 (0.9)	-42.5 (0.4)	25.9 (0.1)	-57.9 (-2.6)	7.9 (-5.2)	-7.7 (-20.7)	-57.7 (-4.7)	-41.2 (-3.2)	-29.6 (-3.4)
al-BCX- HisD	-70.8 (0.9)	94.0 (0.4)	-39.5 (1.8)	27.2 (-0.1)	-77.3 (-2.5)	9.6 (-4.9)	1.2 (-92.4)	-66.6 (-0.6)	-54.5 (-2.9)	-39.6 (-3.3)
al-BCX- HisE	-82.8 (0.7)	105.6 (0.5)	-52.1 (0.9)	32.3 (-0.3)	-73.2 (-2.3)	9.8 (-3.5)	-13.2 (1.0)	-76.7 (-1.6)	-51.3 (-2.3)	-37.0 (-2.7)
al-BCX- Ile	-59.0 (1.3)	88.2 (0.4)	-36.6 (2.7)	23.8 (-0.4)	-74.1 (-2.4)	9.1 (-4.4)	6.8 (-24.7)	-58.3 (0.5)	-53.7 (-1.9)	-39.3 (-2.1)
al-BCX- Leu	-84.0 (0.0)	125.4 (0.6)	-56.0 (0.6)	36.7 (-0.2)	-81.4 (-2.0)	11.1 (-3.8)	5.6 (3.8)	-64.7 (-2.2)	-59.5 (-1.7)	-43.8 (-1.9)
al-BCX- Lys	-70.0 (0.4)	111.0 (0.7)	-52.5 (0.9)	31.6 (-0.8)	-75.3 (-2.5)	9.8 (-4.1)	3.2 (15.5)	-62.3 (-3.2)	-55.5 (-2.4)	-41.2 (-2.6)
al-BCX- Met	-69.1 (0.5)	107.4 (0.6)	-47.5 (1.5)	31.7 (0.2)	-76.5 (-2.2)	10.0 (-3.3)	8.7 (-7.3)	-57.9 (-1.3)	-57.1 (-2.2)	-42.1 (-2.4)
al-BCX- Phe	-56.6 (0.4)	85.0 (0.5)	-34.1 (-0.3)	22.9 (-0.8)	-69.8 (-1.6)	8.1 (-3.1)	6.7 (5.2)	-55.0 (-2.2)	-54.1 (-1.8)	-40.1 (-1.9)
al-BCX- Pro	-58.0 (1.7)	76.7 (0.4)	-34.2 (2.5)	20.9 (0.3)	-64.2 (-2.6)	7.8 (-4.5)	-3.5 (50.8)	-59.9 (0.8)	-45.7 (-1.9)	-33.1 (-2.3)
al-BCX- Ser	-97.6 (0.1)	128.8 (0.9)	-62.5 (1.0)	38.4 (0.6)	-73.3 (-2.2)	10.7 (-4.2)	-12.0 (-3.4)	-74.6 (-2.1)	-50.1 (-2.1)	-35.5 (-2.4)
al-BCX- Thr	-60.5 (0.4)	79.8 (0.5)	-29.5 (1.2)	20.2 (0.0)	-67.0 (-2.7)	8.1 (-5.2)	2.5 (-20.8)	-56.4 (-1.6)	-49.2 (-2.8)	-36.2 (-3.2)
al-BCX- Trp	-67.8 (0.7)	97.9 (0.4)	-44.7 (1.2)	28.7 (-1.0)	-77.9 (-1.9)	9.2 (-3.0)	0.5 (-92.9)	-68.2 (-1.1)	-58.6 (-2.2)	-43.4 (-2.5)
al-BCX- Tyr	-79.9 (-0.4)	122.7 (0.5)	-56.1 (0.4)	37.0 (-0.5)	-85.8 (-1.6)	11.1 (-2.9)	7.7 (10.4)	-67.0 (-2.7)	-64.2 (-1.7)	-47.5 (-1.8)
al-BCX- Val	-58.3 (1.4)	86.1 (0.4)	-36.1 (2.6)	23.4 (-0.4)	-71.5 (-2.4)	8.8 (-4.4)	5.5 (-30.0)	-57.2 (0.5)	-51.6 (-1.9)	-37.7 (-2.2)
pc-BCX- Ala	-24.4 (0.9)	40.9 (0.6)	-16.6 (3.3)	11.8 (1.1)	-44.7 (-2.2)	4.9 (-3.4)	8.2 (-6.8)	-31.7 (-0.8)	-34.1 (-2.0)	-25.3 (-2.2)
pc-BCX- Asn	-69.3 (-0.1)	73.0 (0.3)	-34.6 (2.6)	19.7 (0.6)	-35.4 (-1.5)	4.9 (-1.3)	-21.8 (1.5)	-52.2 (-0.2)	-17.2 (-3.0)	-10.5 (-4.2)
pc-BCX- AspH	-38.0 (0.8)	55.9 (0.8)	-22.1 (2.4)	14.7 (1.2)	-54.1 (-2.2)	5.7 (-3.5)	4.9 (-4.7)	-43.5 (-1.7)	-39.3 (-2.7)	-28.5 (-3.1)
pc-BCX- Cys	-38.0 (0.4)	46.6 (0.0)	-20.4 (6.3)	13.7 (0.7)	-32.9 (-1.9)	4.0 (-2.5)	-3.2 (41.9)	-32.1 (2.6)	-22.1 (-1.6)	-15.9 (-1.9)
pc-BCX- Gln	-27.3 (1.3)	48.0 (0.7)	-17.3 (2.8)	12.9 (0.6)	-55.4 (-2.1)	5.7 (-3.4)	12.0 (-3.0)	-37.6 (-1.6)	-42.2 (-2.7)	-31.3 (-3.0)
pc-BCX- GluH	-31.0 (0.7)	50.4 (0.5)	-21.3 (2.4)	14.5 (0.6)	-56.5 (-1.9)	5.8 (-3.0)	7.4 (-4.5)	-43.3 (-1.4)	-42.5 (-2.6)	-31.1 (-2.8)
pc-BCX- Gly	-53.1 (-0.4)	59.6 (0.0)	-27.6 (3.0)	16.9 (0.0)	-28.8 (-2.1)	4.3 (-2.7)	-12.0 (2.3)	-36.4 (-0.6)	-17.4 (-1.3)	-11.9 (-1.6)
pc-BCX- HisD	-30.4 (0.2)	44.2 (0.2)	-19.0 (3.5)	12.4 (0.6)	-46.7 (-1.6)	4.9 (-2.1)	3.1 (-14.1)	-38.7 (-0.6)	-34.7 (-2.1)	-25.6 (-2.3)
pc-BCX- HisE	-48.7 (0.1)	60.0 (-0.1)	-27.6 (3.3)	17.7 (0.6)	-48.9 (-1.8)	5.8 (-2.4)	-5.7 (16.0)	-48.8 (0.4)	-34.3 (-1.3)	-24.8 (-1.5)
pc-BCX- Ile	-17.9 (1.1)	37.9 (0.6)	-11.7 (2.6)	8.6 (0.6)	-47.9 (-2.5)	4.5 (-3.7)	13.7 (-1.3)	-29.7 (-2.8)	-39.2 (-2.8)	-29.6 (-2.9)
pc-BCX- Leu	-23.0 (0.2)	44.7 (0.5)	-14.7 (2.2)	11.3 (0.3)	-53.5 (-1.7)	5.5 (-2.7)	14.7 (-0.9)	-33.3 (-1.9)	-42.5 (-1.9)	-31.9 (-2.1)
pc-BCX- Lys	-28.2 (2.3)	54.0 (0.8)	-18.6 (2.1)	13.8 (0.8)	-59.9 (-1.7)	6.4 (-2.6)	16.7 (-2.6)	-36.8 (-1.2)	-46.6 (-2.1)	-35.0 (-2.2)
pc-BCX- Met	-27.5 (0.5)	49.3 (0.5)	-19.2 (2.2)	14.4 (0.4)	-57.2 (-2.1)	6.1 (-3.2)	12.3 (-1.9)	-38.8 (-1.9)	-44.7 (-2.4)	-33.3 (-2.6)
pc-BCX- Phe	-26.7 (0.2)	51.5 (0.4)	-19.8 (1.7)	16.1 (1.0)	-60.2 (-2.0)	6.5 (-2.6)	16.6 (-0.9)	-37.1 (-2.5)	-48.4 (-2.2)	-36.5 (-2.4)
pc-BCX- Pro	-23.3 (-0.2)	41.0 (0.5)	-14.9 (2.6)	10.7 (0.4)	-49.0 (-2.1)	4.8 (-3.3)	9.7 (-1.4)	-34.4 (-2.1)	-38.6 (-2.4)	-28.8 (-2.6)
pc-BCX- Ser	-34.9 (0.9)	36.6 (0.0)	-16.4 (7.9)	10.0 (0.7)	-22.6 (-1.8)	2.7 (-2.3)	-8.9 (19.1)	-28.7 (4.7)	-13.1 (-0.7)	-8.9 (-0.9)
pc-BCX- Thr	-38.6 (0.4)	43.0 (0.0)	-18.4 (7.0)	11.7 (0.9)	-26.0 (-1.9)	3.2 (-2.7)	-7.2 (19.2)	-30.0 (3.2)	-15.4 (-1.4)	-10.6 (-1.9)
pc-BCX- Trp	-27.8 (0.6)	62.4 (0.4)	-23.3 (3.6)	17.9 (0.6)	-80.1 (-1.9)	8.1 (-2.5)	24.2 (-3.4)	-47.7 (-1.0)	-65.7 (-1.8)	-49.8 (-2.0)
pc-BCX- Tyr	-29.4 (-0.1)	55.4 (0.4)	-21.2 (1.6)	15.1 (0.6)	-66.3 (-2.0)	6.7 (-3.1)	14.6 (-0.1)	-45.0 (-2.4)	-53.3 (-2.3)	-39.8 (-2.4)
pc-BCX- Val	-21.1 (1.1)	42.9 (0.7)	-13.4 (2.8)	9.9 (0.5)	-49.3 (-2.3)	4.9 (-3.8)	14.6 (-1.5)	-29.8 (-2.5)	-39.7 (-2.7)	-29.8 (-2.8)
wc-BCX- Ala	-64.7 (1.1)	72.2 (-0.1)	-34.6 (0.1)	21.1 (-0.5)	-40.7 (-2.3)	5.7 (-3.7)	-15.3 (6.2)	-50.3 (0.5)	-40.2 (0.8)	-32.3 (1.4)
wc-BCX- Asn	-86.9 (0.2)	97.8 (0.0)	-48.6 (-0.6)	27.9 (-1.7)	-47.2 (-1.6)	7.0 (-3.0)	-23.4 (2.3)	-63.6 (0.0)	-51.5 (-0.1)	-42.3 (0.1)
wc-BCX- AspH	-72.6 (0.5)	70.3 (-0.2)	-34.1 (0.7)	18.5 (0.1)	-34.2 (-1.5)	4.6 (-2.8)	-29.0 (1.4)	-58.5 (0.1)	-43.8 (-0.1)	-37.4 (0.1)
wc-BCX- Cys	-70.8 (0.2)	76.2 (-0.1)	-35.9 (-1.0)	21.4 (-1.6)	-39.9 (-1.8)	5.7 (-3.4)	-19.7 (1.2)	-53.9 (-0.5)	-44.4 (-0.7)	-36.5 (-0.6)
wc-BCX- Gln	-82.1 (0.2)	87.9 (0.0)	-45.3 (-0.2)	24.9 (0.0)	-38.2 (-1.5)	5.8 (-2.4)	-29.6 (-1.3)	-62.1 (-1.3)	-47.3 (-1.9)	-40.3 (-2.0)
wc-BCX- GluH	-85.4 (-0.5)	85.7 (-0.4)	-44.7 (-1.2)	23.4 (-0.8)	-34.2 (-1.8)	5.2 (-3.5)	-37.0 (-2.7)	-66.0 (-2.2)	-50.5 (-2.9)	-44.2 (-3.2)
wc-BCX- Gly	-67.9 (0.2)	69.2 (0.1)	-33.7 (-1.1)	19.7 (-1.4)	-32.3 (-1.8)	5.0 (-3.4)	-22.7 (0.8)	-50.0 (-0.5)	-41.5 (-0.6)	-35.1 (-0.5)
wc-BCX- HisD	-78.9 (0.3)	93.0 (0.0)	-47.2 (-0.3)	28.4 (-0.8)	-46.3 (-1.2)	6.9 (-2.0)	-19.0 (0.7)	-58.4 (-0.5)	-50.1 (-0.4)	-41.0 (-0.3)
wc-BCX- HisE	-67.3 (0.4)	78.4 (0.1)	-38.4 (-0.6)	24.9 (-0.9)	-52.8 (-1.5)	7.2 (-2.2)	-12.0 (2.7)	-57.7 (-0.5)	-50.5 (-0.5)	-40.1 (-0.4)
wc-BCX- Ile	-65.4 (0.2)	71.4 (-0.1)	-33.0 (-0.9)	19.4 (-1.5)	-39.7 (-1.6)	5.4 (-3.0)	-17.3 (1.5)	-51.6 (-0.4)	-42.6 (-0.5)	-34.8 (-0.3)
wc-BCX- Leu	-69.5 (0.1)	76.4 (0.0)	-36.0 (-1.0)	21.6 (-1.5)	-41.8 (-1.6)	5.8 (-3.0)	-18.0 (0.8)	-54.0 (-0.6)	-44.6 (-0.7)	-36.3 (-0.7)
wc-BCX- Lys	-94.4 (0.2)	105.4 (-0.1)	-54.2 (-0.4)	30.8 (-0.5)	-45.6 (-1.6)	7.2 (-2.9)	-30.9 (-0.6)	-69.3 (-1.0)	-56.3 (-1.2)	-47.6 (-1.2)
wc-BCX- Met	-64.1 (1.2)	72.8 (-0.1)	-31.9 (0.2)	20.3 (-0.7)	-50.1 (-2.1)	6.3 (-3.5)	-11.0 (9.8)	-54.7 (0.4)	-43.8 (0.6)	-34.1 (1.2)
wc-BCX- Phe	-61.2 (0.9)	73.9 (0.0)	-33.1 (0.5)	22.8 (-1.0)	-58.5 (-1.7)	7.5 (-2.4)	-5.3 (13.6)	-56.3 (-0.1)	-47.3 (-0.1)	-36.1 (0.3)
wc-BCX- Pro	-68.6 (0.9)	76.7 (-0.2)	-37.0 (-0.3)	22.6 (-0.8)	-44.7 (-2.1)	6.1 (-3.2)	-16.5 (5.9)	-55.1 (0.5)	-44.6 (0.9)	-35.9 (1.5)
wc-BCX- Ser	-75.7 (0.4)	78.6 (0.1)	-37.6 (-0.5)	22.3 (-1.3)	-39.9 (-1.9)	5.9 (-3.4)	-23.2 (2.3)	-57.2 (0.0)	-48.3 (0.0)	-40.4 (0.2)
wc-BCX- Thr	-78.6 (0.8)	84.0 (-0.2)	-40.2 (-0.2)	23.9 (-0.8)	-44.2 (-2.1)	6.4 (-3.5)	-22.3 (4.1)	-60.1 (0.4)	-48.2 (0.7)	-39.7 (1.1)
wc-BCX- Trp	-69.3 (0.1)	80.6 (-0.1)	-37.0 (-0.4)	24.1 (-1.0)	-56.7 (-1.3)	7.3 (-2.1)	-11.3 (2.5)	-60.7 (-0.5)	-51.9 (-0.4)	-40.6 (-0.3)
wc-BCX- Tyr	-55.6 (1.0)	60.6 (-0.1)	-27.8 (0.1)	18.1 (-0.3)	-46.6 (-1.7)	5.6 (-2.7)	-11.7 (4.8)	-52.7 (-0.2)	-42.4 (-0.2)	-33.3 (0.2)
wc-BCX- Val	-64.9 (0.2)	70.6 (-0.1)	-32.7 (-0.9)	19.3 (-1.6)	-38.6 (-1.6)	5.3 (-3.0)	-17.4 (1.4)	-50.7 (-0.4)	-41.9 (-0.5)	-34.2 (-0.4)

Table S11: The electronic and total energy (in Hartree) of both empty calixarenes in three considered conformations, and complexes of these conformations with amino acids.

	CX electronic energy			CX total energy			BCX electronic energy			BCX total energy		
amino acid	*al*-CX	*pc*-CX	*wc*-CX	*al*-CX	*pc*-CX	*wc*-CX	*al*-BCX	*pc*-BCX	*wc*-BCX	*al*-BCX	*pc*-BCX	*wc*-BCX
-	-2072.8532	-2072.8663	-2072.8505	-2072.1857	-2072.1977	-2072.1829	-3016.0544	-3016.0651	-3016.0634	-3014.7261	-3014.7358	-3014.7334
Ala	-2396.5666	-2396.5669	-2396.5693	-2395.7908	-2395.7914	-2395.7928	-3339.7753	-3339.7752	-3339.7788	-3338.3383	-3338.3384	-3338.3399
Asn	-2565.2577	-2565.2416	-2565.2420	-2564.4554	-2564.4392	-2564.4379	-3508.4557	-3508.4430	-3508.4575	-3506.9910	-3506.9790	-3506.9910
AspH	-2585.1187	-2585.1146	-2585.1101	-2584.3281	-2584.3238	-2584.3186	-3528.3220	-3528.3148	-3528.3259	-3526.8692	-3526.8617	-3526.8727
Cys	-2794.7879	-2794.7832	-2794.7819	-2794.0125	-2794.0082	-2794.0062	-3737.9999	-3737.9886	-3737.9996	-3736.5626	-3736.5526	-3736.5608
Gln	-2604.5426	-2604.5410	-2604.5408	-2603.7110	-2603.7109	-2603.7090	-3547.7579	-3547.7553	-3547.7621	-3546.2648	-3546.2612	-3546.2676
GluH	-2624.4149	-2624.4138	-2624.4138	-2623.5960	-2623.5945	-2623.5943	-3567.6221	-3567.6218	-3567.6321	-3566.1410	-3566.1408	-3566.1500
Gly	-2357.2739	-2357.2652	-2357.2670	-2356.5261	-2356.5178	-2356.5181	-3300.4725	-3300.4631	-3300.4801	-3299.0628	-3299.0541	-3299.0689
HisD	-2621.5231	-2621.5227	-2621.5251	-2620.6960	-2620.6952	-2620.6968	-3564.7284	-3564.7216	-3564.7335	-3563.2393	-3563.2339	-3563.2438
HisE	-2621.5275	-2621.5241	-2621.5228	-2620.7007	-2620.6966	-2620.6952	-3564.7297	-3564.7301	-3564.7349	-3563.2425	-3563.2420	-3563.2450
Ile	-2514.4667	-2514.4696	-2514.4679	-2513.6067	-2513.6103	-2513.6078	-3457.6725	-3457.6734	-3457.6813	-3456.1514	-3456.1521	-3456.1586
Leu	-2514.4702	-2514.4710	-2514.4573	-2513.6113	-2513.6116	-2513.5981	-3457.6718	-3457.6750	-3457.6816	-3456.1515	-3456.1533	-3456.1594
Lys	-2569.8116	-2569.8106	-2569.8100	-2568.9337	-2568.9312	-2568.9315	-3513.0229	-3513.0119	-3513.0245	-3511.4839	-3511.4707	-3511.4840
Met	-2873.3880	-2873.3822	-2873.3832	-2872.5551	-2872.5501	-2872.5505	-3816.5911	-3816.5924	-3816.5935	-3815.0976	-3815.0980	-3815.0979
Phe	-2627.5284	-2627.5303	-2627.5217	-2626.6721	-2626.6746	-2626.6658	-3570.7325	-3570.7340	-3570.7341	-3569.2155	-3569.2165	-3569.2162
Pro	-2473.9498	-2473.9519	-2473.9424	-2473.1390	-2473.1397	-2473.1303	-3417.1539	-3417.1584	-3417.1546	-3415.6809	-3415.6851	-3415.6802
Ser	-2471.7847	-2471.7728	-2471.7753	-2471.0025	-2470.9927	-2470.9927	-3414.9875	-3414.9762	-3414.9896	-3413.5450	-3413.5346	-3413.5451
Thr	-2511.0888	-2511.0812	-2511.0776	-2510.2795	-2510.2736	-2510.2684	-3454.2891	-3454.2825	-3454.2935	-3452.8185	-3452.8134	-3452.8216
Trp	-2759.0526	-2759.0527	-2759.0490	-2758.1683	-2758.1691	-2758.1646	-3702.2522	-3702.2577	-3702.2659	-3700.7076	-3700.7117	-3700.7184
Tyr	-2702.7420	-2702.7425	-2702.7447	-2701.8820	-2701.8827	-2701.8839	-3645.9501	-3645.9587	-3645.9530	-3644.4294	-3644.4364	-3644.4311
Val	-2475.1645	-2475.1724	-2475.1640	-2474.3336	-2474.3409	-2474.3321	-3418.3751	-3418.3771	-3418.3825	-3416.8824	-3416.8840	-3416.8880

Table S12: Percent errors of the SSMF3 fragmentation scheme in respect to unfragmented results for amino acid + *al*-CX complex. HF, electron-correlated part of MP2, and SCS-MP2 interaction energies, as well as SAPT0 components and total SAPT0 interaction energies, are presented. Energies are in millihartree.

	No Fragmentation									
	$E^{(10)}_{elst}$	$E^{(10)}_{exch}$	$E^{(20)}_{ind,resp}$	$E^{(20)}_{exch-ind,resp}$	$E^{(20)}_{disp}$	$E^{(20)}_{exch-disp}$	E^{HF}_{int}	E^{SAPT0}_{tot}	$E^{MP2}_{corr,int}$	$E^{SCS-MP2}_{corr,int}$
Ala	-66.511	97.946	-46.244	29.354	-56.574	8.258	-0.086	-48.402	-40.727	-29.551
Asn	-86.888	110.433	-58.943	35.023	-57.001	8.461	-20.297	-68.837	-37.901	-26.816
AspH	-67.476	94.486	-46.685	29.850	-55.251	7.647	-3.743	-51.346	-40.064	-29.220
Cys	-84.532	104.660	-51.560	31.424	-58.791	8.604	-15.811	-65.998	-38.390	-26.874
Gln	-102.619	128.131	-58.977	36.001	-68.946	10.127	-16.477	-75.296	-44.391	-30.807
GluH	-59.400	66.858	-30.384	18.749	-53.972	6.523	-11.791	-59.240	-34.899	-24.336
Gly	-74.734	91.281	-46.798	28.239	-50.562	7.491	-16.605	-59.676	-32.565	-22.670
HisD	-57.099	67.446	-30.630	18.306	-54.576	6.384	-10.228	-58.420	-38.355	-27.373
HisE	-67.587	96.522	-49.049	30.577	-63.662	8.701	-5.170	-60.130	-45.940	-33.812
Ile	-56.079	76.037	-32.572	21.456	-61.625	7.591	0.430	-53.604	-43.552	-31.421
Leu	-58.328	83.653	-39.000	25.015	-57.618	7.485	-0.308	-50.441	-43.076	-31.938
Lys	-75.732	102.650	-48.311	29.825	-62.988	8.689	-7.944	-62.242	-44.735	-32.550
Met	-58.322	74.616	-33.669	21.212	-62.184	7.571	-4.888	-59.501	-43.708	-31.501
Phe	-52.761	68.503	-29.500	18.249	-53.481	6.393	-4.526	-51.614	-36.450	-26.021
Pro	-85.332	115.454	-59.798	36.191	-58.267	8.771	-13.833	-63.330	-39.544	-28.187
Ser	-61.679	74.400	-31.141	19.750	-53.122	6.839	-6.247	-52.531	-37.622	-26.983
Thr	-62.370	72.339	-29.794	18.937	-57.117	7.069	-8.288	-58.336	-40.953	-29.510
Trp	-53.373	79.108	-34.654	24.261	-67.661	8.347	5.740	-53.574	-53.026	-39.628
Tyr	-60.824	73.332	-31.702	20.102	-60.059	7.360	-6.874	-59.573	-43.366	-31.718
Val	-60.088	78.762	-36.571	22.854	-57.711	7.250	-5.408	-55.869	-42.277	-30.991
	Percent error of SSMF3									
	$E^{(10)}_{elst}$	$E^{(10)}_{exch}$	$E^{(20)}_{ind,resp}$	$E^{(20)}_{exch-ind,resp}$	$E^{(20)}_{disp}$	$E^{(20)}_{exch-disp}$	E^{HF}_{int}	E^{SAPT0}_{tot}	$E^{MP2}_{corr,int}$	$E^{SCS-MP2}_{corr,int}$
Ala	-0.32	0.61	1.39	0.72	-1.39	-2.92	-423.90	-1.88	-0.98	-1.06
Asn	0.38	0.11	-1.09	-1.36	-0.88	-1.31	-3.80	-1.69	-1.41	-1.68
AspH	-0.28	0.32	-1.04	-1.88	-1.12	-1.40	-24.66	-2.79	-1.38	-1.51
Cys	0.37	0.12	-0.35	-1.09	-1.46	-2.68	-0.77	-1.14	-1.77	-2.17
Gln	0.00	0.57	-0.31	-0.53	-0.93	-1.38	-4.80	-1.72	-1.29	-1.59
GluH	1.25	0.13	0.51	-0.01	-1.78	-3.54	7.08	0.18	-2.05	-2.65
Gly	0.51	0.16	-0.40	-1.20	-1.55	-2.71	-1.21	-1.31	-1.36	-1.63
HisD	1.72	0.21	2.69	0.32	-1.41	-3.15	18.68	2.29	-1.07	-1.41
HisE	-0.10	0.37	-1.35	-0.85	-0.84	-0.81	-26.73	-3.07	-1.28	-1.38
Ile	-0.28	0.10	0.86	-0.59	-1.52	-3.32	-73.79	-0.69	-1.53	-1.86
Leu	-0.06	0.30	-1.09	-1.74	-1.21	-2.14	-267.77	-2.70	-1.46	-1.60
Lys	0.22	0.44	-0.55	-1.51	-0.92	-2.02	-11.27	-2.09	-1.15	-1.30
Met	1.88	0.14	1.50	-0.42	-1.74	-3.53	38.44	1.79	-1.13	-1.43
Phe	-0.23	-0.06	-0.38	-0.37	-1.00	-2.00	-11.22	-1.77	-1.46	-1.74
Pro	-0.12	0.30	-1.49	-1.10	-1.15	-1.73	-11.91	-3.42	-1.19	-1.36
Ser	-0.42	0.38	1.64	0.76	-1.80	-4.27	3.13	-0.89	-1.44	-1.68
Thr	-0.16	0.17	1.23	0.23	-1.67	-3.62	3.75	-0.66	-1.43	-1.66
Trp	-0.26	0.31	-0.38	-0.69	-0.65	-1.37	5.41	-1.19	-0.87	-0.98
Tyr	-0.09	0.40	1.94	0.82	-1.56	-3.47	7.67	-0.26	-1.47	-1.75
Val	-0.77	0.24	-0.20	-1.66	-1.24	-2.94	-11.18	-1.98	-1.75	-1.96

Table S13: Percent errors of the SSMF3 fragmentation scheme in respect to unfragmented results for amino acid + *pc*-CX complex. HF, electron-correlated part of MP2, and SCS-MP2 interaction energies, as well as SAPT0 components and total SAPT0 interaction energies, are presented. Energies are in millihartree.

	No Fragmentation									
	$E^{(10)}_{elst}$	$E^{(10)}_{exch}$	$E^{(20)}_{ind,resp}$	$E^{(20)}_{exch-ind,resp}$	$E^{(20)}_{disp}$	$E^{(20)}_{exch-disp}$	E^{HF}_{int}	E^{SAPT0}_{tot}	$E^{MP2}_{corr,int}$	$E^{SCS-MP2}_{corr,int}$
Ala	-55.113	62.693	-29.486	18.193	-30.112	4.566	-12.025	-37.571	-17.987	-12.317
Asn	-34.136	37.688	-14.354	10.096	-31.154	3.398	-3.350	-31.106	-20.272	-14.425
AspH	-26.622	32.757	-13.509	8.736	-26.938	2.751	-1.450	-25.637	-18.350	-13.225
Cys	-14.631	19.574	-7.016	4.967	-19.711	1.774	1.371	-16.566	-14.435	-10.536
Gln	-50.095	57.311	-26.055	16.187	-35.378	4.457	-9.701	-40.622	-21.051	-14.355
GluH	-41.790	45.630	-20.656	13.288	-29.835	3.583	-8.460	-34.712	-17.805	-12.302
Gly	-33.437	35.977	-15.939	9.697	-20.125	2.612	-7.984	-25.497	-11.386	-7.659
HisD	-39.837	52.540	-22.550	16.312	-47.012	5.367	2.014	-39.630	-34.133	-25.078
HisE	-24.949	36.153	-15.155	11.786	-39.352	4.267	4.986	-30.099	-30.287	-22.593
Ile	-22.192	39.788	-14.672	11.352	-41.197	4.351	11.115	-25.731	-32.075	-24.058
Leu	-30.955	43.766	-16.135	11.803	-38.547	4.127	4.893	-29.527	-28.114	-20.703
Lys	-19.312	38.923	-12.384	9.442	-43.176	4.534	13.446	-25.196	-35.416	-26.701
Met	-17.447	25.627	-8.626	5.954	-26.880	2.348	3.517	-21.015	-20.126	-14.779
Phe	-39.893	56.243	-24.854	18.446	-49.130	6.037	4.626	-38.467	-36.419	-27.115
Pro	-20.118	34.235	-12.811	9.561	-38.956	4.087	7.711	-27.158	-30.777	-22.907
Ser	-30.005	34.680	-14.420	9.586	-24.822	2.761	-3.506	-25.566	-16.184	-11.503
Thr	-37.853	39.620	-17.701	10.500	-22.471	2.811	-10.427	-30.087	-12.121	-7.979
Trp	-35.839	54.524	-22.220	16.868	-55.662	6.201	8.864	-40.596	-42.728	-32.088
Tyr	-17.303	27.443	-10.792	7.041	-31.439	3.132	3.969	-24.338	-24.297	-17.888
Val	-23.078	37.511	-12.952	9.634	-39.479	4.192	8.003	-27.284	-30.447	-22.574
	Percent error of SSMF3									
	$E^{(10)}_{elst}$	$E^{(10)}_{exch}$	$E^{(20)}_{ind,resp}$	$E^{(20)}_{exch-ind,resp}$	$E^{(20)}_{disp}$	$E^{(20)}_{exch-disp}$	E^{HF}_{int}	E^{SAPT0}_{tot}	$E^{MP2}_{corr,int}$	$E^{SCS-MP2}_{corr,int}$
Ala	-0.19	0.08	2.89	-0.05	-1.42	-1.94	2.84	0.01	-1.36	-1.76
Asn	1.18	-0.01	13.32	2.10	-2.01	-2.65	65.49	5.33	-2.05	-2.62
AspH	2.12	0.43	13.89	2.75	-1.44	-1.84	147.35	7.01	-1.61	-2.06
Cys	2.70	0.35	14.82	1.89	-1.14	-1.05	-95.46	6.65	-1.07	-1.28
Gln	0.17	0.34	6.52	1.34	-1.50	-1.83	15.83	2.67	-1.25	-1.68
GluH	0.63	0.16	9.72	2.02	-1.61	-2.06	25.19	4.97	-1.96	-2.59
Gly	0.81	0.00	6.82	0.93	-1.40	-1.88	15.30	3.88	-1.32	-1.90
HisD	0.58	0.11	6.97	1.21	-1.05	-1.14	-84.75	3.22	-0.89	-1.08
HisE	0.24	0.32	6.98	1.56	-0.63	-0.09	-18.89	2.31	-0.66	-0.74
Ile	2.36	0.77	4.08	1.20	-1.33	-1.75	-6.79	1.10	-1.62	-1.82
Leu	1.32	0.27	10.36	1.73	-1.03	-1.36	-40.45	5.54	-0.82	-1.04
Lys	0.29	0.21	3.20	0.50	-0.99	-1.39	-3.48	0.42	-0.90	-1.03
Met	2.05	0.49	17.65	2.21	-0.89	-0.22	-49.23	7.13	-1.61	-1.93
Phe	0.91	-0.02	5.82	0.89	-1.02	-1.66	-36.21	3.31	-0.78	-0.97
Pro	0.02	0.21	3.36	0.68	-0.88	-0.85	-4.93	0.26	-1.11	-1.25
Ser	1.43	0.29	11.54	2.03	-1.46	-1.82	57.03	6.60	-1.30	-1.74
Thr	0.71	-0.29	4.34	0.16	-1.30	-1.74	11.04	3.02	-1.36	-1.98
Trp	0.97	0.02	7.70	1.12	-0.92	-1.07	-22.89	3.90	-0.68	-0.83
Tyr	1.85	0.13	4.57	1.23	-0.78	-0.66	-18.42	2.09	-0.46	-0.54
Val	0.65	0.48	4.15	1.35	-1.12	-1.31	-5.77	0.28	-1.10	-1.25

Table S14: Percent errors of the SSMF3 fragmentation scheme in respect to unfragmented results for amino acid + *wc*-CX complex. HF, electron-correlated part of MP2, and SCS-MP2 interaction energies, as well as SAPT0 components and total SAPT0 interaction energies, are presented. Energies are in millihartree.

	No Fragmentation									
	$E^{(10)}_{elst}$	$E^{(10)}_{exch}$	$E^{(20)}_{ind,resp}$	$E^{(20)}_{exch-ind,resp}$	$E^{(20)}_{disp}$	$E^{(20)}_{exch-disp}$	E^{HF}_{int}	E^{SAPT0}_{tot}	$E^{MP2}_{corr,int}$	$E^{SCS-MP2}_{corr,int}$
Ala	-72.884	80.455	-39.285	23.007	-36.439	5.665	-20.904	-51.678	-21.250	-14.151
Asn	-92.079	108.997	-55.302	31.454	-45.361	7.106	-23.809	-62.064	-25.633	-16.900
AspH	-49.717	48.131	-22.429	12.494	-30.021	3.581	-17.615	-44.056	-16.662	-10.797
Cys	-54.894	57.958	-27.841	15.979	-30.850	4.102	-17.669	-44.417	-16.670	-10.783
Gln	-83.647	85.179	-41.127	22.207	-39.288	5.699	-31.393	-64.981	-18.148	-10.838
GluH	-71.491	73.171	-37.095	19.877	-34.024	4.827	-28.005	-57.202	-15.965	-9.573
Gly	-74.211	80.043	-39.474	23.001	-34.674	5.579	-22.909	-52.005	-19.788	-12.999
HisD	-89.046	104.420	-52.597	31.528	-52.916	7.882	-22.038	-67.072	-34.922	-24.624
HisE	-71.048	83.268	-40.817	25.906	-51.162	7.140	-13.673	-57.695	-36.330	-26.355
Ile	-68.096	75.550	-34.973	20.515	-39.448	5.492	-17.677	-51.634	-24.554	-16.790
Leu	-73.804	86.407	-42.484	25.242	-43.586	6.392	-17.017	-54.212	-26.261	-17.673
Lys	-92.131	105.094	-55.859	32.243	-44.792	7.135	-29.271	-66.928	-24.665	-16.132
Met	-77.540	90.093	-43.903	25.611	-42.446	6.311	-19.694	-55.829	-25.725	-17.434
Phe	-78.849	88.198	-43.554	25.254	-41.302	6.227	-22.354	-57.429	-24.420	-16.377
Pro	-70.665	81.086	-39.778	24.680	-46.706	6.577	-15.367	-55.496	-29.042	-19.996
Ser	-81.076	84.059	-41.457	23.296	-39.381	5.887	-27.341	-60.835	-22.016	-14.208
Thr	-77.149	85.870	-40.021	23.701	-43.481	6.282	-19.305	-56.504	-25.822	-17.136
Trp	-79.851	93.771	-43.320	27.378	-60.352	7.973	-13.928	-66.307	-40.777	-28.924
Tyr	-72.223	83.761	-40.223	26.709	-53.663	7.462	-12.652	-58.853	-37.068	-26.583
Val	-71.502	78.800	-37.333	21.794	-40.313	5.676	-19.363	-54.000	-24.650	-16.736
	Percent error of SSMF3									
	$E^{(10)}_{elst}$	$E^{(10)}_{exch}$	$E^{(20)}_{ind,resp}$	$E^{(20)}_{exch-ind,resp}$	$E^{(20)}_{disp}$	$E^{(20)}_{exch-disp}$	E^{HF}_{int}	E^{SAPT0}_{tot}	$E^{MP2}_{corr,int}$	$E^{SCS-MP2}_{corr,int}$
Ala	-0.06	0.09	-1.28	-1.94	-1.25	-3.02	-0.43	-0.72	-1.31	-1.65
Asn	-0.13	0.17	-0.64	-1.44	-0.99	-2.46	-0.96	-0.81	-1.32	-1.76
AspH	0.56	-0.30	-0.82	-1.17	-1.17	-2.32	1.58	0.02	-2.04	-2.77
Cys	-0.29	-0.30	-0.18	-0.67	-1.33	-3.17	-1.42	-1.19	-1.99	-2.58
Gln	-0.19	-0.36	0.11	-0.99	-1.33	-2.87	0.71	-0.21	-2.04	-2.89
GluH	0.20	-0.37	-0.93	-0.87	-1.34	-3.07	-0.69	-0.88	-2.57	-3.71
Gly	0.01	0.22	-1.23	-1.79	-1.32	-3.30	-0.39	-0.70	-1.51	-1.93
HisD	0.19	0.07	-0.49	-1.22	-1.04	-2.42	0.04	-0.52	-1.11	-1.34
HisE	0.06	0.08	-0.59	-1.25	-0.96	-1.96	1.40	-0.28	-0.74	-0.84
Ile	0.01	-0.03	-0.99	-1.84	-1.11	-2.68	0.69	-0.33	-1.20	-1.51
Leu	0.13	-0.09	-0.20	-1.10	-1.16	-2.15	2.09	-0.02	-1.35	-1.68
Lys	0.20	-0.31	-0.57	-0.92	-1.36	-2.93	0.15	-0.53	-2.14	-2.84
Met	-0.13	0.14	-0.76	-1.86	-1.14	-2.85	-0.13	-0.59	-1.12	-1.41
Phe	0.13	0.09	-1.19	-2.06	-1.24	-3.09	0.45	-0.38	-1.35	-1.72
Pro	0.90	-0.11	-0.07	-0.62	-1.60	-3.11	6.10	0.71	-1.62	-1.96
Ser	-0.02	-0.10	-0.45	-1.88	-0.96	-2.79	1.37	0.27	-1.28	-1.73
Thr	0.31	-0.03	-0.20	-1.89	-1.31	-3.01	3.77	0.62	-1.69	-2.23
Trp	0.21	-0.10	0.13	-1.56	-1.09	-2.64	5.79	0.54	-1.26	-1.56
Tyr	0.08	-0.06	-0.72	-1.30	-0.75	-1.65	1.50	-0.15	-0.66	-0.78
Val	0.17	-0.08	-1.14	-2.03	-1.14	-2.80	1.15	-0.14	-1.28	-1.64

Table S15: Percent errors of the SSMF3 fragmentation scheme in respect to unfragmented results for amino acid + *al*-BCX complex. HF, electron-correlated part of MP2, and SCS-MP2 interaction energies, as well as SAPT0 components and total SAPT0 interaction energies, are presented. Energies are in millihartree.

	No Fragmentation									
	$E^{(10)}_{elst}$	$E^{(10)}_{exch}$	$E^{(20)}_{ind,resp}$	$E^{(20)}_{exch-ind,resp}$	$E^{(20)}_{disp}$	$E^{(20)}_{exch-disp}$	E^{HF}_{int}	E^{SAPT0}_{tot}	$E^{MP2}_{corr,int}$	$E^{SCS-MP2}_{corr,int}$
Ala	-72.586	94.819	-43.129	27.796	-58.940	8.075	-5.907	-56.772	-41.228	-29.779
Asn	-107.118	133.174	-64.800	39.473	-79.087	11.095	-18.234	-86.225	-51.372	-35.824
AspH	-76.579	91.098	-42.327	26.185	-71.923	8.849	-13.069	-76.143	-48.087	-34.066
Cys	-75.995	108.237	-52.038	32.984	-67.607	9.069	-3.042	-61.580	-48.808	-35.474
Gln	-72.572	88.558	-39.919	23.697	-71.163	8.753	-11.207	-73.617	-47.879	-34.037
GluH	-66.459	81.030	-34.100	20.985	-64.294	7.545	-7.976	-64.725	-42.341	-29.812
Gly	-68.549	90.820	-42.527	25.907	-57.926	7.899	-7.678	-57.705	-41.208	-29.601
HisD	-70.793	94.017	-39.496	27.157	-77.301	9.562	1.155	-66.584	-54.544	-39.558
HisE	-82.761	105.599	-52.117	32.282	-73.195	9.757	-13.248	-76.686	-51.279	-36.999
Ile	-58.990	88.174	-36.551	23.839	-74.143	9.050	6.787	-58.306	-53.726	-39.310
Leu	-84.040	125.391	-56.033	36.736	-81.377	11.080	5.623	-64.674	-59.509	-43.778
Lys	-70.017	110.985	-52.510	31.601	-75.269	9.778	3.209	-62.282	-55.511	-41.222
Met	-69.110	107.446	-47.500	31.731	-76.489	9.963	8.667	-57.859	-57.080	-42.074
Phe	-56.578	85.046	-34.136	22.896	-69.848	8.117	6.749	-54.982	-54.066	-40.079
Pro	-58.031	76.715	-34.178	20.935	-64.162	7.811	-3.498	-59.850	-45.711	-33.078
Ser	-97.562	128.790	-62.524	38.360	-73.285	10.684	-11.970	-74.572	-50.080	-35.484
Thr	-60.507	79.828	-29.472	20.161	-67.001	8.132	2.455	-56.414	-49.236	-36.241
Trp	-67.796	97.888	-44.665	28.653	-77.904	9.178	0.542	-68.184	-58.586	-43.377
Tyr	-79.945	122.719	-56.056	37.031	-85.777	11.132	7.688	-66.956	-64.241	-47.490
Val	-58.273	86.089	-36.087	23.372	-71.503	8.766	5.524	-57.213	-51.573	-37.681
	Percent error of SSMF3									
	$E^{(10)}_{elst}$	$E^{(10)}_{exch}$	$E^{(20)}_{ind,resp}$	$E^{(20)}_{exch-ind,resp}$	$E^{(20)}_{disp}$	$E^{(20)}_{exch-disp}$	E^{HF}_{int}	E^{SAPT0}_{tot}	$E^{MP2}_{corr,int}$	$E^{SCS-MP2}_{corr,int}$
Ala	0.68	0.51	0.41	-0.86	-2.39	-3.77	10.95	-0.81	-2.39	-2.79
Asn	0.54	0.74	0.60	0.15	-2.16	-4.00	0.70	-1.32	-2.63	-3.17
AspH	1.39	0.21	1.80	-0.35	-2.79	-5.97	12.63	0.23	-2.59	-3.06
Cys	-0.06	0.60	-0.31	-1.37	-2.52	-3.49	-27.13	-3.60	-3.06	-3.38
Gln	1.17	0.65	2.29	1.26	-2.59	-5.23	11.70	-0.10	-2.82	-3.45
GluH	0.84	0.41	1.06	0.44	-2.54	-4.68	5.53	-1.30	-3.26	-3.91
Gly	-0.30	0.90	0.44	0.05	-2.60	-5.17	-20.67	-4.65	-3.15	-3.42
HisD	0.92	0.37	1.78	-0.05	-2.53	-4.92	-92.44	-0.62	-2.86	-3.28
HisE	0.71	0.54	0.88	-0.31	-2.29	-3.46	0.98	-1.57	-2.34	-2.68
Ile	1.26	0.36	2.70	-0.43	-2.38	-4.35	-24.65	0.52	-1.92	-2.12
Leu	0.00	0.59	0.60	-0.22	-1.97	-3.81	3.82	-2.16	-1.69	-1.88
Lys	0.36	0.73	0.87	-0.81	-2.48	-4.13	15.54	-3.15	-2.39	-2.62
Met	0.51	0.61	1.47	0.24	-2.22	-3.33	-7.32	-1.27	-2.15	-2.40
Phe	0.42	0.47	-0.34	-0.79	-1.61	-3.09	5.21	-2.22	-1.80	-1.94
Pro	1.67	0.43	2.47	0.28	-2.61	-4.50	50.80	0.76	-1.92	-2.25
Ser	0.05	0.91	0.97	0.62	-2.24	-4.23	-3.41	-2.14	-2.12	-2.44
Thr	0.41	0.45	1.23	0.04	-2.71	-5.15	-20.81	-1.57	-2.82	-3.17
Trp	0.68	0.39	1.22	-0.96	-1.92	-3.04	-92.89	-1.05	-2.21	-2.47
Tyr	-0.40	0.54	0.40	-0.50	-1.56	-2.86	10.36	-2.71	-1.72	-1.83
Val	1.39	0.38	2.61	-0.44	-2.43	-4.43	-30.03	0.54	-1.94	-2.15

Table S16: Percent errors of the SSMF3 fragmentation scheme in respect to unfragmented results for amino acid + *pc*-BCX complex. HF, electron-correlated part of MP2, and SCS-MP2 interaction energies, as well as SAPT0 components and total SAPT0 interaction energies, are presented. Energies are in millihartree.

	No Fragmentation									
	$E^{(10)}_{elst}$	$E^{(10)}_{exch}$	$E^{(20)}_{ind,resp}$	$E^{(20)}_{exch-ind,resp}$	$E^{(20)}_{disp}$	$E^{(20)}_{exch-disp}$	E^{HF}_{int}	E^{SAPT0}_{tot}	$E^{MP2}_{corr,int}$	$E^{SCS-MP2}_{corr,int}$
Ala	-24.358	40.912	-16.633	11.803	-44.745	4.860	8.209	-31.677	-34.146	-25.290
Asn	-69.279	73.027	-34.606	19.689	-35.362	4.874	-21.753	-52.241	-17.162	-10.524
AspH	-38.017	55.904	-22.077	14.719	-54.104	5.721	4.901	-43.482	-39.263	-28.516
Cys	-38.020	46.603	-20.419	13.692	-32.926	3.980	-3.198	-32.143	-22.101	-15.877
Gln	-27.349	48.027	-17.341	12.883	-55.350	5.739	12.014	-37.596	-42.233	-31.275
GluH	-31.013	50.424	-21.297	14.472	-56.548	5.835	7.393	-43.320	-42.480	-31.063
Gly	-53.093	59.566	-27.594	16.864	-28.760	4.335	-11.974	-36.400	-17.381	-11.928
HisD	-30.363	44.213	-18.976	12.395	-46.748	4.906	3.124	-38.718	-34.676	-25.606
HisE	-48.730	60.037	-27.576	17.678	-48.906	5.776	-5.666	-48.796	-34.335	-24.820
Ile	-17.854	37.891	-11.720	8.565	-47.900	4.471	13.718	-29.712	-39.193	-29.638
Leu	-22.958	44.680	-14.697	11.313	-53.452	5.451	14.683	-33.318	-42.508	-31.932
Lys	-28.166	53.969	-18.615	13.818	-59.919	6.392	16.743	-36.783	-46.643	-34.978
Met	-27.478	49.251	-19.163	14.409	-57.212	6.100	12.310	-38.802	-44.732	-33.258
Phe	-26.719	51.509	-19.796	16.138	-60.179	6.506	16.621	-37.052	-48.398	-36.461
Pro	-23.322	41.016	-14.883	10.652	-49.012	4.841	9.722	-34.448	-38.628	-28.769
Ser	-34.898	36.563	-16.355	9.975	-22.562	2.711	-8.895	-28.746	-13.057	-8.905
Thr	-38.583	43.039	-18.386	11.672	-25.957	3.190	-7.201	-29.968	-15.415	-10.646
Trp	-27.770	62.443	-23.302	17.913	-80.071	8.141	24.237	-47.693	-65.711	-49.795
Tyr	-29.375	55.410	-21.160	15.098	-66.290	6.680	14.635	-44.976	-53.283	-39.842
Val	-21.081	42.860	-13.424	9.851	-49.319	4.927	14.589	-29.803	-39.659	-29.834
	Percent error of SSMF3									
	$E^{(10)}_{elst}$	$E^{(10)}_{exch}$	$E^{(20)}_{ind,resp}$	$E^{(20)}_{exch-ind,resp}$	$E^{(20)}_{disp}$	$E^{(20)}_{exch-disp}$	E^{HF}_{int}	E^{SAPT0}_{tot}	$E^{MP2}_{corr,int}$	$E^{SCS-MP2}_{corr,int}$
Ala	0.87	0.60	3.31	1.12	-2.22	-3.36	-6.84	-0.84	-2.02	-2.18
Asn	-0.08	0.32	2.59	0.59	-1.48	-1.33	1.53	-0.24	-2.99	-4.19
AspH	0.84	0.78	2.39	1.15	-2.19	-3.47	-4.66	-1.74	-2.74	-3.05
Cys	0.43	-0.01	6.29	0.70	-1.88	-2.51	41.87	2.55	-1.58	-1.91
Gln	1.27	0.70	2.81	0.62	-2.07	-3.43	-2.96	-1.57	-2.73	-3.01
GluH	0.68	0.51	2.43	0.55	-1.93	-3.01	-4.50	-1.35	-2.56	-2.84
Gly	-0.42	0.02	3.01	-0.03	-2.12	-2.70	2.26	-0.61	-1.26	-1.56
HisD	0.22	0.16	3.48	0.57	-1.62	-2.08	-14.13	-0.55	-2.11	-2.34
HisE	0.14	-0.09	3.33	0.61	-1.76	-2.41	15.98	0.38	-1.29	-1.48
Ile	1.14	0.56	2.59	0.64	-2.48	-3.73	-1.32	-2.82	-2.75	-2.93
Leu	0.16	0.49	2.24	0.33	-1.73	-2.71	-0.94	-1.91	-1.92	-2.05
Lys	2.25	0.81	2.13	0.75	-1.71	-2.59	-2.62	-1.15	-2.06	-2.21
Met	0.49	0.54	2.16	0.39	-2.05	-3.17	-1.87	-1.92	-2.40	-2.60
Phe	0.16	0.38	1.73	0.98	-2.04	-2.63	-0.90	-2.45	-2.21	-2.36
Pro	-0.17	0.50	2.56	0.43	-2.07	-3.25	-1.41	-2.09	-2.38	-2.58
Ser	0.88	-0.03	7.86	0.66	-1.84	-2.30	19.11	4.68	-0.65	-0.90
Thr	0.37	0.00	7.04	0.89	-1.93	-2.67	19.23	3.23	-1.43	-1.89
Trp	0.57	0.37	3.62	0.59	-1.90	-2.53	-3.42	-1.01	-1.84	-1.96
Tyr	-0.05	0.39	1.61	0.63	-1.99	-3.09	-0.14	-2.43	-2.29	-2.44
Val	1.06	0.66	2.78	0.54	-2.33	-3.77	-1.45	-2.53	-2.66	-2.84

Table S17: Percent errors of the SSMF3 fragmentation scheme in respect to unfragmented results for amino acid + *wc*-BCX complex. HF, electron-correlated part of MP2, and SCS-MP2 interaction energies, as well as SAPT0 components and total SAPT0 interaction energies, are presented. Energies are in millihartree.

No Fragmentation										
	$E^{(10)}_{elst}$	$E^{(10)}_{exch}$	$E^{(20)}_{ind,resp}$	$E^{(20)}_{exch-ind,resp}$	$E^{(20)}_{disp}$	$E^{(20)}_{exch-disp}$	E^{HF}_{int}	E^{SAPT0}_{tot}	$E^{MP2}_{corr,int}$	$E^{SCS-MP2}_{corr,int}$
Ala	-64.693	72.210	-34.610	21.052	-40.690	5.695	-15.325	-50.320	-24.844	-16.932
Asn	-86.934	97.753	-48.553	27.913	-47.173	7.022	-23.414	-63.565	-28.101	-18.907
AspH	-72.604	70.311	-34.120	18.460	-34.170	4.645	-28.997	-58.522	-14.798	-8.426
Cys	-70.799	76.206	-35.870	21.443	-39.934	5.711	-19.652	-53.874	-24.756	-16.841
Gln	-82.064	87.852	-45.266	24.889	-38.243	5.807	-29.619	-62.054	-17.677	-10.684
GluH	-85.440	85.689	-44.687	23.400	-34.197	5.210	-36.984	-65.972	-13.555	-7.258
Gly	-67.930	69.237	-33.708	19.673	-32.280	4.981	-22.651	-49.950	-18.860	-12.485
HisD	-78.865	92.964	-47.186	28.429	-46.290	6.909	-19.003	-58.383	-31.070	-22.011
HisE	-67.324	78.367	-38.435	24.900	-52.790	7.155	-12.038	-57.672	-38.493	-28.101
Ile	-65.411	71.415	-32.966	19.420	-39.691	5.359	-17.292	-51.624	-25.349	-17.489
Leu	-69.450	76.437	-35.987	21.582	-41.823	5.839	-17.989	-53.973	-26.566	-18.317
Lys	-94.394	105.385	-54.154	30.838	-45.567	7.166	-30.948	-69.349	-25.355	-16.690
Met	-64.087	72.754	-31.908	20.331	-50.058	6.330	-10.998	-54.725	-32.835	-23.111
Phe	-61.198	73.861	-33.098	22.796	-58.474	7.463	-5.302	-56.313	-41.957	-30.839
Pro	-68.570	76.716	-37.043	22.598	-44.742	6.113	-16.471	-55.101	-28.133	-19.448
Ser	-75.705	78.611	-37.556	22.327	-39.875	5.876	-23.159	-57.157	-25.148	-17.257
Thr	-78.588	83.978	-40.231	23.937	-44.171	6.397	-22.347	-60.122	-25.860	-17.378
Trp	-69.280	80.560	-36.989	24.122	-56.704	7.345	-11.323	-60.681	-40.567	-29.319
Tyr	-55.553	60.607	-27.835	18.099	-46.605	5.562	-11.668	-52.711	-30.697	-21.621
Val	-64.865	70.582	-32.735	19.289	-38.610	5.265	-17.375	-50.721	-24.479	-16.841
Percent error of SSMF3										
	$E^{(10)}_{elst}$	$E^{(10)}_{exch}$	$E^{(20)}_{ind,resp}$	$E^{(20)}_{exch-ind,resp}$	$E^{(20)}_{disp}$	$E^{(20)}_{exch-disp}$	E^{HF}_{int}	E^{SAPT0}_{tot}	$E^{MP2}_{corr,int}$	$E^{SCS-MP2}_{corr,int}$
Ala	1.07	-0.09	0.12	-0.47	-2.29	-3.65	6.24	0.46	-2.53	-3.01
Asn	0.22	-0.01	-0.56	-1.70	-1.63	-3.04	2.34	-0.01	-2.08	-2.62
AspH	0.50	-0.15	0.74	0.06	-1.47	-2.78	1.43	0.07	-2.99	-4.48
Cys	0.17	-0.07	-0.95	-1.59	-1.76	-3.38	1.22	-0.50	-2.18	-2.65
Gln	0.15	-0.03	-0.16	0.01	-1.49	-2.42	-1.34	-1.33	-2.73	-3.84
GluH	-0.49	-0.43	-1.17	-0.82	-1.80	-3.53	-2.74	-2.19	-3.29	-5.20
Gly	0.21	0.09	-1.06	-1.43	-1.80	-3.41	0.81	-0.46	-2.23	-2.75
HisD	0.29	0.00	-0.31	-0.81	-1.23	-2.00	0.70	-0.51	-0.99	-1.15
HisE	0.44	0.08	-0.55	-0.93	-1.48	-2.18	2.69	-0.52	-1.54	-1.71
Ile	0.21	-0.08	-0.88	-1.53	-1.56	-3.02	1.46	-0.40	-1.77	-2.13
Leu	0.13	-0.02	-1.02	-1.51	-1.56	-3.00	0.75	-0.63	-1.74	-2.07
Lys	0.15	-0.11	-0.39	-0.49	-1.63	-2.87	-0.60	-1.04	-1.86	-2.41
Met	1.16	-0.14	0.22	-0.65	-2.13	-3.49	9.84	0.43	-2.49	-2.89
Phe	0.88	0.02	0.48	-0.09	-1.66	-2.41	13.56	-0.13	-1.79	-1.96
Pro	0.90	-0.18	-0.27	-0.77	-2.07	-3.21	5.94	0.45	-2.03	-2.35
Ser	0.41	0.05	-0.45	-1.30	-1.85	-3.36	2.29	-0.02	-2.10	-2.52
Thr	0.76	-0.18	-0.21	-0.79	-2.08	-3.48	4.09	0.36	-2.29	-2.76
Trp	0.09	-0.09	-0.36	-1.00	-1.29	-2.05	2.53	-0.49	-1.27	-1.44
Tyr	0.97	-0.09	0.08	-0.30	-1.70	-2.74	4.78	-0.16	-2.02	-2.34
Val	0.21	-0.09	-0.93	-1.56	-1.60	-3.04	1.37	-0.44	-1.81	-2.17

Table S18: A complete list of frequencies and intensities of *al*-CX

$\nu(cm^{-1})$	$\epsilon(M^{-1}cm^{-1})$	$\nu(cm^{-1})$	$\epsilon(M^{-1}cm^{-1})$	$\nu(cm^{-1})$	$\epsilon(M^{-1}cm^{-1})$	$\nu(cm^{-1})$	$\epsilon(M^{-1}cm^{-1})$	$\nu(cm^{-1})$	$\epsilon(M^{-1}cm^{-1})$
14.5463	26.3285	495.0850	39.2852	838.4876	10.4882	1214.6275	14.7104	1584.6166	11.4907
19.4371	12.4585	504.4324	25.4952	842.7385	54.1391	1216.5265	83.0606	1586.3525	8.5934
23.5183	3.8506	508.3115	13.1248	865.9595	0.8928	1224.6861	106.3227	1589.5766	44.2600
34.6594	27.5902	508.9294	41.4085	873.2158	6.8886	1230.8213	211.5466	1591.5016	41.6406
41.0821	37.4642	514.0823	17.0151	875.5811	6.9821	1234.8176	52.7541	1594.6810	6.8541
43.5153	24.4505	521.3989	23.5011	882.8863	9.6811	1242.6016	103.3617	1597.8091	10.5574
51.2342	5.1703	528.5987	38.3613	883.8686	11.2099	1247.7705	60.4783	1601.7505	5.5723
62.4299	18.8767	533.7761	4.7743	886.1587	1.8629	1250.4130	66.7901	1602.5017	2.6157
66.8794	62.7226	536.5084	6.9503	892.3477	0.3438	1252.1043	4.5377	1606.6441	2.1364
70.6396	23.4486	538.2889	27.7337	896.0461	2.6037	1252.9513	169.1940	1608.2052	1.7930
72.0310	15.7846	540.0741	8.9084	903.6718	2.4236	1266.7450	93.8969	2912.1040	42.1475
96.9760	97.1391	542.7642	6.2087	905.5891	8.7102	1268.2323	188.9794	2958.2446	11.9764
103.5459	86.7878	546.3105	3.3416	908.6835	5.9331	1274.6362	45.8495	2965.9103	22.3386
118.5987	71.7830	548.6449	6.7667	909.9360	4.4430	1277.8097	45.5199	2987.0847	30.4129
122.7609	43.2864	551.2258	6.6504	913.1299	13.4012	1295.9261	13.5204	2987.8030	33.6778
146.1541	10.8774	556.8053	80.5123	917.3583	14.2014	1296.8085	4.2745	2990.0243	43.9976
159.4686	61.7165	561.5293	122.7086	919.3250	10.3887	1300.8195	20.1614	2990.3342	33.0081
163.8398	27.6365	565.9997	11.7039	922.1677	26.1089	1303.1870	17.1819	2991.7894	13.5956
164.8909	1.3694	578.4437	48.0540	927.1253	12.8762	1307.7012	46.0902	3028.9042	8.7719
185.7668	11.6224	585.6320	80.4805	931.9306	1.6913	1308.6646	47.8808	3029.4035	7.5235
195.0398	137.0048	596.5045	33.4144	943.5201	10.4974	1324.3586	32.1129	3031.3899	9.6735
201.8931	23.8898	604.8870	7.0405	947.0725	24.9316	1328.6221	12.8718	3033.4853	9.3681
213.0776	10.1814	628.7004	324.6391	952.9274	1.8203	1337.1851	9.2409	3096.3181	2.2934
224.9516	28.5897	637.5997	59.9987	955.6633	9.0603	1346.9546	20.8732	3099.0086	8.3629
233.6431	93.5679	643.9591	123.2517	975.9579	27.8538	1355.8670	20.5704	3100.1511	1.2481
234.8060	43.3182	655.0031	259.8322	976.9202	5.0437	1359.1059	45.4709	3101.4736	0.0915
254.7852	132.1197	666.6047	122.1611	1072.6855	1.7100	1381.5867	80.7563	3101.8794	28.0707
257.4359	98.1528	678.2506	5.3337	1073.3354	4.5193	1383.0184	151.4814	3103.4374	0.5903
260.7157	60.1694	698.3981	2.1592	1084.9090	62.9241	1408.8616	152.2777	3103.9503	12.5984
267.6790	100.0483	701.1396	7.3246	1086.7913	112.6562	1412.3863	181.0674	3107.0482	27.3906
276.3064	45.7839	713.0633	8.4604	1092.8867	131.8437	1438.1726	36.4140	3108.2537	21.2290
289.9355	50.5665	727.3402	11.9851	1094.0161	110.8792	1438.9333	76.7866	3110.1047	21.6103
294.7316	30.5474	729.9265	41.1152	1146.6231	14.0430	1446.5359	240.5945	3112.7176	11.0953
301.5457	45.5622	735.3646	275.5535	1148.2130	7.3189	1447.4414	199.2051	3122.2442	16.8414
306.7919	58.3770	737.9174	176.6311	1153.6866	0.3617	1452.9404	247.3572	3129.7933	18.5913
319.2204	22.2365	741.6821	130.1504	1154.3458	8.8864	1453.8852	93.6040	3130.3334	53.1980
320.6048	90.8800	742.0965	282.6411	1166.9709	4.3081	1458.4286	57.3544	3130.5219	26.6320
337.2118	828.6334	744.1417	248.4192	1168.3351	15.3630	1460.1153	144.5291	3131.7938	38.1206
339.6894	8.3055	758.0776	48.1368	1168.4492	32.8681	1473.1695	10.2830	3132.2477	30.7456
380.6371	498.6659	760.7480	403.4092	1170.8717	41.9696	1473.8767	19.3053	3136.9533	17.3735
390.0101	5.8356	770.4679	43.8490	1171.2025	0.8155	1474.5917	21.3342	3336.6567	875.3730
397.8454	195.5645	773.7010	437.9607	1171.5320	2.6772	1475.0757	45.4030	3364.8904	1559.7284
411.7279	79.3911	784.2829	409.1681	1175.7854	124.1489	1478.2266	9.0989	3395.3292	861.6841
419.3905	80.6735	793.8193	22.3186	1183.9592	28.3911	1478.3149	2.1581	3418.5505	756.1878
456.9773	12.9404	808.9074	0.7482	1190.4374	28.8307	1484.2719	46.8096	3600.5463	401.3957
464.5947	3.7524	817.7799	165.5450	1192.3405	27.8435	1484.9284	5.3495	3708.5748	83.1382
470.1569	37.4437	820.1740	62.0142	1194.2072	65.4141	1492.5480	16.1482		
472.4867	23.6846	824.7983	56.5414	1198.1549	48.4902	1493.3752	28.0828		
479.8210	6.2466	835.5699	42.8809	1199.0537	73.9383	1582.6874	12.3013		
488.9047	13.6082	837.7050	18.2215	1202.6437	113.0405	1583.4606	2.7580		

Table S19: A complete list of frequencies and intensities of *pc*-CX

$\nu(cm^{-1})$	$\epsilon(M^{-1}cm^{-1})$	$\nu(cm^{-1})$	$\epsilon(M^{-1}cm^{-1})$	$\nu(cm^{-1})$	$\epsilon(M^{-1}cm^{-1})$	$\nu(cm^{-1})$	$\epsilon(M^{-1}cm^{-1})$	$\nu(cm^{-1})$	$\epsilon(M^{-1}cm^{-1})$
17.0983	3.1498	515.2522	29.3925	869.2877	27.4824	1226.3365	42.1072	1583.3227	0.0310
18.2819	15.6897	516.8028	31.7074	871.5125	4.0168	1229.0021	3.7359	1586.2851	38.4654
25.8963	18.9331	519.3386	1.6078	875.3824	76.9239	1233.7005	15.0214	1587.8370	38.3215
34.0150	6.5796	519.9187	43.4644	877.8246	6.4879	1240.4864	411.1421	1590.9378	11.4340
37.6063	95.1354	528.3081	55.9149	879.3630	10.4657	1244.9659	345.2372	1599.3679	35.2957
47.6546	16.8267	538.6503	26.8048	883.8123	565.4813	1250.1971	22.7210	1600.9406	16.2794
51.0623	1.7266	538.9389	0.8157	885.1051	15.8462	1251.1026	0.7178	1604.0745	32.5551
56.3187	36.1902	540.1207	8.2281	887.0679	0.4088	1256.2134	39.4486	1604.9200	2.9130
68.5519	12.2210	541.0468	52.0332	896.6969	3.4595	1256.9064	58.6485	1606.4588	1.8886
68.9053	114.0336	541.6618	6.2832	896.9656	0.4661	1259.5390	106.9762	1606.6791	7.5168
81.4116	3.1509	543.8475	29.9956	901.9322	2.0630	1260.6438	0.1259	2982.9728	11.2931
106.3619	3.0194	547.7748	3.1266	906.0118	5.1875	1263.1964	193.6660	2983.3541	28.1937
114.4007	462.4883	557.1559	49.5206	907.7991	0.4663	1295.1039	12.7734	2986.8416	19.6885
125.6847	9.2780	563.5493	98.0131	909.2195	0.0004	1296.9754	0.2135	2986.8603	32.0152
136.6781	4.1944	570.2161	54.7488	910.1339	2.8342	1298.5471	11.0034	2989.1006	26.7163
146.4559	18.4276	572.8583	0.0648	910.7910	14.7366	1299.3108	4.6237	2989.1404	48.7538
172.6796	4.1169	577.4807	61.9065	912.4579	12.6560	1305.6219	0.0144	3025.9235	10.7179
173.7341	311.0821	587.8753	44.9574	912.7383	11.3431	1306.3874	50.6638	3025.9506	11.8107
186.0988	15.0573	605.3577	0.3170	914.6303	4.2165	1319.7332	1.7886	3028.9705	28.6487
187.4336	5.1700	610.3943	26.0006	920.3173	26.5312	1319.8244	3.3821	3028.9946	1.4369
211.8258	60.7301	624.3057	85.1888	949.4313	27.1484	1324.3848	7.0376	3031.5142	29.9109
213.6511	12.6992	646.8227	0.0074	950.4175	0.0034	1334.7984	1.8536	3031.5470	0.7196
223.7128	0.1106	666.8783	2.5945	960.3654	20.2850	1336.7829	14.0544	3098.8718	0.7535
224.6542	3.0970	682.9718	1.6133	961.1869	27.2451	1343.9076	0.3004	3098.9827	3.0118
246.3997	24.8188	692.2602	29.6119	963.3983	1.2742	1380.8959	36.7416	3099.0035	11.4759
261.3534	36.8314	701.9972	27.5554	964.0581	0.5247	1388.7997	28.5000	3099.7899	7.9079
267.9865	0.0060	703.2188	71.2138	1070.6292	8.6098	1395.3806	0.0789	3099.8722	5.7275
273.0141	381.6473	712.8726	932.6712	1074.7541	0.0004	1413.4015	406.4112	3100.6342	1.5727
279.3432	6.0939	719.5273	61.9916	1080.2778	1.2744	1432.6340	428.2235	3101.0821	0.4145
281.2983	271.2083	725.2546	395.8156	1087.1470	202.3356	1444.3424	24.7273	3104.6286	4.3475
285.8903	4.2477	727.6622	0.0751	1092.1637	166.5901	1446.9042	434.9922	3105.0426	50.1476
294.8975	385.0335	731.1252	912.4077	1094.3486	49.8498	1449.7387	475.4623	3106.0538	28.9086
301.4448	21.7306	738.5022	16.7181	1148.0645	10.9723	1450.0748	145.8391	3106.6296	41.4465
310.5220	58.7691	738.7783	24.2573	1149.2864	6.6685	1451.6180	3.0338	3107.5256	0.3158
315.6876	3.0557	741.4260	600.5377	1153.5110	8.4754	1453.3928	1.6736	3107.7451	27.0168
323.2890	53.5261	743.0562	11.7579	1153.7336	7.2109	1454.7735	21.8713	3129.6825	42.7767
380.9201	10.7380	752.4569	141.1209	1158.4897	2.6172	1472.3042	46.2477	3129.7510	6.3485
383.3904	35.8566	759.1319	33.4841	1158.5683	6.0039	1473.1529	2.3192	3130.1705	0.0716
397.3983	30.6916	765.0515	302.5460	1167.7939	3.0271	1474.3018	110.8133	3130.2074	120.5067
423.0057	1.0223	779.4622	6.4637	1168.3099	2.1062	1475.1100	67.2906	3130.5527	134.9528
427.2092	56.2930	786.6031	73.1615	1170.2441	0.6290	1475.7896	62.5454	3130.7758	28.8698
443.4506	30.6143	787.3061	57.4898	1171.4546	0.0153	1480.3512	67.6875	3162.1332	3789.7298
450.6428	5.6976	798.6025	95.3416	1172.6740	1.3268	1481.0559	3.0478	3185.0320	5303.8190
452.5948	13.2764	799.1413	283.1033	1172.7661	0.0007	1483.0414	29.2068	3243.4649	378.8283
461.0610	0.8289	827.1912	139.9304	1193.3021	137.9641	1484.2797	6.4396	3246.0559	261.8728
474.3623	10.0542	828.4536	73.3951	1193.7241	0.1768	1491.3156	0.0599	3269.7592	44.2543
479.4404	12.5954	830.5301	54.2226	1197.6408	120.0256	1501.2474	5.1718		
495.1138	1.8299	833.0692	21.6305	1199.6196	18.6035	1511.9702	41.1212		
504.5708	92.2698	835.3227	14.3114	1202.2146	7.4790	1581.1988	3.6352		
509.1231	68.1105	839.2507	37.4787	1204.4168	34.6706	1581.8070	2.6499		

Table S20: A complete list of frequencies and intensities of *wc*-CX

$\nu(cm^{-1})$	$\epsilon(M^{-1}cm^{-1})$	$\nu(cm^{-1})$	$\epsilon(M^{-1}cm^{-1})$	$\nu(cm^{-1})$	$\epsilon(M^{-1}cm^{-1})$	$\nu(cm^{-1})$	$\epsilon(M^{-1}cm^{-1})$	$\nu(cm^{-1})$	$\epsilon(M^{-1}cm^{-1})$
14.9066	1.3381	492.9495	0.4823	843.2165	2.8250	1219.8718	11.6405	1587.2501	3.0498
25.6640	0.3886	500.7797	3.9593	845.6482	62.0128	1221.0006	121.5560	1587.8722	44.1359
26.7415	8.3543	508.5550	3.0437	865.2531	14.5310	1223.9207	1.1135	1590.4727	43.4570
31.5179	1.5442	509.8424	0.4327	874.3670	6.0149	1224.1343	106.5466	1591.1382	21.1876
34.8494	18.5336	517.2245	45.7841	874.9697	0.6684	1231.4036	184.0664	1595.8231	1.6304
46.0521	50.7293	521.9985	0.5518	878.3363	6.1035	1235.7928	41.8331	1596.7776	7.5439
57.0764	48.3679	532.9705	30.2238	881.8391	0.8614	1238.1617	134.6500	1601.2511	17.3797
57.6698	0.0138	534.5285	0.6650	882.7924	0.8975	1243.0020	127.5921	1602.2852	4.2153
63.6442	23.1926	535.1031	22.8530	893.4491	4.1749	1251.5463	83.7382	1608.5219	2.1711
70.1088	36.2699	536.7086	37.2360	898.2086	1.4155	1251.9105	22.2600	1608.8183	1.6748
73.7532	55.5733	538.9417	1.0992	904.6838	5.9487	1261.8186	250.4671	2930.4758	36.3974
75.0182	39.5918	539.9518	9.3013	907.3462	6.3265	1262.0677	0.1046	2930.5059	60.2948
113.5309	152.2586	542.8011	7.5922	908.0251	4.5512	1274.6629	2.4941	2969.6426	17.0474
129.3125	0.2869	547.8840	0.9852	910.4267	28.5056	1275.1746	45.0296	2969.6937	0.8925
130.2708	99.8982	553.5166	9.2449	912.9140	0.5344	1289.6128	5.2936	2988.1331	56.9035
136.1211	52.6425	559.2379	76.3869	914.1802	11.2968	1290.8316	64.8246	2988.1854	13.4901
155.6992	2.8979	560.7764	21.0185	919.7227	49.7454	1299.7338	49.6892	2991.7749	45.3840
160.1781	20.6047	567.1500	56.4910	924.5165	9.6754	1301.6822	32.8994	2991.8829	37.6897
167.1409	0.2220	573.3030	1.2407	927.4542	6.4517	1308.8477	63.2041	3028.2217	5.9827
185.0824	189.1146	573.5381	18.7256	930.0030	51.5137	1309.3132	22.1284	3028.2632	8.2015
193.0186	38.8154	590.5334	107.1192	941.2018	1.1868	1321.0103	16.7257	3034.3425	7.5936
195.1911	6.1417	613.0111	79.5223	941.4491	8.6801	1322.6178	86.2644	3034.4572	7.2570
207.3827	53.7267	632.8452	216.7346	957.0945	5.6363	1330.7322	4.9780	3096.2607	2.2489
211.6465	0.6220	633.8809	288.5021	957.5103	10.3465	1331.4770	0.8258	3096.2783	1.2544
224.6085	147.8863	638.5236	427.2109	979.3098	5.6359	1357.6236	0.1181	3099.6041	3.0367
235.7842	4.3771	657.0858	63.4049	979.3290	8.7737	1357.6953	105.9308	3099.6280	3.5789
249.0169	7.5089	667.1810	97.1038	1074.8288	2.8739	1365.1378	75.6594	3101.2366	19.9830
252.0302	184.6316	674.8105	56.9219	1075.8853	22.8499	1365.5416	13.5483	3101.2900	40.0663
255.3623	0.1656	693.3712	388.0705	1083.7949	29.0585	1376.5788	81.3480	3104.1255	4.8038
258.8086	110.1009	693.8379	881.5713	1086.4593	60.6499	1376.7495	297.4021	3104.2801	14.0578
281.1621	2.0248	698.0285	6.2890	1093.0751	90.5071	1445.3573	263.4855	3107.6098	20.4828
289.6985	65.6140	700.6672	27.6902	1093.5500	143.1192	1445.7183	93.8878	3107.6639	20.0412
297.9884	61.5154	705.8149	96.8559	1147.7855	71.9046	1445.8446	276.3744	3126.2982	2.4935
310.8462	12.8212	725.3400	18.5973	1148.9203	3.4167	1446.9875	70.6615	3127.3111	16.2169
323.8462	13.9375	737.3383	105.9145	1151.7446	3.4357	1451.2579	72.4578	3130.1109	21.5139
329.0932	41.9338	738.3630	268.6495	1152.6495	3.0606	1451.6093	87.3004	3130.1270	17.2527
343.0132	34.6227	738.9286	157.0965	1161.2543	99.0463	1462.9148	127.3431	3130.4348	85.2151
347.2206	24.7680	741.7947	1.8092	1161.7442	413.0402	1463.3076	0.0714	3130.5079	16.8876
363.8727	1700.9385	741.8982	457.6978	1167.2609	3.6054	1470.9704	93.4831	3144.1620	8.0057
370.0718	0.6694	743.3545	36.1118	1167.7731	5.2214	1471.5576	13.7670	3144.2484	7.4726
384.9315	4.2285	764.2565	79.3812	1170.3820	5.1239	1473.5356	13.0814	3442.6744	3.4892
386.0130	32.9424	765.8958	1.7470	1170.3918	0.3899	1474.9377	56.6570	3447.6801	1906.6723
410.1324	31.1394	782.7748	8.5045	1175.4586	106.0545	1475.6445	0.1765	3492.3215	857.6370
412.1952	32.1149	791.8412	12.5928	1176.0024	18.4387	1477.7003	7.4224	3493.0451	81.7737
454.3133	1.3988	801.0199	11.9754	1186.4498	95.5653	1483.8599	40.1693	3728.6552	47.3387
456.2547	13.8248	805.0055	50.5837	1188.1723	0.9777	1485.8081	13.4975	3728.8177	85.6627
469.2497	23.5615	816.8185	24.3300	1189.6690	0.0439	1488.6056	0.0075		
473.5731	22.0516	817.4008	83.3624	1190.3970	1.7899	1489.5582	51.6692		
486.0069	6.1933	833.8461	22.2878	1197.3886	160.3147	1584.3175	9.8829		
489.2253	6.9379	835.4695	46.7591	1198.2968	65.7681	1584.4377	1.7892		

Table S21: A complete list of frequencies and intensities of *al*-BCX

$\nu(cm^{-1})$	$\epsilon(M^{-1}cm^{-1})$	$\nu(cm^{-1})$	$\epsilon(M^{-1}cm^{-1})$	$\nu(cm^{-1})$	$\epsilon(M^{-1}cm^{-1})$	$\nu(cm^{-1})$	$\epsilon(M^{-1}cm^{-1})$	$\nu(cm^{-1})$	$\epsilon(M^{-1}cm^{-1})$	$\nu(cm^{-1})$	$\epsilon(M^{-1}cm^{-1})$
7.4177	15.3817	351.4051	1.4077	796.8953	53.2166	1190.7587	45.4076	1451.5621	0.1325	2987.3992	35.2968
10.1063	6.9080	351.5802	12.3978	801.9885	245.0515	1192.2215	42.7730	1452.0768	0.0764	2989.2679	46.9365
13.2003	4.1102	352.1368	3.3104	804.4660	41.3908	1192.5215	63.5562	1452.2961	0.2253	2990.1480	33.0666
16.7322	11.6352	352.5801	14.7376	805.1558	30.2011	1192.6016	15.9592	1452.3530	0.5573	2991.3337	13.3953
19.4045	13.5074	352.9250	1.1281	808.7697	21.5893	1193.0007	2.2926	1452.5325	0.2675	3027.2028	9.1862
22.2156	17.1316	357.1510	5.7381	809.8964	60.9215	1193.1149	24.2798	1452.7196	0.4045	3029.1372	6.8488
25.0017	19.2914	360.0038	168.4439	811.8454	37.9635	1193.4624	17.5984	1454.8245	0.1138	3030.1584	9.5900
25.9358	26.2414	369.5441	529.6035	817.5963	272.1649	1193.5206	19.5589	1455.5804	0.3790	3030.7251	8.6805
29.4660	6.1602	374.2090	8.7942	863.5015	44.5416	1193.7368	82.6001	1455.7316	0.1411	3030.9253	9.0753
30.8047	0.6216	374.6240	4.2309	868.0417	44.8110	1194.7287	29.8117	1456.1243	1.3466	3031.1000	6.2307
37.4904	2.5219	375.5089	2.8706	869.7890	86.2235	1195.8433	121.8757	1456.4182	1.6117	3031.5632	6.8613
38.1734	0.7002	375.9608	3.7542	871.2196	12.5838	1196.6971	20.8951	1456.5344	1.4106	3031.7715	15.0462
38.2380	2.3787	377.3868	9.6895	873.1966	59.7453	1198.9117	3.6157	1458.3798	102.6978	3031.8778	7.0295
40.7018	5.7241	378.2468	70.4070	877.4037	12.9039	1199.5242	76.3109	1458.9958	68.4272	3032.5516	5.5474
41.0643	1.1464	392.7032	7.2189	881.2635	15.8021	1200.3808	24.1312	1459.4100	34.4730	3032.5649	11.5958
41.4784	2.1256	396.0179	96.6015	886.0917	17.9482	1202.1771	20.0642	1460.5399	30.5617	3032.6084	22.1437
44.9674	6.9643	411.0096	78.9009	886.3873	2.8589	1202.9727	70.1749	1460.8144	31.0740	3032.9975	8.3024
61.7171	29.2626	415.9913	85.1112	889.2265	16.3887	1208.3900	176.8667	1461.1781	39.9855	3033.1627	15.4934
85.6090	19.6373	427.3054	0.4033	890.9626	1.2685	1211.7322	32.9442	1466.1531	115.4467	3033.9553	10.3778
91.2951	65.7434	428.8308	19.8674	895.9306	26.0346	1223.0063	56.4675	1466.4929	69.5206	3034.3486	13.2221
94.1260	31.8725	431.4171	15.9052	906.7078	1.5131	1229.3817	88.4534	1468.1093	130.1626	3037.5182	43.8827
96.0826	15.6449	433.2186	6.2767	907.3544	1.9464	1235.1618	41.6584	1470.7520	101.8141	3038.3287	43.1977
98.6110	72.7559	435.5264	21.0312	908.4921	2.5100	1236.1529	217.7741	1471.4988	103.9337	3038.3391	41.5708
101.4565	18.1468	436.8569	7.2673	908.7038	3.3330	1248.3019	48.8851	1472.0018	18.1501	3038.9622	34.8532
105.4036	4.2012	437.9147	2.2010	908.8960	3.0391	1253.2339	56.5908	1476.9855	30.6638	3039.4768	21.0354
105.8302	28.5926	439.3314	1.4093	909.4923	15.9182	1255.4017	69.4241	1477.3857	4.5236	3039.6881	155.5559
112.6724	9.2908	440.2274	23.6549	909.8718	14.0749	1256.7546	204.4824	1478.0724	23.0086	3040.3494	87.9779
114.7150	49.5427	442.3742	7.6303	911.2350	7.0206	1265.2040	25.1358	1478.3096	143.2404	3040.5753	70.7051
118.1922	64.6582	445.4358	3.5798	914.3318	5.4003	1268.7506	27.0326	1480.1403	18.8069	3040.8135	161.5572
125.7548	57.8639	448.9361	1.8253	916.5261	1.8647	1269.3372	232.6009	1480.1589	2.3166	3040.9109	25.3453
141.2557	20.1905	473.7019	40.2610	918.9042	4.9549	1273.5311	74.5747	1480.6580	25.0708	3040.9806	184.8625
144.8079	80.4338	477.2724	22.5059	919.3481	1.1538	1277.0859	30.2090	1480.7379	8.8251	3042.0559	184.9010
153.8834	16.5426	481.2492	14.4365	920.8759	3.7495	1277.5394	45.3684	1481.0269	13.8821	3045.9449	20.4940
172.6157	8.4334	488.3378	0.7459	921.6991	6.3055	1280.5267	170.7199	1481.8529	20.7609	3046.2028	23.2179
179.4042	107.6756	503.0728	11.5763	924.6254	2.9477	1286.4957	3.1754	1481.9188	12.7716	3046.2844	26.4796
185.9368	5.3425	506.8621	1.5836	925.6816	3.3193	1287.0823	165.9523	1483.1238	71.8056	3046.5226	21.2558
191.1733	126.2574	507.6389	48.9764	926.8132	1.8535	1288.5169	51.1426	1488.2969	193.4225	3046.8758	17.7400
198.9107	45.7182	513.1560	4.9677	928.6188	0.7273	1293.4671	23.0598	1489.8098	15.8651	3048.0189	22.0664
205.1435	24.2016	523.4653	0.8040	930.6481	1.0044	1294.1414	17.7096	1494.3110	20.9095	3048.5736	41.9857
209.9276	7.4399	525.6883	78.6658	932.7072	4.6814	1298.6573	8.7664	1494.5898	85.3578	3048.6446	58.0337
223.1565	70.9883	525.8110	40.7141	935.0323	12.7016	1301.4678	14.4431	1494.8561	101.6500	3048.8513	88.8511
225.2575	1.2450	530.8128	27.7996	938.2966	32.0318	1301.6518	62.3254	1495.0810	18.0919	3049.5192	72.7479
229.5579	2.3374	540.2322	19.0353	940.6985	36.6353	1309.6952	33.2376	1495.4991	65.8271	3049.6844	35.4506
231.4920	15.4601	546.8050	18.2235	945.6242	24.4171	1313.9871	41.8293	1495.9284	3.0693	3051.2305	63.6686
234.0623	6.4904	551.4606	40.4018	951.0932	0.1938	1320.1221	16.5702	1496.2140	42.4077	3102.0443	24.1389
234.7918	8.0097	555.6905	22.7809	951.6857	1.3066	1323.6739	21.5574	1497.0452	139.4480	3105.1457	11.0615
235.9326	8.9973	561.0176	30.3256	951.9613	1.0531	1327.7242	37.7173	1589.4018	39.7805	3105.9443	18.5433
238.1401	27.3364	573.3866	29.8391	951.9798	1.3569	1339.9891	51.8650	1591.7206	32.6311	3110.6339	30.3152
242.2672	25.6901	578.0426	40.5246	952.2324	0.5480	1354.1343	21.7784	1592.5949	2.4195	3116.6827	7.9886
245.0540	12.7795	582.7882	12.9049	952.5811	0.0666	1359.1294	28.6252	1593.2656	7.3355	3125.8569	8.5927
249.8788	25.0991	588.1027	47.9126	959.6618	9.6012	1368.6323	6.7567	1594.1908	6.4298	3126.2851	15.5263
252.6387	35.8296	591.4034	19.0099	960.9641	30.7203	1368.9816	17.2053	1595.1826	3.2194	3129.3533	7.8521
261.4734	80.1912	593.2891	8.7953	967.8285	27.5247	1369.6077	12.2084	1600.9289	4.8254	3131.3053	14.3365
267.3870	31.5840	595.1460	105.2788	968.4718	32.7041	1369.9423	19.2349	1601.7209	4.6461	3132.8162	9.0545
274.4497	11.9355	600.1475	18.8273	988.3744	23.0692	1370.7215	27.4617	1602.8385	15.2752	3133.2280	16.2093
276.4757	2.3708	610.4106	9.7851	991.4656	43.6825	1370.8266	8.8692	1603.0034	4.6183	3135.0272	4.2946
277.0773	5.4655	617.7632	101.2055	1030.9685	1.8802	1370.9579	14.5401	1603.9084	8.8093	3351.1715	931.7463
277.9995	18.3972	622.9724	11.8272	1031.0854	10.3240	1371.2891	20.5953	1606.3016	7.4932	3378.2373	1624.3517
278.9415	5.5062	628.4111	192.3339	1031.4707	3.0199	1371.5317	15.4703	2914.2303	43.3748	3407.4805	850.2954
279.4915	20.4044	632.5915	11.3882	1031.6172	2.6451	1371.6251	25.0781	2954.8190	13.1871	3431.7536	699.4819
280.4935	11.7964	636.2564	10.2886	1031.9804	3.9006	1372.1186	17.8568	2960.8901	42.5283	3594.4124	428.8844
283.9485	0.8823	641.8765	458.8193	1032.8176	5.5877	1372.4741	18.8111	2961.4194	34.1224	3712.3435	82.6011
284.4471	18.8105	663.9935	81.9337	1034.8595	0.0563	1383.7918	104.4532	2961.8324	35.2587		
287.5355	32.7062	670.0222	5.9392	1035.4732	0.3152	1384.8310	149.5504	2961.9102	33.0054		
289.8033	7.2849	673.0649	47.4728	1035.5978	0.3752	1402.7725	33.2325	2962.1184	51.8410		
293.7565	9.6626	676.3683	83.2233	1035.6190	0.5200	1403.5030	61.6353	2962.1674	47.5385		
300.0806	5.7060	684.6246	10.0302	1035.7234	0.5500	1404.1841	11.8464	2962.2602	53.9500		
303.7848	22.5337	692.2611	79.3631	1036.3256	0.5436	1404.5195	4.7722	2962.5436	41.0178		
306.5841	317.2711	713.3996	283.5857	1093.9310	0.9103	1404.8961	15.7938	2962.8301	22.0348		
311.6082	104.8470	720.3413	160.1826	1098.7780	3.4107	1405.2336	4.0665	2962.8375	82.9947		
316.4236	28.9424	733.8706	8.5798	1114.0872	91.1216	1405.7170	58.8030	2963.0411	15.0599		
320.8900	84.7808	739.3913	38.9173	1117.1235	28.0941	1408.0412	66.3634	2964.1906	38.5838		
329.1476	20.7513	741.6893	3.7232	1130.2775	97.4547	1419.6890	17.0435	2966.3754	23.6419		
331.4604	103.1000	743.7755	17.2867	1130.9499	69.7301	1419.9033	20.0068	2968.8267	45.6180		
334.9625	463.0756	750.5086	38.9617	1162.6660	21.9562	1421.5987	9.2582	2969.0640	46.5801		
340.6578	18.8030	759.5030	238.7757	1166.0476	70.1325	1423.3563	20.0137	2969.2054	38.2207		
341.8047	3.9111	763.2406	118.2052	1175.6036	16.1252	1431.0527	52.6336	2969.6122	81.0864		
343.6242	37.3591	771.5640	74.8145	1176.9126	12.9368	1432.0291	115.7991	2969.7096	48.1245		
344.8065	27.0425	782.7131	315.5934	1181.1865	184.9262	1442.5847	36.1162	2970.8382	57.6763		
345.8224	23.5184	787.1209	9.8062	1189.4623	54.4745	1445.0148	26.7522	2985.4130	29.7603		

Table S22: A complete list of frequencies and intensities of *pc*-BCX

$\nu(cm^{-1})$	$\epsilon(M^{-1}cm^{-1})$	$\nu(cm^{-1})$	$\epsilon(M^{-1}cm^{-1})$	$\nu(cm^{-1})$	$\epsilon(M^{-1}cm^{-1})$	$\nu(cm^{-1})$	$\epsilon(M^{-1}cm^{-1})$	$\nu(cm^{-1})$	$\epsilon(M^{-1}cm^{-1})$	$\nu(cm^{-1})$	$\epsilon(M^{-1}cm^{-1})$
6.8854	10.3713	352.7482	0.1086	804.5321	104.3861	1191.3130	50.3031	1452.5546	0.2458	2989.9273	36.2254
7.7802	12.2550	353.1703	14.3798	804.5521	81.6239	1191.4113	211.3862	1452.5953	0.1538	2990.0752	44.4829
10.0093	19.8089	355.6687	2.2411	806.2049	74.4427	1192.4933	1.3469	1453.9055	320.1645	3023.5615	10.2469
16.7981	21.6592	356.1712	37.1776	806.3447	4.4918	1192.5791	48.7203	1455.0851	0.8126	3024.1619	10.9519
18.6781	65.0586	373.2431	4.0541	808.3315	20.0977	1193.0102	8.6351	1455.1000	1.4457	3028.9420	16.1264
23.0197	0.7452	373.6601	5.3030	808.4732	21.4280	1193.1172	15.4675	1455.6958	11.4149	3029.1772	13.0329
23.6314	34.7427	375.0400	1.7073	863.3812	99.9293	1193.4865	5.1289	1455.8579	9.0633	3032.0546	6.6601
29.0485	1.6343	375.2754	8.2355	867.3994	22.8243	1193.5451	45.8033	1456.0801	21.3054	3032.1109	7.1859
30.1380	10.8809	376.8169	10.6390	868.1942	54.5718	1194.4529	246.4870	1456.2088	8.9598	3032.4002	6.2710
36.2763	0.0990	377.0428	3.1277	871.0674	450.8330	1194.6234	22.2138	1456.4564	24.3866	3032.5411	10.1285
37.0820	0.5809	386.1837	16.7031	871.6177	241.3170	1196.1555	8.6865	1457.2736	283.2575	3032.5431	7.0964
37.5015	0.5319	387.0743	56.3912	871.8054	47.9356	1196.3852	82.7657	1458.7683	3.2957	3032.5916	13.0131
37.6418	3.3173	398.0741	38.2470	873.7495	105.9014	1197.9799	7.6416	1459.8617	20.2902	3032.6278	14.9545
37.9876	3.5706	418.5951	3.5958	875.5969	52.6679	1198.1073	43.5951	1460.2771	3.2376	3032.9084	13.9182
41.0799	4.0011	421.5204	37.0897	877.8157	62.2040	1199.1403	131.1783	1460.4120	124.0186	3033.5135	12.5883
44.1887	6.6266	426.5135	12.9275	877.9380	27.0698	1200.8337	44.4423	1463.7352	0.0717	3033.6563	10.1490
48.1995	6.1497	428.9290	14.9595	883.2182	0.0041	1202.3059	52.9761	1465.3456	201.4655	3033.8569	22.6631
51.8862	51.9990	429.8717	15.3099	885.6503	0.2351	1203.7970	162.8187	1467.1679	1.2875	3033.9317	10.1210
87.9170	100.3918	433.1015	0.0101	889.8453	16.8933	1231.3825	8.4441	1468.7530	409.3791	3033.9777	15.9201
89.8357	0.0400	433.8261	2.1601	891.2938	2.7030	1234.3772	0.0423	1469.5673	14.9584	3034.0430	10.8776
95.3469	0.0084	434.4794	32.6715	907.4822	9.6970	1239.9302	14.7748	1469.8845	46.7135	3039.8079	25.8721
99.0890	2.5243	434.8236	18.6458	907.6919	0.4105	1247.0069	90.5124	1474.5293	4.1517	3040.1022	24.3345
102.1313	82.1306	438.4125	22.8801	908.0195	2.5851	1252.2576	142.2995	1475.4087	9.0227	3040.1048	17.5604
105.5698	14.3183	439.0756	0.3071	908.1543	3.0601	1257.3594	6.9974	1475.5183	9.4301	3040.1898	38.6140
107.2276	5.6626	442.0959	6.9339	908.7811	6.7213	1263.4960	8.3075	1476.5841	16.2698	3040.6783	38.2477
110.2006	20.2039	444.8765	73.0137	908.9338	5.0461	1266.0697	4.0323	1478.1472	15.3172	3040.8707	34.4845
114.4055	75.7914	452.3304	0.0168	911.9968	0.5700	1266.9343	29.4928	1478.5957	134.5081	3041.3574	56.7176
116.9932	13.0260	454.2202	20.7067	912.5549	8.6804	1270.7595	231.6693	1480.1483	2.7850	3041.5049	42.0246
127.1288	117.8410	464.9637	23.5153	914.4938	5.6899	1275.7431	183.3694	1480.1583	23.9985	3041.6263	132.1053
134.7152	0.0207	470.8465	27.8197	915.3654	0.1717	1276.3276	3.1066	1480.8130	17.1932	3041.7756	239.5399
141.8493	180.3323	484.7154	37.4861	917.9138	1.8397	1277.3497	3.7553	1480.8901	10.5979	3041.7963	400.5807
143.5933	4.1813	485.4690	0.7790	918.0070	6.0910	1277.4384	62.3197	1480.9305	31.5129	3042.1095	104.0537
161.4081	8.2626	505.2529	19.9702	919.4667	0.0534	1280.1329	382.4886	1481.0124	16.5552	3046.4597	20.7861
178.5164	33.1212	511.5459	5.6587	919.8175	3.4992	1280.6754	0.5554	1483.3444	34.0706	3046.6847	20.6222
190.7632	287.0139	513.0063	6.2251	922.4675	5.0318	1282.6738	233.9078	1483.5150	24.8927	3047.2214	25.4769
196.7940	1.3238	516.9127	17.1064	924.7882	3.7673	1284.4418	111.6694	1486.2414	222.1540	3047.2763	20.1783
202.6421	1.0670	523.4818	27.2935	924.9284	2.7997	1292.9847	35.5206	1488.0615	74.2893	3047.3153	36.8378
210.3878	14.4852	526.8443	121.9731	927.0175	4.2351	1293.8790	0.3068	1491.4407	214.2133	3047.5693	28.7080
216.6881	255.2673	528.7184	49.5258	929.0086	0.2714	1296.7550	30.6463	1493.9604	45.2178	3048.9679	51.7588
221.6896	1.6808	528.8141	50.7458	930.3776	0.1136	1296.9623	9.2719	1494.1742	13.4761	3049.1389	47.8379
222.9104	0.0895	545.5817	55.5076	932.6876	20.8348	1307.9295	50.3963	1495.1961	37.1646	3049.8731	59.4442
226.7129	10.1726	548.7433	7.8837	935.0157	2.5698	1308.2803	3.4506	1495.2517	3.0429	3049.9226	63.4181
228.6866	19.8383	554.1099	39.7521	939.8027	26.0015	1316.4150	17.6724	1495.4442	30.4398	3050.4821	48.2093
229.0492	0.1759	559.3131	147.3204	942.8285	42.6317	1316.5191	0.2215	1495.6720	25.0355	3050.5628	44.5562
234.4556	14.0413	571.0244	5.5716	951.4517	0.5690	1319.3496	1.1968	1496.2049	128.8666	3104.1675	13.3952
235.5171	12.1757	571.3370	36.7485	951.5530	0.6155	1325.4298	8.6769	1503.1345	5.3023	3104.3292	33.5971
235.9997	13.0011	587.5607	27.3675	951.8205	0.8881	1328.8525	37.5276	1592.6398	0.3176	3104.4095	3.0798
236.5047	1.7273	587.6255	3.7706	951.9961	0.7711	1332.4541	0.6009	1592.6583	44.4411	3104.5468	21.2293
244.3710	24.5122	591.2685	34.0524	952.2475	1.2581	1368.7490	6.2012	1593.6367	2.0322	3108.0020	71.5461
250.6201	3.8888	595.8583	0.1386	952.2740	1.3582	1369.0210	31.9575	1594.0498	64.7161	3108.1946	35.0440
261.4127	62.8933	597.1117	0.9634	964.5726	10.8568	1369.4096	5.2386	1596.3398	14.2178	3115.2761	35.2003
265.7721	2.9601	599.1639	24.5118	966.0091	1.7638	1369.5808	22.2184	1597.7372	15.8411	3127.3144	2.7938
267.4853	113.3752	607.6356	38.8858	971.3676	88.0280	1369.8486	21.9764	1599.9182	0.5974	3127.3770	3.2304
272.3873	3.6527	608.2621	5.1971	972.4362	8.7026	1369.9158	14.5995	1600.8190	2.0941	3129.4478	23.9727
274.9806	0.5426	611.4696	17.7297	982.1864	0.0865	1370.9806	10.6336	1601.9794	11.4715	3129.4967	6.8423
275.8269	6.6358	626.0014	1.0209	983.3608	91.9010	1371.0246	18.6244	1603.2090	4.0464	3130.6345	17.6150
276.1679	25.4921	630.2233	6.7137	1030.8544	2.6734	1371.0410	11.9760	1604.6688	6.0626	3131.0037	17.7643
276.4007	1.8157	636.1515	3.3864	1031.0867	5.6775	1371.2681	15.8268	1604.9466	0.0584	3176.9250	5154.2923
280.3152	3.1210	668.5493	3.3679	1031.4169	0.3856	1371.6684	17.4192	2962.2813	38.5145	3194.7011	5074.1883
280.5600	50.7377	670.5790	2.0269	1031.5474	0.0410	1371.6861	17.9218	2962.3724	41.6211	3247.4759	23.4560
281.1033	11.2485	671.0277	22.4533	1031.8579	12.4423	1386.1519	32.6398	2962.8283	42.5711	3262.8035	547.5284
286.5477	21.5085	683.2519	0.5086	1032.1862	0.0356	1393.0186	34.1660	2962.9067	46.8444	3280.2674	59.7214
291.4947	11.7234	687.4167	61.1935	1035.2054	0.3580	1397.1148	0.0180	2963.0214	30.1929		
292.8831	124.8608	689.2425	753.3309	1035.2801	0.1098	1402.9619	33.7803	2963.0393	87.7434		
295.5415	0.4806	693.1448	141.6268	1035.3112	0.2744	1403.3398	1.3648	2963.4537	22.7230		
297.8749	16.1411	702.7805	148.6393	1035.4671	0.3783	1403.8549	17.0289	2963.5654	38.1963		
303.3297	7.9320	716.6096	5.5147	1035.7245	0.4838	1404.0455	6.4718	2963.7320	52.1791		
306.7084	4.2013	718.3937	1079.1565	1035.7632	0.3917	1404.4401	20.5980	2963.8518	41.2165		
315.3645	87.3149	725.9141	681.2471	1095.3422	11.1133	1404.5810	3.6498	2963.9312	38.1306		
317.7331	10.1916	733.2098	5.7522	1099.3765	0.9896	1408.4470	156.9534	2964.0293	40.1733		
326.5795	0.1612	735.1448	15.9886	1106.6180	1.9456	1412.1947	71.5631	2970.2535	30.4553		
333.3277	72.6793	735.6701	82.3057	1118.4361	101.3804	1414.4409	3.7667	2970.3534	24.4428		
340.3375	0.2508	741.7227	39.7534	1124.4333	104.0097	1419.9112	4.9415	2970.4707	62.7401		
342.5850	67.2405	746.3974	6.2166	1133.6360	39.2199	1422.2394	0.6107	2970.5654	61.5458		
347.0200	50.1095	755.5181	38.3585	1161.2652	0.3985	1424.1211	0.0056	2970.6830	105.9769		
347.0966	17.7002	770.9125	8.3657	1163.2006	19.6297	1434.4237	337.4642	2970.8168	61.1996		
347.6903	4.2224	777.9292	88.7363	1173.2929	25.8773	1451.7722	0.7227	2980.7836	20.9076		
350.8496	23.0847	781.4596	5.3465	1174.8766	6.6391	1451.8230	0.6894	2981.1884	15.4981		
351.1076	7.0833	791.3019	217.8691	1181.5641	8.5848	1452.2360	0.2308	2987.3148	28.4891		
352.1366	126.6233	792.1795	30.0965	1181.6539	2.0750	1452.3225	0.2329	2987.6005	29.2347		

Table S23: A complete list of frequencies and intensities of *wc*-BCX

$\nu(cm^{-1})$	$\epsilon(M^{-1}cm^{-1})$	$\nu(cm^{-1})$	$\epsilon(M^{-1}cm^{-1})$	$\nu(cm^{-1})$	$\epsilon(M^{-1}cm^{-1})$	$\nu(cm^{-1})$	$\epsilon(M^{-1}cm^{-1})$	$\nu(cm^{-1})$	$\epsilon(M^{-1}cm^{-1})$	$\nu(cm^{-1})$	$\epsilon(M^{-1}cm^{-1})$
13.6659	40.1395	349.0709	10.2240	792.6767	25.0398	1188.7379	23.6108	1451.8674	1.7404	2984.3075	37.2843
18.2798	11.6322	354.0498	12.0059	796.0666	5.0525	1188.8920	17.5039	1452.0809	6.2764	2984.8031	32.6716
20.9954	0.7981	355.4755	8.1791	803.5055	21.2924	1191.4285	19.5095	1453.2127	0.1729	2990.0583	44.2501
27.2159	4.1923	356.0003	38.0685	804.1012	73.2042	1191.5270	8.0449	1454.7730	0.1878	2990.2955	41.2073
29.2666	19.5472	358.6117	70.2627	807.3847	5.2050	1192.6257	61.2951	1454.8338	3.4976	3024.6761	6.8930
30.8641	1.1116	360.6283	1184.4328	807.6622	21.3127	1192.7384	12.1213	1455.3688	0.1360	3025.3022	7.0651
36.2772	9.6554	369.5445	17.2770	811.3334	47.0924	1193.2264	10.3133	1455.7134	3.9817	3031.0517	18.0750
37.7270	11.4732	374.5510	15.4293	811.4052	0.7434	1193.3548	15.9355	1456.7454	48.9133	3031.0773	13.6272
37.8683	1.7383	374.9904	27.9212	868.0364	50.1646	1193.6119	13.1342	1456.7595	118.9719	3031.5931	5.9878
38.6345	2.2511	375.7720	0.7750	868.2205	32.2457	1194.6976	53.2674	1457.2353	29.7363	3031.9254	3.2238
39.6463	17.9816	377.2468	1.8591	871.3309	59.3201	1194.9394	29.8930	1457.5896	22.0004	3032.0097	13.2472
46.0078	0.3642	378.3652	6.2008	871.3902	69.7319	1196.1975	6.3790	1458.6670	3.2305	3032.2647	13.6553
50.2485	4.4063	378.6136	81.4141	876.6417	34.9354	1199.1221	9.4249	1459.7777	112.1795	3032.8470	14.4729
53.1061	0.2554	381.9429	2.9340	878.2425	0.0173	1199.1766	9.8769	1460.1323	64.6278	3032.9102	10.8435
61.6921	15.1966	390.1408	0.0746	885.2704	14.3502	1200.5051	22.9453	1463.5596	14.5123	3033.5228	8.6142
61.7160	6.5223	391.2181	38.4686	885.7443	39.3029	1201.0414	45.6868	1463.6623	7.2251	3033.5953	8.1437
77.4600	6.1031	410.4886	20.1118	887.0463	7.1504	1207.0332	78.1709	1464.7283	22.4401	3037.3170	67.1083
85.8898	33.5772	411.7719	37.0735	887.4528	13.9724	1207.7875	69.8964	1464.8745	60.7140	3037.4570	47.0815
91.3078	17.0922	426.8871	7.1707	889.5466	0.5552	1212.9174	337.7835	1466.1315	93.3604	3037.6822	26.0990
95.9665	13.3608	427.5582	2.2450	889.8315	1.2074	1213.2846	8.8792	1468.1717	69.1126	3037.9258	14.5698
100.5103	4.8146	433.3833	10.8318	906.2470	0.0216	1226.8751	86.5378	1468.6627	42.9082	3038.8435	86.1708
100.6304	8.0477	435.5725	0.7437	907.3689	3.5121	1228.0180	41.0028	1469.4514	181.2245	3038.9714	72.6874
105.1423	26.9736	435.9291	36.8009	907.8321	1.9059	1235.4945	123.9712	1470.6606	110.6740	3040.1257	36.9807
105.6044	0.4155	436.4246	0.0338	908.1900	15.4724	1235.7225	17.8976	1470.7678	7.0475	3040.6685	37.3713
110.4268	27.8757	440.2177	1.0984	908.6451	8.4745	1249.0126	174.6390	1475.7461	17.4004	3041.1550	120.9237
111.8226	69.7433	440.7510	11.3088	908.9518	11.3702	1249.1620	163.0325	1475.8288	5.7534	3041.3753	95.1669
114.1861	25.9936	441.1691	2.3801	909.0171	10.9015	1262.2068	20.2731	1476.0734	26.7733	3043.1891	94.4572
115.0721	26.6741	441.9267	0.6202	909.8983	6.1983	1262.6951	68.6999	1476.3715	20.5454	3043.4539	95.9157
120.9760	79.2003	444.5702	10.6391	913.2743	9.2781	1266.8105	103.6167	1479.0728	5.7990	3046.8306	86.0143
127.5194	11.1530	447.6982	0.1898	915.5067	16.4298	1266.9833	66.8480	1480.2559	15.5875	3047.0055	66.5250
137.5732	1.4586	468.2317	18.0831	918.2572	0.3345	1273.2185	57.8288	1480.5171	9.9916	3047.0978	18.4751
146.0100	136.9936	479.9488	0.6949	918.7638	2.0078	1273.9128	92.8300	1480.7040	23.9107	3047.1477	10.6652
158.4973	2.8493	485.3583	22.0480	920.5721	1.0223	1277.6173	6.8992	1480.8646	19.0808	3047.3714	17.1731
163.2554	115.4211	491.6801	0.6085	920.7998	8.3943	1278.6900	46.5313	1482.8487	53.2460	3047.5146	22.5903
173.6813	4.2195	496.5010	0.6307	924.3492	2.1148	1285.4764	180.7340	1485.0992	16.8771	3049.8167	72.5413
177.8349	119.0527	507.2617	7.6554	924.5418	5.6703	1286.3937	31.6995	1488.7520	49.2450	3049.8443	47.7120
195.3913	0.7269	512.4674	3.8355	928.1072	0.8197	1287.1572	13.1457	1489.7643	63.5306	3051.1460	42.4941
196.3351	142.6742	519.3511	1.3059	928.5052	0.3407	1287.4135	15.7467	1490.2499	136.4196	3051.5359	44.7131
201.0423	20.6949	521.1760	54.1395	930.0817	6.1890	1296.9324	4.6983	1493.0671	8.6993	3061.9459	67.8104
214.3292	0.8804	522.9174	28.6122	933.2494	14.4683	1298.3950	154.3796	1494.5332	359.7481	3063.8772	26.4164
216.8313	0.2410	528.0284	3.7346	934.0633	11.6898	1304.9998	17.1144	1494.6774	14.2467	3067.6821	41.6364
220.1423	13.2689	530.8585	0.8958	937.9745	20.2491	1306.2816	22.7857	1495.7614	10.1364	3070.6539	1.1604
225.9241	0.0618	544.3629	28.0589	940.5397	13.5078	1313.1334	54.0088	1495.8826	52.1385	3072.8983	23.3928
229.6437	13.2706	545.5218	5.5103	947.4808	68.4771	1313.5233	19.1530	1496.1328	4.0405	3073.2092	5.5356
235.1226	15.0992	554.8363	34.5160	949.6509	3.3271	1320.5134	57.2721	1497.3574	9.6895	3100.9902	20.6778
236.5144	0.0270	556.1090	1.4183	949.9914	1.3623	1320.7724	27.6418	1502.4111	14.7833	3101.3936	21.8594
242.5973	50.0950	560.1481	85.4331	950.3170	2.1506	1325.1686	14.6096	1589.5861	8.2532	3107.7526	16.1598
244.8899	0.3845	565.7336	0.0769	950.3976	1.7307	1325.3889	23.3806	1590.4662	41.1610	3108.8980	16.2391
246.0091	4.5017	577.9695	58.0315	951.3044	0.1656	1359.4071	52.0192	1592.9326	19.5509	3124.5047	16.9770
246.8821	31.5361	578.1496	10.9356	952.1607	2.0497	1359.9717	9.2638	1593.6199	0.7135	3124.5443	12.0491
256.0755	8.0388	591.4648	48.9258	958.9716	38.1874	1365.2861	14.5625	1595.3296	0.3641	3127.7693	8.1791
257.9857	126.0240	593.3848	14.1461	959.4289	5.6924	1365.6288	14.8197	1595.4237	0.2996	3128.3750	8.1361
258.2159	27.2134	598.9756	28.4125	970.6444	2.9892	1368.4386	2.4725	1601.3712	5.3716	3131.9657	11.7147
261.8309	12.0948	599.4427	4.3538	971.7316	67.8200	1368.5750	44.7190	1601.5742	19.9124	3132.0030	8.0966
275.1926	0.5770	602.1226	1.3350	986.7707	5.3952	1370.3479	34.0011	1604.3945	2.3386	3143.0974	2.4597
276.0558	17.9790	608.6497	19.3627	987.3336	1.4174	1370.4627	37.7651	1604.6290	5.2588	3143.8940	2.5352
277.5110	2.8593	619.6442	30.3021	1030.3086	2.1978	1370.6270	29.3593	1607.5299	4.1494	3426.1948	227.0508
277.9141	1.5001	621.5555	124.2168	1030.4382	1.6458	1370.8050	12.8395	1607.6209	7.6206	3431.9207	2090.6441
282.2139	10.5003	624.1785	296.9800	1030.8573	0.1761	1371.1035	8.6704	2931.5240	48.3724	3492.4335	536.0405
282.8109	12.6984	628.2318	163.3233	1031.1730	0.4097	1372.5885	3.7857	2931.5783	49.0187	3493.4178	344.3209
284.6265	4.7109	637.0455	86.7197	1031.4574	9.2133	1372.6395	27.2286	2961.4620	69.6721	3730.4556	58.7715
286.2754	3.3738	641.6908	318.4389	1031.6932	1.8433	1373.3539	25.5651	2961.5292	30.1061	3730.9439	74.9227
294.4096	47.5148	660.6828	1.1400	1032.7627	0.3623	1374.1318	60.8142	2961.6361	45.9595		
295.8215	0.2967	661.1924	129.2068	1035.0134	0.9540	1375.6557	18.0986	2961.7887	53.1259		
298.5524	8.7043	672.5911	12.2780	1035.0316	0.1985	1381.5432	228.8661	2963.1698	44.5634		
303.6827	16.4564	673.4966	2.7029	1035.1604	0.1283	1382.0291	174.3902	2963.2255	48.5633		
308.9939	308.0056	681.0983	4.2073	1035.1694	0.0794	1403.3970	0.4702	2963.5962	38.9388		
311.0032	28.7824	683.3448	99.8819	1037.1848	1.4762	1403.6932	0.2146	2964.0938	24.4909		
315.2056	0.7277	693.0846	669.6891	1098.2107	2.9377	1403.8100	10.3923	2967.0940	35.3230		
316.2213	15.7206	695.1151	581.7444	1098.9196	13.1620	1403.9453	8.4937	2967.3907	32.3974		
317.7540	67.0336	724.4620	7.7622	1112.0658	72.7584	1404.1234	24.7977	2969.7859	105.8235		
319.2200	5.5413	725.0373	110.3911	1113.6116	76.5533	1407.5324	13.7306	2969.8331	18.6706		
328.2529	27.2237	737.8159	5.7023	1129.2160	124.2052	1419.9444	3.9940	2971.2140	22.2619		
331.4900	5.1846	739.4255	2.7699	1129.7942	37.0333	1420.4526	2.6341	2971.6275	48.6727		
334.8431	18.2347	741.2839	24.4266	1168.4192	29.9272	1421.4327	25.3748	2973.1172	10.3485		
336.1560	3.0903	747.5281	2.4544	1168.6675	40.2685	1421.4888	37.5888	2973.4983	9.5291		
336.5934	4.0499	752.1166	104.4795	1173.8304	133.6662	1424.0353	1.6134	2975.3580	39.8763		
345.2593	0.7522	759.2361	23.6079	1174.1561	605.6748	1424.3602	27.1577	2975.5510	31.6612		
347.3715	4.4376	774.0923	5.3268	1178.3322	0.4696	1451.4810	0.5214	2976.5486	34.7863		
348.5950	9.8020	785.1750	118.7483	1178.6559	17.7670	1451.5212	1.6936	2977.7537	89.3152		

Table S24: The total SAPT0 interaction energies of unfragmented and the SSMF3 fragments for *al*-BCX conformer + Gly complex are presented. Energies are in millihartree.

No.	weight	E_{tot}^{SAPT0}	No.	weight	E_{tot}^{SAPT0}	No.	weight	E_{tot}^{SAPT0}
1	-1.000	-12.664	51	0.500	-16.709	101	-0.167	-0.366
2	-1.000	-8.466	52	0.500	-17.573	102	-0.167	-0.502
3	-1.000	-8.394	53	-0.500	-9.500	103	-0.167	-0.463
4	-1.000	-8.074	54	-0.500	-9.853	104	0.500	-4.996
5	1.000	-8.500	55	-0.500	-9.641	105	0.500	-5.205
6	0.500	-12.796	56	-0.167	-1.503	106	0.500	-5.221
7	0.500	-12.711	57	-0.167	-1.253	107	0.500	-0.538
8	-1.000	-17.355	58	-0.167	-1.281	108	0.167	-5.208
9	-1.000	-11.174	59	0.500	-10.035	109	0.167	-5.235
10	-1.000	-12.054	60	0.500	-9.842	110	0.167	-5.444
11	-1.000	-10.312	61	0.500	-10.117	111	0.167	-4.907
12	1.000	-10.705	62	0.500	-1.464	112	0.167	-4.926
13	0.500	-15.776	63	-1.000	-10.364	113	0.167	-5.133
14	0.500	-16.868	64	-1.000	-10.308	114	-1.000	-15.320
15	-0.500	-2.576	65	-1.000	-10.442	115	0.500	-15.774
16	-0.500	-2.608	66	1.000	-11.451	116	0.500	-15.357
17	-0.500	-2.615	67	0.500	-17.169	117	-0.500	-8.053
18	-0.167	-0.042	68	0.500	-17.187	118	-0.500	-8.564
19	-0.167	-0.042	69	0.500	-13.363	119	-0.500	-8.741
20	-0.167	-0.052	70	0.500	-13.258	120	-0.167	-1.048
21	0.500	-2.600	71	0.167	-10.533	121	-0.167	-0.986
22	0.500	-2.600	72	0.167	-10.874	122	-0.167	-1.285
23	0.500	-2.613	73	0.167	-10.662	123	0.500	-8.627
24	0.500	-0.064	74	0.167	-10.668	124	0.500	-8.820
25	-1.000	-3.272	75	0.167	-11.014	125	0.500	-9.205
26	-1.000	-2.805	76	0.167	-10.796	126	0.500	-1.363
27	-1.000	-2.818	77	-1.000	-18.149	127	0.500	-15.636
28	1.000	-3.018	78	0.500	-18.359	128	0.500	-16.575
29	0.500	-17.498	79	0.500	-18.883	129	0.167	-8.548
30	0.500	-17.126	80	-0.500	-11.691	130	0.167	-9.047
31	0.500	-12.914	81	-0.500	-11.636	131	0.167	-9.226
32	0.500	-12.435	82	-0.500	-11.688	132	0.167	-8.376
33	0.167	-2.777	83	-0.167	-0.057	133	0.167	-8.892
34	0.167	-2.809	84	-0.167	-0.061	134	0.167	-9.050
35	0.167	-2.815	85	-0.167	-0.055	135	-0.500	-8.002
36	0.167	-2.791	86	0.500	-11.680	136	-0.500	-7.988
37	0.167	-2.824	87	0.500	-11.688	137	-0.500	-7.984
38	0.167	-2.831	88	0.500	-11.678	138	-0.167	-0.028
39	-1.000	-12.155	89	0.500	-0.075	139	-0.167	-0.025
40	-1.000	-4.427	90	0.500	-18.588	140	-0.167	-0.016
41	-1.000	-5.125	91	0.500	-17.702	141	0.500	-8.011
42	-1.000	-4.821	92	0.167	-12.076	142	0.500	-8.008
43	1.000	-5.113	93	0.167	-12.020	143	0.500	-8.001
44	0.500	-12.202	94	0.167	-12.072	144	0.500	-0.031
45	0.500	-12.923	95	0.167	-10.337	145	0.167	-8.419
46	-1.000	-16.146	96	0.167	-10.289	146	0.167	-8.406
47	-1.000	-7.490	97	0.167	-10.333	147	0.167	-8.401
48	-1.000	-8.486	98	-0.500	-4.896	148	0.167	-8.106
49	-1.000	-8.319	99	-0.500	-4.922	149	0.167	-8.089
50	1.000	-8.798	100	-0.500	-5.132	150	0.167	-8.082
Sum								-55.022
No Fragmentation								-57.705

Table S25: The total SAPT0 interaction energies of unfragmented and the SSMF3 fragments for *pc*-BCX conformer + Gly complex are presented. Energies are in millihartree.

No.	weight	E_{tot}^{SAPT0}	No.	weight	E_{tot}^{SAPT0}	No.	weight	E_{tot}^{SAPT0}
1	-1.000	-11.088	54	0.500	-1.502	104	-0.167	-0.043
2	-1.000	-19.245	55	0.167	0.891	105	0.500	-1.915
3	-1.000	-12.846	56	0.167	0.900	106	0.500	-1.911
4	-1.000	-12.928	57	0.167	0.898	107	0.500	-1.920
5	-1.000	-12.850	58	0.167	0.843	108	0.500	-0.051
6	1.000	-13.137	59	0.167	0.856	109	0.500	-12.562
7	0.500	-11.367	60	0.167	0.850	110	0.500	-12.405
8	0.500	-11.400	61	-1.000	-8.049	111	0.167	-2.028
9	0.500	-19.563	62	-1.000	-3.671	112	0.167	-2.050
10	0.500	-19.400	63	-1.000	-3.271	113	0.167	-2.037
11	-0.167	-0.018	64	-1.000	-3.281	114	0.167	-1.190
12	-0.167	-0.014	65	1.000	-3.399	115	0.167	-1.216
13	-0.500	-12.656	66	0.500	-8.144	116	0.167	-1.205
14	-0.167	-0.012	67	0.500	-7.780	117	-1.000	-15.160
15	-0.500	-12.669	68	-0.500	-3.531	118	0.500	-14.087
16	-0.500	-12.648	69	-0.500	-3.523	119	0.500	-14.664
17	0.500	-0.016	70	-0.500	-3.534	120	-0.500	-3.120
18	0.500	-12.686	71	-0.167	-0.023	121	-0.500	-3.156
19	0.500	-12.680	72	-0.167	-0.020	122	-0.500	-3.154
20	0.500	-12.680	73	-0.167	-0.023	123	-0.167	-0.039
21	0.167	-12.874	74	0.500	-3.531	124	-0.167	-0.040
22	0.167	-12.952	75	0.500	-3.533	125	-0.167	-0.046
23	0.167	-12.884	76	0.500	-3.532	126	0.500	-3.141
24	0.167	-12.963	77	0.500	-0.032	127	0.500	-3.143
25	0.167	-12.865	78	-1.000	-3.824	128	0.500	-3.155
26	0.167	-12.945	79	-1.000	-3.537	129	0.500	-0.060
30	-1.000	-1.590	80	-1.000	-3.660	130	0.500	-15.253
31	-1.000	-1.390	81	1.000	-3.667	131	0.500	-14.909
32	-1.000	-2.027	82	0.500	-19.417	132	0.167	-3.243
33	-1.000	-1.192	83	0.500	-19.119	133	0.167	-3.278
34	1.000	-1.320	84	0.500	-8.044	134	0.167	-3.275
35	0.500	-0.851	85	0.500	-7.905	135	0.167	-3.252
36	0.500	-1.538	86	0.167	-3.539	136	0.167	-3.288
37	-0.500	1.116	87	0.167	-3.534	137	0.167	-3.287
38	-0.500	1.129	88	0.167	-3.540	138	-0.500	-9.941
39	-0.500	1.123	89	0.167	-3.659	139	-0.500	-9.889
40	-0.167	-0.034	90	0.167	-3.651	140	-0.500	-9.936
41	-0.167	-0.037	91	0.167	-3.661	141	-0.167	-0.065
42	-0.167	-0.037	92	-1.000	-12.456	142	-0.167	-0.064
43	0.500	1.111	93	-1.000	-9.705	143	-0.167	-0.060
44	0.500	1.108	94	-1.000	-8.994	144	0.500	-9.927
45	0.500	1.110	95	-1.000	-10.139	145	0.500	-9.940
46	0.500	-0.043	96	1.000	-9.223	146	0.500	-9.927
47	-1.000	0.534	97	0.500	-12.717	147	0.500	-0.087
48	-1.000	0.910	98	0.500	-12.011	148	0.167	-9.016
49	-1.000	0.862	99	-0.500	-1.888	149	0.167	-8.966
50	1.000	0.633	100	-0.500	-1.910	150	0.167	-9.010
51	0.500	-11.381	101	-0.500	-1.897	151	0.167	-10.157
52	0.500	-11.015	102	-0.167	-0.039	152	0.167	-10.107
53	0.500	-1.816	103	-0.167	-0.036	153	0.167	-10.152
Sum								-36.179
No Fragmentation								-36.400

Table S26: The total SAPT0 interaction energies of unfragmented and the SSMF3 fragments for *wc*-BCX conformer + Gly complex are presented. Energies are in millihartree.

No.	weight	E_{tot}^{SAPT0}	No.	weight	E_{tot}^{SAPT0}	No.	weight	E_{tot}^{SAPT0}
1	-1.000	-21.689	54	0.500	-23.018	104	-0.167	-0.023
2	-1.000	-9.639	55	0.167	-15.755	105	0.500	-7.546
3	-1.000	-3.350	56	0.167	-15.723	106	0.500	-7.551
4	-1.000	-3.249	57	0.167	-15.754	107	0.500	-7.547
5	-1.000	-3.384	58	0.167	-15.203	108	0.500	-0.031
6	1.000	-3.344	59	0.167	-15.176	109	0.500	-9.384
7	0.500	-21.631	60	0.167	-15.203	110	0.500	-11.084
8	0.500	-21.623	61	-1.000	-15.489	111	0.167	-6.030
9	0.500	-9.603	62	-1.000	-10.328	112	0.167	-6.028
10	0.500	-9.764	63	-1.000	-10.061	113	0.167	-6.031
11	-0.167	-0.016	64	-1.000	-10.178	114	0.167	-7.710
12	-0.167	-0.019	65	1.000	-10.112	115	0.167	-7.713
13	-0.500	-3.260	66	0.500	-15.577	116	0.167	-7.713
14	-0.167	-0.019	67	0.500	-15.282	117	-1.000	-14.217
15	-0.500	-3.259	68	-0.500	-5.625	118	0.500	-14.214
16	-0.500	-3.256	69	-0.500	-5.617	119	0.500	-14.147
17	0.500	-0.023	70	-0.500	-5.625	120	-0.500	-10.131
18	0.500	-3.263	71	-0.167	-0.024	121	-0.500	-10.149
19	0.500	-3.264	72	-0.167	-0.035	122	-0.500	-10.110
20	0.500	-3.262	73	-0.167	-0.023	123	-0.167	-0.100
21	0.167	-3.355	74	0.500	-5.635	124	-0.167	-0.086
22	0.167	-3.254	75	0.500	-5.646	125	-0.167	-0.100
23	0.167	-3.354	76	0.500	-5.634	126	0.500	-10.156
24	0.167	-3.252	77	0.500	-0.037	127	0.500	-10.133
25	0.167	-3.352	78	-1.000	-5.592	128	0.500	-10.155
26	0.167	-3.251	79	-1.000	-5.737	129	0.500	-0.118
30	-1.000	-23.418	80	-1.000	-5.629	130	0.500	-14.101
31	-1.000	-6.035	81	1.000	-5.741	131	0.500	-13.992
32	-1.000	-6.013	82	0.500	-9.575	132	0.167	-10.085
33	-1.000	-7.694	83	0.500	-9.803	133	0.167	-10.098
34	1.000	-6.183	84	0.500	-15.589	134	0.167	-10.060
35	0.500	-23.569	85	0.500	-15.637	135	0.167	-10.194
36	0.500	-23.590	86	0.167	-5.756	136	0.167	-10.211
37	-0.500	-15.691	87	0.167	-5.748	137	0.167	-10.174
38	-0.500	-15.656	88	0.167	-5.755	138	-0.500	-2.776
39	-0.500	-15.688	89	0.167	-5.646	139	-0.500	-2.775
40	-0.167	-0.105	90	0.167	-5.633	140	-0.500	-2.771
41	-0.167	-0.100	91	0.167	-5.645	141	-0.167	-0.019
42	-0.167	-0.087	92	-1.000	-10.930	142	-0.167	-0.021
43	0.500	-15.695	93	-1.000	-2.931	143	-0.167	-0.021
44	0.500	-15.696	94	-1.000	-2.800	144	0.500	-2.780
45	0.500	-15.687	95	-1.000	-2.840	145	0.500	-2.779
46	0.500	-0.120	96	1.000	-2.868	146	0.500	-2.780
47	-1.000	-15.630	97	0.500	-11.024	147	0.500	-0.026
48	-1.000	-15.730	98	0.500	-10.877	148	0.167	-2.804
49	-1.000	-15.175	99	-0.500	-7.529	149	0.167	-2.804
50	1.000	-15.243	100	-0.500	-7.530	150	0.167	-2.801
51	0.500	-21.143	101	-0.500	-7.532	151	0.167	-2.845
52	0.500	-21.295	102	-0.167	-0.023	152	0.167	-2.844
53	0.500	-23.512	103	-0.167	-0.028	153	0.167	-2.840
Sum								-49.723
No Fragmentation								-49.950

Table S27: The accuracy of the SSMF3-HF(b) and SSMF3-MP2(b) electric dipole moments and dipole static polarizabilities for three conformers of calix[6]arene. "(b)" denotes that only the bonded part of the property has been used in calculations. The same orientation as presented the Supplementary information has been used to define axes. The cc-pVDZ basis has been used. All data in a.u.

	al-CX				*pc*-CX				*wc*-CX			
method	μ_x	μ_y	μ_z	μ_{tot}	μ_x	μ_y	μ_z	μ_{tot}	μ_x	μ_y	μ_z	μ_{tot}
SSMF3-HF(b)	-0.31	-0.22	-0.09	0.39	0.00	0.00	0.66	0.66	0.00	0.00	0.50	0.50
HF	-0.28	-0.19	-0.13	0.37	0.00	0.00	0.71	0.71	0.00	0.00	0.51	0.51
SSMF3-MP2(b)	-0.09	-0.23	0.01	0.25	0.00	0.00	0.37	0.37	0.00	0.00	0.16	0.16
MP2	-0.07	-0.21	-0.03	0.22	0.00	0.00	0.46	0.46	0.00	0.00	0.19	0.19
	α_{xx}	α_{yy}	α_{zz}	α_{aver}	α_{xx}	α_{yy}	α_{zz}	α_{aver}	α_{xx}	α_{yy}	α_{zz}	α_{aver}
SSMF3-HF(b)	511.87	447.78	358.77	439.47	518.82	456.14	338.79	437.92	526.60	400.05	382.25	436.30
HF	506.38	451.05	345.66	434.36	523.71	466.53	323.01	437.75	518.95	396.16	356.71	423.94
SSMF3-MP2(b)	531.93	465.22	368.23	455.13	540.83	473.36	347.32	453.84	548.28	413.68	392.97	451.64
MP2	525.99	468.95	353.86	449.60	546.39	484.98	329.92	453.77	540.07	409.14	365.69	438.30
	α_{xy}	α_{xz}	α_{yz}		α_{xy}	α_{xz}	α_{yz}		α_{xy}	α_{xz}	α_{yz}	
SSMF3-HF(b)	-1.27	-22.37	1.21		-2.22	0.00	0.00		17.19	0.00	0.00	
HF	-1.89	-28.53	1.05		-3.13	0.00	0.00		24.10	0.00	0.00	
SSMF3-MP2(b)	-1.36	-23.04	1.45		-2.39	0.00	0.00		18.15	0.00	0.00	
MP2	-2.05	-29.80	1.25		-3.44	0.00	0.00		26.02	0.00	0.00	

Table S28: The F-SAPT partitioning for selected CX+ amino acid pairs. Energies are in millihartree.

Complex	Group A	Group B	E_{elst} (ratio)	E_{exch} (ratio)	$E_{ind,eff}$ (ratio)	$E_{disp,eff}$ (ratio)	E_{total}	bond type
al-CX Ala	OH-5	NH2	-44.2 (1.9)	49.9 (2.1)	-22.5 (1.0)	-6.7 (0.3)	-23.5	typical H-bond
al-CX Asn	OH-2	NH2-1	-53.9 (1.8)	60.7 (2.0)	-29.0 (1.0)	-7.5 (0.3)	-29.7	typical H-bond
al-CX AspH	OH-2	NH2	-46.3 (1.7)	48.3 (1.8)	-22.5 (0.8)	-6.3 (0.2)	-26.8	typical H-bond
al-CX Cys	OH-2	NH2	-35.6 (1.8)	35.8 (1.8)	-14.7 (0.7)	-5.2 (0.3)	-19.8	typical H-bond
al-CX Gln	OH-2	CONH	-28.3 (1.7)	30.0 (1.8)	-13.5 (0.8)	-5.0 (0.3)	-16.8	typical H-bond
al-CX Gln	OH-5	NH2	-35.4 (1.8)	36.7 (1.8)	-15.9 (0.8)	-5.4 (0.3)	-19.9	typical H-bond
al-CX Gly	OH-2	NH2	-39.5 (1.7)	39.1 (1.7)	-16.9 (0.7)	-5.4 (0.2)	-22.8	typical H-bond
al-CX HisD	OH-5	COOH	-15.0 (1.5)	12.9 (1.3)	-5.3 (0.5)	-2.9 (0.3)	-10.3	typical H-bond
al-CX Ile	OH-5	COOH	-14.8 (1.4)	13.7 (1.3)	-6.1 (0.6)	-3.1 (0.3)	-10.2	typical H-bond
al-CX Leu	OH-2	NH2	-39.3 (1.6)	37.3 (1.5)	-16.9 (0.7)	-5.3 (0.2)	-24.2	typical H-bond
al-CX Lys	OH-2	NH2-2	-46.9 (1.6)	49.0 (1.7)	-24.6 (0.8)	-6.5 (0.2)	-28.9	typical H-bond
al-CX Met	OH-5	COOH	-17.0 (1.5)	14.6 (1.3)	-6.1 (0.5)	-3.1 (0.3)	-11.5	typical H-bond
al-CX Pro	OH-6	COOH	-25.9 (1.6)	22.6 (1.4)	-9.0 (0.5)	-4.4 (0.3)	-16.7	typical H-bond
al-CX Trp	OH-5	NH2	-28.9 (1.5)	24.3 (1.2)	-11.1 (0.6)	-4.0 (0.2)	-19.6	typical H-bond
al-CX Tyr	OH-6	OH	-21.3 (1.7)	18.3 (1.5)	-6.3 (0.5)	-3.2 (0.3)	-12.5	typical H-bond
al-CX Val	OH-2	NH2	-29.0 (1.6)	26.2 (1.4)	-11.3 (0.6)	-4.1 (0.2)	-18.3	typical H-bond
wc-CX Ala	OH-3	NH2	-40.1 (1.9)	42.2 (2.0)	-17.9 (0.8)	-5.8 (0.3)	-21.6	typical H-bond
wc-CX Cys	OH-5	COOH	-29.7 (1.6)	25.9 (1.4)	-10.7 (0.6)	-4.5 (0.2)	-19.0	typical H-bond
wc-CX Gln	OH-5	COOH	-30.3 (1.7)	27.7 (1.5)	-11.1 (0.6)	-4.7 (0.3)	-18.4	typical H-bond
wc-CX Gln	OH-6	CONH	-24.1 (1.4)	20.5 (1.2)	-9.2 (0.5)	-4.0 (0.2)	-16.8	typical H-bond
wc-CX GluH	OH-2	COOH-2	-33.8 (1.5)	28.9 (1.3)	-12.2 (0.6)	-5.0 (0.2)	-22.2	typical H-bond
wc-CX Gly	OH-3	NH2	-39.9 (1.9)	42.2 (2.0)	-18.0 (0.8)	-5.8 (0.3)	-21.4	typical H-bond
wc-CX HisD	OH-3	NH2	-37.9 (1.8)	39.4 (1.9)	-16.7 (0.8)	-5.6 (0.3)	-20.7	typical H-bond
wc-CX HisE	OH-3	NH2	-34.9 (1.8)	34.1 (1.7)	-14.0 (0.7)	-5.0 (0.3)	-19.7	typical H-bond
wc-CX Ile	OH-3	NH2	-35.7 (1.8)	35.3 (1.7)	-14.7 (0.7)	-5.1 (0.3)	-20.2	typical H-bond
wc-CX Leu	OH-3	NH2	-31.4 (1.7)	29.8 (1.6)	-12.3 (0.7)	-4.5 (0.2)	-18.4	typical H-bond
wc-CX Met	OH-6	NH2	-44.5 (1.9)	48.3 (2.0)	-21.3 (0.9)	-6.4 (0.3)	-24.0	typical H-bond
wc-CX Phe	OH-3	NH2	-42.5 (1.9)	45.6 (2.0)	-19.3 (0.9)	-6.1 (0.3)	-22.3	typical H-bond
wc-CX Ser	OH-6	NH2	-31.2 (1.8)	29.0 (1.6)	-11.1 (0.6)	-4.4 (0.2)	-17.7	typical H-bond
wc-CX Thr	OH-3	NH2	-32.9 (1.8)	33.5 (1.9)	-13.5 (0.8)	-5.0 (0.3)	-17.9	typical H-bond
wc-CX Trp	OH-6	NH2	-32.9 (1.8)	33.0 (1.8)	-13.8 (0.7)	-5.0 (0.3)	-18.7	typical H-bond
wc-CX Tyr	OH-6	NH2	-32.1 (1.7)	30.7 (1.6)	-12.7 (0.7)	-4.6 (0.2)	-18.7	typical H-bond
wc-CX Val	OH-3	NH2	-36.6 (1.8)	37.1 (1.9)	-15.1 (0.8)	-5.3 (0.3)	-19.8	typical H-bond
al-CX Cys-	OH-6	COOH	-19.9 (1.6)	17.9 (1.5)	-6.4 (0.5)	-3.8 (0.3)	-12.2	typical H-bond+disp
al-CX HisE	OH-2	Ring	-39.6 (1.9)	51.2 (2.5)	-23.9 (1.2)	-8.1 (0.4)	-20.5	typical H-bond+disp
al-CX Phe	OH-5	COOH	-13.1 (1.6)	13.8 (1.7)	-5.6 (0.7)	-3.1 (0.4)	-8.0	typical H-bond+disp
al-CX Pro	OH-2	Ring	-44.7 (1.7)	54.0 (2.1)	-26.2 (1.0)	-8.7 (0.3)	-25.5	typical H-bond+disp
pc-CX Gln	OH-6	COOH	-19.3 (2.0)	19.1 (2.0)	-5.7 (0.6)	-3.7 (0.4)	-9.6	typical H-bond+disp
pc-CX Thr	OH-1	COOH	-21.2 (1.9)	21.7 (1.9)	-7.5 (0.7)	-4.3 (0.4)	-11.4	typical H-bond+disp
wc-CX Asn	OH-3	CONH2	-15.4 (1.6)	16.0 (1.7)	-6.7 (0.7)	-3.5 (0.4)	-9.6	typical H-bond+disp
wc-CX Asn	OH-6	NH2-1	-47.0 (2.1)	53.8 (2.4)	-21.9 (1.0)	-6.9 (0.3)	-22.1	typical H-bond+disp
wc-CX GluH	OH-3	COOH-1	-17.2 (1.8)	17.8 (1.8)	-6.8 (0.7)	-3.5 (0.4)	-9.8	typical H-bond+disp
wc-CX HisD	OH-6	Ring	-18.8 (1.7)	22.6 (2.0)	-9.7 (0.9)	-5.4 (0.5)	-11.3	typical H-bond+disp
wc-CX Lys	OH-3	NH2-2	-40.1 (2.1)	45.8 (2.4)	-19.0 (1.0)	-6.0 (0.3)	-19.3	typical H-bond+disp
wc-CX Pro	OH-3	Ring	-26.8 (1.8)	29.1 (2.0)	-11.2 (0.8)	-5.8 (0.4)	-14.6	typical H-bond+disp
pc-CX AspH	OH-3	COOH-1	-15.2 (1.4)	9.4 (0.8)	-2.5 (0.2)	-2.9 (0.3)	-11.2	$H\cdots\pi$
pc-CX GluH	OH-5	COOH-1	-11.7 (1.4)	7.6 (0.9)	-1.9 (0.2)	-2.6 (0.3)	-8.6	$H\cdots\pi$
wc-CX Gln	OH-1	CONH	-14.0 (1.2)	7.8 (0.7)	-3.0 (0.3)	-2.4 (0.2)	-11.6	$H\cdots\pi$
wc-CX Leu	Ph-6	COOH	-19.7 (0.9)	7.5 (0.3)	-4.7 (0.2)	-4.8 (0.2)	-21.7	$H\cdots\pi$
wc-CX Pro	Ph-6	COOH	-16.6 (0.9)	6.2 (0.3)	-3.8 (0.2)	-4.5 (0.2)	-18.6	$H\cdots\pi$
al-CX GluH	Ph-5	COOH-2	-6.8 (0.8)	6.0 (0.7)	-3.0 (0.3)	-4.9 (0.6)	-8.7	disp
pc-CX HisE	Ph-6	Ring	-11.9 (0.9)	10.1 (0.8)	-2.7 (0.2)	-8.5 (0.7)	-13.0	disp
wc-CX HisE	Ph-2	Ring	-11.8 (0.9)	7.5 (0.6)	-1.7 (0.1)	-7.2 (0.5)	-13.2	disp
al-CX AspH	Ph-3	COOH-2	-8.6 (0.9)	0.1 (0.0)	-0.2 (0.0)	-1.1 (0.1)	-9.7	elst
al-CX Cys	Ph-3	COOH	-9.7 (0.8)	0.7 (0.1)	-1.3 (0.1)	-1.7 (0.1)	-12.0	elst
al-CX Cys	Ph-4	COOH	-6.3 (0.8)	0.5 (0.1)	-0.7 (0.1)	-1.7 (0.2)	-8.1	elst
al-CX Gln-	Ph-3	CONH	-7.2 (0.8)	0.4 (0.0)	-1.1 (0.1)	-1.5 (0.2)	-9.2	elst
al-CX GluH	Ph-4	COOH-2	-7.7 (0.8)	0.4 (0.0)	-1.1 (0.1)	-1.6 (0.2)	-10.0	elst
al-CX HisD	Ph-4	COOH	-11.7 (0.8)	0.3 (0.0)	-2.3 (0.2)	-1.5 (0.1)	-15.1	elst
al-CX HisD	Ph-5	COOH	-8.0 (0.8)	0.2 (0.0)	-1.5 (0.1)	-1.3 (0.1)	-10.6	elst
al-CX HisE	Ph-5	COOH	-8.8 (0.8)	0.1 (0.0)	-1.2 (0.1)	-1.1 (0.1)	-10.9	elst
al-CX Ile	Ph-4	COOH	-8.0 (0.8)	0.3 (0.0)	-1.3 (0.1)	-1.6 (0.2)	-10.6	elst
al-CX Ile	Ph-5	COOH	-6.7 (0.8)	0.3 (0.0)	-1.2 (0.1)	-1.2 (0.1)	-8.9	elst
al-CX Lys	OH-1	NH2-1	8.3 (1.0)	0.7 (0.1)	0.1 (0.0)	-0.5 (0.1)	8.6	elst
al-CX Lys	OH-1	COOH	-12.9 (1.0)	1.7 (0.1)	-0.8 (0.1)	-0.9 (0.1)	-12.9	elst
al-CX Met	Ph-4	COOH	-11.3 (0.8)	0.2 (0.0)	-1.8 (0.1)	-1.0 (0.1)	-14.0	elst
al-CX Met	Ph-5	COOH	-8.1 (0.7)	0.3 (0.0)	-1.6 (0.2)	-1.4 (0.1)	-10.9	elst
al-CX Ser	Ph-3	COOH	-9.8 (0.9)	0.1 (0.0)	-1.1 (0.1)	-0.6 (0.1)	-11.5	elst
al-CX Thr	OH-2	COOH	9.7 (0.9)	2.5 (0.2)	0.3 (0.0)	-1.2 (0.1)	11.3	elst
al-CX Thr	Ph-3	COOH	-10.4 (0.8)	0.1 (0.0)	-1.4 (0.1)	-0.8 (0.1)	-12.5	elst
al-CX Trp	Ph-6	NH2	8.3 (0.9)	0.2 (0.0)	1.3 (0.1)	-0.3 (0.0)	9.4	elst
pc-CX AspH	OH-6	COOH-2	-8.3 (1.0)	0.5 (0.1)	-0.2 (0.0)	-0.5 (0.1)	-8.5	elst
pc-CX Cys	OH-4	COOH	-15.7 (1.1)	3.1 (0.2)	-0.7 (0.0)	-1.4 (0.1)	-14.8	elst
pc-CX Met	OH-1	S	-8.1 (0.9)	0.3 (0.0)	-0.2 (0.0)	-0.6 (0.1)	-8.6	elst
pc-CX Met	OH-3	COOH	-13.0 (1.0)	1.9 (0.1)	-0.5 (0.0)	-1.0 (0.1)	-12.6	elst
pc-CX Pro	Ph-4	COOH	-6.9 (0.9)	0.0 (0.0)	-0.9 (0.1)	-0.4 (0.0)	-8.0	elst
pc-CX Tyr	Ph-2	COOH	-9.2 (0.8)	1.4 (0.1)	-1.4 (0.1)	-2.1 (0.2)	-11.3	elst
pc-CX Tyr	Ph-3	COOH	-15.7 (0.9)	1.2 (0.1)	-1.4 (0.1)	-2.2 (0.1)	-18.1	elst
pc-CX Val	Ph-1	COOH	-8.8 (0.9)	0.1 (0.0)	-0.7 (0.1)	-0.6 (0.1)	-10.0	elst
pc-CX Val	Ph-2	COOH	-7.7 (0.8)	0.3 (0.0)	-1.0 (0.1)	-1.4 (0.1)	-9.8	elst
pc-CX Val	Ph-6	COOH	-7.0 (0.8)	0.7 (0.1)	-1.0 (0.1)	-1.7 (0.2)	-8.9	elst
wc-CX AspH	Ph-5	COOH-1	7.7 (0.9)	0.2 (0.0)	1.2 (0.1)	-0.4 (0.0)	8.7	elst
wc-CX Ser	Ph-3	COOH	-6.7 (0.8)	0.2 (0.0)	-1.2 (0.1)	-1.0 (0.1)	-8.6	elst
wc-CX Ser	OH-5	COOH	-10.3 (0.9)	0.4 (0.0)	-1.4 (0.1)	-0.6 (0.1)	-11.9	elst
wc-CX Thr	Ph-6	COOH	-6.6 (0.7)	0.2 (0.0)	-1.4 (0.2)	-1.2 (0.1)	-9.0	elst

Table S29: The F-SAPT partitioning for selected BCX+ amino acid pairs with their classification. Energies are in millihartree.

Complex	Group A	Group B	E_{elst} (ratio)	E_{exch} (ratio)	$E_{ind,eff}$ (ratio)	$E_{disp,eff}$ (ratio)	E_{total}	bond type
al-BCX Ala	OH-5	NH2	-39.5 (1.9)	43.5 (2.0)	-19.3 (0.9)	-6.0 (0.3)	-21.3	typical H-bond
al-BCX Asn	OH-5	NH2-1	-36.3 (1.8)	34.3 (1.7)	-13.2 (0.6)	-5.1 (0.3)	-20.4	typical H-bond
al-BCX Cys	OH-2	NH2	-45.8 (1.7)	48.9 (1.8)	-23.2 (0.9)	-6.4 (0.2)	-26.5	typical H-bond
al-BCX Gln	OH-5	CONH2	-16.9 (1.5)	15.7 (1.3)	-6.9 (0.6)	-3.5 (0.3)	-11.7	typical H-bond
al-BCX Gly	OH-2	NH2	-35.5 (1.7)	34.2 (1.6)	-15.0 (0.7)	-5.1 (0.2)	-21.3	typical H-bond
al-BCX HisE	OH-2	NH2	-39.3 (1.7)	39.1 (1.7)	-17.7 (0.8)	-5.6 (0.2)	-23.5	typical H-bond
al-BCX Leu	OH-5	NH2	-41.3 (1.9)	45.9 (2.1)	-20.4 (0.9)	-6.3 (0.3)	-22.2	typical H-bond
al-BCX Lys	OH-2	NH2-1	-48.3 (1.6)	47.9 (1.6)	-23.1 (0.8)	-6.5 (0.2)	-29.9	typical H-bond
al-BCX Met	OH-5	NH2	-40.0 (1.8)	42.5 (1.9)	-19.0 (0.8)	-6.0 (0.3)	-22.3	typical H-bond
al-BCX Phe	OH-5	NH2	-29.2 (1.6)	27.7 (1.5)	-12.3 (0.7)	-4.4 (0.2)	-18.1	typical H-bond
al-BCX Ser	OH-2	OH	-22.5 (2.0)	21.5 (1.9)	-7.2 (0.6)	-3.3 (0.3)	-11.5	typical H-bond
al-BCX Ser	OH-5	NH2	-44.7 (1.9)	48.4 (2.1)	-20.5 (0.9)	-6.6 (0.3)	-23.4	typical H-bond
al-BCX Trp	OH-2	NH2	-41.3 (1.6)	38.2 (1.5)	-17.4 (0.7)	-5.4 (0.2)	-25.9	typical H-bond
al-BCX Tyr	OH-5	NH2	-45.2 (2.0)	51.2 (2.2)	-22.0 (1.0)	-6.7 (0.3)	-22.7	typical H-bond
wc-BCX Ala	OH-6	NH2	-25.3 (1.7)	22.6 (1.5)	-8.3 (0.6)	-3.8 (0.3)	-14.8	typical H-bond
wc-BCX Asn	OH-3	NH2-1	-41.5 (1.9)	43.8 (2.0)	-17.7 (0.8)	-6.1 (0.3)	-21.5	typical H-bond
wc-BCX AspH	OH-2	COOH-1	-31.6 (1.5)	26.3 (1.3)	-11.1 (0.5)	-4.5 (0.2)	-20.8	typical H-bond
wc-BCX Cys	OH-3	NH2	-34.7 (1.8)	33.5 (1.7)	-13.5 (0.7)	-4.9 (0.3)	-19.7	typical H-bond
wc-BCX Gln	OH-2	COOH	-33.7 (1.7)	31.8 (1.6)	-13.3 (0.7)	-5.2 (0.3)	-20.3	typical H-bond
wc-BCX GluH	OH-2	COOH-2	-30.6 (1.7)	28.3 (1.5)	-11.4 (0.6)	-4.7 (0.3)	-18.5	typical H-bond
wc-BCX GluH	OH-5	COOH-1	-32.0 (1.7)	30.3 (1.6)	-12.4 (0.7)	-4.9 (0.3)	-19.0	typical H-bond
wc-BCX Gly	OH-6	NH2	-34.4 (1.8)	34.1 (1.8)	-13.8 (0.7)	-5.0 (0.3)	-19.1	typical H-bond
wc-BCX HisD	OH-3	NH2	-37.1 (1.9)	39.1 (2.0)	-15.9 (0.8)	-5.5 (0.3)	-19.3	typical H-bond
wc-BCX HisE	OH-6	NH2	-30.9 (1.7)	28.7 (1.6)	-11.3 (0.6)	-4.5 (0.2)	-18.0	typical H-bond
wc-BCX Ile	OH-3	NH2	-33.7 (1.7)	32.5 (1.7)	-13.2 (0.7)	-4.9 (0.3)	-19.3	typical H-bond
wc-BCX Leu	OH-3	NH2	-35.9 (1.8)	35.8 (1.8)	-14.7 (0.7)	-5.2 (0.3)	-19.9	typical H-bond
wc-BCX Lys	OH-2	COOH	-30.4 (1.5)	25.0 (1.2)	-10.9 (0.5)	-4.3 (0.2)	-20.7	typical H-bond
wc-BCX Lys	OH-3	NH2-2	-40.6 (1.9)	44.4 (2.1)	-18.9 (0.9)	-5.9 (0.3)	-21.0	typical H-bond
wc-BCX Ser	OH-6	NH2	-32.1 (1.7)	30.6 (1.6)	-12.6 (0.7)	-4.7 (0.2)	-18.8	typical H-bond
wc-BCX Thr	OH-3	NH2	-27.2 (1.7)	25.5 (1.6)	-10.3 (0.6)	-4.2 (0.3)	-16.1	typical H-bond
wc-BCX Trp	OH-6	NH2	-28.7 (1.7)	26.3 (1.5)	-10.5 (0.6)	-4.2 (0.2)	-17.1	typical H-bond
wc-BCX Val	OH-6	NH2	-33.8 (1.8)	32.6 (1.7)	-13.2 (0.7)	-4.9 (0.3)	-19.2	typical H-bond
al-BCX Asn	OH-2	CONH2	-27.2 (2.4)	34.3 (3.0)	-13.1 (1.1)	-5.5 (0.5)	-11.6	typical H-bond+disp
al-BCX GluH	OH-6	COOH-2	-20.5 (1.6)	18.4 (1.4)	-6.7 (0.5)	-4.0 (0.3)	-12.8	typical H-bond+dis
al-BCX Ile	OH-5	COOH	-19.3 (1.7)	19.0 (1.7)	-7.1 (0.6)	-3.8 (0.3)	-11.1	typical H-bond+dis
al-BCX Pro	OH-5	COOH	-17.5 (1.6)	16.3 (1.5)	-6.6 (0.6)	-3.4 (0.3)	-11.1	typical H-bond+dis
al-BCX Val	OH-5	COOH	-19.4 (1.8)	19.5 (1.8)	-7.2 (0.7)	-3.8 (0.3)	-10.9	typical H-bond+dis
pc-BCX Asn	OH-1	COOH	-15.5 (1.8)	15.6 (1.8)	-5.4 (0.6)	-3.4 (0.4)	-8.7	typical H-bond+dis
pc-BCX Asn	OH-2	COOH	-19.1 (2.1)	19.9 (2.2)	-6.2 (0.7)	-3.9 (0.4)	-9.2	typical H-bond+dis
pc-BCX Asn	OH-4	CONH2	-19.5 (1.6)	17.2 (1.4)	-6.4 (0.5)	-3.8 (0.3)	-12.5	typical H-bond+dis
wc-BCX Asn	OH-6	COOH	-11.9 (1.4)	11.5 (1.4)	-5.1 (0.6)	-2.8 (0.3)	-8.3	typical H-bond+dis
wc-BCX Gln	OH-3	CONH2	-28.0 (1.7)	28.7 (1.7)	-12.2 (0.7)	-5.4 (0.3)	-16.9	typical H-bond+dis
wc-BCX HisD	OH-6	Ring	-18.6 (1.6)	20.8 (1.8)	-8.7 (0.8)	-5.0 (0.4)	-11.6	typical H-bond+dis
wc-BCX Lys	OH-6	COOH	-10.7 (1.3)	9.8 (1.2)	-4.6 (0.6)	-2.6 (0.3)	-8.1	typical H-bond+dis
wc-BCX Pro	OH-6	Ring	-25.7 (1.8)	28.4 (2.0)	-11.0 (0.8)	-5.6 (0.4)	-13.9	typical H-bond+dis
al-BCX HisD	OH-6	Ring	-17.1 (1.3)	10.7 (0.8)	-3.4 (0.3)	-2.8 (0.2)	-12.7	H$\cdots\pi$
al-BCX Ser	Ph-5	COOH	-19.4 (0.9)	10.2 (0.5)	-5.8 (0.3)	-5.8 (0.3)	-20.8	H$\cdots\pi$
pc-BCX AspH	OH-3	COOH-1	-11.8 (1.2)	6.9 (0.7)	-2.7 (0.3)	-2.0 (0.2)	-9.6	H$\cdots\pi$
pc-BCX HisD	OH-1	COOH	-11.8 (1.1)	6.0 (0.6)	-2.7 (0.3)	-2.2 (0.2)	-10.6	H$\cdots\pi$
pc-BCX Tyr	Ph-4	OH	-10.2 (1.0)	5.5 (0.6)	-2.5 (0.3)	-2.8 (0.3)	-9.9	H$\cdots\pi$
wc-BCX Tyr	OH-2	COOH	-15.8 (1.4)	10.2 (0.9)	-3.3 (0.3)	-2.5 (0.2)	-11.4	H$\cdots\pi$
al-BCX HisE	Ph-5	COOH	-11.0 (0.9)	10.0 (0.8)	-4.7 (0.4)	-6.9 (0.5)	-12.6	disp
pc-BCX HisE	Ph-5	Ring	-13.4 (0.9)	10.6 (0.7)	-3.5 (0.2)	-8.8 (0.6)	-15.1	disp
wc-BCX HisE	Ph-5	Ring	-13.9 (0.9)	10.0 (0.7)	-2.3 (0.2)	-9.0 (0.6)	-15.1	disp
wc-BCX Trp	Ph-5	Ring	-12.1 (0.8)	10.7 (0.7)	-2.6 (0.2)	-10.8 (0.7)	-14.8	disp
al-BCX Ala	OH-6	COOH	-8.3 (0.9)	1.1 (0.1)	-0.4 (0.0)	-1.2 (0.1)	-8.7	elst
al-BCX Asn	Ph-4	COOH	-8.7 (0.8)	1.8 (0.2)	-1.1 (0.1)	-2.5 (0.2)	-10.5	elst
al-BCX AspH	Ph-4	COOH-2	-10.3 (0.8)	0.2 (0.0)	-1.8 (0.1)	-1.0 (0.1)	-13.0	elst
al-BCX AspH	Ph-5	COOH-1	-12.4 (0.8)	0.4 (0.0)	-1.4 (0.1)	-1.5 (0.1)	-14.9	elst
al-BCX AspH	Ph-5	COOH-2	-6.7 (0.7)	0.3 (0.0)	-1.6 (0.2)	-1.6 (0.2)	-9.6	elst
al-BCX Cys	Ph-3	COOH	-8.4 (0.8)	0.5 (0.0)	-0.3 (0.0)	-1.8 (0.2)	-10.0	elst
al-BCX Gln	OH-1	COOH	-11.8 (1.0)	2.1 (0.2)	-0.7 (0.1)	-1.0 (0.1)	-11.5	elst
al-BCX Gln-	Ph-5	CONH2	-11.1 (0.7)	0.5 (0.0)	-2.3 (0.2)	-2.0 (0.1)	-14.9	elst
al-BCX GluH	Ph-4	COOH-2	-10.1 (0.8)	0.3 (0.0)	-1.0 (0.1)	-1.5 (0.1)	-12.2	elst
al-BCX Gly	OH-2	COOH	9.1 (1.0)	0.5 (0.0)	0.4 (0.0)	-0.6 (0.1)	9.3	elst
al-BCX Gly	Ph-3	COOH	-6.7 (0.8)	0.1 (0.0)	-0.7 (0.1)	-0.8 (0.1)	-8.2	elst
al-BCX Gly	Ph-4	COOH	-21.3 (0.9)	5.5 (0.2)	-4.1 (0.2)	-3.7 (0.2)	-23.6	elst
al-BCX Gly	Ph-5	COOH	-7.7 (0.7)	0.8 (0.1)	-1.4 (0.1)	-2.1 (0.2)	-10.5	elst
al-BCX HisD	Ph-4	COOH	-9.1 (0.9)	0.1 (0.0)	-0.6 (0.1)	-1.0 (0.1)	-10.5	elst
al-BCX HisD	Ph-5	Ring	-9.0 (0.8)	1.0 (0.1)	-0.8 (0.1)	-2.7 (0.2)	-11.5	elst
al-BCX Ile	Ph-4	COOH	-11.2 (0.8)	0.3 (0.0)	-2.0 (0.1)	-1.4 (0.1)	-14.2	elst
al-BCX Ile	Ph-5	COOH	-6.4 (0.7)	1.1 (0.1)	-2.1 (0.2)	-2.2 (0.2)	-9.6	elst
al-BCX Pro	Ph-4	COOH	-12.5 (0.8)	0.2 (0.0)	-2.0 (0.1)	-1.2 (0.1)	-15.5	elst
al-BCX Pro	Ph-5	COOH	-9.7 (0.8)	0.3 (0.0)	-1.9 (0.2)	-1.5 (0.1)	-12.9	elst
al-BCX Ser	Ph-4	COOH	-13.5 (0.9)	0.8 (0.0)	-1.2 (0.1)	-1.8 (0.1)	-15.7	elst
al-BCX Ser	OH-5	COOH	9.2 (1.0)	0.0 (0.0)	0.8 (0.1)	-0.5 (0.0)	9.5	elst
al-BCX Thr	Ph-3	COOH	-8.8 (0.9)	0.1 (0.0)	-0.2 (0.0)	-0.7 (0.1)	-9.6	elst
al-BCX Val	Ph-4	COOH	-11.2 (0.8)	0.3 (0.0)	-1.9 (0.1)	-1.3 (0.1)	-14.1	elst
al-BCX Val	Ph-5	COOH	-6.9 (0.7)	1.0 (0.1)	-2.1 (0.2)	-2.2 (0.2)	-10.2	elst
pc-BCX Ala	Ph-5	COOH	-8.4 (0.9)	2.0 (0.2)	-1.0 (0.1)	-2.4 (0.2)	-9.8	elst
pc-BCX Asn	Ph-5	CONH2	13.0 (1.0)	0.2 (0.0)	1.1 (0.1)	-0.8 (0.1)	13.4	elst
pc-BCX AspH	Ph-4	COOH-1	-10.7 (0.8)	0.1 (0.0)	-1.5 (0.1)	-0.9 (0.1)	-12.9	elst
pc-BCX AspH	Ph-4	COOH-2	-7.2 (0.8)	0.8 (0.1)	-0.8 (0.1)	-1.7 (0.2)	-8.9	elst
pc-BCX Gln	Ph-5	COOH	-11.3 (0.8)	2.5 (0.2)	-1.6 (0.1)	-3.1 (0.2)	-13.5	elst
pc-BCX GluH	Ph-3	COOH-1	-15.4 (0.8)	2.3 (0.1)	-2.2 (0.1)	-3.2 (0.2)	-18.5	elst
pc-BCX HisD	OH-2	COOH	8.6 (0.9)	1.4 (0.1)	0.5 (0.1)	-0.8 (0.1)	9.7	elst
pc-BCX HisD	OH-4	COOH	-8.8 (0.9)	0.0 (0.0)	-0.7 (0.1)	-0.2 (0.0)	-9.7	elst
pc-BCX Met	Ph-6	COOH	-13.6 (0.8)	2.0 (0.1)	-2.3 (0.1)	-3.0 (0.2)	-17.0	elst
pc-BCX Pro	Ph-3	COOH	-9.7 (0.9)	0.3 (0.0)	-0.6 (0.1)	-1.1 (0.1)	-11.2	elst
pc-BCX Pro	Ph-4	COOH	-8.3 (0.9)	0.0 (0.0)	-1.0 (0.1)	-0.4 (0.0)	-9.7	elst
wc-BCX Ala	Ph-3	COOH	-14.4 (0.8)	3.7 (0.2)	-2.9 (0.2)	-3.6 (0.2)	-17.2	elst
wc-BCX Asn	OH-2	COOH	-6.9 (0.9)	0.4 (0.1)	-0.9 (0.1)	-0.7 (0.1)	-8.1	elst
wc-BCX Gln	Ph-3	CONH2	-8.5 (0.7)	0.5 (0.0)	-1.9 (0.2)	-1.8 (0.2)	-11.7	elst
wc-BCX HisD	Ph-5	Ring	10.5 (0.9)	0.2 (0.0)	2.5 (0.2)	-1.2 (0.1)	11.9	elst
wc-BCX Met	Ph-3	COOH	-11.1 (0.8)	2.1 (0.1)	-2.2 (0.2)	-3.0 (0.2)	-14.1	elst
wc-BCX Pro	Ph-3	COOH	-11.7 (0.8)	2.4 (0.2)	-2.3 (0.2)	-3.2 (0.2)	-14.8	elst
wc-BCX Thr	Ph-6	COOH	-13.0 (0.8)	2.8 (0.2)	-2.8 (0.2)	-3.3 (0.2)	-16.3	elst

Table S30: Interaction of hydroxy groups of the BCX calixarene with and without the presence of Gly as calculated with the I-SAPT method. Energies are in millihartree.

OH#	OH#	*al*-BCX Gly	*al*-BCX	*pc*-BCX Gly	*pc*-BCX	*wc*-BCX Gly	*wc*-BCX
1	2	-13.2	-10.9	-13.1	-12.3	-11.1	-9.3
1	3	-0.3	-0.5	-1.7	-1.6	0.0	0.0
1	4	-0.3	-0.4	-0.9	-1.0	-0.1	0.0
1	5	-0.2	-0.7	-0.1	-3.2	-0.4	-0.3
1	6	-16.0	-11.4	-4.2	-15.1	-10.2	-9.7
2	3	-1.4	-0.1	-15.2	-14.3	-1.7	-1.2
2	4	-0.6	-0.2	-3.6	-3.2	-0.3	-0.3
2	5	0.4	0.4	3.3	2.1	0.7	0.0
2	6	-6.5	-4.0	-5.1	-5.7	-3.0	-2.7
3	4	-10.6	-11.6	-17.3	-15.1	-10.8	-9.6
3	5	-2.8	-2.9	-6.3	-5.7	-5.2	-2.7
3	6	0.1	-0.3	-0.4	-1.5	1.1	1.2
4	5	-10.5	-10.5	-10.5	-12.3	-13.5	-9.3
4	6	-0.2	-0.3	0.0	-1.6	-0.2	0.0
5	6	-0.8	-1.8	-11.8	-14.3	-3.3	-1.2
